# Protein-protein interactions reveal key canonical pathways, upstream regulators, interactome domains, and novel targets in ALS

**DOI:** 10.1038/s41598-018-32902-4

**Published:** 2018-10-03

**Authors:** Ina Dervishi, Oge Gozutok, Kevin Murnan, Mukesh Gautam, Daniel Heller, Eileen Bigio, P. Hande Ozdinler

**Affiliations:** 1 Department of Neurology, Northwestern University, Feinberg School of Medicine, Chicago, USA; 2 Mesulam Cognitive Neurology and Alzheimer Disease Center, Chicago, IL 60611 USA; 3 Department of Pathology, Northwestern University, Chicago, IL 60611 USA; 4 Robert H. Lurie Comprehensive Cancer Center, Northwestern University, Feinberg School of Medicine, Chicago, IL 60611 USA; 5 Les Turner ALS Center, Northwestern University, Feinberg School of Medicine, Chicago, IL 60611 USA

## Abstract

Developing effective treatment strategies for neurodegenerative diseases require an understanding of the underlying cellular pathways that lead to neuronal vulnerability and progressive degeneration. To date, numerous mutations in 147 distinct genes are identified to be “associated” with, “modifier” or “causative” of amyotrophic lateral sclerosis (ALS). Protein products of these genes and their interactions helped determine the protein landscape of ALS, and revealed upstream modulators, key canonical pathways, interactome domains and novel therapeutic targets. Our analysis originates from known human mutations and circles back to human, revealing increased PPARG and PPARGC1A expression in the Betz cells of sALS patients and patients with TDP43 pathology, and emphasizes the importance of lipid homeostasis. Downregulation of YWHAZ, a 14-3-3 protein, and cytoplasmic accumulation of ZFYVE27 especially in diseased Betz cells of ALS patients reinforce the idea that perturbed protein communications, interactome defects, and altered converging pathways will reveal novel therapeutic targets in ALS.

## Introduction

Understanding the cellular and molecular basis of selective vulnerability has proven to be difficult, and yet it remains one of the most important challenges for building effective treatment strategies for neurodegenerative diseases, in which distinct neuron populations display early signs of vulnerability and undergo progressive degeneration.

To date many gene mutations are detected in amyotrophic lateral sclerosis (ALS) patients, and these are characterized as “causative” and “associated” with the disease, or are “disease modifying”^1–3^. (Table 1) It is interesting that mutation in one gene would lead to a distinct motor neuron disease, and that mutations in different, and at times unrelated, genes would lead to the same pathology^4–6^. Identification genes that are within the same biological pathway began to suggest the presence of converging paths. The heterogeneity among patient populations could indeed be due to the interplay between converging and diverging paths, which are not well defined. Genetics offer an important source for understanding the intricate balance between mutation and pathology. Developments in the fields of human genetics, protein detection systems as well as protein interaction assays, and large data management applications have begun to offer a unique opportunity to link genes with proteins and protein interaction networks^7^ to reveal the intricate cellular balance that becomes perturbed in the presence of a single mutation. Upon identification of numerous genes that are linked and associated with ALS, revealing the biological link between mutations and neuronal vulnerability has recently became a possibility^8–11^.

**Table 1:** List of genes that are reported to be “*Causative”*, “*Associated” and “Disease Modifier” with* respect to ALS disease.

Causative Genes*
ALS2, ALS3, ALS7, ANG, ANXA11, ATXN2, CFAP410, C9orf72, CHCHD10, CHMP2B, DAO, DCTN1, ELP3, ERBB4, Erlin1, FIG4, FUS, HNRNPA1, LMNB1, MATR3, NEFH, NEK1, OPTN, PFN1, PRPH, SETX, SIGMAR1, SOD1, SPAST, SPG11, SQSTM1, TAF15, TARDBP, TIA1, TUBA4A, UBQLN2, UNC13A, VAPB, VCP.
Associated and Disease Modifier Genes*:
AGT, ALAD, APEX1, APOE, AR, ARHGEF28, ATRN, ATXN1, B4GALT6, BCL6, BCL11B, BIRC6, C1orf27, C1QTNF7, CCNF, CCS, CDH13, CDH22, CHGB, CNTF, CNTN4, CNTN6, CREB3L2, CRIM1, CRYM, CSNK1G3, CST3, CX3CR1, CYP2D6, DCC, DIAPH3, DISC1, DOC2B, DPP6, DYNC1H1, EFEMP1, EPHA4, EWSR1, FEZF2, FGGY, GARS, GLE1, GRB14, GRN, HEXA, HFE, HNRNPA2B1, ITPR2, KCNN1, KDR, KIF5A, KIFAP3, LIF, LIPC, LOX, LUM, MAOB, MAPT, MOBP, MT-ND2, NAIP, NETO1, NIPA1, NT5C1A, OGG1, OMA1, PARK7, PCP4, PEAK1, PLEKHG5, POLDIP2, PON1, PON2, PON3, PSEN1, PVR, RAMP3, RBMS1, RNASE2, RNF19A, SARM1, SCFD1, SCN7A, SELL, SEMA6A, SLC1A2, SLC39A11, SMN1, SMN2, SNCG, SOD2, SOX5, SPG7, SS18L1, STX12, SUSD1, SYNE, SYT9, TBK1, TRPM7, VDR, VEGFA, VPS54, WDR49, ZFP64, ZNF746, ZNF512B, ZSCAN5B.

*Please see Supplementary Table 1 for references.

Current findings support the hypothesis that the main underlying factor accounting for neuronal vulnerability is perturbed cellular homeostasis and the lack of the neuron’s ability to perform its key function or functions^8–12^. Mutations that result in selective neuronal vulnerability imply that protein encoded by that mutated gene has a role that is indispensable for the vulnerable neuron. Even though other cells and neurons carry the same mutation, they may not feel the same burden. Therefore, mutations detected in patients have immense value for uncovering the path from genes to proteins and then from proteins to protein-protein interaction domains. We suggest that this path reveals the most crucial canonical pathways and cellular events that are indispensable for the function and the health of the degenerating neurons.

Here, we focus our attention to ALS, a disease of the motor neuron circuitry, in which corticospinal and spinal motor neuron populations progressively degenerate^13–15^. To date, 147 different mutations have been identified in ALS patients and 39 have been accepted to be causative with strong correlation and 108 of them have been accepted to be associated with the disease or are modifying disease initiation and progression, by increase the chance of developing ALS. At first sight these genes may appear unrelated, but some are within the same cellular pathways, such as axonal transport defects, unfolded protein response, and ER stress, suggesting that these cellular events are important for the proper function of the motor neuron circuitry^3,4,6^.

Realization of the fact that each human mutation actually reveals an important information about the system that collapses in patients, and that each cellular event is a result of orchestrated actions of proteins that work within interactome domains, motivated us to reveal the protein interaction landscape in ALS, to understand how proteins are related to each other and what that means for maintaining the health of the motor neuron circuitry. We thus determined the list of ALS proteins, proteins that bind to the protein product of the mutated genes associated with and linked to ALS. Our study helps transform the knowledge of human mutations into protein landscape of ALS, and uncovers how these proteins interact, which canonical pathways they are primarily involved in, and more importantly, it begins to suggest how different gene mutations converge into significant perturbations in protein interaction domains, which could in fact be novel therapeutic targets.

## Materials and Methods

### Post-mortem human brain samples

Postmortem human tissue was collected according to protocols approved by an institutional review board of Northwestern University’s Institutional Review Board. Clinical records were available for every subject, and informed consents were obtained from each patient. A neuropathologist with expertise in neurodegenerative disorders examined all samples. Brains were fixed either in 10% neutral buffered formalin for 2 weeks or 4% paraformaldehyde (PFA) at 4 °C for 30 h, and sections were paraffin embedded. Areas of the primary motor cortex (Brodmann area 4) were retrieved, 4 μm thick serial sections were cut, mounted on a charged glass slide (Fisher Scientific), and used for immunocytochemical analyses. ALS patients positive for TDP-43 pathology were included in the study. Presence of TDP43 pathology was confirmed by immunostaining with an antibody to phosphorylated TDP43 (monoclonal TDP43 antibody pS409/410-2, 1:5000 dilution, AEC chromogen (Cosmo-Bio USA, Carlsbad CA) (Supplementary Fig. 1). Cortical layer 5 of motor cortex was evaluated for TDP43 immunoreactivity in neuronal cytoplasmic inclusions (NCIs), glial/microglial cytoplasmic inclusions (GCIs) and extracellular dystrophic neurites (DNs). In this study a total of 12 control cases (*n* = 3 female, *n* = 9 male) and a total of 8 sALS cases (*n* = 3 female, *n* = 5 male) and 9 ALS cases with confirmed TDP43 pathology (*n* = 2 females, *n* = 7 males) are used. A more detailed explanation of patients can be found in Table 2. Both males and females analyzed together as no sex differences were observed at a cellular level.

**Table 2:** List of post-mortem human cases included in this study. The table shows Clinical-Pathological diagnosis, age, sex, PMI/h, and the type of TDP43 inclusions observed in patients. NCIs: neuronal cytoplasmic inclusions; GIs: glial/microglial cytoplasmic inclusions; DNs: extracellular dystrophic neurites; PMI/h: post-mortem interval in hrs.

Clinical-Pathological Diagnosis	Case #	Age	Sex	PMI/h	Type of TDP-43 inclusion		
					TDP + NCIs	TDP + GIs	TDP + DNs
ALS with TDP43	1	61	M	29	+	0	0
ALS with TDP43	2	64	M	24	+	++	+
ALS with TDP43	3	61	M	13	+	0	0
ALS with TDP43	4	64	M	14	0	+	0
ALS with TDP43	5	64	F	19	+	+	0
ALS with TDP43	6	73	M	23	0	+	0
ALS with TDP43	7	57	M	18	+	+++	0
ALS with TDP43	8	40	M	19	+	++	+
ALS with TDP43	9	82	F	36	++	0	0
Sporadic ALS	10	66	F	12			
Sporadic ALS	11	71	F	15			
Sporadic ALS	12	64	F	9			
Sporadic ALS	13	55	M	16			
Sporadic ALS	14	53	M	6			
Sporadic ALS	15	51	M	19			
Sporadic ALS	16	63	M	21			
Sporadic ALS	17	61	M	17			
Normal Control	18	66	F	18			
Normal Control	19	62	F	15			
Normal Control	20	54	M	12			
Normal Control	21	64	M	10			
Normal Control	22	63	M	8			
Normal Control	23	59	M	12			
Normal Control	24	69	M	12			
Normal Control	25	60	M	19			
Normal Control	26	72	M	14			
Normal Control	27	45	F	15			
Normal Control	28	67	M	5			
Normal Control	29	74	M	8			

### Determination of ALS protein pool and data analysis

To compile the list of mutations that are either causative or associated with ALS (*n* = 147 genes), previously published results and open public resources and databases are used (i.e. http://www.omim.org/, http://www.ncbi.nlm.nih.gov/pubmed, ALSoD (http://alsod.iop.kcl.ac.uk) and ALS Gene Databases (ALSgene.org)). Here we included genes that were reported up until January 2018. To determine the list of all proteins that bind to the protein product of mutated genes, previously published results with experimental outcomes were used and ingenuity pathway analysis (IPA) [QIAGEN Comp, LA, USA] was utilized. IPA is a toolbox that incorporates previously published knowledge domain for large data analysis. The binding partners for the protein product of each mutated gene related to ALS were determined by very stringent inclusion criteria: only findings that are only experimentally observed for direct binding with a published report, findings that are obtained from neurons and mammalian systems were included. Results obtained from non-mammalian systems, cancer cell lines, proteins that do not directly interact but are found within the same complex and curated findings were excluded. Only results with a published record of experimentally observed findings were selected. Setting stringent criterion limited the numbers of proteins included in the study, but increased confidence levels in future analysis. Please see Supplementary Table 2 with a list of all protein products of ALS related genes, the protein binding partners determined by experimentation, and the references associated with these findings. Proteins with 3 or more binding partners are marked bold.

### Immunocytochemistry

#### Post-mortem Human samples

Slides were baked for 60 min at 60 °C, deparaffinized with xylene for 5 min, rehydrated in ethanol (100%, 95%, 70%, and 50%). For antigen retrieval, slides were immersed in 10 mM sodium citrate and subjected to high heat and pressure for 20 min. After cooling, slides were rinsed with PBS for 10 min, and 3% H_2_O_2_ for 10 min. Slides were blocked in normal goat serum (ABC Kit, Vector Laboratories, Inc.) and 0.3%Triton x-100 in PBS and incubated overnight at 4 °C with primary antibodies: PPAR gamma (Bioss, catalogue no. bs-4590R, 1:200, Woburn, MA), PPARGC1A (Proteintech, catalogue no. 66369-1-Ig, 1:200, Rosemont, IL). YWHAZ (Bioss, catalogue no. bsm-215397M, 1:200, Woburn, MA), ZFYVE27 (Bioss, catalogue no. bs-11777R, 1:200, Woburn, MA). After PBS rinses, slides were incubated with biotinylated secondary antibodies diluted in the blocking solution for 2 hours at RT. The slides were developed using ImmPACT^TM^ DAB chromogen in ImmPACT^TM^ DAB diluent (ImmPACT^TM^ DAB peroxidase substrate kit, catalogue no. SK-410, Burlingame CA) and counterstained with hematoxylin. Slides were then dehydrated in ethanol (50%, 70%, 95%, 100%), immersed in xylene and coverslipped with DEPEX mounting medium (Electron microscopy sciences, cat# 13514, Hatfield, PA).

### Data acquisition and imaging

Nikon SMZ1500 and Nikon Eclipse TE2000-E fluorescence microscopes equipped with Intensilight C-HGFI (Nikon, Inc.) were used. Epifluorescence images were acquired using a Digital Sight DS-Qi1MC CCD camera (Nikon, Inc.) and light images were acquired using a Ds-Fi1 camera (Nikon, Inc.).

### Statistical Analysis

Ingenuity Pathway Analysis (IPA) uses an array of statistical analysis to determine whether the analyzed data set has significant coverage with any of the previously determined canonical pathways, cellular events, protein-protein interaction domains and pathways. (https://www.qiagenbioinformatics.com/products/ingenuity-pathway-analysis). Statistical data were analyzed through the use of IPA, which mainly uses Fisher’s exact test. In summary, the significance value associated with functional analysis of a dataset is a measure of the likelihood that the association between the experimental group (i.e. ALS proteins) and the given pathway is due to random chance or not. For the IPA analysis, *the ratio* is calculated by taking the number of genes from ALS protein list that participate in a Canonical Pathway, and dividing it by the total number of genes in that Canonical Pathway. The ratio is therefore useful for determining which pathways overlap the most with the ALS protein list. The *p-value* measures how likely the observed association between a specific pathway and the dataset would be if it was only due to random chance. p < 0.05 or (−log p-value = 1.3) is considered significant and that the ratio of ALS proteins in that canonical pathway cannot be explained by randomness. The p-value is calculated by considering: 1) the number of functions/pathways that participate in the cellular event; 2) the total number of molecules in the ALS protein list known to be associated with that pathway; 3) the total number of molecules in the selected reference set. The p-value calculation depends on the statistical null model, such as the “random” model. Fisher’s exact test is used to determine the likelihood of randomness. The *threshold line* that appears in the bar charts represents a p-value of 0.05. The *p-value of overlap* indicates the statistical significance of proteins in the dataset that are downstream of the upstream regulator. It compares the proportion of ALS proteins that are associated with a particular biological attribute to the proportion of molecules that are expected to emerge if the dataset was made up of randomly selected molecules. It is calculated using the right-tailed Fisher’s Exact Test. The p-value of overlap calculation only takes into account the exact number and types of proteins, but not their ‘direction” of expression values. The activation *z-score* predicts the activation state of the upstream regulator, using the expression patterns of the genes/proteins that are downstream of an upstream regulator. The z-score calculation needs a minimum of 4 targets with an expected expression pattern. An absolute z-score of ≥2 is considered significant. An upstream regulator is “Activated” if the z-score is ≥2 and “Inhibited” if the z-score ≤2.

Betz cells with high and low levels of protein expression were counted and average percentages of Betz cells with high level of protein expression was also quantified. Slides which harbored similar and comparable regions of the motor cortex were used for immunocytochemical analysis. Betz cells, located in layer V of the motor cortex with large and pyramidal cell body were identified and counted based on their level of protein expression. Betz cells expressing PPARG (control: 47; sALS: 31; TDP43: 36), PPARGCA1 (control: 37; sALS: 24; TDP43: 27), YWHAZ (control: 160; sALS: 77; TDP43: 78), and ZFYVE27 (control: 106; sALS: 63; TDP43: 74) were counted in control (*n* = 12), sALS (*n* = 8) and TDP43 (*n* = 9) cases investigated, and the average number of Betz cells with high level of protein expression was determined for each case. All statistical analyses were performed using Prism software (version 5a; Graphpad Software Inc.La Jolla, CA). Statistical differences were determined by student’s t-test and one-way analysis of variance (ANOVA) with Tukey’s *post hoc* test after D’Agostino & Pearson normality test was performed on all data sets. Statistically significant differences were considered at least *p* < 0.05 and values were expressed as the independent mean ± standard error of the mean (SEM).

## Results

### Mutations in ALS patients help reveal the protein interactome for ALS

To date, mutations in many genes are reported to cause ALS (*n* = 39; eg*. FUS, SOD1, SQSTM1, TARDBP, UBQLN2, VCP*), are associated with the disease, or act as a disease modifier (*n* = 108; eg. *SPASTIN, KIFAP3, CNTN6, PON3;* Table 1, Supplementary Table 1). The protein products of these mutated genes are known, and numerous published studies investigated their binding partners either by yeast-two hybrid assays, immune-precipitation coupled with proteomic studies, or other protein-protein interaction assays in different experimental system (Supplementary Table 2).

**Figure 1:**
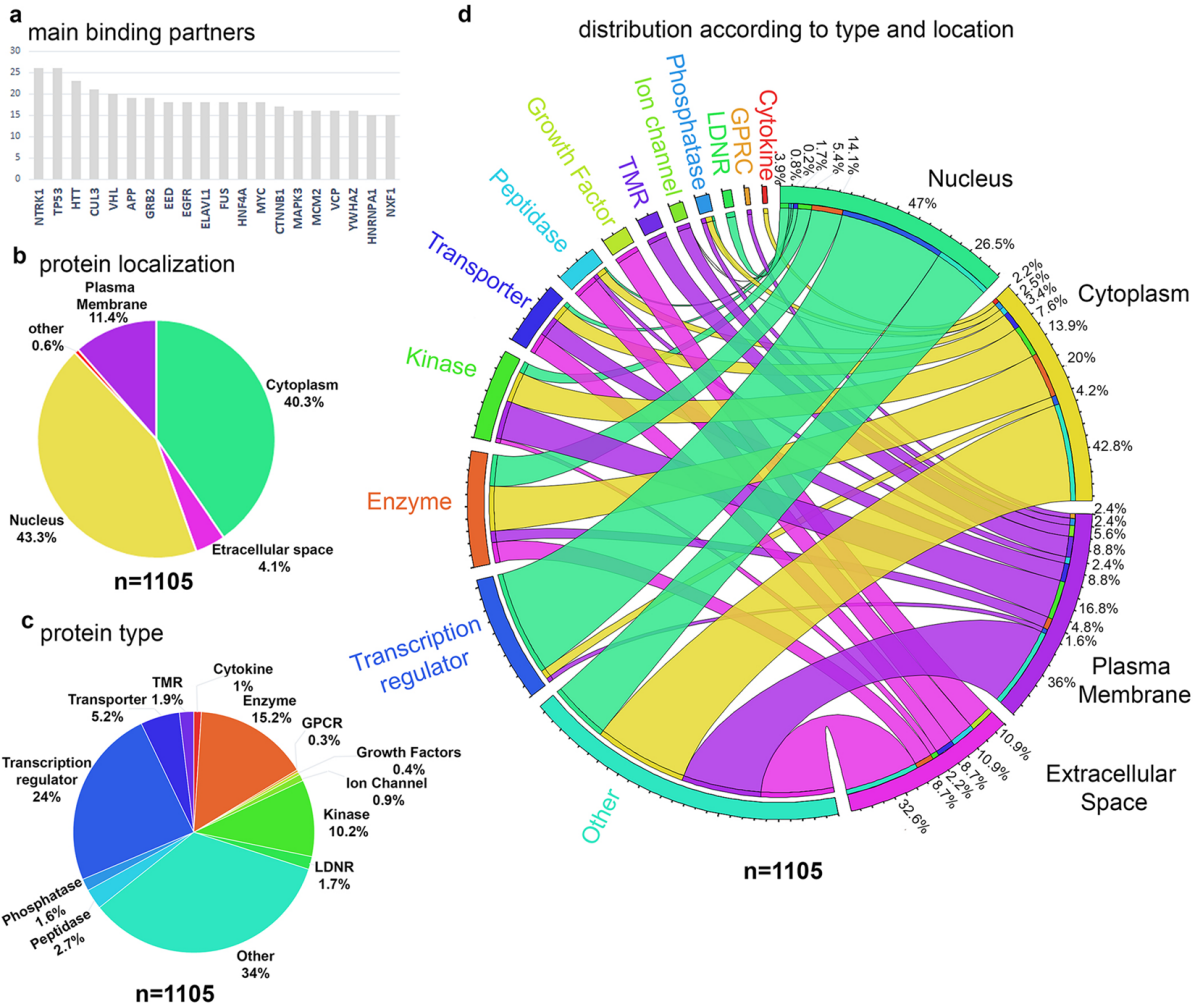
Distribution of ALS proteins based on location and type. (**a**) Bar graph representing the top binding partners ranked based on the number of interactions they have with protein products of genes linked and associated with ALS. (**b,c**) Pie charts show the distribution of ALS proteins based on their cellular location (**b**) and protein type (**c**,**d**) the circular plot shows the relationship between location and type of ALS proteins.

In an effort to identify proteins that are proven binding partners of the protein products of these mutated genes, we utilized Ingenuity Pathway Analysis (IPA) with a very stringent inclusion criteria. Only the experimentally observed results, proteins with experimental proof of direct binding, and results obtained from mammalian systems or from neurons were included. All data obtained from non-mammalian systems or cell lines that are not related to neurons, such as non-neuronal cells and cancer cell lines were excluded. This stringent inclusion criteria resulted in the identification of proteins that are known to bind to the protein of interest with a convincing experimental data. Proteins were thus numbered based on the times they appear as a binding partner for the protein product of the mutated genes in ALS patients (Supplementary Table 3). The main binding partners were identified based on the number of direct interaction they have with the protein products of genes that are either linked or associated with ALS. It was remarkable to note that some of the protein products of ALS related genes were also detected as frequent binding partners, such as FUS (*n* = 18), HNF4A (*n* = 18), VCP (*n* = 16), HNRNPA1 (*n* = 15; Fig. 1a). To increase stringency, a cutoff of 3 was applied, and 1105 proteins were selected. We will refer those 1105 proteins, “ALS proteins” throughout the text. (See Supplementary Table 3, for all ALS proteins, their associated binding partner number, location, and type).

43.4% of ALS proteins were in the nucleus, 40.3% in the cytoplasm, 11.4% in the cellular membrane, and 4.1% of them were present in the extracellular space (Fig. 1b). 24% were playing a role in transcriptional regulation, 15.2% were an enzyme, 5.2% a transporter, 10.2% a kinase, and only 1.9% were a transmembrane receptor (TMR). 34% were grouped under the category “other” because they either had multiple roles or unknown function (Fig. 1c), Circular diagram (Fig. 1d) integrated the location and the type information, revealing that 2.2% of proteins present in the extracellular space were a kinase, 16.1% a transporter, and 6.1% a peptidase. Majority of cytoplasmic proteins (42.8%) had dual functions, and 13.9% were a kinase, 20% an enzyme, 7.6% a transporter, 2.5% a phosphatase, and 3.4% a peptidase. Among proteins present in the plasma membrane, 8.8% were a TMR, 5.6% an ion channel, 16.8% a kinase, 8.8% a transporter, and 4.8% an enzyme. A large percentage of proteins in the nucleus were important for transcriptional control (47%), and 14.1% were an enzyme, 5.4% a kinase, 1.7% a phosphatase, 0.2% a peptidase, 0.8% a transporter, and 3.9% an LDNR.

**Figure 2:**
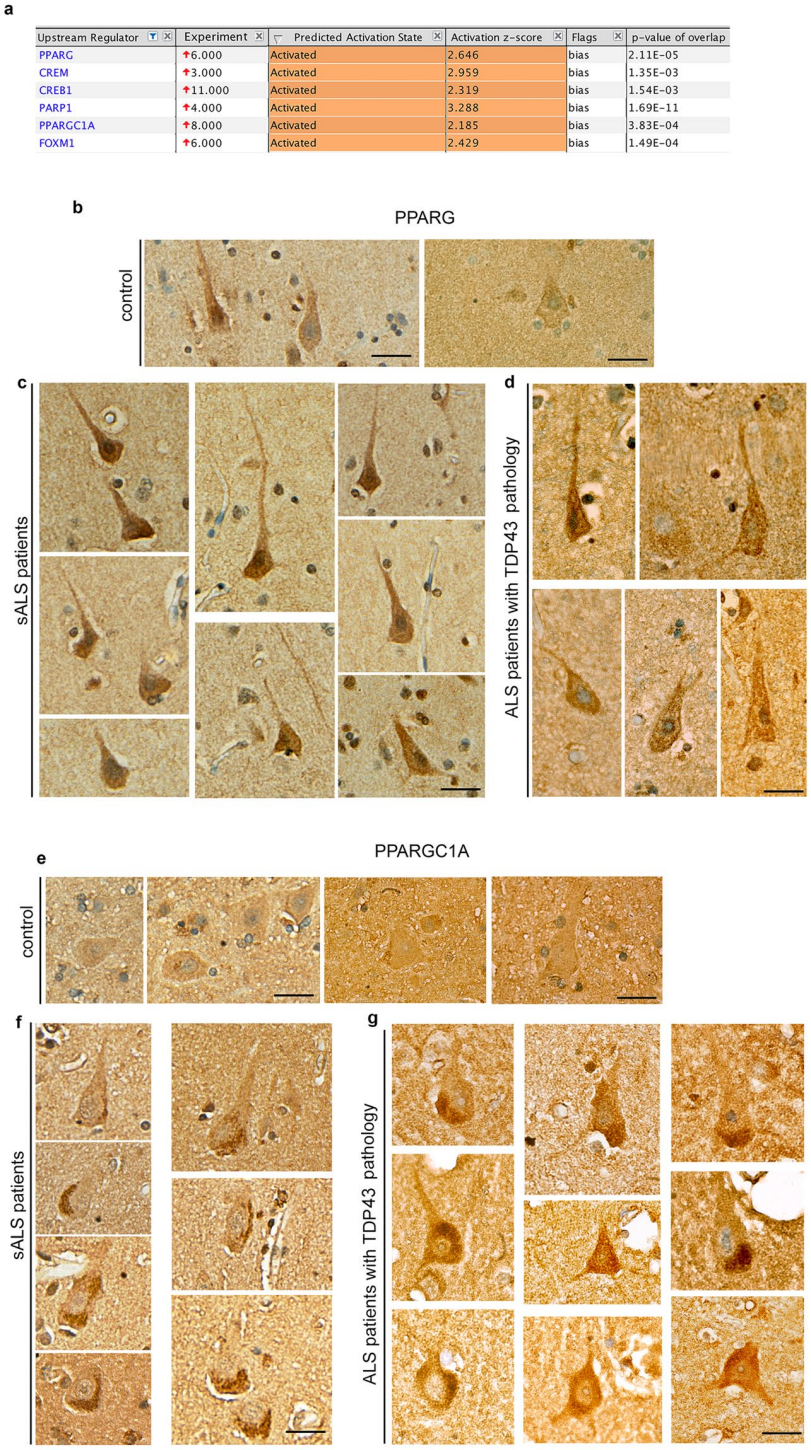
Upstream regulators for ALS proteins. (**a**) Table of upstream regulators, the number of their binding partners, their predicted activation state and score, and p-value of overlap. (**b**) Representative image of PPARG expression in the Betz cells of control cases (*n* = 12). PPARG is detected in the cytoplasm and in some cases in the nucleus. (**c**) Representative images of PPARG expression in the Betz cells of sporadic ALS patients (*n* = 8). (**d**) Representative images of PPARG expression in the Betz cells of ALS patients with TDP43 pathology (*n* = 9). (**e**) Representative image of PPARGC1A expression in the Betz cells of control cases (*n* = 12). PPARG is low but is detected in the cytoplasm. (**f**) Representative images of PPARGC1A expression in the Betz cells of sporadic ALS patients (*n* = 8). (**g**) Representative images of PPARGC1A expression in the Betz cells of sporadic ALS patients (*n* = 8). PPARGC1A expression is increased and the proteins is accumulated in the cytoplasm, z-score ≥2 and p ≥ 0.05 is significant. Scale bar = 50 μm in b-g.

An initial “core analysis” was performed with direct relationships, 35 molecules per network, 25 networks per analysis, confidence set to experimentally observed findings, and species restricted to mammals only. This was one of the most strict analysis option. We first asked for the presence of upstream regulators among ALS proteins (Fig. 2). 6 proteins were suggested to be upstream regulators with bias, because the proteins they act upon (e.g. modulate activation, expression, determination of location) were present among ALS proteins that cannot be explained by sheer luck and that based on the list of ALS proteins analyzed these 6 proteins were on top of the hierarchical order, and were most likely (estimated by z-score and p value of overlap) acting as the upstream regulator. The list included proteins such as CREM (z-score: 2.959; p-value of overlap: 1.35E-5), CREB1 (z-score: 2.319; p-value of overlap: 1.54E-3) and FOXM1 (z-score: 2.429; p-value of overlap: 1.48E-4), all of which were transcriptional regulators. Interestingly, another group of proteins that are particularly important for lipid biosynthesis and homeostasis, such as PPARG (z-score: 2.646; p-value of overlap: 2.111E-5), PARP1 (z-score: 3.288; p-value of overlap: 1.69E-11), and PPARGC1A (z-score: 2.185; p-value of overlap: 3.83E-4) were predicted with bias that they played a significant role in the activation of downstream effectors (Fig. 2a).

To investigate whether proteins involved in lipid biosynthesis and that are highlighted as potential modulators of ALS are expressed in Betz cells of ALS patients, and whether their presence is altered with respect to disease, we investigated the expression profiles of PPARG (Fig. 2b,c) and PPARGC1A (Fig. 2d,e) in the motor cortex of control cases (*n* = 12), as well as sALS patients with no known mutations (*n* = 8), and ALS patients with TDP43 pathology (*n* = 9). PPARG was present in the cytoplasm of Betz cells at low levels in the control cases. However, PPARG expression was prominently increased in the Betz cells of both sALS patients and ALS patients with TDP43 pathology (average percentage of Betz cells with increased PPARG expression: control: 22 ± 3; sALS: 83 ± 5; TDP43: 74 ± 12). The results were comparable between sALS and patients with TDP43 pathology with no significance (p = 0.4289) However, both sALS (p = 0.0001; Fig. 2c) and TDP43 (p = 0.0001; Fig. 2d) cases had significantly higher numbers of Betz cells with increased PPARG expression. Similar to PPARG, the presence of PPARGC1A protein was evident in the Betz cells of control cases (*n* = 12), albeit at low levels (Fig. 2e), and was increased in the Betz cells of sALS patients and ALS patients with TDP43 pathology (average percentage of Betz cells with increased PPARGC1A expression: control: 27 ± 6; sALS: 82 ± 3; TDP43: 81 ± 4; Fig. 2e). Different from PPARG, PPARGC1A was not present throughout the cytoplasm, but rather displayed enlarged cytoplasmic inclusions with different sizes and intensities only in the Betz cells of all sALS patients. Other cells and neurons present in the motor column expressed low levels of PPARGC1A, and did not have large accumulations, similar to that of Betz cells.

### ALS proteins take part in a distinct set of canonical pathways

Since three important proteins for the lipid biosynthesis were suggested to be upstream and that both PPARG and PPARGC1A expression were upregulated in 17 different and unrelated ALS cases, we next investigated whether ALS protein distribution among cellular events that are related to lipid homeostasis were also significant, as assessed with increased ratio and p-value. The ratio is determined by the total number of proteins among ALS proteins that are common with the proteins of a given canonical pathway. Therefore, the higher the representation within a canonical pathway, the higher the ratio. Likewise, the p-value suggests whether this ratio could indeed be due to sheer luck or due to significant association between proteins and the given canonical pathway. p < 0.05 is considered significant and that the ratio of ALS proteins in that canonical pathway cannot be explained by random distribution. The smaller the p value, the more likely that this association is indeed a strong correlation that cannot be explained by luck. The p-values are determined both by Fisher Exact test and Benjamin-Hochberg method of multiple testing correction, both resulted in comparable findings. The figures report the p-values based on the Fisher Exact test and the comparative values between two tests can be found in Supplementary Table 4.

#### Lipid Homeostasis

**Figure 3:**
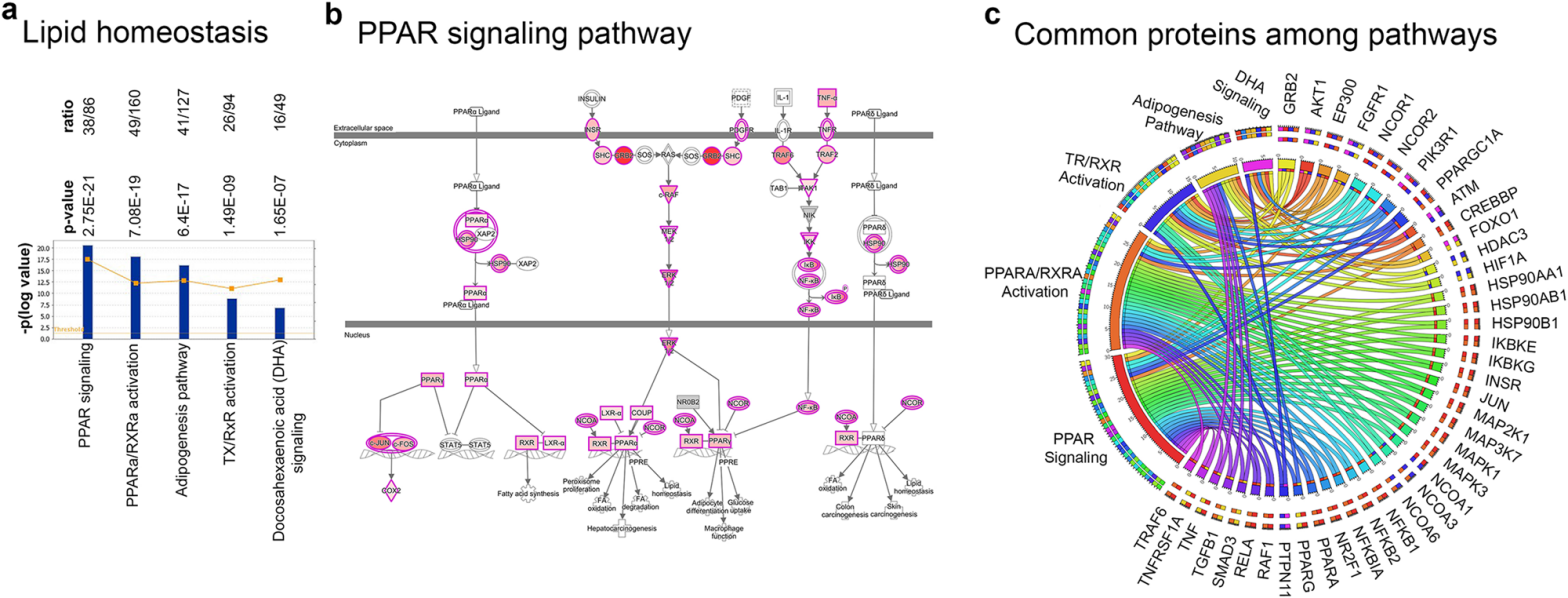
Involvement of ALS proteins in lipid homeostasis. (**a**) Bar graphs of canonical pathways involved in lipid homeostasis, as highlighted by ALS protein interactions (−p(log value)) and their overlap (ratio, yellow line). (**b**) Image of PPAR Signaling pathway, representing the extent of ALS protein involvement. ALS proteins with higher binding partners are marked with increasing color intensity. (**c**) Circular representation of ALS proteins that are commonly present among different canonical pathways.

Many of the ALS proteins were indeed present in canonical pathways that are involved in lipid homeostasis (Fig. 3a), such as PPAR signaling (38/86; p = 2.75E-21; Fig. 3b), adipogenesis pathway (41/127; p = 6.4E-17; Supplementary Fig. 2), DHA signaling (16/49; p = 1.65E-7; Supplementary Fig. 3), TX/RXR activation (26/94; p = 1.49E-9; Supplementary Fig. 4), and PPARa/RXRa activation (49/160; p = 7.08E-19; Supplementary Fig. 5). Maintaining a strict balance for the production, utilization and recycling of lipids appear to be an important task for ALS. For example, in PPAR signaling, which is one of the major canonical pathways for the lipid biosynthesis and function, the ALS proteins were present in almost all aspects of the signaling event, suggesting a significant coverage of this cellular event by the ALS protein and the importance of this for the health and function of the motor neuron circuitry (Fig. 3b). The color intensity of proteins are determined by the number of interactions they have with the protein products of the ALS related genes. The darker proteins have higher numbers of interactions. Some proteins involved in multiple different canonical pathways, and thus potentially were more central for the success of this biological event (Supplementary Table 5). The circular diagram displays ALS proteins that are part of two or more canonical pathways, such as GRB2, AKT1, EP300, FGFR1, NCOR1, NCOR2, PIK3R1, PPARGC1, STAT5B, suggesting that they may be particularly important for cellular events that are related to lipid homeostasis (Fig. 3c). PPARG and PPARGC1 were also present in multiple canonical pathways.

We next investigated other canonical pathways in which presence of ALS proteins cannot be explained by luck. We find that the ALS proteins were mainly involved in cellular events that maintain the integrity of the cyto-architecture, protein homeostasis, as well as cellular events that are involved in the preparedness for DNA repair and hypoxic insult. In addition, the growth factors appeared to play an important role for maintaining the health and stability of the motor neuron circuitry.

#### Protein homeostasis

**Figure 4:**
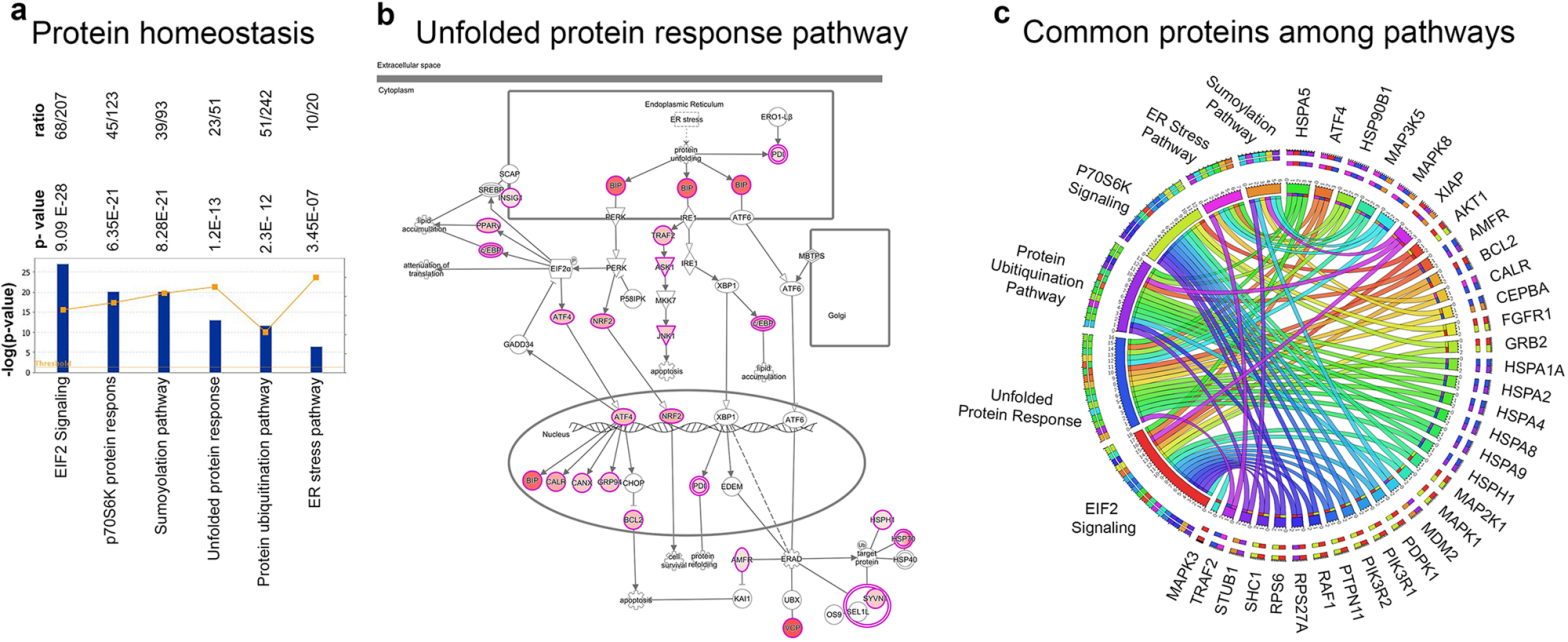
Involvement of ALS proteins in protein homeostasis. (**a**) Bar graphs of canonical pathways involved in protein homeostasis, as highlighted by ALS protein interactions (−p(log value)) and their overlap (ratio, yellow line). (**b**) Image of Unfolded protein response pathway, representing the extent of ALS protein involvement. ALS proteins with higher binding partners are marked with increasing color intensity. (**c**) Circular representation of ALS proteins that are commonly present among different canonical pathways.

The ALS proteins determined based on the human mutations and their binding partners were primarily involved in maintaining protein production/degradation balance. Canonical pathways, such as EIF2 Signaling (68/207; p = 9.09E-28; Supplementary Fig. 6), ER stress pathway (10/20; p = 3.45E-7; Supplementary Fig. 7), p70S6K protein response (45/123; p = 6.35E-21; Supplementary Fig. 8), protein ubiquitination pathway (81/242; p = 2.3E-13; Supplementary Fig. 9), sumoylation pathway (39/93; p = 8.28E-21; Supplementary Fig. 10), and unfolded protein response (23/51; p = 1.2E-13; Fig. 4b), were primarily highlighted. Here we show the unfolded protein response pathway, which reveals the extent of ALS protein involvement in all aspects of the pathway. Interestingly, some of the proteins were present in numerous canonical pathways and thus their absence may be more impactful (Supplementary Table 6). HSPA5 was involved in EIF2 signaling, unfolded protein response, protein ubiquitination and ER stress pathways (Fig. 4c), suggesting that its presence and activity is crucially important for protein homeostasis. Likewise, HSP90B1, HSPA1A, HSPA2, HSPA4, HSPA9, HSPH1, and many of the kinases, such as MAP3K5, MAPK8, MAP2K1 were also involved in more than 2 different canonical pathways.

#### Response to hypoxic insult

**Figure 5:**
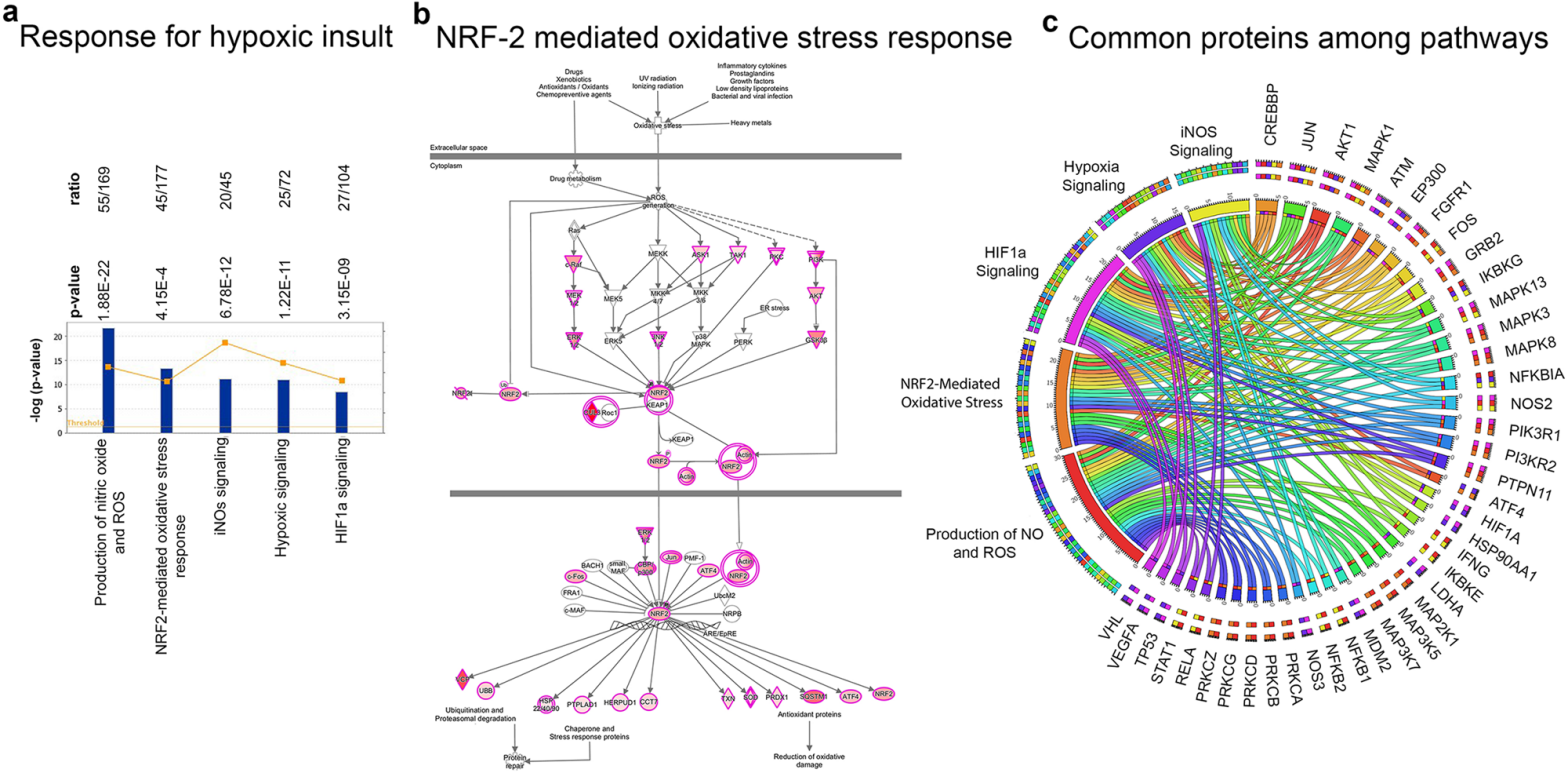
Involvement of ALS proteins in response to hypoxic insult. (**a**) Bar graphs of canonical pathways involved in response to hypoxic insult, as highlighted by ALS protein interactions (−p(log value)) and their overlap (ratio, yellow line). (**b**) Image of NRF-2 mediated oxidative stress response pathway, representing the extent of ALS protein involvement. ALS proteins with higher binding partners are marked with increasing color intensity. (**c**) Circular representation of ALS proteins that are commonly present among different canonical pathways.

Neurons have mechanisms to prevent hypoxic insult mediated toxicity. The ALS proteins appear to be part of canonical pathways that are particularly important for maintaining homeostasis, such as HIF1a signaling (27/104; p = 3.15E-9; Supplementary Fig. 11), hypoxic signaling (25/72; p = 1.22E-11; Supplementary Fig. 12), iNOS signaling (20/45; p = 6.78E-12; Supplementary Fig. 13), production of NO and ROS (55/169; p = 1.88E-22; Supplementary Fig. 14), and NRF2-mediated oxidative stress response (45/177; p = 4.15E-13, Fig. 5b). NRF-2 appears to be an important converging transcriptional regulator, which controls the expression of numerous genes, –that code for an ALS protein–, and that play and active role in protein repair and reduction of oxidative damage (Fig. 5b). Many of the proteins that are important for this cellular event also took part in more than 2 canonical pathways (Supplementary Table 7). Interestingly, CREBBP and JUN were present in all canonical pathways, and many of the proteins, especially kinases were shared among different canonical pathways, as depicted in the circular distribution (Fig. 5c).

#### DNA damage repair

**Figure 6:**
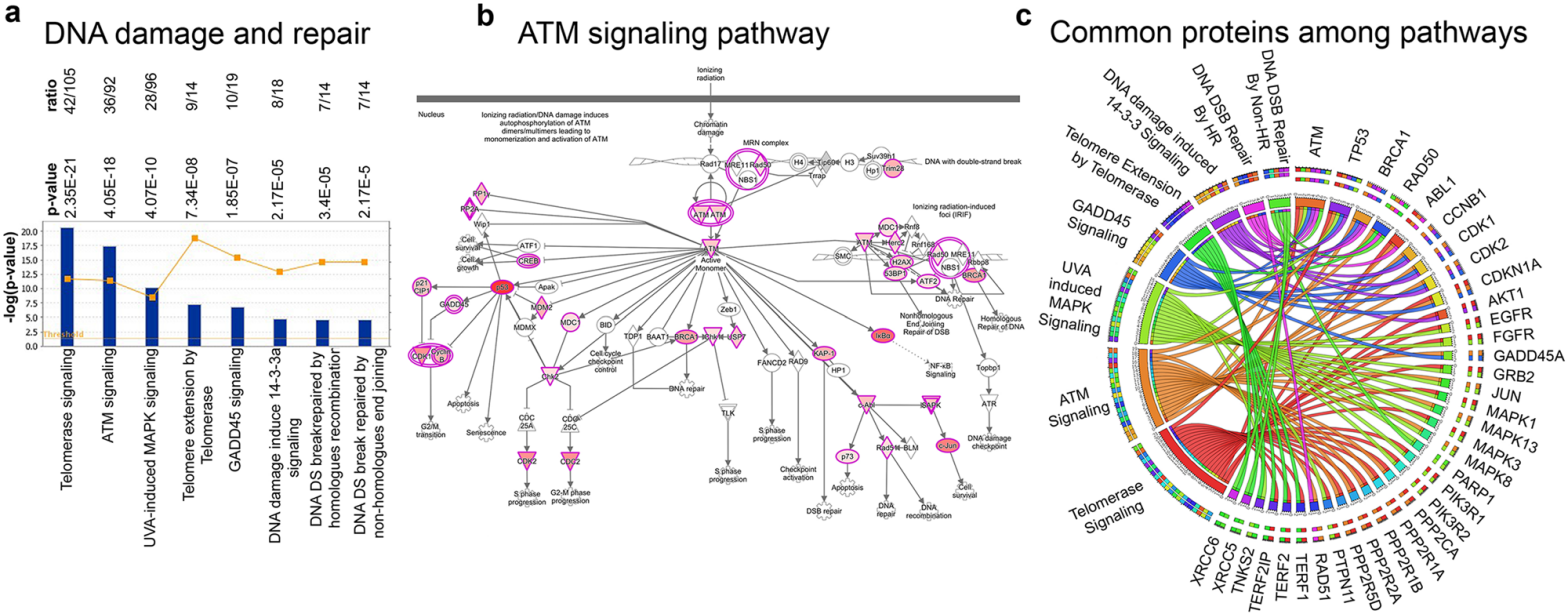
Involvement of ALS proteins in DNA damage and repair. (**a**) Bar graphs of canonical pathways involved in DNA damage and repair, as highlighted by ALS protein interactions (−p(log value)) and their overlap (ratio, yellow line). (**b**) Image of ATM signaling pathway, representing the extent of ALS protein involvement. ALS proteins with higher binding partners are marked with increasing color intensity. (**c**) Circular representation of ALS proteins that are commonly present among different canonical pathways.

Based on the canonical pathways that are highlighted as significantly covered by ALS proteins, maintaining the stability of DNA appear to be an important task. DNA double stranded break repair by homologous recombination (7/14; p = 3.4E-5; Supplementary Fig. 15), DNA damage induced 14-3-3 signaling (8/18; p = 2.17E-5; Supplementary Fig. 16), GADD45 signaling (10/19; p = 1.85E-7; Supplementary Fig. 17), DNA double stranded break repair by non-homologous end joining (7/14; p = 2.41E-5; Supplementary Fig. 18), telomere extension by telomerase (9/14; p = 7.34E-8; Supplementary Fig. 19), Telomerase signaling (42/105; p = 2.35E-21; Supplementary Fig. 20), UV-induced MAPK signaling (28/96; p = 4.07E-10; Supplementary Fig. 21), and ATM signaling (36/92; p = 4.05E-18; Fig. 6b), were highlighted for their high ratio and p-values, suggesting that presence of ALS proteins in these pathways cannot be explained by luck and that an important portion of ALS proteins are mainly involved in cellular events that ensure stability and integrity of DNA (Supplementary Table 8). For example, the canonical pathway figure for the ATM signaling reveal that most of the proteins involved in key components of the cellular event are indeed an ALS protein (Fig. 6b), and that having active ATM monomer is critically important for the proper function of downstream events that ensure DNA repair. The circular distribution of ALS proteins and canonical pathways highlight the key proteins that are part of more than 3 of the canonical pathways (Fig. 6c), again suggesting the importance of ATM, which is present in 7 different canonical pathways, and other proteins such as CCNB1 and CDKN1A, which play important roles in 3 different canonical pathways.

#### Maintaining cyto-architectural integrity

**Figure 7:**
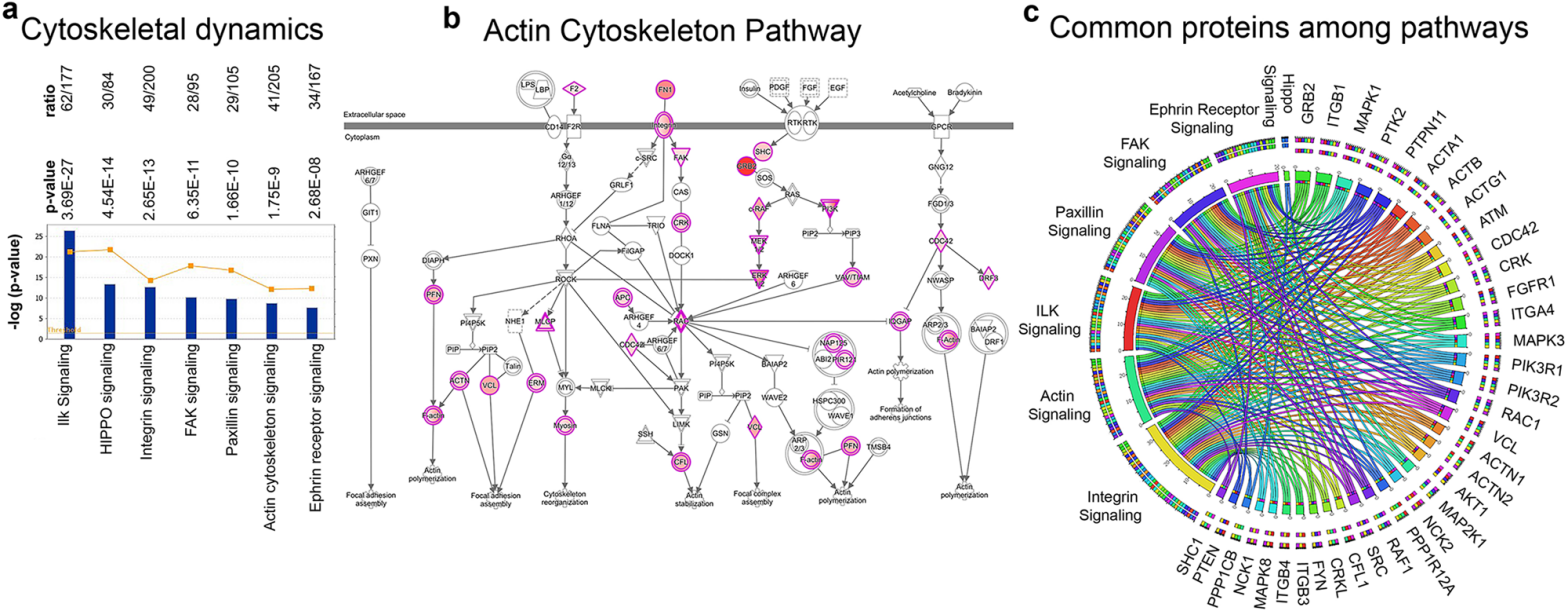
Involvement of ALS proteins in cytoskeleton dynamics. (**a**) Bar graphs of canonical pathways involved in cytoskeleton dynamics, as highlighted by ALS protein interactions (−p(log value)) and their overlap (ratio, yellow line). (**b**) Image of Actin cytoskeleton pathway, representing the extent of ALS protein involvement. ALS proteins with higher binding partners are marked with increasing color intensity. (**c**) Circular representation of ALS proteins that are commonly present among different canonical pathways.

Maintaining cyto-architectural integrity requires activation of numerous canonical pathways. Here we find that ALS proteins are significantly present in the ILK signaling (62/117; p = 3.69E-27; Supplementary Fig. 22), integrin signaling (49/200; p = 2.65E-13; Supplementary Fig. 23), HIPPO signaling (30/84; p = 4.54E-14; Supplementary Fig. 24), FAK signaling (28/95; p = 6.35E-11; Supplementary Fig. 25), Paxillin signaling (29/105; p = 1.66E-10; Supplementary Fig. 26), Ephrin receptor signaling (34/167; p = 2.68E-8; Supplementary Fig. 27), and actin cytoskeleton signaling (41/205; p = 1.75E-9; Fig. 7b). Here we highlighted the actin cytoskeleton pathway (Fig. 7b), but other canonical pathways also equally demonstrate how integrated ALS proteins are within these canonical pathways and how they are involved in almost all aspects. It is important to note that some ALS proteins were present in more than one canonical pathway, suggesting that they are involved in multiple cellular events (Supplementary Table 9). For example, GRB2, ITGB1, PTK2, PTPN11 were present in 6 different canonical pathways (Fig. 7c). These results suggest that among all ALS proteins, some may play multiple roles and thus are involved in numerous cellular activities.

#### Response to growth factors

**Figure 8:**
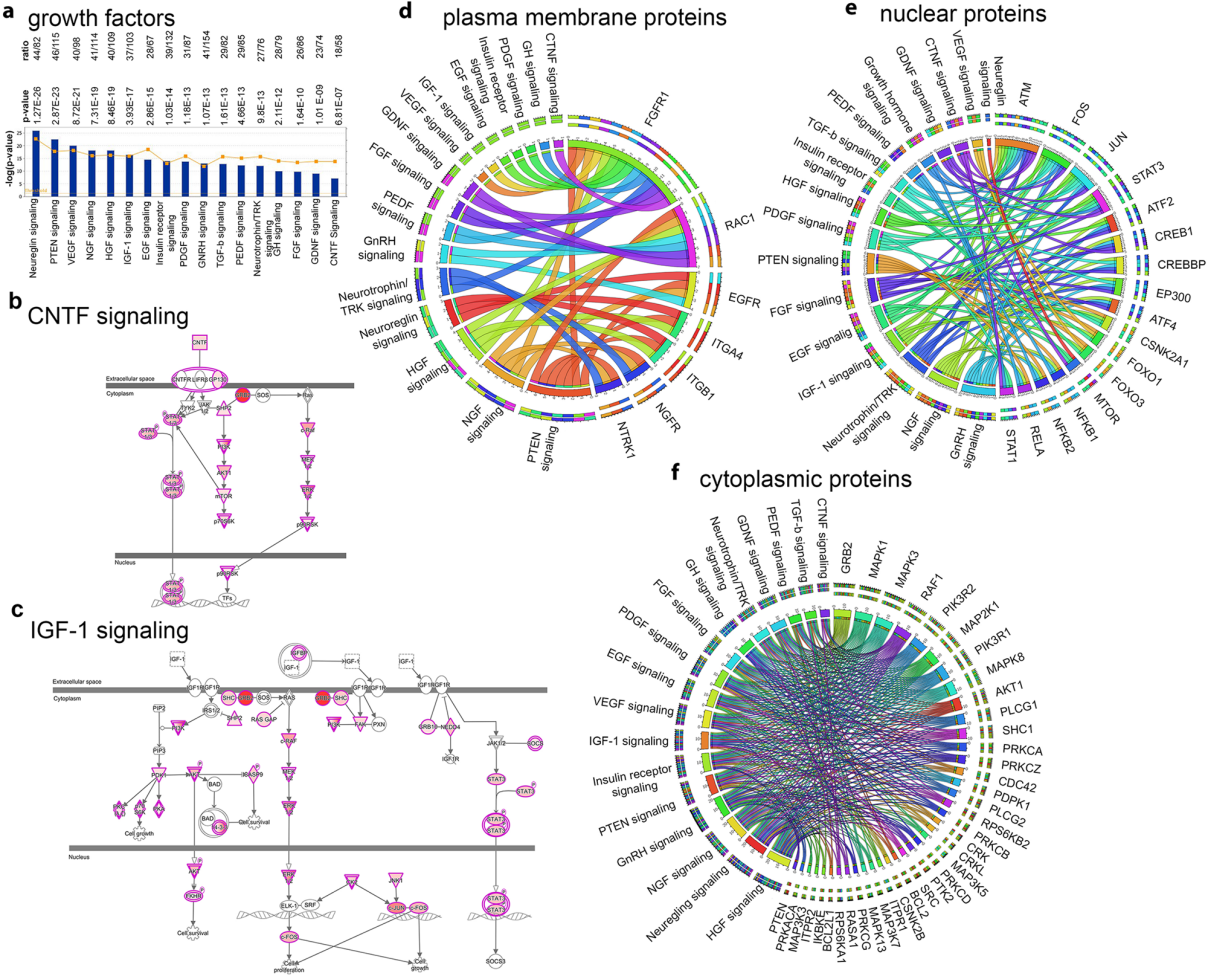
Involvement of ALS proteins in growth factor mediated signaling pathways. (**a**) Bar graphs of growth factor mediated signaling pathways, as highlighted by ALS protein interactions (−p(log value)) and their overlap (ratio, yellow line). (**b,c**) Image of CNTF signaling pathway(**a**) and IGF-1 signaling representing the extent of ALS protein involvement. ALS proteins with higher binding partners are marked with increasing color intensity. (**d,e,f**) Circular representation of ALS proteins that are commonly present among different canonical pathways per cellular location: plasma membrane (**d**), nuclear (**e**), cytoplasm (**f**).

It is no surprise that growth factors are important for neuronal survival, growth, maturation and function. However, not all growth factors support all neuron populations to the same extent and degree. There is a very precise selective response to growth factors. Here, we also find that ALS proteins are particularly involved in a select set of growth factor response signaling. For example, EGF (28/67; p = 2.86E-15; Supplementary Fig. 28), FGF (26/86; p = 1.64E-10; Supplementary Fig. 29), GDNF (23/74; p = 1.01E-9; Supplementary Fig. 30) Growth hormone (28/79; p = 2.11E-12; Supplementary Fig. 31), GNRH (41/154; p = 1.07E-13; Supplementary Fig. 32), HGF (40/109; p = 8.46E-19; Supplementary Fig. 33), insulin (39/132; p = 1.03E-14; Supplementary Fig. 34), Neurotrophin/TRK (27/76; p = 9.8E-13, Supplementary Fig. 35), Neuroregulin (44/82; p = 1.27E-26; Supplementary Fig. 36), NGF (41/114; p = 7.31E-19; Supplementary Fig. 37), PDGF (31/87; p = 1.8E-13; Supplementary Fig. 38), PTEN (46/98; p = 2.87E-23; Supplementary Fig. 39), TGF-β (29/82; 1.61E-13; Supplementary Fig. 40), VEGF (40/98; 7.31E-19; Supplementary Fig. 41), IGF1 (37/103; p = 3.93E-17; Fig. 8c), PDGF (31/87; p = 1.18E-13; Supplementary Fig. 42), CNTF (18/58; p = 6.8E-7; Fig. 8b) and IGF-1 signaling (37/103; p = 3.93E-17, Fig. 8c) were primarily highlighted by the high level presence of ALS proteins in these growth factor mediated signaling events. Here we highlight only CNTF (Fig. 8b) and IGF-1 signaling (Fig. 8c), both of which have been previously reported to improve the health of motor neurons and increase survival of ALS mouse models^16–19^. But, we encourage you to look at other canonical pathways which have better coverage of ALS proteins (Supplementary Figs 28–38, Supplementary Table 10). Since many proteins were present in growth factor signaling pathways and it was not possible to demonstrate them within one circular diagram, we divided proteins bases on their location. Among proteins that are present in the plasma membrane, FGFR1 was present in 14 different canonical pathways (Fig. 8d), ATM, FOS and Jun, were nuclear proteins highlighted to be present among more than 10 canonical pathways (Fig. 8e). MAPK1, MAPK3, MAPK2K1, MAPK8 were the Map kinases with highest representation among different canonical pathways and were important contributors to the successful execution of almost all growth factor mediated signaling events.

Upon identification of key cellular events ALS proteins that part in, we investigated presence of proteins that are present not only in different canonical pathways, but have an overarching presence in different cellular events. YWHAZ, a 14-3-3 protein was present with active roles in three different canonical pathways; the growth factor signaling (i.e. IGF-1 signaling), protein homeostasis (i.e. P70S6K protein response), and DNA repair mechanisms (i.e. Hippo signaling). It’s key presence in three independent but important cellular events suggested that maintaining its levels would be important in ALS.

**Figure 9:**
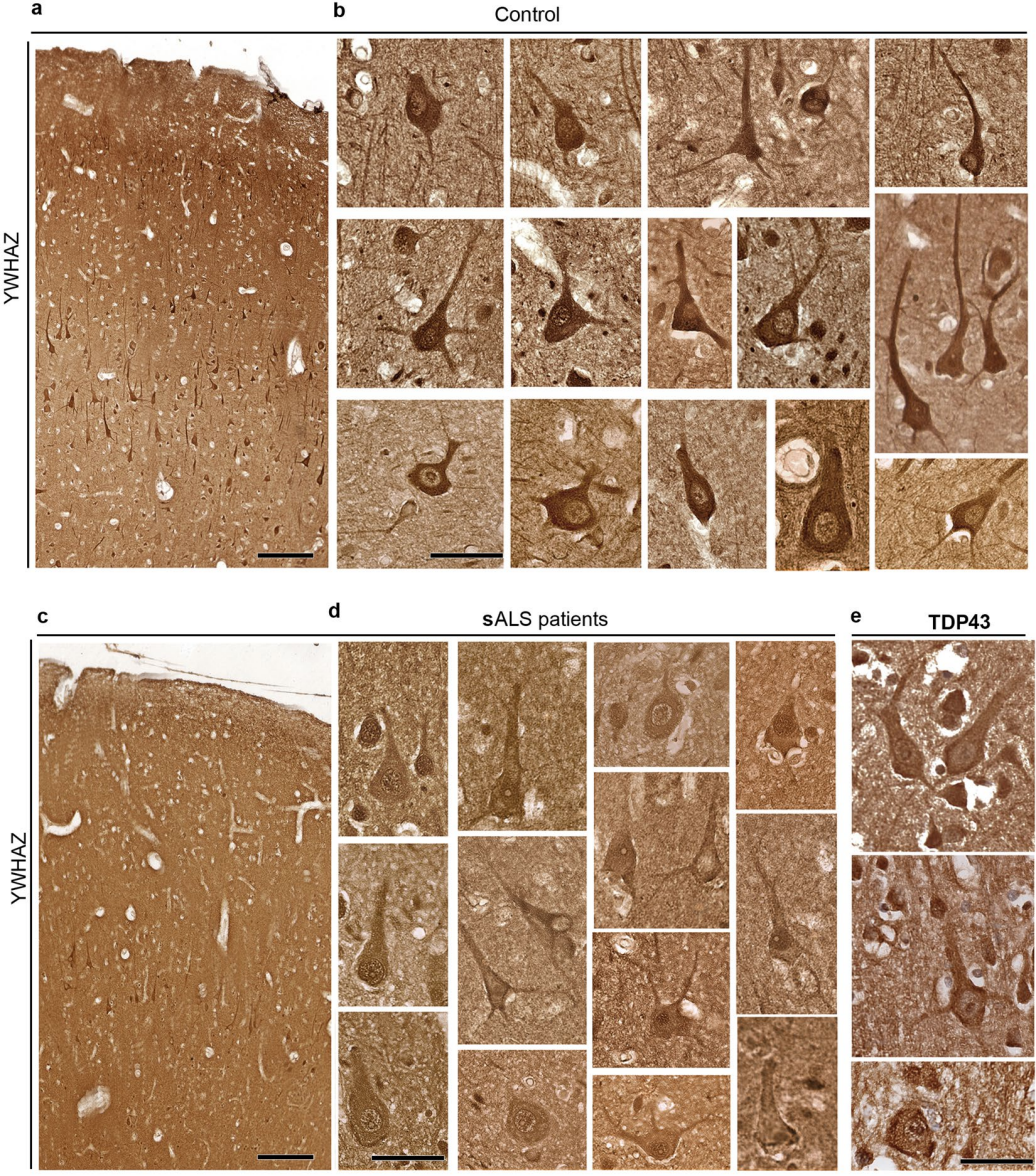
YWHAZ expression is reduced in the Betz cells of ALS patients. (**a**) Representative image of the motor cortex in control cases (*n* = 12). Betz cells are located in layer V and can be visualized based on their high-level and selective YWHAZ expression. (**b**) Representative images of control Betz cells, expressing high levels of YWHAZ in their cytoplasm, and apical dendrites. (**c**) Low magnification view of the motor cortex in sporadic ALS cases (n = 8), displaying reduced levels of YWHAZ expression in Betz cells. (**d**) Representative images of sporadic ALS patient Betz cells, and (**e**) Betz cells of ALS patients with TDP43 pathology (n = 9), expressing reduced levels of YWHAZ, where the protein is mainly localized along the cell membrane. Scale bar = 150 μm (a,c); scale bar = 50 μm (b,d).

Investigation of YWHAZ expression in the motor cortex of controls cases revealed high levels of YWHAZ expression primarily in the Betz cells, and the level of expression was so high and primarily restricted to Betz cells that it could be visualized with 4X objective. Other cells and neurons either did not express YWHAZ, or had very low level of expression in comparison to Betz cells (Fig. 9a). YWHAZ protein was present throughout the neuron, also detected in the apical and basal dendrites (Fig. 9b). In striking contrast, first there were fewer Betz cells detected in the motor cortex of sALS cases, as expected, but more importantly, diseased Betz cells displayed either reduced levels of YWHAZ expression, or the protein location shifted towards the cell membrane (Fig. 9c). The YWHAZ expression was reduced both in the Betz cells of sALS patients and ALS patients with TDP43 pathology. There were less Betz cells with high levels of YWHAZ in patients (average percentage of Betz cells with high levels of YWHAZ: control: 91 ± 3; sALS: 16 ± 9; TDP43: 14 ± 4). Even though results from sALS and TDP43 were comparable (p = 0.8338), control versus sALS (p = 0.001) and control versus TDP43 (p = 0.001) differed significantly.

### Protein Network Analysis

We next investigated whether ALS proteins closely interacted with each other, forming protein interactome domains that are particularly important for key cellular events. One of the key protein interactomes included ZFYVE27 protein with the highest number of interactions (n = 17) at the heart of the network. Upon removal of ZFYVE27, the interactome disintegrated, suggesting a crucial role for ZFYVE27 for keeping the integrity of this interaction. This interactome included 27 proteins, and was suggested to be particularly important for stress response of cells (*n* = 7; p = 2.28E-8), and more specifically ER stress response (*n* = 12; p = 5.85E-7) and oxidative stress response (*n* = 4; p = 9.76E-6). Transport of molecules (*n* = 19; p = 6.61E-11), such as metal ions (*n* = 8; p = 1.62E-6), and more specifically transport of K + (*n* = 3; p = 7.91E-4) were highlighted. In addition maintaining transmembrane potential of mitochondria (*n* = 7; p = 6.24E-7), and maintain Ca^+2^ balance (*n* = 6; p = 4.51E-4) were also important functions of this interactome domain.

**Figure 10:**
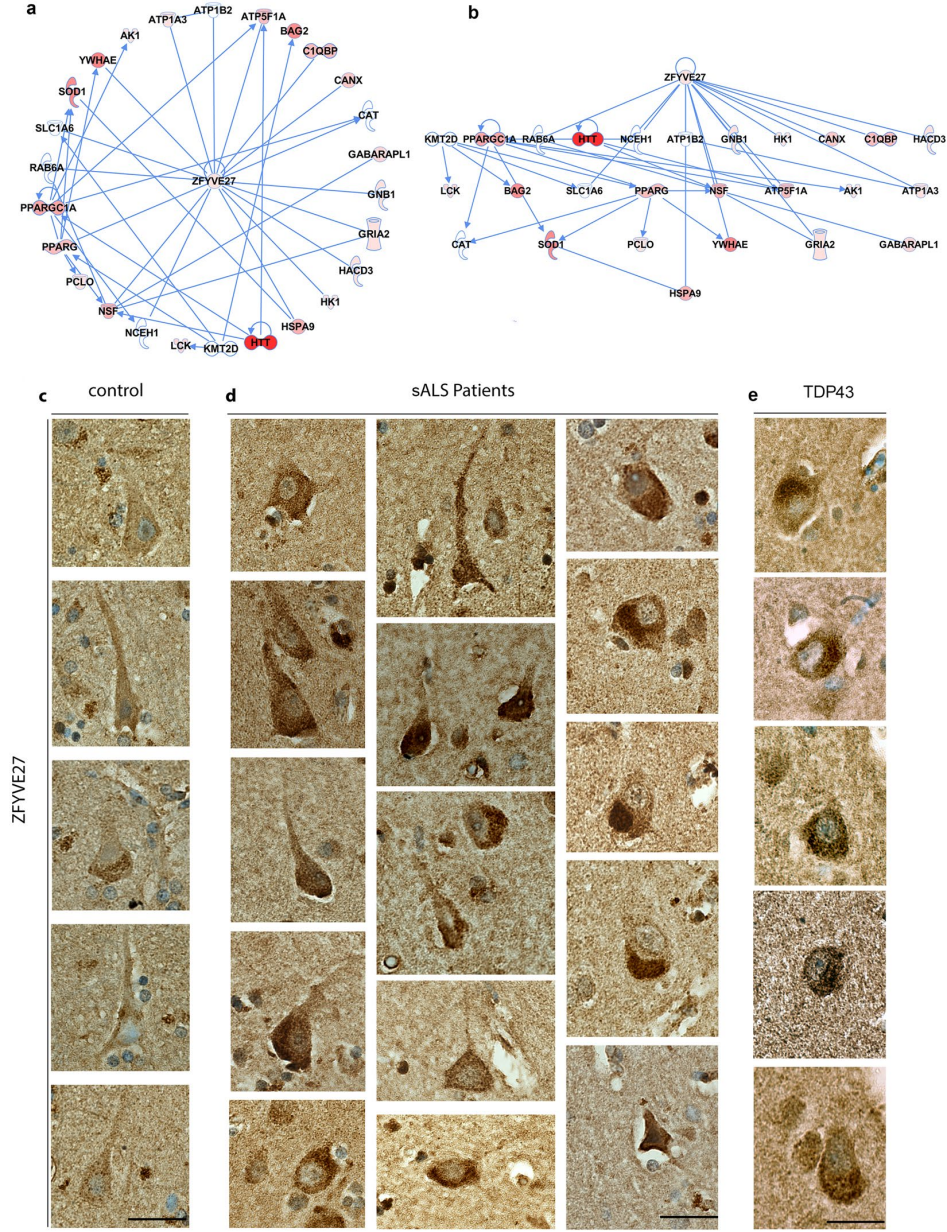
Protein interactome analysis suggests the importance of ZFYVE27. (**a**) ZFYVE27 is at the center of a prominent protein interactome domain. (**b**) ZFYVE27 is the upstream regulator of proteins present in the interactome domain. (**c**) Representative images of Betz cells in control cases (n = 12). ZFYVE27 is present in the cytoplasm at low levels. (**d**) Representative images of Betz cells in sporadic ALS patients (n = 8). (**e**) Representative images of Betz cells in ALS patients with TDP43 pathology (n = 9). ZFYVE27 expression is increased and protein is accumulated in discrete regions within the cytoplasm. Scale bar = 50 μm in c–e.

Investigation of the same interactome domain via hierarchical view to determine the top regulators and the downstream effectors, suggested that ZFYVE27 was the effective modulator, further suggesting its importance. We next investigated whether ZFYVE27 expression was altered in the Betz cells of sALS patients (Fig. 10c). ZFYVE27 protein was detected in all Betz cells of control cases (n = 12). There was robust but low levels of expression within the soma. In contrast, ZFYVE27 expression was significantly increased in the Betz cells of all sALS patients (*n* = 8) and patients with TDP43 (*n* = 9) pathology. (Average percentage of Betz cells expressing high levels of ZFYVE27: control: 24 ± 5; sALS: 74 ± 4; TDP43: 83 ± 7). Both sALS and TDP43 cases were significantly different from the control cases (p < 0.0001), but they were comparable among each other (p = 0.2431).

In summary, our analysis which originated from the mutations detected in ALS patients, circled back to human patients and investigated whether the proteins that are suggested to play key roles within the ALS protein domain were expressed in the Betz cells, and whether their expression profiles were altered with respect to disease in sALS patients and ALS patients with TDP43 pathology. Our findings reveal that proteins, their interactions, and their dynamics offer novel insights into cellular events that are particularly important in ALS, and suggest the importance of understanding protein dynamics to reveal cellular basis of neuronal vulnerability in ALS. By shifting our attention from genes to proteins and to their interactions, we may begin to understand the cellular events that are perturbed due to different mutations, and the underlying consequences that lead to neuronal vulnerability and progressive degeneration.

## Discussion

Patients with seemingly unrelated gene mutations are diagnosed with ALS, but they display similar disease pathology. To date 147 different genes are either linked to ALS or are closely associated. Therefore, understanding why so many different and seemingly unrelated genes lead to the same neuronal pathology has been an important puzzle.

If a mutation in a single gene leads to selective motor neuron degeneration, the protein product of that particular gene and its binding partners must be critically important for a cellular event that is especially required for the health and function of the motor neuron that degenerates. Therefore, we first decided to generate a list of “ALS proteins”, which include the protein products of the mutated genes in ALS and their direct binding partners, determined by experimental results that are published. Investigation of these proteins began to reveal which canonical pathways ALS proteins are particularly involved in, how they interact, which cellular events they upregulate and how they ensure that the system retains homeostasis.

IPA and other large-data management tool boxes offer a great tool to analyze large data sets, but they are approached with great skepticism for valid reasons. The analysis is unbiased and includes both experimental findings and curated suggestions. It also includes data from a vast variety of resources (e.g. pure biochemistry, cell lines, *in vivo* systems) and fields (e.g. cancer field, neuroscience, medicine). Therefore, many feel troubled distinguishing real suggestion from a false negative or false positive affirmation. However, recent studies began to utilize IPA and other network analysis to reveal protein dynamics and interactions with respect to disease^7^. To overcome many of the challenges of using large data management toolboxes, we first utilized a very stringent inclusion criteria: only the experimentally observed and previously published direct interactions using neuronal systems are included. Many different protein-protein interaction studies, such as Co-IP followed by proteomics, yeast-two-hybrid and protein mobility shift assays experimentally determined the binding partners of these proteins in mammalian systems and CNS. Taking advantage of 2456 previously peer reviewed and published studies that report direct protein-protein interaction with the protein products of the ALS linked and associated genes, and using a very stringent inclusion criteria for analysis allowed us to generate a refined list with proven experimental results, yielding suggestions with higher confidence intervals. (Supplementary Table 1, 2). Since the starting point of our analysis was the mutations detected in ALS patients, circling back to human and investigating the validity of the data driven hypothesis was very powerful.

The distribution of ALS proteins among known canonical pathways, suggested the importance of maintaining homeostasis for lipids, proteins, preparing the cell for a potential DNA damage, hypoxic insult, and ensuring cytoarchitectural integrity and stability. Perturbations that alter the balance for these biological events may become detrimental over time. Recent evidence suggest that lipid metabolism is altered in ALS and there is significant remodeling of lipidome in the cortex of ALS mice and CSF of ALS patients^20,21^. We find that PPARG and PPARGC1A, two key proteins that are important in lipid biosynthesis and were reported to activate mechanisms responsible for scavenging lipid peroxidation by-products^22^, were indeed upregulated especially in the Betz cells of sALS patients and patients with TDP43 pathology. This suggests potential perturbations in cellular events related to lipid homeostasis and that either motor neurons are trying to compensate by increasing the protein expression, or increased presence of these proteins contribute to disease pathology. It is important to note that not every increase in protein expression translates to causality. It is possible that diseased neurons increase the presence of a distinct set of proteins to maintain perturbed balance, and it indeed may be a protective for the neuron.

Maintaining homeostasis for the lipids and fatty acids require controlled activation of numerous canonical pathways. PPAR (peroxisome proliferator-activated receptor) is a family of proteins, consisting of PPARα, PPARβ, PPARγ, acting as a ligand-activated transcriptional regulators that regulate intracellular lipid levels^23,24^. PPAR signaling is one of the most significant canonical pathways involved in lipid metabolism, fatty acid oxidation and uptake^23,24^, and has been investigated within the context of ALS^25^. In addition, upon binding to the retinoid X receptor (RXR), genes that affect fatty acid metabolism are upregulated^26^. Interestingly, in endothelial cells and macrophages they exert an anti-inflammatory and anti-oxidative effects, linking lipid and fatty acid homeostasis to inflammation^27–29^. Similar to PPAR/RXR activation, the Thyroid hormone receptor and RXR activation leads to expression of genes that modulate lipid metabolism. Docosahexaenoic acid (DHA) is a member of the Omega-3 family of essential fatty acids, and it accumulates in phospholipid bilayer of neuronal cell membranes^30^. DHA signaling is important for initiation and modulation of survival signaling, and in the presence of fatty acid deficiency DHA signaling is perturbed, and promotion of neuronal survival is affected^31,32^. The ratio and p-values suggest that key canonical pathways that are responsible for maintaining lipid and fatty acid homeostasis are highly active, and mutations that perturb this balance may contribute to neuronal vulnerability in ALS. This idea has already been investigated and recent evidence suggests how perturbations in lipid homeostasis is observed in ALS patients and how it may contribute to disease pathology^20,21^.

Since neurons are very active, there is a constant production and turn-over of proteins, which need to be closely monitored. Protein synthesis requires ribosomal proteins, modification enzymes and ribosome associated translation factors to cooperate. eIFs (eukaryotic translation initiation factors) escort the met-tRNA onto the ribosome to initiate translation^33^. The protein ubiquitination pathway is one of the key canonical pathways that modulate addition of ubiquitin to promote protein degradation. However, addition of ubiquitin have numerous other functions, such as labeling of proteins for proper intracellular transport, activation, and secretion^34,35^. In addition to ubiquitination, sumoylation is also an important posttranslational modification capable of modifying stability, protein-protein interaction and subcellular localization of proteins. Sumoylation canonical pathway includes proteins that add and remove the small ubiquitin-like modifying SUMO, to the SUMO-affinity sites of proteins^36^. For example, on the nuclear membrane RanGAP and conjugated Ubc9 sumoylates proteins as they are imported inside nucleus, where RanBP serves as the E3 ligase of the reaction^37^, very important for controlling the location and the function of some key proteins.

ER is the site of protein synthesis and folding and protein accumulations are closely linked to initiation of ER-stress. A battery of quality controls ensure that properly folded proteins exit and others are either corrected with the help of chaperone proteins or are retained in the ER^38^. Accumulation of such proteins in the ER is one of the leading causes of neurodegeneration. ER stress activates the unfolded protein response (UPR) signaling network to restore ER homeostasis. The three ER proteins, inositol requiring 1 (IRE1), PKR-like ER kinase (PERK), and activating transcription factor 6 (ATAF6) are the key regulators of the UPR signaling^39,40^. They are kept in the inactive state by BiP, but during ER stress they become active and initiate a cascade of events that leads to the phosphorylation of eIF2 and a general halt of translation, activation of UPR genes, upregulation in the expression of chaperone proteins, ER-associated protein degradation (ERAD), and activation of autophagy. Based on the canonical pathways highlighted in this study, ALS proteins are indeed actively involved to ensure that the protein folding and turn-over is properly controlled.

Maintaining the cytoarchitectural integrity of motor neurons, which have large soma and send out axons to very long distances, is not an easy task. Therefore, there is an ongoing activity to maintain homeostasis of cytoskeleton, with the active involvement of numerous canonical pathways that are particularly important for ensuring the availability of key components of building blocks and their continuous supply and proper assembly. Actin cytoskeleton is important for cell motility, mobility of organelles inside the cell, phagocytosis and endocytosis^41^. Numerous signaling pathways control the rearrangement of actin cytoskeleton, such as Rho family of GTPases and kinases. For example, RhoA activates Rho kinase (ROCK), which then increases myosin light chain and LIM-kinase phosphorylation and inhibits myosin light chain phosphatase, resulting in the facilitation of actinomyosin assembly, and inhibition of cofilin, an actin depolymerizing protein^42,43^. This stabilizes the actin and helps cells and neurons maintain a strong cyto-architecture. Neurons, especially large, polarized neurons, such as the motor neurons highly require controls over actin dynamics so that they can maintain their large, yet fragile posture.

Integrins are another important player of cellular architecture, as they are the cell surface glycoproteins involved in cell-cell and cell- extracellular matrix (ECM) interactions^44^. They facilitate the communication between the ECM and the cytoplasm, and help translate extracellular effectors into structural changes inside the cell. The dynamic nature of attachment and detachment of cell membrane to and from ECM and the controls over these cellular events are important for maintaining cell shape, stability and at times proper function. Binding of cells to the ECM results in clustering of integrins at the site of attachment and recruitment of structural molecules, such as vinculin, paxillin and talin, as well as signaling proteins/enzymes, such as kinases and GTPases^45^. The paxillin, FAK, ILK, HIPPO, and ephrin receptor signaling all require proper assembly of cell-ECM interaction domains and recruitment of proper proteins to the site. When such connections cannot be formed, or proteins of interest cannot be recruited to the site of interest, the signaling cascade of events will not be properly initiated. This in turn will have severe consequences on maintaining cellular cyto-architecture, proper respond to extracellular stimuli, and activation of key signaling cascade of events that are responsible for cellular response to stress and activation of gene expression. Thus, it is not surprising that many of the ALS proteins are actively involved in canonical pathways that are responsible for maintaining cyto-architectural homeostasis.

Neurons need to preemptively activate canonical pathways that protects them from damaging agents, such as free radicals, UV radiation, and nitric oxide. Based on the canonical pathways that are detected in our study, it appears that in ALS maintaining the integrity of DNA is crucial. Many of the canonical pathways that are responsible for DNA-repair, such as the repair via non-homologous end joining and homologous recombination, as well as signaling cascade of events, such as GADD45 (growth arrest and DNA-damage inducible 45), ATM (Ataxia telangiectasia) and UV-induced MAPK signaling pathways were detected to be important cellular events significantly covered by the ALS proteins. DNA is exposed to many damaging agents, such as mutagenic chemicals, UV radiation, as well as mechanical stress on chromosomes, heavy metal ion redox cycling. In addition, reactive oxygen species and free radicals damage DNA. One of the major cellular response to DNA damage is mediated via p53 tumor suppressor protein, which upon activation induce expression of genes that help improve the integrity of damaged DNA. *GADD45* is one of such genes, and in response to environmental stress and DNA damage, GADD45 promotes DNA repair and initiates activation of JNK/p38 MAPK signaling and survival pathways^46^. In addition, ATM is one of the key responders to DNA damage^47^. Downstream of ATM lies a plethora of factors, such as Rad50, Chk2, Rad51, GADD45, c-Abl and NF-Kβ, all of which play significant roles in DNA damage repair and promotion of cell survival^48^. For example, ATM phosphorylates c-Abl and this leads to the activation of SAPK, and Rad51. Phosphorylation of Rad51^49^, further enhancing DNA repair pathways, and SAPK further activates c-Jun and cell survival^50^. There are numerous cellular events that combat potential DNA defects, and these require presence and activity of numerous proteins that work in synchrony.

Double stranded breaks are one of the most dangerous for cell survival as they may lead to disintegration of DNA, deregulation of gene expression and chromosomal instability. The non-homologous end joining and homologous recombination are the most two prominent canonical pathways that repair DNA damage and they both were detected in our studies. Interestingly, 14-3-3 protein mediated signaling events were present with high significance, and these 14-3-3- proteins are a large family of highly conserved, small, acidic polypeptides involved in numerous signal transduction events^51^. One of them is the DNA-damage induced 14-3-3 signaling. When DNA damage is detected, 14-3-3 proteins ensure that the cell stays at Go phase and that the damage is repaired. Problems with 14-3-3 proteins and their signaling promote immature entry to cell-cycle, losing control over DNA repair checkpoints, and numerous problems with DNA stability. Therefore, it is reasonable to think that in ALS, emphasis is given to canonical pathways that ensure integrity and stability of DNA.

Motor neurons, like many other neurons in our body, require growth factors for survival and improved health. Based on the canonical pathways identified in our study, some of the growth factors, hormones, and their signaling cascade of events are particularly important in ALS. For example, among all growth factors the VEGF, NGF, IGF-1, HGF, EGF, PDGF, FGF, and CNTF were particularly prominent. These growth factors are not important only for motor neurons, but are also important for the survival of other neurons and non-neuronal cells. For example, VEGF (vascular endothelial growth factor), a family of growth factors that consist of PLGF, VEGFA, VEGFB, VEGFC and VEGFD, plays an important role in restoring oxygen supply to tissues by promoting generation of new blood vessels^52^. It acts by binding to receptors such as VEGFR1, VEGFR2 (KDR/Flk1), VEGR3(FLT4) and initiates signal transduction that promotes a plethora of events such as control of vasopermeability and blood flow, stimulation of Ca^+2^ release from internal stores, and promotion of cellular survival^52^. VEGF activity is partly controlled by the expression of appropriate receptors on cell surface, and the presence of auxiliary proteins that facilitate signaling cascade of events. Interestingly, the hypoxia-inducible factor (HIF-1) and HuR promote VEGF expression under hypoxic conditions, increasing VEGF levels mainly in the vascular endothelial cells, generating a feedback loop^53^.

Hepatocyte growth factor (HGF), a ligand for c-Met, is a cytokine with numerous functions. For example, HGF signaling mediated via activation of GAB1-Akt-PAK1 leads to promotion of DNA repair and blocking apoptosis^54^. In addition, activation of c-Met receptor leads to activation of PLC-γ, generation of IP3 and DAG from PIP2, and thus mobilization of intracellular Ca^+2^. These are very important cellular events for improved survival and HGF signaling is crucial for maintaining homeostasis. Likewise, FGF (fibroblast growth factor) a heparin binding protein and is closely related to HGF, initiates a similar set of signaling cascade of events that promote initiation of Ras/Raf/MEK/ERK, Rac1/MEKK/p38MAPK or the PI3K/AKT pathways, which are linked to cell growth and survival^55^. In addition, by activating PLCγ and protein kinase C, FGF plays a role in maintaining Ca^+2^ homeostasis inside the cell^55^.

IGF-1 (insulin-like growth factor 1) is one of the major factors reported to support the survival of both upper and lower motor neurons and its mode of action is mostly controlled by the six different binding proteins, (i.e IGF1BP1-6). IGF-1 binds to IGF-1R, which results in the downstream signaling cascade of events that activate Ras/Raf/MEK and ERK pathways. Translocation of ERK to the nucleus activates ELK-1, c-Jun, c-Fos, and thus induction of genes that promote survival and growth^56^. In addition, IGF-1 induce phosphorylation of JAK-1, JAK-2 and STAT-3, promoting the JAK/STAT pathway^57^. Similar to IGF-1, the growth hormone (GH) is an anabolic hormone with broad regulatory actions on protein, lipid and carbohydrate metabolisms, and it signals via the GH receptor, activating similar signaling cascade of events that promote survival. Therefore, IGF-1 and GH signaling are important for many cell types and overall survival.

CNTF (ciliary neurotrophic factor) is produced mainly by glial cells and promote survival in many different cells and neurons. It is a member of the GP130 cytokine family and is related to cytokines such as IL-6, IL-11, leptin, and cardiotrophin-1. CNTF binds to CNTFRα, and this activates Ras/Raf/MEK/ERK/p90RSK line of signaling cascade of events^58^. CNTF acts on a broad range of neurons and cells and it is one of the most potent survival molecule. Based on the distribution of ALS proteins, the CNTF signaling appear to be an important cellular event in ALS.

Our studies identified YWAHZ, 14-3-3 protein zeta, to be involved in at least 3 different important cellular events, and investigation of its expression in Betz cells revealed dramatic reduction of its expression in all sALS and TDP43 cases studied. 14-3-3 proteins are ubiquitously expressed molecular chaperons that regulate a plethora of cellular events, and are reported to act as adaptors in numerous signal transduction pathways. Most related to neurodegeneration, 14-3-3 proteins are important for the localization and function of ion channels^59^, promote UPS^60^, and facilitate protein trafficking from ER^61^. There are seven different 14-3-3 isoforms, encoded by seven different genes, and they act either by forming homodimers or heterodimers with each other^62^. YWHAZ is mainly expressed in the nervous system and is linked to ER function. When YWHAZ levels are reduced it is reported to increase ER stress, and vulnerability to excitatoxicity. Depletion of YWHAZ exacerbates kainic acid mediated excitatoxicty^63,64^, and YWHAZ expression was decreased during epilepsy development in rat models^63^. Likewise, overexpression of YWHAZ improves unfolded protein response pathway, and protects hippocampal neurons from tunicamycin-induced ER stress and neuronal degeneration^63^, suggesting that maintaining high levels of YWHAZ is crucially important for neuronal survival in different neurodegenerative diseases^65^. Here we find that YWHAZ levels are significantly reduced in Betz cells of sALS patients and ALS patients with TDP43 pathology. We included a total of 8 sALS and 9 cases of ALS patients with TDP43+ aggregations, representing a broad spectrum of patients based both on age and sex, and disease pathology. It was remarkable that all patient cases investigated displayed comparable findings with reduced YWHAZ expression in their Betz cells, suggesting this could indeed be one of the common neuronal defects observed in vulnerable and diseased upper motor neurons. Based on previous reports and our findings, this reduction may contribute to neuronal vulnerability, and thus more detailed assessment of its role for motor neuron health and function is needed.

The protein interactome studies suggested a unique importance for ZFYVE27 (a.k.a Protrudin), as it was at the heart of an interactome domain, not only holding the interaction together, but also acting as a upstream effector. This interactome was highlighted especially for events that are important for maintaining homeostasis upon ER and oxidative stress, and also ensuring proper transport of molecules and ions, especially K^+^ and Ca^+2^. Interestingly, ZFYVE27 gene has recently been associated with hereditary spastic paraplegia (HSP), as mutations in ZFYVE27 were detected in HSP patients^66^. This is no surprise because ZFYVE27 is a very important protein with multiple important functions for the motor neuron. For example, ZFYVE27 has both protein (i.e. RBD11) and lipid binding (i.e. FFAT, FYVE) domains, and is a membrane protein that regulates vesicular trafficking, and via its interaction with KIF5a, Rab1, VAPB, Surf4, and reticulon proteins, it serves as an adaptor for protein transport within neurons. Dysregulation of its function results in impairment of vesicular trafficking and transport, two important underlying causes of degeneration in motor neurons. ZFYVE27 is reported to be associated mainly with the tubular structure of ER, contributing to the formation of the ER network^67^, and that mutations in ZFYVE27 results in increased susceptibility to ER^67^.

Interestingly, many proteins that interact with ZFYVE27 has already been associated with ALS (e.g. VAPB), HSP (e.g KIF5a, PLP1, ATL1, SPG2, REEP1), and Charcot-Marie Toot disease (e.g. HK1), further confirming a pivotal role for ZFYVE27 in protein interactome domains in many diseases, affecting motor function. We find increased ZFYVE27 expression that is restricted and accumulated in distinct regions of the Betz cell soma in ALS patients. Based on previous reports of ZFYVE27 location in the ER, it is possible that overexpression of ZFYVE27 may contribute to ER dysfunction and ER stress in Betz cells. We previously showed that upper motor neurons become primarily vulnerable when there is ER-stress and that they begin to degenerate before any other cortical neuron^68^. ZFYVE27 may indeed be the protein that links ER dynamics and protein trafficking, especially in motor neurons, and enhancing its function and interaction with other proteins may have profound consequences for improving motor neuron health.

One of the main strengths of our findings is to reveal dysregulation of expression profiles of some of the key proteins in a broad spectrum of ALS patients. We investigated the Betz cells of both sALS patients with unknown mutations, and ALS patients with TDP43 pathology. As one can imagine, this is one of the broad spectrum of coverage of ALS patients, and given the heterogeneity of the disease, it is remarkable that Betz cells of all patients investigated displayed similar cellular pathologies of increased protein expression as in the case of ZFYVE27, PPARG, and PPARGCA1, decreased expression of YWHAZ, and accumulation of ZFYVE27 within the cytoplasm. These findings were comparable between the Betz cells of sALS patients and patients with TDP43 pathology. TDP43+ inclusions is one of the most common pathology in ALS, and we report that TDP43 has 323 binding partners, 11 of which are proteins with their coding genes linked with ALS (Supplementary Table 1, 2). TDP43 binding proteins are present in canonical pathways that are highlighted in this report, suggesting that proteins that accumulate with TDP43 may be depleted in the cytoplasm. In such cases, mutations may not be required in these proteins. Because they are trapped in aggregates, they may lack their ability to function, and that indeed could be the cause of neurodegeneration. Numerous studies using many different model systems highlight the importance of TDP43 pathology with respect to motor neuron degeneration, and here we find that Betz cells of ALS patients with TDP43 and sALS patients display altered expression profiles of proteins that govern important functions as connectors and upstream regulators. It is now important to reveal whether such changes are detrimental and contribute to disease pathology.

In summary, our efforts began to reveal the protein landscape of ALS, and to decipher how these proteins interact with each other, which set of proteins act as the upstream regulators, which canonical pathways they are particularly involved in, and which interaction domains they favor. The ALS proteins suggest that maintaining lipid homeostasis is important for motor neuron circuitry, and PPARG, PPARGCA1 could be important modulators of lipid dynamics. Our studies also reveal dysregulation of both YWHAZ and ZFYVE27, two key proteins at the heart of many important canonical pathways and protein interaction networks, especially in the Betz cells of both sALS patients and ALS patients with TDP43 pathology. Since all cellular activities are carried by proteins and their interactions, understanding the protein landscape of ALS will help us uncover the underlying cellular mechanisms that are perturbed, and how that imbalance contributes to neuronal vulnerability and degeneration in diseases.

## Electronic supplementary material

All Supplementary Figures

All Supplementary Tables
