# Supplementary material for: Protein-protein interactions reveal key canonical pathways, upstream regulators, interactome domains, and novel targets in ALS
Source: Sci Rep. 2018 Oct 3;8:14732. doi: 10.1038/s41598-018-32902-4 (PMC6170493; doi:10.1038/s41598-018-32902-4)
Supplement: Supplementary file 1 — All Supplementary Figures [file 41598_2018_32902_MOESM1_ESM.pdf]

Supplementary Information-Figures

Title: Protein-protein interactions reveal key canonical pathways, upstream regulators, interactome domains, and novel targets in ALS

Manuscript number: SREP-18-22830A

Authors: Ina Dervishi[1], Oge Gozutok[1], Kevin Murnan[1], Mukesh Gautam[1], Daniel Heller[1], Eileen Bigio[2,3], and P. Hande Ozdinler[1,2,4,5]

[1]Department of Neurology, Northwestern University, Feinberg School of Medicine, [2]Mesulam Cognitive Neurology and Alzheimer Disease Center, [3]Department of Pathology, Northwestern University, Chicago, IL 60611, USA, [4]Les Turner ALS Center, [5]Robert H. Lurie Comprehensive Cancer Center, Northwestern University, Feinberg School of Medicine, Chicago, IL, 60611

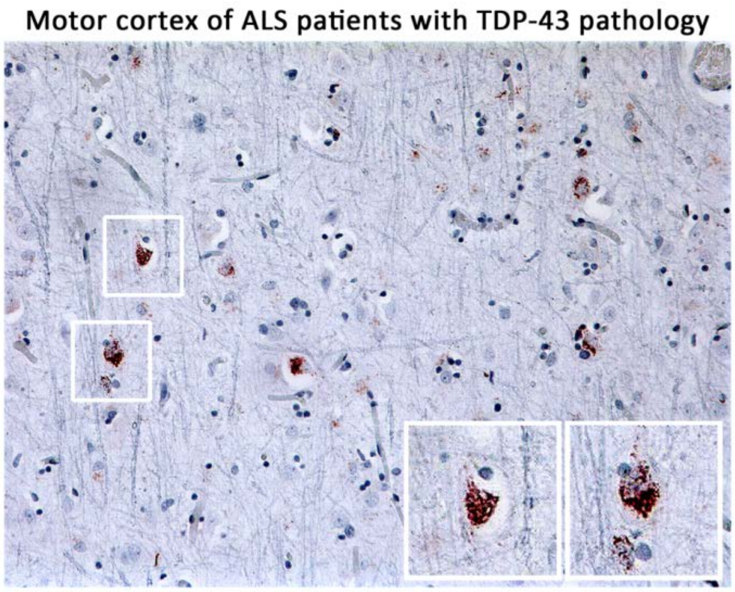

Supplementary Figure 1 Motor cortex of ALS patients with TDP-43 pathology. A represantative image of the motor cortex, which shows aggregates of phosphorylated TDP-43, confirming TDP-43 pathology. Boxed areas are enlarged within the figure.

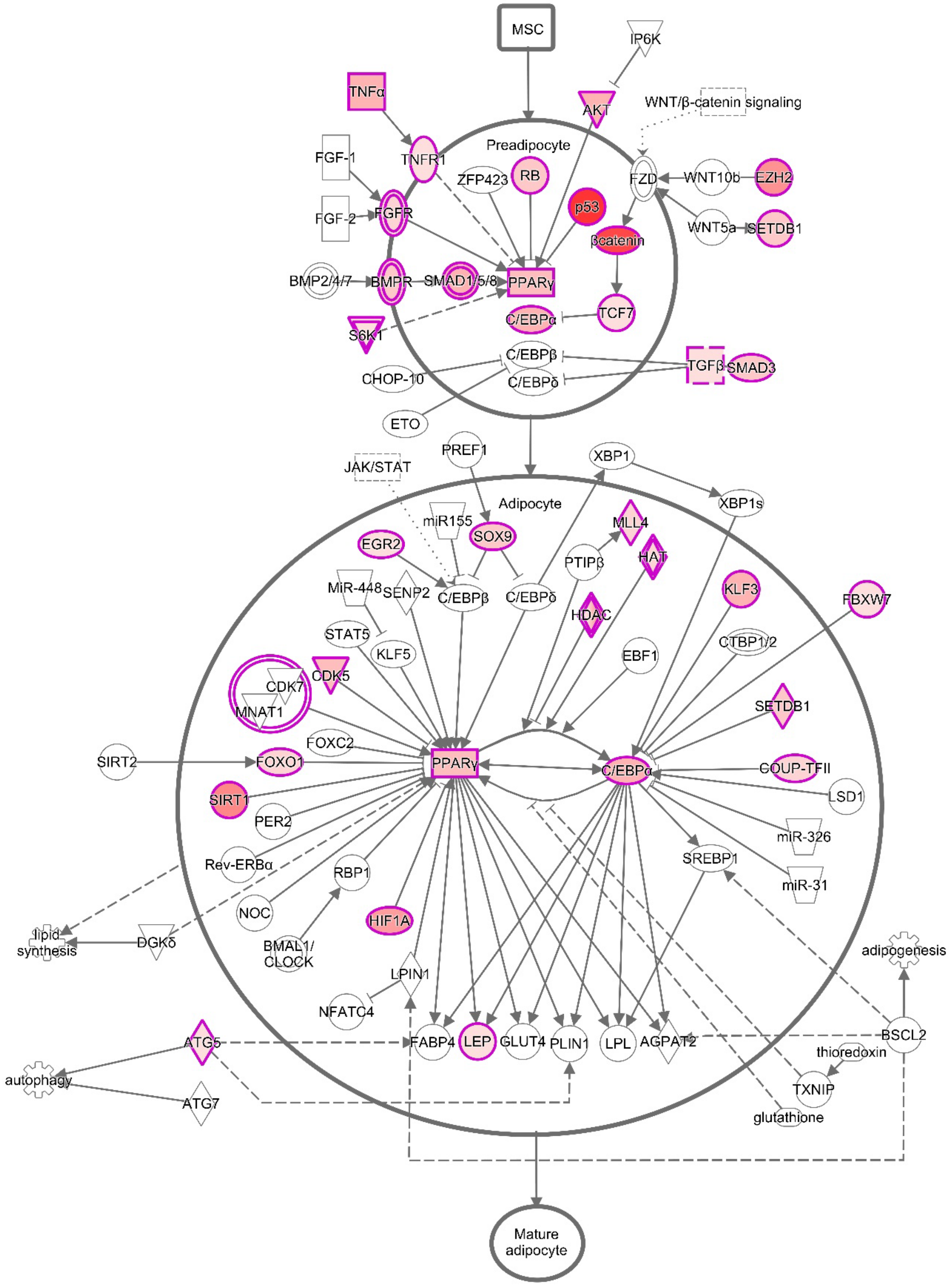

Supplementary Figure 2 Image of Adipogenesis pathway, representing the location and the extent of ALS protein involvement. ALS proteins with higher number of binding partners are marked with increasing color intensity. Obtained from IPA.

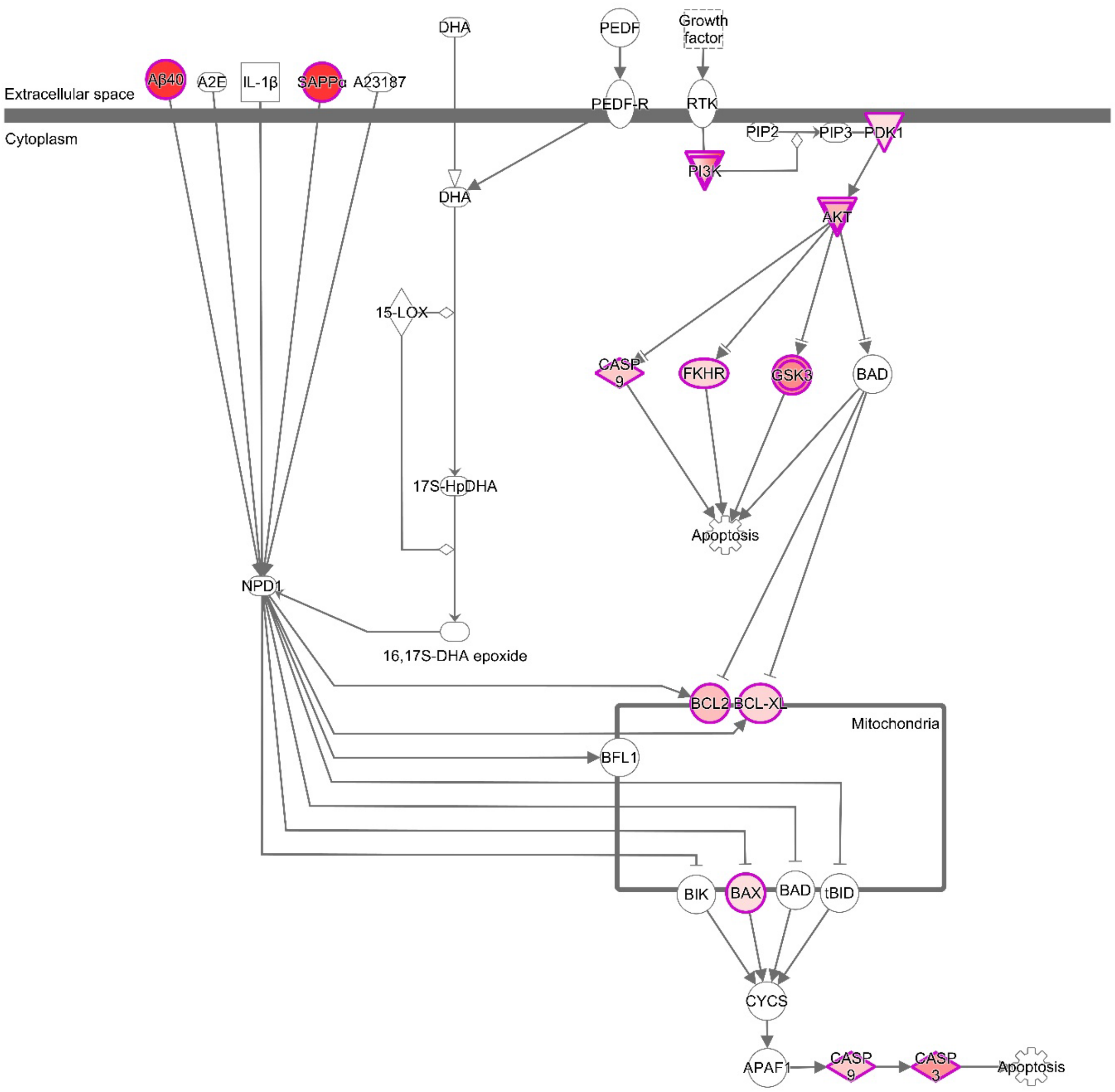

Supplementary Figure 3 Image of Docosahexaenoic acid signaling pathway, representing the location and the extent of ALS protein involvement. ALS proteins with higher number of binding partners are marked with increasing color intensity. Obtained from IPA.

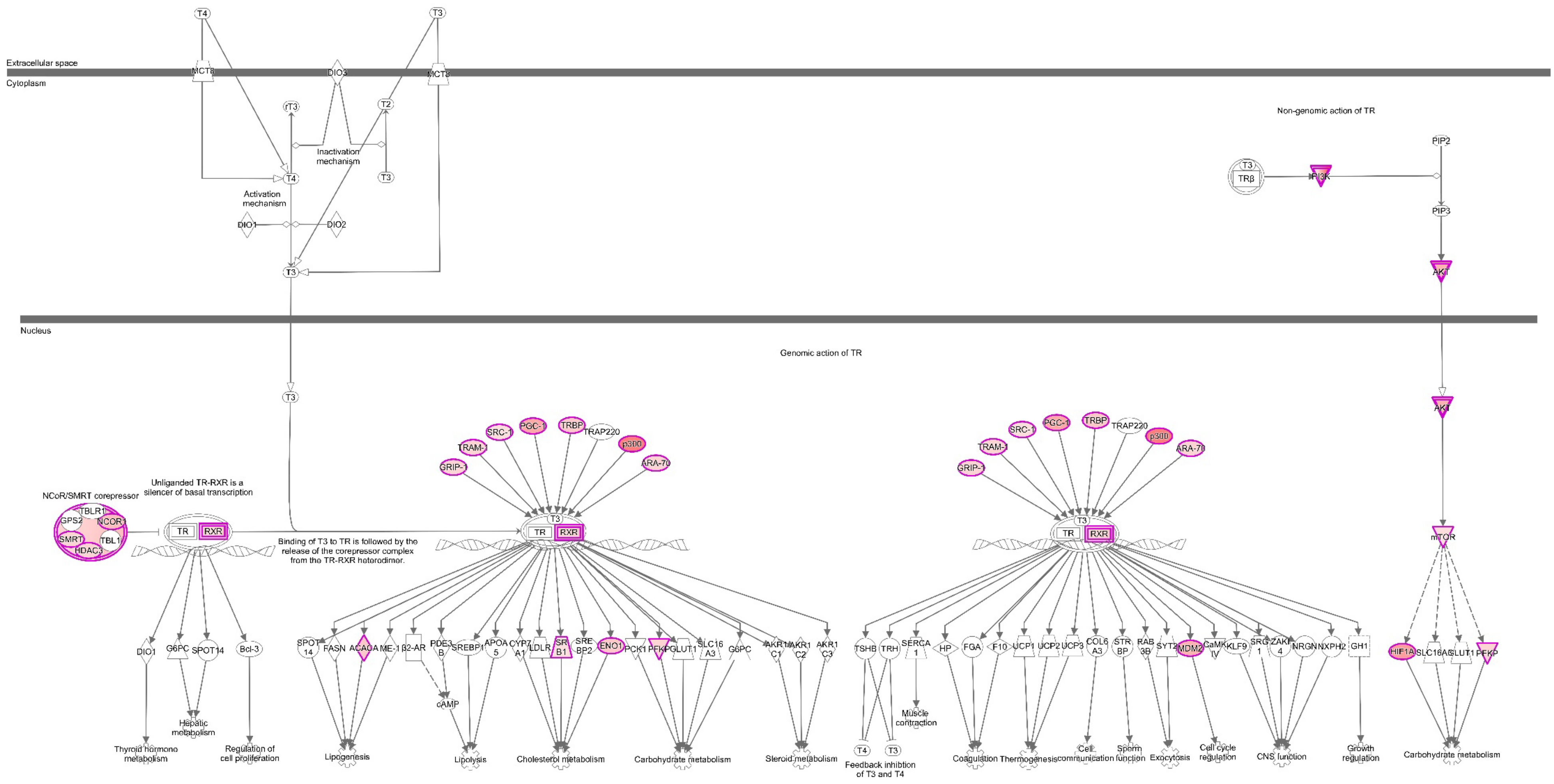

Supplementary Figure 4 Image of TX/RxR activation pathway, representing the location and the extent of ALS protein involvement. ALS proteins with higher number of binding partners are marked with increasing color intensity. Obtained from IPA.

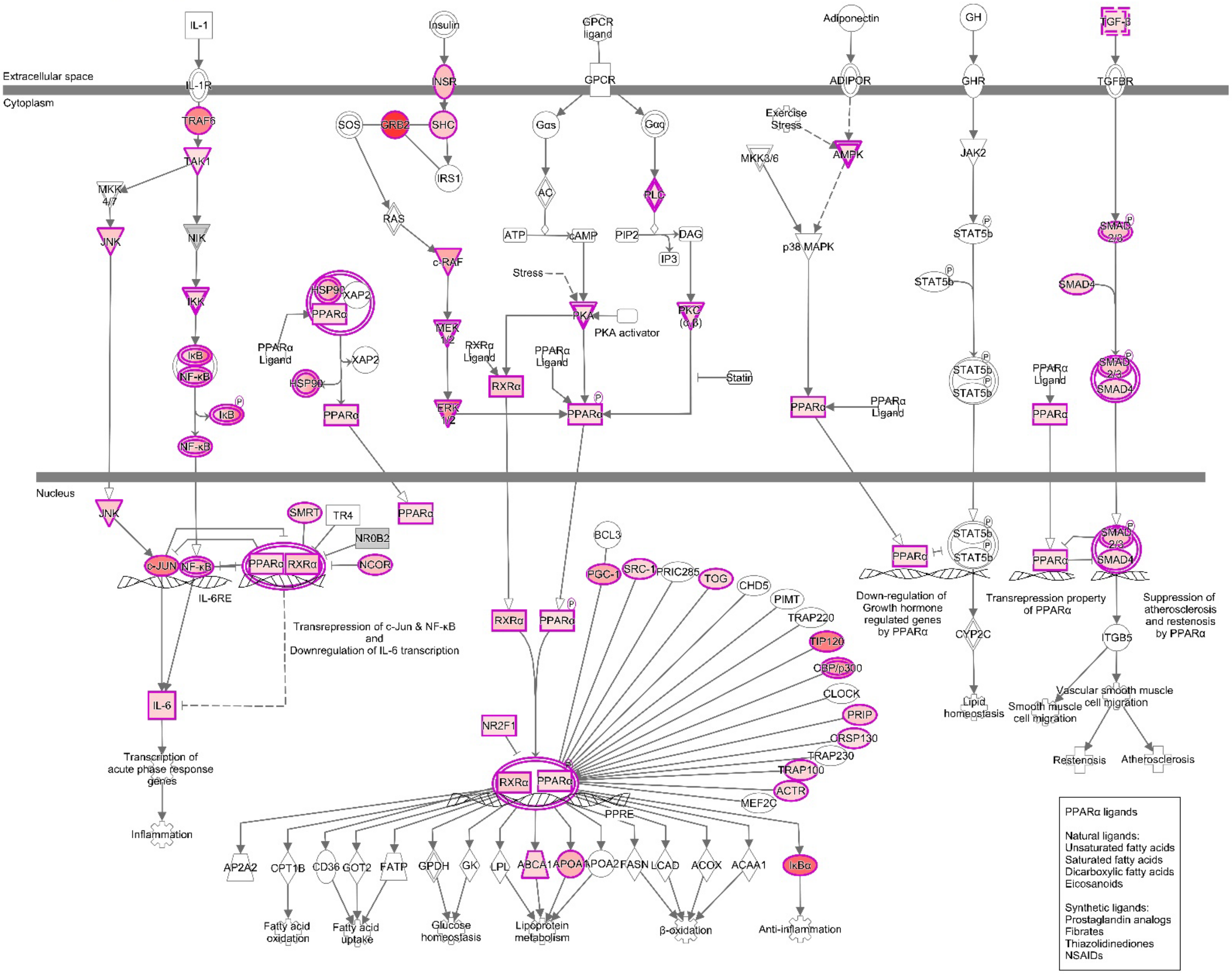

Supplementary Figure 5 Image of PPARa RxR signaling pathway, representing the location and the extent of ALS protein involvement. ALS proteins with higher number of binding partners are marked with increasing color intensity. Obtained from IPA.

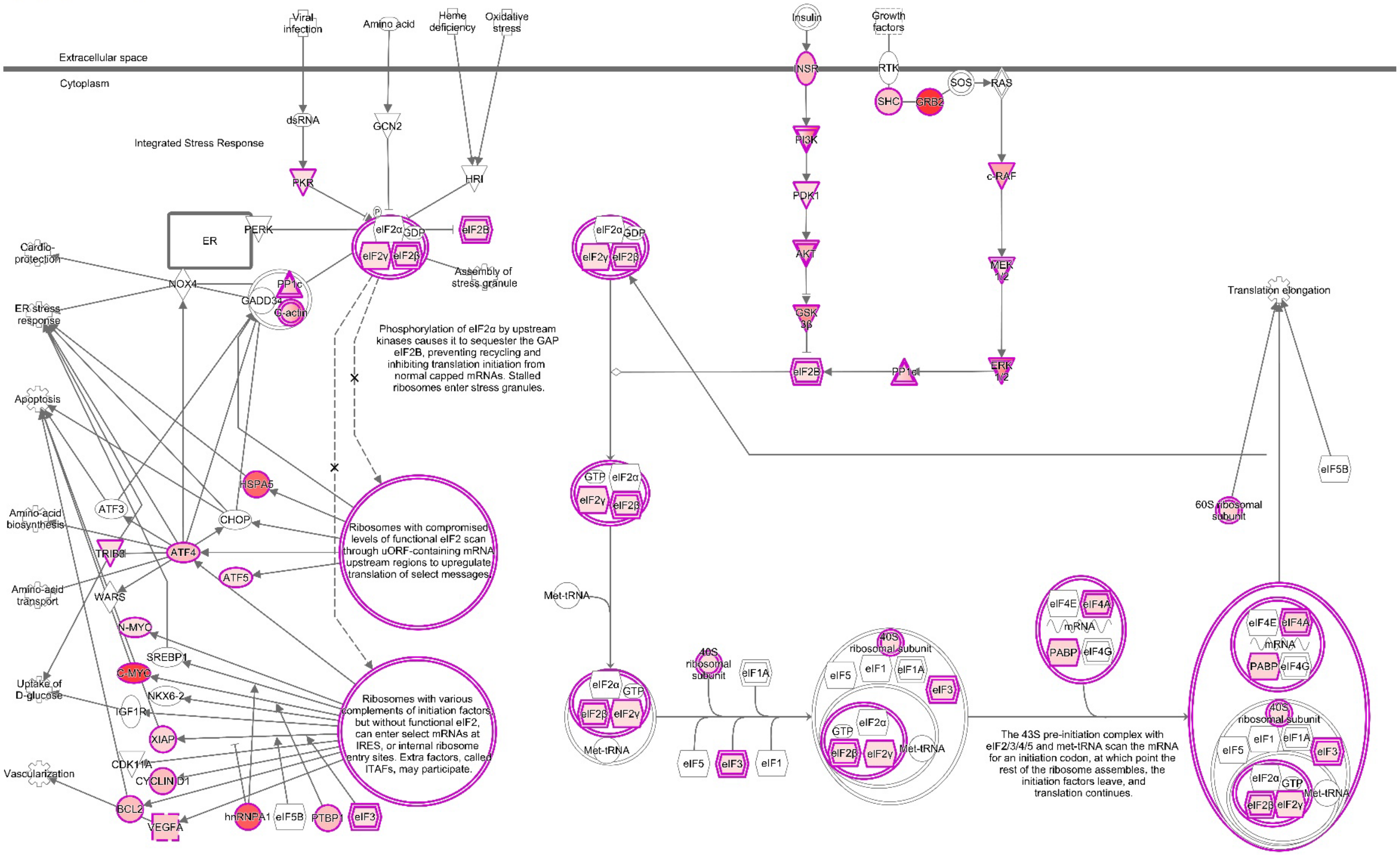

Supplementary Figure 6 Image of EIF2 signaling pathway, representing the location and the extent of ALS protein involvement. ALS proteins with higher number of binding partners are marked with increasing color intensity. Obtained from IPA.

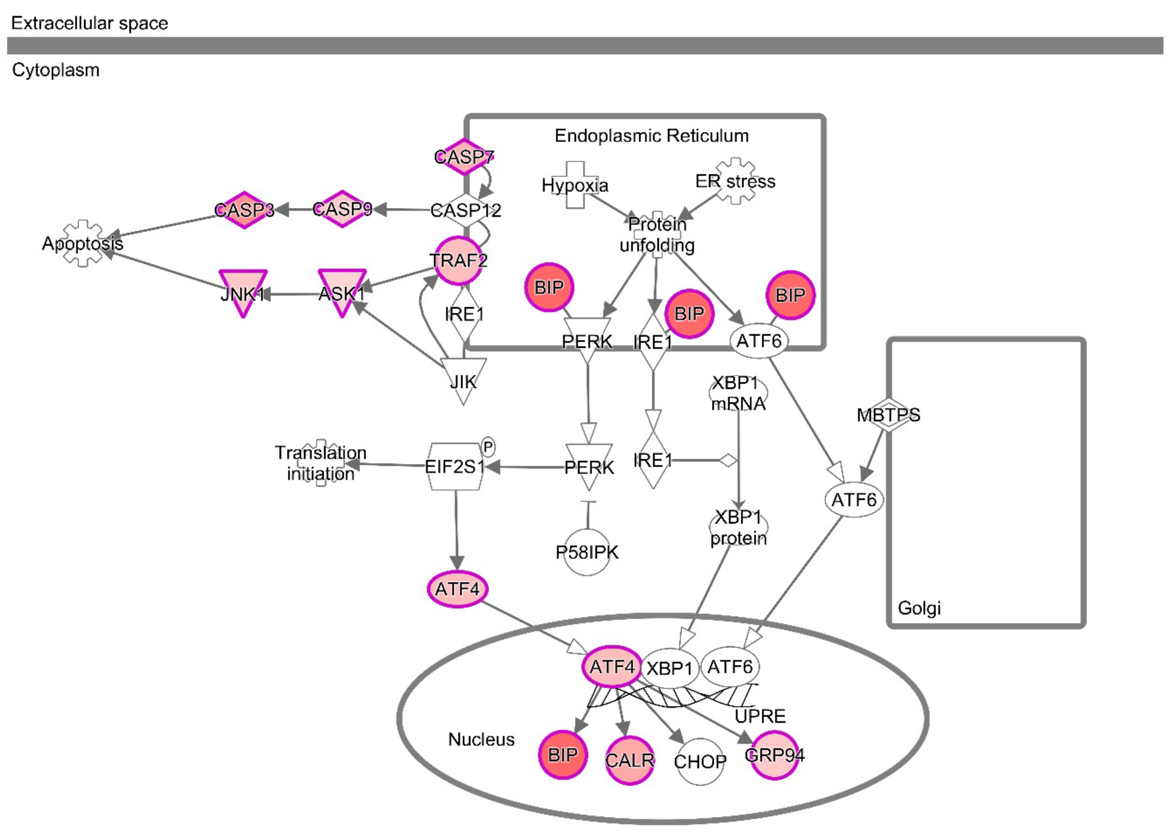

Supplementary Figure 7 Image of ER stress pathway, representing the location and the extent of ALS protein involvement. ALS proteins with higher number of binding partners are marked with increasing color intensity. Obtained from IPA.

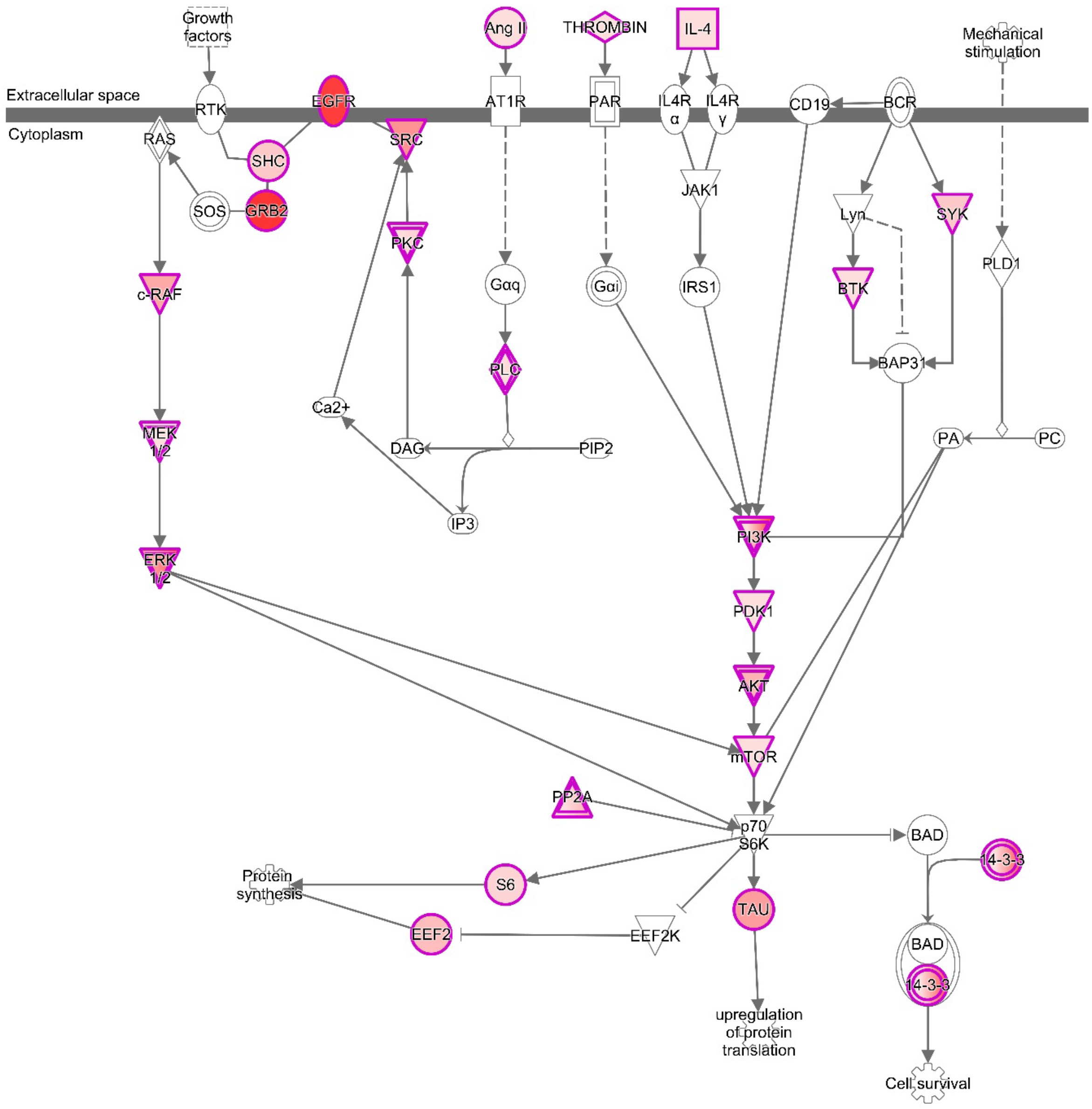

Supplementary Figure 8 Image of P70S6K protein response pathway, representing the location and the extent of ALS protein involvement. ALS proteins with higher number of binding partners are marked with increasing color intensity. Obtained from IPA.

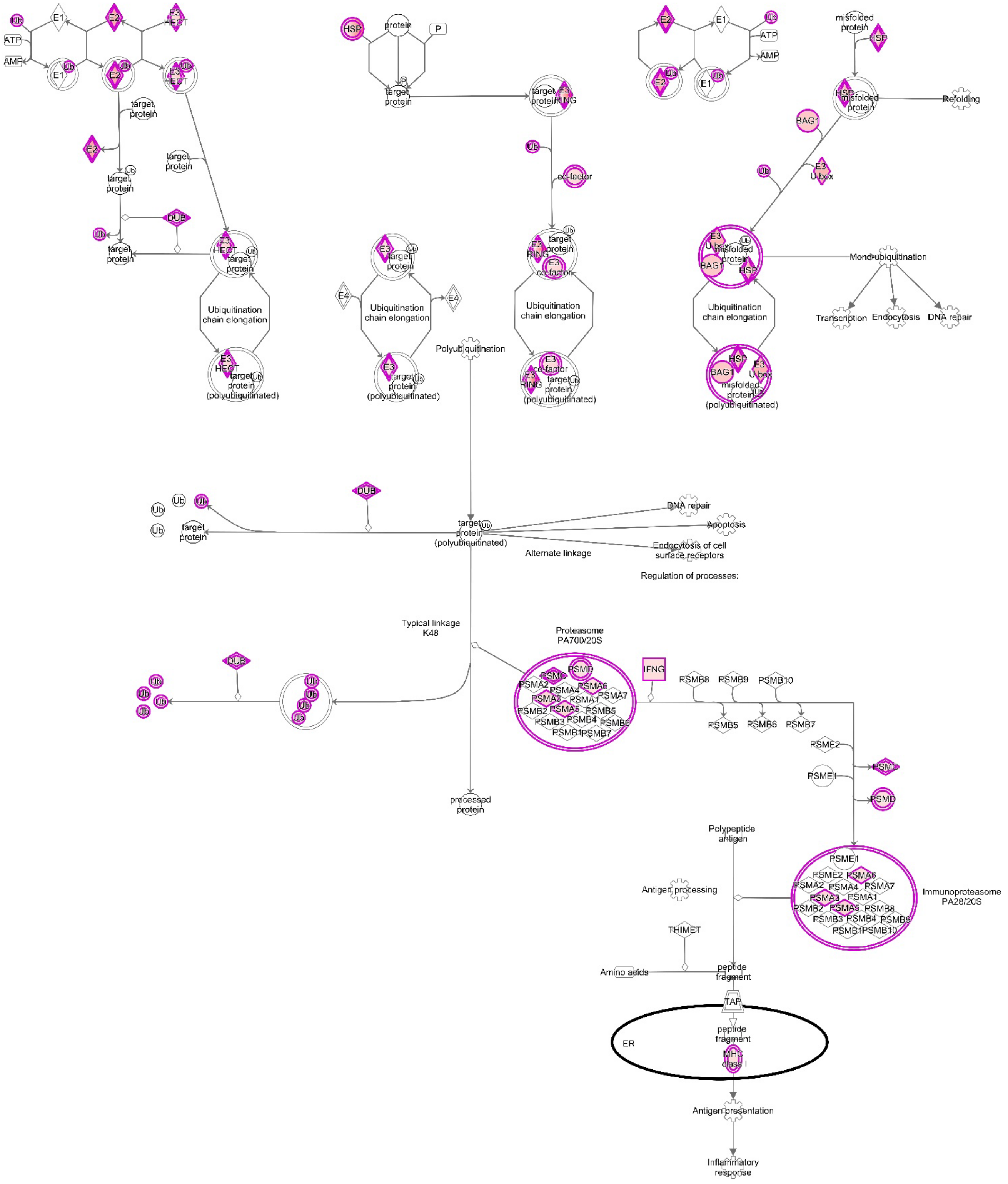

Supplementary Figure 9 Image of Protein ubiquitination pathway, representing the location and the extent of ALS protein involvement. ALS proteins with higher number of binding partners are marked with increasing color intensity. Obtained from IPA.

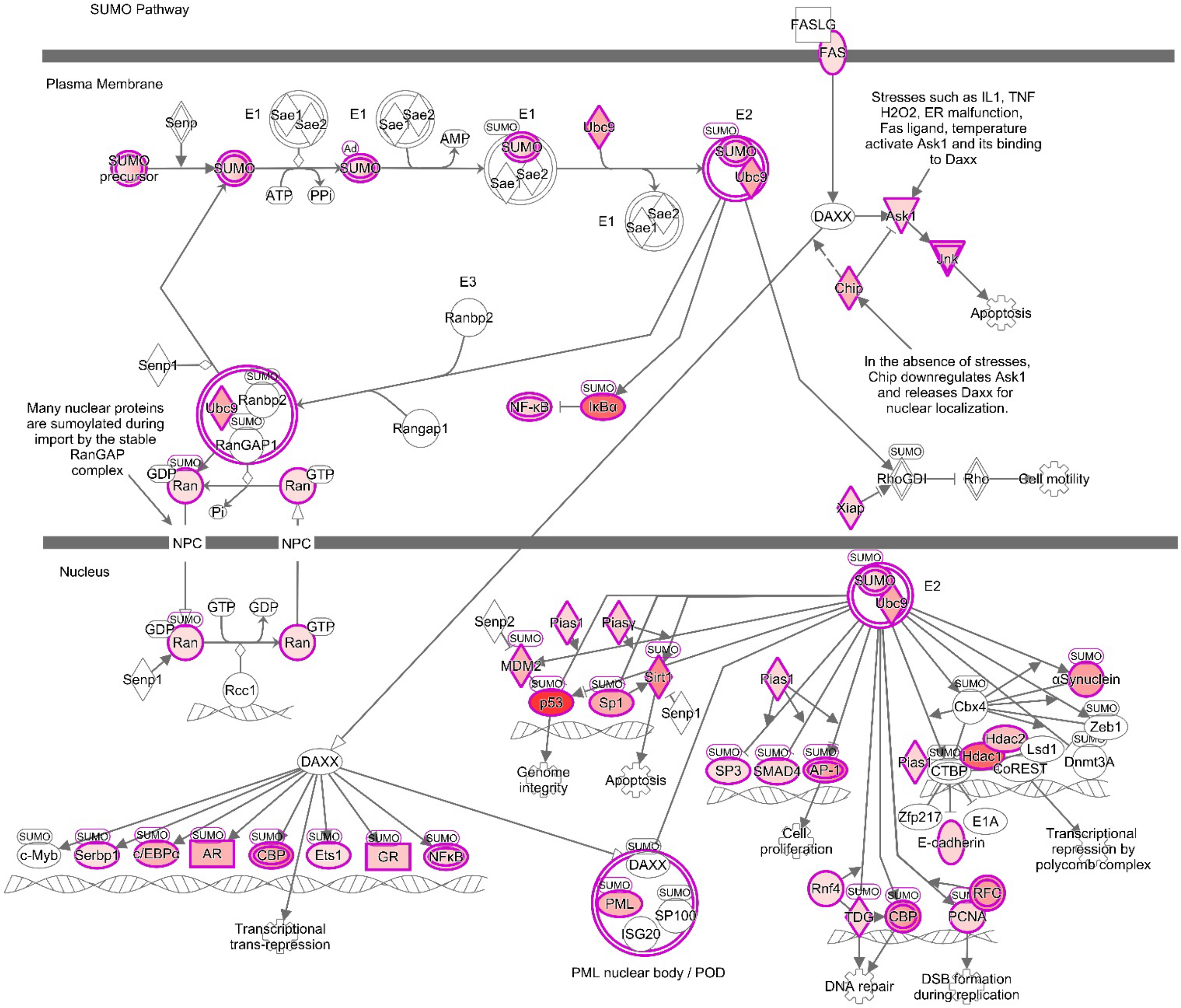

Supplementary Figure 10 Image of Sumoylation pathway, representing the location and the extent of ALS protein involvement. ALS proteins with higher number of binding partners are marked with increasing color intensity. Obtained from IPA.

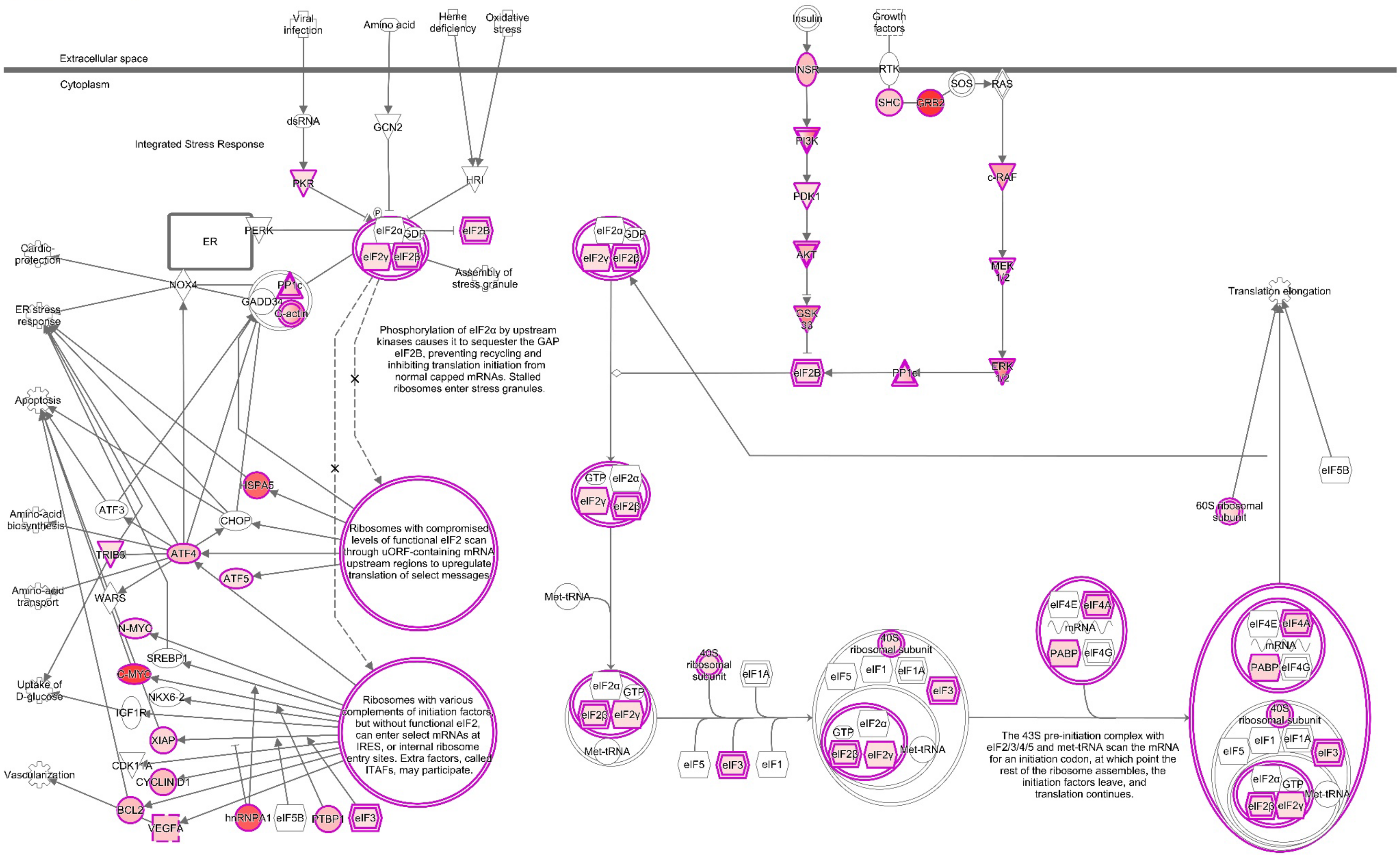

Supplementary Figure 11 Image of Hif1a signaling pathway, representing the location and the extent of ALS protein involvement. ALS proteins with higher number of binding partners are marked with increasing color intensity. Obtained from IPA.

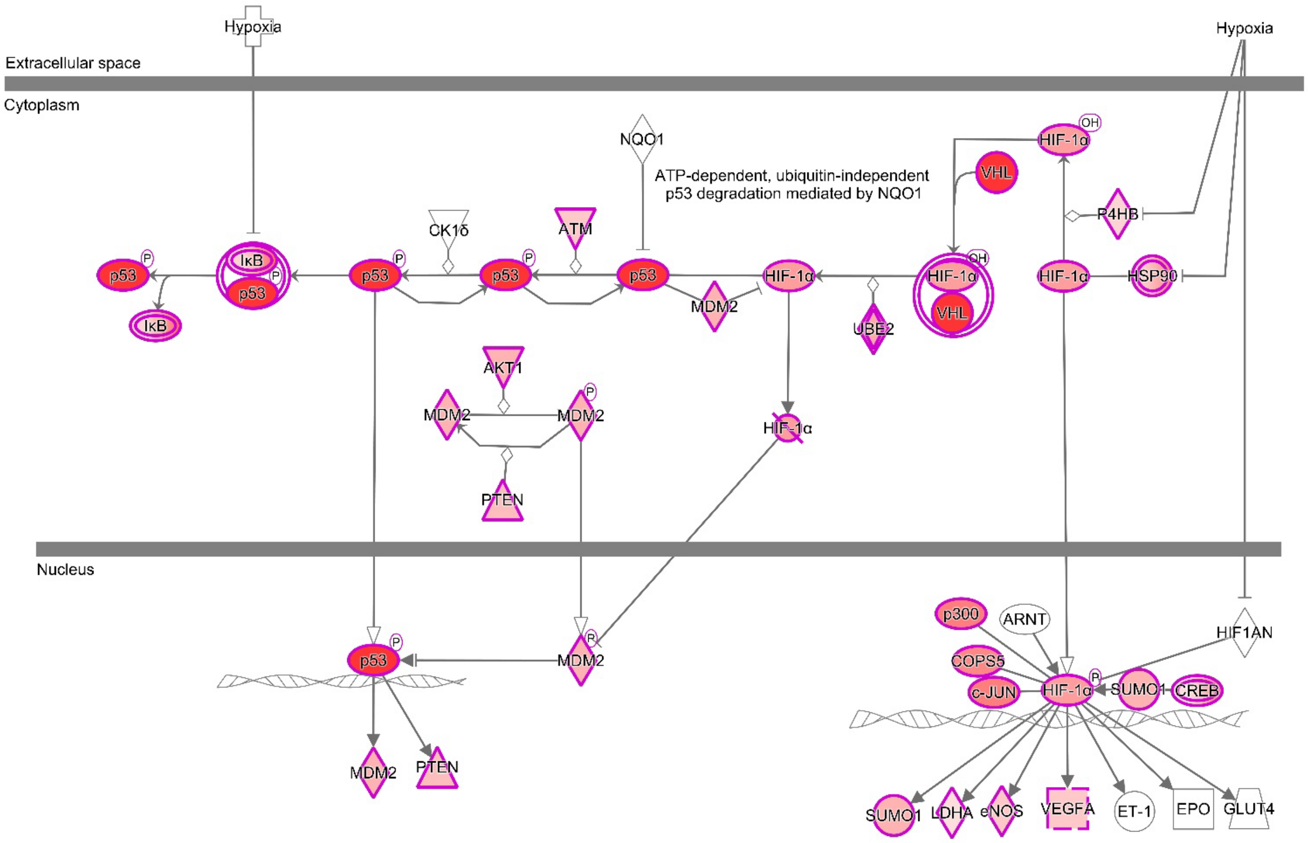

Supplementary Figure 12 Image of Hypoxic signaling pathway, representing the location and the extent of ALS protein involvement. ALS proteins with higher number of binding partners are marked with increasing color intensity. Obtained from IPA.

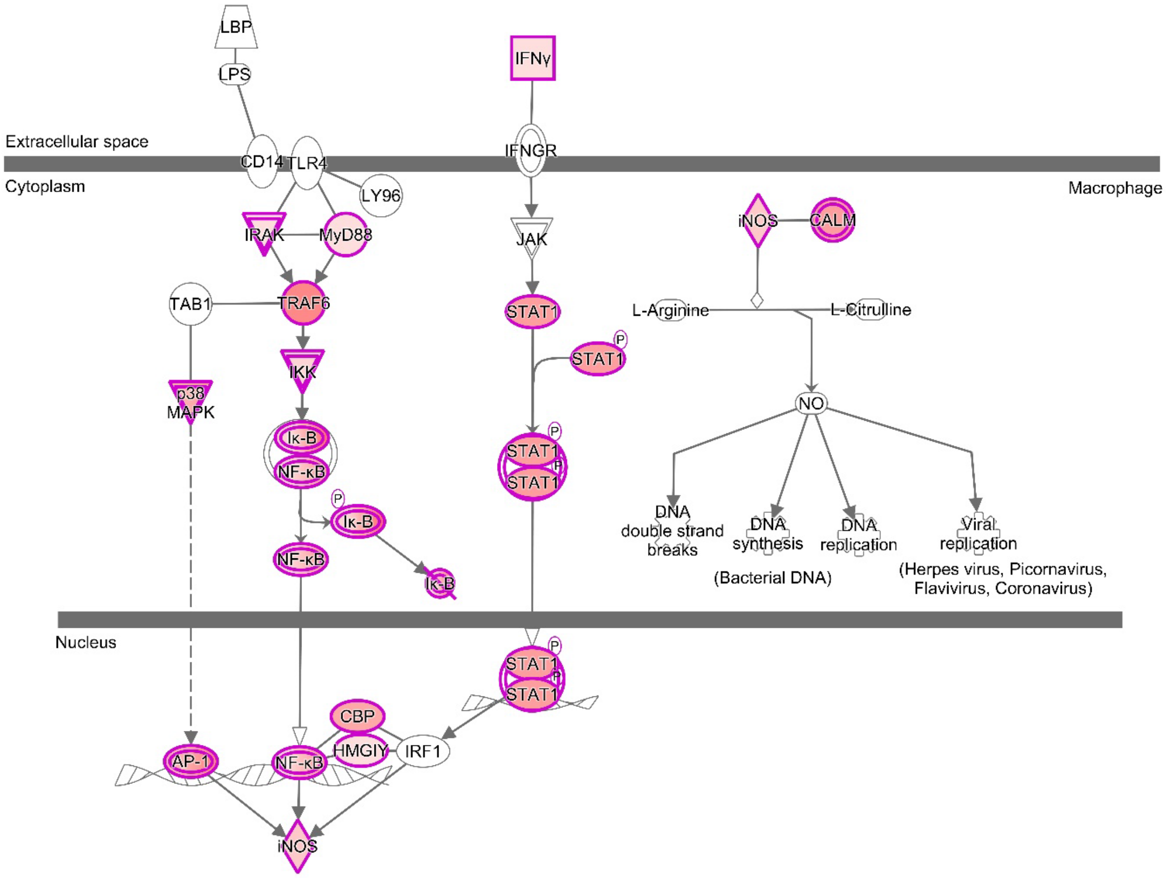

Supplementary Figure 13 Image of iNOS signaling pathway, representing the location and the extent of ALS protein involvement. ALS proteins with higher number of binding partners are marked with increasing color intensity. Obtained from IPA.

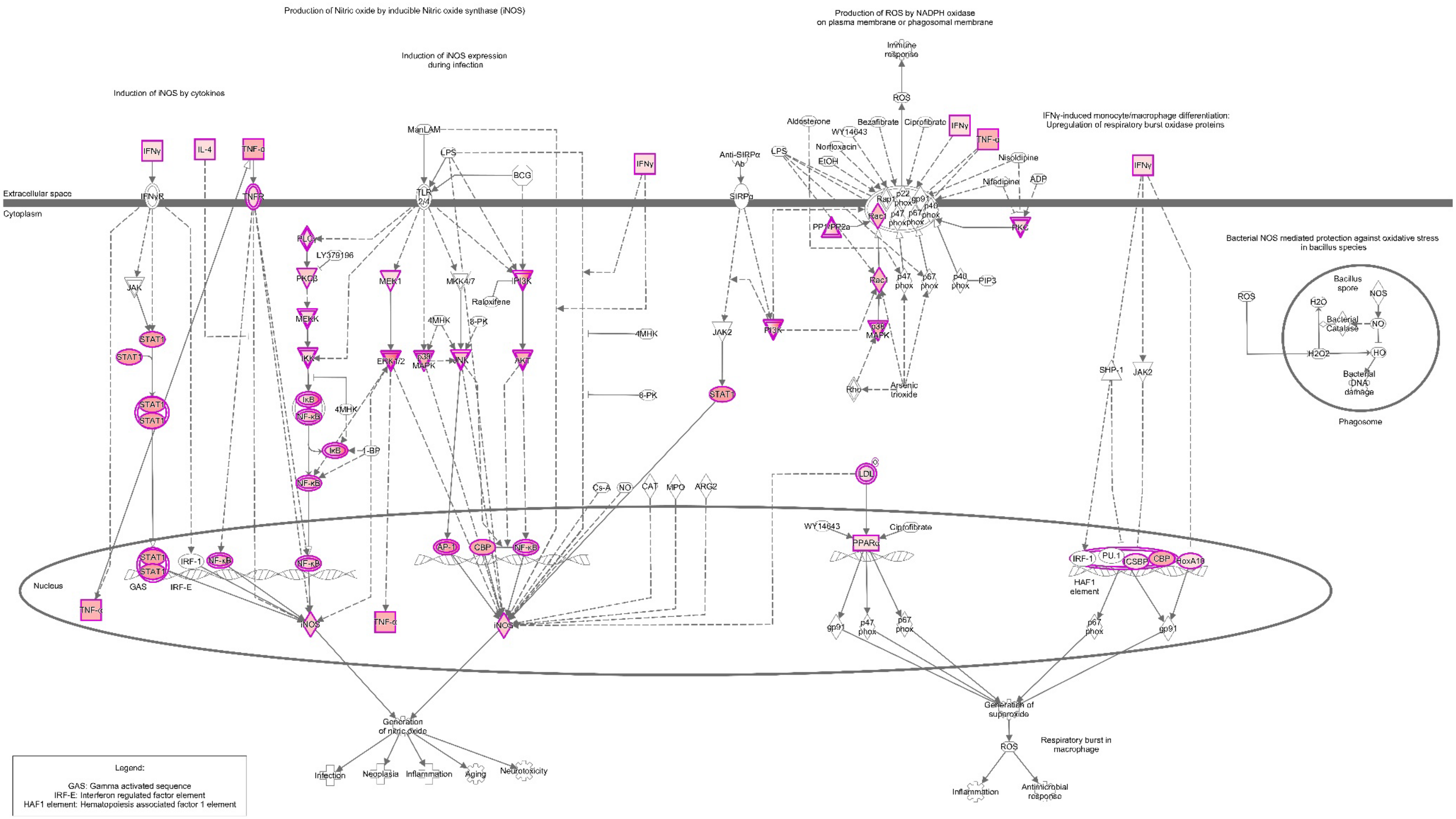

Supplementary Figure 14 Image of Production of NO and ROS signaling pathway, representing the location and the extent of ALS protein involvement. ALS proteins with higher number of binding partners are marked with increasing color intensity. Obtained from IPA.

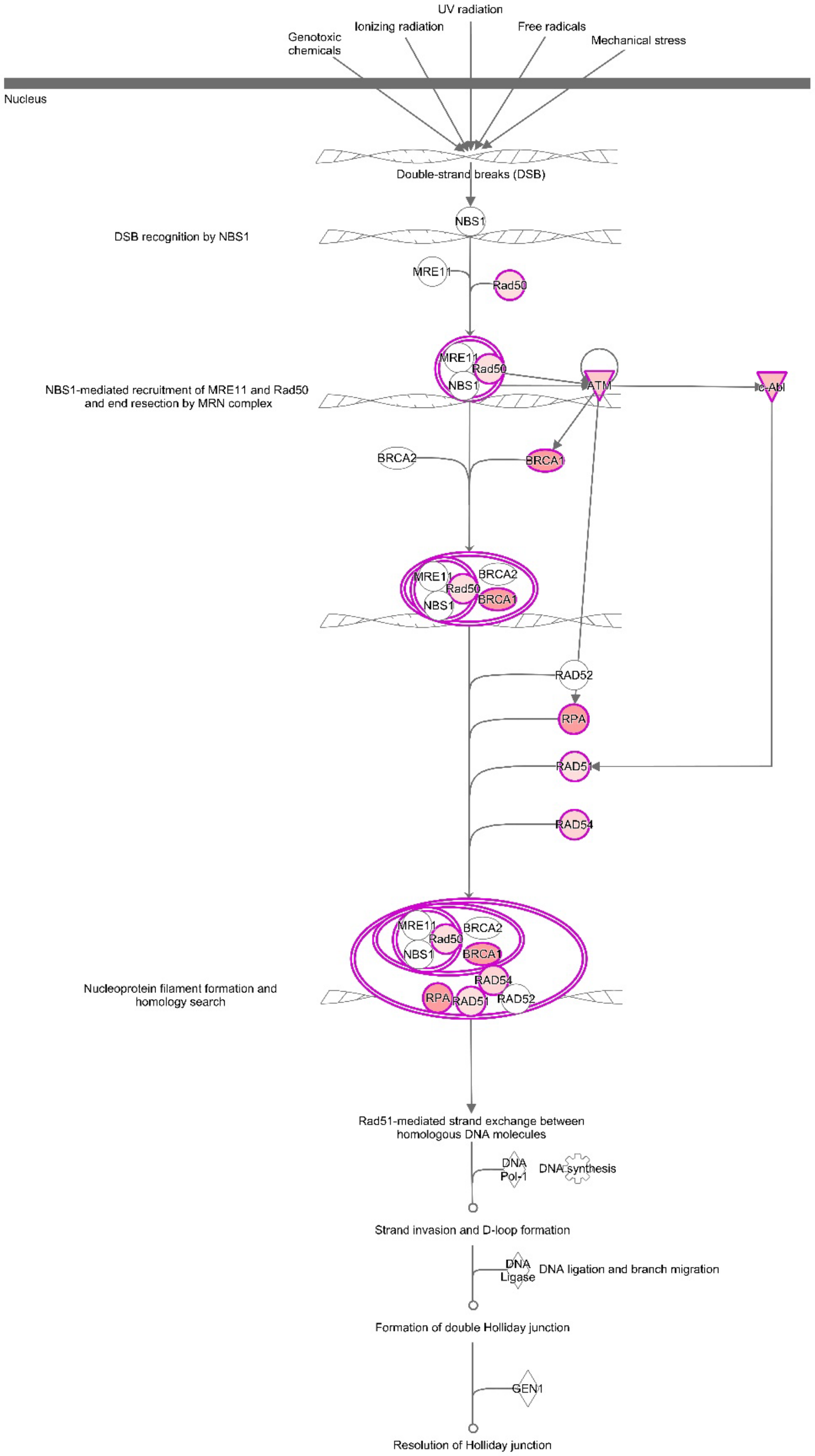

Supplementary Figure 15 Image of DNA double strand break repaired by homologous recombination pathway, representing the location and the extent of ALS protein involvement. ALS proteins with higher number of binding partners are marked with increasing color intensity. Obtained from IPA.

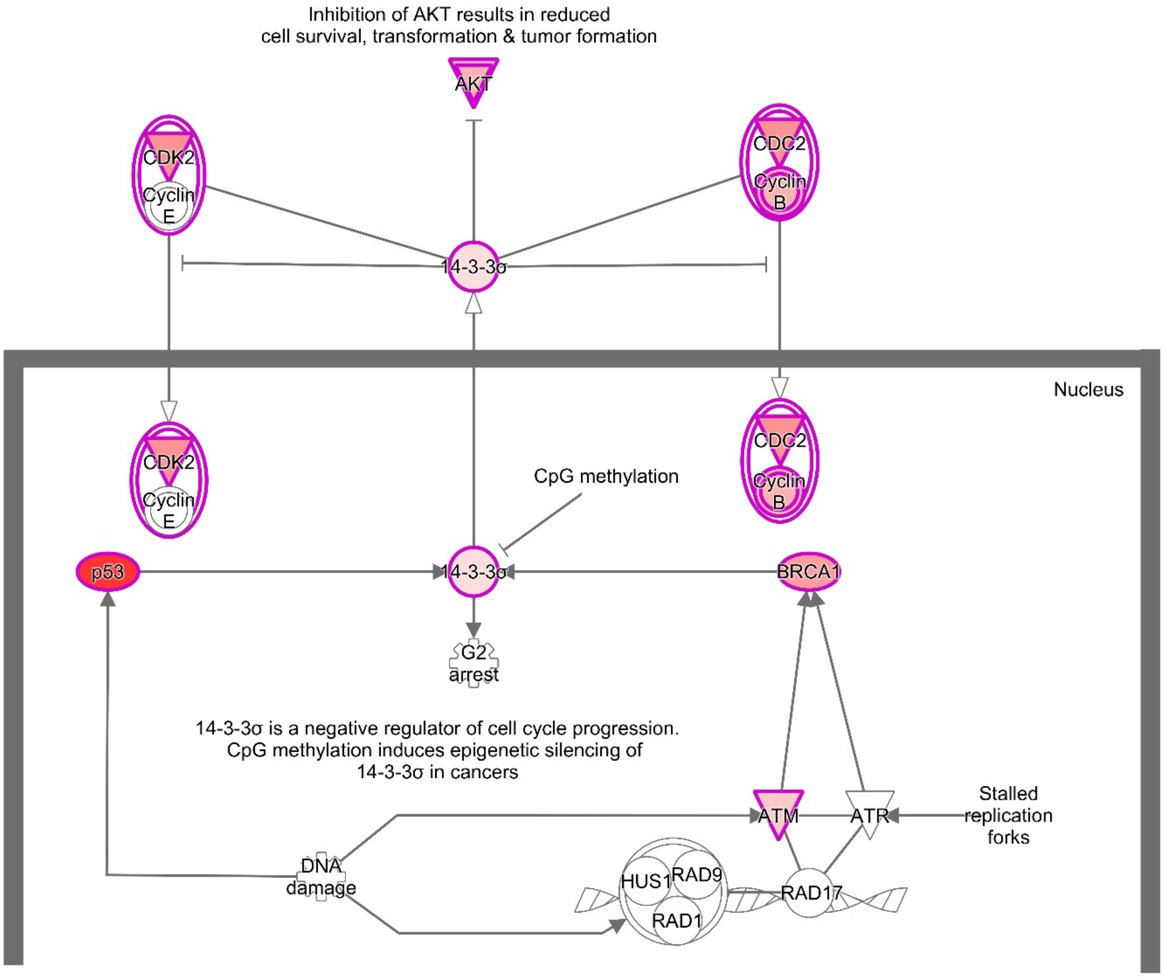

Supplementary Figure 16 Image of DNA damage induced 14-3-3 signaling pathway, representing the location and the extent of ALS protein involvement. ALS proteins with higher number of binding partners are marked with increasing color intensity. Obtained from IPA.

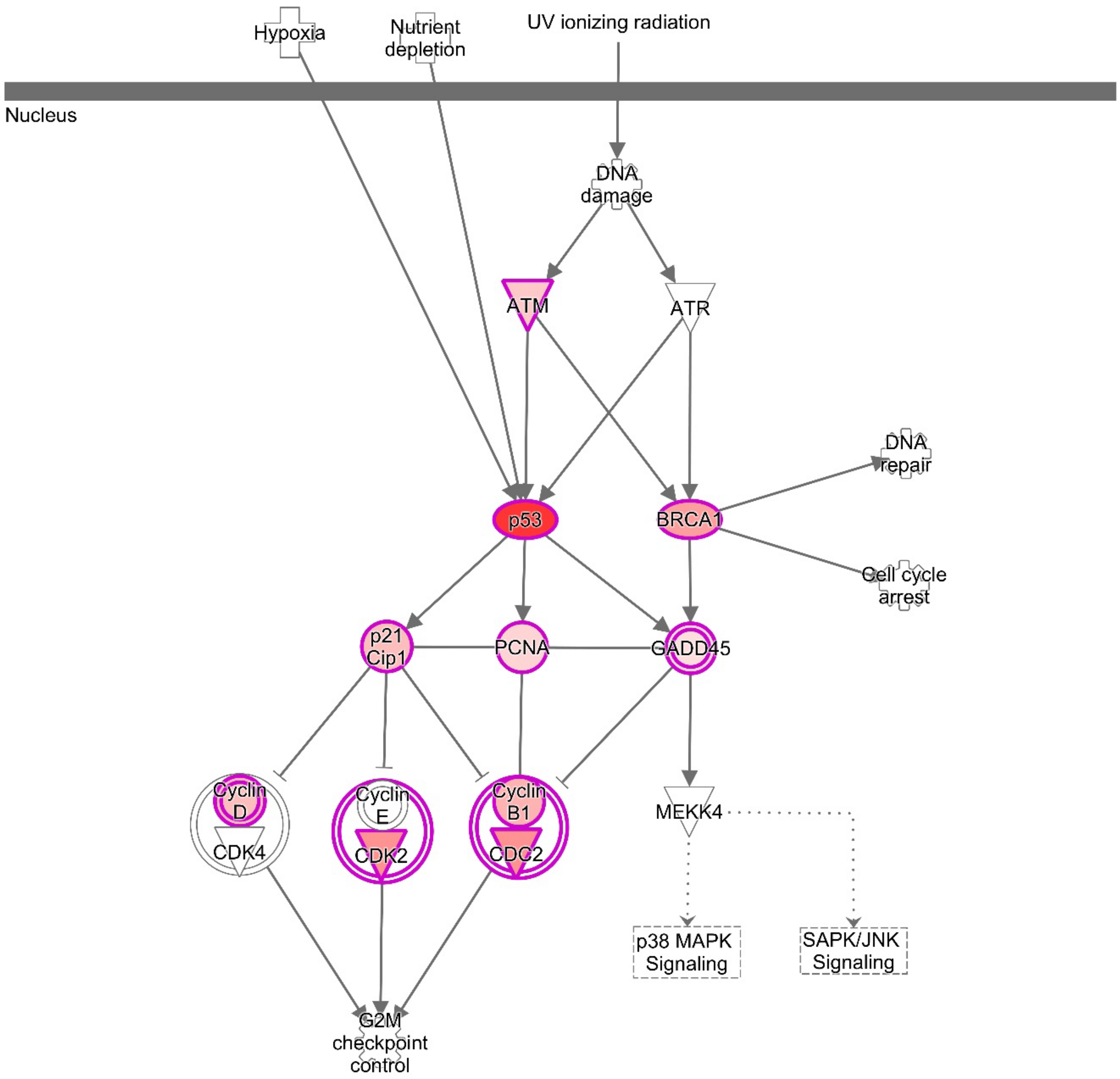

Supplementary Figure 17 Image of GADD45 signaling pathway, representing the location and the extent of ALS protein involvement. ALS proteins with higher number of binding partners are marked with increasing color intensity. Obtained from IPA.

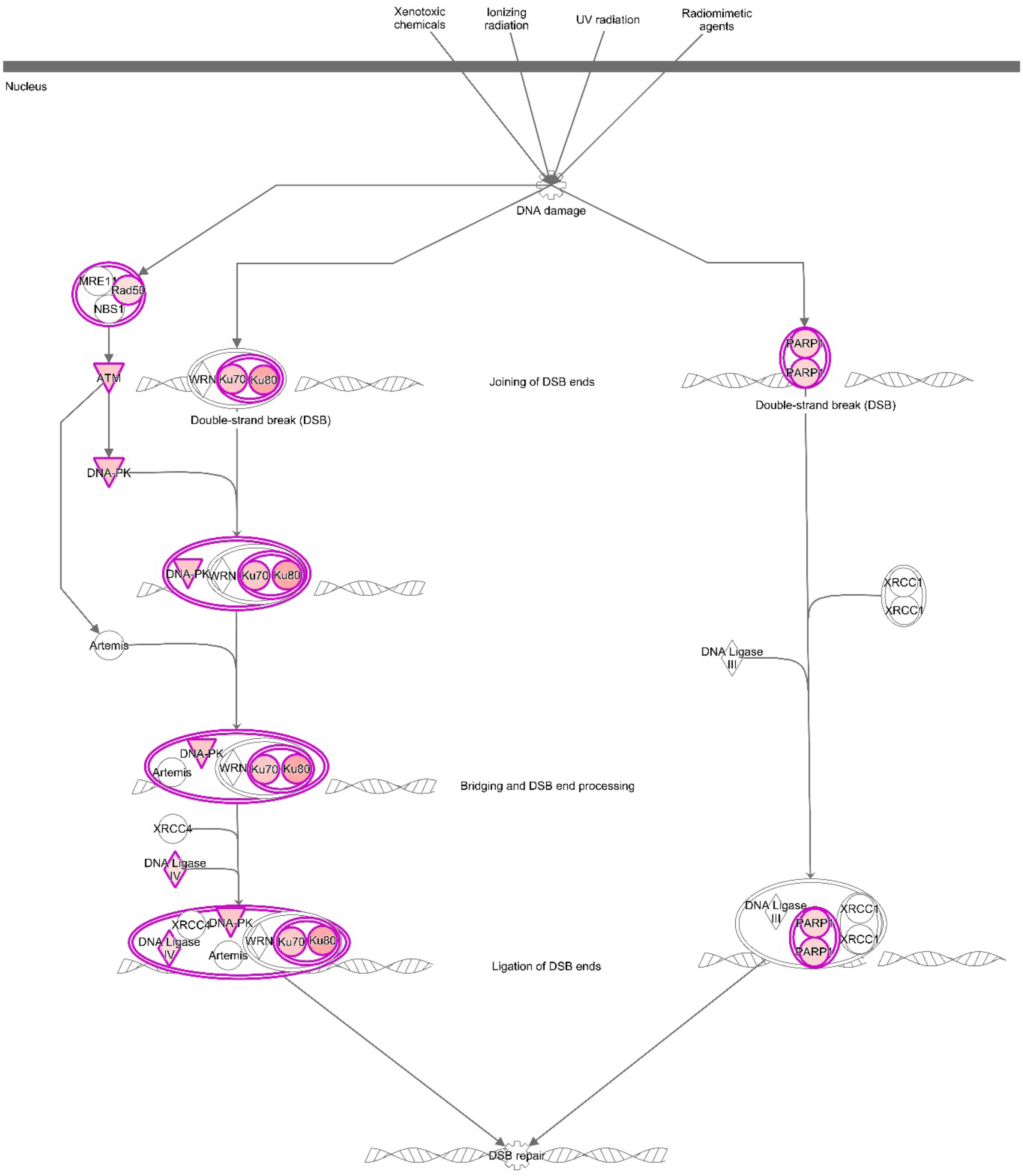

Supplementary Figure 18 Image of DNA double strand break repaired by non-homologous end joining pathway, representing the location and the extent of ALS protein involvement. ALS proteins with higher number of binding partners are marked with increasing color intensity. Obtained from IPA.

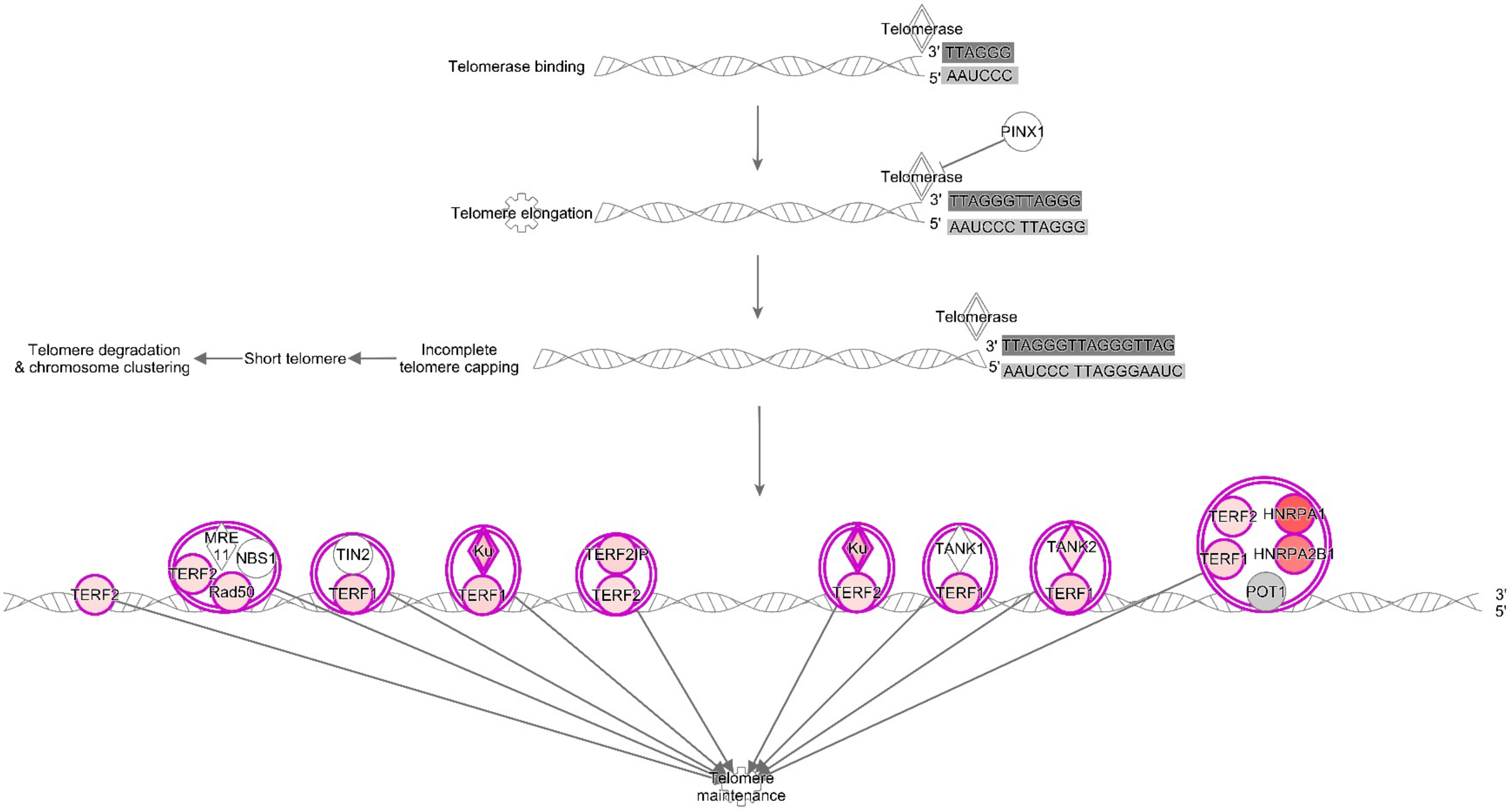

Supplementary Figure 19 Image of telomere extension by telomerase pathway, representing the location and the extent of ALS protein involvement. ALS proteins with higher number of binding partners are marked with increasing color intensity. Obtained from IPA.

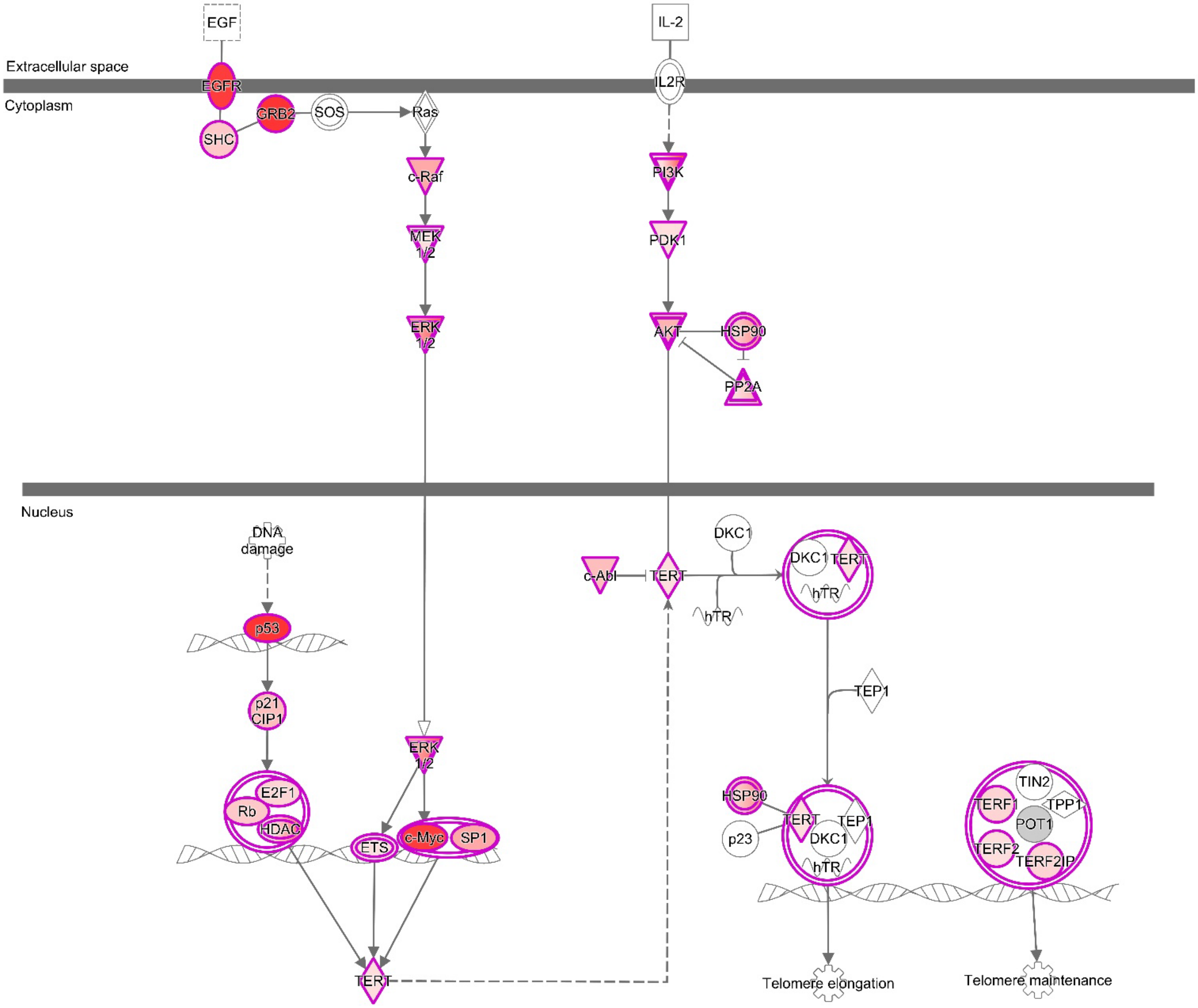

Supplementary Figure 20 Image of Telomerase signaling pathway, representing the location and the extent of ALS protein involvement. ALS proteins with higher number of binding partners are marked with increasing color intensity. Obtained from IPA.

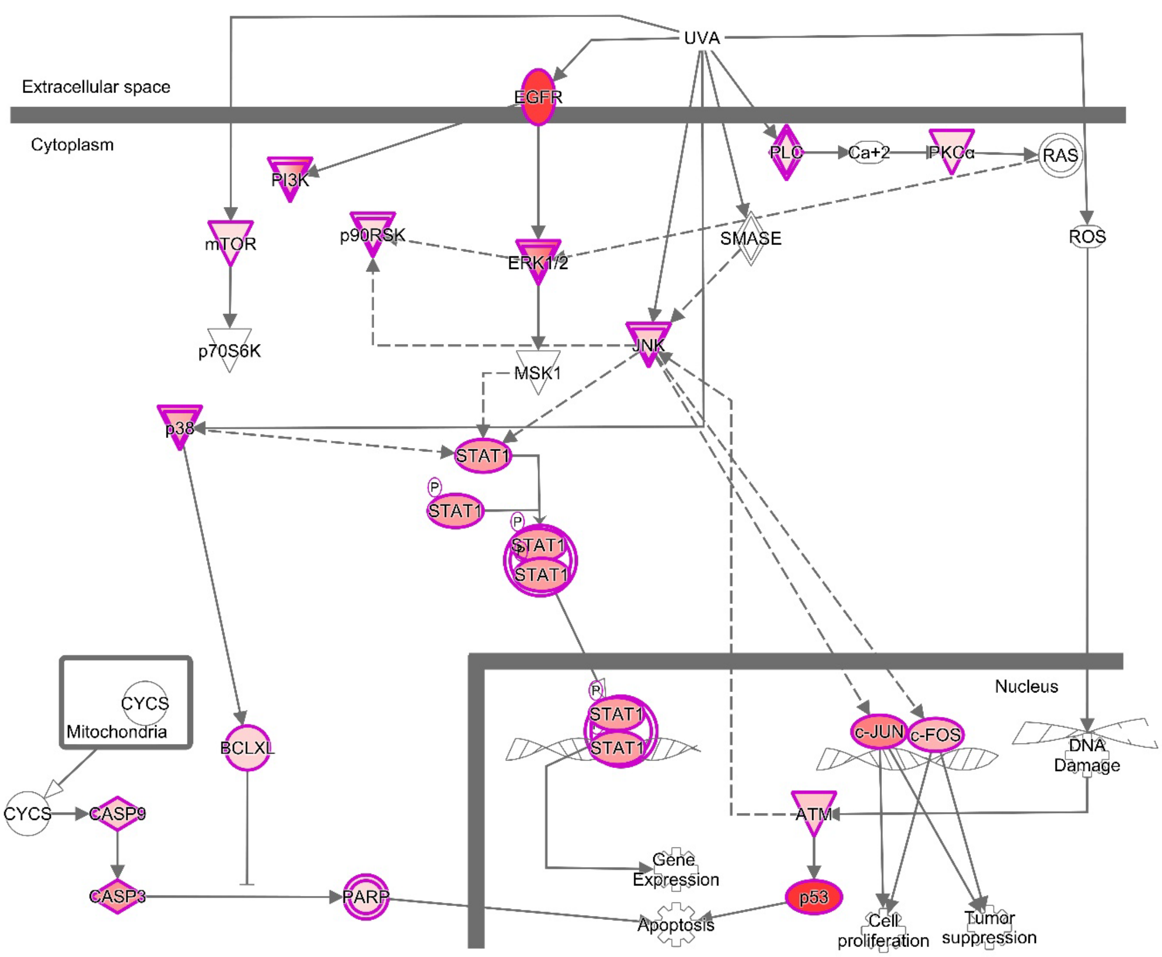

Supplementary Figure 21 Image of UVA induce MAPK signaling pathway, representing the location and the extent of ALS protein involvement. ALS proteins with higher number of binding partners are marked with increasing color intensity. Obtained from IPA.

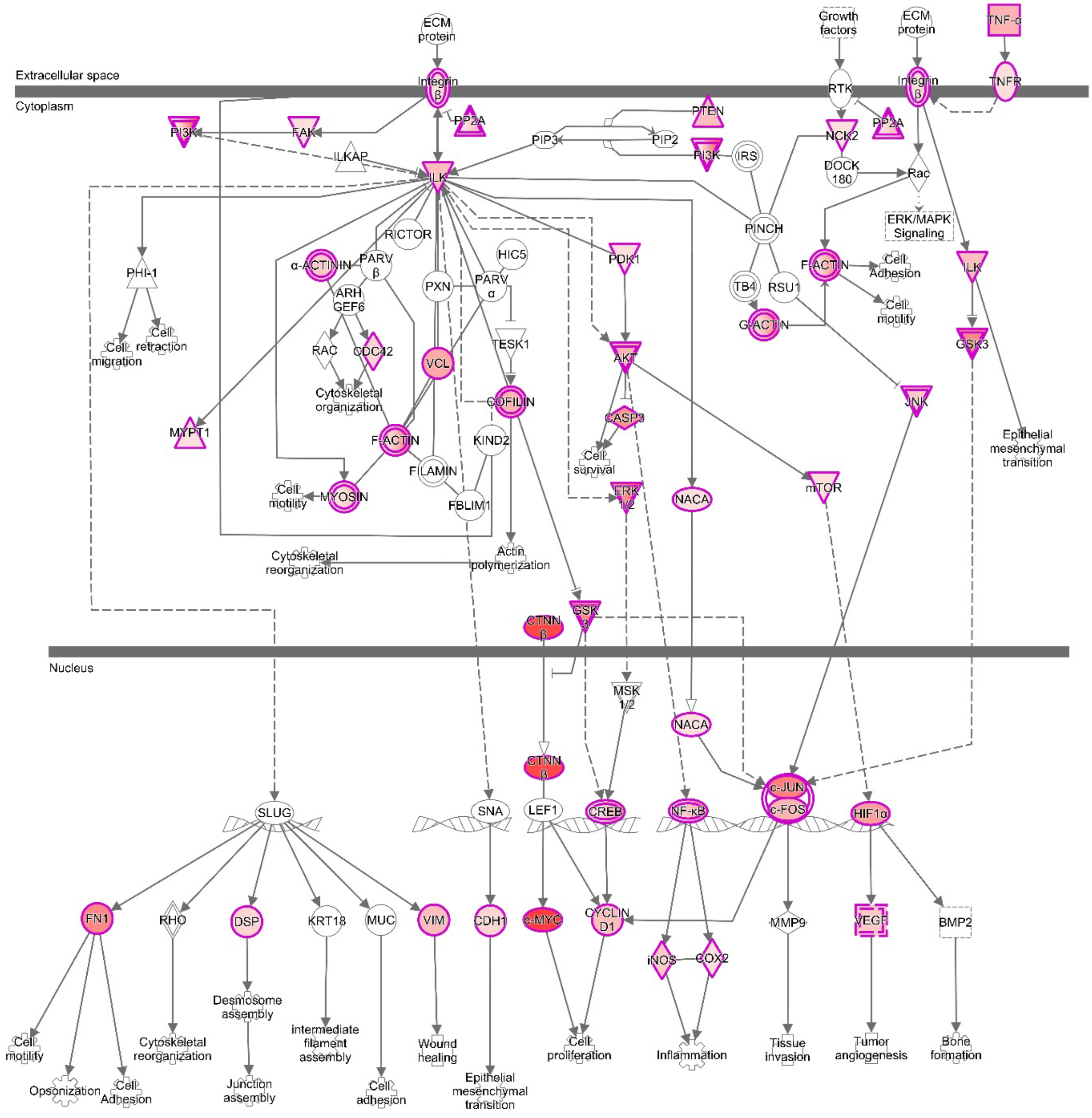

Supplementary Figure 22 Image of ILK signaling pathway, representing the location and the extent of ALS protein involvement. ALS proteins with higher number of binding partners are marked with increasing color intensity. Obtained from IPA.

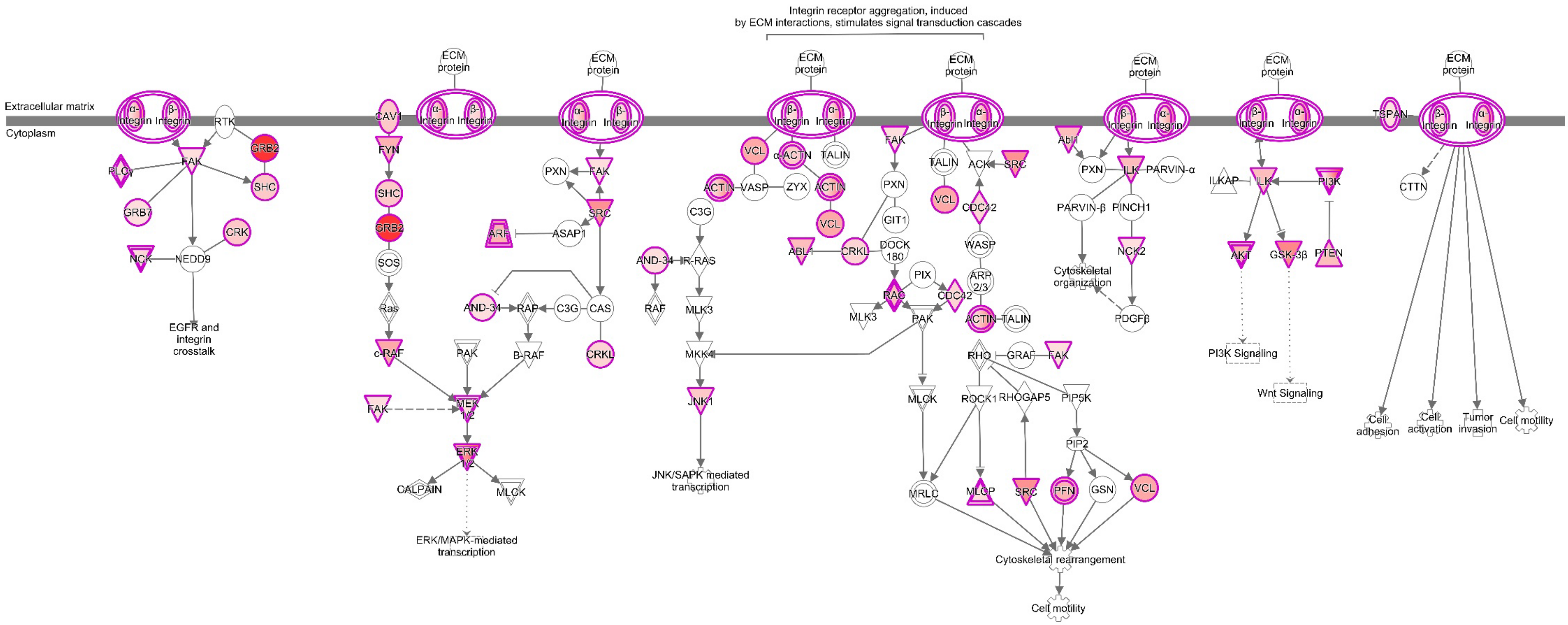

Supplementary Figure 23 Image of Integrin signaling pathway, representing the location and the extent of ALS protein involvement. ALS proteins with higher number of binding partners are marked with increasing color intensity. Obtained from IPA.

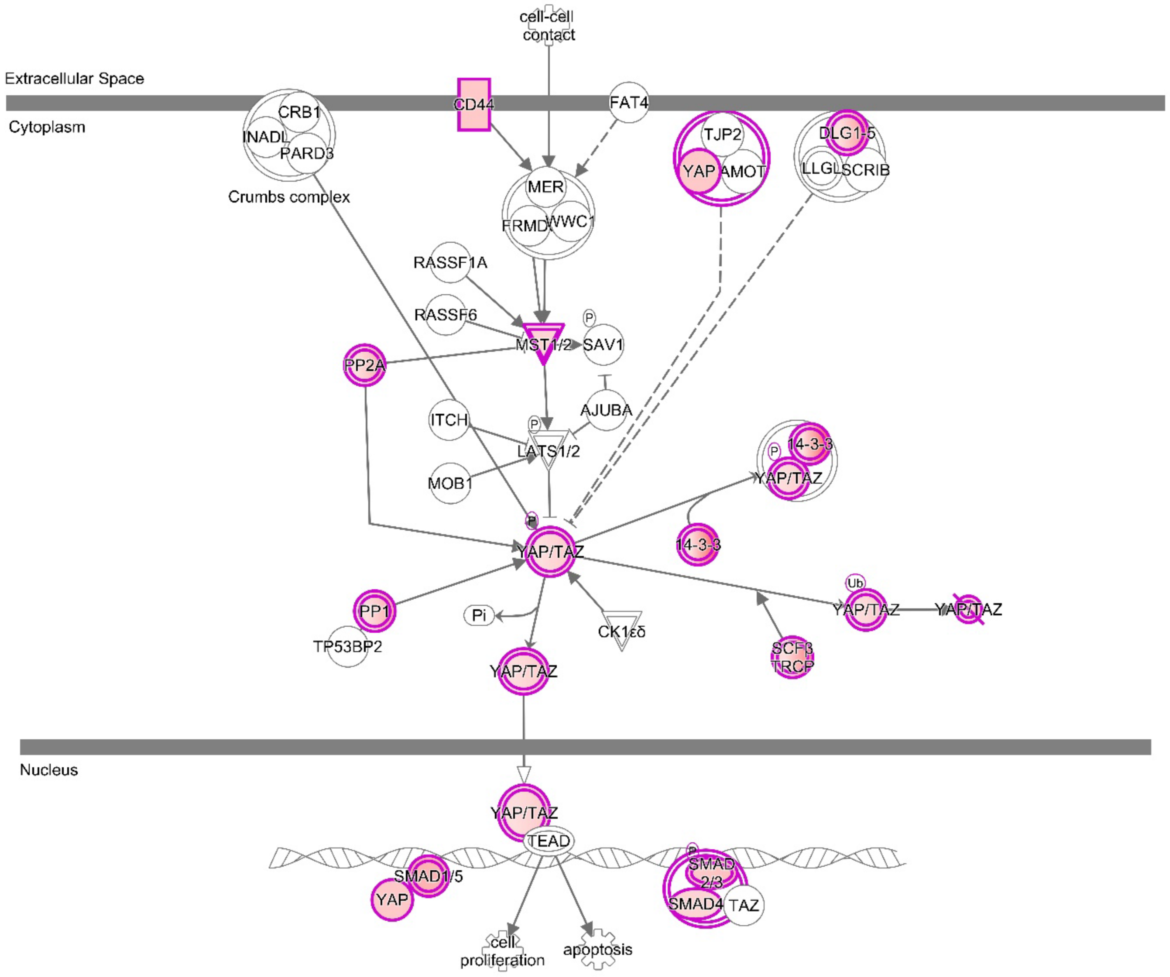

Supplementary Figure 24 Image of HIPPO signaling pathway, representing the location and the extent of ALS protein involvement. ALS proteins with higher number of binding partners are marked with increasing color intensity. Obtained from IPA.

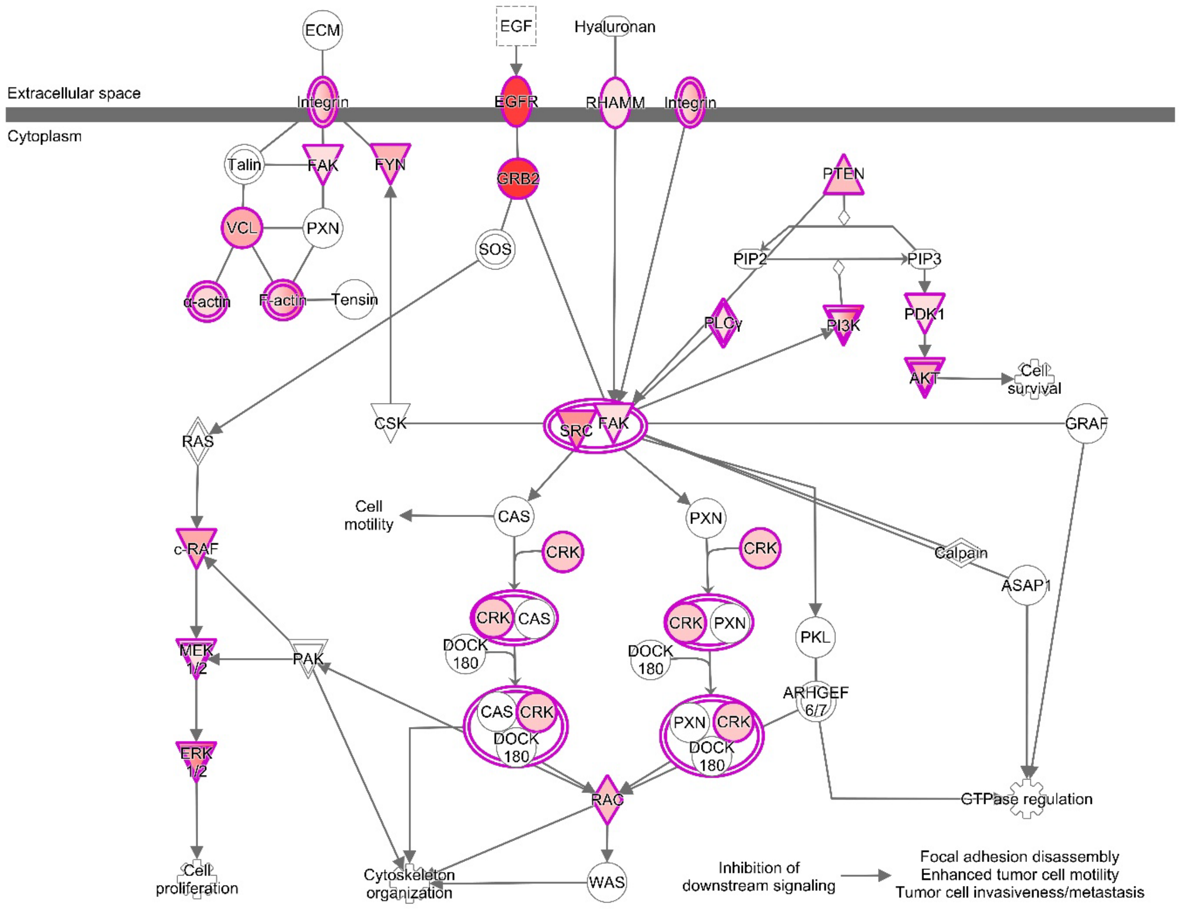

Supplementary Figure 25 Image of FAK signaling pathway, representing the location and the extent of ALS protein involvement. ALS proteins with higher number of binding partners are marked with increasing color intensity. Obtained from IPA.

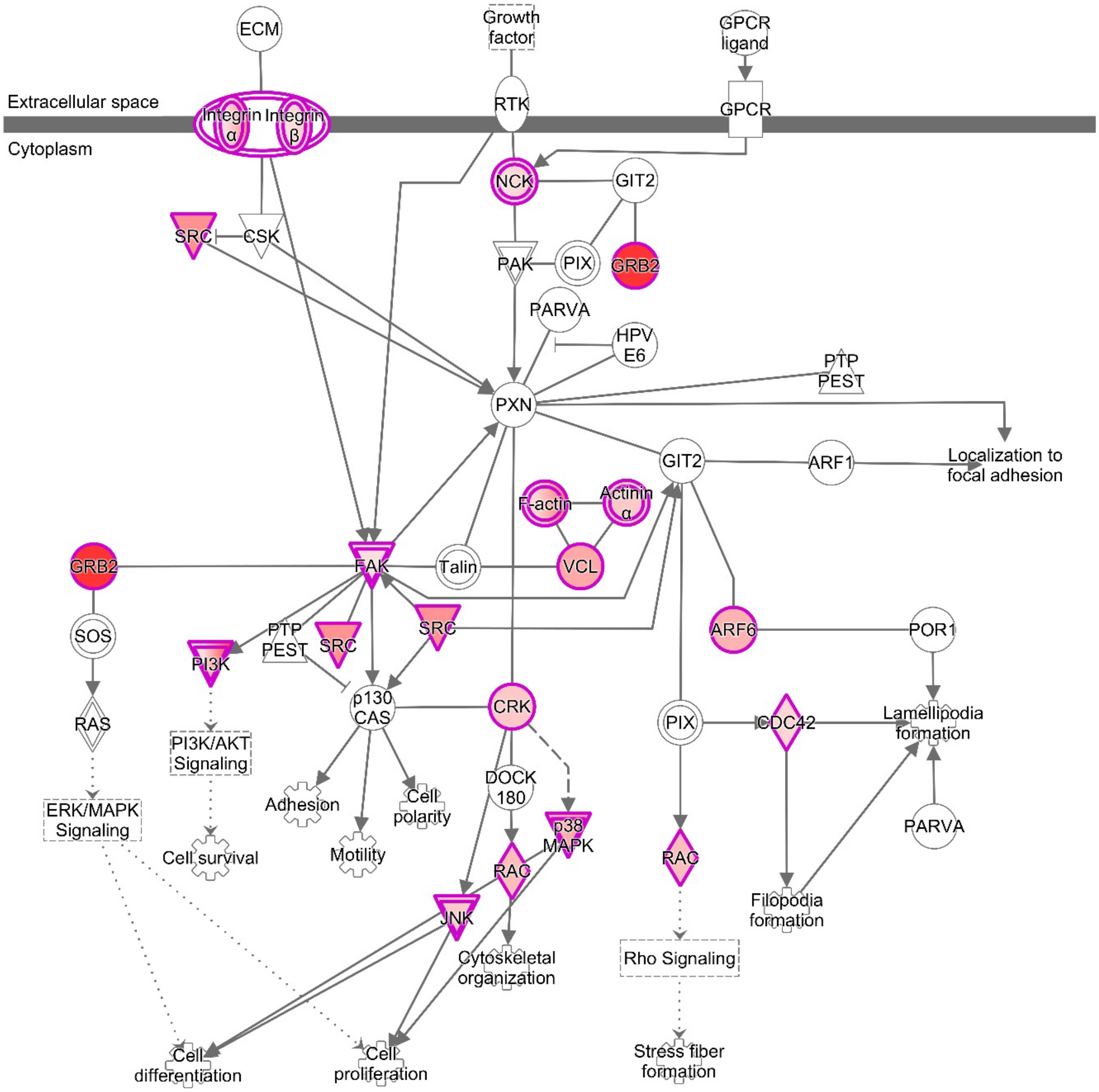

Supplementary Figure 26 Image of Paxillin signaling pathway, representing the location and the extent of ALS protein involvement. ALS proteins with higher number of binding partners are marked with increasing color intensity. Obtained from IPA.

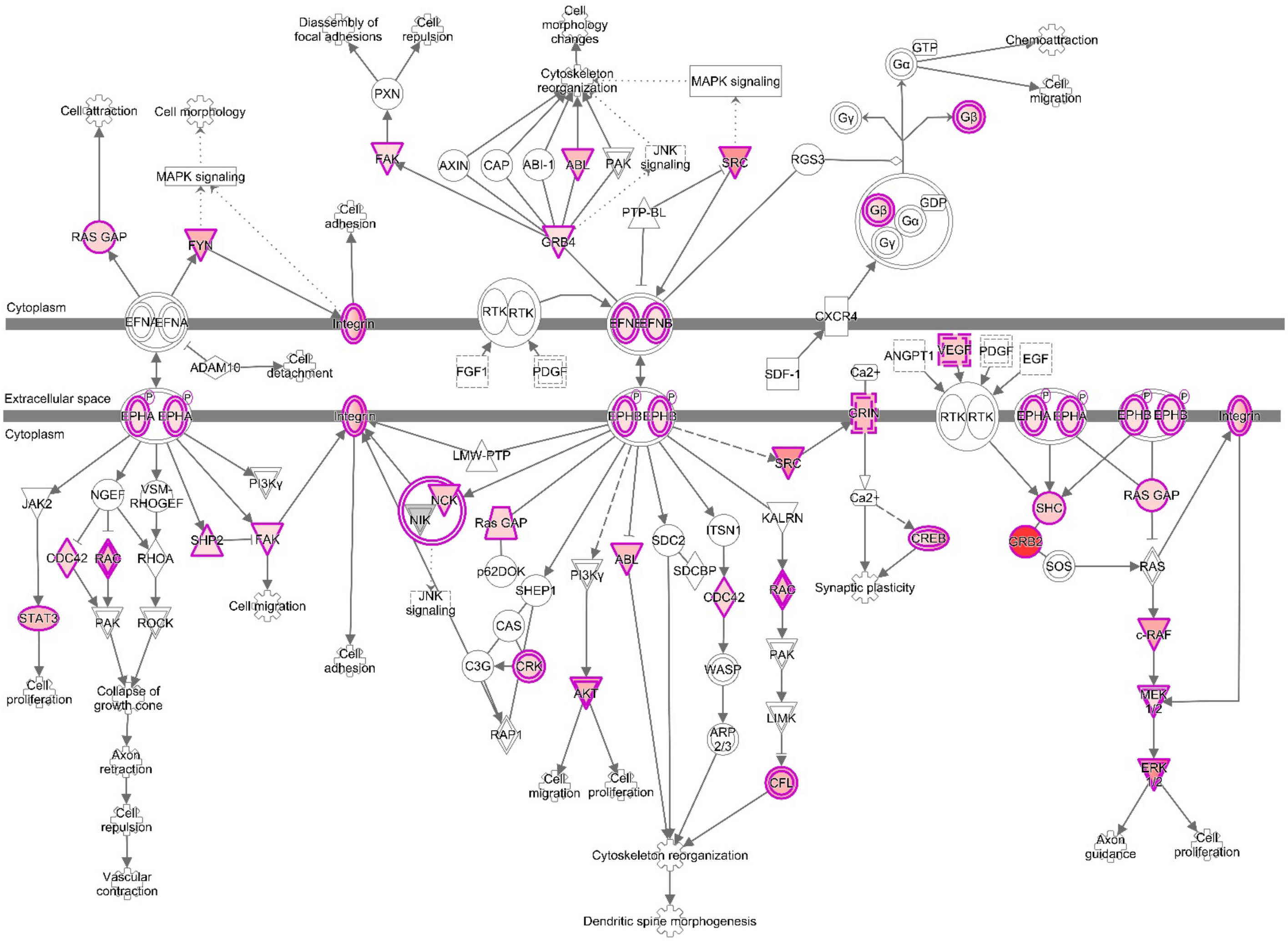

Supplementary Figure 27 Image of Ephrin signaling pathway, representing the location and the extent of ALS protein involvement. ALS proteins with higher number of binding partners are marked with increasing color intensity. Obtained from IPA.

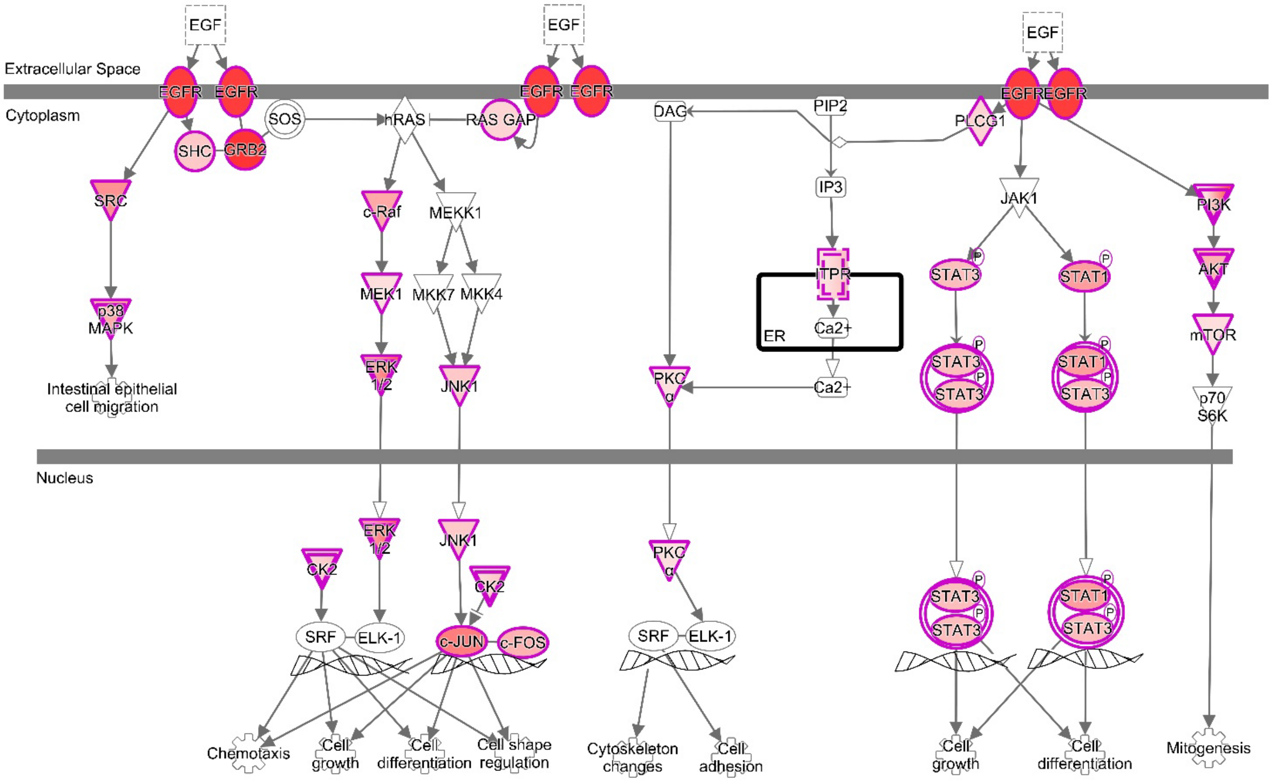

Supplementary Figure 28 Image of EGF signaling pathway, representing the location and the extent of ALS protein involvement. ALS proteins with higher number of binding partners are marked with increasing color intensity. Obtained from IPA.

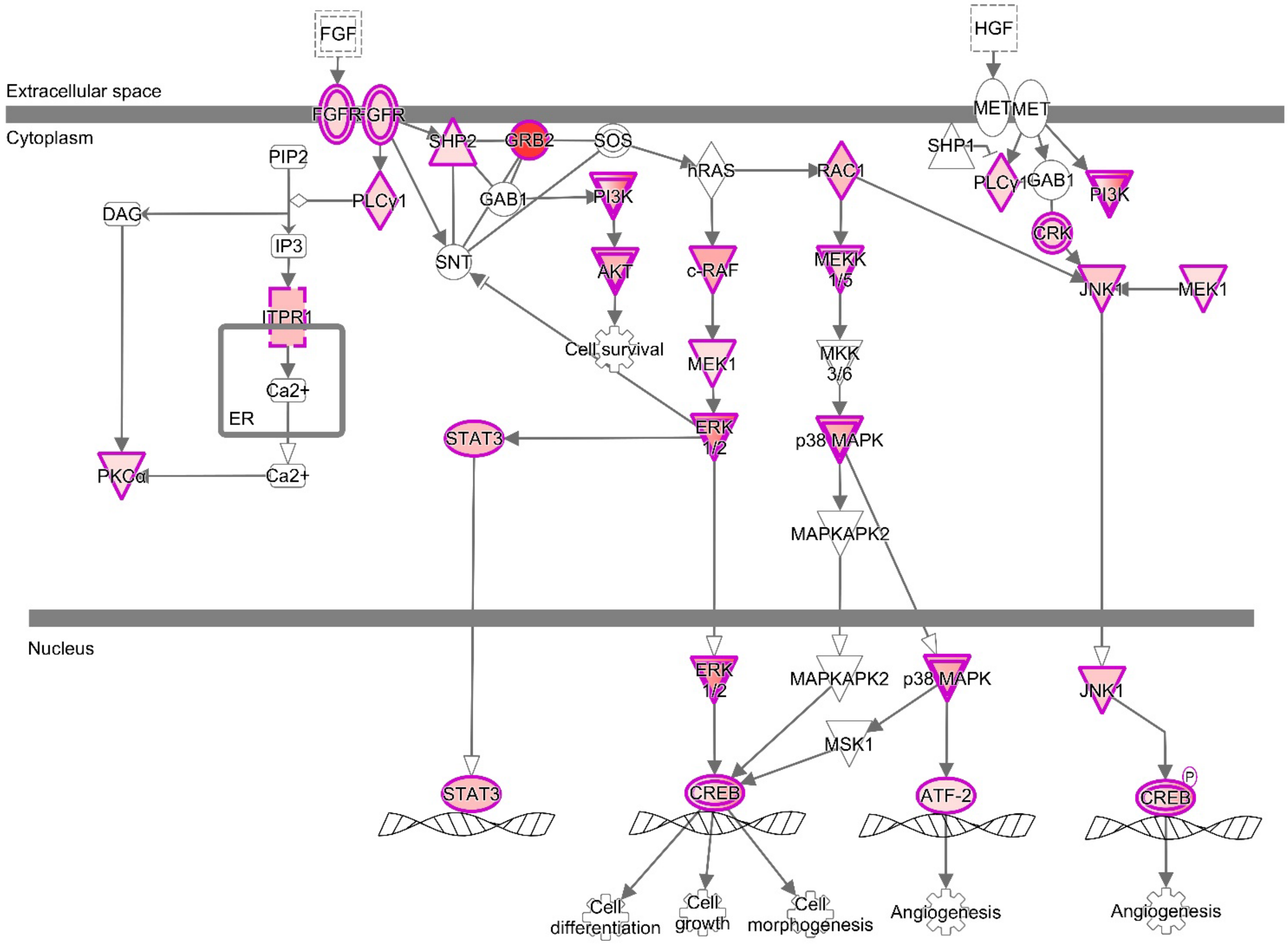

Supplementary Figure 29 <u>Image of FGF signaling pathway,</u> representing the location and the extent of ALS protein involvement. ALS proteins with higher number of binding partners are marked with increasing color intensity. Obtained from IPA.

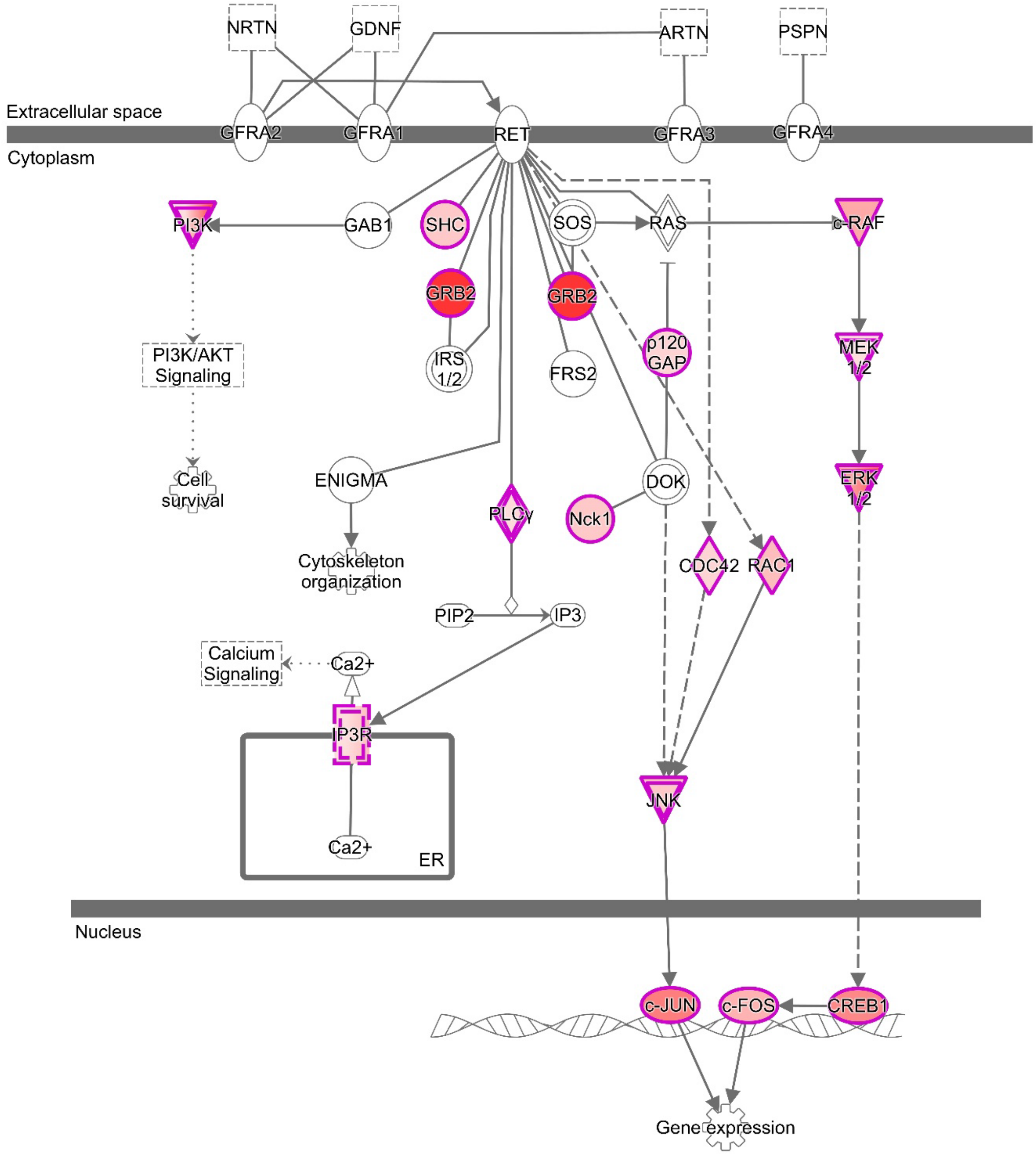

Supplementary Figure 30 Image of GDNF signaling pathway, representing the location and the extent of ALS protein involvement. ALS proteins with higher number of binding partners are marked with increasing color intensity. Obtained from IPA.

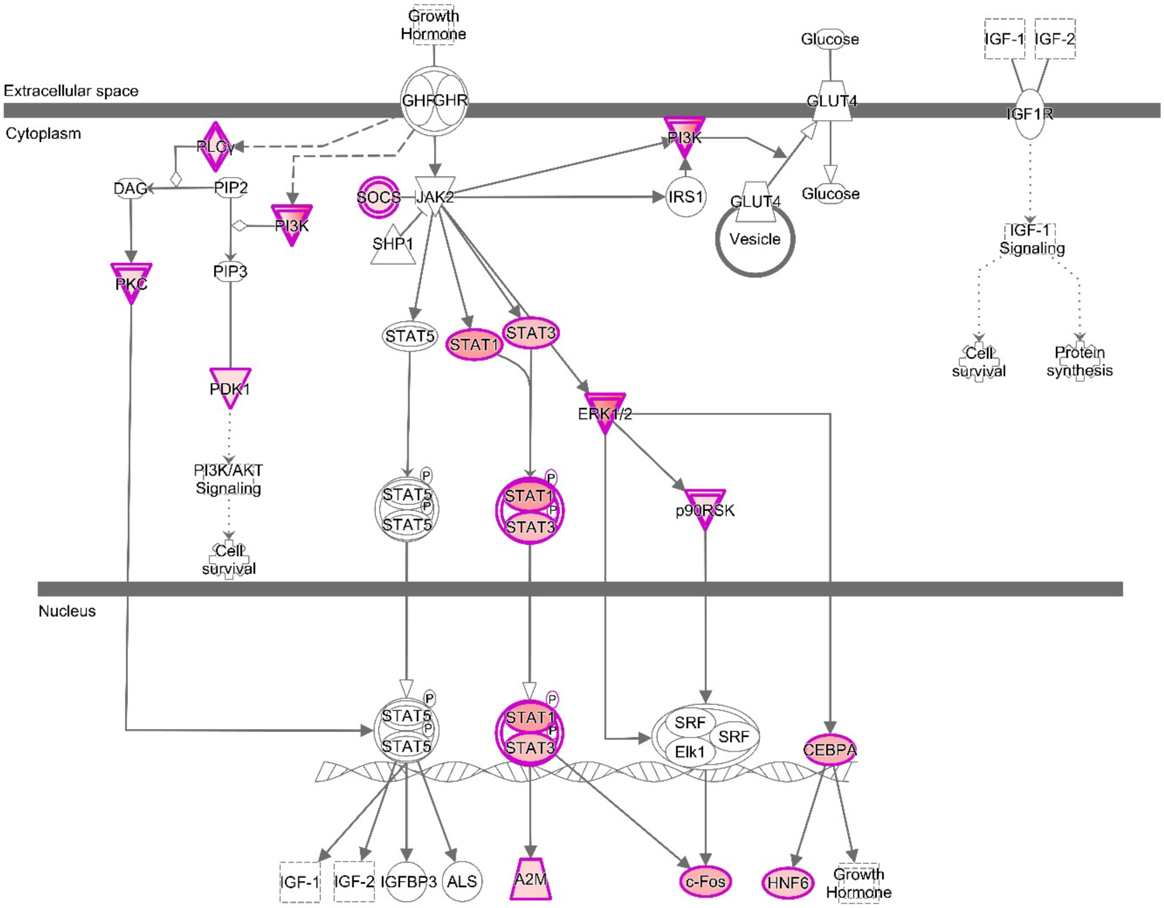

Supplementary Figure 31 Image of Growth hormone signaling pathway, representing the location and the extent of ALS protein involvement. ALS proteins with higher number of binding partners are marked with increasing color intensity. Obtained from IPA.

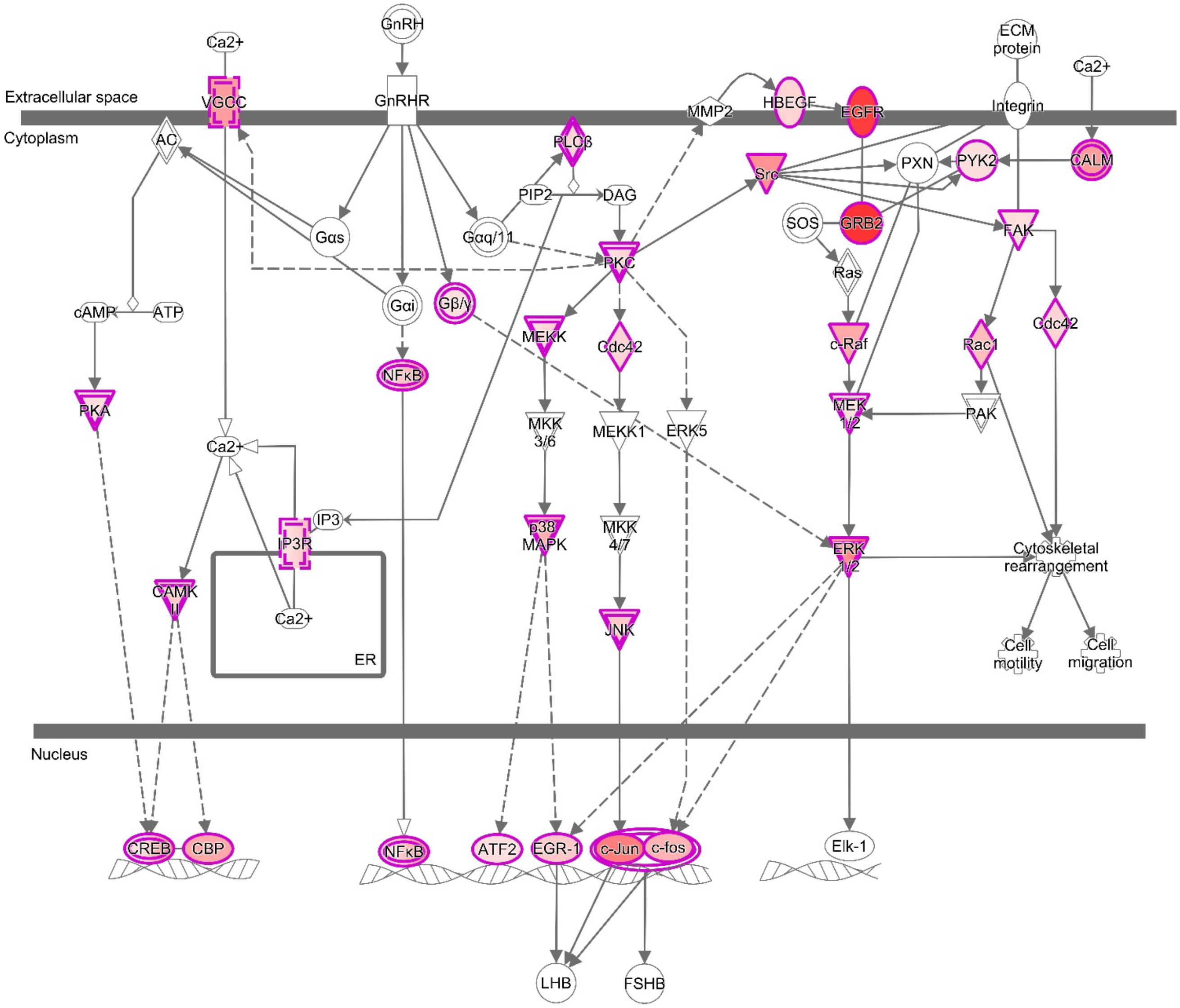

Supplementary Figure 32 Image of GNRH signaling pathway, representing the location and the extent of ALS protein involvement. ALS proteins with higher number of binding partners are marked with increasing color intensity. Obtained from IPA.

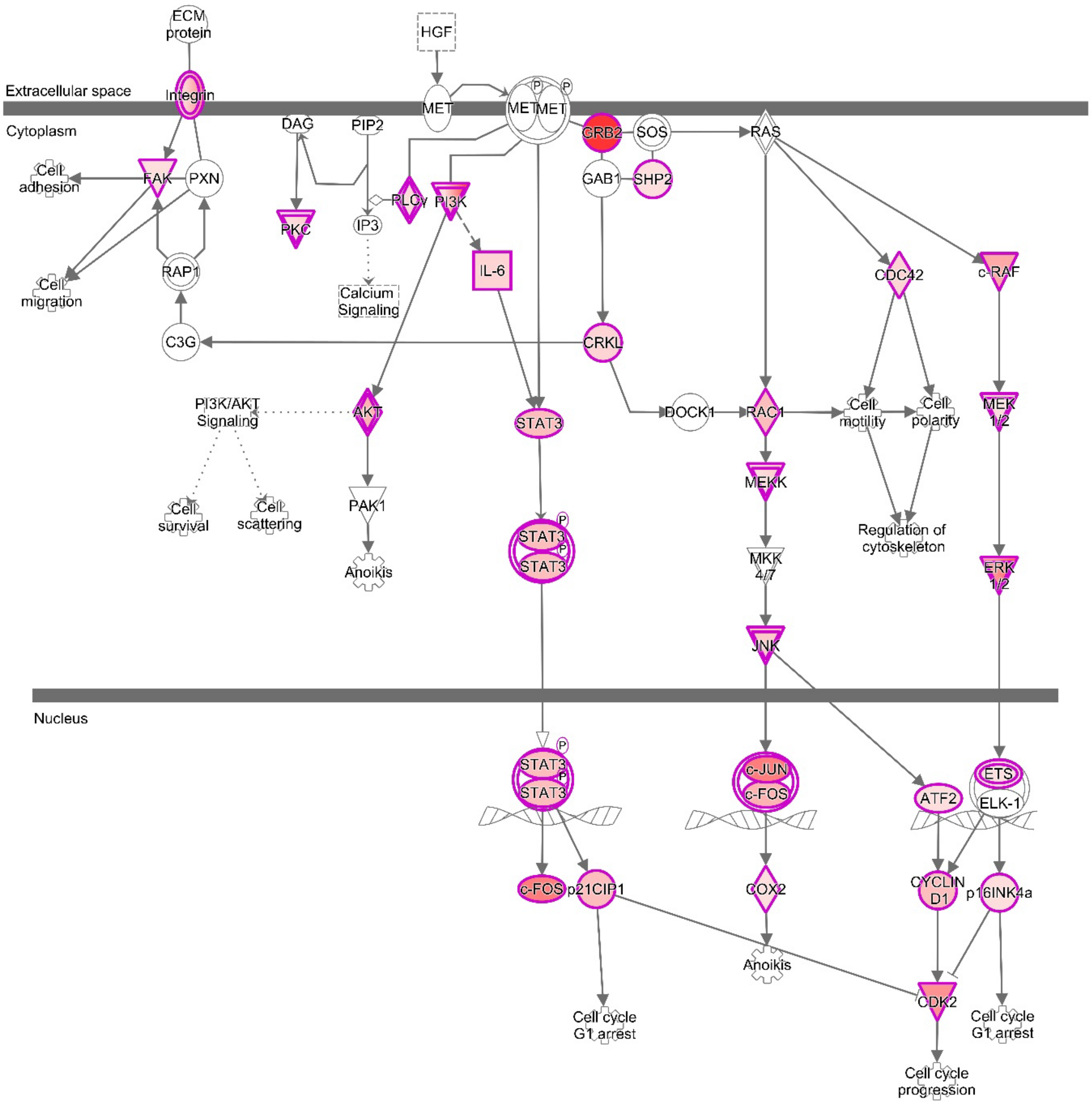

Supplementary Figure 33 Image of HGF signaling pathway, representing the location and the extent of ALS protein involvement. ALS proteins with higher number of binding partners are marked with increasing color intensity. Obtained from IPA.

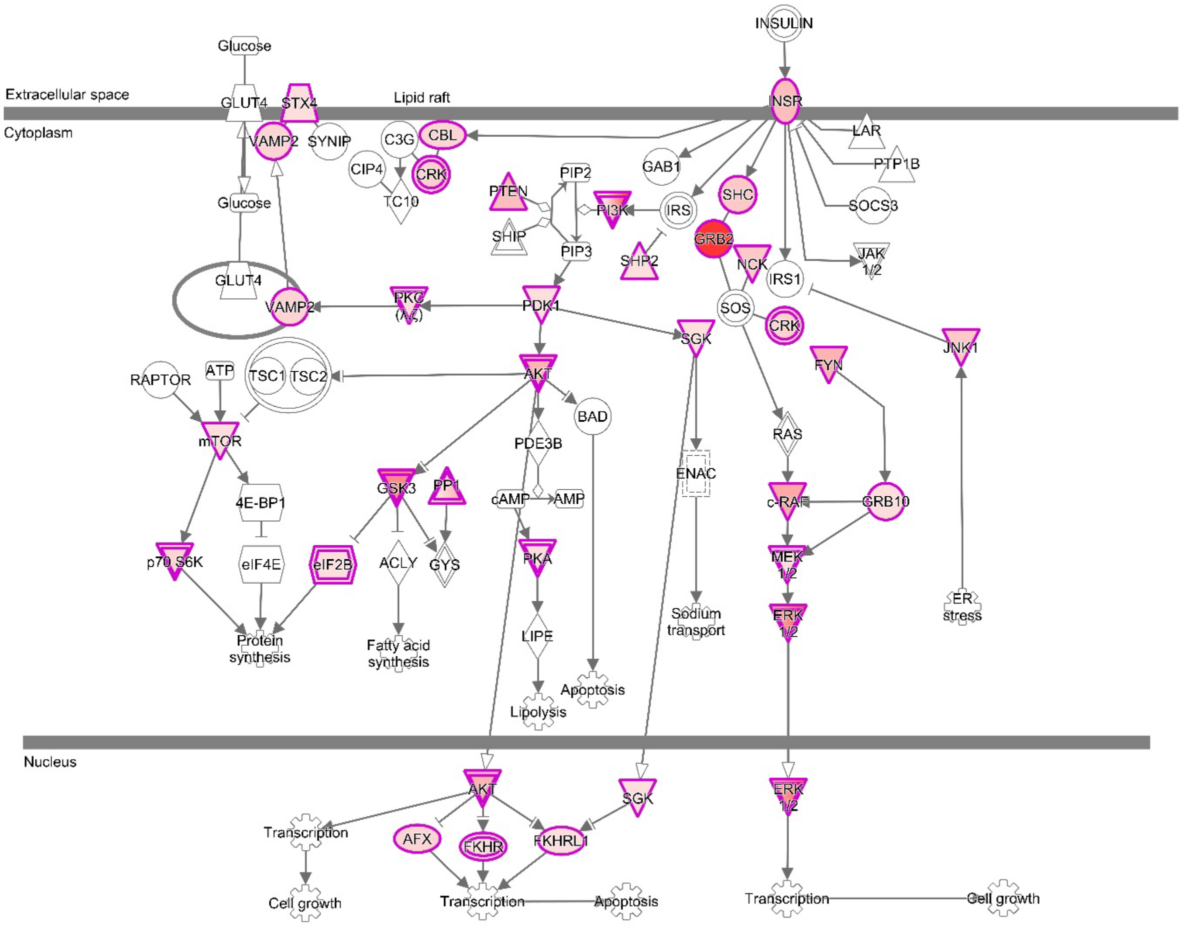

Supplementary Figure 34 Image of Insulin receptor signaling pathway, representing the location and the extent of ALS protein involvement. ALS proteins with higher number of binding partners are marked with increasing color intensity. Obtained from IPA.

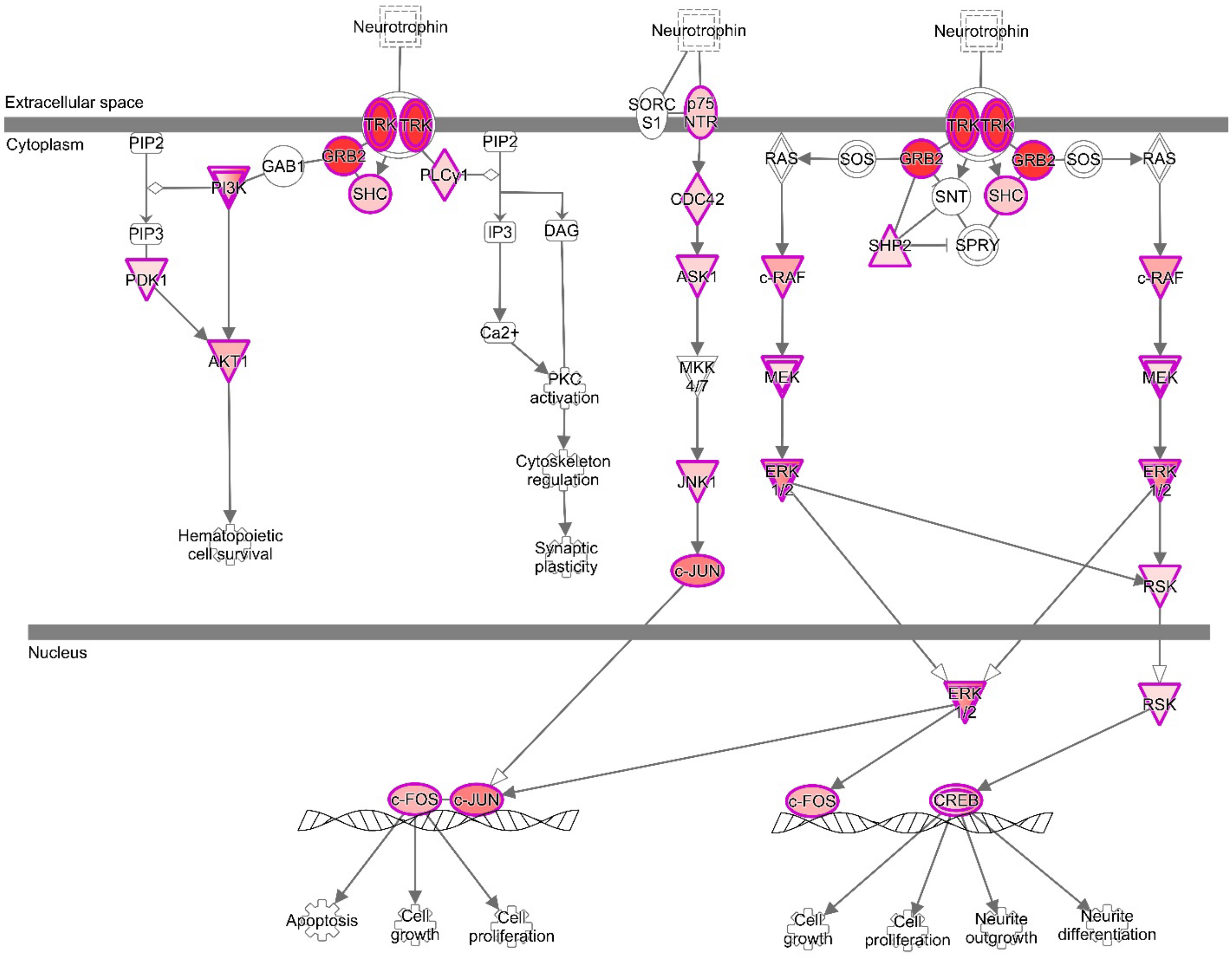

Supplementary Figure 35 Image of Neurotrophin/TRK signaling pathway, representing the location and the extent of ALS protein involvement. ALS proteins with higher number of binding partners are marked with increasing color intensity. Obtained from IPA.

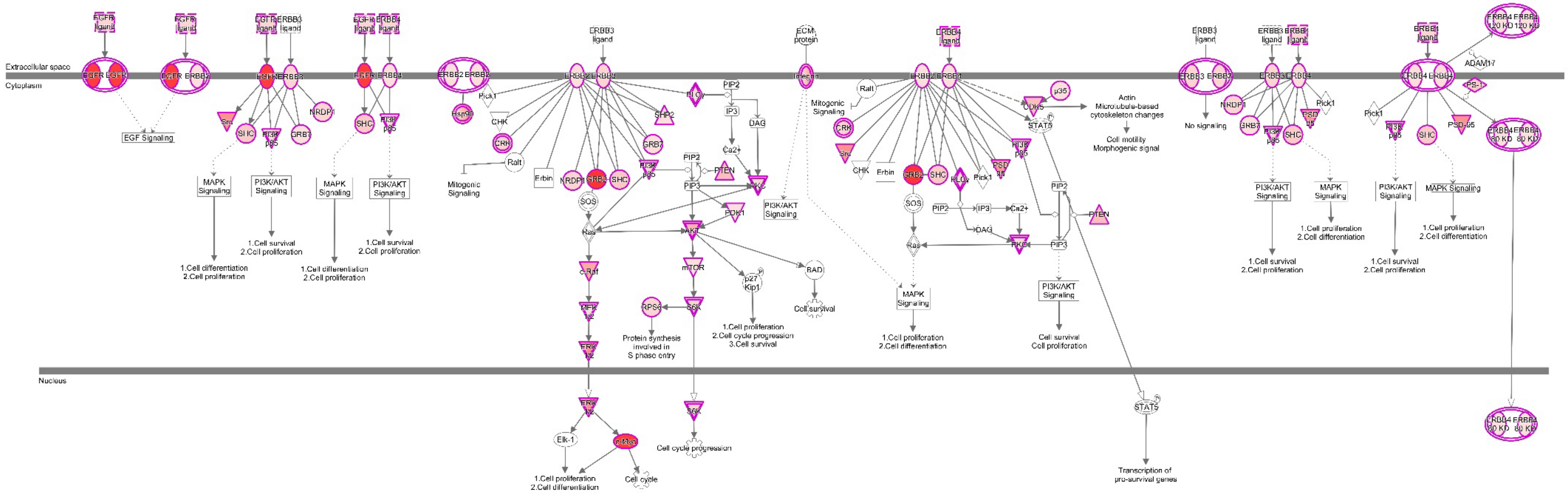

Supplementary Figure 36 Image of Neuroreglin signaling pathway, representing the location and the extent of ALS protein involvement. ALS proteins with higher number of binding partners are marked with increasing color intensity. Obtained from IPA.

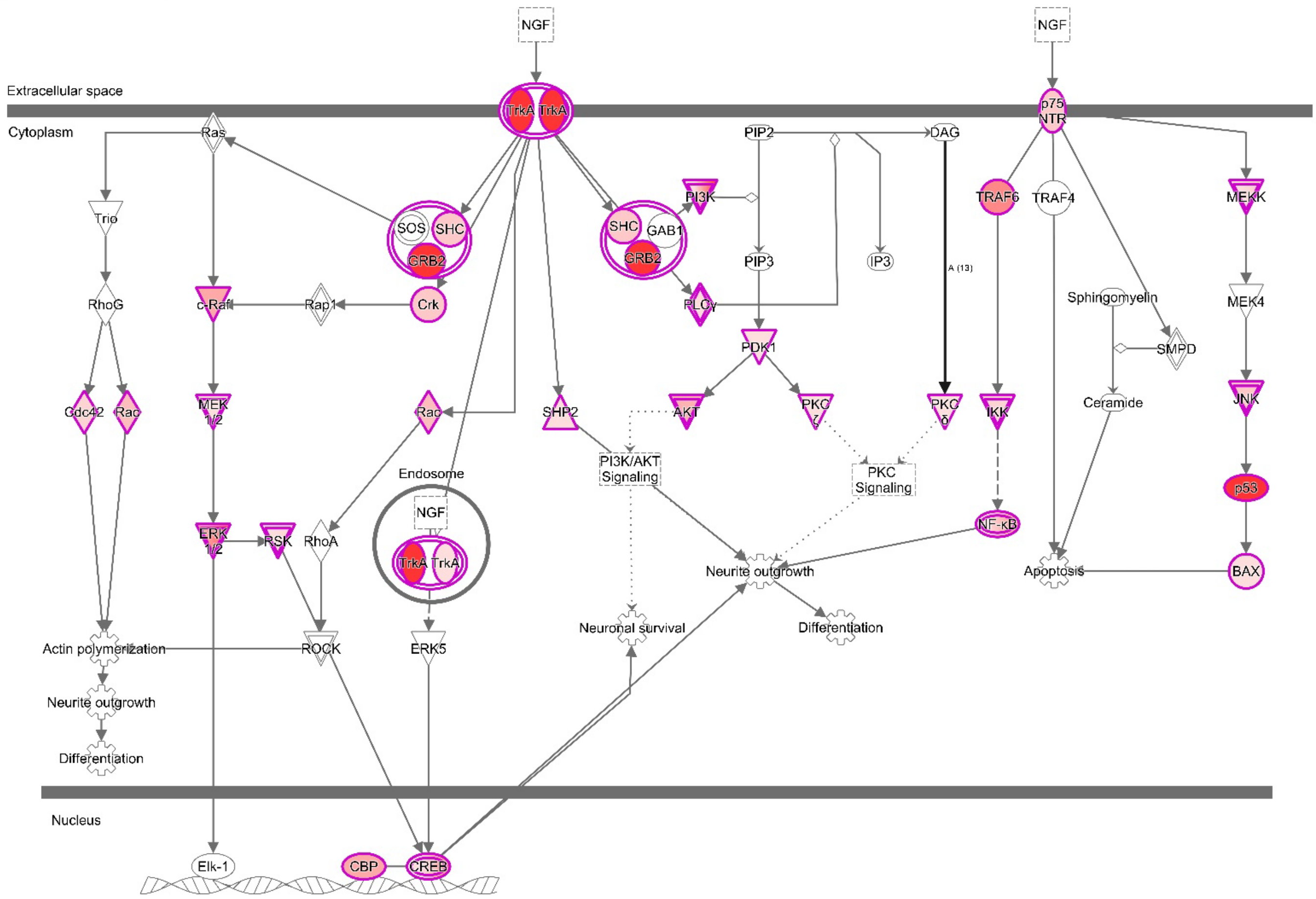

Supplementary Figure 37 Image of NGF signaling pathway, representing the location and the extent of ALS protein involvement. ALS proteins with higher number of binding partners are marked with increasing color intensity. Obtained from IPA.

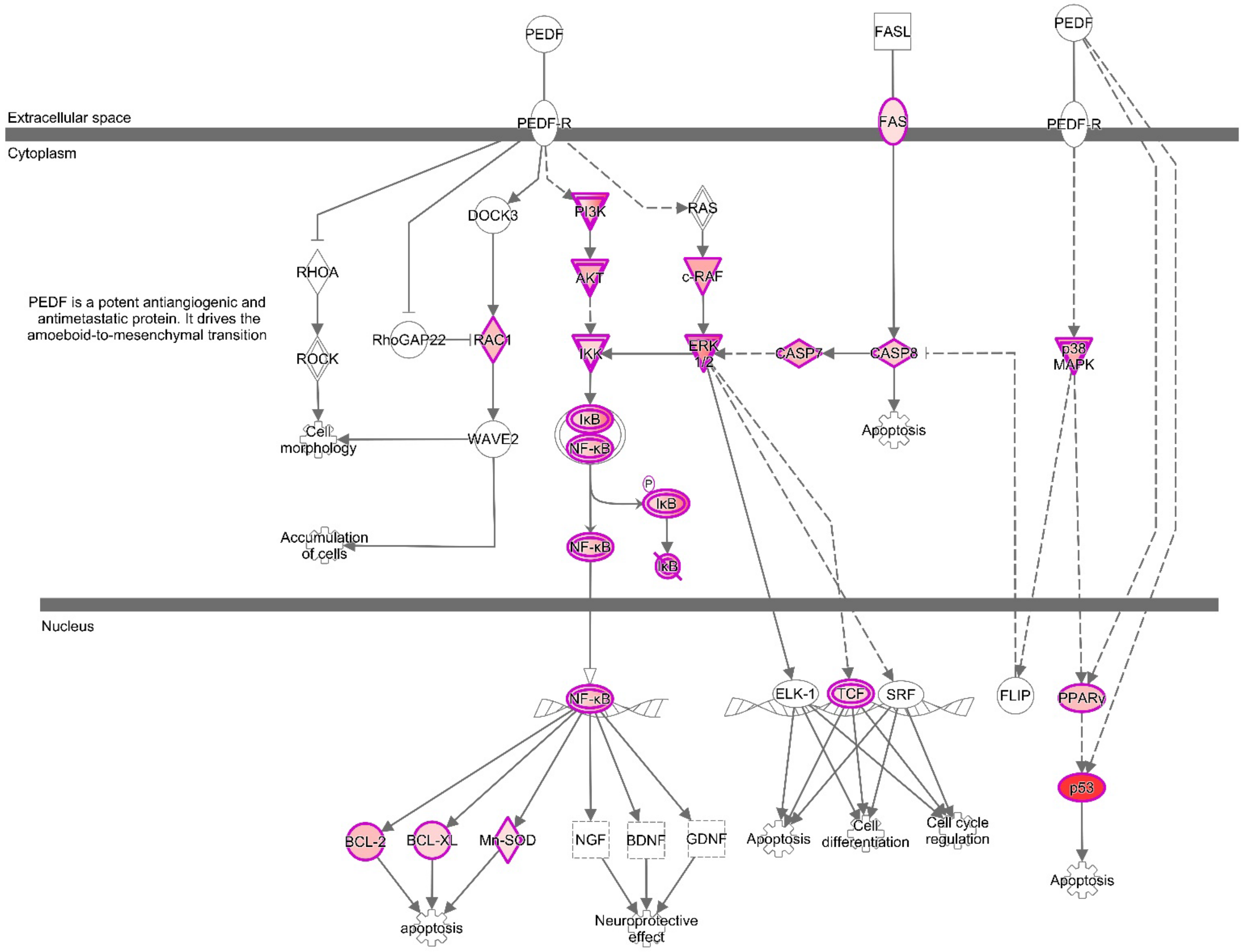

Supplementary Figure 38 Image of PEDF signaling pathway, representing the location and the extent of ALS protein involvement. ALS proteins with higher number of binding partners are marked with increasing color intensity. Obtained from IPA.

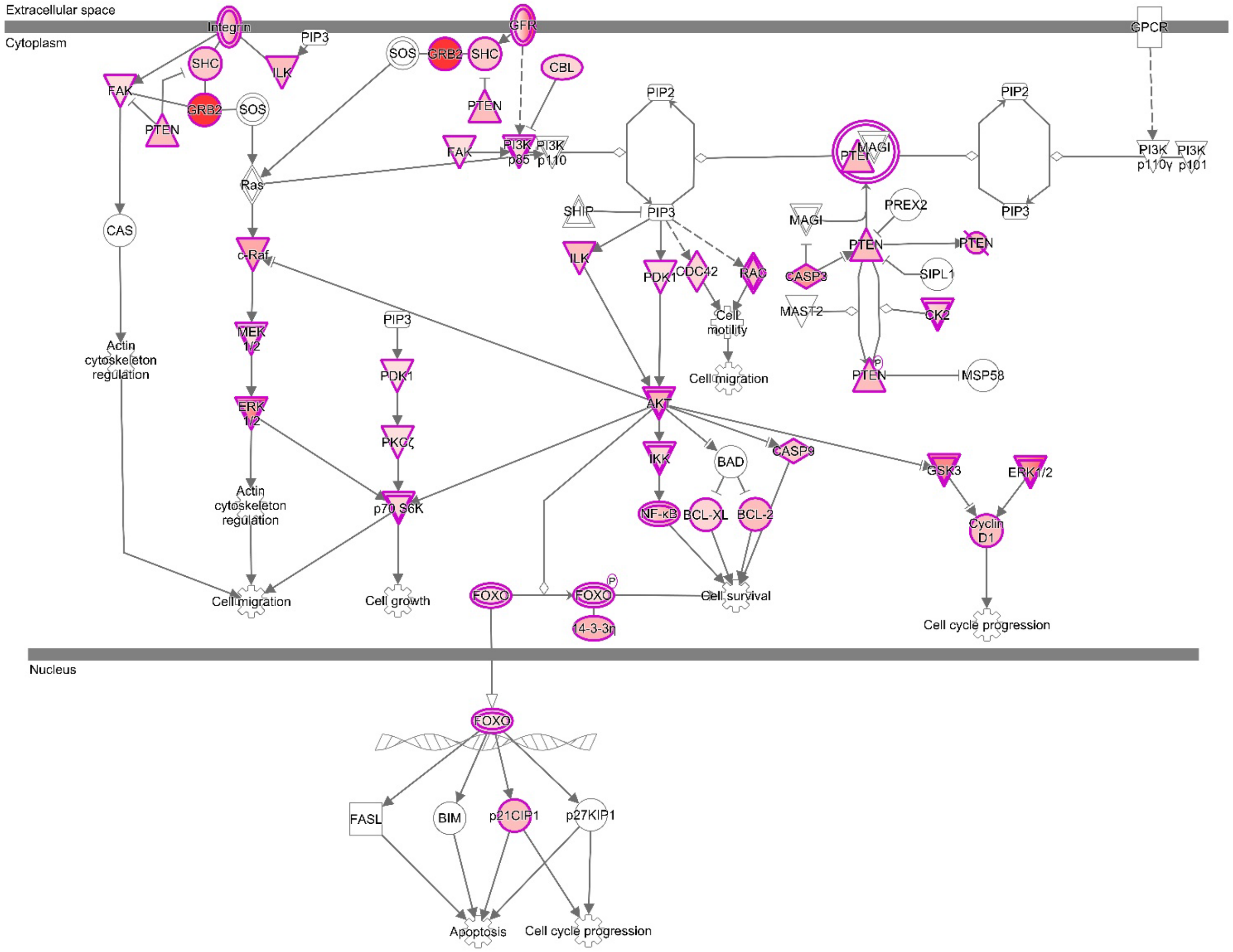

Supplementary Figure 39 Image of PTEN signaling pathway, representing the location and the extent of ALS protein involvement. ALS proteins with higher number of binding partners are marked with increasing color intensity. Obtained from IPA.

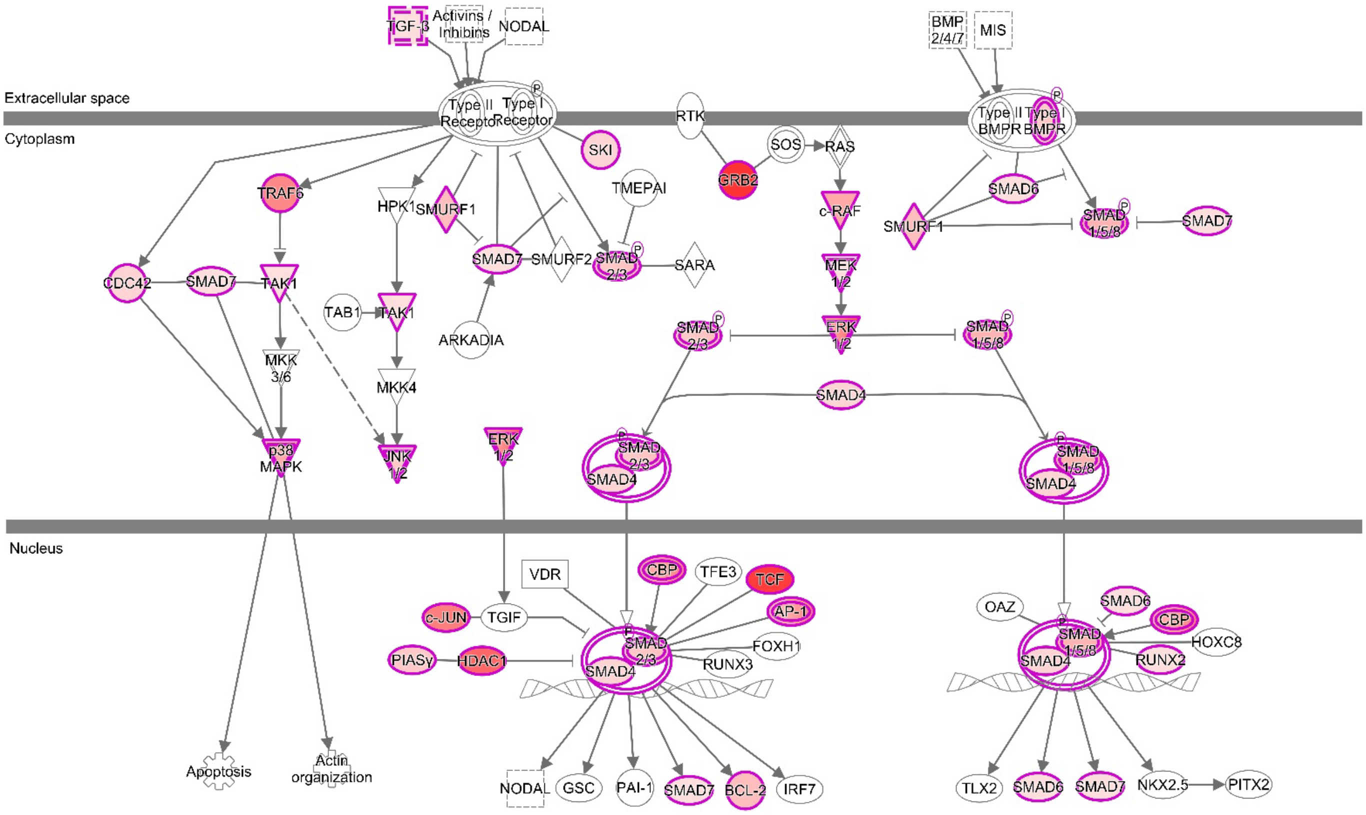

Supplementary Figure 40 Image of TGF-b signaling pathway, representing the location and the extent of ALS protein involvement. ALS proteins with higher number of binding partners are marked with increasing color intensity. Obtained from IPA.

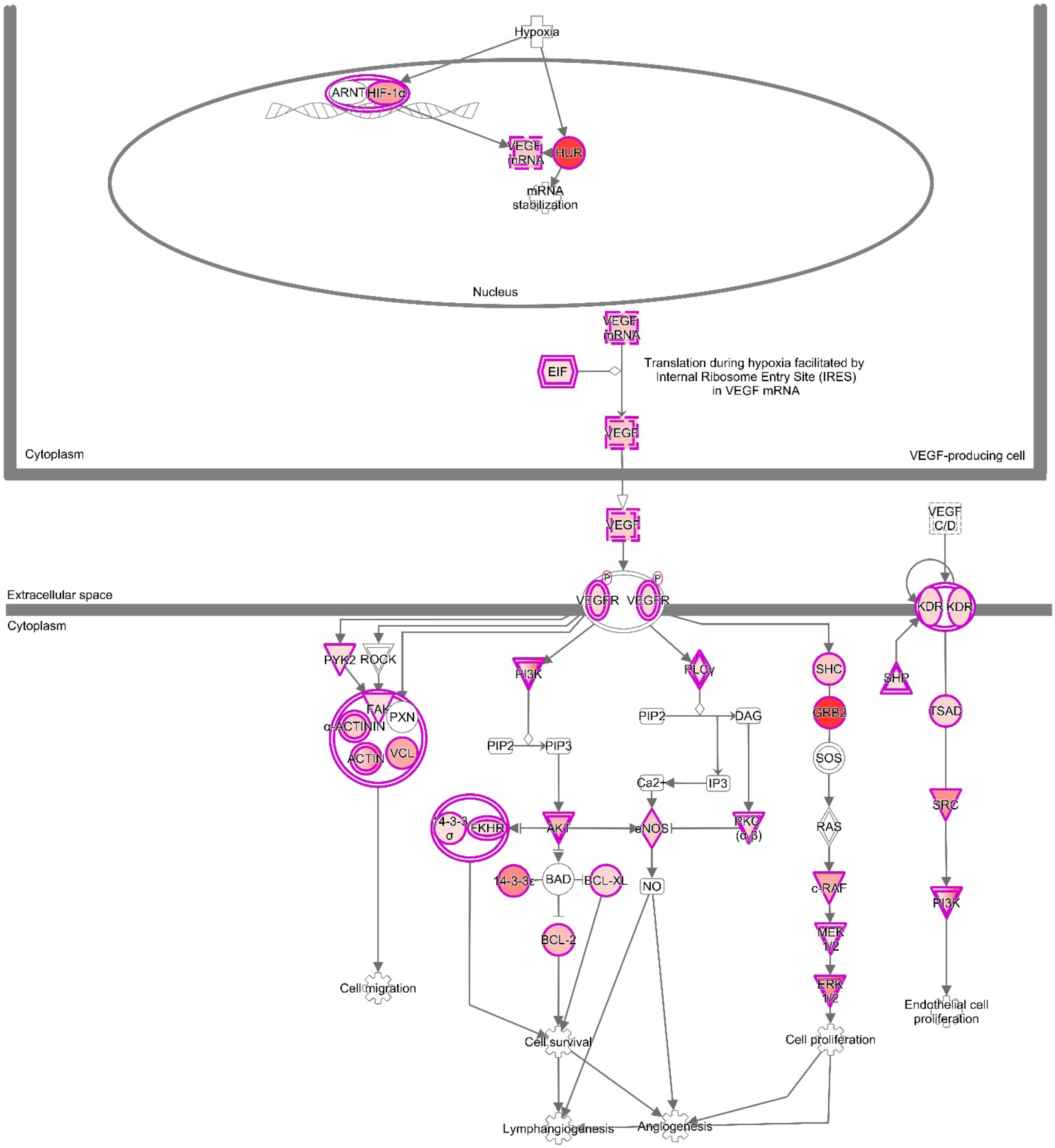

Supplementary Figure 41 Image of VEGF signaling pathway, representing the location and the extent of ALS protein involvement. ALS proteins with higher number of binding partners are marked with increasing color intensity. Obtained from IPA.

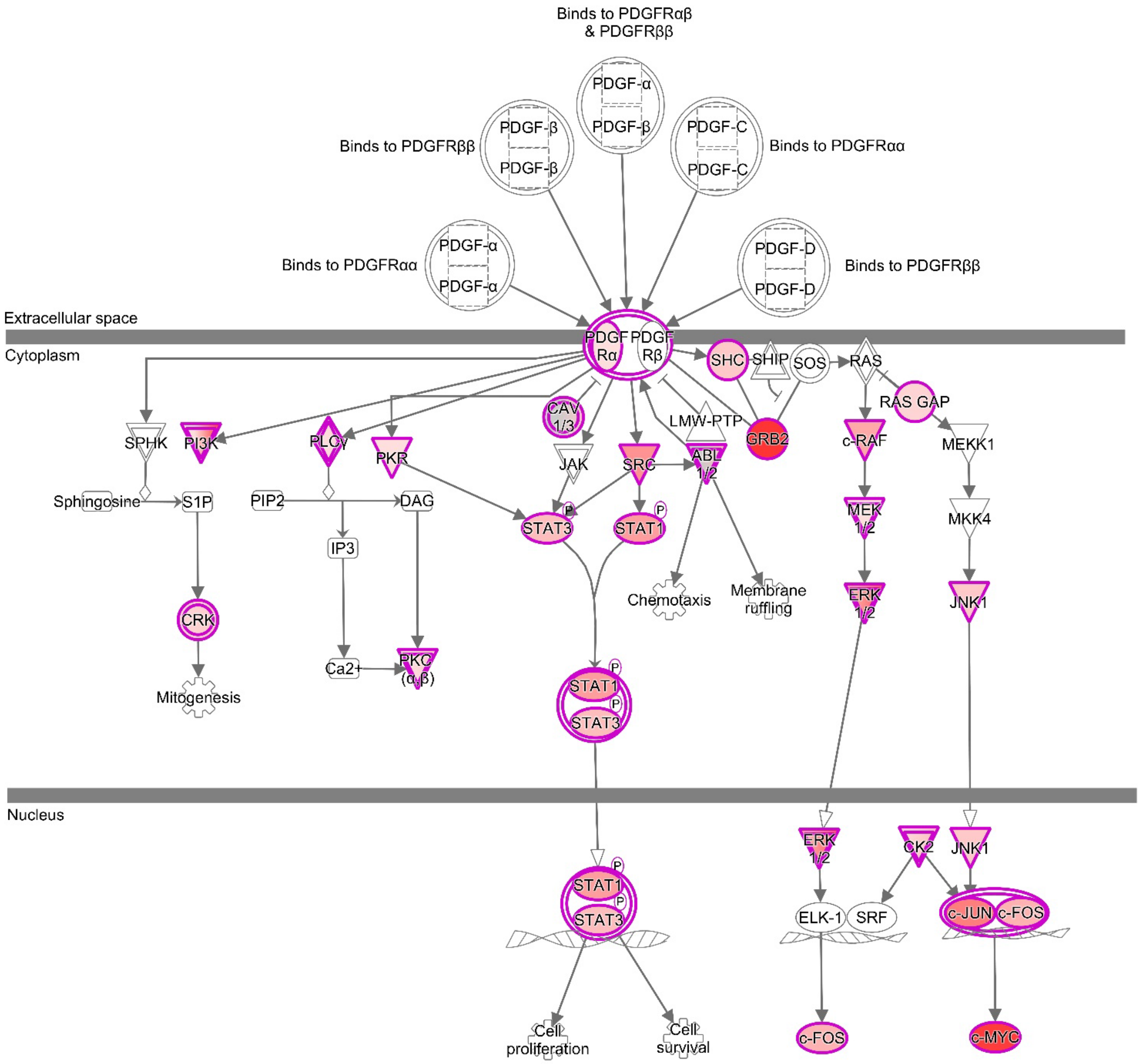

Supplementary Figure 42 Image of PDGF signaling pathway, representing the location and the extent of ALS protein involvement. ALS proteins with higher number of binding partners are marked with increasing color intensity. Obtained from IPA.
