# Supplementary material for: Protein-protein interactions reveal key canonical pathways, upstream regulators, interactome domains, and novel targets in ALS
Source: Sci Rep. 2018 Oct 3;8:14732. doi: 10.1038/s41598-018-32902-4 (PMC6170493; doi:10.1038/s41598-018-32902-4)
Supplement: Supplementary file 2 — All Supplementary Tables [file 41598_2018_32902_MOESM2_ESM.pdf]

Supplementary Information-Tables

Title: Protein-protein interactions reveal key canonical pathways, upstream regulators, interactome domains, and novel targets in ALS

Manuscript number: SREP-18-22830A

Authors: Ina Dervishi[1], Oge Gozutok[1], Kevin Murnan[1], Mukesh Gautam[1], Daniel Heller[1], Eileen Bigio[2,3], and P. Hande Ozdinler[1,2,4,5]

[1]Department of Neurology, Northwestern University, Feinberg School of Medicine, [2]Mesulam Cognitive Neurology and Alzheimer Disease Center, [3]Department of Pathology, Northwestern University, Chicago, IL 60611, USA, [4]Les Turner ALS Center, [5]Robert H. Lurie Comprehensive Cancer Center, Northwestern University, Feinberg School of Medicine, Chicago, IL, 60611

Supplementary Table 1

Causative and associated genes related to ALS and their supporting references.

Causative Genes:

ALS2[1], ALS3[2], ALS7[3], ANG[4], ANXA11, ATXN2[5], CFAP410[6], C9orf72[7,8], CHCHD10[9], CHMP2B[10], DAO[11], DCTN1[12], ELP3[13], ERBB4[14], Erlin1[15], FIG4[16], FUS[17], HNRNPA1[18], LMNB1[19], MATR3[19], NEFH[20], NEK1[21,22], OPTN[23], PFN1[24], PRPH[25,26], SETX[27], SIGMAR1[28], SOD1[29], SPAST[30], SPG11[31], SQSTM1[32], TAF15[33], TARDBP[34], TIA1[35,36], TUBA4A[37], UBQLN2[38], UNC13A[39], VAPB[40], VCP[41].

Associated and Disease Modifier Genes:

AGT[42], ALAD[43], APEX1[44], APOE[45],AR[46,47], ARHGEF28[48], ATRN[22], ATXN1[49], B4GALT6[50], BCL6[51], BCL11B[52], BIRC6[22], C1orf27[53], C1QTNF7[54],CCNF[55], CCS[56], CDH13[51], CDH22[51], CHGB[57],CNTF[58], CNTN4[59], CNTN6[51], CREB3L2[22], CRIM1[51], CRYM[60], CSNK1G3[61], CST3[62], CX3CR1[63],CYP2D6[64], DCC[22], DIAPH3[51], DISC1[65], DOC2B[51], DPP6[66],DYNC1H1[67], EFEMP1[61], EPHA4[68], EWSR1[69], FEZF2[51], FGGY[66], GARS[70], GLE1[71], GRB14[51], GRN[72], HEXA[73], HFE[74], HNRNPA2B1[75], ITPR2[76], KCNN1[6], KDR[77], KIF5A[78], KIFAP3[79], LIF[80], LIPC[81], LOX[82], LUM[51], MAOB[83,84], MAPT[85], MOBP[6], MT-ND2[86], NAIP[87], NETO1[51], NIPA1[88], NT5C1A[89], OGG1[90], OMA1[51], PARK7[91], PCP4[51], PEAK1[22], PLEKHG5[92], POLDIP2[6], PON1[93], PON2[94], PON3[94], PSEN1[95], PVR[96], RAMP3[51], RBMS1[61], RNASE2[97], RNF19A[98], SARM1[6], SCFD1[6], SCN7A[99],SELL[61], SEMA6A[61], SLC1A2[100], SLC39A11[101], SMN1[102], SMN2[103], SNCG[104], SOD2[105], SOX5[106], SPG7[107], SS18L1[108], STX12[22], SUSD1[109], SYNE[92], SYT9[51], TBK1[110], TRPM7[111], TUBA4A[37], VDR[43], VEGFA[112], VPS54[113], WDR49[22],ZFP64[114], ZNF746[115], ZNF512B[116], ZSCAN5B[22].

References for the main genes:

1 Kress, J. A. *et al.* Novel mutation in the ALS2 gene in juvenile amyotrophic lateral sclerosis. *Ann Neurol* **58**, 800-803, doi:10.1002/ana.20665 (2005).

2 Oda, M., Izumi, Y. & Kaji, R. [Gene mutations in familial amyotrophic lateral sclerosis]. *Brain Nerve* **63**, 165-170 (2011).

3 Sapp, P. C. *et al.* Identification of two novel loci for dominantly inherited familial amyotrophic lateral sclerosis. *Am J Hum Genet* **73**, 397-403, doi:10.1086/377158 (2003).

4 Greenway, M. J. *et al.* ANG mutations segregate with familial and 'sporadic' amyotrophic lateral sclerosis. *Nat Genet* **38**, 411-413, doi:10.1038/ng1742 (2006).

5 Elden, A. C. *et al.* Ataxin-2 intermediate-length polyglutamine expansions are associated with increased risk for ALS. *Nature* **466**, 1069-1075, doi:10.1038/nature09320 (2010).

6 van Rheenen, W. *et al.* Genome-wide association analyses identify new risk variants and the genetic architecture of amyotrophic lateral sclerosis. *Nat Genet* **48**, 1043-1048, doi:10.1038/ng.3622 (2016).

7 DeJesus-Hernandez, M. *et al.* Expanded GGGGCC hexanucleotide repeat in noncoding region of C9ORF72 causes chromosome 9p-linked FTD and ALS. *Neuron* **72**, 245-256, doi:10.1016/j.neuron.2011.09.011 (2011).

8 Renton, A. E. *et al.* A hexanucleotide repeat expansion in C9ORF72 is the cause of chromosome 9p21-linked ALS-FTD. *Neuron* **72**, 257-268, doi:10.1016/j.neuron.2011.09.010 (2011).

9 Bannwarth, S. *et al.* A mitochondrial origin for frontotemporal dementia and amyotrophic lateral sclerosis through CHCHD10 involvement. *Brain* **137**, 2329-2345, doi:10.1093/brain/awu138 (2014).

10 Parkinson, N. *et al.* ALS phenotypes with mutations in CHMP2B (charged multivesicular body protein 2B). *Neurology* **67**, 1074-1077, doi:10.1212/01.wnl.0000231510.89311.8b (2006).
11 Mitchell, J. *et al.* Familial amyotrophic lateral sclerosis is associated with a mutation in D-amino acid oxidase. *Proc Natl Acad Sci U S A* **107**, 7556-7561, doi:10.1073/pnas.0914128107 (2010).
12 Munch, C. *et al.* Point mutations of the p150 subunit of dynactin (DCTN1) gene in ALS. *Neurology* **63**, 724-726 (2004).
13 Simpson, C. L. *et al.* Variants of the elongator protein 3 (ELP3) gene are associated with motor neuron degeneration. *Hum Mol Genet* **18**, 472-481, doi:10.1093/hmg/ddn375 (2009).
14 Takahashi, Y. *et al.* ERBB4 mutations that disrupt the neuregulin-ErbB4 pathway cause amyotrophic lateral sclerosis type 19. *Am J Hum Genet* **93**, 900-905, doi:10.1016/j.ajhg.2013.09.008 (2013).
15 Tunca, C. *et al.* ERLIN1 mutations cause teenage-onset slowly progressive ALS in a large Turkish pedigree. *Eur J Hum Genet*, doi:10.1038/s41431-018-0107-5 (2018).
16 Chow, C. Y. *et al.* Deleterious variants of FIG4, a phosphoinositide phosphatase, in patients with ALS. *Am J Hum Genet* **84**, 85-88, doi:10.1016/j.ajhg.2008.12.010 (2009).
17 Kwiatkowski, T. J., Jr. *et al.* Mutations in the FUS/TLS gene on chromosome 16 cause familial amyotrophic lateral sclerosis. *Science* **323**, 1205-1208, doi:10.1126/science.1166066 (2009).
18 Krebs, B. B. & De Mesquita, J. F. Amyotrophic Lateral Sclerosis Type 20 - In Silico Analysis and Molecular Dynamics Simulation of hnRNPA1. *PLoS One* **11**, e0158939, doi:10.1371/journal.pone.0158939 (2016).
19 Johnson, J. O. *et al.* Mutations in the Matrin 3 gene cause familial amyotrophic lateral sclerosis. *Nat Neurosci* **17**, 664-666, doi:10.1038/nn.3688 (2014).
20 Figlewicz, D. A. *et al.* Variants of the heavy neurofilament subunit are associated with the development of amyotrophic lateral sclerosis. *Hum Mol Genet* **3**, 1757-1761 (1994).
21 Brenner, D. *et al.* NEK1 mutations in familial amyotrophic lateral sclerosis. *Brain* **139**, e28, doi:10.1093/brain/aww033 (2016).
22 Kenna, K. P. *et al.* NEK1 variants confer susceptibility to amyotrophic lateral sclerosis. *Nat Genet* **48**, 1037-1042, doi:10.1038/ng.3626 (2016).
23 Maruyama, H. *et al.* Mutations of optineurin in amyotrophic lateral sclerosis. *Nature* **465**, 223-226, doi:10.1038/nature08971 (2010).
24 Wu, C. H. *et al.* Mutations in the profilin 1 gene cause familial amyotrophic lateral sclerosis. *Nature* **488**, 499-503, doi:10.1038/nature11280 (2012).
25 Migheli, A., Pezzulo, T., Attanasio, A. & Schiffer, D. Peripherin immunoreactive structures in amyotrophic lateral sclerosis. *Lab Invest* **68**, 185-191 (1993).
26 Gros-Louis, F. *et al.* A frameshift deletion in peripherin gene associated with amyotrophic lateral sclerosis. *J Biol Chem* **279**, 45951-45956, doi:10.1074/jbc.M408139200 (2004).
27 Chen, Y. Z. *et al.* DNA/RNA helicase gene mutations in a form of juvenile amyotrophic lateral sclerosis (ALS4). *Am J Hum Genet* **74**, 1128-1135, doi:10.1086/421054 (2004).
28 Al-Saif, A., Al-Mohanna, F. & Bohlega, S. A mutation in sigma-1 receptor causes juvenile amyotrophic lateral sclerosis. *Ann Neurol* **70**, 913-919, doi:10.1002/ana.22534 (2011).
29 Siddique, T. & Hentati, A. Familial amyotrophic lateral sclerosis. *Clin Neurosci* **3**, 338-347 (1995).
30 McDermott, C. J., Roberts, D., Tomkins, J., Bushby, K. M. & Shaw, P. J. Spastin and paraplegin gene analysis in selected cases of motor neurone disease (MND). *Amyotroph Lateral Scler Other Motor Neuron Disord* **4**, 96-99 (2003).
31 Daoud, H. *et al.* Exome sequencing reveals SPG11 mutations causing juvenile ALS. *Neurobiol Aging* **33**, 839 e835-839, doi:10.1016/j.neurobiolaging.2011.11.012 (2012).
32 Nakano, T., Nakaso, K., Nakashima, K. & Ohama, E. Expression of ubiquitin-binding protein p62 in ubiquitin-immunoreactive intraneuronal inclusions in amyotrophic lateral sclerosis with

dementia: analysis of five autopsy cases with broad clinicopathological spectrum. *Acta Neuropathol* **107**, 359-364, doi:10.1007/s00401-004-0821-7 (2004).
33 Ticozzi, N. *et al.* Mutational analysis reveals the FUS homolog TAF15 as a candidate gene for familial amyotrophic lateral sclerosis. *Am J Med Genet B Neuropsychiatr Genet* **156B**, 285-290, doi:10.1002/ajmg.b.31158 (2011).
34 Sreedharan, J. *et al.* TDP-43 mutations in familial and sporadic amyotrophic lateral sclerosis. *Science* **319**, 1668-1672, doi:10.1126/science.1154584 (2008).
35 Mackenzie, I. R. *et al.* TIA1 Mutations in Amyotrophic Lateral Sclerosis and Frontotemporal Dementia Promote Phase Separation and Alter Stress Granule Dynamics. *Neuron* **95**, 808-816 e809, doi:10.1016/j.neuron.2017.07.025 (2017).
36 Hirsch-Reinshagen, V. *et al.* Clinical and neuropathological features of ALS/FTD with TIA1 mutations. *Acta Neuropathol Commun* **5**, 96, doi:10.1186/s40478-017-0493-x (2017).
37 Rademakers, R. & van Blitterswijk, M. Excess of rare damaging TUBA4A variants suggests cytoskeletal defects in ALS. *Neuron* **84**, 241-243, doi:10.1016/j.neuron.2014.10.002 (2014).
38 Deng, H. X. *et al.* Mutations in UBQLN2 cause dominant X-linked juvenile and adult-onset ALS and ALS/dementia. *Nature* **477**, 211-215, doi:10.1038/nature10353 (2011).
39 van Es, M. A. *et al.* Genome-wide association study identifies 19p13.3 (UNC13A) and 9p21.2 as susceptibility loci for sporadic amyotrophic lateral sclerosis. *Nat Genet* **41**, 1083-1087, doi:10.1038/ng.442 (2009).
40 Nishimura, A. L. *et al.* A mutation in the vesicle-trafficking protein VAPB causes late-onset spinal muscular atrophy and amyotrophic lateral sclerosis. *Am J Hum Genet* **75**, 822-831, doi:10.1086/425287 (2004).
41 Ishigaki, S. *et al.* Physical and functional interaction between Dorfin and Valosin-containing protein that are colocalized in ubiquitylated inclusions in neurodegenerative disorders. *J Biol Chem* **279**, 51376-51385, doi:10.1074/jbc.M406683200 (2004).
42 Kotni, M. K., Zhao, M. & Wei, D. Q. Gene expression profiles and protein-protein interaction networks in amyotrophic lateral sclerosis patients with C9orf72 mutation. *Orphanet J Rare Dis* **11**, 148, doi:10.1186/s13023-016-0531-y (2016).
43 Kamel, F. *et al.* Amyotrophic lateral sclerosis, lead, and genetic susceptibility: polymorphisms in the delta-aminolevulinic acid dehydratase and vitamin D receptor genes. *Environ Health Perspect* **111**, 1335-1339 (2003).
44 Manabe, Y. *et al.* Early decrease of redox factor-1 in spinal motor neurons of presymptomatic transgenic mice with a mutant SOD1 gene. *Brain Res* **915**, 104-107 (2001).
45 Buee, L. *et al.* Apolipoprotein E in Guamanian amyotrophic lateral sclerosis/parkinsonism-dementia complex: genotype analysis and relationships to neuropathological changes. *Acta Neuropathol* **91**, 247-253 (1996).
46 Garofalo, O. *et al.* Androgen receptor gene polymorphisms in amyotrophic lateral sclerosis. *Neuromuscul Disord* **3**, 195-199 (1993).
47 Aggarwal, T. *et al.* Androgens affect muscle, motor neuron, and survival in a mouse model of SOD1-related amyotrophic lateral sclerosis. *Neurobiol Aging* **35**, 1929-1938, doi:10.1016/j.neurobiolaging.2014.02.004 (2014).
48 Droppelmann, C. A. *et al.* Detection of a novel frameshift mutation and regions with homozygosis within ARHGEF28 gene in familial amyotrophic lateral sclerosis. *Amyotroph Lateral Scler Frontotemporal Degener* **14**, 444-451, doi:10.3109/21678421.2012.758288 (2013).
49 Lattante, S. *et al.* ATXN1 intermediate-length polyglutamine expansions are associated with amyotrophic lateral sclerosis. *Neurobiol Aging* **64**, 157 e151-157 e155, doi:10.1016/j.neurobiolaging.2017.11.011 (2018).

50 Deng, L. B. *et al.* Polymorphism of rs3737597 in DISC1 Gene on Chromosome 1q42.2 in sALS Patients: a Chinese Han Population Case-Control Study. *Molecular Neurobiology* **54**, 3162-3179, doi:10.1007/s12035-016-9869-3 (2017).
51 Daoud, H. *et al.* Resequencing of 29 candidate genes in patients with familial and sporadic amyotrophic lateral sclerosis. *Arch Neurol* **68**, 587-593, doi:10.1001/archneurol.2010.351 (2011).
52 Lennon, M. J., Jones, S. P., Lovelace, M. D., Guillemin, G. J. & Brew, B. J. Bcl11b: A New Piece to the Complex Puzzle of Amyotrophic Lateral Sclerosis Neuropathogenesis? *Neurotox Res* **29**, 201-207, doi:10.1007/s12640-015-9573-5 (2016).
53 Couthouis, J., Raphael, A. R., Daneshjou, R. & Gitler, A. D. Targeted exon capture and sequencing in sporadic amyotrophic lateral sclerosis. *PLoS Genet* **10**, e1004704, doi:10.1371/journal.pgen.1004704 (2014).
54 Berjaoui, S. *et al.* Complex Inflammation mRNA-Related Response in ALS Is Region Dependent. *Neural Plast* **2015**, 573784, doi:10.1155/2015/573784 (2015).
55 Williams, K. L. *et al.* CCNF mutations in amyotrophic lateral sclerosis and frontotemporal dementia. *Nat Commun* **7**, 11253, doi:10.1038/ncomms11253 (2016).
56 Culotta, V. C. *et al.* The copper chaperone for superoxide dismutase. *J Biol Chem* **272**, 23469-23472 (1997).
57 Gros-Louis, F. *et al.* Chromogranin B P413L variant as risk factor and modifier of disease onset for amyotrophic lateral sclerosis. *Proc Natl Acad Sci U S A* **106**, 21777-21782, doi:10.1073/pnas.0902174106 (2009).
58 Sendtner, M. *et al.* Ciliary neurotrophic factor prevents degeneration of motor neurons in mouse mutant progressive motor neuronopathy. *Nature* **358**, 502-504, doi:10.1038/358502a0 (1992).
59 Xie, T. *et al.* Genome-wide association study combining pathway analysis for typical sporadic amyotrophic lateral sclerosis in Chinese Han populations. *Neurobiol Aging* **35**, 1778 e1779-1778 e1723, doi:10.1016/j.neurobiolaging.2014.01.014 (2014).
60 Fukada, Y. *et al.* Gene expression analysis of the murine model of amyotrophic lateral sclerosis: studies of the Leu126delTT mutation in SOD1. *Brain Res* **1160**, 1-10, doi:10.1016/j.brainres.2007.05.044 (2007).
61 Landers, J. E. *et al.* Reduced expression of the Kinesin-Associated Protein 3 (KIFAP3) gene increases survival in sporadic amyotrophic lateral sclerosis. *Proc Natl Acad Sci U S A* **106**, 9004-9009, doi:10.1073/pnas.0812937106 (2009).
62 Watanabe, M. *et al.* Genetic analysis of the cystatin C gene in familial and sporadic ALS patients. *Brain Res* **1073-1074**, 20-24, doi:10.1016/j.brainres.2005.12.046 (2006).
63 Lopez-Lopez, A. *et al.* CX3CR1 is a modifying gene of survival and progression in amyotrophic lateral sclerosis. *PLoS One* **9**, e96528, doi:10.1371/journal.pone.0096528 (2014).
64 Siddons, M. A., Pickering-Brown, S. M., Mann, D. M., Owen, F. & Cooper, P. N. Debrisoquine hydroxylase gene polymorphism frequencies in patients with amyotrophic lateral sclerosis. *Neurosci Lett* **208**, 65-68 (1996).
65 Ogawa, F. *et al.* DISC1 complexes with TRAK1 and Miro1 to modulate anterograde axonal mitochondrial trafficking. *Hum Mol Genet* **23**, 906-919, doi:10.1093/hmg/ddt485 (2014).
66 Daoud, H., Valdmanis, P. N., Dion, P. A. & Rouleau, G. A. Analysis of DPP6 and FGGY as candidate genes for amyotrophic lateral sclerosis. *Amyotroph Lateral Scler* **11**, 389-391, doi:10.3109/17482960903358857 (2010).
67 Kuzma-Kozakiewicz, M. *et al.* Dynactin Deficiency in the CNS of Humans with Sporadic ALS and Mice with Genetically Determined Motor Neuron Degeneration. *Neurochem Res*, doi:10.1007/s11064-013-1160-7 (2013).

68 Van Hoecke, A. *et al.* EPHA4 is a disease modifier of amyotrophic lateral sclerosis in animal models and in humans. *Nat Med* **18**, 1418-1422, doi:10.1038/nm.2901 (2012).
69 Couthouis, J. *et al.* Evaluating the role of the FUS/TLS-related gene EWSR1 in amyotrophic lateral sclerosis. *Hum Mol Genet* **21**, 2899-2911, doi:10.1093/hmg/dds116 (2012).
70 Banks, G. T. *et al.* Mutant glycyl-tRNA synthetase (Gars) ameliorates SOD1(G93A) motor neuron degeneration phenotype but has little affect on Loa dynein heavy chain mutant mice. *PLoS One* **4**, e6218, doi:10.1371/journal.pone.0006218 (2009).
71 Kaneb, H. M. *et al.* Deleterious mutations in the essential mRNA metabolism factor, hGle1, in amyotrophic lateral sclerosis. *Hum Mol Genet* **24**, 1363-1373, doi:10.1093/hmg/ddu545 (2015).
72 Schymick, J. C. *et al.* Progranulin mutations and amyotrophic lateral sclerosis or amyotrophic lateral sclerosis-frontotemporal dementia phenotypes. *J Neurol Neurosurg Psychiatry* **78**, 754-756, doi:10.1136/jnnp.2006.109553 (2007).
73 Drory, V. E., Birnbaum, M., Peleg, L., Goldman, B. & Korczyn, A. D. Hexosaminidase A deficiency is an uncommon cause of a syndrome mimicking amyotrophic lateral sclerosis. *Muscle Nerve* **28**, 109-112, doi:10.1002/mus.10371 (2003).
74 Wang, X. S. *et al.* Increased incidence of the Hfe mutation in amyotrophic lateral sclerosis and related cellular consequences. *J Neurol Sci* **227**, 27-33, doi:10.1016/j.jns.2004.08.003 (2004).
75 Kim, H. J. *et al.* Mutations in prion-like domains in hnRNPA2B1 and hnRNPA1 cause multisystem proteinopathy and ALS. *Nature* **495**, 467-473, doi:10.1038/nature11922 (2013).
76 van Es, M. A. *et al.* ITPR2 as a susceptibility gene in sporadic amyotrophic lateral sclerosis: a genome-wide association study. *Lancet Neurol* **6**, 869-877, doi:10.1016/S1474-4422(07)70222-3 (2007).
77 Vijayalakshmi, K. *et al.* Role of VEGF and VEGFR2 Receptor in Reversal of ALS-CSF Induced Degeneration of NSC-34 Motor Neuron Cell Line. *Mol Neurobiol* **51**, 995-1007, doi:10.1007/s12035-014-8757-y (2015).
78 Kaji, S. *et al.* Late-onset spastic paraplegia type 10 (SPG10) family presenting with bulbar symptoms and fasciculations mimicking amyotrophic lateral sclerosis. *J Neurol Sci* **364**, 45-49, doi:10.1016/j.jns.2016.03.001 (2016).
79 Tateno, M. *et al.* Mutant SOD1 impairs axonal transport of choline acetyltransferase and acetylcholine release by sequestering KAP3. *Hum Mol Genet* **18**, 942-955, doi:10.1093/hmg/ddn422 (2009).
80 Meyer, M. A. & Potter, N. T. Sporadic ALS and chromosome 22: evidence for a possible neurofilament gene defect. *Muscle Nerve* **18**, 536-539, doi:10.1002/mus.880180510 (1995).
81 Buscema, M., Penco, S. & Grossi, E. A Novel Mathematical Approach to Define the Genes/SNPs Conferring Risk or Protection in Sporadic Amyotrophic Lateral Sclerosis Based on Auto Contractive Map Neural Networks and Graph Theory. *Neurol Res Int* **2012**, 478560, doi:10.1155/2012/478560 (2012).
82 Li, P. A. *et al.* Up-regulation and altered distribution of lysyl oxidase in the central nervous system of mutant SOD1 transgenic mouse model of amyotrophic lateral sclerosis. *Brain Res Mol Brain Res* **120**, 115-122 (2004).
83 Aquilonius, S. M., Jossan, S. S., Ekblom, J. G., Askmark, H. & Gillberg, P. G. Increased binding of 3H-L-deprenyl in spinal cords from patients with amyotrophic lateral sclerosis as demonstrated by autoradiography. *J Neural Transm Gen Sect* **89**, 111-122 (1992).
84 Jossan, S. S., Ekblom, J., Aquilonius, S. M. & Oreland, L. Monoamine oxidase-B in motor cortex and spinal cord in amyotrophic lateral sclerosis studied by quantitative autoradiography. *J Neural Transm Suppl* **41**, 243-248 (1994).
85 Sundar, P. D. *et al.* Two sites in the MAPT region confer genetic risk for Guam ALS/PDC and dementia. *Hum Mol Genet* **16**, 295-306, doi:10.1093/hmg/ddl463 (2007).

86 Keeney, P. M. & Bennett, J. P., Jr. ALS spinal neurons show varied and reduced mtDNA gene copy numbers and increased mtDNA gene deletions. *Mol Neurodegener* **5**, 21, doi:10.1186/1750-1326-5-21 (2010).
87 Jackson, M., Morrison, K. E., Al-Chalabi, A., Bakker, M. & Leigh, P. N. Analysis of chromosome 5q13 genes in amyotrophic lateral sclerosis: homozygous NAIP deletion in a sporadic case. *Ann Neurol* **39**, 796-800, doi:10.1002/ana.410390616 (1996).
88 Blauw, H. M. *et al.* NIPA1 polyalanine repeat expansions are associated with amyotrophic lateral sclerosis. *Hum Mol Genet* **21**, 2497-2502, doi:10.1093/hmg/dds064 (2012).
89 Kulkarni, S. S. *et al.* Suppression of 5'-nucleotidase enzymes promotes AMP-activated protein kinase (AMPK) phosphorylation and metabolism in human and mouse skeletal muscle. *J Biol Chem* **286**, 34567-34574, doi:10.1074/jbc.M111.268292 (2011).
90 Murakami, T. *et al.* Early decrease of mitochondrial DNA repair enzymes in spinal motor neurons of presymptomatic transgenic mice carrying a mutant SOD1 gene. *Brain Res* **1150**, 182-189, doi:10.1016/j.brainres.2007.02.057 (2007).
91 Yamashita, S. *et al.* DJ-1 forms complexes with mutant SOD1 and ameliorates its toxicity. *J Neurochem* **113**, 860-870, doi:10.1111/j.1471-4159.2010.06658.x (2010).
92 Ozoguz, A. *et al.* The distinct genetic pattern of ALS in Turkey and novel mutations. *Neurobiol Aging* **36**, 1764 e1769-1764 e1718, doi:10.1016/j.neurobiolaging.2014.12.032 (2015).
93 Slowik, A. *et al.* Paraoxonase gene polymorphisms and sporadic ALS. *Neurology* **67**, 766-770, doi:10.1212/01.wnl.0000219565.32247.11 (2006).
94 Chen, Y. *et al.* Association analysis of PON polymorphisms in sporadic ALS in a Chinese population. *Neurobiol Aging* **33**, 2949 e2941-2943, doi:10.1016/j.neurobiolaging.2012.06.024 (2012).
95 Panas, M. *et al.* Genotyping of presenilin-1 polymorphism in amyotrophic lateral sclerosis. *J Neurol* **247**, 940-942 (2000).
96 Saunderson, R., Yu, B., Trent, R. J. & Pamphlett, R. A polymorphism in the poliovirus receptor gene differs in motor neuron disease. *Neuroreport* **15**, 383-386 (2004).
97 Liu, G. T. *et al.* Eosinophil-derived neurotoxin is elevated in patients with amyotrophic lateral sclerosis. *Mediators Inflamm* **2013**, 421389, doi:10.1155/2013/421389 (2013).
98 Tanaka, F., Waza, M., Niwa, J., Yamamoto, M. & Sobue, G. [Exploration of pathogenesis-associated molecules and development of disease models for sporadic ALS]. *Rinsho Shinkeigaku* **48**, 970-972 (2008).
99 Boutahar, N. *et al.* Differential effect of oxidative or excitotoxic stress on the transcriptional profile of amyotrophic lateral sclerosis-linked mutant SOD1 cultured neurons. *J Neurosci Res* **89**, 1439-1450, doi:10.1002/jnr.22672 (2011).
100 Rothstein, J. D., Van Kammen, M., Levey, A. I., Martin, L. J. & Kuncl, R. W. Selective loss of glial glutamate transporter GLT-1 in amyotrophic lateral sclerosis. *Ann Neurol* **38**, 73-84, doi:10.1002/ana.410380114 (1995).
101 Landers, J. E. *et al.* Reduced expression of the Kinesin-Associated Protein 3 (KIFAP3) gene increases survival in sporadic amyotrophic lateral sclerosis. *P Natl Acad Sci USA* **106**, 9004-9009, doi:10.1073/pnas.0812937106 (2009).
102 Corcia, P. *et al.* Abnormal SMN1 gene copy number is a susceptibility factor for amyotrophic lateral sclerosis. *Ann Neurol* **51**, 243-246 (2002).
103 Veldink, J. H. *et al.* Homozygous deletion of the survival motor neuron 2 gene is a prognostic factor in sporadic ALS. *Neurology* **56**, 749-752 (2001).
104 Kruger, R., Muller, T. & Riess, O. Involvement of alpha-synuclein in Parkinson's disease and other neurodegenerative disorders. *J Neural Transm (Vienna)* **107**, 31-40, doi:10.1007/s007020050002 (2000).

105 Andreassen, O. A. *et al.* Partial deficiency of manganese superoxide dismutase exacerbates a transgenic mouse model of amyotrophic lateral sclerosis. *Ann Neurol* **47**, 447-455 (2000).
106 Jones, A. R. *et al.* Stratified gene expression analysis identifies major amyotrophic lateral sclerosis genes. *Neurobiol Aging* **36**, 2006 e2001-2009, doi:10.1016/j.neurobiolaging.2015.02.017 (2015).
107 Mitsumoto, H. *et al.* Phenotypic and molecular analyses of primary lateral sclerosis. *Neurol Genet* **1**, e3, doi:10.1212/01.NXG.0000464294.88607.dd (2015).
108 Chesi, A. *et al.* Exome sequencing to identify de novo mutations in sporadic ALS trios. *Nat Neurosci* **16**, 851-855, doi:10.1038/nn.3412 (2013).
109 Schymick, J. C. *et al.* Genome-wide genotyping in amyotrophic lateral sclerosis and neurologically normal controls: first stage analysis and public release of data. *Lancet Neurology* **6**, 322-328, doi:10.1016/S1474-4422(07)70037-6 (2007).
110 Korac, J. *et al.* Ubiquitin-independent function of optineurin in autophagic clearance of protein aggregates. *J Cell Sci* **126**, 580-592, doi:10.1242/jcs.114926 (2013).
111 Hermosura, M. C. *et al.* A TRPM7 variant shows altered sensitivity to magnesium that may contribute to the pathogenesis of two Guamanian neurodegenerative disorders. *Proc Natl Acad Sci U S A* **102**, 11510-11515, doi:10.1073/pnas.0505149102 (2005).
112 Lambrechts, D. *et al.* VEGF is a modifier of amyotrophic lateral sclerosis in mice and humans and protects motoneurons against ischemic death. *Nat Genet* **34**, 383-394, doi:10.1038/ng1211 (2003).
113 Meisler, M. H. *et al.* Evaluation of the Golgi trafficking protein VPS54 (wobbler) as a candidate for ALS. *Amyotroph Lateral Scler* **9**, 141-148, doi:10.1080/17482960801934403 (2008).
114 Sakamoto, K., Tamamura, Y., Katsube, K. & Yamaguchi, A. Zfp64 participates in Notch signaling and regulates differentiation in mesenchymal cells. *J Cell Sci* **121**, 1613-1623, doi:10.1242/jcs.023119 (2008).
115 Stevens, D. A. *et al.* Parkin loss leads to PARIS-dependent declines in mitochondrial mass and respiration. *Proc Natl Acad Sci U S A* **112**, 11696-11701, doi:10.1073/pnas.1500624112 (2015).
116 Iida, A. *et al.* A functional variant in ZNF512B is associated with susceptibility to amyotrophic lateral sclerosis in Japanese. *Hum Mol Genet* **20**, 3684-3692, doi:10.1093/hmg/ddr268 (2011).

Supplementary Table 2

Table of protein products of all genes related to ALS and their binding partners, determined by experimental findings. Reference for each protein-protein interaction is included, and proteins that bind to more than 3 ALS related gene product is marked Bold.

1	AGT	ACE, ACE2, AGTR1, Agtr1b, AGTR2, AGTRAP, Angiotensin II receptor type 1, **ATP6AP2**[1], AVP, BCL3, BLVRA, CALCA, **CEBPA**[2], CEBPB, CEBPD, CNP, **CREBBP**[3], CRH, CTSG, DBP, ECE1, ENPEP, **EP300**[4], **EWSR1**[5], GSDMB, HDL, **HIF1A**[6], Histone h4, **HNF1A**[7], **HNRNPK**[8], **ITSN2**[1], **JUND**[6], KLK1, MME, MMEL1, MMP9, **NKX2-1**[9], **NOS3**[10], NPPA, NPPB, NPY, **NR1H2**[11], **NR2F1**[12], PRCP, **REN**[1], STAT, **STAT1**[13], TAC1, TEK, Tgf beta, THOP1, TIE1, USF1, USF2
2	ALAD	26s Proteasome,ALAD,CNN2,delta-aminolevulinic acid,HSD17B10,**KCNMA1**[14],miR-3180-3p (and other miRNAs w/seed GGGGCGG),porphobilinogen synthase ,UBC
3	ALS2	ALS2,**CDC37**[15],**DNAAF2**[16],GDP,**GRIP1**[17],**HNF1A**[18],**HNF4A**[18],RAB17,Rab5,RAB5A,RAB5B,RAB5C, **RAC1**[19],**SOD1**[20],**UXT**[21],VCP,**YWHAB**[22]
4	ALS3	Igf, IGF1, IGF2, IGFALS, IGFBP5, STAT5a/b, ZBTB20
5	ALS7	No interactions
6	ANG	2'-cytidylic acid, AARS, ABCE1, ABCG8, ACTB, Actin, **ACTN2**[23], ACTN4, AIMP1, **ALDOA**[24], ANG, ANXA2, ATP6AP1, copper, CREB3L4, ELANE, **EZR**[24], **FHL3**[25], FST, GAPDH, heparin, Histone h3, **HK1**[24], **HMGB1**[24], **HNF4A**[18], Hsp70, Hsp90, LARS, **MDM2**[26], **MYH14**[24], **NFE2L2**[27], NR1I2, **PHGDH**[24], **PKM**[24], **PLSCR1**[28], **PPIA**[24], **PRDX1**[24], **PTEN**[29],RARS, **RNH1**[16], **RPL11**[24], RPL4, RPLP0, RPS19, **RPS5**[24], **STAT1**[24], TDGF1, **TP53**[26], **UCHL1**[24], **VIM**[24], **YWHAB**[24], YWHAZ
7	APOE	4-hydroxynonenal, **A2M**[30], ABCA1, ACTG1, AEBP1, **ALB**[31], ANKH, **APOA1**, APOA2, APOA4, APOA5, APOC1, Apoc1, APOC2, APOC3, Apoc3, **APOE**[20], **APP**[32], ARFGAP1, B2M, BDNF, BGN, C19orf52, **CDC37**[33], CES1, CETP, cholesterol, chondroitin sulfate, chondroitin sulfate proteoglycan, CLSTN3, **CNTF**[34], CREB1, **CREM**[35], CRP, **CRYAB**[36], **CSNK2A1**[37], CYP2C18, CYP2C8, **DHX9**[38], dimyristoylphosphatidylcholine, ECSIT, **ELAVL1**[33], EPN2, ESR1, F2, FARSA, FBXL12, FOXG1, FXYD7, GCDH, **HBB**[39], HDL, heparan sulfate, heparan sulfate proteoglycan, heparin, HLA-DPA1, **HOXA10**[40], HP, HTRA1, **IFIT3**[33], IFIT5, Ins1, IQSEC1, **JRK**[41], JUN, **KCNMA1**[14], **KLF3**[42], L-alpha-palmitoyloleolyl-phosphatidylcholine, Laminin, LCAT, **LCK**[43], LDL, LDLR, lipid, lipopolysaccharide, LONP1, LOXL4, LPA, LPL, LRP, **LRP1**[44], LRP2, LRP8, **MAPT**[45], MAST1, MID1IP1, MMP3, MMRN1, **NEFM**[45], **NFKB1**[46], NOS3, Nr1h, **NR1H2**[47], **NR1H3**[48], NR2C2, **NR4A1**[49], PARP, PCMT1, PDCD4, phosphatidylcholine, phospholipid, PLEKHA6, PLTP, **PML**[50], **PPARG**[51], PRAM1, PRDX2, PRKDC, PRNP, proteoglycan, PSEN1, RELA, RHEB, RNF32, **RORA**[52], RPL4, Rxr, **RXRA**[47], SAA, **SCARB1**[53], SERPINA3, **SET**[54], SORL1, **SORT1**[55], **SP1**[46], **SPIB**[56], **ST13**[33], **STAT1**[57], **STAT6**[58], TFAP2A, thyroid hormone, TMCC2, **TP53**[59], transglutaminase, TREM2, triolein, TYRO3, UBC, VDAC1, vitamin K, VLDL, VLDLR, **XRCC5**[38], XRCC6, ZIC1, ZIC2, **ZNF202**[60], ZNF558
8	AR	ATP2A2, **ATP4A**[61], ATRX, AVPR1A, AZD3514, AZGP1, **BAG1**[62], bicalutamide, bisphenol A, BLK, BLNK, BMF, BMP15, BMS-641988, BMX, BRCA2, BRD8, **BTG2**[63], **BTK**[64], C18orf21, **C3**[65], C4A/C4B, CA4, CA7, **CACNA1A**[66], CACNA1C, CALCB, **CALR**[67], **CAMK2A**[68], CAMKK2, CAPZA3, carnosol, CASP1, **CASP7**[69], CASQ2, CAST, CAV2, CBX1, Ccdc74a, CCDC85B, CCL3L3, CCNA1, CCND3, CCNH, CDC25B, CDH11, **CDH2**[70], CDK11A, CDK11B, CDK14, **CDK5**[71], CDK7, **CDK9**[72], Cdkn1c, CDT1, CEND1, Ces1e, CGA, CHD8, chlormadinone acetate, CHRNA1, CHRNB1, CHTF18, CKAP4, CLCN1, CLDN11, CLDN3, Cmtm2a, COL3A1, COPS2, cortisone, COX5B, Cox5b, CRISP1, **CRKL**[73], **CRY1**[74], CSRP3, Cst12, CTDSP2, CTNNBIP1, CTSO, **CUL4B**[75], CUX1, CUX2, CYC1, CYP11A1, CYP17A1, Cyp2j13, cyproterone acetate, DACH1, danazol, **DAP3**[76], DAPL1, DAXX, DBI, DCAF6, **DCC**[77], DCTPP1, DDB2, DDC, DDT, DDT, Defb2, dehydroisoandrosterone, DENND5A, DEPDC1, DES, DHCR24, DHH, DHT-AR, diethylstilbestrol, DISP2, DMRT1, Dmrtc1a, DNAJC7, drospirenone, Dynein, DYRK1A, E2F4, EDNRA, EDNRB, EFCAB6, EGR4, EHD3, EIF2B5, **ELF3**[78], ELK4, ELMO1, ELOVL2, EMB, enzalutamide, EPB41L4B, EPHX1, EPI-506, epinephrine, EPPIN, EPPIN-WFDC6, **ERCC2**[79], ERCC3, **ERG**[80], ESPN, ETV1, ETV5, F9, FABP5, **FAF2**[43], FAM174B, FAM89B, FES, FGF10, FGF7, FGF8, FGFR2, FGR, **FHL2**[81], Filamin, **FKBP4**[82], FLII, fludrocortisone, fluoxymesterone,

		flutamide, FOXA1, FOXH1, **FOXM1**[83], FOXP2, FSHR, FXYD3, GADD45GIP1, GAK, galeterone, **GATA1**[84], **GDF15**[85], **GDF9**[86], GDI1, GDNF, ginsenoside Re, GLCCI1, **GLI1**[87], **GLI2**[87], GLI3, **GNB1**[88], GPR182, GPX5, GRAP2, **GRB7**[64], **GRIP1**[89], GSK3A, GSN, GSTM1, **GSTP1**[90], **GTF2F1**[91], GTF2F2, GTF2H1, GTF2H2, GTF2H3, GUSB, **HAP1**[92], HCK, **HDAC4**[93], **HDAC7**[94], **HEYL**[94], HIP1, **HIPK3**[95], **HMGB1**[96], **HMGB2**[96], **HOXA13**[97], HOXB13, HSD17B3, HSD3B1, HSD3B2, HSH2D, HSP, **HSPB2**[98], HSPB7, **HSPH1**[99], hydrocortisone, hydroxyflutamide, IDE, **IGFBP3**[100], IGFBP5, IL1R1, **IL6ST**[101], IMPDH2, INHA, INPPL1, INSL3, IPO7, ITGA2, JMJD1C, JUN/JUNB/JUND, **JUND**[102], KAT2B, **KAT5**[103], KAT7, KCNA1, KCNJ2, KCNJ3, KCNQ1, KDM3A, KDM4A, KDM4C, KDM4D, KDM5B, KIF1A, **KIFAP3**[104], KISS1R, KLF8, KLK2, KLK3, KLKB1, KRT14, LAMA5, LAMC3, LATS2, **LCK**[64], Lcn5, LCOR, LHCGR, LMOD1, **LRRK1**[105], Ly6a (includes others), MAGEA11, MAK, Marcks, MATK, **MCM4**[105], **MDN1**[104], MED12L, **MED24**[104], medroxyprogesterone acetate, MEF2C, megestrol acetate, MEIS1, mesterolone, methyltestosterone, metribolone, mibolerone, MID1, mifepristone, mir-1, mir-10, mir-133, mir-221, MLH3, MME, MRAS, MSI1, MSMB, MSTN, MT1X, **MTA2**[106], MYH6, MYL3, MYO1B, MYOG, MYOM1, MYOT, MYOZ2, N-cor, **NACA**[1063], nandrolone, nandrolone decanoate, nandrolone phenpropionate, **NAP1L1**[105], **NCOA2**[107], **NCOA4**[108], **NCOA6**[109], **NCOR1**[110], **NCOR2**[110], Ncx, **NDE1**[111], **NDRG1**[100], Nectin, NELFCD, NFAT5, **NFKB2**[112], NGFR, nilutamide, NKX3-1, NLRP10, NMRK2, NONO, norethindrone, NR0B1, **NR0B2**[113], NR2C1, NR2C2, **NR2F2**[114], NR5A1, NR5A2, Nrgn, NRIP1, NSD1, NTF4, Nuclear factor 1, OCLN, ODC1, ODM-201, ostarine, OVGP1, OVOS2, oxandrolone, oxymetholone, P-TEFb, p160, P2RX1, **PA2G4**[115], PAGR1, PAK6, **PARK7**[116], PATZ1, PAX6, Pbsn, **PCBP1**[117], **PCBP2**[117], PCGEM1, PDE9A, PDGFA, **PDGFRA**[118], PDYN, PELP1, PEMT, PENK, PER1, PGC, **PHB**[119], **PIAS1**[120], PIAS2, **PIAS3**[120], PIAS4, PIGR, **PIK3R2**[64], PIK3R3, PIM1, PIP, **PKN1**[121], PLAGL1, PLAT, **PLCG2**[64], Pln, PLS3, PMEPA1, PNMT, PNRC1, POMT2, POU2F1, POU2F2, POU3F3, **POU4F1**[122], POU5F2, PPID, **PPP5C**[82], **PRDX1**[123], Pre2, PRKG1, Prm1, **PRMT1**[124], PRPF6, PRPF8, PSCA, PSMC3, PSMC3IP, PSPC1, **PTBP1**[108], PTCD3, **PTCH1**[118], **PTGDS**[125], PTGES3, PTGS1, PTK6, **PURA**[126], PYCR2, R5020, RAB3C, RAC3, **RACK1**[127], **RAD54L2**[128], RAD9A, **RAN**[129], RANBP9, RARRES1, **RASA1**[64], Rb, RBL1, RCHY1, recombinant human endostatin, **REN**[125], REPS2, RHBG, RHOB, Rhox5, RLN1, RNASEL, **RNF14**[130], RNF20, **RNF4**[131], RNF40, RNF6, RP2, RREB1, **RUVBL1**[132], **RXRB**[133], S1PR3, SART3, SCAP, SCEL, Scgb1b27 (includes others), Scgb2b27 (includes others), SCN4A, SELENBP1, SERPINB5, SETD7, **SFPQ**[134], **SGK1**[135], SH2D1A, SH2D1B, **SH2D2A**[64], SH2D3C, SH3YL1, SHB, SHBG, SHC3, SHC4, SHE, SHR3680, SIAH2, Sik1, **SIN3A**[136], **SIN3B**[136], SKP2, **SLC25A4**[137], SLC26A7, SLC2A3, **SLC38A5**[84], SLC39A8, SLC45A3, SLC7A4, SLCO3A1, **SMAD1**[138], **SMARCA2**[104], **SMARCC1**[139], SMARCD1, **SMARCE1**[140], SMTN, SMYD1, SNAI2, SNRPA, SNURF, **SOCS6**[64], SORBS3, SORD, SOX9, SPDEF, SPINK1, spironolactone, SPO11, SPOP, **SRC**[64] (family), SRCAP, SRD5A1, SRD5A2, **SRSF1**[141], SRY, stanozolol, STAP1, STAR, **STAT5B**[105], STEAP2, steroid, STK39, **STK4**[142], **STUB1**[82], SULT1E1, SULT2A1, Sult5a1, **SUMO1**[93], SVIL, Svs2, TAB2, TACSTD2, TADA3, **TAF1**[143], **TAF4**[144], TAF7L, TAP1, TARP, TAT, TBL1XR1, TBXAS1, TCF19, TCF20, TCF21, **TDG**[145], TEAD3, testosterone cypionate, testosterone enanthate, testosterone propionate, Testosterone-AR dimer, TF, **TFEB**[146], TFF1, TFIIH, TGFB1I1, TGFB2, TGIF1, thymidine kinase, **TJP1**[84], TLX3, TMEM158, TMF1, TMPRSS2, TNFAIP2, TNK2, TNNT2, TNS1, TNS4, TPD52, **TRIM24**[147], TRIM25, TRIM68, TRMT12, **TSG101**[148], TSPAN8, TTC5, **TUBB3**[149], **TWIST1**[150], **U2AF2**[141], UBA3, UBE2L3, **UBE3A**[151], **USP10**[152], **USP12**[153], USP26, USP7, **UXT**[154], VAV1, VAV2, VAV3, **VCL**[155], VIP, VSNL1, **WDR77**[138], WEE1 , WIPF1, WIPI1, **WT1**[84], **XRCC5**[156], XRCC6, **YES1**[64], **YWHAH**[157], ZBTB1, **ZBTB16**[104], ZBTB32, **ZBTB7A**[110], ZEB1, ZK 230211, ZMIZ1, ZMIZ2, ZNF318, ZNF451, ZYX
9	ARGHEF28	CLOCK, Filamin, Focal adhesion kinase, **FUS**[158], GNA12, GNA13, GNAQ, GNAZ, MAPK8IP1,Ras homolog, RHPN1, SQSTM1, **TARDBP**[158]
10	ATXN2	**ACTN1**[159], **ACTN2**[159], **ATXN1**[159], ATXN2, **ATXN2L**[160], **BAG6**[159], BCAT1, BMI1, CAND1, **CARD8**[16], CBL, CCNDBP1, CELA2B, **CHGB**[29], **CUL3**[161], **DDX6**[16], **DLG4**[162], **EGFR**[163], FGL1, **FOS**[164], **FOXP3**[165],

		**G3BP1**[166], **GFI1B**[159], **HTT**[167], IDH3B, **ISG15**[168], ITPR, , **ITPR1**[169], **KIF1BP**[159], LCP1, **MDC1**[170], MOV10, **MYCN**[171], **NCOA4**[159], **NTRK1**[172], NXF1, **PABPC1**[173], PARK2, PLS3, **RBFOX1**[159], **RBFOX2**[174], **RBPMS**, RPL10, **SEC16A**[43], SH3GL2, SH3GL3, **SH3KBP1**[163], **SKAP1**[174], **SRC**[163], STXBP5L, TDRD7, **TSG101**[175], **UBR5**[176], **VHL**[177], **WWOX**[178], YEATS4, ZNRD1
11	B4GALT6	A4GALT, B4GALT6, **ESRRA**[179], Galactosyltransferase beta 1,4, ST3GAL5
12	BCL11B	ADORA1, ARHGAP6, BCL11B, CBX5, **CHD4**[180], CHRM4, EP300, **FEZF2**[181], Foxp1, **FOXP3**[165], **GATA3**[182], **GRIA1**[183], HDAC1, **HDAC2**[180], **HDAC3**[184], HDAC6, **HTT**[185], **IFNG**[186], **IL10**[187], IL17A, IPMK, KCNIP2, MAP3K8, **MBD3**[180], **MDM2**[188], miR-292b-5p (and other miRNAs w/seed CUCAAAA), miR-515-3p (and other miRNAs w/seed AGUGCCU), **MTA1**[180], **MTA2**[180], NCEH1, **NECTIN3**[183], **NEK4**[189], **NETO1**[183], NGEF, **NOTCH1**[190], **NR2F1**[191], **NR2F2**[191], NR2F6, OPRM1, Pcp4l1, PLXND1, PPP1R1B, **RBBP4**[180], **RBBP7**[180], RELA, **RELN**[183], Rxr, SATB2, SIRT1, **SKI**[192], **SP1**[184], SPRY1, **SUMO3**[193], SUV39H1, TBR1, **TCF7**[194], TGFBR1, **TNF**[186], **TP53**[188], Vmn1r171 (includes others), Vmn1r172 (includes others), Vmn1r180 (includes others), Vmn1r232, Vmn1r47/Vmn1r48, Vmn1r49 (includes others), Vmn1r51 (includes others), Vmn1r63 (includes others), Vmn2r123, Vmn2r26 (includes others), Vmn2r32 (includes others), Vmn2r88 (includes others), Vmn2r89
13	BCL6	**A2M**[195], **ACAN**[196], **AES**[197], AICDA, ALCAM, ARHGAP35, ARHGEF9, ARNT2, ATF7, **ATN1**[198], BACH2, **BAX**[199], BCL11A, **BCL2**[200], BCL2A1, BCL2L1, BCL3, **BCL6**[201], BCL6B, BCOR, BLZF1, CABP4, **CACNA1A**[202], CCDC36, **Ccl2**[203], **CCL2**[204], CCL3, CCL3L3, CCL4, Ccl6, Ccl7, **CCND1**[205], CCND2, CCR8, CD151, CD2, **CD44**[196], CD69, CD80, CDK19, CDK8, **CDKN1A**[206], CDKN1B, **CDKN2A**[207], CHD3, CHM, **COL1A1**[196], COL2A1, **CREBBP**[208], **CSF1**[209], CSMD1, CTBP1, CTCF, CTLA4, **CTNNB1**[210], **CUL3**[211], CUTC, CXCL10, CXCL3, CXCR5, **DDX6**[202], DNAH9, **DROSHA**[202], DVL2, E2F3, EBF1, EBI3, **EGR2**[212], EGR3, EHMT1, EIF2AK3, EIF4ENIF1, **ENO1**[213], ENTPD1, EP300, EPHB6, ERCC5, **FAS**[214], FBXO11, Fcer, FCER2, **FGF2**[215], FMOD, **FOXM1**[216], **FOXO4**[217], **FOXP3**[215], **GATA1**[197], **GATA3**[215], **GLI1**[218], **GLI2**[218], **GLRX3**[197], **GOLGA2**[197], Gzmb, HAPLN1, **HBEGF**[219], HDAC1, **HDAC2**[220], **HDAC4**[221], **HDAC5**[221], **HDAC7**[221], HDAC9, HELZ2, **HNF1A**[202], **HNF4A**[18], HSF4, HSP90AA1, **HTT**[204], **ID2**[222], **ID3**[222], IDI2, **IL10**[223], IL12A, IL13, IL13RA1, IL17A, IL18, IL1A, IL1B, IL21R, IL23A, IL2RA, IL2RB, IL32, **IL4**[224], IL4R, IL5, **IL6**[225], IL6R, IL7R, IL9, IRF4, **IRF8**[226], ITGA3, JUN, **JUNB**[227], **JUND**[227], KDM2A, KIF13B, **KIFC3**[201], **KMT2B**[202], LIMS3/LIMS4, LITAF, LPAR2, LPAR4, LSM1, Ly6a (includes others), MAF, MAPK8IP3, **MAX**[228], **MBD3**[229], MCM3AP, MDFI, MED1, MED17, **MED23**[202], **MED6**[230], MGP, miR-30c-5p (and other miRNAs w/seed GUAAACA), miR-339-5p (and other miRNAs w/seed CCCUGUC), mir-361, mir-9, MIR155HG, MIR17HG, MITF, MNT, MTA3, **MTUS2**[197], MYB, **MYC**[228], **MYOD1**[231], NACC1, **NCOR1**[202], **NCOR2**[232], **NFKB1**[233], **NKRF**[202], NOS2, **NOTCH1**[234], OLA1, **OSBPL1A**[202], PAK1, PANX3, PARP12, PATZ1, PCDH9, PCGF1, PDCD2, PELI1, PIAS2, PIK3CG, PIKFYVE, **PIN1**[216], PLA2G2A, PLPP3, **PML**[235], POU2AF1, POU2F3, **PPARA**[236], PPARD, **PPARG**[236], **PPARGC1A**[202], PRDM1, PRKCE, PRKD3, **PTEN**[237], **PTPRO**[238], **RAF1**[239], RGS4, RORC, RUNX1T1, SAE1, SAP30, SENP7, **SIAH1**[201], **SIN3A**[202], **SIN3B**[228], **SIRT1**[172], SMAD3, **SMAD4**[240], **SMARCA4**[202], SMO, SOCS3, **SOX5**[196], SOX6, SOX9, **SP1**[241], SPI1, **SPIB**[242], SPP1, SPTLC2, **STAT3**[243], STAT5a/b, **STAT6**[244, 245], TBX21, **TCF3**[38], TFIP11, Tgf beta, **TGFB1**[215], TH2 Cytokine, TNFRSF18, TNFRSF8, **TP53**[210], **TRAF1**[201], TRAF3IP3, **TRIB3**[202], **TRIM27**[197], TSN, **TWIST1**[210], USP7, UTP6, WDR35, WNK4, WNT16, XBP1, **YY1**[202], **ZBTB16**[235], ZBTB17, ZBTB6, **ZBTB7A**[246], ZBTB7B, ZMYND8, ZNF443
14	C1orf27	C1orf27, FAM177A1, UBXN4
15	C9orf72	**APP**[247], **ATG101**[260], **ATG13**[196], CRX, EIF2B2, **ELAVL1**[248], ESRRB, HNRNPA2B1, **HSBP1**[260], **MMS19**[249], **NFS1**[260], NMI, PLK1, **RB1CC1**, **REL**[201], **SMCR8**[197], **SRPK1**[250], **SRSF1**[251], TBC1D32, **ULK1**[252], WDR41
16	APEX1	ABCE1, AICDA, **ANP32A**[253], APEX1, **APP**[247], **ARF6**[177], Arnt-Hif1a, ASCL2, **ATF4**[254], cation, CCNA1, **CDK5R1**[255], CFAP74, **EED**[256], **EEF1A1**[257], **EEF2**[257], **EGFR**[258], **EIF1B**[177], EIF6, EP300, **EPAS1**[259], ERCC1, **ERCC2**[199], ERCC4, FCGR2A, FEN1, **FUS**[199], **GADD45A**[260], GFRA1, GZMA, GZMK, HDAC1, **HDAC2**[261], **HDAC3**[262], **HIF1A**[263], **HLA-B**, **HMGA1**[264], HMGA2, **HMGB1**[265], **HNRNPL**[266], HOXC13, Hsp70,

		HSPA1A/HSPA1B, HTRA1, **ILF2**[199], ILF3, JUN, KLHL36, **KPNA2**[267], KPNA3, **KRT8**[268], **LMNA**[199], **MCC**[177], MCM2, MCM3, **MCM4**[199], **MCM5**[257], **MDM2**[269], MORF4L1, **MTA1**[270], MUTYH, **MYC**[271], **MYCN**[272], **NCL**[199], **NDRG1**[273], NPM1, **NTRK1**[172],NUDT3, **OGG1**[274], ORC2, PAK2, **PARP1**[199], **PCNA**[275], Pde, **PLCB1**[276], POLB, POLR3D, POU2F1, **PPARA**[277], PRDX6, PRKDC, **PRPF19**[206], PTH, **RAD50**[257], **RNF4**[278], **RPA1**[279], **RPA2**[279], **RPA3**[279], RPSA, **RUVBL2**[257], **SET**[253], SET complex, **SIN3A**[261], SIRT1, **SRPK1**[250], SRPK2, **STAT3**[280], TCF21, **TCP1**[268], **TDG**[281], **TERF1**[282], **TERF2**[282], **TERF2IP**[282], TFAP4, **TOP1**[199], **TP53**[283], **TRAF2**[284], TRAF6, **TRIM28**[199], **TXN**[285], TXNRD1, **UBE2I**[286], Vegf, **WDR77**[268], **XPC**[287], XRCC1, **XRCC5**[288], **XRN2**[199], **YBX1**[289]
17	CCNF	**CCNB1**[290],CCNF, CCP110, CDC6, CDK1, CUL1, **E2F1**[291], **FOXM1**[292], **FOXO1**[293], miR-16-5p (and other miRNAs w/seed AGCAGCA), miR-503-5p (miRNAs w/seed AGCAGCG), MYBL2, NEURL4, **NFATC2**[294], NUSAP1, POU5F1, **PSMA6**[295], RBX1, RCAN1, RRM1, RRM2, SCYL1, **SKP1**[296], TEAD2, YAP1
18	CCS	APBA1, **BACE1**[297], CAPZA2, CCS, CCS-Cu2+, cobalt, copper, Cu2+, **CUL4B**[43], **HIF1A**[298], SCRN2, SLC31A1, **SOD1**[43], **TCF3**[299], **XIAP**[300], **YWHAG**[22], zinc, Zn2+
19	CDH13	ADIPOQ, **AURKA**[301], Cadherin, **CASP8**[301], **CCND1**[301], CDH13, **CHEK2**[301], CREB1, DGUOK, **DNAAF2**[16], **HAX1**[16], **HMMR**[301], **HTT**[302], **ILK**[303], **INSR**[304], MAPK6, **MTNR1B**[305], NFKBIA, **NOVA1**[306], NUFIP1, PTPN1, **RAD51**[301]
20	CDH22	AKAP5, CDH22, **CTNNB1**[307], IKZF1, UBC
21	CHCHD10	CHCHD10, **KLF3**[37]
22	CHGB	**ACACA**[29], ANXA7, **APP**[247], **ATN1**[198], ATXN2, CBFB, CCDC92, **CDKN1A**[308], CHGA, Chromogranin, CREB1, FGFR3, **Hmgb1**[309], ITPR, **ITPR1**[310], **ITPR2**[310], ITPR3, **LRRK1**[311], **LRRK2**[311], MAPK3, MARK3, MGLL, MOGS, **NFE2L2**[312], **OGG1**[29], PARK2, **PDX1**[313], PHF21A, PKN2, POLD1, POLR2E, **PTEN**[29], RAC2, RAP1B, RCC1, **REST**[314], RXRG, **S100A8**[29], **SAFB2**[29], SGK223, SLC25A6, **SMAD2**[315], **SOD1**[315], **TAZ**[29], **TK1**[308], **TUBB2A**[29], TWF2, **UBQLN1**[316], UBTF, VGF, YLPM1
23	CHMP2B	ACO2, **ACTN1**[317], ACTR2, ADD2, ADD3, AGAP2, **AHSA1**[317], **ANK2**[317], ANK3, AP3D1, AP3M2, ARPC4, ARPC5L, ATAD3A, ATP2B1, ATP2B2, ATP5C1, ATP5F1, ATP5J2, ATP5O, ATP6AP2, **ATP6V1A**[317], ATP6V1B1, **BAG6**[317], **BMPR1A**[43], BUB1, **C1QBP**[43], **CACNA1A**[317], CAMK2B, CAMK2G, CAND1, CAPZA2, CAPZB, CASKIN1, CD2AP, CENPF, **CEP55**[43], CHMP1A, CHMP2, CHMP2A, **CHMP2B**[318], CHMP3, **CHMP4B**[43], **Chmp4b**[43], CHMP5, CHMP6, **CRYM**[317], **Cyfip2**[317], DBN1, **Dclk1**[317], **DLG4**[241], **DNM1**[317], DPY30, DSG1, **DYNC1H1**[317], **DYNLL1**[317], **EIF4A2**[317], EPB41L1, **EPB41L3**[317], FASN, **GET4**[317], **GJA1**[317], **GLUD1**[317], **GNB1**[317], **GOLT1B**[43], GPHN, GRIN2B, **HADHA**[317], **HK1**[317], HOMER1, HSPA12A, IDH2, **IMMT**[317], **JUP**[43], KCNH1, KIF2A, Ldha/RGD1562690, LTN1, MAD2L1, MALSU1, MAOA, MINK1, MRAS, **MRPL48**[317], MRPS9, **MYH10**[317], **MYH14**[43], **MYH9**[317], MYO1D, **MYO6**[317], **NCKAP1**[317], NDUFA4, NDUFS2, NDUFS7, NDUFV1, NDUFV2, **NR4A1**[315], **NSF**[317], **NUP98**[43], OGT, PAPSS1, **PCLO**[317], **PDCD6IP**[317], PDE2A, PDHB, PFKM, **PFKP**[317], **PHGDH**[318], PLEC, PNMA1, **PPP1CA**[317], **Ppp1cc**[317], PRKAR2B, PRKCB, PRKCG, PTPN23, **PURA**[317], PYGB, **RAB14**[317], RAB18, RAB2A, RAB3C, RAB5C, RAB7A, RABL6, **RAC1**[317], **RAN**[317], **RAPGEF4**[317], RCC1, RIMS1, **Rpl23a**[319], RTN1, SEPT5, SFXN3, SGO1, SHANK1, SIPA1L1, SLC2A1, SNAP25, SNAP91, SNF8, SPAST, SPTAN1, **SPTBN1**[317], SPTBN2, SQSTM1, **SRCIN1**[317], SSBP1, **STX1B**[317], SUCLA2, SYN2, Syt7, TEN1, **TERF2IP**[282], TIMELESS, TIPIN, Tpi1 (includes others), **TRAF2**[5], **TSG101**[43], **UBL4A**[317], UQCRC2, USP54, **USP8**[320], **VHL**[177], **VPS28**[43], VPS4B, **WDR7**[317], XPO1
24	CNTF	**APOE**[34], **CNTF**[321], CNTFR, **EIF3E**[177], EXT1, **EXT2**[177], ICA1, IL6R, **IL6ST**[322], KRT40, **LHX2**[323], **LIF**[324], LIFR, **LRPPRC**[324], Pro-inflammatory Cytokine, **SORT1**[325], SOX10, STK16, **TRIP6**[201], **VHL**[177]
25	CNTN4	APLP1, **APP**[326], **BAG3**[327], **BARD1**[170], **BRCA1**[170], CNTN4, COPS5, **GJA1**[328], SETDB1, **WDR77**[329]
26	CNTN6	CHL1, CNTN6, PTPRA, SIRT1
27	CRIM1	**ATXN7**[330], **CACNA1A**[330], CREB1, **CREBBP**[331], CRIM1, EP300, miR-17-5p (and other miRNAs w/seed AAAGUGC)

28	CRYM	C7orf25, **CDC37**[308], **CHMP2B**[317], CREB1, CSHL1, L-triiodothyronine, **MIB1**[332], RNF126, **TERF1**[282], TG, thiomorpholine-carboxylate dehydrogenase
29	CSNK1G3	ACTB, CK1, CORO1C, CSNK1G3, DBNDD2, **ELAVL1**[248], **KLF3**[37], LIMA1, **LRRK2**[333], **NSF**[334], PPP1R14A, RD3, SLC25A41, **SNCA**[335], SYNPO, **TSPAN3**[174], UBXN7
30	CST3	**ALB**[31], **APP**[336], **ATXN1**[159], C4, C4A/C4B, CST1, **CST3**[337], CSTB, CTSB, CTSD, CTSL, HSD17B10, MMP25, PAX3, **PDPK1**[338], RNF115
31	CX3CR1	C-X3-C chemokine receptor, chemokine receptor, CX3CL1, CX3CR1, Gpcr, **NFATC1**[339], **NFATC2**[339], NKX3-1
32	CYP2D6	amitriptyline, atomoxetine, carvedilol, chloropromazine, chlorpheniramine, citalopram, clomipramine, clozapine, codeine, CYB5A, CYP2C9, Cyp2d, CYP2D6, debrisoquine, desipramine, dextromethorphan, doxepin, flecainide, fluoxetine, fluvoxamine, gefitinib, haloperidol, imipramine, maprotiline, metoclopramide, metoprolol, mexiletine, mianserin, morphine, nortriptyline, **NR2F1**[340], paroxetine, POR, quinidine, risperidone, tamoxifen, thioridazine, timolol, tolterodine, tramadol, unspecific monooxygenase
33	DAO	acyclovir, AP1B1, BSN, CEP97, CLINT1, **CRMP1**[341], D-amino-acid oxidase, **DAO**[341], DAOA, **DSP**[341], **DYNC1H1**[341], **DYNLL2**[341], EP300, ERC1, **MAP1B**[341], **MYOD1**[231], **NCOA6**[341], **PABPC1**[341], PC, **PCLO**[341], PEX5, **PFN1**[341], PHYHIP, **PPP1CB**[341], PRKAB2, PRKCG, **RAPGEF4**[341], RASD2, RIDA, **SRCIN1**[341], YLPM1
34	DCTN1	ABCG1, ABRACL, Actin, ACTR10, ACTR1A, ACTR1B, ADGRL2, AGK, AHI1, AKAP9, **AKT1**[308], AKTIP, **ANKLE2**[43], ANKS1B, **ANXA1**[342], **ARF6**[177], ARFIP2, ARHGEF2, ASMTL, AURKB, **BAG3**[784], BBS1, BBS4, BCAS3, BICD1, BICD2, BICDL1, BICDL2, BTF3, BTF3L4, BZW1, CADPS2, **CAMK2A**[343], CAMSAP1, CAMSAP2, CAPZA1, CAPZA2, CAPZB, CASP4, CC2D1A, CCDC124, **CCNB1**[342], **CCT5**[284], **CCT6A**[284], **CDC37**[308], CDK1, **CDK5**[344], **CENPE**[43], **CEP170**[342], CEP170B, CEP250, **CEP290**[345], CEP41, CEP44, CEP76, CEP83, CFAP97, **CHTOP**[342], CIAO1, CKAP2, CKAP2L, CLASP1, CLASP2, **Clip1**[346], **CLIP1**[346], **CLTC**[342], CORO1C, CPVL, **CUL3**[161], CYFIP1, DAZ2, DCDC2, **DCLK1**[342], DCLK2, **DCTN1**[43], Dctn1-**Dync1l2**-Hap1-Hd, **DCTN2**[174], DCTN3, DCTN4, DCTN5, DCTN6, **DERL1**[43], **DISC1**[347], DLGAP5, DNM2, **DST**[348], **Dst**, **DTNBP1**[347], DUOXA1, DVL1, **DYNC1H1**[43], **DYNC1I1**[308], **Dync1i2**[342], **DYNC1I2**[342], **DYNC1LI1**[43], DYNC2LI1, Dynein, **DYNLL1**[349], DYNLRB1, **DYNLT1**[342], EBNA1BP2, **EED**[350], EMC3, EML1, EML4, ENO3, EPB41, **ERLIN2**[342], **ESYT2**[345], FAM83D, FBXL5, FBXW4, FDXR, GAS2L3, GPATCH1, GPX1, **GRB2**[351], GSK3B, GSTK1, GTSE1, **HAP1**[352], HAUS4, HAUS6, HAUS7, HAUS8, Hd-perinuclear inclusions, HDAC6, HK2, **HMMR**[342], HSPA5, **HSPB2**[353], HTR1A, **HTT**[354], **HYOU1**[342], HYPK, IFT140, **IGF2BP2**[342], IPO7, IPO9, IRF3, **KCTD5**[43], **KIAA0368**[355], KIAA0408, KIF11, KIF14, KIF16B, KIF18B, **KIF23**[304], KIF2A, KIF2C, **KIF3A**[356], **KIF5A**[357], **KIF5B**[358], KIT, KLHL2, KNSTRN, LASP1, LGR4, LONP1, LSM14B, LTV1, LUZP1, MAGEA3/MAGEA6, **MAP1LC3B**[342], MAP1S, MAP2, MAP2K3, MAP2K6, MAP7, MAP7D1, MAP7D2, MAP7D3, MAP9, **MAPRE1**[43], MAPRE2, MAPRE3, **MAPT**[359], **MARK2**[342], MED14, MOV10, MRPS12, MTERF1, MTPN, MTUS1, **MYC**[177], MYO19, **MYO1C**[43], NAA10, NAA15, **NACA**[342], **NAP1L1**[342], **NAP1L4**[342], NDC80, **NDEL1**[360], NDN, NDUFA5, NEBL, **NEDD1**[342], NEK2, **NIN**[342], **NINL**[343], NMT1, NNT, NPLOC4, **NTRK1**[172], NUBP2, **NUDC**[358], NXF1, **PAFAH1B1**[343], PAPOLG, PDZD11, **PEX14**[361], PFDN1, PGAM1, PLEKHA5, PLK1, **PPP1CA**[362], PRC1, **PSMA5**[345], PSMB1, PSMD14, PSMD7, **PTDSS1**[43], RAB2A, RAB35, RAB6A, RAB8A, RBL2, RFXANK, SDHC, **SEC16A**[43], Sec23, SEC23A, SEC24B, SEC24D, SHCBP1, SKA1, SKA2, SKA3, SLAIN2, SLK, SLMAP, SMC2, **SMURF1**[363], **SNAP29**[304], **SNW1**[364], SNX27, SNX5, **SNX6**[365], **SOD1**[784], **SORT1**[345], SPAG5, SPC24, **SPTBN1**[366], **SRP9**[342], SRSF2, **STAT1**[367], STK26, SUPT16H, SYNGR3, TANC2, TBCEL, TDRD3, TMEM256, **TP53BP1**[368], TP53RK, TRIM29, TRIM36, TRIP11, TRO, TSFM, TSR1, **TTF2**[342], TTK, **TUBB2A**[284], **TUBB2B**[284], **TUBB4B**[284], TUBB6, tubulin (family), UBC, **UPF1**[342], UQCRC1, UQCRQ, **VHL**[177], **VIM**[369], VTI1B, YTHDC2, YTHDF1, **YTHDF2**[342], ZC2HC1A, ZC3H15, **ZNF512B**[370], ZNF622

35	DIAPH3	ABI1, ANLN, APC, BAIAP2, CCDC183, **CDC42**[371], CENPA, COMTD1, **CTNNB1**[372], DIAPH1, **DIAPH3**[373], **DLG4**[134], **ENO1**[374], ENPP6, **ERG**[375], FNBP1, FNBP1L, HDAC6, **HIPK2**[376], HS2ST1, INF2, **MAPRE1**[377], **MYC**[177], NCKIPSD, **NIN**[342], **NINL**[342], NXF1, **OFD1**[342], PCM1, **PNKD**[16], **RAC1**[371], **RHOA**[371], RHOF, SCN2B, SIRT1, SMAD9, **SNW1**[364], **SPICE1**[342], **SRC**[378], TRIP10, VCP, WWOX, XPO1, YAP1
36	DISC1	ACTG1, **ACTN1**[379], **ACTN2**[380], AGTPBP1, AKAP6, AKAP9, **ANKHD1**/ANKHD1-EIF4EBP3, APLP1, **APP**[381], ARFGEF2, ARHGEF11, ARIH2, ARMCX5-GPRASP2/GPRASP2, **ATF4**[380], **ATF5**[380], ATF7IP, BICD1, BORCS6, CCDC136, CCDC141, CCDC24, CCDC88A, CDC5L, CDK5RAP3, CEP126, **CEP170**[347], **CEP290**[379], CEP57L1, **CEP63**[380], CIT, **CLU**[347], COL4A1, COL6A2, CRNKL1, DCTN1, **DCTN2**[347], **DISC1**, DMD, DMTN, DNAJC7, DPYSL2, DPYSL3, DRD2, **DST**[347], **DTNBP1**[382], **DYNC1H1**[347], **DYNC1I1**[16], **EEF2**[347], EIF3, **EIF3H**[347], EXOC1, **EXOC4**[347], EXOC7, FBXL3, FBXO41, **FEZ1**[383], FRYL, **GNB1**[347], GNPTAB, **GOLGA2**[379], **GRB2**[384], **GRIN1**[385], GRIPAP1, Gsk3, GSK3B, **HERC2**[347], IFT20, **IMMT**[347], ITSN1, KALRN, KANSL1, KATNAL1, KCNQ5, **KIF3A**[347], KIF3C, **KIF5A**[384], **KIF5B**[386], **KIFAP3**[347], **KLC1**[386], **KLC2**[386], MACF1, **MAP1A**[380], **MATR3**[347], MEMO1, MGAT3, MLC1, MLLT10, MPPED1, MVP, MYH7, MYT1L, **NDE1**[111], **NDEL1**[387], **NEFM**[347], NUP160, OLFM1, PACS1, **PAFAH1B1**[347], PCNT, PCNX4, PDE4A, PDE4B, PDE4C, PDE4D, **PGK1**[347], PPFIA4, PPM1E, PPP4R1, **PPP5C**[347], RABGAP1, **RACK1**[386], RAD21, RANBP9, RASSF7, RBSN, ROGDI, SCOC, SH3BP5, SLC12A2, **SMARCE1**[347], SMC2, SMC3, **SNX6**[347], SPARCL1, Spectrin, SPTAN1, **SPTBN1**[347], SPTBN4, SRGAP2, SRGAP3, SRR, **STX18**[347], SYBU, SYNE1, SYNM, TCL1B, TFIP11, TIAM2, TNIK, TNKS, **TNKS2**[388], TNS2, TRAF3IP1, TRIO, TSPOAP1, **TUBB**[347], **TUBB2A**[347], **TUBG1**[380], UTRN, XPNPEP1, **XRN2**[347], YWHAE, **YWHAG**[389], YWHAQ, YWHAZ, ZNF197, ZNF365
37	DOC2B	**DLG4**[134], DOC2A, DOC2B, **DYNLT1**[390], Snare, **STX4**[391], **STXBP1**[392], STXBP3
38	DPP6	CACNA1B, **DNAAF2**[16], DPP6, **HAX1**[16], KCND2, KCNIP1, **NOVA1**[306], **PHB**[315], PRND, PRNP, **ZFYVE27**[393]
39	DYNC1H1	1-palmitoyl-2-oleoylglycero-3-phosphoglycerol, ACTR1A, **ADRB2**[394], AP3D1, **APOA1**[394], **ARF6**[177], **ARRB2**[395], ATP6V1C1, BI-167107, BICD2, BICDL1, BICDL2, BMI1, **BRCA1**[170], C20orf24, **CALM1**[396] (includes others), CALML3, carazolol, CCDC8, **CCND1**[397], **CCT5**[284], **CCT6A**[284], CDC5L, CDK1, CDK2, **CENPE**[43], **CFTR**[398], **CHMP2B**[317], cholic acid, **CKAP5**[399], **CLIP1**[43], CNTRL, COPS5, **CREBBP**[43], **CRY2**[400], **CSF1**[401], **CSNK2B**[402], **CUL3**[161], CUL7, **CYFIP2**[403], Cytoplasmic Dynein, **DAO**[341], DCTN1, **DCTN2**[43], DCTN4, DCTN5, **DISC1**[347], DMXL2, **DYNC1H1**[399], **DYNC1I1**[16], **Dync1i2**[399], **DYNC1I2**[43], **DYNC1LI1**[43], DYNC1LI2, **DYNLL1**[43], DYNLRB1, **DYNLT1**[342], DYNLT3, **EED**[350], **EEF1A1**[404], **EEF1D**[315], **EGFR**[405], EPB41, FAM46A, **FBXW11**[406], FN1, FOLR1, FOXB1, FOXL1, **FOXP3**[165], **GABARAPL2**[177], GLTSCR1, **GRB2**[407], HDAC6, **HNRNPD**[43], HSP90AA1, HSPA1A/HSPA1B, **HTT**[302], IDH3G, IFT140, IKBKAP, **IKBKG**[408], ITGA4, KATNA1, KATNB1, L-alpha-palmitoyloleolyl-phosphatidylcholine, **LRRK2**, MAGED1, MAP2K5, MAPK3, **MAPRE1**[43], **MATR3**[409], **MAX**[315], MCM2, METTL21A, METTL21C, **MIB1**[332], MKLN1, **MTNR1B**[305], **MYC**[410], **MYH10**[404], **MYH9**[404], **NCKAP1**[409], **NDE1**[267], **NDEL1**[411], NF1, **NIN**[342], **NINL**[342], NME2, NPHP1, **NRP1**[404], NSUN2, **NTRK1**[172], **NUDC**[259], **NUMA1**[412], OBSL1, **OFD1**[342], OTOF, **PAFAH1B1**[43], PAFAH1B2, **PEX14**[361], **PFKL**[409], **PFKP**[409], **PHB2**[177], PHLDA3, POC5, POU5F1, **PPP1R12A**[399], **PPP2R3C**[43], **PPP4C**[177], **PRKCD**[413], PRKCG, PSEN2, RNF2, **RPGRIP1L**[342], RPRD2, RUFY1, RYK, **SCLT1**[342], SIRT1, **SIRT7**[414], **SKI**[415], **SMAD2**[370], **SMARCA4**[416], **SNCA**[43], **SNRPB**[177], **SPRTN**[417], **SUZ12**[350], **SYNCRIP**[43], TNIK, TOR1A, TPD52, TRAF6, TRIM58, **TSC22D1**[177], **TUBB2A**[284], **TUBB2B**[284], **TUBB4B**[284], TUBB6, **TUBG1**[399], UBC, **UNK**[388], USP7, **VCAM1**[418], **WDR7**[409], **YWHAG**[22], YWHAQ, YWHAZ, ZNF16
40	EFEMP1	**AHR**[419], ARAF, **ATN1**[159], **ATXN7**[330], **BAG6**[201], **CACNA1A**[330], **CALR**[420], **CANX**[420], COL15A1, Collagen type XVIII, DUX4, EFEMP1, ELN, **ENO1**[374], **GFI1B**[159], GMNN, **HSF1**[421], **HSP90B1**[420], HSPA5, **KHDRBS1**[422], **KLF4**[423], MOV10, **MYC**[423], NOS3, NXF1, **PDIA3**[424], POU5F1, PROP1, **RAF1**[425], RERE, RIC8A, Rnr, SGTA, **SHMT2**[374], **SMAD4**, **SMAD7**, **SOCS6**[422], SOX1, **SOX2**[423], SOX3, TFAP2A, **TFAP2C**[426], TIMP3, **TNIP1**[422], **TRAF2**[201], **TRIM28**[427], TXNDC5, **UBQLN1**[428]

41	ELP3	**ALB**, ANLN, BACH1, CIAO1, CTDP1, **DCP2**[429], ELP2, ELP3, ELP4, ELP5, ELP6, FAM219B, FAM96B, FGFR1OP, FOXP1, FOXP2, **FOXP3**[430], FOXP4, **HIST1H4A**[431], HIST2H3C, **HNF4A**[18], IFI30, IKBKAP, IP6K3, KIF18A, KPTN, **MMS19**[249], MPG, **NANOG**[432], NFKBIA, **NTRK1**[172], **P4HA1**[345], PARD6A, PCGF1, PCGF6, **PCNA**[433], **POLR2A**[434], POU5F1, **PPP2R1A**[43], PRPS1, **PSMA5**[345], PSMB4, QPCT, **RACGAP1**[43], RASSF1, RNA polymerase II, **SKAP1**[174], SMC6, **SMURF1**[435], SOGA1, USP11
42	EPHA4	**ABL1**[436], Akt, ANAPC2, ARHGEF15, **BACE1**[437], CEACAM21, Chn1, CHN1, Efna, EFNA1, EFNA2, EFNA3, EFNA4, EFNA5, EFNB, **EFNB2**[438], EFNB3 , Eph Receptor, EPHA, EPHA2, **EPHA4**[439], EPHB2, Fgfr, **FGFR1**[440], **FYN**[441], FZR1, GNAT2, GNAZ, GPN3, **GRB10**[442], **GRB2**[442], HLA-DPA1, **HOXA13**[443], HSP90AA1, **HSP90AB1**[444], **IKBKG**[445], **ITGB3**[446], JAK2, KIDINS220, **L1CAM**[359], LYN, Mapk, MSX1, MSX2, NGEF, **NOTCH1**[447], p85 (pik3r), PLC gamma, POU3F4, PTPRB, PTPRH, PTPRJ, **PTPRO**[448], **RBPJ**[447], RIPK3, RYK, SH2D3C, **SRC**[442], TBC1D22A, **TSPAN3**[174], **TUBB2B**[16], VAPB, VAV2, **VHL**[449], ZIC2
43	ERBB4	**ABL1**[450], **ABL2**[450], ADAM17, afatinib, **AKT1**[451], ANKS1A, ANKS1B, BMS-599626, BTC, BYSL, CADM1, **CAV3**[452], CBFA2T3, CBL, **CD44**[452], **CFL1**[453], **CRK**[454], **CRKL**[454], DCN, **DLG1**[455], DLG2, DLG3, **DLG4**[198], EDNRA, **EGFR**[456], **EGFR** ligand, ERBB, Erbb1-Erbb4 dimer, **ERBB2**[457], **ERBB3**[458], Erbb3-Erbb4 dimer, **ERBB4**[459], Erbb4 dimer, ERBB4 ligand, ErbB4(s80)-YAP1, Erbb4/p120 dimer, Erbb4/p80 dimer, EREG, ESR1, EZH2, **FYN**[460], GAPDH, **GRB2**[454], **GRB7**[461], **GRIN1**[462], GRM5, **HBEGF**[452], HDAC6, **HIF1A**[463], HSP90AA1, **HSP90AB1**[444], ITCH, JNJ-26483327, **LRIG1**[464], **MAP3K14**[461], **MAPK1**[465], MAPK3, **MDM2**[466], MUC1, N-cor, **NCK1**[454], **NCK2**[454], **NCOR1**[467], **NEDD4**[468], NRG (family), NRG1, NRG2, NRG3, NRG4, NUDCD1, osimertinib, p85 (pik3r), pelitinib, phosphatidylinositol-4,5-bisphosphate 3-kinase, PI3K (complex), PI3K p85, **PIAS3**[469], PIAS4, PICK1, **PIK3R1**[452], **PIK3R2**[349], pirotinib, PLC gamma, poziotinib, PPP1R12C, PSEN1, **PTK2B**[355], **PTPN11**[454], **RASA1**[450], **RNF41**[470], Secretase gamma, Shc, SHC1, SNTA1, SNTB2, **STAT5A**[471], STAT5a/b, **STAT5B**, SYK, TAB2, TFAP2A, **TFAP2C**[459], **TP53**[466], **TRIM28**[466], TRO, **UBB**[472], **USP8**[473], WWOX, WWP1, YAP1
44	EWSR1	ABTB1, ANKRA2, ANKRD1, ANKRD49, ANKRD7, ASB6, ASH1L, ATG2A, **BRCA1**[474], **CEBPA**[475], CERS2, CERS5, CIITA, DEDD, Ewsr1, FLI1, HDAC10, JARID2, KDM5D, KLHL12, KRT5, MED20, MTF2, NAA15, NUDT18, OTX2, PER1, PHF1, PMS1, POLR1A, POLR2B, POLR3F, POU5F1, **PPARGC1A**[476], PWP1, RAI14, **REST**[477], RFXAP, RORC, SALL4, SIRT2, SMARCA1, SNAPC4, TLE6, TRIP4, TRPS1, UBP1, YWHAE, ZBTB24, ZDHHC6, ZFP42, ZFP82, ZNF180, ZNF226, ZNF281, ZNF292, ZSWIM4
45	FEZF2	BCL11B, BHLHE22, HDAC1, **HNF4A**[18], RNF20, SLC17A7, TBR1
46	FGGY	EAF1, FGGY, **NSFL1C**[16]
47	FIG4	CMAS, FIG4, **HNF4A**[18], MYO18A, phosphatase, phosphatidylinositol 3,5-diphosphate, phosphatidylinositol 4,5-diphosphate, phosphatidylinositol-3,4,5-triphosphate, phosphatidylinositol-3-phosphatase, PIKFYVE, TWISTNB, VAC14
48	FUS	HPRT1, HSP90AA1, **HSP90AB1**[478], **HSP90B1**[478], **HSPA2**[395], HSPA5, HSPA8, **HSPA9**[478], HSPB1, **HSPD1**[478], **ICAM1**[479], IGKV1-5, IL7R, **ILF2**[199], ILF3, ITGA4, ITK, JUN, KHDRBS3, **KIF22**[43], KLHL40, **KLHL5**[478], **KPNA2**[478], **KPNB1**[478], **LDHA**[478], LDHB, **LMNA**[478], LRRIQ4, LSM12, LUZP4, LYAR, LYZ, MAGEB2, **MAP3K3**[478], **MAPK13**[480], MAT2A, **MATR3**[43], **MAX**[315], MCM2, **MCM4**[199], MDH1, MDH2, **MECP2**[481], MFGE8, mir-132, mir-192, mir-199, mir-9, MTAP, MTHFD1, MTSS1L, **MYH10**[478], **NANOG**[482], **NAP1L1**[478], NCAPG2, **NCL**[199], **NCOA6**[478], **NEDD8**[161], **NEFH**[483], **NKD2**[422], NONO, NPM1, NSDHL, **NTRK1**[172], **NUDT21**[478], NUPR1, OAT, OBSL1, OTUB1, **PA2G4**[484], PAICS, PARK2, **PARP1**[199], **PCBP2**[478], PCM1, **PCNA**[478], **PDIA3**[478], PDS5A, **PFKL**[478], **PFN1**[478], **PGK1**[478], PGM1, **PHB**[478], **PHGDH**[478], **PIK3R2**[407], PIP, **PKM**[478], PLCE1, PLEC, PM20D2, poly(ADP-ribose), poly(U)RNA, **POU4F1**[485], POU4F2, **PPIA**[478], PPIL4, **PRDX1**[478], PRKCB, **PRMT1**[478], PSMB7, PSMD12, PSPC1, PTBP2, PYCR1, PYCRL, PYM1, RAB7A, RAD54B, **RAN**[478], RAVER1, **RBBP4**[478], **RBMX**[422], RELA, RNA polymerase II, **RPA1**[279], **RPA2**[279], **RPA3**[279], **RPL11**[478], RPL12, RPL14, RPL18, RPL37, RPL4, **RPL9**[478], RPLP0, **RPLP1**[483], RPN2, **RPS14**[478], **RPS5**[478], **RPS6KB2**[486], **RPS8**[478], **RPS9**[478], **RTCB**[478], **RXRA**[487], SAE1,

		**SAFB2**[422], SARNP, SEC13, **SERBP1**[478], SF1, **SF3A1**[478], **SF3A2**[488], SF3A3, SF3B3, SF3B4, **SFPQ**[478], SHOC2, SHROOM3, **SIK2**[489], **SIRT7**[414], **SLC1A5**[478], SLC22A11, SLC25A1, SLC25A13, **SLC25A3**[478], **SLC25A5**[478], **SNRPD2**[478], **SNW1**[364], SPATA6, SPI1, SQSTM1, **SRPK1**[489], SRRM1, **SRRM2**[395], SRSF10, SRSF2, SRSF9, SSBP1, **ST13**[478], STATH, SUV39H1, **SUZ12**[350], **SYNCRIP**[478], **TAF15**[422], **TARDBP**[490], TBPL1, TCF7L2, **TCP1**[478], TDRD3, THRA, **TIMM50**[478], TKT, TLN1, **TNIP1**[422], **TOP1**[199], **TP63**[491], **TP73**[478], Transportin, **TRIM28**[199], TRIP4, TRPM4, TTI2, TUBA1A, **TUBA1B**[478], **TUBA1C**[483], **TUBA4A**[395], **TUBB**[478], TUFM, TXNL4B, UACA, UBA1, UBAP2, UBAP2L, UBC, **UBE2I**[484], **UBL4A**[492], **UFL1**[478], USF2, **VCAM1**[418], **VCL**[493], VCP, WBP4, **WWOX**[178], **XRN2**[199], **YBX1**[494], **YBX3**[395], YEATS4, YPEL1, YWHAE, YWHAQ, YWHAZ, **ZMYM2**[495]
49	GARS	**ATF4**[496], Bis(5'-nucleosyl)-tetraphosphatase (asymmetrical), CDK2, CELSR2, **CRY1**[400], **CRY2**[400], CUL5, **DCP2**[429], **EED**[350], **EEF1D**[497], **ELF3**[177], **FBF1**[342], FBXO6, **GARS**[498], glycine-tRNA ligase, HDAC6, **HNF1A**[18], HSPB1, **HSPB2**[353], IARS, **IKBKE**[177], MAP3K10, **MAPK8**[422], NPHP1, **NTRK1**[172], **OFD1**[342], **PARK7**[499], POC5, **PSMA3**[500], PSMC3, **RPGRIP1L**[342], **SPICE1**[342], **STAU1**[501], **SUMO3**[193], TCTN1, **TCTN3**[342], TNRC6B, TRAF6, UBC, **UNK**[502], YWHAZ
50	GLE1	**ABHD5**[248], CARNMT1, CCDC8, EIF3F, **ELAVL1**[248], GLE1, **HIST1H1A**[16], IER2, **IFI16**[503], **KPNB1**[43], KRT10, **KTN1**[504], MEX3C, **NUP107**[43], NUP153, NUP155, NUP35, **NUP98**[43], NUPL2, RGPD4 (includes others), RNF2, SEH1L, **UBE2I**[43], **UXT**[505]
51	GRB14	ARCN1, ATIC, CDC5L, CHFR, CNGA1, DAPK3, **EGFR**[506], **ERBB2**[356], **FGFR1**[507], FRS3, **GRB14**[508], GSK3B, **HACD3**[1043], HCK, IGF1R, **INSR**[509], IRS1, **MAP3K14**[356], MET, **NFKB1**[315], NRL, PDGFRB, **PDPK1**[510], PLVAP, **PRKCZ**[511], SQSTM1, STK26, TEK, **TNKS2**[512]
52	GRN	**ACACA**[425], ACTG1, ADAMTS7, AHCYL2, ARFGAP1, **ATN1**[159], **ATXN7**[330], **CACNA1A**[330], CCDC33, CCDC8, CCNG1, CCNT1, CD68, CDK2, **CDK9**[513], CELA2B, **CFTR**[398], CLEC4M, COMP, COX6B1, **CRKL**[514], **CRY1**[422], **CSNK2B**[425], CTSO, CTTN, CUL7, DCP1B, DLK1, DLX2, **EED**[350], **EGFL7**[16], **EGFR**[515], ELANE, FAM131C, FAM207A, FANCL, FBXO6, FLYWCH2, FRAT1, **GFI1B**[159], **GLRX3**[201], GNB2, **GRIA1**[516], **GRIA2**[516], **GRIA3**[516], GRN, **HECW2**[517], HK3, HOXA1, HSP90AA1, **HSP90AB1**[425], **HSPG2**[518], IGFBP4, KCNQ2, KRT18, **KRTAP10-7**[201], KRTAP26-1, **LEP**[519], **MAPK1**[425], MASTL, mir-103, NAPSA, NF2, NFKBIA, NLK, **NOTCH1**[520], NPM1, **NTRK1**[172], NXF1, OBFC1, OTUD5, OTX1, P-TEFb, **PDX1**[521], PIAS2, **PIK3R2**[422], PKP2, **POT1**[422], **PPP2CA**[174], **PRKAA1**[522], PRKAB2, PRTN3, **PTGDS**[519], **RAC1**[422], **RPS6KA1**[523], RPS6KA2, RPS6KA3, SAV1, SGTA, SIRT3, SLPI, SMAD9, **SORT1**[524], SPACA4, SYTL1, TAT, **TGM2**[422], TLE3, TLK2, TLR9, Tnf receptor, **TNFRSF1A**[525], TNFRSF1B, TOP3B, **TRIB3**[526], **VHL**[449], **YY1**[422], ZC3HC1, ZFP41
53	HEXA	**ABL1**[527], **BAG6**[43] beta-L-N-acetylhexosaminidase, **CRK**[527], FAN1, FBXO6, **FYN**[527], GM2A, **H2AFX**[528], HEXA, HEXB, hexosaminidase, **HNF4A**[18], **HNRNPD**[43], **IQCB1**[529], SGTB, **SYNCRIP**[43] , TUBA3E, USP22, YPEL5
54	HFE	B2M, HFE, HFE2, NKX2-3, SYVN1, TF, TFR2, **TFRC**[530]
55	HNRNPA1	**EED**[350], **EEF1A1**[486], EEF1B2, **EEF1G**[486], **EEF2**[486], **EIF2AK2**[531], **EIF2B1**[486], EIF2B3, **EIF3H**[486], **EIF4A2**[486], EIF4A3, EIF4E, **ELAVL1**[486], **ENO1**[486], EPPK1, **ERG**[532], ESR1, EWSR1, **EXOSC4**[43], **FAM120A**[486], FAM120C, FAM35A, FAM98B, FARS2, FASTKD2, FBXO6, **FBXW11**[533], FEN1, FIP1L1, FLG, FLG2, FLNA, **FMR1**[534], FN1, FRMD4A, **FUBP3**[43], **FUS**[486], **G3BP1**[486], **G3BP2**[486], **GADD45A**[486], GADD45GIP1, **GAN**[535], GAPDH, GDE1, GM2A, GPKOW, GSN, **GSTP1**[486], GTF2A1, **GTF2E2**[536], **GTF2F1**[43], **GTF2I**[486], H1FX, **H2AFX**[528], H3F3A/H3F3B, HAL, **HBB**[537], **HDAC5**[538], **HIPK3**[539], HIST1H1E, HIST1H2AB, **HIST3H3**[540], **HMGA1**[264], **HNRNPA0**[43], HNRNPA1, HNRNPA1L2, HNRNPA2B1, **HNRNPA3**[43], **Hnrnpa3**[486], HNRNPAB, **HNRNPC**[486], HNRNPCL1/HNRNPCL2, **HNRNPD**[43], **HNRNPDL**[43], **HNRNPF**[486], **HNRNPH1**[486], **HNRNPH2**[486], **HNRNPH3**[486], HNRNPK, **HNRNPL**[486], **HNRNPM**[486], **HNRNPR**[486], **HNRNPU**[486], **HNRNPUL1**[486], **HNRNPUL2**[486], HNRPA1-HNRPA2B1-POT1-TERF1-TERF2, HRAS, HRNR, **HSP90AB1**[486], HSPA1A/HSPA1B, **HSPA2**[536], HSPA5, HSPA8, **HSPA9**[536], HSPB1, **HSPD1**[486], **ICAM1**[541], **IFIT2**[542], **IFIT3**[542], IGF2BP1, **IGF2BP2**[486], IGF2BP3, **IGSF8**[541], IL36G, **ILF2**[43],

		ILF3, ITGA4, IVL, IVNS1ABP, JUN, **JUNB**[43], **JUP**[486], **KHDRBS1**[43], **KHDRBS2**[536], KHSRP, **KIAA0368**[536], **KIF4A**[543], KIN, KMT2E, KPRP, KRT1, KRT10, KRT14, KRT15, KRT16, KRT17, KRT2, KRT33A, KRT34, KRT5, KRT6A, KRT6B, KRT6C, KRT78, KRT79, KRT80, KRT83, KRT9, KSR1, LAMP1, LARP1, LARP1B, LARP7, LCN2, **LDHA**[486], LGALS7/LGALS7B, **LIG4**[170], **LMNA**[486], **LMNB1**[544], **LRPPRC**[486], LTV1, MAEL, **MAG**[545], MAGOH, MALSU1, **MAP3K14**[408], **MAPK13**[480], **MARK2**[486], **MATR3**[43], **MAX**[546], MCAT, MCM2, **MCM5**[547], MEF2A, **MEPCE**[174], **MIB1**[332], MKNK1, MKNK2, MOV10, MRPL1, MRPL10, MRPL11, MRPL13, MRPL15, MRPL16, MRPL18, MRPL19, MRPL21, MRPL22, MRPL24, MRPL27, MRPL28, MRPL3, MRPL32, **MRPL37**[486], MRPL38, MRPL39, MRPL4, MRPL43, MRPL44, MRPL45, MRPL46, MRPL47, **MRPL48**[486], MRPL52, MRPL57, MRPL58, MRPL9, MRPS10, MRPS15, MRPS18A, MRPS18B, MRPS18C, MRPS2, MRPS22, MRPS23, MRPS24, MRPS27, MRPS28, MRPS30, MRPS31, MRPS35, MRPS5, MRPS7, MRPS9, MTERF3, MX1, **MYC**[548], **MYH9**[486], MYL12A, NAMPT, NBEAL1, **NCBP1**[544], NCCRP1, **NCL**[486], **NCOA5**[43], **NEDD1**[549], **NEDD8**[161], **NEK4**[153], **NFATC1**[544], **NFATC2**[544], NFKBIA, NGRN, **NKRF**[544], NNT, NONO, **NOP56**[550], NOS2, **NR0B2**[422], **NR3C1**[867], NSUN5, **NTRK1**[342], **NUDT21**[544], NUP205, NXF1, OBSL1, **P4HB**[486], **PABPC1**[486], PABPC4, PAIP2, PAIP2B, **PAN2**[551], PARD6B, PARK2, **PARK7**[499], **PCBP2**[486], PDCD11, PDCD4, **PFN1**[486], **PGK1**[486], **PHB**[439], **PHB2**[486], PHYHIP, PICALM, PIP, **PKM**[486], **PKP1**[486], PLOD1, PLOD2, PLOD3, PNN, PNO1, PNP, POF1B, POLDIP3, **POLR2A**[444], poly(ADP-ribose), **PPIA**[486], PPIL1, PPL, **PPP2R1A**[486], **PRDX1**[486], PRKDC, **PRMT1**[486], PRMT2, PRMT3, **PRMT5**[319], PRMT8, **PRPF19**[486], PRPF31, **PRPF4**[544], PRPF8, **PRRC2A**[552], PRSS3, **PSMA3**[500], PSMB4, PSMD9, **PTBP1**[486], PTCD1, PTCD3, PUF60, **PURA**[536], quercetin, **RACK1**[486], **RAD50**[544], RALY, RBM12, **RBM12B**[43], **RBM14**[43], **RBM26**[544], **RBM3**[43], RBM39, **RBM6**[43], RBM7, RBM8A, **RBMS1**[16], **RBMX**[43], RBMX2, RIPK3, **RNF14**[553] , RNF2, RNPS1, ROCK1, RPA1, **RPA2**[279], **RPA3**[279], RPAP1, **RPL21**[29], **RPL26**[174], RPS15, **RPS27A**[16], **RPS3A**[16], **RPS6KB2**[486], RPS7, RPUSD3, RRAGA, RRP1B, **RTCB**[486], **SAFB**[544], **SAFB2**[43], SAP18, SAP25, SART3, SCAI, **SEC16A**[43], SEPT2, SEPT6, **SERBP1**[534], **SF3A2**[488], **SFPQ**[544], SKIL, **SMAD6**[554], **SMN1**/SMN2, **SMURF1**[363], SNIP1, SNRNP200, **SNRPD1**[544], **SNRPD2**[544], **SNRPD3**[544], **SNW1**[364], SREK1, SRP72, **SRPK1**[523], SRPK2, **SRRM2**[555], **SRRT**[544], **SRSF1**[544], SRSF4, SRSF6, SRSF7, SSB, **STAU1**[501], **STRAP**[486], STRBP, **SUGP2**[544], **SUMO3**[193], SUPV3L1, **SYNCRIP**[43], SYTL2, **TAF15**[43], **TARDBP**[544], TFEC, THAP12, **THRAP3**[556], **TNF**[557], **TNFRSF10B**[558], **TNK1**[43], TNPO1, **TOP1**[559], TOPORS, **TP53**[549], TRA2B, Transportin, TRUB2, **TTF2**[543], TUBGCP3, U2af, **UBQLN1**[560], **UBQLN2**[560], UBR1, **UBR2**[536], **UPF1**[16], UPF3A, UPF3B, **VCAM1**[418], VCP, VDAC1, VDAC2, VDAC3, **VHL**[449], WDR33, **WDR77**[304], WDR82, **XAB2**[544], **XIAP**[561], XPO1, **XRCC5**[544], XRCC6, XRN1, **XRN2**[544], YARS2, **YBX1**[562], **YBX3**[486], YLPM1, YTHDC1, **YTHDF2**[536], YWHAE, YWHAQ, YWHAZ, ZBP1, **ZBTB7A**[563], ZCCHC3, **ZFR**[16], ZNF207, **ZNF326**[43], ZNF638, ZWINT
56	HNRNPA2B1	ADARB1, **ADRB2**[564], **AHR**[565], **AHSA1**[566], **AKT1**[567], **ANK2**[260], AP2A1, AP2M1, APOBEC3B, ARHGEF5, **ARRB2**[395], **ASB2**[568], **ATG101**[260], **ATG13**[260], **ATG16L1**[260], ATG3, **ATG5**[260], ATG7, **ATXN2L**[483], **AURKA**[549], BAG2, **BRCA1**[569], **C9orf72**[260], **CALR**[260], CAND1, **CD4**[570], **CD81**[541], CDC5L, **CDK9**[536], **CDKN1A**[549], **CDKN2A**[571], CEP57, **CKAP5**[572], COPS5, **COPS6**[161], **CSF2**[573], **CSNK2A1**[574], **CSNK2B**[574], **CTNNB1**[575], CUL1, **CUL2**[161], **CUL3**[161], **CUL4A**[161], **CUL4B**[161], CUL5, CUL7, **DCUN1D1**[161], DEAF1, **DHX9**[576], DMRT2, **DYNLL1**[483], **DYNLL2**[483], **EDC4**[483], **EED**[350], **EGFR**[471], **EMC2**[43], **ERBB3**[577], **ERG**[532], ERRFI1, ESR1, **FBXO32**[578], FBXO6, **FBXW11**[533], FN1, **FUS**[483], FYCO1, **GABARAP**[260], **GABARAPL2**[260], **GAN**[260], **GRB2**[577], **H2AFX**[528], **HAUS1**[549], **HDAC5**[538], HDGF, **HIPK3**[539], HIST1H2BA, **HIST3H3**[540], HNRNPA1, HNRNPA2B1, **Hnrnpa3**[43], **HNRNPD**[43], **HNRNPH1**[536], **HNRNPH2**[43], **HNRNPL**[579], **HNRNPU**[43], HNRPA1-HNRPA2B1-POT1-TERF1-TERF2, **HSBP1**[260], HSPA5, HSPB1, **HUWE1**[580], **ICAM1**[541], **IFIT2**[542], **IFIT3**[542], **IGSF8**[541], **IQCB1**[529], **ISG15**[168], ITGA4, JUN, **JUNB**[43], **KHDRBS2**[536], **KIF4A**[543], KMT2E, **KPNA2**[594], KRT82, KRT85, LACRT, **LIG4**[170], **LMNA**[581], MAEL, **MAP1A**[260], **MAP1B**[260], **MAP1LC3A**[260], **MAP1LC3B**[260], MAP1LC3B2, **MAP1LC3C**[260], MAP4K2, **MATR3**[43], **MAX**[315], MBNL1, **MBP**[582], MCM2, **MCM5**[547], **MEPCE**[536], MGMT, MKRN1, MMGT1, MRPL13, **MYC**[583], NDUFB5, **NEDD1**[549], **NEDD4**[260], **NEDD8**[161], **NEFH**[483], **NEK4**[153], NEK9, **NFS1**[260], NFX1, NONO, **NR4A1**[308], **NTRK1**[172], NXF1, OBSL1, ONECUT1, **PA2G4**[584], **PAN2**[551], PARK2, PHF6, PIP,

		PLK1, poly(ADP-ribose), **POT1**[585], **PPP1CB**[43], **PPP1CC**[43], **PPP2CA**[586], PRMT6, PRNP, **PSMA3**[500], PTPN12, RALY, RASSF5, **RB1CC1**[260], **RBM3**[587], RIBC2, RMST, **RPA1**[279], **RPA2**[279], **RPA3**[279], RPL12, RPL35, RPLP0, **RPLP1**[483], **RPS6KB2**[486], **S100A8**[471], S100A9, **SET**[452], **SF3A1**[260], **SF3A2**[488], SHC1, **SMCR8**[260], **SMN1**[588]/SMN2, **SMURF1**[363], **SNCA**[589], snRNP, **SNW1**[364], SPATA5, SQSTM1, SREK1, SRP72, **SRRM2**[555], **SRSF1**[43], **STAU1**[501], **STK4**[260], **SUMO3**[193], **TAF15**[43], **TARDBP**[590], TBC1D32, **TCEB1**[260], **TERT**[591], TFCP2, **TFEB**[592], **TNF**[593], **TOB1**[594], **TOP1**[559], **TP53**[549], **TTF2**[543], TUBA1A, **TUBA1C**[483], TXNDC16, **UBA5**[260], UBASH3B, UBC, **UBL4A**[492], **ULK1**[260], **VCAM1**[418], **VHL**[579], WDR41, WDR62, XRCC3, YAE1D1, **YBX1**[595], YWHAE, YWHAQ, ZFP36, ZMAT3, **ZMYM2**[495], ZNF207
57	ITPR2	1,4,5-IP3, AHCYL1, BANK1, BBOX1, BCL2, BOK, CA8, CACNG2, CAPZA2, CCDC8, CHGA, **CHGB**[310], CIB1, FBXL14, **HERC2**[596], ITPR, **ITPR1**[597], **ITPR2**[598], LIMA1, LPAR1, MECOM, mir-133, **MYH9**[43], **MYO1C**[43], **NFATC1**[599], **NUP107**[43], NXF1, P2RY12, PSMC3, **SUMO3**[43], TESPA1, TRPC1, TRPC3, TRPC4, UBC
58	KDR	AAR2, ACP1, AEE 788, AGTR2, **AIMP2**[308], Akt, **AKT1**[600], altiratinib, anlotinib, **ANXA5**[601], apatinib, axitinib, BMS-690514, BMX, brivanib, brivanib alaninate, BSG, BTRC, cabozantinib, **CAV1**[602], CBL, CD36, **CD44**[603], CD63, **CDC42**[604], **CDH1**[604], CDH5, cediranib, CEP 7055, CHIR-265, **COL18A1**[605], CP-547632, CREB1, **CRK**[606], CSF2RB, CSNK1D, **CTNNB1**[607], CUL1, CXCR1, CXCR2, CXXC5, CYC 116, DCN, **E2F1**[608], **EEA1**[609], **EGR2**[610], EHF, EP300, **EPAS1**[611], EPN1, ERK, ERK1/2, ETV2, F2RL2, famitinib, **FBXO25**[612], **FBXW11**[308], FIGF, FLNB, FLT1, FLT4, Focal adhesion kinase, foretinib, FOXC2, **FOXM1**[613], fruquintinib, fucoidin, GAB1, Gata, **GATA1**[614], GATA2, GATC, golvatinib, **GRB10**[615], **GRB2**[616], GREM1, growth factor receptor, **GTF2I**[617], GTF2IRD1, HAND1, HDAC1, heparan sulfate, heparin, HEY1, HHEX, Histone h3, HSPB6, **HSPG2**[618], IGF1R, Integrin, **IQGAP1**[619], isoliquiritigenin, ITGA2, ITGA5, **ITGAV**[620], **ITGB1**[621], **ITGB3**[622], JAK inhibitor I, JI 101, **KDR**[623], **KDR** dimer, KL, **KLF2**[519], **KLF4**[624], lenvatinib, **LGALS1**[625], LGALS3, lucitanib, MAPK3, MCAM, MET, MGCD-265, MGCD516, motesanib, MYB, MYOF, **NANOG**[626], NAXD, **NCK1**[616], **NCOA1**[627], **NCOA3**[627], **NCOA4**[43], Neuropilin, nintedanib, NOS3, NPHS1, NPM1, **NR2F2**[628], **NR3C2** [629], **NRP1**[630], NRP2, orantinib, OSI-930, P110, p85 (pik3r), PALLD, pazopanib, **PDCD6**[631], PDCL3, Pdgfr, pegaptanib, pegdinetanib, pexmetinib, PF 00337210, PGF, PI3K (complex), **PKM**[632], PLC gamma, **PLCG1**[633], PLXNA1, PLXNA4, **PRKCZ**[604], PROX1, **PTK2**[634], PTPN1, **PTPN11**[635], PTPN6, PTPRB, PTPRH, PTPRJ, **PVR**[636], RAB5A, RALGDS, ramucirumab, **RASA1**[637], RB1, rebastinib, regorafenib, Rhox4b (includes others), SAA, SAR103168, **SCARB1**[638], semaxinib, **SH2D2A**[639], SHB, SHC1, SHC2, sorafenib, Sos, SOX17, **SOX2**[640], **SP1**[641], **SP3**[642], **SPIB**[51], **SRC**[619], SRF, **STAT1**[643], **STAT3**[644], sulfatinib, sunitinib, SYK, TAK 593, telatinib, tesevatinib, TIMP3, tivozanib, **TJP1**[645], TMEM204, tofacitinib, TRPC1, TXNIP, vandetanib, vatalanib, Vegf, VEGF inhibitor drug, Vegf Receptor, VEGFA, VEGFC, VEGFR2-targeted Contrast Agent BR55, XL820, XL999, **YWHAG**[43], **YY1**[646], ZCCHC6, ZFPM2, ZSCAN21
59	KIFAP3	AIM1, **Ank2**[647], APC, **AR**[104], BUD13, **CDH2**[481], **CEP170**[343], **CEP19**[342], **CEP290**[345], CEP350, chloropromazine, COL4A1, **CTNNB1**[648], **CTNND1**[648], DDX23, **DHX15**[43], **DISC1**[347], DNAJC9, DNPEP, EFR3A, FAM192A, GBE1, GCC2, guanosine 5'-O-(3-thiotriphosphate), HMGXB4, HSDL2, IFI30, **IQGAP3**[343], **KIF3A**[43], KIF3B, KIF3C, **KIFAP3**[16], **KPNA2**[43], MAP3K10, MAP3K11, **MED24**[43], MEST, MRPS17, MRPS25, MUC13, NAA10, NCF2, NIPSNAP3A, **NTRK1**[172], **NUDT21**[308], ORC3, **OSBPL1A**[43], PEX19, PRPF3, **PRPF4**[43], **PSMC5**[267], PSMD5, RANBP2, RAP1GDS1, **RBFOX1**[159], RBM22, **RBM26**[43], RHOG, RUSC2, SART1, SLC9A3R2, SMC3, SNAP47, SON, SPTAN1, **SRC**[16], SSNA1, **TAF1**[43], **TAF4**[43], TAF5, **TAF7**[43], **VHL**[649], **XAB2**[649], XRCC4, ZNF821
60	LIF	**CNTF**[247], CRH, **CSF1**[650], CTF1, GP130-LIFR, **HSF1**[651], HSF4, IGF2R, **IL6ST**[652], **IRF8**[653], JUN, **LHX2**[323], **LIF**[253], LIFR, **LRPPRC**[247], NFKBIA, NFKBIE, Pro-inflammatory Cytokine, **RBPJ**[654], **SORT1**[248], **STAT3**[655], **STAT6**[656], **TP53**[657]

61	LIPC	ACTB, acylglycerol lipase, ADHFE1, **APOB**[658], **CALR**[659], **CANX**[659], CNPY2, **EEF1A1**[659], EEF1A2, FKBP9, GANAB, GAPDH, HDL, heparin, HMGCR, **HNF4A**[660], **HSP90B1**[486], HSPA5, HSPA8, lipase, LIPC, LMF1, **LRP1**[661], lysophospholipase, NOMO1 (includes others), **NR1H4**[662], **NR3C1**[663], **NR4A1**[44], **PDIA3**[659], PDIA6, phospholipase A1, **PML**[45], PPIB, **RCN1**[659], **RPL11**[659], SERPINH1, **SLC25A3**[659], **SLC25A4**[659], **SLC25A5**[659], SSR4, TOR1B, triacylglycerol lipase, TUBA1A, **TUBB4A**[659], UGGT1, **ZNF202**[55]
62	LMNB1	ABCE1, ABCG8, **ADRB2**[564], AIRE, ANPEP, **ATF4**[422], ATG12, **ATM**[304], **BCAR3**[177], C17orf49, CASP3, CASP6, CCDC8, **CCNA2**[664], **CCNB1**[664], **CCND1**[397], CDK1, CDK2, **CDK5**[665], **CEBPA**[666], **CUL3**[667], **CUL4A**[304], CUL7, DAG1, DCAF6, DCAF8, **DDB1**[304], **DDX5**[668], **EED**[350], EGF, **ELAVL1**[248], **EMD**[669], FBXO6, FN1, GIT2, **GJA1**[670], **HECW2**[517], HNRNPA1, **HNRNPF**[43], **ILK**[489], ITGA4, KPNA1, Lamin, Lamin b, LEMD3, **LMNA**[671], **LMNB1**[672], LMNB2, **LRRK2**[673], MBIP, MCM2, MELK, **MIB1**[332], MSH6, **MYBBP1A**[668], **NANOG**[674], **NCSTN**[43], **NDEL1**[675], NES, **NTRK1**[172], OBSL1, PARK2, **PARP1**[668], PASK, PCGF2, PDE6D, PIAS2, **PLCB1**[276], PLCB2, PLEC, POU2F1, **PRKCA**[676], PRKCB, PRNP, **RAC1**[677], RNF13, RSL1D1, RTCA, SEPT12, SERPINA3, SMARCAD1, SMC1A, SMC4, SPAG4, **SPRTN**[417], SPTAN1, SRPK2, SUN1, SUN2, **TFEB**[592], TMEM63B, **TMPO**[43], TOP2A, UBC, URB2, **VCAM1**[418], **VIM**[678], YWHAQ, YWHAZ, **YY1**[668], **ZBTB16**[667], ZWINT
63	LOX	APAF1, BMP1, **BTK**[338], CBL, CD2AP, **COL1A1**[679], COL1A2, COL3A1, Collagen(s), CSH1/CSH2, DHDDS, **EFEMP2**[680], **EGFL7**[681], ELN, **EPAS1**[682], FBLN5, FBN1, FN1, **FOS**[164], **FOXM1**[83], GATAD2B, **HIF1A**[682], **HIST1H1A**[683], HIST2H2BE, Histone H1, **HNRNPK**[684], HSPA1A/HSPA1B, **Hspa1b**[685], **HTT**[686], **IQCB1**[529], **LEP**[687], LOX, **MDC1**[688], MEMO1, MKS1, MMP25, **MYC**[689], NFIA, NFIB, **NOTCH1**[690], Nuclear factor 1, **PA2G4**[688], **PCBP1**[684], **PTPRK**[501], **RAF1**[691], **SH3KBP1**[692], SNAI1, SOS2, **SOX2**[693], **TFAP2C**[426], TLL1, TLL2, **TP53**[694], tranylcypromine
64	LUM	**ACAN**[695], CD14, Collagen(s), E. coli lipopolysaccharide, GNAQ, IGF2BP1, ITGAL, ITGAM, ITGB2, LGALS9, LUM, MMP14, [696], RELA, SMAD3, **TAF4**[245], TRA2B
65	MAOB	1-methyl-4-phenyl-1,2,3,6-tetrahydropyridine, 1-methyl-4-phenylpyridinium, 2-phenethylamine, 5-hydroxytryptamine, adenine-riboflavin dinucleotide, benzphetamine, benzylamine, dextroamphetamine, **DLG4**[134], dopamine, **EGR1**[554], **ESRRA**[697], HOXD10, **HSPB2**[353], hydrogen peroxide, isocarboxazid, JUN, **KLF11**[698], ladostigil, MAO, MAOB, methamphetamine, NFYB, pargyline, **PDX1**[521], phenelzine, phentermine, **PPARGC1A**[697], procainamide, rasagiline, safinamide, selegiline, **SP1**[699], **SP3**[699], **SRPK1**[422], tranylcypromine
66	MAPT	14-3-3, AATF, **ABL1**[700], **ABL2**[701], **AK1**[702], AK2, **AKT1**[703], Alpha tubulin, aluminum chloride, ANXA2, **APOE**[45], **APP**[704], ARVCF, **BAG1**[705], **BAG6**[43], BIN1, **CALM1**[706] (includes others), **CAMK2A**[707], CAPN2, CASP1, CASP3, CASP6, **CASP7**[708], **CASP8**[708], **CCNB1**[709], **CDC37**[710], CDK1, CDK2, **CDK5**[711], **CDK5R1**[711], **CHEK1**[712], **CHEK2**[712], cinnamaldehyde, **CKAP5**[43], **CRYAB**[713], **CSNK1A1**[714], CSNK1D, **CSNK2A1**[37], DAPK1, **DCTN1**[342], DGUOK, **DNAAF2**[16], DNAJB1, DNMBP, DYRK1A, DYRK2, EFHD2, **EGFR**[210], **EIF2AK2**[715], ELAVL4, EP300, EPM2A, EWSR1, F Actin, F2, FGR, **FKBP4**[716], **FYN**[535], GAPDH, **GAR1**[43], **GRB2**[717], GRIN2B, Gsk3, GSK3A, GSK3B, **GTF2E2**[43], **HAX1**[16], HDAC6, heparan sulfate proteoglycan, heparin, **HMGA1**[421], **HSF1**[322], Hsp27, Hsp70, Hsp90, **HSP90AB1**[718], HSPA1A/HSPA1B, **HSPA4**[719], HSPA8, **ILK**[720], **INSR**[721], **KAT5**[722], KATNA1, **KIF5B**[723], **KIFC3**[43], **KLC1**[723], **KLC2**[723], LIMS1, **LRRK2**[724], **MAP1A**[725], MAP2, MAP3K1, **MAPK1**[726], MAPK10, MAPK11, MAPK12, **MAPK13**[727], MAPK14, MAPK3, **MAPK8**[727], **MAPT**[704], MARCH7, MARK1, **MARK2**[728], MARK3, MARK4, MOAP1, **MTOR**[729], Muscarinic cholinergic receptor, myricetin, NKX2-1, NUB1, NUFIP1, OGT, **P4HB**[730], PACSIN1, PARK2, PASK, PDIA2, **PDX1**[521], PEG10, **PIN1**[731], Pka, **PKN1**[732], **PLCG1**[717], **PLCG2**[717], **PPP1CA**[733], Ppp2c, **PPP2R2A**[734], **PPP3CA**[735], **PPP5C**[736], **PRKACA**[736], PRKAR1A, PRNP, PSEN1, **PSMC2**[737], **PTK2B**[707], PTPA, RBM4, **RELN**[738], **RPS6KA1**[707], RPS6KA3, RPS6KA5, RPS6KB1, S100B, SCGN, **SCLT1**[342], **SFN**[714], **SGK1**[707], SIRT1, **SLC1A2**[711], SLC6A8, **SNCA**[739], Spectrin, **SPICE1**[342], SQSTM1, **SRC**[740], SRC (family), **SRPK1**[741], SRPK2, SRSF2, **STAU1**[742], STH, **STUB1**[743], **STXBP1**[744],

		SUMO, SYK, **SYNCRIP**[43], SYVN1, TAOK1, TAOK2, **TFEB**[43], TIGD6, TNIK, TPI1, TRAF6, TTBK1, TTLL6, TUBA1A, **TUBA1B**[745], **TUBA4A**[746], **TUBB3**[745], tubulin (complex), tubulin (family), UBASH3B, **UBB**[703], UBC, UBE2D2, UBE2W, Ubiquitin, USP43, VCP, **WWOX**[747], **YWHAB**[748], YWHAE, **YWHAG**[714], YWHAH, YWHAQ, YWHAZ
67	MATR3	ACTA1, **ADAR**[43], **AGO1**[562], **AGO2**[562], **AIMP2**[308], **AKAP8**[43], AKAP8L, ALKBH3, **ALYREF**[43], APOBEC3B, ASH2L, **ATM**[749], ATP6V1C1, **AURKA**[549], B9D2, CACNA1D, CACNA1E, CACNB4, **CALM1**[750] (includes others), CAND1, CCDC8, CDC5L, CDK2, **CDK9**[536], **CEBPA**[666], CELF6, **CENPE**[43], CEP250, **CIRBP**[751], **COPA**[43], COPS5, **CRY1**[400], **CRY2**[400], CUL1, **CUL3**[161], CUL7, CXorf23, **CYFIP2**[323], DAZAP1, **DDX17**[43], DDX28, **DDX3X**[43], **DDX5**[43], **DHX9**[43], **DISC1**[347], DMXL2, DNAJB6, DPY30, **EED**[350], **EGFR**[405], EIF2AK4, EIF3D, **EIF4A2**[752], EIF4A3, Elf5, **ERG**[532], ESCO2, EWSR1, **FAM120A**[43], FAM208A, **FAM98A**[406], **FBXW11**[315], FN1, **FOS**[315], **FUBP3**[43], **FUS**[43], **GFI1B**[159], GSG2, GSTK1, **H2AFX**[528], **HNRNPA0**[43], HNRNPA1, HNRNPA2B1, **Hnrnpa3**[43], **HNRNPA3**[43], **HNRNPC**[43], **HNRNPDL**[43], **HNRNPF**[43], **HNRNPH1**[43], **HNRNPH2**[43], **HNRNPH3**[43], **HNRNPK**[201], **HNRNPL**[43], **HNRNPM**[43], **HNRNPR**[43], **HNRNPUL1**[43], **HNRNPUL2**[43], IDH3G, **IFIT2**[542], **ILF2**[43], ILF3, ITGA4, KDM6A, **KHDRBS1**[43], **KIF4A**[543], KMT2D, **KPNA2**[753], LGR4, MAGOH, **MAP1LC3A**[754], **MAP3K3**[408], **MATR3**[201], **MCM5**[547], **MEPCE**[536], MKRN1, MPHOSPH8, **MYC**[177], **NAP1L4**[43, 409], **NCOA5**[43], **NCOA6**[755], **NEDD8**[161], **NEK4**[153], **NFKB2**[408], NFX1, NLRP5, NONO, **NOVA1**[756], NOVA2, **NR4A1**[315], **NTRK1**[172], **NUMA1**[43], OBSL1, PAGR1, **PAN2**[551], PARK2, PAXIP1, **PCBP1**[159], **PFKL**[409], **PFKP**[409], PKP2, PKP3, PKP4, POLR3B, POU5F1, **PPHLN1**[43], **PPP1CA**[43], **PPP1CB**[43], PRKCE, PRKCG, PRR3, **PTBP1**[43], PTBP2, PTBP3, PUM1, RALY, RASD1, **RBBP5**[755], **RBFOX2**[43], **RBM12B**[43], **RBM14**[43], RBM15B, **RBM3**[43], RBM4B, **RBM6**[43], **RBMX**[43], RBMXL1, **RCN1**[43], RELA, RNF2, **RPA1**[279], **RPA2**[279], **RPA3**[279], **RTCB**[43], **RUVBL2**[536], **SAFB**[43], **SAFB2**[43], SENP3, **SET**[43], SIRT6, SLTM, **SMAD6**[751], SMARCAD1, SNAP23, SNRPA, SQRDL, SRPK2, SRSF9, **STAU1**[501], STAU2, STK24, **SUGP2**[43], **SUMO3**[193], **TAF15**[43], **TARDBP**[590], **TFEB**[592], TIMM13, TNFRSF21, **TNK1**[43], **TTF2**[543], **TUBA1C**[516], **TUBB4A**[516], UBC, **UBE2I**[757], **UNK**[502], WDR5, **WDR7**[409], **WWOX**[178], **YBX1**[399], YLPM1, YWHAZ, **YY1**[758], ZNF106, **ZNF326**[43], ZNF335, ZNF638, ZNF668
68	MT-ND2	**GRIN1**[759], **HNRNPK**[760], Mitochondrial complex 1, MT-ND2, NADH dehydrogenase, NDUFA11, **SNCA**[589], **SRC**[759], STK11, TIMM21, TIMMDC1, YME1L1
69	NAIP	APAF1, CASP3, **CASP7**[761], **CASP9**[762], Ciap, **COMMD1**[763], **DIABLO**[762], **HECW2**[517], HPCA, **MAP3K7**[764], NAIP, NLRC4, NLRC4 Inflammasome, POU5F1, TAB1, XAF1
70	NEFH	ASAH1, **ATN1**[198], **ATXN2L**[483], BAG2, C16orf70, CCDC51, **CDK5**[765], **CDK5R1**[766], **DST**[767], **Dst**[606], DUSP7, [483], **DYNLL2**[483], **EDC4**[483], **FADD**[174], **FUS**[483], **GAN**[535], **GATA1**[768], GLYR1, **GRB2**[577], GSK3B, HDAC1, HNRNPA2B1, HSD17B6, **HTT**[198], **INA**[16], JUN, **KCNMA1**[14], **MAP3K5**[769], **MAPK1**[770], **MECP2**[771], **NDEL1**[360], **NEFH**[772], **NEFL**[16], **NEFM**[16], **Nefm**[12], **NEK4**[153], OTOF, **PIN1**[773], PIP, **PKN1**[774], **PPARGC1A**[775], **PRPH**[776], **PURA**[777], RCOR2, **REST**[771], RFFL, RPL12, RPLP0, **RPLP1**[483], **SIN3A**[771], TUBA1A, **TUBA1C**[483], **TUBB3**[778], tubulin (family), **VHL**[779], ZFP36L2
71	NEK1	ATRX, BRAP, C21orf2, **CENPE**[43], **CEP104**[342], **CEP290**[342], **CSN2**[780], CTNNAL1, **DCAF7**[267], DPM3, **EXOSC4**[781], **FEZ1**[782], FEZ2, **KCTD5**[267], **KIF3A**[782], **LRRK1**[311], **LRRK2**[311], MRE11A, **NEK1**[782], **NEK5**[343], PP2A, **PPP2R1A**[267], PPP2R5A, **PPP2R5D**[782], **RAF1**[267], **RPGRIP1L**[343], **SKAP1**[174], **SPATA7**[343, 783], **TEKT4**[16], TOP1MT, **TP53BP1**[782], TSC2, **VHL**[784], **VPS26B**[16], VTN, WDR83, WWTR1, XPO1, **XRCC5**[784], **YWHAH**[782], YWHAQ, ZNF350
72	NETO1	**BAG3**[327], BCL11B, GRIK1, GRIK2, GRIK5, **GRIN1**[785], **HDAC4**[786], VSX1
73	NIPA1	NO INTERACTIONS
74	NT5C1A	5'-nucleotidase, NT5C1A, **WDYHV1**[201]
75	OGG1	APEX1, AXIN1, **CHGB**[29], **CTNNB1**[787], ERCC6, **ERCC8**[788], LAMB1, MUTYH, NFYA, **OGG1**[399], **PARP1**[789], **PPARA**[277], **PRKCA**[790], RAD52, **SNRPF**[177], **SP1**[791], **TP53**[792], **XPC**[793], XRCC1
76	OMA1	**EPAS1**[682], IFNA8, OMA1, SLC39A4, TOR3A

77	OPTN	AKAP6, **CALM1**[750] (includes others), CDC23, **CDKN1A**[794], **CENPB**[315], **CLTC**[213], CMYA5, COL11A2, **CYLD**[795], **DAZAP2**[201], **DST**[213], DYSF, DZIP1, EBNA1BP2, EIF4G2, **EIF5A2**[796], EPS15, FAM175B, FAM189A2, FBXW5, **FOS**[315], FTH1, **GABARAP**[797], **GABARAPL1**[797], **GABARAPL2**[797], GDP, GNAS, GRM1, GRM5, guanosine triphosphate, HACE1, **HSPA9**[315], HSPB1, **HTT**[798], IKBKB, KANSL1L, **KCTD17**[796], LNX2, MACF1, **MAP1LC3A**[797], **MAP1LC3B**[797], **MAP1LC3C**[799], mGLUR Group I, **MPP1**[796], MPP6, MYH3, **MYO6**[799], NDUFAF2, NEB, NRL, **OPTN**[201], PDHA1, PIAS4, PKN2, **POLR1C**[800], **PPARG**[315], RAB10, Rab11, RAB11A, RAB11B, RAB12, **RAB14**[801], RAB25, RAB3IL1, RAB8A, RIPK1, **RNF11**[370], RPL37A, RTN3, SEMA4A, SLF2, SLMAP, **SNX6**[213], **SOD1**[802], SQSTM1, **STX12**[16], SURF2, SYNGAP1, **TAX1BP1**[794], TBC1D15, TBC1D17, TBK1, **TFRC**[795], **TNF**[803], **TNFRSF1A**[803], **TNIP1**[213], TRADD, TRAF3, TTN, **UBB**[213], UBC, Ubiquitin, **UBXN1**[800], UQCRQ, **USP12**[800], VCP, VWF, WDR20, WDR48, **YWHAB**[796], **YWHAG**[796], **YWHAH**[796], YWHAQ, YWHAZ, ZDHHC17, ZMAT2, ZNF181, ZNF302, ZNF329, ZNF384, ZNF398, ZNF426, ZNF670, ZNF711
78	PARK7	3,4-dihydroxybenzaldehyde, **ABL1**[804], ACLY, ACTA1, ACTC1, **AR**[116], ARHGDIA, **ATP5B**[649], BABAM1, **BAG1**[805], BAG2, **Basp1**[499], **BAX**[806], BBS1, BCL2L1, **CALR**[499], **CANX**[499], CASP3, **CASP8**[807], CAT, CCDC130, **CCT3**[499], **CCT4**[499], **CFL1**[499], CHD2, **CHD4**[808], CLCF1, **CLTA**[499], **CLTC**[499], COPB2, CSN1S1, Cu+, Cu2+, CXCL8, CYCS, DAXX, **DLG4**[403], DPYSL2, EDF1, **EEF1A1**[499], **EEF2**[809], EFCAB6, **EIF2S3**[499], EPB41L5, ERK1/2, ETF1, **FADD**[807], FAU, **FOXP3**[165], **GARS**[499], GATAD2B, glutamylcysteine synthetase gamma, glutathione peroxidase, GOPC, **GTF2F1**[499], **GTF2I**[810], HDAC1, **HDAC2**[808], Hg2+, **HIF1A**[649], HIPK1, **Hmgb1**[499], HNRNPA1, **HNRNPK**[499], Hsp70, **HSP90B1**[499], **HSPA4**[805], **HSPA9**[499], **HSPD1**[499], **ICAM1**[811], **IQGAP1**[812], **KCNMA1**[14], **KDM5A**[812], KIF1B, LRRC59, Manf, **MAP1B**[422], **MAP3K5**[813], **MBD3**[808], MCM2, MDH1, Mn2+, MT-ND1, **MTA1**[808], **MTA2**[808], MTERF1, MTRF1, Myl6l, **NCL**[499], NDUFA4, NDUFS3, NFKBIA, NONO, NOX4, NPM1, **NUDT21**[499], NXF1, OSCP1, OTUD7B, PARK2, **PARK7**[814], PARP, PDAP1, **PHB**[499], PIAS2, **PIAS3**[815], PIAS4, PINK1, **PML**[816], **PPP1R12A**[641], **PPP2R1A**[817], **PPP2R1B**[817], PREX1, **PTBP1**[641], **PTEN**[818], PYCR1, **RACK1**[819], RB1, **RBBP4**[808], **RBBP7**[808], RPL17, RPL23, **Rpl23a**[499], RPL24, **RPL26**[499], RPL27, Rpl32, RPL4, Rpl8 (includes others), RPS10, RPS20, **RPS25**[499], **RPS3**[499], **RPS5**[499], **RPS6**[499], RREB1, SEPT11, SEPT2, SEPT7, **SFPQ**[820], SLC18A2, **SNCA**[821], **SOD1**[822], **SOD2**[823], SREBF2, **STAT1**[824], **STUB1**[814], SUCLG1, SUMO, **SUMO1**[825], SYP, TCEA1, **TCF3**[826], **TDP2**[827], TNFRSF10A, **TP53**[816], Tpi1 (includes others), Tpm3, TRAF6, TUBA1A, **Tuba1b**[499], **TUBB**[404], **TXN**[809], UBA2, **UBE2I**[825], **UBR5**[43], **UNK**[502], UQCRHL, USP19, **VAMP2**[828], VCP, **VHL**[829], YARS, **YWHAG**[499], YWHAZ
79	PCP4	AIRE, ATOH1, Ca2+, **CALM1** [830](includes others), Calmodulin, **HOXA10**[35], HOXA2, **HTT**[831], **IFI16**[832], **IQCB1**[529], **JRK**[36], LHX1, PCP4, **POT1**[282], RNASEH1, **TERF1**[282], **TERF2IP**[282]
80	PFN1	Abcg3, ACTA1, ACTB, Actin, adenosine triphosphate, **AFDN**[833], AGR2, AKAP12, ANXA13, ANXA4, APBB1IP, ASB1, ASB12, **ASB2**[568], **ATF2**[834], Beta Tubulin, BLVRA, C11orf68, C14orf1, CAND1, CBR1, **Cdc42**[835], CDK2, Ces1e, **CFL1**[835], Clathrin, **CLTC**[836], **CRMP1**[29], CSNK1E, **CTNNB1**[837], CTSZ, CTTN, **CUL3**[161], CUL7, **DAO**[341], DIAPH1, DLG5, **DNM1**[838], DSTN, Dynamin, **EED**[350], **ELAVL1**[248], ENAH, ESR1, **EZR**[835], **FLOT1**[43], FMNL1, FN1, **FOS**[839], **FUS**[478], G-Actin, **GJA1**[670], GLRB, GPHN, GRID2IP, HNRNPA1, Hsp70, **Hspa1b**[836], **HTT**[840], INPP5J, **ISG15**[168], ITGA4, LGALS2, LGALS4, **LIG4**[170], LOC290704, **LRIF1**[29], **MAPK13**[480], MAPK3, MAST3, MCM2, Mg2+, MYPOP, **NCK1**[841], **NTRK1**[172], OBSL1, PALLD, **PCLO**[842], PFN, **PFN1**[843], **PFN2**[838], phosphatidylinositol 4,5-diphosphate, poly-L-proline, PP1-C, **PPIA**[653], Profilin, **RAD51**[844], RAD52, RHOQ, **RIF1**[24], SLC33A1, SLC9A3R1, **SMN1**[845]/SMN2, **SRPK1**[523], **STAT6**[338], **STUB1**[846], **SUMO3**[193], **TLE1**[29], **TMSB10**[847]/TMSB4X, **TP53**[848], TRIM15, **TUBB**[595], UCHL5, **UNC119**[29], VASP, **VCAM1**[418], **VCL**[493], VCP, VIPR1, WAS, WASF1, Wasl, WASL, XPO6, YWHAQ, YWHAZ, ZNF16
81	PLEKHG5	ACTG1, AMOT, AMOTL1, AMOTL2, CSPG4, GIPC1, LIN7C, **LNX1**[849], LNX2, MPDZ, MPP5, **MYO1C**[850], **MYO6**[851], PATJ, PLEKHG5, **RHOA**[851], RND1, RND2, RND3

82	PON1	**APOA1**[852], aryldialkylphosphatase, arylesterase, **CCL2**[853], **CLU**[854], FOXA2, GHRL, HDL, **HNF4A**[18], LDL, malondialdehyde, naringenin, NKX2-1, **NR1H4**[46], ONECUT1, PON1
83	PON2	ABCA2, **ADRB2**[564], AMIGO1, ARSK, aryldialkylphosphatase, arylesterase, ATP2A2, ATP6AP2, B3GNT2, B4GALT1, B4GALT3, C1QL4, CACNA2D1, CACNA2D2, CDC6, CHST10, CHST12, CHSY1, CTSF, **CUL3**[161], **EGFR**[16], ENPP1, ENTPD6, **ERBB3**[577], FBXO6, GPATCH8, **GRB2**[422], HSPA5, HYAL2, IMPAD1, **INSR**[16], ITGA4, ITGA6, KCNK1, **Ktn1**[43], LINGO1, LMLN, LRFN3, **LRIG1**[16], LRIG2, LRP11, M6PR, MAGEA10, miR-199a-3p (and other miRNAs w/seed CAGUAGU), MOXD1, MR1, NETO2, **NUP98**[43], NXPH4, PCNT, PGAP1, POGLUT1, POMGNT2, POMT2, **PPP2R5D**[16], PTP4A2, RDH11, RNF130, RNF167, RNF170, **RUVBL2**[177], SAR1B, **SCARB1**[16], SIL1, **SLC39A11**[16], SMOC1, SPPL2B, ST3GAL6, ST6GALNAC3, STRN3, SULF2, **TCF3**[826], TMEM219, TMEM30A, **TUBG1**[43], TXNDC15, **UBR2**[16], UGT8
84	PON3	arylesterase, dihydrocoumarin hydrolase, ESR1, HDL, PON3
85	PRPH	1-methyl-4-phenylpyridinium, AP-3, BAPTA-AM, calpeptin, **CCND1**[397], **CUL3**[161], **Dst**[855], **FBXW7**[856], **GAN**[535], GFAP, **GJA1**[670], **IL6**[857], iono**myc**in, **ISL1**[858], **KCNMA1**[14], KRT15, **LNX1**[859], MAPK3, **MYC**[860], MYO5A, **NEFH**[776], **NEFL**[776], **NEFM**[776], **NR3C1**[861], PD 150606, **POU4F1**[858], PPP2R2B, **PRPH**[862], sphingosine-1-phosphate, **VIM**
86	PSEN1	ACHE, ACTB, **ACTN1**[33], Alph1, Alpha catenin, AP1M2, APBA1, APBA2, APBA3, APH-1, APH1A, APH1B, **APOE**[33], **APP**[259], ARC, **ATF2**[863], **ATF4**[864], ATP2A2, ATP6V0A1, **ATP6V0D1**[865], **ATP6V1A**[865], ATP6V1D, ATP6V1E1, ATP6V1G2, **BACE1**[259], **BCL2**[866], BCL2L1, BIRC2, BIRC3, **CALR**[867], CASP3, caspase, **CAV3**[868], **CD44**[869], CD63, **CDC37**[33], CDC42BPB, **CDH1**[870], CDH10, CDH11, CDH18, **CDH2**[871], CDH6, CDH8, **CDK5**[33], **CFL1**[33], CFLAR, CIB1, CLSTN1, CLSTN2, **CLTA**[865], CLTB, **CLTC**[865], CREB1, **CSN2**[872], CTNNA1, CTNNA2, CTNNA3, **CTNNB1**[873], **CTNND1**[874], CTNND2, CYP2C18, CYP2C8, **DLG4**[875], DLL1, DNM2, DOCK3, E2012-BPyne, ECSIT, EFHD1, EFNB1, **EFNB2**[876], EIF4G1, ENSA, **EPB41L3**[33], **ERBB4**[877], Erbb4 dimer, Erbb4/p80 dimer, **ERLIN2**[878], ERN1, ester, ETFA, **ETS1**[879], ETS2, F2RL1, FBXL12, **FBXW7**[880], FGF13, FKBP8, FLNA, FLNB, **FLOT2**[881], GAPDH, GCDH, GDI1, GDI2, GFAP, GNAO1, GOLGA5, **GRB2**[882], **GRIA3**[883], **GRIN1**[884], GRIN2A, GSAP, GSK3B, **HBEGF**[885], **HERPUD1**[886], HIGD1A, HM13, **HMGB1**[33], HSPA1A/HSPA1B, HSPA8, **HTRA2**[887], ICAM5, **IGSF8**[865], **IRAK2**[888], **ITGB1**[889], ITPR, **ITSN2**[33], JAGN1, **JUP**[890], KANK2, KCNIP3, KCNIP4, **KLF3**[37], L-685,458, L-685,458-BPyne, **L1CAM**[865], **LRP1**[891], LRP8, **MAG**[865], MAOA, Mapk, **MAPT**[892], **MBP**[865], MTCH1, NAPG, NCAM1, NCAM2, **NCSTN**[893], NEUROG3, NFASC, NGFR, NOS3, Notch, **NOTCH1**[894], NOTCH2, NOTCH3, NOTCH4, NPR1, NPTX1, OXCT1, PDCD4, PDZD2, pep11-Bt, pepstatin, phosphatidylinositol, **PIK3R1**[895], PKP4, PLD1, PLP1, PLP2, PRAM1, PRDX2, Presenilin, **PRKACA**[896], PSEN1, PSEN2, PSENEN, **PSMA5**[897], PSMB1, PSMD1, PTGER4, RAB3A, **RAD23A**[33], RER1, RHEB, **RMDN3**[33], RNF32, RPL10, **RPS27A**[865], RYR3, SCAMP1, SCAMP2, SCAMP3, SCAMP4, SCN1A, SEC61A1, Secretase gamma, SH3GL1, SH3GL3, SLC16A1, SNAP25, **ST13**[33], STAMBPL1, STT3B, **STX1A**[898], **STX1B**[865], STX5, **STXBP1**[865], SV2A, SV2B, Syntaxin, SYT1, SYT12, SYT2, tarenflurbil, TCF4, TCF7L2, **TDP2**[422], **TFG**[865], TGFBR1, THEM6, TMED10, **TMED2**[899], **TP53**[879], TRAF6, **TSPAN3**[865], **TUBA1B**[33], **TUBA1C**[865], **TUBB**[865], **TUBB2A**[865], TYR, **UBQLN1**[900], UMPS, **UNK**[502], **VAMP2**[865], VAMP3, VASN, **XIAP**[901], YME1L1
87	PVR	**AFDN**[902], AP1M2, CD226, CD96, **CDH1**[903], **DYNLT1**[904], **ELAVL1**[248], **GLI1**[905], GLI3, ITGA6, **ITGAV**[906], **ITGB3**[906], **ITGB4**[907], **KDR**[636], **KRTAP10-3**[201], **KRTAP10-7**[201], KRTAP10-8, **KRTAP10-9**[201], **LOC100996763**[160]/NOTCH2NL[201], **NECTIN3**[908], NRF1, PLA2G3, **PVR**[373], SLC30A2, TIGIT, **TJP1**[902], VTN, **WNK1**[422]
88	RAMP3	ADM, CALCR, CALCRL, Gpcr, HSPB3, IAPP, **KRTAP10-3**[201], KRTAP5-9, **LOC100996763**[160]/NOTCH2NL[201], **NR4A1**[909], **NSF**[910], **PDCD6IP**[24], pramlintide, RAMP3, SLC31A2, SLC9A3R1

89	RBMS1	**ERG**[911], EWSR1, **FHL3**[16], GRK5, **HIST1H1A**[16], HNRNPA1, **LMNA**[912], mir-383, **MYC**[913], POLA1, **RBFOX2**[16], SF1, TFCP2, XPO1
90	RNASE2	ABCE1, CEBPE, FOXA2, Gi-coupled receptor, heparan sulfate proteoglycan, heparin, **HNF4A**[18], **KLF2**[914], RNase A, RNASE2, **RNH1**[915], Tlr, TLR2, YWHAQ,
91	RNF19A	C11orf58, **CASR**[916], **ILK**[308], **MAP3K7**[308], **NCOA3**[917], NFKBIA, **NUDT21**[308], **PIAS1**[917], PSMC3, RPS6KA5, SNCAIP, **SOD1**[918], **SP1**[919], SUFU, UBE2L3, UBE2L6, VCP, ZAK
92	SARM1	**ATP1A3**[920], ATP1B2, CARNMT1, CCDC47, CDIPT, CLGN, GHITM, **HYOU1**[796], **IRAK1**[796], **IRAK2**[796], **MAPK8**[921], **MYD88**[922], NFXL1, NLRX1, **NTRK1**[172], PINK1, RNF2, RS1, SARM1, SDC2, STT3A, TICAM1, TICAM2, Tlr, TOMM20, **TRAF2**[923], TRAF6, UBC, **UXT**[924]
93	SCN7A	**DLG1**[925], ERBIN, GIPC1, **ISL1**[858], **LNX1**[925], LRRC7, NKX2-1, NOS1, **POU4F1**[858], **RAPGEF2**[925], riluzole, SCN7A, SCNN1B, SNTB1, **TJP1**[925], voltage-gated sodium channel
94	SELL	Alpha Actinin, **ANXA1**[926], ARNT, **CALM1** [927](includes others), Calmodulin, carbohydrate, cardiolipin, CD34, CEBPE, cerebroside 3-sulfate, CFH, chondroitin sulfate B, chondroitin sulfate E,**COL18A1**[928], dextran sulfate, ELF4, EMCN, **EZR**[929], **FOXO1**[930], **FOXP3**[931], fucoidin, fucose, ganglioside GM3, Glycam1, glycosaminoglycan, **GRB2**[932], H32, heparan sulfate, heparin, **ID2**[222], **ID3**[222], **KLF2**[933], **KLF3**[934], lipopolysaccharide, MADCAM1, **MSN**[929], MUC7, Mucin, **NFATC1**[935], oligosaccharide, phospholipid, PLAUR, PNAD, PODXL, PODXL2, **PRKCA**[936], PRKCI, PRKCQ, PRKG1, **SATB1**[937], SELE, Selectin, SELL, SELP, SELPLG, sialyl 6-sulfo Lewis X, sialyl lewis X, SOX4, **SRC**[938] (family), sulfatides, SYK, VCAN
95	SEMA6A	EVL, ITM2B, **ITSN2**[939], **NCK2**[16], ONECUT1, PLXNA4, **PTCH1**[16], SEMA6, SEMA6A, **SFPQ**[940], SH3RF1, **SMAD2**[315], SORBS1, SORBS2, **TMEM17**[342], **TMEM216**[342], **TP53**[941], TRPS1, **UBE2I**[942]
96	SETX	ABI1, AEBP2, **ALB**[31], **APOA1**[31], **BRCA1**[943], C17orf96, CABIN1, **CARD8**[174], CCDC15, CDC5L, CHD3, **CLU**[43], CNTROB, COMTD1, **EED**[944], **ESR2**[945], EXOSC9, EZH1, EZH2, **FLOT2**[43], **HDAC5**[345], **HIPK2**[946], **ISLR**[16], JARID2, **KAT5**[947], KIF7, **KPNA2**[753], LCN15, MAPKAPK3, MEF2A, MEF2C, MEF2D, **MLST8**[174], MORC3, MTF2, NHSL1, **PIAS1**[946], **PNKD**[16], **POLR2A**[948], POLR2F, POLR2G, **PTEN**[949], **RBBP4**[731], **RBBP7**[731], REV3L, RPRD1B, **SETX**[946], **SKAP1**[174], **SMN1**[948]/SMN2, STK38, **SUMO1**[946], **SUMO2**[950], SUPT5H, **SUZ12**[731], **TDG**[946], TOPORS, TRIM35, TTLL7, UBA2, UBC, **UBE2I**[946], XYLT1, ZCWPW1
97	SIGMAR1	(+)-SKF10047, 3-methoxyphencyclidine, 4-methoxyphencyclidine, **ANK2**[951], BD 1063, bicifadine, **CFTR**[398], cholesterol, CLN3, cocaine, CRHR1, cyproheptadine, dehydroepiandrosterone sulfate, dehydroisoandrosterone, dextromethorphan, dihydrocodeine, ELMOD1, ELMOD2, ELMOD3, etorphine, fluvoxamine, haloperidol, HCRTR1, HSPA5, hydromorphone, IL24, ITPR3, KCNA2, KCNA3, KCNH2, MOV10, N,N-dimethylsphingosine, N-ethyl-1-(3-methoxyphenyl)cyclohexanamine, naloxone, naltrexone, NXF1, opioid, opioid receptor, opipramol, OPR, oxycodone, PDZD11, pentazocine, progesterone, SA 4503, SCN5A, SIGMAR1, sphinganine, SR 31747, steroid, **TCTN3**[342]
98	SLC1A2	ACAT1, Adaptor protein 2, AJUBA, **AK1**[952], Ant, AP2A1, AP2B1, ARF3, **ARF6**[952], ATP1A1, **ATP1A3**[952], ATP1B1, ATP5C1, ATP5F1, **ATP6V1A**[655], BDH1, **C3**[952], CAND1, CASP3, **CCT3**[952], **CCT4**[952], **CCT8**[952], CLOCK, CNP, **CREBBP**[953], **CTNNB1**[954], **Dclk1**[952], **DDX5**[952], DLAT, **DLG1**[955], **DLG4**[403], **DNM1**[952], DNM1L, Dynamin, EHD3, GAPDH, GNAO1, **GRB2**[367], **HADHA**[952], HDAC1, **HDAC3**[956], HDAC6, **HDAC7**[956], **HK1**[952], **HOXA13**[957], HSPA12A, HSPA8, **HTT**[302], IDH3A, IDH3B, **INA**[952], K+, L-glutamic acid, **MAP2K1**[952], **MAPT**[711], MTDH, **MYCN**[958], Na+, NDUFS1, **NEFL**[665], NFKBIA, NPEPPS, **NSF**[665], OPA1, PC, PDHB, PFK, PFKM, **PFKP**[665], **PGK1**[665], **PHB**[665], **PHB2**[665], **PKM**[665], PLP1, **PML**[959], **PPP3CA**[739], PYGB, RELA, riluzole, **RORA**[960], RORC, **RPL11**[665], **RPL22**[665], RPL23, **RPS14**[665], RPS16, Rps3a1, RTN1, SFXN3, **SLC1A2**[961], SLC1A2/3, SLC25A11, Slc25a12, Slc25a22, **SLC25A3**[665], **SLC25A4**[665], **SNCA**[589], **SRCIN1**[952], STOML2, **STXBP1**[952], **SUMO1**[959], SYN1, SYN2, TCF7L2, **TCP1**[665], UBC, **UBE2I**[959], UQCRC2, VDAC2, VDAC3, **YY1**[740]

99	SLC39A11	C10orf35, CD274, CD33, **EGFR**[962], **ELAVL1**[248], **KLF3**[37], **PON2**[16], PVRIG, SCN2B, SLAMF1, SPN, TMEFF1, TMEM51, WBP1
100	SMN1	A1BG, **A2M**[308], ACTB, ACTL6B, ADAMTS10, **AFG3L2**[43], **AGAP1**[308], ALDH3A2, APLP1, ARFGAP1, **ATP5A1**[43], ATP5I, **ATP6V1A**[308], BAG2, **BAG6**[308], **BCL2**[963], **BLOC1S6**[201], BMI1, BYSL, C19orf60, C19orf70, CAMSAP3, CARHSP1, CCDC90B, CDC5L, **CDKN2A**[43], **CENPB**[308], **CHTOP**[160], CLNS1A, CLPTM1L, **COIL**[964], COL4A2, COL4A5, COP I, **COPA**[965], **COPS6**[308], COX11, COX4I1, CPNE6, CREB1, **CRIP2**[308], CSAD, **CUL3**[161], DDAH2, **DDX17**[588], DDX20, **DDX5**[588], **DERL2**[43], DHCR7, **DHX9**[966], **DICER1**[967], **DMPK**[308], DOCK1, **DOCK7**[308], DUS2, **DYNC1I1**[308], **EEF1A1**[308], **EGFL7**[43], EIF3G, ELOVL1, ERH, **ESR2**[658], **ESYT2**[43], EWSR1, **EXT2**[308], EZH2, FAM20C, FAM9B, **FBL**[588], FGB, **FGF2**[968], FLAD1, **FUBP1**[588], GAPDH, **GAR1**[969], **GDF15**[43], **GDF9**[308], GEMIN2, **GEMIN4**[588], GEMIN5, GEMIN6, GEMIN7, GEMIN8, GGCX, **GIGYF2**[970], **GLUD1**[43], GSK3B, **HADHB**[308], HDAC1, HDAC11, **HDAC2**[971], **HIST1H4A**[588], **HIST2H2AC**[588], **HIST3H3**[972], Histone h3, Histone h4, HMGXB3, HNRNPA1, HNRNPA2B1, **HNRNPD**[973], **HNRNPH1**[588], **HNRNPH3**[588], HNRNPLL, **HNRNPM**[973], **HNRNPR**[588], **HNRNPUL1**[201], HSPB1, **IGHM**[308], ILF3, **IMMT**[308], INPP5K, INTS3, **IQCB1**[529], **IQGAP3**[43], JADE1, KDM1A, **KHDRBS1**[974], **KIF5A**[308], **KLHL5**[308], **KLHL8**[43], **KMT2B**[308], **KPNB1**[975], **KRT8**[43], LENG8, **LGALS1**[976], **LRIF1**[308], LSM10, LSM11, LSM2, LSM4, **LSM6**[977], LSM7, **MAP3K5**[978], MAST2, **MDC1**[170], MED31, **MIB1**[979], MKI67, **MPP1**[308], **MRPL37**[308], MSH2, **MYBBP1A**[588], **MYC**[980], NCBP2-AS2, NGFR, NKIRAS2, NMT2, NONO, **NOP56**[588], **NOP58**[588], NOP9, NOS2, NPM1, OSTF1, **P4HA1**[588], **PAN2**[551], **PDE4DIP**[308], **PFN1**[845], **PFN2**[845], PHAX, PHLDB2, PINX1, **PKM**[308], PLXNA3, **PNKD**[43], **POLR1C**[201], **POLR2A**[948], POP7, PPIG, **PPP2R3C**[43], **PPP4C**[981], **PRKACB**[43], **PRMT5**[948], Profilin, PRSS56, PSME1, **PTDSS1**[43], PTPN23, QARS, **RAF1**[43], **RBBP4**[308], **RBBP6**[982], **RBFOX2**[983], RBM48, REEP5, RNA polymerase II, RNF2, RNF5, RPL10, **RPL13**[308], **RPL6**[588], **RPL7**[588], **RPS2**[174], **RXRA**[308], **SDF4**[308], **SEC22B**[43], SEMA5B, SETDB1, **SETX**[948], SF3B1, **SFPQ**[588], **SIN3A**[971], **SIN3B**[971], **SLC1A5**[43], SLC35E1, **SLC38A5**[43], SLC7A5, SLX1A/SLX1B, SMC5, **SMN1**[5]/SMN2, **SNRNP70**[588], **SNRPA1**[588], **SNRPB**[588], **SNRPD1**[588], **SNRPD2**[588], **SNRPD3**[977], SNRPE, **SNRPF**[588], SNRPG, SNUPN, **SNW1**[364], SP110, SRP19, SRP54, SRP68, **SRP9**[984], **SRSF1**[973], SRSF2, **SRSF3**[588], **STRAP**[43], SULT1A3/SULT1A4, **SUMO3**[308], **SYNCRIP**[973], TAF1C, TCERG1, **TERT**[985], **THRAP3**[588], **TIA1**[43], **TIAL1**[973], TIMM23, **TIMM50**[43], **TLE1**[308], TMEM161A, TMEM209,**TMPO**[588], **TMSB10**[240]/TMSB4X, **TOE1**[986], **TP53**[308], TRAM1, TRMT2A, TUBA1A, **TUBB3**[308], **TXLNG**[43], **TXN**[43], **U2AF2**[973], **UBL4A**[492], **UCHL1**[987], **UNC119**[308], USP4, **USP9X**[693], VAV2, **VKORC1**[43], **VPS28**[988], WDR18, WDR73, **WDYHV1**[5], WIZ, **WWOX**[148], XPO1, **XRN2**[948], **YBX1**[973], YBX2, **ZBTB16**[308], ZNF431, ZPR1, ZXDC
101	SMN2	A1BG, ACTB, ACTL6B, ADAMTS10, ALDH3A2, APLP1, ARFGAP1, ATP5I, BAG2, BMI1, BYSL, C19orf60, C19orf70, CAMSAP3, CARHSP1, CCDC90B, CDC5L, **CHTOP**[201], CLNS1A, CLPTM1L, COL4A2, COL4A5, COP I, COX11, COX4I1, CPNE6, CREB1, CSAD, DDAH2, DDX20, DHCR7, DOCK1, DUS2, EIF3G, ELOVL1, ERH, EWSR1, EZH2, FAM20C, FAM9B, FGB, FLAD1, GAPDH, GEMIN2, GEMIN5, GEMIN6, GEMIN7, GEMIN8, GGCX, GSK3B, HDAC1, HDAC11, Histone h3, Histone h4, HMGXB3, HNRNPA1, HNRNPA2B1, HNRNPLL, HSPB1, ILF3, INPP5K, INTS3, JADE1, KDM1A, LENG8, LSM10, LSM11, LSM2, LSM4, LSM7, MAST2, MED31, MKI67, MSH2, NCBP2-AS2, NGFR, NKIRAS2, NMT2, NONO, NOP9, NOS2, NPM1, OSTF1, PHAX, PHLDB2, PINX1, PLXNA3, POP7, PPIG, Profilin, PRSS56, PSME1,PTPN23, QARS, RBM48, REEP5, RNA polymerase II, RNF2, RNF5, RPL10, SEMA5B, SETDB1, SF3B1, SLC35E1, SLC7A5, SLX1A/SLX1B, SMC5, **SMN1**[16]/SMN2, **SNRNP70**[16], **SNRPB**[989], **SNRPD1**[989], **SNRPD2**[989], **SNRPD3**[989], SNRPE, **SNRPF**[989], SNRPG, SNUPN, **SNW1**[364], SP110, SRP19, SRP54, SRP68, SRSF2, **STRAP**[16], SULT1A3/SULT1A4, TAF1C, TCERG1, TIMM23, TMEM161A, TMEM209, TRAM1, TRMT2A, TUBA1A, USP4, **USP9X**[990], VAV2, **VPS28**[201], WDR18, WDR73, WIZ, **WWOX**[178], XPO1, YBX2, ZNF431, ZPR1, ZXDC
102	SNCG	**ABHD5**[991], **ADRB2**[992], **BMPR1A**[43], BUB1B, **CENPE**[43], **DDX5**[177], **DYNLL1**[177], **EXT2**[177], FABP4, **FLOT2**[993], **FUBP1**[177], GRK1, GRK5, GRK6, **HNF4A**[177], IRS2, JUN, **MAPK1**[994], MAPK3, **MAPK8**[994], MCM3, **MSN**[177], **POT1**[282], **POU4F1**[995], **PPARG**[996], SLC6A4, SNCG, **TERF1**[282], **TERF2IP**[282], TIMP2

103	SOD1	1-palmitoyl-2-oleoylglycero-3-phosphoglycerol, 2-methoxyestradiol, 3-nitro-L-tyrosine, **ABHD5**[991], ACTB, **ADRB2**[314], **ALB**[43], ALS2, **AMFR**[997], **APOA1**[314], ASCC1, **ATP5A1**[705], **BAG3**[784], **BCL2**[998], **BTG2**[999], C4BPA, carazolol, CCS, CCS-Cu2+, **CEBPA**[1000], **CEP55**[43], **CHGB**[1001], cholic acid, Co2+, **COMMD1**[1002], copper, **CRYAB**[1003], Cu2+, CXCR4, **DCTN1**[784], **DERL1**[1004], DNAJ, DNAJA1, DNAJB2, DRAP1, DVL1, **DYNC1I1**[1005], DYNC2LI1, Dynein, **DYNLT1**[1006], **EEF1D**[809], **EGR1**[1007], EIF5A, **EPAS1**[1008], EWSR1, FAAP20, **FBXO32**[578], **FMR1**[1009], **FOXO3**[1010], **FYN**[43], GAPDH, **GAR1**[43], **GJA1**[670], GLUL, HDAC6, HECW1, HEXB, HIVEP1, Hsp70, HSPA1A/HSPA1B, **Hspa1b**[1011], **HSPA2**[1012], **HSPA4**[1013], HSPA4L, HSPA5, HSPA8, **HSPA9**[1014], HSPB1, **HSPH1**[1012], **HTT**[1015], hydrogen peroxide, hydroxyl radical, **IFI16**[832], **INVS**[529], **IQGAP1**[809], **JRK**[36], **KLC2**[43], L-alpha-palmitoyloleoyl-phosphatidylcholine, LGMN, MAPK3, MARCH5, METTL14, **NCBP1**[43], NCBP2, **NEFL**[1014], **NFE2L2**[1016], NFKBIA, **NFS1**[43], NR4A2, **NUDT21**[43], oleic acid, **OPTN**[802], oxygen radical, P3H1, **P4HB**[809], **PARK7**[809], **PDCD6**[43], PDIA2, peroxynitrite, PHF21A, POU2F1, **PPARG**[1017], **PPARGC1A**[1018], **PPP1R12A**[641], PRCC, PRR12, PRSS1, **PSMD4**[1013], **PTBP1**[641], **RAC1**[1019], **RACK1**[641], **RBBP6**[43], RCAN1, **RNF19A**[918], **RNH1**[705], RRAS2, SKIV2L2, SLC27A4, **SMAD2**[315], Sod, **SOD1**[1003], SQSTM1, **SRRT**[43], SSR4, steroid hormone, **STUB1**[1020], STXBP5L, SUMO, SYNE2, **TP53**[1021], TUBA1A, **TUBB2B**[705], UBC, **UBE3A**[1022], **USP9X**[43], WTAP, WWP2, XBP1, **YES1**[38], ZC3H18, zinc, ZNF346
104	SOD2	1-palmitoyl-2-oleoylglycero-3-phosphoglycerol, **ADRB2**[394], **AHR**[1023], **APOA1**[394], APP, **ARF6**[177], ATF1, **ATF2**[1024], **ATXN3**[1025], **AURKA**[177], **BTG2**[999], carazolol, CDK1, cholic acid, CST1, CST4, CYP4F12, DDB2, **DYNC1LI1**[315], **E2F1**[1026], **EGR1**[1027], **EIF1B**[177], EP300, **EPAS1**[1028], FAM192A, **FOXO1**[1029], **FOXO3**[1030], **FOXO4**[1025], **GET4**[29], GOLGB1, GST, **H2AFX**[528], **HIF1A**[1031], **HSP90B1**[315], **HTT**[686], **IKBKE**[177], IRF6, JUN, **KAT5**[1032], **KCNMA1**[14], **KDM5A**[812], KIAA1549, **KLF11**[1033], **KLF4**[1034], L-alpha-palmitoyloleoyl-phosphatidylcholine, **MCC**[177], MED30, mir-146, MPG, MYH8, **NFE2L2**[999], NFkB (complex), **NFKB1**[1035], NFKBIA, NOL12, NPM1, **P4HB**[315], PAK2, **PARK7**[1036], peroxynitrite, POLG, **PPARGC1A**[1037], PRDM2, PRKCB, RAB4A, RB1, **REL**[1038], RELA, RIPK2, **RPL21**[315], **RPS3A**[29], SCRN1, SIRT1, SIRT3, SIRT6, **SMARCA2**[315], SMARCA5, Sod, **SOD2**[422], **SP1**[1034], **STAT3**[1039], **STK4**[1040], TCEAL9, TFAP2A, TFAP2B, **TFAP2C**[1041], TMEM131, TNIK, **TP53**[1042], **UBR2**[16], USP36, **VHL**[177]
105	SOX5	**Acan**[1043], **AES**[201], **APP**[247], ARID5A, **BCL6**[196], BMP6, **BMPR1A**[1044], CBX8, CDC23, CDC25A, **CDK6**[1045], CEP85, COL2A1, CRX, **CTNNB1**[1046], FAM46B, FGFR3, FTH1, **GLI1**[1047], **HSPG2**[1043], **ID2**[222], **ID3**[222], **IL10**[1048], **KAT5**[201], **KIFC3**[201], LIPE, LMO1, LMO2, MAF, MED27, MITF, MORN3, **MYC**, POU5F1, PRR20A (includes others), RORC, **RPS2**[422], **RUNX1**[1049], **RUNX2**[1043], SHOX, **SMAD1**[370], SMAD5, **SMAD7**[370], SMARCD3, Sox, **SOX2**[1050], **SOX5**[201], SOX6, SOX9, SPAG6, T, TAF6, TTC1, UQCRFS1, XIST, ZNF581
106	SPAST	**ALB**[31], ATL1, ATP6AP1, ATP6V0A1, **ATP6V0D1**[43], **ATP6V1A**[43], ATP6V1B2, ATP6V1G1, CAPN7, **CAV1**[43], CCDC115, CD2AP, CENPF, CHMP1A, CHMP1B, CHMP2A, **CHMP2B**[43], CHMP3, **CHMP4B**[43], CHMP5, **CLTA**[43], **CLTC**[43], CPM, **ELAVL1**[248], EMX2, ERLIN1, **FLOT1**[43], **FLOT2**[43], FOSL2, **HECW2**[517], **HNF4A**[18], **HOXA10**[1051], IST1, KIDINS220, **LCK**[43], LYPLAL1, MBOAT7, microtubule-severing ATPase, MISP, MITD1, MVB12A, **NECTIN3**[43], NLRP3, ONECUT1, **PDCD6**[43], **PDCD6IP**[43], PTPN23, RAD9A, RTN2, SARAF, SNTB2, SOAT1, SPAST, STOM, TGS1, TM9SF1, TMED9, **TSG101**[43], TUBA3E, VCP, VPS37C, VPS4B, VPS9D1
107	SPG11	AP5M1, **ELAVL1**[43], SERTAD3, SPG11
108	SPG7	ART3, CCNDBP1, CERK, **ELF3**[177], **EMC2**[43], **FAF2**[174], **FLOT1**[43], GPR55, **HNRNPK**[201], HTR3C, **ISLR**[16], **JUNB**[43], **KHDRBS2**[201], **KIF1BP**[43], KRT40, **KRTAP10-3**[201], **KRTAP10-7**[201], **KRTAP10-9**[201], KRTAP4-12, KRTAP5-9, , **LRIF1**[29], LZTS2, MAPK6, MDFI, **MTUS2**[201], NDC80, NDUFB9, PDK1, **PLSCR1**[5], PNMA1, PSME3, PSTPIP1, RALY, **RBPMS**[201], **RIF1**[29], RING1, **SDF4**[174], SPATS1, SPG7, STAMBP, TPX2, **TRIM28**[29]
109	SQSTM1	26s Proteasome, ABHD10, ACVR1, **AGAP1**[1052], AIM2, AJUBA, AKT1S1, **ANK2**[260], **AR**[1053], **ARHGEF28**[158], ARID5A, ASPH, **ATG16L1**[1054], ATG3, ATG4B, **ATG5**[1055], ATG7, **ATM**[1056], **ATXN3**[1057],

		atypical protein kinase C, BACH1, **BAG3**[1058], BCL10, **BCL2**[1059], BECN1, BID, BLOC1S5, BMPR1B, **BNIP1**[1060], BPTF, C10orf2, **C1QBP**[395], CALCOCO2, **CALM1**[750] (includes others), **CALR**[260], **CAMK2A**[1061], CASP10, **CASP8**[1062], **CASP9**[1062], **CAV1**[1063], Ccl7, **CCNB1**[1064], **CCND1**[397], **CD44**[43], CD48, CD70, **CDC37**[953], CDK1, CEP135, CEP78, CFLAR, **CFTR**[1065], CHAF1A, CHDH, **CHMP2B**[1066], CHRNA7, CITED2, CNTRL, CREB1, CRHBP, **CSNK2A1**[925], CSNK2A2, **CTNND1**[334], CUL1, **CUL2**[1066], **CUL3**[1067], CUL7, Cxcl11, CXCL2, **CYLD**[1068], CYP2A6 (includes others), **DAZAP2**[308], DCAF1, **DCP2**[16], DIP2B, **DLG4**[1069], DNAI1, DNAI2, DNAJB2, DVL2, Dynein, **DYNLL1**[395], EDEM1, EDN1, **EEF1D**[422], EGLN3, EMILIN3, ENC1, ENPP1, **EPAS1**[1070], EPDR1, EPM2A, **FADD**[1071], **FAS**[1072], FAU, FGFR1OP, FGL2, FHOD3, **FKBP4**[1061], FLCN, FNIP1, **FUS**[1066], **GABARAP**[334], **GABARAPL1**[334], **GABARAPL2**[334], GABRR1, GABRR2, GABRR3, GAS6, GBAS, **GEMIN4**[971], GFM2, **GJA1**[1073], GLG1, GMPPA, GPC4, **GRB10**[396], **GRB14**[396], **GRIA1**[1074], **GRIA2**[1075], **GRIA3**[1075], GRIN2A, GSTM5, GTF3C3, HACE1, **HADHA**[334], **HADHB**[334], **HBEGF**[1056], HDAC6, **HIF1A**[1076], HNRNPA2B1, HSP90AA1, HSPA1A/HSPA1B, **HSPA4**[1061], HSPA5, HSPB1, **HTT**[1077], **IFI16**[503], IFNB1, **IKBKG**[1078], IL15, IL15RA, **INA**[334], **INSR**[1079], IPO5, IRAK, **IRAK1**[1080], IRF1, **IRF8**[1068], IRS1, **ISG15**[1081], KCNA5, KCNAB1, KCNAB2, KEAP1, KIAA0753, **KIF5B**[43], **KLF2**[1082], **KLF3**[1082], KLHL3, KRT18, **KRT8**[1083], KRT82, KRT85, **LCK**[1084], **LDHA**[1079], **LHX2**[1056], LIMD1, LLGL1, LLGL2, LTA, MALT1, **MAP1A**[1085], **MAP1B**[1085], MAP1LC3, **MAP1LC3A**[260], **MAP1LC3B**[308], **MAP1LC3C**[334], MAP2K5, MAP3K2, **MAP3K3**[1086], **MAPK1**[1064], **MAPK13**[1064], MAPK14, MAPK3, **MAPT**[1087], **MBP**[1061], **MED4**[1088], MEIS2, MGP, MLH1, **MLST8**[1089], MOV10, MRPL38, **MTOR**[1089], **MYC**[410], **MYD88**[1090], MYO10, **NAP1L4**[304], NBR1, **NCOR1**[1061], NDUFA5, NDUFS2, NDUFS3, **NEDD4**[260], **NEFM**[334], NEK9, **NFE2L2**[1091], NFKBIA, NGFR, NIPSNAP1, NLRC4, NLRP3, NOD2, NPM1, NQO1, **NR1H4**[1092], **NR2F2**[1093], NSUN2, NSUN4, **NTRK1**[1094], NTRK2, NTRK3, NXF1, **OPTN**[1095], ORMDL1, OSBPL8, OTUD7B, P38 MAPK, **PAN2**[551], PARD6A, PARD6B, PARD6G, PARK2, PARP10, PAWR, PCK1, PDE4A, PELI3, PIK3CA, **PIK3R1**[1052], **PIK3R2**[1052], Pkc(s), PLAT, PLK2, PLN, **PML**[43], POC1A, POC1B, **PPHLN1**[308], **PRKAA1**[260], **PRKCD**[1064], PRKCI, **PRKCZ**[1080], **PSMC2**[1096], **PSMD4**[1096], PYCARD, **RAD23A**[5], **RAD23B**[1097], **RAD54L2**[1098], **RAPGEF2**[1099], **RARA**[1100], RBM45, RCN2, **RELN**[1061], RGCC, RILPL1, RIPK1, RPL37, RPS6KB1, RPTOR, RRAGB, RRAGC, RSAD2, SASS6, SCCPDH, SESN1, SESN2, SH3GLB1, SH3GLB2, **SH3KBP1**[1101], SHANK1, **SKI**[415], SLC40A1, **SMAD2**[435], SMAD3, **SMAD4**[435], **SMURF1**[435], SMURF2, **SOD1**[1102], SPDEF, **Spg20**[1103], SPRED2, SQSTM1, **SRRM2**[555], **STAT5A**[1104], **STAT6**[190], **STXBP1**[1061], SYNPO2, **TARDBP**[1066], TBK1, TCF20, **TGM2**[1105], TKT, **TMEM17**[342], TNFRSF10A, **TNFRSF10B**[1071], TNFSF10, TNK2, **TOE1**[29], TOLLIP, **TP53**[1105], TP53INP1, **TRAF1**[1106], TRAF4, TRAF6, **TRIB3**[1107], TRIM13, TRIM21, Trim30a/Trim30d, TRIM5, TRIM50, TRIM55, TRIM63, TRMT61B, **TSC22D1**[1056], TTN, TUBA1A, **TWIST1**[1108], **UBA5**[260], UBC, Ubiquitin, **UBR5**[319], **UBXN1**[1109], **ULK1**[1110], ULK2, **USP12**[1056], USP7, WDFY3, WDR62, **WT1**[1111], XPO1, YWHAZ, ZNF274, ZNF768
110	SS18L1	AATF, ANKRD22, ATF3, BMI1, BRD1, CLPB, CPSF7, **CREBBP**[1112], CSTF2, CTAGE5, **CUL3**[161], DGCR6/LOC102724770, DPF2, DPF3, ELF5, EP300, FAM168A, GATAD1, **HDAC2**[1113], **HDAC4**[1113], LGALS3, MAPK1IP1L, MED30, MPND, **NR1H3**[1113], PCGF6, QKI, **RBM14**[751], RFX6, RLIM, **SF3A1**[800], SF3B4, **SMAD1**[1113], SMAD3, **SMARCA2**[1114], **SMARCA4**[1115], **SMARCC1**[1116], SMARCC2, SMARCD1, **SMARCE1**[16], **SNRPB**[201], SNRPC, SS18L1, **STAT3**[1113], TAF9B, **TCF7**[1113], **TFG**[197], TIMM8A, USP30, WHSC1L1
111	SUSD1	ATF7IP, **EGFR**[577], FBXO6, miR-636 (miRNAs w/seed GUGCUUG), **PTPRK**[16], RNASE13, SUSD1
112	SYNE	APC, BMI1, CAPN1, CCSER2, CCT2, **CCT3**[483], **CCT4**[483], **CCT5**[483], **CCT6A**[483], CCT7, **CCT8**[483], **CDC42**[290], CDC5L, **CEP63**[271], **DISC1**[271], DIXDC1, **DTNBP1**[271], **EMD**[1117], EZH2, **GAN**[436], **IFI16**[408], IFT57, **KIAA0368**[279], **LMNA**[1117], MCM2, MUSK, MYOM1, **NDE1**[319], **NDEL1**[271], **NTRK1**[142], **NUP107**[1118], NUP35, OBSL1, **PAFAH1B1**[319], PPME1, RNF2, **RPA2**[319], SH3BP5, SUN1, SUN2, **SUZ12**[198], SYNE1, SYNE3, **TCP1**[483], **TERF2**[1119], TNIK, TP53RK, TRIM29, TUBA1A, **TUBA1C**[483], **VCL**[399]
113	SYT9	ARF1, Creb, **CSNK1A1**[1120], DNAJC5, **POU4F1**[995], SNAP25, **STX1A**[1121], **SYNCRIP**[1122], SYT9, TRPV1, **TUBB**[1123], **WNK1**[1124]

114	TAF15	APOBEC3B, BLMH, BMI1, BTBD10, **CACNA1A**[330], CAND1, COPS5, **COPS6**[161], COPS7B, CUL1, **CUL2**[161], **CUL3**[161], **CUL4A**[161], **CUL4B**[161], CUL5, CUL7, **DCUN1D1**[161], **DDX3X**[43], **DGCR8**[43], **DHX9**[43], **EED**[350], EWSR1, EZH2, FAM208A, FAM208B, **FBXO32**[578], **FUS**[751], GOLGA1, **HIST1H3A**[1125], **HIST2H2AC**[1125], HIST2H4A, **HIST3H3**[540], HNRNPA1, HNRNPA2B1, **HNRNPA3**[43], **HNRNPC**[43], **HNRNPDL**[43], **HNRNPH3**[43], **HNRNPK**[43], **HNRNPR**[43], **HNRNPUL1**[43], Holo RNA polymerase II, ILF3, KAT2B, **KHDRBS1**[43], **MAPK13**[480], **MATR3**[43], MCM2, MED12, MED26, **NCOA5**[43], **NEDD8**[161], NF2, OBSL1, PHLPP1, PKN3, PLEC, **POLR2A**[1126], POLR2C, POLR2E, POLR2G, **PPHLN1**[43], **PPP1CA**[43], **PPP1CB**[43], **PPP1CC**[43], PRKAR1A, **PRMT1**[16], PRMT8, RAPH1, **RBM12B**[43], **RBM14**[43], **RBM3**[43], **RBM6**[43], **RBMX**[43], RBMXL1, RNF2, **RPA1**[43], **RPA2**[279], **RPA3**[279], **RPS6KB2**[486], **SAFB**[43], **SAFB2**[43], SF1, **SFN**[1127], **SIRT7**[414], SLC9A1, SLTM, **SNRNP70**[43], SNRPC, SNX29, **SRSF1**[43], **STAT4**[1128], **SUZ12**[350], Taf, TAF10, TAF11, TAF12, TAF13, **TAF15**[399], TAF2, TAF4B, TAF5, TAF6, **TAF7**[1129], TAF7L, TAF9, TBP, **TCF3**[826], **TNK1**[43], **TRIM27**[43], VAMP4, **VCL**[493], YLPM1, **ZNF326**[43], ZNF638
115	TARDBP	ACRV1, **ADAR**[590], **ADRB2**[564], **AGO2**[590], AHNAK, **APP**[188], **ARF6**[151], **ARHGEF28**[158], ASPH, **ATP5A1**[590], **ATXN2L**[590], BAG2, BCL2L11, **BCLAF1**[590], BEX2, BMI1, **BRCA1**[434], **C1QBP**[590], CAND1, CAPRIN1, CASP3, **CCAR2**[434], **Ccl2**[1130], CD36, CDC5L, CDC7, CDK4, **CDK6**[1131], CELF1, **CEP290**[342], **CFTR**[1132], CPSF1, CTAGE5, **CUL2**[1133], **CUL3**[161], CUL7, **DAP3**[590], DCD, **DDX17**[590], DDX21, **DDX3X**[590], **DDX5**[590], DDX50, **DDX6**[590], **DGCR8**[1134], DHX30, DHX36, DHX57, **DHX9**[590], **DICER1**[1134], DIMT1, DNAJB6, **DROSHA**[1134], DSC1, **DSP**[590], **EED**[256], **EEF1G**[590], **EEF2**[590], **EIF2S3**[590], EIF3A, EIF3B, EIF3C, **EIF3H**[590], EIF3I, EIF4G1, EIF4G3, **ELAVL1**[590], ELAVL2, EXOSC10, **FAM120A**[590], **FAM98A**[590], FBXO6, FIS1, FN1, **FUBP3**[590], **FUS**[490], **G3BP1**[590], **G3BP2**[590], **GIGYF2**[590], GNL3, GPR137B, GRSF1, GTF2E1, **GTF2E2**[1113], H1FX, HDAC6, HELZ, HIST1H1D, **HNRNPA0**[590], HNRNPA1, HNRNPA2B1, **HNRNPA3**[590], HNRNPAB, **HNRNPC**[590], **HNRNPDL**[590], **HNRNPF**[43], **HNRNPH1**[590], **HNRNPH2**[43], **HNRNPM**[590], **HNRNPR**[590], **HNRNPU**[590], **HNRNPUL1**[590], **HNRNPUL2**[590], Hsp70, HSP90AA1, **HSPA4**[590], HSPA5, HSPA8, **HSPH1**[590], **HTT**[1135], **IFNG**[1130], **IGF2BP2**[590], IL1B, **IL6**[1130], **ILF2**[590], ILF3, IP6K3, **IRAK1**[796], **IRAK2**[796], ITGA4, **JUP**[590], **KHDRBS2**[1136], KHSRP, **KPNA2**[590], LARP1, LARP4, LCN2, LDB1, LOC102724159/PWP2, LRRC15, LSM12, **LSM6**[874], LUC7L2, MAP1LC3, MAP4, **MATR3**[590], MCM2, **MED16**[1113], **MED6**[1137], MFN1, mir-132, mir-143, mir-558, mir-574, MOV10, MRPL12, MRPL22, MRPL44, **MRPL48**[590], MRPS22, MRPS27, MRPS34, MSI1, MSI2, MTDH, **MYBBP1A**[590], **MYH9**[590], **NACA**[590], NACC1, **NCBP1**[590], **NCL**[590], **NEFL**[1138], **NKRF**[590], NOP14, NOP16, NOP2, **NOP56**[590], **NOP58**[590], NPM1, **NSFL1C**[29], **NTRK1**[172], NUFIP2, NXF1, OTUB1, **PABPC1**[590], PABPC4, PABPN1, PARK2, PATL1, PAXIP1, **PCBP1**[590], **PCBP2**[590], **PHGDH**[590], **PKP1**[590], **PLCB1**[276], POLRMT, **PRPF19**[590], PRPF3, **PRRC2A**[590], PRRC2C, **PSMA3**[500], **PTBP1**[590], **PURA**[590], PURB, PYM1, QPCTL, **RACGAP1**[43], **RACK1**[590], RALY, **RBBP5**[43], RBM39, RBM45, **RBMX**[590], RFC4, RNF10, RNF2, **RPA1**[279], **RPA2**[279], **RPA3**[279], RPL10, RPL10A, **RPL11**[590], RPL12, **RPL13**[590], RPL13A, RPL14, RPL15, RPL17, RPL18A, RPL19, **RPL21**[590], **RPL22**[590], RPL23, **RPL23A**[590], **RPL26**[590], RPL28, RPL3, RPL31, RPL32, RPL35, RPL4, RPL5, **RPL6**[590], **RPL7**[590], RPL7A, RPL8, **RPL9**[590], RPLP0, RPS11, **RPS14**[590], RPS16, RPS17, RPS18, **RPS2**[590], RPS20, RPS23, RPS24, **RPS25**[590], **RPS3**[590], **RPS3A**[590], **RPS4X**[590], **RPS6**[590], RPS7, **RPS9**[590], RPSA, RRBP1, **RUVBL2**[590], SART1, SDAD1, **SERBP1**[590], SETDB1, **SFN**[590], SKIV2L2, SLC25A23, **SLC25A5**[590], SND1, SNRNP200, **SNRPA1**[590], SNU13, **SNW1**[364], SQSTM1, SRBD1, **SRPK1**[590], **SRRT**[590], SRSF6, SRSF7, SSB, **STAU1**[590], **SYNCRIP**[590], **TARDBP**[590], TBC1D1, **TNF**[1130], TNFRSF14, **TOB1**[486], **TOP1**[590], TPI1, **TRIM28**[590], TRMT1L, TSR1, **TUBA1B**[590], **TUBB**[590], U2SURP, UBC, UBE2E1, UBE2E2, UBE2E3, Ubiquitin, **UBQLN1**[1139], **UBQLN2**[1140], **UPF1**[590], **USP10**[590], **USP8**[874], **VCAM1**[418], VCP, **VHL**[1133], **WWOX**[178], XPO5, **XRCC5**[590], XRCC6, XRN1, **XRN2**[590], **YBX3**[590], YTHDC2, **YTHDF2**[590], ZC3H11A, ZC3HAV1, ZFAND2B, **ZFR**[590], ZHX1, ZNF346, ZNF750
116	TBK1	ACAD11, **AGO2**[796], Akt, **AKT1**[1084], ALDH9A1, ANKRD28, APBA3, **APP**[247], **ATG101**[254], **ATG13**[254], **ATG16L1**[254], ATG9A, **ATP5A1**[408], AZI2, BBOX1, BIRC2, BIRC3, BTRC, c-Src, CALCOCO2, CCDC191, CCDC8, CCT2, **CCT3**[408], **CCT4**[408], **CCT5**[408], **CCT6A**[408], CCT7, **CCT8**[408], CDC37, CDC42BPG, CEP128, CEP135, CEP152, CEP44, **CEP63**[342], CHUK, CNKSR2, CNTROB, **CREBBP**[1141], CUL1, **CYLD**[1142],

		**DDX3X**[1143], DDX41, DDX58, **DIABLO**[254], DOK3, DTX4, DYRK2, **EED**[256], **EGFR**[515], **EIF2AK2**[1144], **EIF5A2**[621], EP300, ESR1, EXOC2, **EXOC4**[1145], FBXO3, **FMR1**[796], FXR2, GAPDH, **GFI1**[1146], GLDC, GOLGA3, GSK3B, **HERC2**[1147], **HMMR**[796], **HNRNPM**[408], **HSBP1**[254], HSP90AA1, **HSP90AB1**[408], HSPA5, HSPA8, **HSPA9**[408], **HTRA2**[260], **IFIT**[1148]**3**, IKBKB, **IKBKE**[796], **IKBKG**[408], IKK (complex), IRF, IRF1, IRF3, IRF5, IRF7, JAK inhibitor I, **KCTD17**[621], KIF11, LATS2, LDHB, LETM1, LGALS3BP, LRRC47, **MAP1LC3B**[797], **MAP3K14**[1149], MAVS, **MBP**[1150], MCM2, MCM6, **MED4**[1088], **MIB1**[796], MIB2, **MPP1**[621], MPP6, MTPAP, **MYD88**[1151], **NCK1**[1152], **NCOA2**[1153783], NDUFS1, **NEDD4**[1154], **NFKB1**[408], **NFKB2**[408], NFKBIA, **NINL**[342], NLRP4, NONO, **NTRK1**[172], OBSL1, ODF2, **OFD1**[342], **OPTN**[1155], PELI1, PPM1B, **PPP2R1B**[800], PPP6C, PPP6R1, **PPP6R2**[408], PPP6R3, PROS1, **PRRC2A**[621], **PTPN11**[1156], RB1, **RB1CC1**[334], **RBBP5**[796], RBL1, RBL2, **RBMX**[254], **REL**[408], RELA, Rig1, **RNF11**[1157], **RNF41**[1158], **RNH1**[254], **RPA1**[254], [254], **RPL11**[408], **RPL13**[408], **RPL7**[408], RPS18, **RPS5**[408], **RPS8**[408], **RUVBL1**[408], **RUVBL2**[408], **SAFB**[254], **SAFB2**[254], SASS6, **SCLT1**[342], **SFPQ**[408], SIKE1, **SKP1**[408], SLC27A1, **SMCR8**[254], SOCS3, SQSTM1, **SRC**[1159], SSX2IP, **STAT1**[1160], **STAT6**[1161], STIL, STX11, **STX12**[16], STX19, STX3, **SUMO1**[1162], SYK, TANK, **TAX1BP1**[1163], TBK1, TBKBP1, **TCP1**[408], TICAM1, TICAM2, TLR3, TLR4, TLR9, TMEM173, **TNF**[1164], TNFAIP3, **TNFRSF1A**[895], **TNIP1**[1163], TNIP3, TOMM70, **TRAF1**[16], **TRAF2**[408], TRAF3, TRAF3IP2, TRAF6, TRAIP, TRIM10, TRIM11, TRIM26, **TRIM27**[1165], **TUBB**[408], **TUBB4A**[408], TXLNA, TXLNB, **TXLNG**[796], UBC, UBE2N, USP2, VPS37C, **XIAP**[1166], XPO1, **YWHAB**[800], YWHAE, **YWHAG**[621], **YWHAH**[800], YWHAQ, YWHAZ, ZBP1, ZNF384
117	TRPM7	ACTB, BUB1, **C1QBP**[43], **CCAR2**[1167], CEP152, **CTNNB1**[898], **CUL4B**[1167], **DDB1**[1167], **DDX3X**[1167], **EED**[1167], **EEF1A1**[43], EZH2, **FLOT1**[43], HDAC1, **HIST3H3**[1168], Histone h3, INO80, INO80B, **MBP**[1168], **MYH10**[1169], **MYH14**[1169], MYH2, **MYH9**[1170], **PLCB1**[1171], PLCB2, PLCB3, **PLCG1**[1171], RAB7A, RING1, RNF2, **RUVBL1**[898], **RUVBL2**[1167], RYBP, SIRT1, **SNAP29**[16], **TCTN3**[342], TIMELESS, **TMED2**[43], TRPM6, TRPM7, VAPA, **YY1**[898], ZACN
118	TUBA4A	**ACTN2**[29], Alpha tubulin, ANTXR1, APC, ARAF, **ARRB1**[395], **ARRB2**[395], **ATN1**[1172], B3GNT2, BAG2, BCAR1, **BTK**[1172], C4orf17, cabazitaxel, CAND1, CCNJL, **CD81**[541], CDC20, CDC5L, CDK13, CEP57, CEP76, cevipabulin, **CLIP1**[1173], colchicine, COPS5, **COPS6**[161], **CSNK1A1**[395], CTDSP1, **CTNNB1**[1174], CUL1, **CUL2**[161], **CUL3**[161], **CUL4A**[161], **CUL4B**[161], CUL5, CUL7, davunetide, **DCUN1D1**[161], DDX39A, DNAJB11, DNASE2B, docetaxel, **E2F1**[315], **EGFR**[962], **EMD**[395], ENO2, epothilone B, **ERBB3**[16], eribulin, **FAF2**[1109], **FBXO25**[1175], **FGFR1**[1176], **FMR1**[1009], **FUS**[395], GNAI2, **GOLGA2**[1177], **GRB2**[1178], **GRIN1**[1179], **HAUS1**[549], HAUS2, HERC5, **HSPA2**[395], HSPB1, **HTT**[198], **ICAM1**[16], **IGSF8**[541], IL12RB1, **ILK**[489], INPP5D, INPPL1, **INVS**[529], **IQCB1**[529], **ISLR**[16], JAK3, KLK11, KLK5, larotaxel, LIMK2, **LRRK1**[1178], **LRRK2**[1180], MAGED1, MAGED2, MAPK3, **MAPT**[746], MCM2, MGRN1, **MIB1**[332], milataxel, MOV10, **MYC**[410], **NANOG**[1181], **NEDD8**[161], **NEK4**[153], NPHP4, NR1I3, **NR3C1**[1182], **NR3C2**[1182], NXF1, NXPH3, NYX, OBSL1, paclitaxel, PARK2, **PEX14**[284], plinabulin, podophyllotoxin, **PPP2R2A**[1183], PRG2, PSMD14, PTP4A1, PTP4A3, PTPN12, RBM23, **RPA1**[279], **RPA2**[279], **RPA3**[279], RPL12, **S100A8**[29], **SATB1**[724], SHC1, SHROOM3, **SIK2**[489], SIMC1, SIRT2, **SKI**[415], SLAMF1, SLC25A1, SLC25A13, **SMAD1**[1184], **SMAD2**[315], SNAP25, **SNCA**[589], **SNW1**[364], **SOCS6**[1185], SOCS7, **SOX2**[693], **SRRM2**[555], STARD13, **STAU1**[501], **STXBP1**[1186], STXBP5L, **SUMO3**[193], THBS3, TM4SF1, TMEM25, **TP53**[549], TRAC, **TRIM28**[395], TUBA1A, **TUBA1B**[909], **TUBA1C**[16], **TUBA4A**[1187], **TUBB**[909], **TUBB2A**[909], **TUBB3**[16], **TUBB4A**[909], **TUBB4B**[909], **TUBG1**[549], TUBGCP2, tubulin (complex), tubulin (family), TXNDC15, TYK2, UBC, UCHL5, **VHL**[449], vinblastine, vincristine, vinflunine, vinorelbine, vintafolide, XPO1, YWHAE
119	UBQLN2	26s Proteasome, ADRM1, **ATXN7**[330], BCL2L11, BTRC, C1QA, **CCNB1**[1188], CD47, CD93, EDEM1, **ELAVL1**[248], EMC3, EMC7, EMC8, EPN1, EPN2, EPS15, **FAF2**[1189], **FBXO25**[612], **FBXO32**[578], HERC3, HERC6, **HERPUD1**[1190], **HNF4A**[18], HNRNPA1, **HNRNPA3**[560], **HNRNPU**[560], HSPA13, **HTT**[1191], **HUWE1**[1192], INSIG2, **ITGB3**[1193], MMGT1, **MTNR1B**[305], **Nedd4**[1194], **NFE2L2**[22], **NTRK1**[172], PRPF40A, PSMA2, **PSMA5**[1195], **PSMA6**[1195], PSMC1, **PSMC2**[1196], **RAD23A**[1197], **RAD23B**[1192], **RNF11**[370], RNF181, RNF185, **SHFM1**[1192], **TARDBP**[1140], **TP53**[848], UBC, **UBE3A**[1198], UBE3C, **UBQLN1**[1199], **UBQLN2**[1199], UBQLN4, UBR4

120	UNC13A	BSN, **CACNA1A**[1200], CACNA1B, **Calm1**[1201] (includes others), Calmodulin, DNPEP, DOC2A, **EPB41L3**[939], ERC2, FBXO45, MOV10, phorbol 12,13-dibutyrate, RIMS1, RIMS2, **SHMT2**[374], **STX1B**[1202], UBXN10, UNC13A
121	VAPB	ACBD4, ACBD5, AFTPH, AHCTF1, AKAP11, ARHGEF12, ASNA1, ATL3, BAG2, **BAG6**[1203], **BMPR1A**[43], BRIP1, CCNB2, **CCT3**[831], **CCT4**[831], **CCT5**[831], CEBPZ, CTU2, DDOST, **DICER1**[1204], **EFNB2**[929], EHMT2, **EPHA4**[1205], **ERBB2**[43], ESRRB, FAF1, **FAF2**[1206], FAM118B, FAM170A, FAM83G, **GATA3**[1207], **GET4**[1208], **GOLT1B**[43], GPR25, **HACD3**[1209], HEATR5B, HSPA4L, **HTT**[1210], **INSIG1**[1211], ITPRIP, JMY, **KIF5A**[1049], **Ktn1**[43], LSG1, LTBR, MAP1S, MAPK3, MIGA2, MT-ND5, MTNR1A, NACAD, NBR1, NDUFA7, NDUFB8, NDUFV3, NOL11, **NTRK1**[172], OSBP, OSBP2, OSBPL10, OSBPL11, **OSBPL1A**[16], OSBPL2, OSBPL3, OSBPL6, OSBPL9, PITPNM1, PITPNM2, PKN2, POTEE/POTEF, PPT1, **PRKACA**[523], **PRKACB**[16], PRKAR1B, PRKAR2B, RAB3GAP1, RAB3GAP2, RAB5C, RASSF1, RASSF3, **RB1CC1**[16], RFT1, RHBDD3, RMDN2, **RMDN3**[43], **RNF41**[11], RPN2, SEPT2, SLC25A35, SLC26A2, SLC6A15, SLC7A2, **SNCA**[589], SNX2, SNX25, SNX5, **SNX6**[16], STARD3, STK3, **STK4**[16], **STX1A**[1203], **STX1B**[1203], STX2, **STX4**[1212], STX5, SYNE4, SYNRG, TACC1, TCTN2, TMEM109, **TMEM216**[342], TTC1, TTC39B, TUBA1A, UBC, UBE4A, **UBL4A**[1203], USP20, USP33, VAMP1, **VAMP2**[1213], VAPA, VAPB, VCP, **VHL**[1214], **VKORC1**[1215], VPS13A, WDR44, **YBX1**[1216], YIF1A, YIF1B, ZDBF2, **ZFYVE27**[1217]
122	VCP	**HNRNPH1**[549], **HNRNPH2**[1218], **HNRNPH3**[1218], HOOK1, HS1BP3, **HSBP1**[1218], **HSF1**[1219], Hsp70, Hsp90, HSP90AA1, **HSP90AB1**[1218], HSPA1A/HSPA1B, **HSPA4**[1218], HSPA5, HSPA8, HSPB1, **HSPD1**[549], HSPE1, **HTRA2**[1109], **HTT**[1218], **HUWE1**[1218], **HYOU1**[1220], ICK, IFT74, IFT88, IGHG1, **IGHM**[1221], **IKBKE**[177], IL2RB, IL9R, INF2, **INSIG1**[1222], INSIG2, **IQCB1**[1218], **IQGAP1**[549], IRS4, **ISG15**[168], ITGA4, **ITGB1**[1223], **ITGB4**[43], **ITPR1**[1224], JAK3, JKAMP, KCMF1, KCNJ11, **KCNMA1**[14], KDM3A, KDM3B, KIAA1468, KIAA1524, **KIF1BP**[1218], KIF20A, L3MBTL1, LMAN1, **LMNA**[1218], LMNB2, **LNX1**[201], **LRIG1**[1218], LYAR, **MAP2K1**[1218], **MAP3K3**[408], MAP7D3, **MAPK1**[1218], **MAPK13**[1218], MAPK3, MAPK8IP2, **MAPT**[1225], **MARK2**[1218], **MCC**[177], MCM2, **MDM2**[1218], **MDN1**[1218], MFN2, MRPS18B, MRPS23, MSH4, **MTOR**[1218], NAPA, NBEA, NCAPH, NCDN, **NCOA1**[783], **NCSTN**[1226], **NDRG1**[1227], NEK2, NEPRO, NF1, **NFE2L2**[1228], **NFKB1**[1229], **NFKB2**[1218], NFKBIA, NFKBIB, NFKBIE, NGLY1, NIPSNAP1, NMD3, NME2, NOS2, NPLOC4, NPM1, **NSF**[1230], **NSFL1C**[1231], **NTRK1**[172], NUB1, **NUMA1**[1218], **NUP107**[1218], NUP205, NUP54, NUP58, NUP62, OBSL1, ONECUT1, **OPTN**[213], OS9, OTULIN, PACRG, PARK2, **PARK7**[1218], PDCD10, PDCD4, **PDCD6**[1218], PDK3, PDXDC1, PEX19, **PFN1**[836], PHKG2, PIK3C2B, **PIK3R2**[1218], PIK3R3, PKD2, **PKM**[1218], PKN2, PLAA, PLEC, PLK1, PLPP3, PNO1, POLR2B, POLR3C, PPFIBP1, PPM1B, **PPP1CC**[1232], PPP1R18, **PPP2CA**[1233], PPP2CB, **PPP2R1A**[1233], **PPP2R1B**[953], **PPP2R2A**[953], **PPP3CA**[549], PPP6C, PPT1, **PRKAA1**[1234], PRKAG1, PRKAR2A, **PRKCD**[413], PRKDC, **PRPF19**[549], PRPF31, **PRPF4**[549], PSMA1, PSMA2, **PSMA6**[1235], PSMA7, PSMC1, PSMC4, **PSMC5**[1236], **PSMD4**[1237], PTCRA, **PTGS2**[1238], PTPN22, PTPN23, **PTPN3**[1239], PTPN9, **PTPRO**[1240], RAB10, RAB11B, **RAB14**[1218], RAB3GAP1, RAB3GAP2, RAB7A, RABGAP1, **RAD23A**[1241], **RAD23B**[1097], **RAF1**[1218], **RARA**[1242], **RBFOX2**[1218], RBM23, RFC3, RFC5, RHBDL3, **RIF1**[1218], RNF103, RNF126, **RNF19A**[1243], RNF2, RNF31, RNF7, RNF8, **RPL13**[549], RPL18A, **RPL22**[549], RPL23, RPL24, RPL30, **RPL6**[1218], **RPL9**[201], RPN1, RPS11, RPS13, **RPS25**[549], **RPS3**[549], **RPS3A**[549], **RPS4X**[549], **RPS6**[549], **RPS6KA1**[308], **RPS8**[549], **RPS9**[1218], RRBP1, RRP12, RSU1, **RUVBL2**[549], **RXRB**[315], RYR2, SAP18, SCD, **SCFD1**[1218], SDCCAG3, **SEC16A**[1218], **SEC22B**[1218], SEC61A1, SELK, SENP3, SERPINA1, SESN2, SFTPC, **SGK1**[177], **SH2D2A**[1244], **SHFM1**[1192], **SIK2**[1245], **SIRT7**[414], **SKP1**[1218], SLC3A2, SLIRP, SLX4, **SMAD1**[1246], SMARCA5, **SMARCC1**[1218], **SMURF1**[363], SNX3, SON, SPAST, SPC24, **SPRTN**[1247], SPTAN1, **SRRM2**[555], SRSF11, **SRSF3**[1218], **ST13**[1218], STAG2, **STAT1**[1218], STAT5a/b, STMN1, **STUB1**[1248], STX5, STXBP5L, **SUMO1**[1218], **SUMO2**[1218], SUPT16H, SUPT6H, **SUZ12**[256], SVIP, SYF2, SYMPK, SYVN1, TAF6L, **TARDBP**[590], **TAX1BP1**[1218], TBC1D10B, TBC1D9B, **TCP1**[1218], TELO2, **TERF2**[1218], TFE3, TIMM44, TMED10, TMEM129, TMEM33, TMPRSS13, TNFRSF14, TNKS1BP1, TNPO3, TOM1, TOM1L1, TOMM34, **TOP1**[1218], **TP53**[1218], **TP53BP1**[1218], **TP63**[491], TPD52L2, TRA, TRAF6, TRIM13, TRIM21, TRIM25, TRIP12, **TSG101**[1218], TSN, TTC26, TTC4, TTK, **TUBA1C**[549], **TUBB**[549], **TUBB4B**[549], TUBGCP2, **UBA5**[1218], UBAC2, **UBB**[549], UBC, UBD, UBE2J1,

		UBE2M, UBE2S, UBE4A, UBE4B, Ubiquitin, **UBL4A**[492], UBOX5, **UBQLN1**[1218], UBR1, UBR4, **UBR5**[1218], **UBXN1**[1249], UBXN10, UBXN11, UBXN2A, UBXN2B, UBXN4, UBXN6, UBXN7, UBXN8, UFD1L, ULK3, **USP10**[1218], USP13, VAPA, VAPB, VBP1, **VCAM1**[1236], **VCL**[1218], VCP, VCPIP1, VCPKMT, vesnarinone, **VHL**[1250], VIL1, **VIM**[1218], VIMP, VPS18, VPS50, VPS53, WAC, WAPL, WBSCR22, WDR43, WDR82, **WDYHV1**[201], **WNK1**[1218], WRAP73, WRN, WRNIP1, YOD1, **YWHAB**[1218], YWHAE, **YWHAG**[1218], **YWHAH**[1218], YWHAQ, YWHAZ, ZFAND2B, **ZFR**[1218], **ZNF326**[1218], ZNF778
123	VDR	2-(3-hydroxypropoxy)calcitriol, 25-hydroxyvitamin D, 3-keto lithocholic acid, ABCB11, ABCC3, ACE, ACTN4, ADIPOQ, **AGT**[1251], AGTR1, alfacalcidol, **ATM**[1252], **BAG1**[1253], BGLAP, **BRCA1**[1254], c-Src, CALB1, calcifediol, calcipotriene, calcitriol, CASC4, CASP3, CASP6, **CASP7**[1255], **CAV1**[1256], **CAV3**[545], **Ccl2**[1257], CCND3, CDCA7L, **CDH1**[1258], CDK11B, CDK7, **CDKN1A**[1259], **CEBPA**[1260], CEBPB, CFH, cholecalciferol, CLASRP, COL10A1, **COL1A1**[1261], COPS2, CPT1, CPT2, CREB1, **CREBBP**[1262], **CSF2**[976], CSH1/CSH2, CSHL1, **CSNK2A1**[1263], CTBP1, **CTNNB1**[1264], curcumin, CXXC5, Cyclin D, CYP19A1, CYP24, CYP24A1, CYP27B1, CYP2B6, CYP2C9, CYP3A4, delphinidin, doxercalciferol, **EGR1**[1265], EMX1, EP300, ergocalciferol, **ETS1**[1266], F3, FABP6, FGF23, **FOS**[1267], **FOXO1**[1268], **FOXO3**[1269], **FOXO4**[1269], FSH, **GABARAPL1**[1270], **GABARAPL2**[1270], **GADD45A**[1271], **GFI1**[1272], **GLI1**[1273], **GLI2**[1274], GNPAT, **GRIP1**[1275], Growth hormone, GTF2B, HDAC1, HIRA, HMGN3, **HNF4A**[1276], HR, **HTT**[1277], **ICAM1**[1278], IER3, **IGFBP3**[1279], IGFBP6, IKK (complex), IL12B, IL18, IL1A, IL1RN, IL2, **IL4**[1280], **IL6**[1281], ILX-23-7553, Ins1, JUN, JUN/**JUNB/JUND**[1282], **KDM5A**[1283], KL, LCOR, LEF1, **LEP**[1284], Lh, LIPE, lithocholic acid, **MAPK1**[1276], MAPK3, **MDM2**[1285], MED1, MED12, MED13, MED14, **MED16**[1286], MED21, **MED23**[1286], **MED24**[1286], **MED4**[1286], **MED6**[1286], MED7, MEN1, mir-155, MMRN2, MXD1, **MYC**[1287], MYF5, MYH3, MYH8, MYOG, N-cor, **NCOA1**[1262], **NCOA2**[1288], **NCOA3**[1269], **NCOA4**[1289], **NCOA5**[1290], **NCOA6**[1291], **NCOR1**[1292], **NCOR2**[1289], NGF, **NKD2**[422], NPHS1, NPPA, **NR0B2**[1270], **NR1H2**[1270], **NR1H4**[1293], **NR4A1**[1294], NRIP1, NSD1, paricalcitol, **PDIA3**[1295], PHEX, **PLCG1**[1296], PLD1, PLIN2, PNPLA2, PODXL, POU1F1, POU2F1, PP1-C, **PPARG**[1297], **PPP1CC**[1269], Ppp2c, PRCP, PRKCB, PRKCSH, PRL, Pro-inflammatory Cytokine, **PSMC5**[1270], **PTGS2**[1298], PTH, **PTPN3**[1299], PYY, quercetin, **RAD50**[1252], Raf, **RARA**[1300], RARB, RB1, RCN2, RELA, RELB, **REN**[1301], **RNF14**[1289], **RUNX1**[1292], RUNX1T1, **RUNX2**[1302], RUNX3, Rxr, **RXRA**[980], **RXRB**[1270], RXRG, S100G, SELE, SENP1, SENP2, seocalcitol, SERPINC1, SIRT1, SLC10A2, SLC34A1, SLC34A2, SLC51A, SLC51B, Slco1a1, SLCO1B3, **Smad2**[1303]/3, SMAD3, SNAI1, SNAI2, **SNW1**[1304], SOCS1, SPP1, **SRC**[1288], **SRPK1**[250], STAR, **STAT1**[1305], **STAT4**[1306], SULT2A1, TAF11, **TAF7**[1307], TBP, **TCF3**[1308], **TDG**[145], **TERT**[1309], **TGFB1**[1310], TGFB2, THBD, **THRAP3**[1311], thyroid hormone receptor, **TK1**[1312], TMPRSS2, TNFSF11, TOB2, **TP53**[1310], **TP63**[1313], **TP73**[1314], TPH1, TPH2, **TRIM24**[1315], TRPV5, TRPV6, TSLP, UCP1, UCP2, UCP3, Vdac, **VDR**[1316], Vegf, vitamin D, VitaminD3-VDR, VitaminD3-VDR-RXR, VitaminD3-VDR-SUG1, **WT1**[1317], **ZBTB16**[1292]
124	**VEGFA**	**A2M**[1318], ADAMTS1, aflibercept, **AHR**[1319], Ap1, ARNT, ARNT2, ASXL1, **ATF4**[1320], ATF6, **ATXN1**[1321], BCL6B, bevacizumab, bevacizumab-IRDye 800CW, CBX4, CCND2, **CDK6**[1322], CITED2, conbercept, **CREM**[30], **CRYAB**[1323], CTGF, dalteparin, DLX4, DTX1, **EGR1**[1324], **EGR2**[610], EHF, **EPAS1**[1325], ESRRG, **ETS1**[1326], ETV6, EZH2, **F2**[1327], Fibrin, Fibrinogen, FLI1, FLT1, FLT4, **FOXM1**[1328], **FOXO4**[1329], **FOXP3**[1330], fucoidin, **GLI1**[1331], GMNN, GPC1, GREM1, **HDAC4**[1332], heparan sulfate, heparin, HGS, HHEX, HIF3A, HIPK1, **HIPK2**[1333], **HNRNPD**[1334], **HNRNPL**[579], HOXB7, **HSPA4**[1335], HTATIP2, **HYOU1**[1336], IGFBP7, ILF3, ITGA3, ITGA9, **ITGAV**[304], **ITGB1**[304], **ITGB3**[304], ITGB6, **JUND**[1337], **KDR**[1338], **KLF4**[624], KLF6, LYVE1, **MECP2**[1339], mir-1, MMP1, MMP16, MMP19, MMP3, MMP7, MYOCD, Neuropilin, **NFE2L2**[1340], NFKBIB, NKX2-1, NPM1, NPTX1, **NR1H2**[1341], **NR1H3**[1341], **NR3C2**[1182], **NRP1**[630], NRP2, PDGFB, **PDGFRA**[1342], PDGFRB, pegaptanib, PGF, PLAG1, PLAU, PLAUR, PLG, **POLR2A**[1322], PPARD, **PPARGC1A**[1343], PPARGC1B, PRRG4, PTN, PTTG1, ranibizumab, RARB, **RBPJ**[1344], RORC, **RUNX1**[1345], **RUNX2**[1010], **RXRA**[1341], SALL1, Smad, **SMAD1**[1346], **SMAD4**[1347], SMAD5, **SMAD7**[419], SOX1, SOX3, SOX9, **SP3**[1348], SPARC, **SREBF1**[1349], **STAT4**[1128], STK16, TEAD4, TFAP2A, **TFEB**[1350], THBS1, **TIA1**[1351], **TP63**[1352], **TP73**[1353], **TRIM24**[1354], U2AF1/U2AF1L5, UBIAD1, VAV1, Vegf Receptor, VEGFA, VEGFB, **VHL**[1355], VPS35, VTN, **WT1**[1356], **ZNF202**[55]

125	VPS54	**PPP1CA**[1357], **STX6**[1358], SYN1, TSSC1, **UFL1**[43], VPS50, VPS51, VPS52, VPS53, VPS54
126	ZFP64	AEN, **BARD1**[170], BEGAIN, BYSL, C14orf1, CDK1, FAM124A, **FHL2**[201], GTF2A1, HDAC1, **HECW2**[517], **HNF4A**[18], **LMNA**[637], LMO2, **LNX1**[201], MAFB, **MED16**[1113], MFAP1, **MSN**[43], **MTUS2**[5], PBK, PPARGC1B, RELA, RHOU, RNF2, SETDB1, **TLE1**[29], TRIM41, **UNC119**[29], ZBTB9, ZFP64, ZNF513, ZNF70
127	ZNF512B	ANAPC15, **ANKLE2**[370], ANKRD28, AP1G1, APLP2, ATRX, BRD1, BTBD2, CCDC8, CDC123, CREBZF, CTBP2, **DCTN1**[370], DDX27, **DDX3X**[370], **DROSHA**[370], DVL3, EHMT2, ESR1, FAM129B, **FHL3**[201], **GLRX3**[370], HDAC1, **HEYL**[370], HIST1H2BG, **HIST1H3A**[1088], HTATSF1, IL36RN, **INVS**[370], KDM2A, KDM3B, KIF18B, KLF10, **KMT2B**[370], L1TD1, LAMB2, let-7, LZTS2, MBD1, mir-17, mir-25, **MTA1**[16], **MYH10**[370], OS9, PAPPA2, PBRM1, **PDE4DIP**[370], PEG10, PIAS4, **PLCG2**[370], PPP1R15A, **PTPN3**[370], PTPRF, **RAPGEF2**[43], **RBBP7**[16], RGPD4 (includes others), **RPS27A**[370], RPTOR, **SDF4**[370], **SIAH1**[16], **SMAD1**[370], SMYD2, **SPTBN1**[370], SUPT5H, **TGM2**[370], **TNKS2**[370], TOM1, TOX4, **TRAF1**[16], TRAF4, **UBA5**[43], UBE2A, **VHL**[370], VPS35, VPS41, VWF, **XPC**[370], XRCC6, **YES1**[370], **ZMYM2**[370]
128	ZNF746	**DIABLO**[308], DUSP23, **ELAVL1**[248], EPHA8, FAM118B, FES, G6PC, GSK3B, HMOX2, **ILK**[308], **MAP3K7**[308], PARK2, PCK1, **PPARGC1A**[1359], PPP2R2C, PRKRA, RPS6KA6, ZAK, ZNF746
129	STX12	ABCA1, ACP2, ADPGK, APBA2, **APP**[1360], **ATP4A**[1361], ATP6V1B1, **BLOC1S6**[1362], **BNIP1**[16], BZW2, CCDC138, CLRN1, CLSTN1, DTX2, **EEA1**[1363], **EIF2B1**[16], EIF2B2, EIF2B3, EIF2B4, EIF2B5, **ELAVL1**[248], EWSR1, F3, FAM234A, FAM234B, **FGFR1**[1176], FRMD5, **GOLGA2**[16], GORASP1, GOSR1, GOSR2, HGS, **HTT**[1210], MARCH2, MOSPD2, MOV10, **MYH9**[43], NAPA, NAPB, NAPG, NBAS, NOC4L, NOP14, **NSF**[334], NSG1, OPN4, **OPTN**[16], PRMT9, RAB11A, RABEP1, RABGEF1, RNF13, SCFD2, Secretase gamma, SNAP23, SNAP25, **SNAP29**[201], SNAP47, STAT2, STX10, **STX18**[16], **STX1A**[1364], **STX4**[16], STX5, **STX6**[16], STX7, STX8, STXBP5, Syntaxin, SYP, TAF9, TBC1D15, TBC1D17, TBK1, **TMEM17**[342], **TMEM216**[342], **TNFRSF10B**[174], TXLNA, USE1, Vamp1, **VAMP2**[16], VAMP3, VAMP4, VAMP7, VAMP8, VPS18, VPS45, VTI1A, VTI1B, YKT6, ZKSCAN1, ZNF219
130	CREB3L2	**ATF5**[1365], **CEP19**[422], CREB3, CREB3L2, **DYNLL1**[399], Ecm, **ELAVL1**[248], EVX1, **FBXW7**[856], GAS7, GCFC2, GULP1, HIVEP3, HSPA5, IHH, MCL1, MX1, **PTCH1**[1366], PTP4A1, PXN, RGS16, RND1, Sec23, SEC23A, SEC23B, SEC24C, SOX9, SYVN1, TCF7L2, UBC, ZNF212
131	DCC	ADORA2B, AGAP2, **ALB**[31], ALDOB, ANP32B, **APPL1**[1367], **AR**[77], ASCL1, **BARD1**[170], CASP3, **CASP7**[1368], **CASP9**[1369], CBLN4, CORO2A, CTSV, DAB1, **DCC**[1370], Dcc dimer, Dcc-Unc5, DSCAM, EIF1AX, EIF2A, **EIF2B1**[1371], EIF2B2, EIF2B4, EIF2S1, EIF2S2, **EIF2S3**[1371], **EIF3E**[1371], EIF4E, EIF4E3, **EIF5A2**[1371], **EZR**[1371], FBP1, **FYN**[1372], HEMGN, heparin, **ISL1**[858], ITGB5, **MAG**[1373], MAP2K1/2, **MAPK1**[1371], MAPK3, MAPKAPK3, MAZ, **MBP**[1373], **MSN**[1374], MYO10, NCK, **NCK1**[1292], NEO1, Netrin, NTN1, NTN3, **POU4F1**[858], **PPP1CA**[1375], **PTK2**[1371], **RASA1**[1376], RDX, ROBO1, ROBO3, RPL10A, **RPL13**[1371], RPL23, RPL28, RPL38, RPL5, RPS10, RPS13, RPS23, RPS24, **RPS4X**[1371], **RPS6**[1033], SFRP2, **SIAH1**[1377], SIAH2, **SRC**[1372], TBC1D2, TDRD7, TMOD1, TRIM9, TRMO, TSTD2, **TUBB3**[1378], UNC5, UNC5B, UNC5C, YAP1
132	KIF5A	ACTB, **Alyref**[909], ANKRD27, **APP**[1379], ARC, ATIC, **CAMK2A**[909], CHRNA9, **DCTN1**[357], **DDX1**[909], **DDX3X**[909], **DISC1**[384], **DNM1**[1380], DTNB, **DTNBP1**[16], EXOC1, [909], GABA-A receptor, **GABARAP**[1381], **GABARAPL1**[1381], **GABARAPL2**[1041], **GRB2**[293], **GRIA2**[1382], **GRIP1**[911], **HACD3**[1043], **HAP1**[357], HNRNPU, **INSR**[1383], ITSN1, KCNC1, KCNE3, **KIF5A**[1384], **KIF5B**[1384], KIF5C, **KLC1**[16], **KLC2**[1385], MAP4K4, **MDM2**[315], **MED4**[1088], **NCOA2**[1386], **NDEL1**[295], NONO, PCBD1, **PIN1**[308], PRKAA2, **PURA**[1044], PURB, Rab11, **RAPGEF2**[367], **RTCB**[909], RTN3, **RXRB**[315], SALL4, **SFPQ**[909], **SMN1**[308]/SMN2, SNPH, **STAU1**[1387], SURF4, **SYNCRIP**[909], SYNE4, TFCP2L1, **TK1**[308], TP53BP2, TRAF3IP1, TRAK1, TRAK2, **TRIP6**[16], **TSG101**[1388], VAPA, VAPB, **VPS26B**[43], YAP1, YWHAE, **ZFYVE27**[1389]
133	PEAK1	**AURKA**[16], BCAR1, **CARD8**[16], **CRK**[407], **CRKL**[43], ERK, **GRB2**[16], **MIB1**[332], **MTNR1B**[305], **NCK2**[16], PEAK1, **PPP1CA**[16], **PPP1CC**[16], PPP1R3A, PXN, **SH2D2A**[16], SHC1, **SORT1**[43], **SRC**[1390] (family), TOR1AIP1, **XRCC5**[43]
134	BIRC6	ANAPC11, BANK1, BIRC5, BIRC6, C3orf20, CA11, **CALM1**[396] (includes others), CASP3, CASP6, **CASP7**[1391], **CASP8**[1391], **CASP9**[1391], **CCNA2**[1392], CDC27, CDK1, CHIA, CLEC11A, **CSNK2A1**[1393], DFNA5,

		**DIABLO**[1394], **EED**[256], EXOC3, **EXOC4**[1395], FAM175B, FAM58A, FIGF, **HERC2**[16], HOXD10, **HTRA2**[1391], IAP, **KIF22**[43], **KIF23**[1395], LLGL2, **MAP2K1**[1395], MCPH1, MOS, MYH13, NPHP4, **NUDC**[266], NUDCD2, NUDCD3, PLK1, POU5F1, PRSS45, RAB8B, **RACK1**[213], RASSF2, **RNF41**[1391], RPGR, **RPGRIP1L**[266], RTBDN, **SIRT7**[414], TBC1D22A, TEX35, **TP53**[1396], UBC, UBE2, UBE2D1, Ubiquitin, UNC45A, **UNK**[502], **USP8**[1395], VAV2, **VCL**[213], WDR4, WDR48
135	MOBP	**APP**[247], **ATN1**[198], **CACNA1A**[1200], CACNA1E, **HTT**[198], KRTAP10-1, **KRTAP10-3**[201], KRTAP10-5, **KRTAP10-7**[201], **KRTAP10-9**[201], MOBP, TCF7L2, UBC
136	SCFD1	**AGO1**[1059], **AGO2**[1059], **AKAP8**[1059], **APP**BP2, ATP6AP2, BET1, BET1L, **BNIP1**[1397], **CLTC**[1059], COG3, COG4, COG7, COG8, CUL5, DKKL1, DLST, **EED**[256], **EGFR**[515], EPRS, EVC2, **FBF1**[342], **GOLGA2**[1398], GOLGB1, GOSR1, GOSR2, **HNF4A**[18], HSP90AA1, **HSP90AB1**[1059], HSPA8, **HSPA9**[1059], **KAT5**[1399], **LRRK2**[1400], MCM2, **MDN1**[1059], MOV10, NBAS, NFKBIA, **NSF**[334], **NTRK1**[172], NXF1, **PDGFRA**[43], **PKP1**[254], PPBP, PRKDC, RAB5C, RINT1, **SCFD1**[254], SEC22A, **SEC22B**[16], **SOX2**[693], **SRPK1**[1059], **STAU1**[501], **STX18**[16], STX5, **STX6**[174], STXBP5L, TLN1, **TMED2**[43], **TMEM17**[342], **TMEM216**[342], USE1, USO1, USP22, **VCL**[493], VCP, **VIM**[1059], **XRN2**[781], YKT6, ZW10
137	C21ORF2	ATOX1, C21orf2, **CEP104**[342], **CEP290**[342], **DCAF7**[343], GMCL1, **KCTD17**[16], **KCTD5**[343], **NEK1**[343], **NEK5**[267], **PPP2R1A**[343], **RAF1**[267], **RPGRIP1L**[343], **SKAP1**[16], **SPATA7**[343], **TEKT4**[16], **VPS26B**[16], VTN, XPO1
138	ATRN	ASIP, ATRN, BTNL8, COPS5, FBXO6, **PTPRK**[16], **XRN2**[781], ZNF408
139	WDR49	NO INTERACTIONS
140	C1QTNF7	**BAG3**[327], C1QTNF7
141	ZSCAN5B	NO INTERACTIONS
142	POPLDIP2	ALAS1, **APP**[247], ASCC2, **C1QBP**[43], CEACAM1, CPE, **CUL3**[161], CYBA, **EGFR**[515], HDAC6, MAD2L2, MCM2, **MED23**[1088], MOV10, MRPL58, **MTUS2**[201], NXF1, **PCNA**[1401], **PDPK1**[308], POLD1, POLD2, POLDIP2, POLL, POLN, **PPP2R1A**[549], **PTEN**[949], REV1, SELENBP1, **SHMT2**[374], **SMAD6**[554], TAB1, **TP53**[549], **TSC22D1**[177]
143	KCNN1	Calmodulin, **GRB2**[527], KCNN1, Kcnn2, MAPK3, potassium channel
144	ANXA11	ALG2, ANXA11, **ATP4A**[1361], **BAG3**[327], CCDC8, CDC5L, **CEP55**[201], CUL5, **EGFR**[1402], ENO2, EWSR1, HLA-DRA, HLA-DRB1, HLA-DRB3, HLA-DRB4, HLA-DRB5, **HNRNPH3**[29], **KIF23**[1403], MUS81, **NTRK1**[147], **PDCD6**[1404], **PLSCR1**[5], **PTK2B**[1402], **RACGAP1**[49], S100A6, SEPT9, **SIRT7**[313], **TFG**[162], **TUBB2A**[1403], ZDHHC17
145	ATXN1	**ANP32A**[1405], ACOT7, ADCY6, ADD3, AHDC1, **ANKHD1**[159]/ANKHD1-EIF4EBP3, APBB1, ARHGAP32, ARID5A, ASNS, **ATF5**[1406], **ATP1A3**[159], **ATP6V0D1**[159], **ATXN1**[162], ATXN2, ATXN1L, **ATXN2L**[159], BAALC, **BASP1**[1406], BATF2, BEND2,BTG3, C1orf94, C2orf27A/C2orf27B, CAMK2B, CCDC136, CCNK, CDIP1, **CDK6**[159], **CFL1**[159], CHRNA7, CIC, **CIRBP**[159], CLASRP, CLCN2, CLEC4F, **COIL**[159], CPSF7, **CREM**[1406], **CRIP2**[1406], **CRK**[159], **CRY2**[1406], **CST3**[159], **DAZAP2**[159], DCTN3, DHRSX, DHX37, DIXDC1, **DMPK**[159], DNAJA3, DOCK5, DZIP3, **EFEMP2**[159], EHMT1, EIF1AY, **EIF1B**[1406], EIF3F, EIF4ENIF1, ELP5, ENTPD6, ESRP1, **ESRRA**[1406], ETV4, FAM193B, FAM46A, FAM46B, FAR1, FAT4, FDPS, **FHL2**[159], FOSL1, **FUBP3**[1406], **FYN**[159], GAPDH, GATAD1, GCA, GCFC2, GGA2, GMEB2, GPATCH8, GSPT1, **HDAC3**[1405], **HDAC4**[1407], HES1, HEY2, **HEYL**[1406], **HIST1H3A**[1406], HIVEP1, HNRNPLL, HPCAL1, HSFX1/HSFX2, **HSPA4**[1408], HSPA8, HSPA1A/HSPA1B, HYPM, IGF2R, IL34, ILVBL, **IMMT**[1406], IPO8, IST1, **ITGB4**[159], **KAT5**[1409], KAT6A, KCNAB2, KCTD15, KIAA2026, KIAA1549L, **KIF22**[1406], **KLF11**[159], **KLHL8**[159], KLHL12, **KMT2B**[159], LASP1, LDB1, LITAF, LOXL1, LPAR2, LRSAM1, MAGEB2, MAGEB6, MAGEB18, MATN2, **MBP**[159], MED15, METTL17, **MLST8**[1406], MSMO1, MSX2, MTERF4, MYEF2, MYO18A, NARS, NCAM1, **NCOR1**[159], **NCOR2**[1410], NLK, **NOVA1**[306], NPHP3, **NR4A1**[159], **NUDT21**[159], NUMBL, NUTM2A/NUTM2B, OAZ1, OPCML, OTX2, PEPD, PHPT1, **PIAS1**[1406], PIM2, PLEKHA5, PLEKHB1, **PML**[1411], POM121/POM121C, POMP, PPAT, **PPP6R2**[159], PQBP1, PRDM1, PRR20A (includes others), **PRRC2A**[159], PRRC2B, PSMC3, PSPH, **PTGDS**[159], PUM1, QKI, QRICH1, R3HDM1, R3HDM2, **RAD54L2**[159], RAI2, RAPGEF1, **RAPGEF4**[159], **RBFOX1**[159], **RBFOX2**[159], RBM17, **RBM26**[1406],

		**RBPJ**[1412], **RBPMs**[159], RCC1L, **RCN1**[159], **REL**[162], RHOXF2/RHOXF2B, **RNF4**[1411], RNF31, **RORA**[1409], SAR1A, SEMA4G, SETD2, SETD6, SF1, SGMS1, SIX5, SLC25A51, SLC4A2, SLC51A, SLC6A13, SMARCC2, SPEN, **SREBF1**[159], SREBF2, ST6GALNAC6, STAC2, STAM2, **STUB1**[927], **SUGP2**[159], **SUMO1**[1413], SUPT20H, SV2A, SYBU, TBC1D5, TBX15, TCAP, TCEANC, TCTA, **TDP2**[1406], TMX2, TOLLIP, TOMM20, TOX4, TP53I11, **TRAF2**[159], TRIM32, TRIM38, **TRIP6**[159], TSC1, TTC19, **U2AF2**[159], UBAP2L, UBE2E1, UBE2E3, **UBE2I**[159], UBQLN4, UHRF2, UHRF1BP1L, **UNK**[159], USP7, USP54, VCP, VSNL1, VSTM2L, WASHC2A/WASHC2C, **WNK1**[159], WNK2, YWHAE, YWHAZ, YY1AP1, ZBTB32, ZC3H10, ZC3H7B, ZCCHC13, ZHX1, ZHX2, ZHX3,ZNF276, ZNF488, ZNF609, ZNF804A, ZSCAN1, ZSWIM8, ZXDC, ZYX
146	ERLIN1	ADRA1D, AGR3, **AMFR**[819], ASB11, **BMPR1A**[49], **C1QBP**[49], C6orf120, CALML3, CD2AP, **CFTR**[299], **CHMP4B**[49], **CKAP5**[49], COX15, CUL7, DBN1, DUSP3, EDEM3, ERLIN1, **ERLIN2**[819], FA2H, **FAF2**[819], FANCD2, FBXO6, **GOLT1B**[49], **HNF4A**[24], **INSIG1**[1414], **ITPR1**[1415], **Ktn1**[49], MYEF2, **NTRK1**[147], PKN2, RAB5C, RAB7A, **RMDN3**[49], RNF139, RNF170, **RPGRIP1L**[257], SEC22A, SPAST, STOM, **SUZ12**[194], SYVN1, TMED2, TMEM199, TMEM63B, TMUB2, TRAF6, TRIM25, **TSG101**[49], UBAC2, UBC, **UFL1**[49], VAPA, VDAC1, XRCC3
147	TIA1	AFF1, AFF4, **AFG3L2**[38], **AIMP2**[38], **AK1**[49], BCOR, BMI1, BRD9, CAND1, CCDC82, CEP85, COPS5, CUL1, **CUL2**[141], **CUL3**[141], CUL5, **CUL4A**[141], CXXC4, **DCUN1D1**[141], DPPA4, EFR3A, **ELAVL1**[1416], ELL2, ESR1, **ESYT2**[38], FASTK, FMNL1, FN1, **G3BP1**[144], GPN3, **GRB7**[1416], **HNF4A**[24], HS6ST2, IRS2, ITGA4, KRT17, MAP3K1, **MAP3K3**[38], MLLT1, MLLT3, **NANOG**[328], NBEAL2, **NCOA3**[1417], NELFCD, NETO2, **NTRK1**[147], POLK, POU5F1, PPP4R3A, **PTK2**[1416], PUS7, **RAD51**[38], RICTOR, SF1, SLC4A2, **SMN1**[49]/SMN2, **SOX2**[505], **SRSF1**[1418], **TIA1**[1419], TMEM168, **TOB1**[455], TRMT6, **VCAM1**[316], WDHD1, ZNF35, ZNFX1

REFERENCES:

1. Zhou A, Carrell RW, Murphy MP, et al. A redox switch in angiotensinogen modulates angiotensin release. Nature 2010;468:108-111.
2. Jain S, Tang X, Narayanan CS, et al. Angiotensinogen gene polymorphism at -217 affects basal promoter activity and is associated with hypertension in African-Americans. J Biol Chem 2002;277:36889-36896.
3. Wang XZ, Kuroda M, Sok J, et al. Identification of novel stress-induced genes downstream of chop. EMBO J 1998;17:3619-3630.
4. Sahar S, Reddy MA, Wong C, Meng L, Wang M, Natarajan R. Cooperation of SRC-1 and p300 with NF-kappaB and CREB in angiotensin II-induced IL-6 expression in vascular smooth muscle cells. Arterioscler Thromb Vasc Biol 2007;27:1528-1534.
5. Rual JF, Venkatesan K, Hao T, et al. Towards a proteome-scale map of the human protein-protein interaction network. Nature 2005;437:1173-1178.
6. Abdul-Hafez A, Shu R, Uhal BD. JunD and HIF-1alpha mediate transcriptional activation of angiotensinogen by TGF-beta1 in human lung fibroblasts. FASEB J 2009;23:1655-1662.
7. Jain S, Li Y, Patil S, Kumar A. HNF-1alpha plays an important role in IL-6-induced expression of the human angiotensinogen gene. Am J Physiol Cell Physiol 2007;293:C401-410.
8. Wei CC, Zhang SL, Chen YW, et al. Heterogeneous nuclear ribonucleoprotein K modulates angiotensinogen gene expression in kidney cells. J Biol Chem 2006;281:25344-25355.

9. Son YJ, Hur MK, Ryu BJ, et al. TTF-1, a homeodomain-containing transcription factor, participates in the control of body fluid homeostasis by regulating angiotensinogen gene transcription in the rat subfornical organ. J Biol Chem 2003;278:27043-27052.

10. Ohishi K, Carmines PK, Inscho EW, Navar LG. EDRF-angiotensin II interactions in rat juxtamedullary afferent and efferent arterioles. Am J Physiol 1992;263:F900-906.

11. Mateos L, Ismail MA, Gil-Bea FJ, et al. Side chain-oxidized oxysterols regulate the brain renin-angiotensin system through a liver X receptor-dependent mechanism. J Biol Chem 2011;286:25574-25585.

12. Yanai K, Hirota K, Taniguchi-Yanai K, et al. Regulated expression of human angiotensinogen gene by hepatocyte nuclear factor 4 and chicken ovalbumin upstream promoter-transcription factor. J Biol Chem 1999;274:34605-34612.

13. Sherman CT, Brasier AR. Role of signal transducers and activators of transcription 1 and -3 in inducible regulation of the human angiotensinogen gene by interleukin-6. Mol Endocrinol 2001;15:441-457.

14. Kathiresan T, Harvey M, Orchard S, Sakai Y, Sokolowski B. A protein interaction network for the large conductance Ca(2+)-activated K(+) channel in the mouse cochlea. Mol Cell Proteomics 2009;8:1972-1987.

15. Taipale M, Tucker G, Peng J, et al. A quantitative chaperone interaction network reveals the architecture of cellular protein homeostasis pathways. Cell 2014;158:434-448.

16. Huttlin EL, Bruckner RJ, Paulo JA, et al. Architecture of the human interactome defines protein communities and disease networks. Nature 2017;545:505-509.

17. Lai C, Xie C, McCormack SG, et al. Amyotrophic lateral sclerosis 2-deficiency leads to neuronal degeneration in amyotrophic lateral sclerosis through altered AMPA receptor trafficking. J Neurosci 2006;26:11798-11806.

18. Odom DT, Zizlsperger N, Gordon DB, et al. Control of pancreas and liver gene expression by HNF transcription factors. Science 2004;303:1378-1381.

19. Otomo A, Kunita R, Suzuki-Utsunomiya K, Ikeda JE, Hadano S. Defective relocalization of ALS2/alsin missense mutants to Rac1-induced macropinosomes accounts for loss of their cellular function and leads to disturbed amphisome formation. FEBS Lett 2011;585:730-736.

20. Kanekura K, Hashimoto Y, Niikura T, Aiso S, Matsuoka M, Nishimoto I. Alsin, the product of ALS2 gene, suppresses SOD1 mutant neurotoxicity through RhoGEF domain by interacting with SOD1 mutants. J Biol Chem 2004;279:19247-19256.

21. Enunlu I, Ozansoy M, Basak AN. Alfa-class prefoldin protein UXT is a novel interacting partner of Amyotrophic Lateral Sclerosis 2 (Als2) protein. Biochem Biophys Res Commun 2011;413:471-475.

22. Jin J, Smith FD, Stark C, et al. Proteomic, functional, and domain-based analysis of in vivo 14-3-3 binding proteins involved in cytoskeletal regulation and cellular organization. Curr Biol 2004;14:1436-1450.

23. Hu H, Gao X, Sun Y, Zhou J, Yang M, Xu Z. Alpha-actinin-2, a cytoskeletal protein, binds to angiogenin. Biochem Biophys Res Commun 2005;329:661-667.

24. Paudel N, Sadagopan S, Balasubramanian S, Chandran B. Kaposi's sarcoma-associated herpesvirus latency-associated nuclear antigen and angiogenin interact with common host proteins, including annexin A2, which is essential for survival of latently infected cells. J Virol 2012;86:1589-1607.

25. Xia W, Fu W, Cai L, et al. Identification and characterization of FHL3 as a novel angiogenin-binding partner. Gene 2012;504:233-237.

26. Sadagopan S, Veettil MV, Chakraborty S, et al. Angiogenin functionally interacts with p53 and regulates p53-mediated apoptosis and cell survival. Oncogene 2012;31:4835-4847.

27. Kwak MK, Wakabayashi N, Itoh K, Motohashi H, Yamamoto M, Kensler TW. Modulation of gene expression by cancer chemopreventive dithiolethiones through the Keap1-Nrf2 pathway. Identification of novel gene clusters for cell survival. J Biol Chem 2003;278:8135-8145.
28. Zhu J, Sheng J, Dong H, Kang L, Ang J, Xu Z. Phospholipid scramblase 1 functionally interacts with angiogenin and regulates angiogenin-enhanced rRNA transcription. Cell Physiol Biochem 2013;32:1695-1706.
29. Stelzl U, Worm U, Lalowski M, et al. A human protein-protein interaction network: a resource for annotating the proteome. Cell 2005;122:957-968.
30. Krimbou L, Tremblay M, Davignon J, Cohn JS. Association of apolipoprotein E with alpha2-macroglobulin in human plasma. J Lipid Res 1998;39:2373-2386.
31. Zhou M, Lucas DA, Chan KC, et al. An investigation into the human serum "interactome". Electrophoresis 2004;25:1289-1298.
32. Spires TL, Hyman BT. Transgenic models of Alzheimer's disease: learning from animals. NeuroRx 2005;2:423-437.
33. Soler-Lopez M, Zanzoni A, Lluis R, Stelzl U, Aloy P. Interactome mapping suggests new mechanistic details underlying Alzheimer's disease. Genome Res 2011;21:364-376.
34. Gutman CR, Strittmatter WJ, Weisgraber KH, Matthew WD. Apolipoprotein E binds to and potentiates the biological activity of ciliary neurotrophic factor. J Neurosci 1997;17:6114-6121.
35. Lemberger T, Parkitna JR, Chai M, Schutz G, Engblom D. CREB has a context-dependent role in activity-regulated transcription and maintains neuronal cholesterol homeostasis. FASEB J 2008;22:2872-2879.
36. Rothbard JB, Kurnellas MP, Brownell S, et al. Therapeutic effects of systemic administration of chaperone alphaB-crystallin associated with binding proinflammatory plasma proteins. J Biol Chem 2012;287:9708-9721.
37. Raftery M, Campbell R, Glaros EN, et al. Phosphorylation of apolipoprotein-E at an atypical protein kinase CK2 PSD/E site in vitro. Biochemistry 2005;44:7346-7353.
38. Ishiguro A, Ideta M, Mikoshiba K, Chen DJ, Aruga J. ZIC2-dependent transcriptional regulation is mediated by DNA-dependent protein kinase, poly(ADP-ribose) polymerase, and RNA helicase A. J Biol Chem 2007;282:9983-9995.
39. Rougeron V, Woods CM, Tiedje KE, et al. Epistatic Interactions between apolipoprotein E and hemoglobin S Genes in regulation of malaria parasitemia. PLoS One 2013;8:e76924.
40. Yao MW, Lim H, Schust DJ, et al. Gene expression profiling reveals progesterone-mediated cell cycle and immunoregulatory roles of Hoxa-10 in the preimplantation uterus. Mol Endocrinol 2003;17:610-627.
41. Liu W, Seto J, Sibille E, Toth M. The RNA binding domain of Jerky consists of tandemly arranged helix-turn-helix/homeodomain-like motifs and binds specific sets of mRNAs. Mol Cell Biol 2003;23:4083-4093.
42. Turchinovich G, Vu TT, Frommer F, et al. Programming of marginal zone B-cell fate by basic Kruppel-like factor (BKLF/KLF3). Blood 2011;117:3780-3792.
43. Hein MY, Hubner NC, Poser I, et al. A human interactome in three quantitative dimensions organized by stoichiometries and abundances. Cell 2015;163:712-723.
44. Carter CJ. Convergence of genes implicated in Alzheimer's disease on the cerebral cholesterol shuttle: APP, cholesterol, lipoproteins, and atherosclerosis. Neurochem Int 2007;50:12-38.
45. Fleming LM, Weisgraber KH, Strittmatter WJ, Troncoso JC, Johnson GV. Differential binding of apolipoprotein E isoforms to tau and other cytoskeletal proteins. Exp Neurol 1996;138:252-260.
46. Yue L, Christman JW, Mazzone T. Tumor necrosis factor-alpha-mediated suppression of adipocyte apolipoprotein E gene transcription: primary role for the nuclear factor (NF)-kappaB pathway and NFkappaB p50. Endocrinology 2008;149:4051-4058.

47. Laffitte BA, Repa JJ, Joseph SB, et al. LXRs control lipid-inducible expression of the apolipoprotein E gene in macrophages and adipocytes. Proc Natl Acad Sci U S A 2001;98:507-512.
48. Lu R, Ito J, Iwamoto N, Nishimaki-Mogami T, Yokoyama S. FGF-1 induces expression of LXRalpha and production of 25-hydroxycholesterol to upregulate the apoE gene in rat astrocytes. J Lipid Res 2009;50:1156-1164.
49. Pearen MA, Muscat GE. Minireview: Nuclear hormone receptor 4A signaling: implications for metabolic disease. Mol Endocrinol 2010;24:1891-1903.
50. Cheng X, Guo S, Liu Y, et al. Ablation of promyelocytic leukemia protein (PML) re-patterns energy balance and protects mice from obesity induced by a Western diet. J Biol Chem 2013;288:29746-29759.
51. Shih DM, Xia YR, Wang XP, et al. Combined serum paraoxonase knockout/apolipoprotein E knockout mice exhibit increased lipoprotein oxidation and atherosclerosis. J Biol Chem 2000;275:17527-17535.
52. Lau P, Nixon SJ, Parton RG, Muscat GE. RORalpha regulates the expression of genes involved in lipid homeostasis in skeletal muscle cells: caveolin-3 and CPT-1 are direct targets of ROR. J Biol Chem 2004;279:36828-36840.
53. Bultel-Brienne S, Lestavel S, Pilon A, et al. Lipid free apolipoprotein E binds to the class B Type I scavenger receptor I (SR-BI) and enhances cholesteryl ester uptake from lipoproteins. J Biol Chem 2002;277:36092-36099.
54. Christensen DJ, Ohkubo N, Oddo J, et al. Apolipoprotein E and peptide mimetics modulate inflammation by binding the SET protein and activating protein phosphatase 2A. J Immunol 2011;186:2535-2542.
55. Carlo AS, Gustafsen C, Mastrobuoni G, et al. The pro-neurotrophin receptor sortilin is a major neuronal apolipoprotein E receptor for catabolism of amyloid-beta peptide in the brain. J Neurosci 2013;33:358-370.
56. Sasaki I, Hoshino K, Sugiyama T, et al. Spi-B is critical for plasmacytoid dendritic cell function and development. Blood 2012;120:4733-4743.
57. Trusca VG, Fuior EV, Florea IC, Kardassis D, Simionescu M, Gafencu AV. Macrophage-specific up-regulation of apolipoprotein E gene expression by STAT1 is achieved via long range genomic interactions. J Biol Chem 2011;286:13891-13904.
58. Schroder AJ, Pavlidis P, Arimura A, Capece D, Rothman PB. Cutting edge: STAT6 serves as a positive and negative regulator of gene expression in IL-4-stimulated B lymphocytes. J Immunol 2002;168:996-1000.
59. Liu T, Laurell C, Selivanova G, Lundeberg J, Nilsson P, Wiman KG. Hypoxia induces p53-dependent transactivation and Fas/CD95-dependent apoptosis. Cell Death Differ 2007;14:411-421.
60. Wagner S, Hess MA, Ormonde-Hanson P, et al. A broad role for the zinc finger protein ZNF202 in human lipid metabolism. J Biol Chem 2000;275:15685-15690.
61. Kerkhofs S, Dubois V, De Gendt K, et al. A role for selective androgen response elements in the development of the epididymis and the androgen control of the 5alpha reductase II gene. FASEB J 2012;26:4360-4372.
62. Shatkina L, Mink S, Rogatsch H, et al. The cochaperone Bag-1L enhances androgen receptor action via interaction with the NH2-terminal region of the receptor. Mol Cell Biol 2003;23:7189-7197.
63. Hu XD, Meng QH, Xu JY, et al. BTG2 is an LXXLL-dependent co-repressor for androgen receptor transcriptional activity. Biochem Biophys Res Commun 2011;404:903-909.
64. Leung KK, Hause RJ, Jr., Barkinge JL, Ciaccio MF, Chuu CP, Jones RB. Enhanced prediction of Src homology 2 (SH2) domain binding potentials using a fluorescence polarization-derived c-Met, c-Kit, ErbB, and androgen receptor interactome. Mol Cell Proteomics 2014;13:1705-1723.

65. Kallio PJ, Poukka H, Moilanen A, Janne OA, Palvimo JJ. Androgen receptor-mediated transcriptional regulation in the absence of direct interaction with a specific DNA element. Mol Endocrinol 1995;9:1017-1028.
66. Wang RS, Yeh S, Chen LM, et al. Androgen receptor in sertoli cell is essential for germ cell nursery and junctional complex formation in mouse testes. Endocrinology 2006;147:5624-5633.
67. Dedhar S, Rennie PS, Shago M, et al. Inhibition of nuclear hormone receptor activity by calreticulin. Nature 1994;367:480-483.
68. Tsai WC, Yang LY, Chen YC, et al. Ablation of the androgen receptor gene modulates atrial electrophysiology and arrhythmogenesis with calcium protein dysregulation. Endocrinology 2013;154:2833-2842.
69. Wellington CL, Ellerby LM, Hackam AS, et al. Caspase cleavage of gene products associated with triplet expansion disorders generates truncated fragments containing the polyglutamine tract. J Biol Chem 1998;273:9158-9167.
70. Sun F, Chen HG, Li W, et al. Androgen receptor splice variant AR3 promotes prostate cancer via modulating expression of autocrine/paracrine factors. J Biol Chem 2014;289:1529-1539.
71. Lindqvist J, Imanishi SY, Torvaldson E, et al. Cyclin-dependent kinase 5 acts as a critical determinant of AKT-dependent proliferation and regulates differential gene expression by the androgen receptor in prostate cancer cells. Mol Biol Cell 2015;26:1971-1984.
72. Lee DK, Duan HO, Chang C. Androgen receptor interacts with the positive elongation factor P-TEFb and enhances the efficiency of transcriptional elongation. J Biol Chem 2001;276:9978-9984.
73. Reebye V, Bevan CL, Nohadani M, Hajitou A, Habib NA, Mintz PJ. Interaction between AR signalling and CRKL bypasses casodex inhibition in prostate cancer. Cell Signal 2010;22:1874-1881.
74. Lamia KA, Papp SJ, Yu RT, et al. Cryptochromes mediate rhythmic repression of the glucocorticoid receptor. Nature 2011;480:552-556.
75. Ohtake F, Baba A, Takada I, et al. Dioxin receptor is a ligand-dependent E3 ubiquitin ligase. Nature 2007;446:562-566.
76. Hulkko SM, Wakui H, Zilliacus J. The pro-apoptotic protein death-associated protein 3 (DAP3) interacts with the glucocorticoid receptor and affects the receptor function. Biochem J 2000;349 Pt 3:885-893.
77. Wafa LA, Cheng H, Rao MA, et al. Isolation and identification of L-dopa decarboxylase as a protein that binds to and enhances transcriptional activity of the androgen receptor using the repressed transactivator yeast two-hybrid system. Biochem J 2003;375:373-383.
78. Shatnawi A, Norris JD, Chaveroux C, et al. ELF3 is a repressor of androgen receptor action in prostate cancer cells. Oncogene 2014;33:862-871.
79. Chymkowitch P, Le May N, Charneau P, Compe E, Egly JM. The phosphorylation of the androgen receptor by TFIIH directs the ubiquitin/proteasome process. EMBO J 2011;30:468-479.
80. Yu J, Yu J, Mani RS, et al. An integrated network of androgen receptor, polycomb, and TMPRSS2-ERG gene fusions in prostate cancer progression. Cancer Cell 2010;17:443-454.
81. Muller JM, Isele U, Metzger E, et al. FHL2, a novel tissue-specific coactivator of the androgen receptor. EMBO J 2000;19:359-369.
82. Schulke JP, Wochnik GM, Lang-Rollin I, et al. Differential impact of tetratricopeptide repeat proteins on the steroid hormone receptors. PLoS One 2010;5:e11717.
83. Cai Y, Balli D, Ustiyan V, et al. Foxm1 expression in prostate epithelial cells is essential for prostate carcinogenesis. J Biol Chem 2013;288:22527-22541.
84. Abel MH, Baker PJ, Charlton HM, et al. Spermatogenesis and sertoli cell activity in mice lacking sertoli cell receptors for follicle-stimulating hormone and androgen. Endocrinology 2008;149:3279-3285.
85. Chauhan S, Kunz S, Davis K, et al. Androgen control of cell proliferation and cytoskeletal reorganization in human fibrosarcoma cells: role of RhoB signaling. J Biol Chem 2004;279:937-944.

86. Yang JL, Zhang CP, Li L, et al. Testosterone induces redistribution of forkhead box-3a and down-regulation of growth and differentiation factor 9 messenger ribonucleic acid expression at early stage of mouse folliculogenesis. Endocrinology 2010;151:774-782.
87. Li N, Chen M, Truong S, Yan C, Buttyan R. Determinants of Gli2 co-activation of wildtype and naturally truncated androgen receptors. Prostate 2014;74:1400-1410.
88. Loew A, Ho YK, Blundell T, Bax B. Phosducin induces a structural change in transducin beta gamma. Structure 1998;6:1007-1019.
89. Hong H, Kohli K, Trivedi A, Johnson DL, Stallcup MR. GRIP1, a novel mouse protein that serves as a transcriptional coactivator in yeast for the hormone binding domains of steroid receptors. Proc Natl Acad Sci U S A 1996;93:4948-4952.
90. Ikeda H, Serria MS, Kakizaki I, et al. Activation of mouse Pi-class glutathione S-transferase gene by Nrf2(NF-E2-related factor 2) and androgen. Biochem J 2002;364:563-570.
91. Andersen RJ, Mawji NR, Wang J, et al. Regression of castrate-recurrent prostate cancer by a small-molecule inhibitor of the amino-terminus domain of the androgen receptor. Cancer Cell 2010;17:535-546.
92. Rong J, Li SH, Li XJ. Regulation of intracellular HAP1 trafficking. J Neurosci Res 2007;85:3025-3029.
93. Li Y, Zhang Q, Yin X, et al. Generation of iPSCs from mouse fibroblasts with a single gene, Oct4, and small molecules. Cell Res 2011;21:196-204.
94. Karvonen U, Janne OA, Palvimo JJ. Androgen receptor regulates nuclear trafficking and nuclear domain residency of corepressor HDAC7 in a ligand-dependent fashion. Exp Cell Res 2006;312:3165-3183.
95. Moilanen AM, Karvonen U, Poukka H, Janne OA, Palvimo JJ. Activation of androgen receptor function by a novel nuclear protein kinase. Mol Biol Cell 1998;9:2527-2543.
96. Boonyaratanakornkit V, Melvin V, Prendergast P, et al. High-mobility group chromatin proteins 1 and 2 functionally interact with steroid hormone receptors to enhance their DNA binding in vitro and transcriptional activity in mammalian cells. Mol Cell Biol 1998;18:4471-4487.
97. Morgan EA, Nguyen SB, Scott V, Stadler HS. Loss of Bmp7 and Fgf8 signaling in Hoxa13-mutant mice causes hypospadia. Development 2003;130:3095-3109.
98. Zoubeidi A, Zardan A, Beraldi E, et al. Cooperative interactions between androgen receptor (AR) and heat-shock protein 27 facilitate AR transcriptional activity. Cancer Res 2007;67:10455-10465.
99. Katsuno M, Sang C, Adachi H, et al. Pharmacological induction of heat-shock proteins alleviates polyglutamine-mediated motor neuron disease. Proc Natl Acad Sci U S A 2005;102:16801-16806.
100. Qi J, Tripathi M, Mishra R, et al. The E3 ubiquitin ligase Siah2 contributes to castration-resistant prostate cancer by regulation of androgen receptor transcriptional activity. Cancer Cell 2013;23:332-346.
101. Xie S, Lin HK, Ni J, et al. Regulation of interleukin-6-mediated PI3K activation and neuroendocrine differentiation by androgen signaling in prostate cancer LNCaP cells. Prostate 2004;60:61-67.
102. Mehraein-Ghomi F, Kegel SJ, Church DR, et al. Targeting androgen receptor and JunD interaction for prevention of prostate cancer progression. Prostate 2014;74:792-803.
103. Gaughan L, Logan IR, Cook S, Neal DE, Robson CN. Tip60 and histone deacetylase 1 regulate androgen receptor activity through changes to the acetylation status of the receptor. J Biol Chem 2002;277:25904-25913.
104. van de Wijngaart DJ, Dubbink HJ, Molier M, de Vos C, Trapman J, Jenster G. Functional screening of FxxLF-like peptide motifs identifies SMARCD1/BAF60a as an androgen receptor cofactor that modulates TMPRSS2 expression. Mol Endocrinol 2009;23:1776-1786.
105. Cloke B, Huhtinen K, Fusi L, et al. The androgen and progesterone receptors regulate distinct gene networks and cellular functions in decidualizing endometrium. Endocrinology 2008;149:4462-4474.
106. Zhang S, Li W, Zhu C, et al. Sertoli cell-specific expression of metastasis-associated protein 2 (MTA2) is required for transcriptional regulation of the follicle-stimulating hormone receptor (FSHR) gene during spermatogenesis. J Biol Chem 2012;287:40471-40483.

107. Ishitani K, Yoshida T, Kitagawa H, Ohta H, Nozawa S, Kato S. p54nrb acts as a transcriptional coactivator for activation function 1 of the human androgen receptor. Biochem Biophys Res Commun 2003;306:660-665.
108. Lin HK, Yeh S, Kang HY, Chang C. Akt suppresses androgen-induced apoptosis by phosphorylating and inhibiting androgen receptor. Proc Natl Acad Sci U S A 2001;98:7200-7205.
109. Goo YH, Na SY, Zhang H, et al. Interactions between activating signal cointegrator-2 and the tumor suppressor retinoblastoma in androgen receptor transactivation. J Biol Chem 2004;279:7131-7135.
110. Cui J, Yang Y, Zhang C, et al. FBI-1 functions as a novel AR co-repressor in prostate cancer cells. Cell Mol Life Sci 2011;68:1091-1103.
111. Corominas R, Yang X, Lin GN, et al. Protein interaction network of alternatively spliced isoforms from brain links genetic risk factors for autism. Nat Commun 2014;5:3650.
112. Nadiminty N, Tummala R, Liu C, et al. NF-kappaB2/p52 induces resistance to enzalutamide in prostate cancer: role of androgen receptor and its variants. Mol Cancer Ther 2013;12:1629-1637.
113. Gobinet J, Auzou G, Nicolas JC, Sultan C, Jalaguier S. Characterization of the interaction between androgen receptor and a new transcriptional inhibitor, SHP. Biochemistry 2001;40:15369-15377.
114. Song CH, Lee HJ, Park E, Lee K. The chicken ovalbumin upstream promoter-transcription factor II negatively regulates the transactivation of androgen receptor in prostate cancer cells. PLoS One 2012;7:e49026.
115. Zhang Y, Fondell JD, Wang Q, et al. Repression of androgen receptor mediated transcription by the ErbB-3 binding protein, Ebp1. Oncogene 2002;21:5609-5618.
116. Tillman JE, Yuan J, Gu G, et al. DJ-1 binds androgen receptor directly and mediates its activity in hormonally treated prostate cancer cells. Cancer Res 2007;67:4630-4637.
117. Yeap BB, Voon DC, Vivian JP, et al. Novel binding of HuR and poly(C)-binding protein to a conserved UC-rich motif within the 3'-untranslated region of the androgen receptor messenger RNA. J Biol Chem 2002;277:27183-27192.
118. Hazra R, Jimenez M, Desai R, Handelsman DJ, Allan CM. Sertoli cell androgen receptor expression regulates temporal fetal and adult Leydig cell differentiation, function, and population size. Endocrinology 2013;154:3410-3422.
119. Gamble SC, Chotai D, Odontiadis M, et al. Prohibitin, a protein downregulated by androgens, represses androgen receptor activity. Oncogene 2007;26:1757-1768.
120. Kotaja N, Aittomaki S, Silvennoinen O, Palvimo JJ, Janne OA. ARIP3 (androgen receptor-interacting protein 3) and other PIAS (protein inhibitor of activated STAT) proteins differ in their ability to modulate steroid receptor-dependent transcriptional activation. Mol Endocrinol 2000;14:1986-2000.
121. Metzger E, Muller JM, Ferrari S, Buettner R, Schule R. A novel inducible transactivation domain in the androgen receptor: implications for PRK in prostate cancer. EMBO J 2003;22:270-280.
122. Berwick DC, Diss JK, Budhram-Mahadeo VS, Latchman DS. A simple technique for the prediction of interacting proteins reveals a direct Brn-3a-androgen receptor interaction. J Biol Chem 2010;285:15286-15295.
123. Park SY, Yu X, Ip C, Mohler JL, Bogner PN, Park YM. Peroxiredoxin 1 interacts with androgen receptor and enhances its transactivation. Cancer Res 2007;67:9294-9303.
124. Koh SS, Chen D, Lee YH, Stallcup MR. Synergistic enhancement of nuclear receptor function by p160 coactivators and two coactivators with protein methyltransferase activities. J Biol Chem 2001;276:1089-1098.
125. O'Shaughnessy PJ, Johnston H, Willerton L, Baker PJ. Failure of normal adult Leydig cell development in androgen-receptor-deficient mice. J Cell Sci 2002;115:3491-3496.
126. Wang LG, Johnson EM, Kinoshita Y, et al. Androgen receptor overexpression in prostate cancer linked to Pur alpha loss from a novel repressor complex. Cancer Res 2008;68:2678-2688.

127. Rigas AC, Ozanne DM, Neal DE, Robson CN. The scaffolding protein RACK1 interacts with androgen receptor and promotes cross-talk through a protein kinase C signaling pathway. J Biol Chem 2003;278:46087-46093.
128. Domanskyi A, Virtanen KT, Palvimo JJ, Janne OA. Biochemical characterization of androgen receptor-interacting protein 4. Biochem J 2006;393:789-795.
129. Hsiao PW, Lin DL, Nakao R, Chang C. The linkage of Kennedy's neuron disease to ARA24, the first identified androgen receptor polyglutamine region-associated coactivator. J Biol Chem 1999;274:20229-20234.
130. Wang J, Zhang W, Ji W, Liu X, Ouyang G, Xiao W. The von hippel-lindau protein suppresses androgen receptor activity. Mol Endocrinol 2014;28:239-248.
131. Moilanen AM, Poukka H, Karvonen U, Hakli M, Janne OA, Palvimo JJ. Identification of a novel RING finger protein as a coregulator in steroid receptor-mediated gene transcription. Mol Cell Biol 1998;18:5128-5139.
132. Kim JH, Lee JM, Nam HJ, et al. SUMOylation of pontin chromatin-remodeling complex reveals a signal integration code in prostate cancer cells. Proc Natl Acad Sci U S A 2007;104:20793-20798.
133. Cai Y, Dai T, Ao Y, et al. Cytochrome P450 genes are differentially expressed in female and male hepatocyte retinoid X receptor alpha-deficient mice. Endocrinology 2003;144:2311-2318.
134. Dong X, Sweet J, Challis JR, Brown T, Lye SJ. Transcriptional activity of androgen receptor is modulated by two RNA splicing factors, PSF and p54nrb. Mol Cell Biol 2007;27:4863-4875.
135. Schmidt EM, Gu S, Anagnostopoulou V, et al. Serum- and glucocorticoid-dependent kinase-1-induced cell migration is dependent on vinculin and regulated by the membrane androgen receptor. FEBS J 2012;279:1231-1242.
136. Zhu P, Baek SH, Bourk EM, et al. Macrophage/cancer cell interactions mediate hormone resistance by a nuclear receptor derepression pathway. Cell 2006;124:615-629.
137. Chen G, Nomura M, Morinaga H, et al. Modulation of androgen receptor transactivation by FoxH1. A newly identified androgen receptor corepressor. J Biol Chem 2005;280:36355-36363.
138. Li Y, Tian L, Ligr M, et al. Functional domains of androgen receptor coactivator p44/Mep50/WDR77and its interaction with Smad1. PLoS One 2013;8:e64663.
139. Hong CY, Suh JH, Kim K, et al. Modulation of androgen receptor transactivation by the SWI3-related gene product (SRG3) in multiple ways. Mol Cell Biol 2005;25:4841-4852.
140. Link KA, Balasubramaniam S, Sharma A, et al. Targeting the BAF57 SWI/SNF subunit in prostate cancer: a novel platform to control androgen receptor activity. Cancer Res 2008;68:4551-4558.
141. Liu LL, Xie N, Sun S, Plymate S, Mostaghel E, Dong X. Mechanisms of the androgen receptor splicing in prostate cancer cells. Oncogene 2014;33:3140-3150.
142. Cinar B, Collak FK, Lopez D, et al. MST1 is a multifunctional caspase-independent inhibitor of androgenic signaling. Cancer Res 2011;71:4303-4313.
143. Tavassoli P, Wafa LA, Cheng H, et al. TAF1 differentially enhances androgen receptor transcriptional activity via its N-terminal kinase and ubiquitin-activating and -conjugating domains. Mol Endocrinol 2010;24:696-708.
144. Yang YC, Chang LK. Role of TAF4 in transcriptional activation by Rta of Epstein-Barr Virus. PLoS One 2013;8:e54075.
145. Chen D, Lucey MJ, Phoenix F, et al. T:G mismatch-specific thymine-DNA glycosylase potentiates transcription of estrogen-regulated genes through direct interaction with estrogen receptor alpha. J Biol Chem 2003;278:38586-38592.
146. Cortes CJ, Miranda HC, Frankowski H, et al. Polyglutamine-expanded androgen receptor interferes with TFEB to elicit autophagy defects in SBMA. Nat Neurosci 2014;17:1180-1189.

147. Kikuchi M, Okumura F, Tsukiyama T, et al. TRIM24 mediates ligand-dependent activation of androgen receptor and is repressed by a bromodomain-containing protein, BRD7, in prostate cancer cells. Biochim Biophys Acta 2009;1793:1828-1836.
148. Hittelman AB, Burakov D, Iniguez-Lluhi JA, Freedman LP, Garabedian MJ. Differential regulation of glucocorticoid receptor transcriptional activation via AF-1-associated proteins. EMBO J 1999;18:5380-5388.
149. Welsh M, Saunders PT, Atanassova N, Sharpe RM, Smith LB. Androgen action via testicular peritubular myoid cells is essential for male fertility. FASEB J 2009;23:4218-4230.
150. Shiota M, Yokomizo A, Tada Y, et al. Castration resistance of prostate cancer cells caused by castration-induced oxidative stress through Twist1 and androgen receptor overexpression. Oncogene 2010;29:237-250.
151. McClurg UL, Cork DMW, Darby S, et al. Identification of a novel K311 ubiquitination site critical for androgen receptor transcriptional activity. Nucleic Acids Res 2017;45:1793-1804.
152. Faus H, Meyer HA, Huber M, Bahr I, Haendler B. The ubiquitin-specific protease USP10 modulates androgen receptor function. Mol Cell Endocrinol 2005;245:138-146.
153. Burska UL, Harle VJ, Coffey K, et al. Deubiquitinating enzyme Usp12 is a novel co-activator of the androgen receptor. J Biol Chem 2013;288:32641-32650.
154. Chen S, Chen K, Zhang Q, Cheng H, Zhou R. Regulation of the transcriptional activation of the androgen receptor by the UXT-binding protein VHL. Biochem J 2013;456:55-66.
155. Zhang C, Yeh S, Chen YT, et al. Oligozoospermia with normal fertility in male mice lacking the androgen receptor in testis peritubular myoid cells. Proc Natl Acad Sci U S A 2006;103:17718-17723.
156. Mayeur GL, Kung WJ, Martinez A, Izumiya C, Chen DJ, Kung HJ. Ku is a novel transcriptional recycling coactivator of the androgen receptor in prostate cancer cells. J Biol Chem 2005;280:10827-10833.
157. Titus MA, Tan JA, Gregory CW, et al. 14-3-3{eta} Amplifies Androgen Receptor Actions in Prostate Cancer. Clin Cancer Res 2009;15:7571-7581.
158. Keller BA, Volkening K, Droppelmann CA, Ang LC, Rademakers R, Strong MJ. Co-aggregation of RNA binding proteins in ALS spinal motor neurons: evidence of a common pathogenic mechanism. Acta Neuropathol 2012;124:733-747.
159. Lim J, Hao T, Shaw C, et al. A protein-protein interaction network for human inherited ataxias and disorders of Purkinje cell degeneration. Cell 2006;125:801-814.
160. Kaehler C, Isensee J, Nonhoff U, et al. Ataxin-2-like is a regulator of stress granules and processing bodies. PLoS One 2012;7:e50134.
161. Bennett EJ, Rush J, Gygi SP, Harper JW. Dynamics of Cullin-RING Ubiquitin Ligase Network Revealed by Systematic Quantitative Proteomics. Cell 2010;143:951-965.
162. Arbuckle MI, Komiyama NH, Delaney A, et al. The SH3 domain of postsynaptic density 95 mediates inflammatory pain through phosphatidylinositol-3-kinase recruitment. EMBO Rep 2010;11:473-478.
163. Nonis D, Schmidt MH, van de Loo S, et al. Ataxin-2 associates with the endocytosis complex and affects EGF receptor trafficking. Cell Signal 2008;20:1725-1739.
164. Johnston IM, Spence HJ, Winnie JN, et al. Regulation of a multigenic invasion programme by the transcription factor, AP-1: re-expression of a down-regulated gene, TSC-36, inhibits invasion. Oncogene 2000;19:5348-5358.
165. Rudra D, deRoos P, Chaudhry A, et al. Transcription factor Foxp3 and its protein partners form a complex regulatory network. Nat Immunol 2012;13:1010-1019.
166. Jain S, Wheeler JR, Walters RW, Agrawal A, Barsic A, Parker R. ATPase-Modulated Stress Granules Contain a Diverse Proteome and Substructure. Cell 2016;164:487-498.
167. Ralser M, Nonhoff U, Albrecht M, et al. Ataxin-2 and huntingtin interact with endophilin-A complexes to function in plastin-associated pathways. Hum Mol Genet 2005;14:2893-2909.

168. Zhao C, Denison C, Huibregtse JM, Gygi S, Krug RM. Human ISG15 conjugation targets both IFN-induced and constitutively expressed proteins functioning in diverse cellular pathways. Proc Natl Acad Sci U S A 2005;102:10200-10205.
169. Foskett JK. Inositol trisphosphate receptor Ca2+ release channels in neurological diseases. Pflugers Arch 2010;460:481-494.
170. Woods NT, Mesquita RD, Sweet M, et al. Charting the landscape of tandem BRCT domain-mediated protein interactions. Sci Signal 2012;5:rs6.
171. Wiedemeyer R, Westermann F, Wittke I, Nowock J, Schwab M. Ataxin-2 promotes apoptosis of human neuroblastoma cells. Oncogene 2003;22:401-411.
172. Emdal KB, Pedersen AK, Bekker-Jensen DB, et al. Temporal proteomics of NGF-TrkA signaling identifies an inhibitory role for the E3 ligase Cbl-b in neuroblastoma cell differentiation. Sci Signal 2015;8:ra40.
173. Kozlov G, Safaee N, Rosenauer A, Gehring K. Structural basis of binding of P-body-associated proteins GW182 and ataxin-2 by the Mlle domain of poly(A)-binding protein. J Biol Chem 2010;285:13599-13606.
174. Huttlin EL, Ting L, Bruckner RJ, et al. The BioPlex Network: A Systematic Exploration of the Human Interactome. Cell 2015;162:425-440.
175. Schlundt A, Sticht J, Piotukh K, et al. Proline-rich sequence recognition: II. Proteomics analysis of Tsg101 ubiquitin-E2-like variant (UEV) interactions. Mol Cell Proteomics 2009;8:2474-2486.
176. Su H, Meng S, Lu Y, et al. Mammalian hyperplastic discs homolog EDD regulates miRNA-mediated gene silencing. Mol Cell 2011;43:97-109.
177. Ewing RM, Chu P, Elisma F, et al. Large-scale mapping of human protein-protein interactions by mass spectrometry. Mol Syst Biol 2007;3:89.
178. Abu-Odeh M, Bar-Mag T, Huang H, et al. Characterizing WW domain interactions of tumor suppressor WWOX reveals its association with multiprotein networks. J Biol Chem 2014;289:8865-8880.
179. Huss JM, Torra IP, Staels B, Giguere V, Kelly DP. Estrogen-related receptor alpha directs peroxisome proliferator-activated receptor alpha signaling in the transcriptional control of energy metabolism in cardiac and skeletal muscle. Mol Cell Biol 2004;24:9079-9091.
180. Cismasiu VB, Adamo K, Gecewicz J, Duque J, Lin Q, Avram D. BCL11B functionally associates with the NuRD complex in T lymphocytes to repress targeted promoter. Oncogene 2005;24:6753-6764.
181. Srinivasan K, Leone DP, Bateson RK, et al. A network of genetic repression and derepression specifies projection fates in the developing neocortex. Proc Natl Acad Sci U S A 2012;109:19071-19078.
182. Scripture-Adams DD, Damle SS, Li L, et al. GATA-3 dose-dependent checkpoints in early T cell commitment. J Immunol 2014;193:3470-3491.
183. Arlotta P, Molyneaux BJ, Jabaudon D, Yoshida Y, Macklis JD. Ctip2 controls the differentiation of medium spiny neurons and the establishment of the cellular architecture of the striatum. J Neurosci 2008;28:622-632.
184. Marban C, Suzanne S, Dequiedt F, et al. Recruitment of chromatin-modifying enzymes by CTIP2 promotes HIV-1 transcriptional silencing. EMBO J 2007;26:412-423.
185. Desplats PA, Lambert JR, Thomas EA. Functional roles for the striatal-enriched transcription factor, Bcl11b, in the control of striatal gene expression and transcriptional dysregulation in Huntington's disease. Neurobiol Dis 2008;31:298-308.
186. Cismasiu VB, Duque J, Paskaleva E, et al. BCL11B enhances TCR/CD28-triggered NF-kappaB activation through up-regulation of Cot kinase gene expression in T-lymphocytes. Biochem J 2009;417:457-466.
187. Vanvalkenburgh J, Albu DI, Bapanpally C, et al. Critical role of Bcl11b in suppressor function of T regulatory cells and prevention of inflammatory bowel disease. J Exp Med 2011;208:2069-2081.

188. Obata M, Kominami R, Mishima Y. BCL11B tumor suppressor inhibits HDM2 expression in a p53-dependent manner. Cell Signal 2012;24:1047-1052.
189. Basei FL, Meirelles GV, Righetto GL, Dos Santos Migueleti DL, Smetana JH, Kobarg J. New interaction partners for Nek4.1 and Nek4.2 isoforms: from the DNA damage response to RNA splicing. Proteome Sci 2015;13:11.
190. Yatim A, Benne C, Sobhian B, et al. NOTCH1 nuclear interactome reveals key regulators of its transcriptional activity and oncogenic function. Mol Cell 2012;48:445-458.
191. Avram D, Fields A, Pretty On Top K, Nevrivy DJ, Ishmael JE, Leid M. Isolation of a novel family of C(2)H(2) zinc finger proteins implicated in transcriptional repression mediated by chicken ovalbumin upstream promoter transcription factor (COUP-TF) orphan nuclear receptors. J Biol Chem 2000;275:10315-10322.
192. Baranek C, Dittrich M, Parthasarathy S, et al. Protooncogene Ski cooperates with the chromatin-remodeling factor Satb2 in specifying callosal neurons. Proc Natl Acad Sci U S A 2012;109:3546-3551.
193. Yang W, Thompson JW, Wang Z, et al. Analysis of oxygen/glucose-deprivation-induced changes in SUMO3 conjugation using SILAC-based quantitative proteomics. J Proteome Res 2012;11:1108-1117.
194. Weber BN, Chi AW, Chavez A, et al. A critical role for TCF-1 in T-lineage specification and differentiation. Nature 2011;476:63-68.
195. Reljic R, Wagner SD, Peakman LJ, Fearon DT. Suppression of signal transducer and activator of transcription 3-dependent B lymphocyte terminal differentiation by BCL-6. J Exp Med 2000;192:1841-1848.
196. Ishii R, Kami D, Toyoda M, et al. Placenta to cartilage: direct conversion of human placenta to chondrocytes with transformation by defined factors. Mol Biol Cell 2012;23:3511-3521.
197. Yachie N, Petsalaki E, Mellor JC, et al. Pooled-matrix protein interaction screens using Barcode Fusion Genetics. Mol Syst Biol 2016;12:863.
198. Luthi-Carter R, Strand AD, Hanson SA, et al. Polyglutamine and transcription: gene expression changes shared by DRPLA and Huntington's disease mouse models reveal context-independent effects. Hum Mol Genet 2002;11:1927-1937.
199. Kojima S, Hatano M, Okada S, et al. Testicular germ cell apoptosis in Bcl6-deficient mice. Development 2001;128:57-65.
200. Yamochi T, Kaneita Y, Akiyama T, Mori S, Moriyama M. Adenovirus-mediated high expression of BCL-6 in CV-1 cells induces apoptotic cell death accompanied by down-regulation of BCL-2 and BCL-X(L). Oncogene 1999;18:487-494.
201. Rolland T, Tasan M, Charloteaux B, et al. A proteome-scale map of the human interactome network. Cell 2014;159:1212-1226.
202. Miles RR, Crockett DK, Lim MS, Elenitoba-Johnson KS. Analysis of BCL6-interacting proteins by tandem mass spectrometry. Mol Cell Proteomics 2005;4:1898-1909.
203. Arima M, Toyama H, Ichii H, et al. A putative silencer element in the IL-5 gene recognized by Bcl6. J Immunol 2002;169:829-836.
204. Luther SA, Cyster JG. Chemokines as regulators of T cell differentiation. Nat Immunol 2001;2:102-107.
205. Shvarts A, Brummelkamp TR, Scheeren F, et al. A senescence rescue screen identifies BCL6 as an inhibitor of anti-proliferative p19(ARF)-p53 signaling. Genes Dev 2002;16:681-686.
206. Hurtz C, Hatzi K, Cerchietti L, et al. BCL6-mediated repression of p53 is critical for leukemia stem cell survival in chronic myeloid leukemia. J Exp Med 2011;208:2163-2174.
207. Suzuki H, Kurita M, Mizumoto K, et al. The ARF tumor suppressor inhibits BCL6-mediated transcriptional repression. Biochem Biophys Res Commun 2005;326:242-248.
208. Pasqualucci L, Dominguez-Sola D, Chiarenza A, et al. Inactivating mutations of acetyltransferase genes in B-cell lymphoma. Nature 2011;471:189-195.

209. Barish GD, Yu RT, Karunasiri M, et al. Bcl-6 and NF-kappaB cistromes mediate opposing regulation of the innate immune response. Genes Dev 2010;24:2760-2765.
210. Kotlyar M, Pastrello C, Pivetta F, et al. In silico prediction of physical protein interactions and characterization of interactome orphans. Nat Methods 2015;12:79-84.
211. Mathew R, Mao AP, Chiang AH, et al. A negative feedback loop mediated by the Bcl6-cullin 3 complex limits Tfh cell differentiation. J Exp Med 2014;211:1137-1151.
212. Ogbe A, Miao T, Symonds AL, et al. Early Growth Response Genes 2 and 3 Regulate the Expression of Bcl6 and Differentiation of T Follicular Helper Cells. J Biol Chem 2015;290:20455-20465.
213. Blandin G, Marchand S, Charton K, et al. A human skeletal muscle interactome centered on proteins involved in muscular dystrophies: LGMD interactome. Skelet Muscle 2013;3:3.
214. Igoillo-Esteve M, Gurzov EN, Eizirik DL, Cnop M. The transcription factor B-cell lymphoma (BCL)-6 modulates pancreatic {beta}-cell inflammatory responses. Endocrinology 2011;152:447-456.
215. Sawant DV, Sehra S, Nguyen ET, et al. Bcl6 controls the Th2 inflammatory activity of regulatory T cells by repressing Gata3 function. J Immunol 2012;189:4759-4769.
216. Phan RT, Saito M, Kitagawa Y, Means AR, Dalla-Favera R. Genotoxic stress regulates expression of the proto-oncogene Bcl6 in germinal center B cells. Nat Immunol 2007;8:1132-1139.
217. Tang TT, Dowbenko D, Jackson A, et al. The forkhead transcription factor AFX activates apoptosis by induction of the BCL-6 transcriptional repressor. J Biol Chem 2002;277:14255-14265.
218. Tiberi L, Bonnefont J, van den Ameele J, et al. A BCL6/BCOR/SIRT1 complex triggers neurogenesis and suppresses medulloblastoma by repressing Sonic Hedgehog signaling. Cancer Cell 2014;26:797-812.
219. Kinugasa Y, Hieda M, Hori M, Higashiyama S. The carboxyl-terminal fragment of pro-HB-EGF reverses Bcl6-mediated gene repression. J Biol Chem 2007;282:14797-14806.
220. Bereshchenko OR, Gu W, Dalla-Favera R. Acetylation inactivates the transcriptional repressor BCL6. Nat Genet 2002;32:606-613.
221. Lemercier C, Brocard MP, Puvion-Dutilleul F, Kao HY, Albagli O, Khochbin S. Class II histone deacetylases are directly recruited by BCL6 transcriptional repressor. J Biol Chem 2002;277:22045-22052.
222. Miyazaki M, Miyazaki K, Chen S, et al. The E-Id protein axis modulates the activities of the PI3K-AKT-mTORC1-Hif1a and c-myc/p19Arf pathways to suppress innate variant TFH cell development, thymocyte expansion, and lymphomagenesis. Genes Dev 2015;29:409-425.
223. Kusam S, Toney LM, Sato H, Dent AL. Inhibition of Th2 differentiation and GATA-3 expression by BCL-6. J Immunol 2003;170:2435-2441.
224. Mondal A, Sawant D, Dent AL. Transcriptional repressor BCL6 controls Th17 responses by controlling gene expression in both T cells and macrophages. J Immunol 2010;184:4123-4132.
225. Ying H, Kang Y, Zhang H, et al. MiR-127 modulates macrophage polarization and promotes lung inflammation and injury by activating the JNK pathway. J Immunol 2015;194:1239-1251.
226. Yoon J, Feng X, Kim YS, et al. Interferon regulatory factor 8 (IRF8) interacts with the B cell lymphoma 6 (BCL6) corepressor BCOR. J Biol Chem 2014;289:34250-34257.
227. Vasanwala FH, Kusam S, Toney LM, Dent AL. Repression of AP-1 function: a mechanism for the regulation of Blimp-1 expression and B lymphocyte differentiation by the B cell lymphoma-6 protooncogene. J Immunol 2002;169:1922-1929.
228. Terragni J, Nayak G, Banerjee S, et al. The E-box binding factors Max/Mnt, MITF, and USF1 act coordinately with FoxO to regulate expression of proapoptotic and cell cycle control genes by phosphatidylinositol 3-kinase/Akt/glycogen synthase kinase 3 signaling. J Biol Chem 2011;286:36215-36227.
229. Fujita N, Jaye DL, Geigerman C, et al. MTA3 and the Mi-2/NuRD complex regulate cell fate during B lymphocyte differentiation. Cell 2004;119:75-86.
230. Fukasawa R, Tsutsui T, Hirose Y, Tanaka A, Ohkuma Y. Mediator CDK subunits are platforms for interactions with various chromatin regulatory complexes. J Biochem 2012;152:241-249.

231. Bergstrom DA, Penn BH, Strand A, Perry RL, Rudnicki MA, Tapscott SJ. Promoter-specific regulation of MyoD binding and signal transduction cooperate to pattern gene expression. Mol Cell 2002;9:587-600.
232. Huynh KD, Fischle W, Verdin E, Bardwell VJ. BCoR, a novel corepressor involved in BCL-6 repression. Genes Dev 2000;14:1810-1823.
233. Li Z, Wang X, Yu RY, et al. BCL-6 negatively regulates expression of the NF-kappaB1 p105/p50 subunit. J Immunol 2005;174:205-214.
234. Zhang P, Zhao Y, Sun XH. Notch-regulated periphery B cell differentiation involves suppression of E protein function. J Immunol 2013;191:726-736.
235. Dhordain P, Albagli O, Honore N, et al. Colocalization and heteromerization between the two human oncogene POZ/zinc finger proteins, LAZ3 (BCL6) and PLZF. Oncogene 2000;19:6240-6250.
236. Lee CH, Chawla A, Urbiztondo N, et al. Transcriptional repression of atherogenic inflammation: modulation by PPARdelta. Science 2003;302:453-457.
237. Ci W, Polo JM, Cerchietti L, et al. The BCL6 transcriptional program features repression of multiple oncogenes in primary B cells and is deregulated in DLBCL. Blood 2009;113:5536-5548.
238. Juszczynski P, Chen L, O'Donnell E, et al. BCL6 modulates tonic BCR signaling in diffuse large B-cell lymphomas by repressing the SYK phosphatase, PTPROt. Blood 2009;114:5315-5321.
239. Jin S, Zhuo Y, Guo W, Field J. p21-activated Kinase 1 (Pak1)-dependent phosphorylation of Raf-1 regulates its mitochondrial localization, phosphorylation of BAD, and Bcl-2 association. J Biol Chem 2005;280:24698-24705.
240. Wang D, Long J, Dai F, Liang M, Feng XH, Lin X. BCL6 represses Smad signaling in transforming growth factor-beta resistance. Cancer Res 2008;68:783-789.
241. Lee DK, Suh D, Edenberg HJ, Hur MW. POZ domain transcription factor, FBI-1, represses transcription of ADH5/FDH by interacting with the zinc finger and interfering with DNA binding activity of Sp1. J Biol Chem 2002;277:26761-26768.
242. Wei F, Zaprazna K, Wang J, Atchison ML. PU.1 can recruit BCL6 to DNA to repress gene expression in germinal center B cells. Mol Cell Biol 2009;29:4612-4622.
243. Walker SR, Nelson EA, Yeh JE, Pinello L, Yuan GC, Frank DA. STAT5 outcompetes STAT3 to regulate the expression of the oncogenic transcriptional modulator BCL6. Mol Cell Biol 2013;33:2879-2890.
244. Tozawa H, Kanki Y, Suehiro J, et al. Genome-wide approaches reveal functional interleukin-4-inducible STAT6 binding to the vascular cell adhesion molecule 1 promoter. Mol Cell Biol 2011;31:2196-2209.
245. Mengus G, Fadloun A, Kobi D, et al. TAF4 inactivation in embryonic fibroblasts activates TGF beta signalling and autocrine growth. EMBO J 2005;24:2753-2767.
246. Davies JM, Hawe N, Kabarowski J, et al. Novel BTB/POZ domain zinc-finger protein, LRF, is a potential target of the LAZ-3/BCL-6 oncogene. Oncogene 1999;18:365-375.
247. Olah J, Vincze O, Virok D, et al. Interactions of pathological hallmark proteins: tubulin polymerization promoting protein/p25, beta-amyloid, and alpha-synuclein. J Biol Chem 2011;286:34088-34100.
248. Abdelmohsen K, Srikantan S, Yang X, et al. Ubiquitin-mediated proteolysis of HuR by heat shock. EMBO J 2009;28:1271-1282.
249. Stehling O, Vashisht AA, Mascarenhas J, et al. MMS19 assembles iron-sulfur proteins required for DNA metabolism and genomic integrity. Science 2012;337:195-199.
250. Varjosalo M, Keskitalo S, Van Drogen A, et al. The protein interaction landscape of the human CMGC kinase group. Cell Rep 2013;3:1306-1320.
251. Reddy K, Zamiri B, Stanley SY, Macgregor RB, Jr., Pearson CE. The disease-associated r(GGGGCC)n repeat from the C9orf72 gene forms tract length-dependent uni- and multimolecular RNA G-quadruplex structures. J Biol Chem 2013;288:9860-9866.

252. Webster CP, Smith EF, Bauer CS, et al. The C9orf72 protein interacts with Rab1a and the ULK1 complex to regulate initiation of autophagy. EMBO J 2016;35:1656-1676.
253. Fan Z, Beresford PJ, Zhang D, et al. Cleaving the oxidative repair protein Ape1 enhances cell death mediated by granzyme A. Nat Immunol 2003;4:145-153.
254. Fung H, Liu P, Demple B. ATF4-dependent oxidative induction of the DNA repair enzyme Ape1 counteracts arsenite cytotoxicity and suppresses arsenite-mediated mutagenesis. Mol Cell Biol 2007;27:8834-8847.
255. Huang E, Qu D, Zhang Y, et al. The role of Cdk5-mediated apurinic/apyrimidinic endonuclease 1 phosphorylation in neuronal death. Nat Cell Biol 2010;12:563-571.
256. Cao Q, Wang X, Zhao M, et al. The central role of EED in the orchestration of polycomb group complexes. Nat Commun 2014;5:3127.
257. Karmakar S, Mahajan MC, Schulz V, Boyapaty G, Weissman SM. A multiprotein complex necessary for both transcription and DNA replication at the beta-globin locus. EMBO J 2010;29:3260-3271.
258. Liccardi G, Hartley JA, Hochhauser D. Importance of EGFR/ERCC1 interaction following radiation-induced DNA damage. Clin Cancer Res 2014;20:3496-3506.
259. Pagel P, Kovac S, Oesterheld M, et al. The MIPS mammalian protein-protein interaction database. Bioinformatics 2005;21:832-834.
260. Jung HJ, Kim HL, Kim YJ, Weon JI, Seo YR. A novel chemopreventive mechanism of selenomethionine: enhancement of APE1 enzyme activity via a Gadd45a, PCNA and APE1 protein complex that regulates p53-mediated base excision repair. Oncol Rep 2013;30:1581-1586.
261. Sengupta S, Chattopadhyay R, Mantha AK, Mitra S, Bhakat KK. Regulation of mouse-renin gene by apurinic/apyrimidinic-endonuclease 1 (APE1/Ref-1) via recruitment of histone deacetylase 1 corepressor complex. J Hypertens 2012;30:917-925.
262. Bhakat KK, Izumi T, Yang SH, Hazra TK, Mitra S. Role of acetylated human AP-endonuclease (APE1/Ref-1) in regulation of the parathyroid hormone gene. EMBO J 2003;22:6299-6309.
263. Carrero P, Okamoto K, Coumailleau P, O'Brien S, Tanaka H, Poellinger L. Redox-regulated recruitment of the transcriptional coactivators CREB-binding protein and SRC-1 to hypoxia-inducible factor 1alpha. Mol Cell Biol 2000;20:402-415.
264. Sgarra R, Furlan C, Zammitti S, et al. Interaction proteomics of the HMGA chromatin architectural factors. Proteomics 2008;8:4721-4732.
265. Prasad R, Liu Y, Deterding LJ, et al. HMGB1 is a cofactor in mammalian base excision repair. Mol Cell 2007;27:829-841.
266. Kuninger DT, Izumi T, Papaconstantinou J, Mitra S. Human AP-endonuclease 1 and hnRNP-L interact with a nCaRE-like repressor element in the AP-endonuclease 1 promoter. Nucleic Acids Res 2002;30:823-829.
267. Kimura M, Okumura N, Kose S, Takao T, Imamoto N. Identification of cargo proteins specific for importin-beta with importin-alpha applying a stable isotope labeling by amino acids in cell culture (SILAC)-based in vitro transport system. J Biol Chem 2013;288:24540-24549.
268. Vascotto C, Fantini D, Romanello M, et al. APE1/Ref-1 interacts with NPM1 within nucleoli and plays a role in the rRNA quality control process. Mol Cell Biol 2009;29:1834-1854.
269. Busso CS, Iwakuma T, Izumi T. Ubiquitination of mammalian AP endonuclease (APE1) regulated by the p53-MDM2 signaling pathway. Oncogene 2009;28:1616-1625.
270. Balasenthil S, Gururaj AE, Talukder AH, et al. Identification of Pax5 as a target of MTA1 in B-cell lymphomas. Cancer Res 2007;67:7132-7138.
271. Mao DY, Watson JD, Yan PS, et al. Analysis of Myc bound loci identified by CpG island arrays shows that Max is essential for Myc-dependent repression. Curr Biol 2003;13:882-886.
272. Perini G, Diolaiti D, Porro A, Della Valle G. In vivo transcriptional regulation of N-Myc target genes is controlled by E-box methylation. Proc Natl Acad Sci U S A 2005;102:12117-12122.

273. Weiler M, Blaes J, Pusch S, et al. mTOR target NDRG1 confers MGMT-dependent resistance to alkylating chemotherapy. Proc Natl Acad Sci U S A 2014;111:409-414.
274. Hegde PM, Dutta A, Sengupta S, et al. The C-terminal Domain (CTD) of Human DNA Glycosylase NEIL1 Is Required for Forming BERosome Repair Complex with DNA Replication Proteins at the Replicating Genome: DOMINANT NEGATIVE FUNCTION OF THE CTD. J Biol Chem 2015;290:20919-20933.
275. Jung HJ, Kim EH, Mun JY, et al. Base excision DNA repair defect in Gadd45a-deficient cells. Oncogene 2007;26:7517-7525.
276. Piazzi M, Blalock WL, Bavelloni A, et al. Phosphoinositide-specific phospholipase C beta 1b (PI-PLCbeta1b) interactome: affinity purification-mass spectrometry analysis of PI-PLCbeta1b with nuclear protein. Mol Cell Proteomics 2013;12:2220-2235.
277. Hays T, Rusyn I, Burns AM, et al. Role of peroxisome proliferator-activated receptor-alpha (PPARalpha) in bezafibrate-induced hepatocarcinogenesis and cholestasis. Carcinogenesis 2005;26:219-227.
278. Hu XV, Rodrigues TM, Tao H, et al. Identification of RING finger protein 4 (RNF4) as a modulator of DNA demethylation through a functional genomics screen. Proc Natl Acad Sci U S A 2010;107:15087-15092.
279. Marechal A, Li JM, Ji XY, et al. PRP19 transforms into a sensor of RPA-ssDNA after DNA damage and drives ATR activation via a ubiquitin-mediated circuitry. Mol Cell 2014;53:235-246.
280. Gray MJ, Zhang J, Ellis LM, et al. HIF-1alpha, STAT3, CBP/p300 and Ref-1/APE are components of a transcriptional complex that regulates Src-dependent hypoxia-induced expression of VEGF in pancreatic and prostate carcinomas. Oncogene 2005;24:3110-3120.
281. Lockyer PJ, Wennstrom S, Kupzig S, Venkateswarlu K, Downward J, Cullen PJ. Identification of the ras GTPase-activating protein GAP1(m) as a phosphatidylinositol-3,4,5-trisphosphate-binding protein in vivo. Curr Biol 1999;9:265-268.
282. Lee OH, Kim H, He Q, et al. Genome-wide YFP fluorescence complementation screen identifies new regulators for telomere signaling in human cells. Mol Cell Proteomics 2011;10:M110 001628.
283. Seemann S, Hainaut P. Roles of thioredoxin reductase 1 and APE/Ref-1 in the control of basal p53 stability and activity. Oncogene 2005;24:3853-3863.
284. Merluzzi S, D'Orlando O, Leonardi A, Vitale G, Pucillo C. TRAF2 and p38 are involved in B cells CD40-mediated APE/Ref-1 nuclear translocation: a novel pathway in B cell activation. Mol Immunol 2008;45:76-86.
285. Powis G, Montfort WR. Properties and biological activities of thioredoxins. Annu Rev Pharmacol Toxicol 2001;41:261-295.
286. Yan MD, Xu WJ, Lu LR, Sun LY, Liu XY, Zheng ZC. Ubiquitin Conjugating Enzyme Ubc9 is Involved in Protein Degradation of Redox Factor-1 (Ref-1). Sheng Wu Hua Xue Yu Sheng Wu Wu Li Xue Bao (Shanghai) 2000;32:63-68.
287. de Melo JT, de Souza Timoteo AR, Lajus TB, et al. XPC deficiency is related to APE1 and OGG1 expression and function. Mutat Res 2016;784-785:25-33.
288. Chung U, Igarashi T, Nishishita T, et al. The interaction between Ku antigen and REF1 protein mediates negative gene regulation by extracellular calcium. J Biol Chem 1996;271:8593-8598.
289. Sengupta S, Mantha AK, Mitra S, Bhakat KK. Human AP endonuclease (APE1/Ref-1) and its acetylation regulate YB-1-p300 recruitment and RNA polymerase II loading in the drug-induced activation of multidrug resistance gene MDR1. Oncogene 2011;30:482-493.
290. Kong M, Barnes EA, Ollendorff V, Donoghue DJ. Cyclin F regulates the nuclear localization of cyclin B1 through a cyclin-cyclin interaction. EMBO J 2000;19:1378-1388.
291. Kapoor A, Yao W, Ying H, et al. Yap1 activation enables bypass of oncogenic Kras addiction in pancreatic cancer. Cell 2014;158:185-197.

292. Wang X, Quail E, Hung NJ, Tan Y, Ye H, Costa RH. Increased levels of forkhead box M1B transcription factor in transgenic mouse hepatocytes prevent age-related proliferation defects in regenerating liver. Proc Natl Acad Sci U S A 2001;98:11468-11473.
293. Tejera MM, Kim EH, Sullivan JA, Plisch EH, Suresh M. FoxO1 controls effector-to-memory transition and maintenance of functional CD8 T cell memory. J Immunol 2013;191:187-199.
294. Caetano MS, Vieira-de-Abreu A, Teixeira LK, Werneck MB, Barcinski MA, Viola JP. NFATC2 transcription factor regulates cell cycle progression during lymphocyte activation: evidence of its involvement in the control of cyclin gene expression. FASEB J 2002;16:1940-1942.
295. Sharma SS, Ma L, Bagui TK, Forinash KD, Pledger WJ. A p27Kip1 mutant that does not inhibit CDK activity promotes centrosome amplification and micronucleation. Oncogene 2012;31:3989-3998.
296. Yoshida Y, Murakami A, Tanaka K. Skp1 stabilizes the conformation of F-box proteins. Biochem Biophys Res Commun 2011;410:24-28.
297. Angeletti B, Waldron KJ, Freeman KB, et al. BACE1 cytoplasmic domain interacts with the copper chaperone for superoxide dismutase-1 and binds copper. J Biol Chem 2005;280:17930-17937.
298. Jiang Y, Reynolds C, Xiao C, et al. Dietary copper supplementation reverses hypertrophic cardiomyopathy induced by chronic pressure overload in mice. J Exp Med 2007;204:657-666.
299. Yang Q, Esplin B, Borghesi L. E47 regulates hematopoietic stem cell proliferation and energetics but not myeloid lineage restriction. Blood 2011;117:3529-3538.
300. Brady GF, Galban S, Liu X, et al. Regulation of the copper chaperone CCS by XIAP-mediated ubiquitination. Mol Cell Biol 2010;30:1923-1936.
301. Arroyo R, Sune G, Zanzoni A, et al. Systematic identification of molecular links between core and candidate genes in breast cancer. J Mol Biol 2015;427:1436-1450.
302. Kaltenbach LS, Romero E, Becklin RR, et al. Huntingtin interacting proteins are genetic modifiers of neurodegeneration. PLoS Genet 2007;3:e82.
303. Joshi MB, Ivanov D, Philippova M, Erne P, Resink TJ. Integrin-linked kinase is an essential mediator for T-cadherin-dependent signaling via Akt and GSK3beta in endothelial cells. FASEB J 2007;21:3083-3095.
304. Philippova M, Joshi MB, Pfaff D, et al. T-cadherin attenuates insulin-dependent signalling, eNOS activation, and angiogenesis in vascular endothelial cells. Cardiovasc Res 2012;93:498-507.
305. Benleulmi-Chaachoua A, Chen L, Sokolina K, et al. Protein interactome mining defines melatonin MT1 receptors as integral component of presynaptic protein complexes of neurons. J Pineal Res 2016;60:95-108.
306. Ule J, Jensen KB, Ruggiu M, Mele A, Ule A, Darnell RB. CLIP identifies Nova-regulated RNA networks in the brain. Science 2003;302:1212-1215.
307. Gorski JA, Gomez LL, Scott JD, Dell'Acqua ML. Association of an A-kinase-anchoring protein signaling scaffold with cadherin adhesion molecules in neurons and epithelial cells. Mol Biol Cell 2005;16:3574-3590.
308. Vinayagam A, Stelzl U, Foulle R, et al. A directed protein interaction network for investigating intracellular signal transduction. Sci Signal 2011;4:rs8.
309. Huttunen HJ, Kuja-Panula J, Rauvala H. Receptor for advanced glycation end products (RAGE) signaling induces CREB-dependent chromogranin expression during neuronal differentiation. J Biol Chem 2002;277:38635-38646.
310. Mikoshiba K. IP3 receptor/Ca2+ channel: from discovery to new signaling concepts. J Neurochem 2007;102:1426-1446.
311. Reyniers L, Del Giudice MG, Civiero L, et al. Differential protein-protein interactions of LRRK1 and LRRK2 indicate roles in distinct cellular signaling pathways. J Neurochem 2014;131:239-250.
312. Masumi A, Yamakawa Y, Fukazawa H, Ozato K, Komuro K. Interferon regulatory factor-2 regulates cell growth through its acetylation. J Biol Chem 2003;278:25401-25407.

313. Khoo C, Yang J, Weinrott SA, et al. Research resource: the pdx1 cistrome of pancreatic islets. Mol Endocrinol 2012;26:521-533.
314. Klajn A, Ferrai C, Stucchi L, et al. The rest repression of the neurosecretory phenotype is negatively modulated by BHC80, a protein of the BRAF/HDAC complex. J Neurosci 2009;29:6296-6307.
315. Miyamoto-Sato E, Fujimori S, Ishizaka M, et al. A comprehensive resource of interacting protein regions for refining human transcription factor networks. PLoS One 2010;5:e9289.
316. Stieren ES, El Ayadi A, Xiao Y, et al. Ubiquilin-1 is a molecular chaperone for the amyloid precursor protein. J Biol Chem 2011;286:35689-35698.
317. Chassefeyre R, Martinez-Hernandez J, Bertaso F, et al. Regulation of postsynaptic function by the dementia-related ESCRT-III subunit CHMP2B. J Neurosci 2015;35:3155-3173.
318. Tsang HT, Connell JW, Brown SE, Thompson A, Reid E, Sanderson CM. A systematic analysis of human CHMP protein interactions: additional MIT domain-containing proteins bind to multiple components of the human ESCRT III complex. Genomics 2006;88:333-346.
319. !!! INVALID CITATION !!! 229.
320. Rigden DJ, Liu H, Hayes SD, Urbe S, Clague MJ. Ab initio protein modelling reveals novel human MIT domains. FEBS Lett 2009;583:872-878.
321. McDonald NQ, Panayotatos N, Hendrickson WA. Crystal structure of dimeric human ciliary neurotrophic factor determined by MAD phasing. EMBO J 1995;14:2689-2699.
322. Derouet D, Rousseau F, Alfonsi F, et al. Neuropoietin, a new IL-6-related cytokine signaling through the ciliary neurotrophic factor receptor. Proc Natl Acad Sci U S A 2004;101:4827-4832.
323. de Melo J, Miki K, Rattner A, et al. Injury-independent induction of reactive gliosis in retina by loss of function of the LIM homeodomain transcription factor Lhx2. Proc Natl Acad Sci U S A 2012;109:4657-4662.
324. Hefti F. Pharmacology of neurotrophic factors. Annu Rev Pharmacol Toxicol 1997;37:239-267.
325. Larsen JV, Hansen M, Moller B, et al. Sortilin facilitates signaling of ciliary neurotrophic factor and related helical type 1 cytokines targeting the gp130/leukemia inhibitory factor receptor beta heterodimer. Mol Cell Biol 2010;30:4175-4187.
326. Osterfield M, Egelund R, Young LM, Flanagan JG. Interaction of amyloid precursor protein with contactins and NgCAM in the retinotectal system. Development 2008;135:1189-1199.
327. Chen Y, Yang LN, Cheng L, et al. Bcl2-associated athanogene 3 interactome analysis reveals a new role in modulating proteasome activity. Mol Cell Proteomics 2013;12:2804-2819.
328. Singh D, Lampe PD. Identification of connexin-43 interacting proteins. Cell Commun Adhes 2003;10:215-220.
329. Gao S, Wu H, Wang F, Wang Z. Altered differentiation and proliferation of prostate epithelium in mice lacking the androgen receptor cofactor p44/WDR77. Endocrinology 2010;151:3941-3953.
330. Kahle JJ, Gulbahce N, Shaw CA, et al. Comparison of an expanded ataxia interactome with patient medical records reveals a relationship between macular degeneration and ataxia. Hum Mol Genet 2011;20:510-527.
331. Kasper LH, Fukuyama T, Lerach S, et al. Genetic interaction between mutations in c-Myb and the KIX domains of CBP and p300 affects multiple blood cell lineages and influences both gene activation and repression. PLoS One 2013;8:e82684.
332. Mertz J, Tan H, Pagala V, et al. Sequential Elution Interactome Analysis of the Mind Bomb 1 Ubiquitin Ligase Reveals a Novel Role in Dendritic Spine Outgrowth. Mol Cell Proteomics 2015;14:1898-1910.
333. Chia R, Haddock S, Beilina A, et al. Phosphorylation of LRRK2 by casein kinase 1alpha regulates trans-Golgi clustering via differential interaction with ARHGEF7. Nat Commun 2014;5:5827.
334. Behrends C, Sowa ME, Gygi SP, Harper JW. Network organization of the human autophagy system. Nature 2010;466:68-76.

335. Negro A, Brunati AM, Donella-Deana A, Massimino ML, Pinna LA. Multiple phosphorylation of alpha-synuclein by protein tyrosine kinase Syk prevents eosin-induced aggregation. FASEB J 2002;16:210-212.
336. Levy E, Sastre M, Kumar A, et al. Codeposition of cystatin C with amyloid-beta protein in the brain of Alzheimer disease patients. J Neuropathol Exp Neurol 2001;60:94-104.
337. Tsiolaki PL, Hamodrakas SJ, Iconomidou VA. The pentapeptide LQVVR plays a pivotal role in human cystatin C fibrillization. FEBS Lett 2015;589:159-164.
338. Papin J, Subramaniam S. Bioinformatics and cellular signaling. Curr Opin Biotechnol 2004;15:78-81.
339. Barlic J, McDermott DH, Merrell MN, Gonzales J, Via LE, Murphy PM. Interleukin (IL)-15 and IL-2 reciprocally regulate expression of the chemokine receptor CX3CR1 through selective NFAT1- and NFAT2-dependent mechanisms. J Biol Chem 2004;279:48520-48534.
340. Cairns W, Smith CA, McLaren AW, Wolf CR. Characterization of the human cytochrome P4502D6 promoter. A potential role for antagonistic interactions between members of the nuclear receptor family. J Biol Chem 1996;271:25269-25276.
341. Popiolek M, Ross JF, Charych E, et al. D-amino acid oxidase activity is inhibited by an interaction with bassoon protein at the presynaptic active zone. J Biol Chem 2011;286:28867-28875.
342. Gupta GD, Coyaud E, Goncalves J, et al. A Dynamic Protein Interaction Landscape of the Human Centrosome-Cilium Interface. Cell 2015;163:1484-1499.
343. Boldt K, van Reeuwijk J, Lu Q, et al. An organelle-specific protein landscape identifies novel diseases and molecular mechanisms. Nat Commun 2016;7:11491.
344. Smith DS, Niethammer M, Ayala R, et al. Regulation of cytoplasmic dynein behaviour and microtubule organization by mammalian Lis1. Nat Cell Biol 2000;2:767-775.
345. Chang B, Khanna H, Hawes N, et al. In-frame deletion in a novel centrosomal/ciliary protein CEP290/NPHP6 perturbs its interaction with RPGR and results in early-onset retinal degeneration in the rd16 mouse. Hum Mol Genet 2006;15:1847-1857.
346. Weisbrich A, Honnappa S, Jaussi R, et al. Structure-function relationship of CAP-Gly domains. Nat Struct Mol Biol 2007;14:959-967.
347. Camargo LM, Collura V, Rain JC, et al. Disrupted in schizophrenia 1 interactome: evidence for the close connectivity of risk genes and a potential synaptic basis for schizophrenia. Mol Psychiatr 2007;12:74-86.
348. Liu JJ, Ding J, Kowal AS, et al. BPAG1n4 is essential for retrograde axonal transport in sensory neurons. J Cell Biol 2003;163:223-229.
349. Faulkner NE, Dujardin DL, Tai CY, et al. A role for the lissencephaly gene LIS1 in mitosis and cytoplasmic dynein function. Nat Cell Biol 2000;2:784-791.
350. Cao Q, Wang XJ, Zhao M, et al. The central role of EED in orchestration of polycomb group complexes. Cancer Research 2014;74.
351. Sahni M, Zhou XM, Bakiri L, Schlessinger J, Baron R, Levy JB. Identification of a novel 135-kDa Grb2-binding protein in osteoclasts. J Biol Chem 1996;271:33141-33147.
352. Engelender S, Sharp AH, Colomer V, et al. Huntingtin-associated protein 1 (HAP1) interacts with the p150Glued subunit of dynactin. Hum Mol Genet 1997;6:2205-2212.
353. Grose JH, Langston K, Wang X, et al. Characterization of the Cardiac Overexpression of HSPB2 Reveals Mitochondrial and Myogenic Roles Supported by a Cardiac HspB2 Interactome. PLoS One 2015;10:e0133994.
354. Brouillet E, Jacquard C, Bizat N, Blum D. 3-Nitropropionic acid: a mitochondrial toxin to uncover physiopathological mechanisms underlying striatal degeneration in Huntington's disease. J Neurochem 2005;95:1521-1540.

355. Gorbea C, Pratt G, Ustrell V, et al. A protein interaction network for Ecm29 links the 26 S proteasome to molecular motors and endosomal components. J Biol Chem 2010;285:31616-31633.
356. Kodani A, Salome Sirerol-Piquer M, Seol A, Garcia-Verdugo JM, Reiter JF. Kif3a interacts with Dynactin subunit p150 Glued to organize centriole subdistal appendages. EMBO J 2013;32:597-607.
357. Colin E, Zala D, Liot G, et al. Huntingtin phosphorylation acts as a molecular switch for anterograde/retrograde transport in neurons. EMBO J 2008;27:2124-2134.
358. Yamada M, Toba S, Takitoh T, et al. mNUDC is required for plus-end-directed transport of cytoplasmic dynein and dynactins by kinesin-1. EMBO J 2010;29:517-531.
359. Magnani E, Fan J, Gasparini L, et al. Interaction of tau protein with the dynactin complex. EMBO J 2007;26:4546-4554.
360. Nguyen MD, Shu T, Sanada K, et al. A NUDEL-dependent mechanism of neurofilament assembly regulates the integrity of CNS neurons. Nat Cell Biol 2004;6:595-608.
361. Bharti P, Schliebs W, Schievelbusch T, et al. PEX14 is required for microtubule-based peroxisome motility in human cells. J Cell Sci 2011;124:1759-1768.
362. Esteves SL, Domingues SC, da Cruz e Silva OA, Fardilha M, da Cruz e Silva EF. Protein phosphatase 1alpha interacting proteins in the human brain. OMICS 2012;16:3-17.
363. Xie Y, Avello M, Schirle M, et al. Deubiquitinase FAM/USP9X interacts with the E3 ubiquitin ligase SMURF1 protein and protects it from ligase activity-dependent self-degradation. J Biol Chem 2013;288:2976-2985.
364. Lleres D, Denegri M, Biggiogera M, Ajuh P, Lamond AI. Direct interaction between hnRNP-M and CDC5L/PLRG1 proteins affects alternative splice site choice. EMBO Rep 2010;11:445-451.
365. Niu Y, Zhang C, Sun Z, et al. PtdIns(4)P regulates retromer-motor interaction to facilitate dynein-cargo dissociation at the trans-Golgi network. Nat Cell Biol 2013;15:417-429.
366. Holleran EA, Tokito MK, Karki S, Holzbaur EL. Centractin (ARP1) associates with spectrin revealing a potential mechanism to link dynactin to intracellular organelles. J Cell Biol 1996;135:1815-1829.
367. Bandyopadhyay S, Chiang CY, Srivastava J, et al. A human MAP kinase interactome. Nat Methods 2010;7:801-805.
368. Lo KW, Kan HM, Chan LN, et al. The 8-kDa dynein light chain binds to p53-binding protein 1 and mediates DNA damage-induced p53 nuclear accumulation. J Biol Chem 2005;280:8172-8179.
369. Payne C, Rawe V, Ramalho-Santos J, Simerly C, Schatten G. Preferentially localized dynein and perinuclear dynactin associate with nuclear pore complex proteins to mediate genomic union during mammalian fertilization. J Cell Sci 2003;116:4727-4738.
370. Colland F, Jacq X, Trouplin V, et al. Functional proteomics mapping of a human signaling pathway. Genome Res 2004;14:1324-1332.
371. Yasuda S, Oceguera-Yanez F, Kato T, et al. Cdc42 and mDia3 regulate microtubule attachment to kinetochores. Nature 2004;428:767-771.
372. Fevr T, Robine S, Louvard D, Huelsken J. Wnt/beta-catenin is essential for intestinal homeostasis and maintenance of intestinal stem cells. Mol Cell Biol 2007;27:7551-7559.
373. He Y, Mueller S, Chipman PR, et al. Complexes of poliovirus serotypes with their common cellular receptor, CD155. J Virol 2003;77:4827-4835.
374. Castello A, Fischer B, Eichelbaum K, et al. Insights into RNA biology from an atlas of mammalian mRNA-binding proteins. Cell 2012;149:1393-1406.
375. Birdsey GM, Dryden NH, Shah AV, et al. The transcription factor Erg regulates expression of histone deacetylase 6 and multiple pathways involved in endothelial cell migration and angiogenesis. Blood 2012;119:894-903.
376. Hattangadi SM, Burke KA, Lodish HF. Homeodomain-interacting protein kinase 2 plays an important role in normal terminal erythroid differentiation. Blood 2010;115:4853-4861.

377. Wen Y, Eng CH, Schmoranzer J, et al. EB1 and APC bind to mDia to stabilize microtubules downstream of Rho and promote cell migration. Nat Cell Biol 2004;6:820-830.
378. Tominaga T, Sahai E, Chardin P, McCormick F, Courtneidge SA, Alberts AS. Diaphanous-related formins bridge Rho GTPase and Src tyrosine kinase signaling. Mol Cell 2000;5:13-25.
379. Millar JK, Christie S, Porteous DJ. Yeast two-hybrid screens implicate DISC1 in brain development and function. Biochem Biophys Res Commun 2003;311:1019-1025.
380. Morris JA, Kandpal G, Ma L, Austin CP. DISC1 (Disrupted-In-Schizophrenia 1) is a centrosome-associated protein that interacts with MAP1A, MIPT3, ATF4/5 and NUDEL: regulation and loss of interaction with mutation. Hum Mol Genet 2003;12:1591-1608.
381. Young-Pearse TL, Suth S, Luth ES, Sawa A, Selkoe DJ. Biochemical and functional interaction of disrupted-in-schizophrenia 1 and amyloid precursor protein regulates neuronal migration during mammalian cortical development. J Neurosci 2010;30:10431-10440.
382. Lee SA, Kim SM, Suh BK, et al. Disrupted-in-schizophrenia 1 (DISC1) regulates dysbindin function by enhancing its stability. J Biol Chem 2015;290:7087-7096.
383. Miyoshi K, Honda A, Baba K, et al. Disrupted-In-Schizophrenia 1, a candidate gene for schizophrenia, participates in neurite outgrowth. Mol Psychiatry 2003;8:685-694.
384. Shinoda T, Taya S, Tsuboi D, et al. DISC1 regulates neurotrophin-induced axon elongation via interaction with Grb2. J Neurosci 2007;27:4-14.
385. Coba MP, Komiyama NH, Nithianantharajah J, et al. TNiK is required for postsynaptic and nuclear signaling pathways and cognitive function. J Neurosci 2012;32:13987-13999.
386. Taya S, Shinoda T, Tsuboi D, et al. DISC1 regulates the transport of the NUDEL/LIS1/14-3-3epsilon complex through kinesin-1. J Neurosci 2007;27:15-26.
387. Kamiya A, Tomoda T, Chang J, et al. DISC1-NDEL1/NUDEL protein interaction, an essential component for neurite outgrowth, is modulated by genetic variations of DISC1. Hum Mol Genet 2006;15:3313-3323.
388. Guettler S, LaRose J, Petsalaki E, et al. Structural basis and sequence rules for substrate recognition by Tankyrase explain the basis for cherubism disease. Cell 2011;147:1340-1354.
389. Ozeki Y, Tomoda T, Kleiderlein J, et al. Disrupted-in-Schizophrenia-1 (DISC-1): mutant truncation prevents binding to NudE-like (NUDEL) and inhibits neurite outgrowth. Proc Natl Acad Sci U S A 2003;100:289-294.
390. Nagano F, Orita S, Sasaki T, et al. Interaction of Doc2 with tctex-1, a light chain of cytoplasmic dynein. Implication in dynein-dependent vesicle transport. J Biol Chem 1998;273:30065-30068.
391. Yu H, Rathore SS, Davis EM, Ouyang Y, Shen J. Doc2b promotes GLUT4 exocytosis by activating the SNARE-mediated fusion reaction in a calcium- and membrane bending-dependent manner. Mol Biol Cell 2013;24:1176-1184.
392. Verhage M, de Vries KJ, Roshol H, Burbach JP, Gispen WH, Sudhof TC. DOC2 proteins in rat brain: complementary distribution and proposed function as vesicular adapter proteins in early stages of secretion. Neuron 1997;18:453-461.
393. Hashimoto Y, Shirane M, Matsuzaki F, Saita S, Ohnishi T, Nakayama KI. Protrudin regulates endoplasmic reticulum morphology and function associated with the pathogenesis of hereditary spastic paraplegia. J Biol Chem 2014;289:12946-12961.
394. Chung KY, Day PW, Velez-Ruiz G, Sunahara RK, Kobilka BK. Identification of GPCR-interacting cytosolic proteins using HDL particles and mass spectrometry-based proteomic approach. PLoS One 2013;8:e54942.
395. Xiao K, McClatchy DB, Shukla AK, et al. Functional specialization of beta-arrestin interactions revealed by proteomic analysis. Proc Natl Acad Sci U S A 2007;104:12011-12016.

396. Berggard T, Arrigoni G, Olsson O, Fex M, Linse S, James P. 140 mouse brain proteins identified by Ca2+-calmodulin affinity chromatography and tandem mass spectrometry. J Proteome Res 2006;5:669-687.
397. Jirawatnotai S, Hu Y, Michowski W, et al. A function for cyclin D1 in DNA repair uncovered by protein interactome analyses in human cancers. Nature 2011;474:230-234.
398. Wang X, Venable J, LaPointe P, et al. Hsp90 cochaperone Aha1 downregulation rescues misfolding of CFTR in cystic fibrosis. Cell 2006;127:803-815.
399. Hutchins JR, Toyoda Y, Hegemann B, et al. Systematic analysis of human protein complexes identifies chromosome segregation proteins. Science 2010;328:593-599.
400. Papp SJ, Huber AL, Jordan SD, et al. DNA damage shifts circadian clock time via Hausp-dependent Cry1 stabilization. Elife 2015;4.
401. Cross M, Nguyen T, Bogdanoska V, Reynolds E, Hamilton JA. A proteomics strategy for the enrichment of receptor-associated complexes. Proteomics 2005;5:4754-4763.
402. Watabe M, Nakaki T. Protein kinase CK2 regulates the formation and clearance of aggresomes in response to stress. J Cell Sci 2011;124:1519-1532.
403. Li J, Zhang W, Yang H, et al. Spatiotemporal profile of postsynaptic interactomes integrates components of complex brain disorders. Nat Neurosci 2017;20:1150-1161.
404. Seerapu HR, Borthakur S, Kong N, et al. The cytoplasmic domain of neuropilin-1 regulates focal adhesion turnover. FEBS Lett 2013;587:3392-3399.
405. Deribe YL, Wild P, Chandrashaker A, et al. Regulation of epidermal growth factor receptor trafficking by lysine deacetylase HDAC6. Sci Signal 2009;2:ra84.
406. Low TY, Peng M, Magliozzi R, Mohammed S, Guardavaccaro D, Heck AJ. A systems-wide screen identifies substrates of the SCFbetaTrCP ubiquitin ligase. Sci Signal 2014;7:rs8.
407. Brehme M, Hantschel O, Colinge J, et al. Charting the molecular network of the drug target Bcr-Abl. Proc Natl Acad Sci U S A 2009;106:7414-7419.
408. Bouwmeester T, Bauch A, Ruffner H, et al. A physical and functional map of the human TNF-alpha/NF-kappa B signal transduction pathway. Nat Cell Biol 2004;6:97-105.
409. Li KW, Chen N, Klemmer P, Koopmans F, Karupothula R, Smit AB. Identifying true protein complex constituents in interaction proteomics: the example of the DMXL2 protein complex. Proteomics 2012;12:2428-2432.
410. Koch HB, Zhang R, Verdoodt B, et al. Large-scale identification of c-MYC-associated proteins using a combined TAP/MudPIT approach. Cell Cycle 2007;6:205-217.
411. Liang Y, Yu W, Li Y, et al. Nudel functions in membrane traffic mainly through association with Lis1 and cytoplasmic dynein. J Cell Biol 2004;164:557-566.
412. Yan K, Li L, Wang X, et al. The deubiquitinating enzyme complex BRISC is required for proper mitotic spindle assembly in mammalian cells. J Cell Biol 2015;210:209-224.
413. Rex EB, Rankin ML, Yang Y, et al. Identification of RanBP 9/10 as interacting partners for protein kinase C (PKC) gamma/delta and the D1 dopamine receptor: regulation of PKC-mediated receptor phosphorylation. Mol Pharmacol 2010;78:69-80.
414. Tsai YC, Greco TM, Boonmee A, Miteva Y, Cristea IM. Functional proteomics establishes the interaction of SIRT7 with chromatin remodeling complexes and expands its role in regulation of RNA polymerase I transcription. Mol Cell Proteomics 2012;11:60-76.
415. Rashidian J, Le Scolan E, Ji X, et al. Ski regulates Hippo and TAZ signaling to suppress breast cancer progression. Sci Signal 2015;8:ra14.
416. Medina PP, Carretero J, Ballestar E, et al. Transcriptional targets of the chromatin-remodelling factor SMARCA4/BRG1 in lung cancer cells. Hum Mol Genet 2005;14:973-982.
417. Kim MS, Machida Y, Vashisht AA, Wohlschlegel JA, Pang YP, Machida YJ. Regulation of error-prone translesion synthesis by Spartan/C1orf124. Nucleic Acids Res 2013;41:1661-1668.

418. Humphries JD, Byron A, Bass MD, et al. Proteomic analysis of integrin-associated complexes identifies RCC2 as a dual regulator of Rac1 and Arf6. Sci Signal 2009;2:ra51.
419. Chang X, Fan Y, Karyala S, et al. Ligand-independent regulation of transforming growth factor beta1 expression and cell cycle progression by the aryl hydrocarbon receptor. Mol Cell Biol 2007;27:6127-6139.
420. Hulleman JD, Kelly JW. Genetic ablation of N-linked glycosylation reveals two key folding pathways for R345W fibulin-3, a secreted protein associated with retinal degeneration. FASEB J 2015;29:565-575.
421. Mendillo ML, Santagata S, Koeva M, et al. HSF1 drives a transcriptional program distinct from heat shock to support highly malignant human cancers. Cell 2012;150:549-562.
422. Wang J, Huo K, Ma L, et al. Toward an understanding of the protein interaction network of the human liver. Mol Syst Biol 2011;7:536.
423. Shu J, Wu C, Wu Y, et al. Induction of pluripotency in mouse somatic cells with lineage specifiers. Cell 2013;153:963-975.
424. Jessop CE, Chakravarthi S, Garbi N, Hammerling GJ, Lovell S, Bulleid NJ. ERp57 is essential for efficient folding of glycoproteins sharing common structural domains. EMBO J 2007;26:28-40.
425. Yuryev A, Wennogle LP. Novel raf kinase protein-protein interactions found by an exhaustive yeast two-hybrid analysis. Genomics 2003;81:112-125.
426. Bogachek MV, Chen Y, Kulak MV, et al. Sumoylation pathway is required to maintain the basal breast cancer subtype. Cancer Cell 2014;25:748-761.
427. Iyengar S, Ivanov AV, Jin VX, Rauscher FJ, 3rd, Farnham PJ. Functional analysis of KAP1 genomic recruitment. Mol Cell Biol 2011;31:1833-1847.
428. Ozaki T, Kondo K, Nakamura Y, Ichimiya S, Nakagawara A, Sakiyama S. Interaction of DA41, a DAN-binding protein, with the epidermal growth factor-like protein, S(1-5). Biochem Biophys Res Commun 1997;237:245-250.
429. Li S, Wang L, Fu B, Berman MA, Diallo A, Dorf ME. TRIM65 regulates microRNA activity by ubiquitination of TNRC6. Proc Natl Acad Sci U S A 2014;111:6970-6975.
430. Li X, Wang W, Wang J, et al. Proteomic analyses reveal distinct chromatin-associated and soluble transcription factor complexes. Mol Syst Biol 2015;11:775.
431. Kim JH, Lane WS, Reinberg D. Human Elongator facilitates RNA polymerase II transcription through chromatin. Proc Natl Acad Sci U S A 2002;99:1241-1246.
432. Oliviero G, Munawar N, Watson A, et al. The variant Polycomb Repressor Complex 1 component PCGF1 interacts with a pluripotency sub-network that includes DPPA4, a regulator of embryogenesis. Sci Rep 2015;5:18388.
433. Li Q, Fazly AM, Zhou H, Huang S, Zhang Z, Stillman B. The elongator complex interacts with PCNA and modulates transcriptional silencing and sensitivity to DNA damage agents. PLoS Genet 2009;5:e1000684.
434. Hawkes NA, Otero G, Winkler GS, et al. Purification and characterization of the human elongator complex. J Biol Chem 2002;277:3047-3052.
435. Barrios-Rodiles M, Brown KR, Ozdamar B, et al. High-throughput mapping of a dynamic signaling network in mammalian cells. Science 2005;307:1621-1625.
436. Yu HH, Zisch AH, Dodelet VC, Pasquale EB. Multiple signaling interactions of Abl and Arg kinases with the EphB2 receptor. Oncogene 2001;20:3995-4006.
437. Hitt B, Riordan SM, Kukreja L, Eimer WA, Rajapaksha TW, Vassar R. beta-Site amyloid precursor protein (APP)-cleaving enzyme 1 (BACE1)-deficient mice exhibit a close homolog of L1 (CHL1) loss-of-function phenotype involving axon guidance defects. J Biol Chem 2012;287:38408-38425.
438. Bowden TA, Aricescu AR, Nettleship JE, et al. Structural plasticity of eph receptor A4 facilitates cross-class ephrin signaling. Structure 2009;17:1386-1397.

439. Theil T, Frain M, Gilardi-Hebenstreit P, Flenniken A, Charnay P, Wilkinson DG. Segmental expression of the EphA4 (Sek-1) receptor tyrosine kinase in the hindbrain is under direct transcriptional control of Krox-20. Development 1998;125:443-452.
440. Fukai J, Yokote H, Yamanaka R, Arao T, Nishio K, Itakura T. EphA4 promotes cell proliferation and migration through a novel EphA4-FGFR1 signaling pathway in the human glioma U251 cell line. Mol Cancer Ther 2008;7:2768-2778.
441. Prevost N, Woulfe D, Tanaka T, Brass LF. Interactions between Eph kinases and ephrins provide a mechanism to support platelet aggregation once cell-to-cell contact has occurred. Proc Natl Acad Sci U S A 2002;99:9219-9224.
442. Kullander K, Mather NK, Diella F, Dottori M, Boyd AW, Klein R. Kinase-dependent and kinase-independent functions of EphA4 receptors in major axon tract formation in vivo. Neuron 2001;29:73-84.
443. Stadler HS, Higgins KM, Capecchi MR. Loss of Eph-receptor expression correlates with loss of cell adhesion and chondrogenic capacity in Hoxa13 mutant limbs. Development 2001;128:4177-4188.
444. Taipale M, Krykbaeva I, Koeva M, et al. Quantitative analysis of HSP90-client interactions reveals principles of substrate recognition. Cell 2012;150:987-1001.
445. Fenner BJ, Scannell M, Prehn JH. Expanding the substantial interactome of NEMO using protein microarrays. PLoS One 2010;5:e8799.
446. Prevost N, Woulfe DS, Jiang H, et al. Eph kinases and ephrins support thrombus growth and stability by regulating integrin outside-in signaling in platelets. Proc Natl Acad Sci U S A 2005;102:9820-9825.
447. Barrantes IB, Elia AJ, Wunsch K, et al. Interaction between Notch signalling and Lunatic fringe during somite boundary formation in the mouse. Curr Biol 1999;9:470-480.
448. Sakuraba J, Shintani T, Tani S, Noda M. Substrate specificity of R3 receptor-like protein-tyrosine phosphatase subfamily toward receptor protein-tyrosine kinases. J Biol Chem 2013;288:23421-23431.
449. Lai Y, Song M, Hakala K, Weintraub ST, Shiio Y. Proteomic dissection of the von Hippel-Lindau (VHL) interactome. J Proteome Res 2011;10:5175-5182.
450. Jones RB, Gordus A, Krall JA, MacBeath G. A quantitative protein interaction network for the ErbB receptors using protein microarrays. Nature 2006;439:168-174.
451. Kainulainen V, Sundvall M, Maatta JA, Santiestevan E, Klagsbrun M, Elenius K. A natural ErbB4 isoform that does not activate phosphoinositide 3-kinase mediates proliferation but not survival or chemotaxis. J Biol Chem 2000;275:8641-8649.
452. Carpenter G. ErbB-4: mechanism of action and biology. Exp Cell Res 2003;284:66-77.
453. Petschnigg J, Groisman B, Kotlyar M, et al. The mammalian-membrane two-hybrid assay (MaMTH) for probing membrane-protein interactions in human cells. Nat Methods 2014;11:585-592.
454. Schulze WX, Deng L, Mann M. Phosphotyrosine interactome of the ErbB-receptor kinase family. Mol Syst Biol 2005;1:2005 0008.
455. Ivarsson Y, Arnold R, McLaughlin M, et al. Large-scale interaction profiling of PDZ domains through proteomic peptide-phage display using human and viral phage peptidomes. Proc Natl Acad Sci U S A 2014;111:2542-2547.
456. Kiuchi T, Ortiz-Zapater E, Monypenny J, et al. The ErbB4 CYT2 variant protects EGFR from ligand-induced degradation to enhance cancer cell motility. Sci Signal 2014;7:ra78.
457. Bourguignon LY, Gilad E, Peyrollier K. Heregulin-mediated ErbB2-ERK signaling activates hyaluronan synthases leading to CD44-dependent ovarian tumor cell growth and migration. J Biol Chem 2007;282:19426-19441.
458. Fernandes AM, Hamburger AW, Gerwin BI. Dominance of ErbB-1 heterodimers in lung epithelial cells overexpressing ErbB-2. Both ErbB-1 and ErbB-2 contribute significantly to tumorigenicity. Am J Respir Cell Mol Biol 1999;21:701-709.

459. Sundvall M, Veikkolainen V, Kurppa K, et al. Cell death or survival promoted by alternative isoforms of ErbB4. Mol Biol Cell 2010;21:4275-4286.
460. Bjarnadottir M, Misner DL, Haverfield-Gross S, et al. Neuregulin1 (NRG1) signaling through Fyn modulates NMDA receptor phosphorylation: differential synaptic function in NRG1+/- knock-outs compared with wild-type mice. J Neurosci 2007;27:4519-4529.
461. Chen D, Xu LG, Chen L, Li L, Zhai Z, Shu HB. NIK is a component of the EGF/heregulin receptor signaling complexes. Oncogene 2003;22:4348-4355.
462. Hahn CG, Wang HY, Cho DS, et al. Altered neuregulin 1-erbB4 signaling contributes to NMDA receptor hypofunction in schizophrenia. Nat Med 2006;12:824-828.
463. Paatero I, Lassus H, Junttila TT, Kaskinen M, Butzow R, Elenius K. CYT-1 isoform of ErbB4 is an independent prognostic factor in serous ovarian cancer and selectively promotes ovarian cancer cell growth in vitro. Gynecol Oncol 2013;129:179-187.
464. Gur G, Rubin C, Katz M, et al. LRIG1 restricts growth factor signaling by enhancing receptor ubiquitylation and degradation. EMBO J 2004;23:3270-3281.
465. Zeng F, Zhang MZ, Singh AB, Zent R, Harris RC. ErbB4 isoforms selectively regulate growth factor induced Madin-Darby canine kidney cell tubulogenesis. Mol Biol Cell 2007;18:4446-4456.
466. Gilmore-Hebert M, Ramabhadran R, Stern DF. Interactions of ErbB4 and Kap1 connect the growth factor and DNA damage response pathways. Mol Cancer Res 2010;8:1388-1398.
467. Sardi SP, Murtie J, Koirala S, Patten BA, Corfas G. Presenilin-dependent ErbB4 nuclear signaling regulates the timing of astrogenesis in the developing brain. Cell 2006;127:185-197.
468. Zeng F, Xu J, Harris RC. Nedd4 mediates ErbB4 JM-a/CYT-1 ICD ubiquitination and degradation in MDCK II cells. FASEB J 2009;23:1935-1945.
469. Sundvall M, Korhonen A, Vaparanta K, et al. Protein inhibitor of activated STAT3 (PIAS3) protein promotes SUMOylation and nuclear sequestration of the intracellular domain of ErbB4 protein. J Biol Chem 2012;287:23216-23226.
470. Diamonti AJ, Guy PM, Ivanof C, Wong K, Sweeney C, Carraway KL, 3rd. An RBCC protein implicated in maintenance of steady-state neuregulin receptor levels. Proc Natl Acad Sci U S A 2002;99:2866-2871.
471. Williams CC, Allison JG, Vidal GA, et al. The ERBB4/HER4 receptor tyrosine kinase regulates gene expression by functioning as a STAT5A nuclear chaperone. J Cell Biol 2004;167:469-478.
472. Sundvall M, Korhonen A, Paatero I, et al. Isoform-specific monoubiquitination, endocytosis, and degradation of alternatively spliced ErbB4 isoforms. Proc Natl Acad Sci U S A 2008;105:4162-4167.
473. Meijer IM, van Rotterdam W, van Zoelen EJ, van Leeuwen JE. Cbl and Itch binding sites in ERBB4 CYT-1 and CYT-2 mediate K48- and K63-polyubiquitination, respectively. Cell Signal 2013;25:470-478.
474. Paronetto MP, Minana B, Valcarcel J. The Ewing sarcoma protein regulates DNA damage-induced alternative splicing. Mol Cell 2011;43:353-368.
475. Bararia D, Trivedi AK, Zada AA, et al. Proteomic identification of the MYST domain histone acetyltransferase TIP60 (HTATIP) as a co-activator of the myeloid transcription factor C/EBPalpha. Leukemia 2008;22:800-807.
476. Park JH, Kang HJ, Lee YK, et al. Inactivation of EWS reduces PGC-1alpha protein stability and mitochondrial homeostasis. Proc Natl Acad Sci U S A 2015;112:6074-6079.
477. Sankar S, Gomez NC, Bell R, et al. EWS and RE1-Silencing Transcription Factor Inhibit Neuronal Phenotype Development and Oncogenic Transformation in Ewing Sarcoma. Genes Cancer 2013;4:213-223.
478. Wang T, Jiang X, Chen G, Xu J. Interaction of amyotrophic lateral sclerosis/frontotemporal lobar degeneration-associated fused-in-sarcoma with proteins involved in metabolic and protein degradation pathways. Neurobiol Aging 2015;36:527-535.

479. Uranishi H, Tetsuka T, Yamashita M, et al. Involvement of the pro-oncoprotein TLS (translocated in liposarcoma) in nuclear factor-kappa B p65-mediated transcription as a coactivator. J Biol Chem 2001;276:13395-13401.
480. Sumara G, Formentini I, Collins S, et al. Regulation of PKD by the MAPK p38delta in insulin secretion and glucose homeostasis. Cell 2009;136:235-248.
481. Coady TH, Manley JL. ALS mutations in TLS/FUS disrupt target gene expression. Genes Dev 2015;29:1696-1706.
482. Eberle I, Pless B, Braun M, Dingermann T, Marschalek R. Transcriptional properties of human NANOG1 and NANOG2 in acute leukemic cells. Nucleic Acids Res 2010;38:5384-5395.
483. Pichlmair A, Kandasamy K, Alvisi G, et al. Viral immune modulators perturb the human molecular network by common and unique strategies. Nature 2012;487:486-490.
484. Oh SM, Liu Z, Okada M, et al. Ebp1 sumoylation, regulated by TLS/FUS E3 ligase, is required for its anti-proliferative activity. Oncogene 2010;29:1017-1030.
485. Gascoyne DM, Thomas GR, Latchman DS. The effects of Brn-3a on neuronal differentiation and apoptosis are differentially modulated by EWS and its oncogenic derivative EWS/Fli-1. Oncogene 2004;23:3830-3840.
486. Roy R, Durie D, Li H, et al. hnRNPA1 couples nuclear export and translation of specific mRNAs downstream of FGF-2/S6K2 signalling. Nucleic Acids Res 2014;42:12483-12497.
487. Powers CA, Mathur M, Raaka BM, Ron D, Samuels HH. TLS (translocated-in-liposarcoma) is a high-affinity interactor for steroid, thyroid hormone, and retinoid receptors. Mol Endocrinol 1998;12:4-18.
488. Behzadnia N, Golas MM, Hartmuth K, et al. Composition and three-dimensional EM structure of double affinity-purified, human prespliceosomal A complexes. EMBO J 2007;26:1737-1748.
489. So J, Pasculescu A, Dai AY, et al. Integrative analysis of kinase networks in TRAIL-induced apoptosis provides a source of potential targets for combination therapy. Sci Signal 2015;8:rs3.
490. Kim SH, Shanware NP, Bowler MJ, Tibbetts RS. Amyotrophic lateral sclerosis-associated proteins TDP-43 and FUS/TLS function in a common biochemical complex to co-regulate HDAC6 mRNA. J Biol Chem 2010;285:34097-34105.
491. Amoresano A, Di Costanzo A, Leo G, et al. Identification of DeltaNp63alpha protein interactions by mass spectrometry. J Proteome Res 2010;9:2042-2048.
492. Xu Y, Cai M, Yang Y, Huang L, Ye Y. SGTA recognizes a noncanonical ubiquitin-like domain in the Bag6-Ubl4A-Trc35 complex to promote endoplasmic reticulum-associated degradation. Cell Rep 2012;2:1633-1644.
493. de Hoog CL, Foster LJ, Mann M. RNA and RNA binding proteins participate in early stages of cell spreading through spreading initiation centers. Cell 2004;117:649-662.
494. Rapp TB, Yang L, Conrad EU, 3rd, Mandahl N, Chansky HA. RNA splicing mediated by YB-1 is inhibited by TLS/CHOP in human myxoid liposarcoma cells. J Orthop Res 2002;20:723-729.
495. Kasyapa CS, Kunapuli P, Cowell JK. Mass spectroscopy identifies the splicing-associated proteins, PSF, hnRNP H3, hnRNP A2/B1, and TLS/FUS as interacting partners of the ZNF198 protein associated with rearrangement in myeloproliferative disease. Exp Cell Res 2005;309:78-85.
496. Ebert SM, Dyle MC, Bullard SA, et al. Identification and Small Molecule Inhibition of an Activating Transcription Factor 4 (ATF4)-dependent Pathway to Age-related Skeletal Muscle Weakness and Atrophy. J Biol Chem 2015;290:25497-25511.
497. Sang Lee J, Gyu Park S, Park H, Seol W, Lee S, Kim S. Interaction network of human aminoacyl-tRNA synthetases and subunits of elongation factor 1 complex. Biochem Biophys Res Commun 2002;291:158-164.
498. Xie W, Nangle LA, Zhang W, Schimmel P, Yang XL. Long-range structural effects of a Charcot-Marie-Tooth disease-causing mutation in human glycyl-tRNA synthetase. Proc Natl Acad Sci U S A 2007;104:9976-9981.

499. Jin J, Li GJ, Davis J, et al. Identification of novel proteins associated with both alpha-synuclein and DJ-1. Mol Cell Proteomics 2007;6:845-859.
500. Fedorova OA, Moiseeva TN, Nikiforov AA, et al. Proteomic analysis of the 20S proteasome (PSMA3)-interacting proteins reveals a functional link between the proteasome and mRNA metabolism. Biochem Biophys Res Commun 2011;416:258-265.
501. Milev MP, Ravichandran M, Khan MF, Schriemer DC, Mouland AJ. Characterization of staufen1 ribonucleoproteins by mass spectrometry and biochemical analyses reveal the presence of diverse host proteins associated with human immunodeficiency virus type 1. Front Microbiol 2012;3:367.
502. Murn J, Zarnack K, Yang YJ, et al. Control of a neuronal morphology program by an RNA-binding zinc finger protein, Unkempt. Genes Dev 2015;29:501-512.
503. Diner BA, Lum KK, Javitt A, Cristea IM. Interactions of the Antiviral Factor Interferon Gamma-Inducible Protein 16 (IFI16) Mediate Immune Signaling and Herpes Simplex Virus-1 Immunosuppression. Mol Cell Proteomics 2015;14:2341-2356.
504. Folkmann AW, Collier SE, Zhan X, Aditi, Ohi MD, Wente SR. Gle1 functions during mRNA export in an oligomeric complex that is altered in human disease. Cell 2013;155:582-593.
505. Rayala HJ, Kendirgi F, Barry DM, Majerus PW, Wente SR. The mRNA export factor human Gle1 interacts with the nuclear pore complex protein Nup155. Mol Cell Proteomics 2004;3:145-155.
506. Daly RJ, Sanderson GM, Janes PW, Sutherland RL. Cloning and characterization of GRB14, a novel member of the GRB7 gene family. J Biol Chem 1996;271:12502-12510.
507. Reilly JF, Mickey G, Maher PA. Association of fibroblast growth factor receptor 1 with the adaptor protein Grb14. Characterization of a new receptor binding partner. J Biol Chem 2000;275:7771-7778.
508. Depetris RS, Hu J, Gimpelevich I, Holt LJ, Daly RJ, Hubbard SR. Structural basis for inhibition of the insulin receptor by the adaptor protein Grb14. Mol Cell 2005;20:325-333.
509. Bereziat V, Kasus-Jacobi A, Perdereau D, Cariou B, Girard J, Burnol AF. Inhibition of insulin receptor catalytic activity by the molecular adapter Grb14. J Biol Chem 2002;277:4845-4852.
510. King CC, Newton AC. The adaptor protein Grb14 regulates the localization of 3-phosphoinositide-dependent kinase-1. J Biol Chem 2004;279:37518-37527.
511. Cariou B, Perdereau D, Cailliau K, et al. The adapter protein ZIP binds Grb14 and regulates its inhibitory action on insulin signaling by recruiting protein kinase Czeta. Mol Cell Biol 2002;22:6959-6970.
512. Lyons RJ, Deane R, Lynch DK, et al. Identification of a novel human tankyrase through its interaction with the adaptor protein Grb14. J Biol Chem 2001;276:17172-17180.
513. Hoque M, Young TM, Lee CG, Serrero G, Mathews MB, Pe'ery T. The growth factor granulin interacts with cyclin T1 and modulates P-TEFb-dependent transcription. Mol Cell Biol 2003;23:1688-1702.
514. Thalappilly S, Suliman M, Gayet O, et al. Identification of multi-SH3 domain-containing protein interactome in pancreatic cancer: a yeast two-hybrid approach. Proteomics 2008;8:3071-3081.
515. Tong J, Taylor P, Moran MF. Proteomic analysis of the epidermal growth factor receptor (EGFR) interactome and post-translational modifications associated with receptor endocytosis in response to EGF and stress. Mol Cell Proteomics 2014;13:1644-1658.
516. Klemmer P, Smit AB, Li KW. Proteomics analysis of immuno-precipitated synaptic protein complexes. J Proteomics 2009;72:82-90.
517. Lu L, Hu S, Wei R, et al. The HECT type ubiquitin ligase NEDL2 is degraded by anaphase-promoting complex/cyclosome (APC/C)-Cdh1, and its tight regulation maintains the metaphase to anaphase transition. J Biol Chem 2013;288:35637-35650.
518. Gonzalez EM, Mongiat M, Slater SJ, Baffa R, Iozzo RV. A novel interaction between perlecan protein core and progranulin: potential effects on tumor growth. J Biol Chem 2003;278:38113-38116.
519. Sui D, Wilson JE. Interaction of insulin-like growth factor binding protein-4, Miz-1, leptin, lipocalin-type prostaglandin D synthase, and granulin precursor with the N-terminal half of type III hexokinase. Arch Biochem Biophys 2000;382:262-274.

520. Klinakis A, Lobry C, Abdel-Wahab O, et al. A novel tumour-suppressor function for the Notch pathway in myeloid leukaemia. Nature 2011;473:230-233.
521. Gauthier BR, Brun T, Sarret EJ, et al. Oligonucleotide microarray analysis reveals PDX1 as an essential regulator of mitochondrial metabolism in rat islets. J Biol Chem 2004;279:31121-31130.
522. Pilot-Storck F, Chopin E, Rual JF, et al. Interactome mapping of the phosphatidylinositol 3-kinase-mammalian target of rapamycin pathway identifies deformed epidermal autoregulatory factor-1 as a new glycogen synthase kinase-3 interactor. Mol Cell Proteomics 2010;9:1578-1593.
523. Varjosalo M, Sacco R, Stukalov A, et al. Interlaboratory reproducibility of large-scale human protein-complex analysis by standardized AP-MS. Nat Methods 2013;10:307-314.
524. Prudencio M, Jansen-West KR, Lee WC, et al. Misregulation of human sortilin splicing leads to the generation of a nonfunctional progranulin receptor. Proc Natl Acad Sci U S A 2012;109:21510-21515.
525. Jian J, Zhao S, Tian Q, et al. Progranulin directly binds to the CRD2 and CRD3 of TNFR extracellular domains. FEBS Lett 2013;587:3428-3436.
526. Zhou Y, Li L, Liu Q, et al. E3 ubiquitin ligase SIAH1 mediates ubiquitination and degradation of TRB3. Cell Signal 2008;20:942-948.
527. Wu C, Ma MH, Brown KR, et al. Systematic identification of SH3 domain-mediated human protein-protein interactions by peptide array target screening. Proteomics 2007;7:1775-1785.
528. Yang X, Zou P, Yao J, et al. Proteomic dissection of cell type-specific H2AX-interacting protein complex associated with hepatocellular carcinoma. J Proteome Res 2010;9:1402-1415.
529. Sang L, Miller JJ, Corbit KC, et al. Mapping the NPHP-JBTS-MKS protein network reveals ciliopathy disease genes and pathways. Cell 2011;145:513-528.
530. West AP, Jr., Bennett MJ, Sellers VM, Andrews NC, Enns CA, Bjorkman PJ. Comparison of the interactions of transferrin receptor and transferrin receptor 2 with transferrin and the hereditary hemochromatosis protein HFE. J Biol Chem 2000;275:38135-38138.
531. Das S, Ward SV, Markle D, Samuel CE. DNA damage-binding proteins and heterogeneous nuclear ribonucleoprotein A1 function as constitutive KCS element components of the interferon-inducible RNA-dependent protein kinase promoter. J Biol Chem 2004;279:7313-7321.
532. Wang S, Kollipara RK, Srivastava N, et al. Ablation of the oncogenic transcription factor ERG by deubiquitinase inhibition in prostate cancer. Proc Natl Acad Sci U S A 2014;111:4251-4256.
533. Coyaud E, Mis M, Laurent EM, et al. BioID-based Identification of Skp Cullin F-box (SCF)beta-TrCP1/2 E3 Ligase Substrates. Mol Cell Proteomics 2015;14:1781-1795.
534. Lee YJ, Wei HM, Chen LY, Li C. Localization of SERBP1 in stress granules and nucleoli. FEBS J 2014;281:352-364.
535. Johnson-Kerner BL, Garcia Diaz A, Ekins S, Wichterle H. Kelch Domain of Gigaxonin Interacts with Intermediate Filament Proteins Affected in Giant Axonal Neuropathy. PLoS One 2015;10:e0140157.
536. Jeronimo C, Forget D, Bouchard A, et al. Systematic analysis of the protein interaction network for the human transcription machinery reveals the identity of the 7SK capping enzyme. Mol Cell 2007;27:262-274.
537. Hamilton BJ, Burns CM, Nichols RC, Rigby WF. Modulation of AUUUA response element binding by heterogeneous nuclear ribonucleoprotein A1 in human T lymphocytes. The roles of cytoplasmic location, transcription, and phosphorylation. J Biol Chem 1997;272:28732-28741.
538. Greco TM, Yu F, Guise AJ, Cristea IM. Nuclear import of histone deacetylase 5 by requisite nuclear localization signal phosphorylation. Mol Cell Proteomics 2011;10:M110 004317.
539. Venables JP, Bourgeois CF, Dalgliesh C, Kister L, Stevenin J, Elliott DJ. Up-regulation of the ubiquitous alternative splicing factor Tra2beta causes inclusion of a germ cell-specific exon. Hum Mol Genet 2005;14:2289-2303.
540. Vermeulen M, Eberl HC, Matarese F, et al. Quantitative interaction proteomics and genome-wide profiling of epigenetic histone marks and their readers. Cell 2010;142:967-980.

541. Perez-Hernandez D, Gutierrez-Vazquez C, Jorge I, et al. The intracellular interactome of tetraspanin-enriched microdomains reveals their function as sorting machineries toward exosomes. J Biol Chem 2013;288:11649-11661.
542. Pichlmair A, Lassnig C, Eberle CA, et al. IFIT1 is an antiviral protein that recognizes 5'-triphosphate RNA. Nat Immunol 2011;12:624-630.
543. Leonard D, Ajuh P, Lamond AI, Legerski RJ. hLodestar/HuF2 interacts with CDC5L and is involved in pre-mRNA splicing. Biochem Biophys Res Commun 2003;308:793-801.
544. Close P, East P, Dirac-Svejstrup AB, et al. DBIRD complex integrates alternative mRNA splicing with RNA polymerase II transcript elongation. Nature 2012;484:386-389.
545. Zhao G, Simpson RU. Interaction between vitamin D receptor with caveolin-3 and regulation by 1,25-dihydroxyvitamin D3 in adult rat cardiomyocytes. J Steroid Biochem Mol Biol 2010;121:159-163.
546. Babic I, Anderson ES, Tanaka K, et al. EGFR mutation-induced alternative splicing of Max contributes to growth of glycolytic tumors in brain cancer. Cell Metab 2013;17:1000-1008.
547. Drissi R, Dubois ML, Douziech M, Boisvert FM. Quantitative Proteomics Reveals Dynamic Interactions of the Minichromosome Maintenance Complex (MCM) in the Cellular Response to Etoposide Induced DNA Damage. Mol Cell Proteomics 2015;14:2002-2013.
548. Shi Y, Yang Y, Hoang B, et al. Therapeutic potential of targeting IRES-dependent c-myc translation in multiple myeloma cells during ER stress. Oncogene 2016;35:1015-1024.
549. Fogeron ML, Muller H, Schade S, et al. LGALS3BP regulates centriole biogenesis and centrosome hypertrophy in cancer cells. Nat Commun 2013;4:1531.
550. Hayano T, Yanagida M, Yamauchi Y, Shinkawa T, Isobe T, Takahashi N. Proteomic analysis of human Nop56p-associated pre-ribosomal ribonucleoprotein complexes. Possible link between Nop56p and the nucleolar protein treacle responsible for Treacher Collins syndrome. J Biol Chem 2003;278:34309-34319.
551. Bett JS, Ibrahim AF, Garg AK, et al. The P-body component USP52/PAN2 is a novel regulator of HIF1A mRNA stability. Biochem J 2013;451:185-194.
552. Lehner B, Semple JI, Brown SE, Counsell D, Campbell RD, Sanderson CM. Analysis of a high-throughput yeast two-hybrid system and its use to predict the function of intracellular proteins encoded within the human MHC class III region. Genomics 2004;83:153-167.
553. Yang Z, Chang YJ, Miyamoto H, et al. Suppression of androgen receptor transactivation and prostate cancer cell growth by heterogeneous nuclear ribonucleoprotein A1 via interaction with androgen receptor coregulator ARA54. Endocrinology 2007;148:1340-1349.
554. Zhang X, Zhang J, Bauer A, et al. Fine-tuning BMP7 signalling in adipogenesis by UBE2O/E2-230K-mediated monoubiquitination of SMAD6. EMBO J 2013;32:996-1007.
555. McCracken S, Longman D, Marcon E, et al. Proteomic analysis of SRm160-containing complexes reveals a conserved association with cohesin. J Biol Chem 2005;280:42227-42236.
556. Heyd F, Lynch KW. Phosphorylation-dependent regulation of PSF by GSK3 controls CD45 alternative splicing. Mol Cell 2010;40:126-137.
557. Buxade M, Parra JL, Rousseau S, et al. The Mnks are novel components in the control of TNF alpha biosynthesis and phosphorylate and regulate hnRNP A1. Immunity 2005;23:177-189.
558. Haselmann V, Kurz A, Bertsch U, et al. Nuclear death receptor TRAIL-R2 inhibits maturation of let-7 and promotes proliferation of pancreatic and other tumor cells. Gastroenterology 2014;146:278-290.
559. Czubaty A, Girstun A, Kowalska-Loth B, et al. Proteomic analysis of complexes formed by human topoisomerase I. Biochim Biophys Acta 2005;1749:133-141.
560. Gilpin KM, Chang L, Monteiro MJ. ALS-linked mutations in ubiquilin-2 or hnRNPA1 reduce interaction between ubiquilin-2 and hnRNPA1. Hum Mol Genet 2015;24:2565-2577.
561. Lewis SM, Veyrier A, Hosszu Ungureanu N, Bonnal S, Vagner S, Holcik M. Subcellular relocalization of a trans-acting factor regulates XIAP IRES-dependent translation. Mol Biol Cell 2007;18:1302-1311.

562. Hock J, Weinmann L, Ender C, et al. Proteomic and functional analysis of Argonaute-containing mRNA-protein complexes in human cells. EMBO Rep 2007;8:1052-1060.
563. Bielli P, Busa R, Di Stasi SM, et al. The transcription factor FBI-1 inhibits SAM68-mediated BCL-X alternative splicing and apoptosis. EMBO Rep 2014;15:419-427.
564. Roy SJ, Glazkova I, Frechette L, et al. Novel, gel-free proteomics approach identifies RNF5 and JAMP as modulators of GPCR stability. Mol Endocrinol 2013;27:1245-1266.
565. Cho SW, Suzuki K, Miura Y, et al. Novel role of hnRNP-A2/B1 in modulating aryl hydrocarbon receptor ligand sensitivity. Arch Toxicol 2015;89:2027-2038.
566. Wolfgeher D, Dunn DM, Woodford MR, et al. The dynamic interactome of human Aha1 upon Y223 phosphorylation. Data Brief 2015;5:752-755.
567. Guha M, Tang W, Sondheimer N, Avadhani NG. Role of calcineurin, hnRNPA2 and Akt in mitochondrial respiratory stress-mediated transcription activation of nuclear gene targets. Biochim Biophys Acta 2010;1797:1055-1065.
568. Andresen CA, Smedegaard S, Sylvestersen KB, et al. Protein interaction screening for the ankyrin repeats and suppressor of cytokine signaling (SOCS) box (ASB) family identify Asb11 as a novel endoplasmic reticulum resident ubiquitin ligase. J Biol Chem 2014;289:2043-2054.
569. Goina E, Skoko N, Pagani F. Binding of DAZAP1 and hnRNPA1/A2 to an exonic splicing silencer in a natural BRCA1 exon 18 mutant. Mol Cell Biol 2008;28:3850-3860.
570. Bernhard OK, Cunningham AL, Sheil MM. Analysis of proteins copurifying with the CD4/lck complex using one-dimensional polyacrylamide gel electrophoresis and mass spectrometry: comparison with affinity-tag based protein detection and evaluation of different solubilization methods. J Am Soc Mass Spectrom 2004;15:558-567.
571. Souza-Rodrigues E, Estanyol JM, Friedrich-Heineken E, et al. Proteomic analysis of p16ink4a-binding proteins. Proteomics 2007;7:4102-4111.
572. Kosturko LD, Maggipinto MJ, D'Sa C, Carson JH, Barbarese E. The microtubule-associated protein tumor overexpressed gene binds to the RNA trafficking protein heterogeneous nuclear ribonucleoprotein A2. Mol Biol Cell 2005;16:1938-1947.
573. Bollig F, Winzen R, Gaestel M, Kostka S, Resch K, Holtmann H. Affinity purification of ARE-binding proteins identifies polyA-binding protein 1 as a potential substrate in MK2-induced mRNA stabilization. Biochem Biophys Res Commun 2003;301:665-670.
574. Pancetti F, Bosser R, Krehan A, Pyerin W, Itarte E, Bachs O. Heterogeneous nuclear ribonucleoprotein A2 interacts with protein kinase CK2. Biochem Biophys Res Commun 1999;260:17-22.
575. Sato S, Idogawa M, Honda K, et al. Beta-catenin interacts with the FUS proto-oncogene product and regulates pre-mRNA splicing. Gastroenterology 2005;129:1225-1236.
576. Prahl M, Vilborg A, Palmberg C, Jornvall H, Asker C, Wiman KG. The p53 target protein Wig-1 binds hnRNP A2/B1 and RNA Helicase A via RNA. FEBS Lett 2008;582:2173-2177.
577. Li J, Bennett K, Stukalov A, et al. Perturbation of the mutated EGFR interactome identifies vulnerabilities and resistance mechanisms. Mol Syst Biol 2013;9:705.
578. Lokireddy S, Wijesoma IW, Sze SK, McFarlane C, Kambadur R, Sharma M. Identification of atrogin-1-targeted proteins during the myostatin-induced skeletal muscle wasting. Am J Physiol Cell Physiol 2012;303:C512-529.
579. Yao P, Potdar AA, Ray PS, et al. The HILDA complex coordinates a conditional switch in the 3'-untranslated region of the VEGFA mRNA. PLoS Biol 2013;11:e1001635.
580. Thompson JW, Nagel J, Hoving S, et al. Quantitative Lys--Gly-Gly (diGly) proteomics coupled with inducible RNAi reveals ubiquitin-mediated proteolysis of DNA damage-inducible transcript 4 (DDIT4) by the E3 ligase HUWE1. J Biol Chem 2014;289:28942-28955.
581. Roux KJ, Kim DI, Raida M, Burke B. A promiscuous biotin ligase fusion protein identifies proximal and interacting proteins in mammalian cells. J Cell Biol 2012;196:801-810.

582. Hoek KS, Kidd GJ, Carson JH, Smith R. hnRNP A2 selectively binds the cytoplasmic transport sequence of myelin basic protein mRNA. Biochemistry 1998;37:7021-7029.
583. Gonzalez V, Guo K, Hurley L, Sun D. Identification and characterization of nucleolin as a c-myc G-quadruplex-binding protein. J Biol Chem 2009;284:23622-23635.
584. Liu Z, Oh SM, Okada M, et al. Human BRE1 is an E3 ubiquitin ligase for Ebp1 tumor suppressor. Mol Biol Cell 2009;20:757-768.
585. He Y, Smith R. Nuclear functions of heterogeneous nuclear ribonucleoproteins A/B. Cell Mol Life Sci 2009;66:1239-1256.
586. Vera J, Jaumot M, Estanyol JM, Brun S, Agell N, Bachs O. Heterogeneous nuclear ribonucleoprotein A2 is a SET-binding protein and a PP2A inhibitor. Oncogene 2006;25:260-270.
587. Dellis S, Strickland KC, McCrary WJ, Patel A, Stocum E, Wright CF. Protein interactions among the vaccinia virus late transcription factors. Virology 2004;329:328-336.
588. Fuller HR, Man NT, Lam le T, et al. The SMN interactome includes Myb-binding protein 1a. J Proteome Res 2010;9:556-563.
589. McFarland MA, Ellis CE, Markey SP, Nussbaum RL. Proteomics analysis identifies phosphorylation-dependent alpha-synuclein protein interactions. Mol Cell Proteomics 2008;7:2123-2137.
590. Freibaum BD, Chitta RK, High AA, Taylor JP. Global analysis of TDP-43 interacting proteins reveals strong association with RNA splicing and translation machinery. J Proteome Res 2010;9:1104-1120.
591. Mizuno H, Honda M, Shirasaki T, et al. Heterogeneous nuclear ribonucleoprotein A2/B1 in association with hTERT is a potential biomarker for hepatocellular carcinoma. Liver Int 2012;32:1146-1155.
592. Marchand B, Arsenault D, Raymond-Fleury A, Boisvert FM, Boucher MJ. Glycogen synthase kinase-3 (GSK3) inhibition induces prosurvival autophagic signals in human pancreatic cancer cells. J Biol Chem 2015;290:5592-5605.
593. Rousseau S, Morrice N, Peggie M, Campbell DG, Gaestel M, Cohen P. Inhibition of SAPK2a/p38 prevents hnRNP A0 phosphorylation by MAPKAP-K2 and its interaction with cytokine mRNAs. EMBO J 2002;21:6505-6514.
594. Miyasaka T, Morita M, Ito K, et al. Interaction of antiproliferative protein Tob with the CCR4-NOT deadenylase complex. Cancer Sci 2008;99:755-761.
595. Tsofack SP, Garand C, Sereduk C, et al. NONO and RALY proteins are required for YB-1 oxaliplatin induced resistance in colon adenocarcinoma cell lines. Mol Cancer 2011;10:145.
596. Galligan JT, Martinez-Noel G, Arndt V, et al. Proteomic analysis and identification of cellular interactors of the giant ubiquitin ligase HERC2. J Proteome Res 2015;14:953-966.
597. Joseph SK, Lin C, Pierson S, Thomas AP, Maranto AR. Heteroligomers of type-I and type-III inositol trisphosphate receptors in WB rat liver epithelial cells. J Biol Chem 1995;270:23310-23316.
598. Alzayady KJ, Chandrasekhar R, Yule DI. Fragmented inositol 1,4,5-trisphosphate receptors retain tetrameric architecture and form functional Ca2+ release channels. J Biol Chem 2013;288:11122-11134.
599. Sankar N, deTombe PP, Mignery GA. Calcineurin-NFATc Regulates Type 2 Inositol 1,4,5-Trisphosphate Receptor (InsP(3)R2) Expression during Cardiac Remodeling. Journal of Biological Chemistry 2014;289:6188-6198.
600. Kasahara Y, Tuder RM, Taraseviciene-Stewart L, et al. Inhibition of VEGF receptors causes lung cell apoptosis and emphysema. J Clin Invest 2000;106:1311-1319.
601. Wen Y, Edelman JL, Kang T, Sachs G. Lipocortin V may function as a signaling protein for vascular endothelial growth factor receptor-2/Flk-1. Biochem Biophys Res Commun 1999;258:713-721.
602. Wu T, Zhang B, Ye F, Xiao Z. A potential role for caveolin-1 in VEGF-induced fibronectin upregulation in mesangial cells: involvement of VEGFR2 and Src. Am J Physiol Renal Physiol 2013;304:F820-830.

603. Tremmel M, Matzke A, Albrecht I, et al. A CD44v6 peptide reveals a role of CD44 in VEGFR-2 signaling and angiogenesis. Blood 2009;114:5236-5244.
604. Jinesh GG, Choi W, Shah JB, Lee EK, Willis DL, Kamat AM. Blebbishields, the emergency program for cancer stem cells: sphere formation and tumorigenesis after apoptosis. Cell Death Differ 2013;20:382-395.
605. Kim YM, Hwang S, Kim YM, et al. Endostatin blocks vascular endothelial growth factor-mediated signaling via direct interaction with KDR/Flk-1. J Biol Chem 2002;277:27872-27879.
606. Liu CH, Chen TC, Chau GY, et al. Analysis of protein-protein interactions in cross-talk pathways reveals CRKL protein as a novel prognostic marker in hepatocellular carcinoma. Mol Cell Proteomics 2013;12:1335-1349.
607. Carmeliet P, Lampugnani MG, Moons L, et al. Targeted deficiency or cytosolic truncation of the VE-cadherin gene in mice impairs VEGF-mediated endothelial survival and angiogenesis. Cell 1999;98:147-157.
608. Pillai S, Kovacs M, Chellappan S. Regulation of vascular endothelial growth factor receptors by Rb and E2F1: role of acetylation. Cancer Res 2010;70:4931-4940.
609. D'Haene N, Sauvage S, Maris C, et al. VEGFR1 and VEGFR2 involvement in extracellular galectin-1- and galectin-3-induced angiogenesis. PLoS One 2013;8:e67029.
610. Nagarajan R, Svaren J, Le N, Araki T, Watson M, Milbrandt J. EGR2 mutations in inherited neuropathies dominant-negatively inhibit myelin gene expression. Neuron 2001;30:355-368.
611. Elvert G, Kappel A, Heidenreich R, et al. Cooperative interaction of hypoxia-inducible factor-2alpha (HIF-2alpha) and Ets-1 in the transcriptional activation of vascular endothelial growth factor receptor-2 (Flk-1). J Biol Chem 2003;278:7520-7530.
612. Teixeira FR, Manfiolli AO, Soares CS, Baqui MM, Koide T, Gomes MD. The F-box protein FBXO25 promotes the proteasome-dependent degradation of ELK-1 protein. J Biol Chem 2013;288:28152-28162.
613. Balli D, Zhang Y, Snyder J, Kalinichenko VV, Kalin TV. Endothelial cell-specific deletion of transcription factor FoxM1 increases urethane-induced lung carcinogenesis. Cancer Res 2011;71:40-50.
614. Minami T, Rosenberg RD, Aird WC. Transforming growth factor-beta 1-mediated inhibition of the flk-1/KDR gene is mediated by a 5'-untranslated region palindromic GATA site. J Biol Chem 2001;276:5395-5402.
615. Giorgetti-Peraldi S, Murdaca J, Mas JC, Van Obberghen E. The adapter protein, Grb10, is a positive regulator of vascular endothelial growth factor signaling. Oncogene 2001;20:3959-3968.
616. Lamalice L, Houle F, Huot J. Phosphorylation of Tyr1214 within VEGFR-2 triggers the recruitment of Nck and activation of Fyn leading to SAPK2/p38 activation and endothelial cell migration in response to VEGF. J Biol Chem 2006;281:34009-34020.
617. Jackson TA, Taylor HE, Sharma D, Desiderio S, Danoff SK. Vascular endothelial growth factor receptor-2: counter-regulation by the transcription factors, TFII-I and TFII-IRD1. J Biol Chem 2005;280:29856-29863.
618. Goyal A, Pal N, Concannon M, et al. Endorepellin, the angiostatic module of perlecan, interacts with both the alpha2beta1 integrin and vascular endothelial growth factor receptor 2 (VEGFR2): a dual receptor antagonism. J Biol Chem 2011;286:25947-25962.
619. Meyer RD, Sacks DB, Rahimi N. IQGAP1-dependent signaling pathway regulates endothelial cell proliferation and angiogenesis. PLoS One 2008;3:e3848.
620. Juliano RL. Signal transduction by cell adhesion receptors and the cytoskeleton: functions of integrins, cadherins, selectins, and immunoglobulin-superfamily members. Annu Rev Pharmacol Toxicol 2002;42:283-323.
621. Tugues S, Honjo S, Konig C, et al. Tetraspanin CD63 promotes vascular endothelial growth factor receptor 2-beta1 integrin complex formation, thereby regulating activation and downstream signaling in endothelial cells in vitro and in vivo. J Biol Chem 2013;288:19060-19071.

622. Koutsioumpa M, Poimenidi E, Pantazaka E, et al. Receptor protein tyrosine phosphatase beta/zeta is a functional binding partner for vascular endothelial growth factor. Mol Cancer 2015;14:19.
623. Mamluk R, Carvajal IM, Morse BA, et al. Anti-tumor effect of CT-322 as an adnectin inhibitor of vascular endothelial growth factor receptor-2. MAbs 2010;2:199-208.
624. Hale AT, Tian H, Anih E, et al. Endothelial Kruppel-like factor 4 regulates angiogenesis and the Notch signaling pathway. J Biol Chem 2014;289:12016-12028.
625. Croci DO, Cerliani JP, Dalotto-Moreno T, et al. Glycosylation-dependent lectin-receptor interactions preserve angiogenesis in anti-VEGF refractory tumors. Cell 2014;156:744-758.
626. Kohler EE, Cowan CE, Chatterjee I, Malik AB, Wary KK. NANOG induction of fetal liver kinase-1 (FLK1) transcription regulates endothelial cell proliferation and angiogenesis. Blood 2011;117:1761-1769.
627. Chen X, Liu Z, Xu J. The cooperative function of nuclear receptor coactivator 1 (NCOA1) and NCOA3 in placental development and embryo survival. Mol Endocrinol 2010;24:1917-1934.
628. Kang J, Yoo J, Lee S, et al. An exquisite cross-control mechanism among endothelial cell fate regulators directs the plasticity and heterogeneity of lymphatic endothelial cells. Blood 2010;116:140-150.
629. Tiberio L, Nascimbeni R, Villanacci V, et al. The decrease of mineralcorticoid receptor drives angiogenic pathways in colorectal cancer. PLoS One 2013;8:e59410.
630. Pan Q, Chanthery Y, Liang WC, et al. Blocking neuropilin-1 function has an additive effect with anti-VEGF to inhibit tumor growth. Cancer Cell 2007;11:53-67.
631. Rho SB, Song YJ, Lim MC, Lee SH, Kim BR, Park SY. Programmed cell death 6 (PDCD6) inhibits angiogenesis through PI3K/mTOR/p70S6K pathway by interacting of VEGFR-2. Cell Signal 2012;24:131-139.
632. Keller KE, Doctor ZM, Dwyer ZW, Lee YS. SAICAR induces protein kinase activity of PKM2 that is necessary for sustained proliferative signaling of cancer cells. Mol Cell 2014;53:700-709.
633. Cunningham SA, Arrate MP, Brock TA, Waxham MN. Interactions of FLT-1 and KDR with phospholipase C gamma: identification of the phosphotyrosine binding sites. Biochem Biophys Res Commun 1997;240:635-639.
634. Dougher M, Terman BI. Autophosphorylation of KDR in the kinase domain is required for maximal VEGF-stimulated kinase activity and receptor internalization. Oncogene 1999;18:1619-1627.
635. Yao Z, Darowski K, St-Denis N, et al. A Global Analysis of the Receptor Tyrosine Kinase-Protein Phosphatase Interactome. Mol Cell 2017;65:347-360.
636. Kinugasa M, Amano H, Satomi-Kobayashi S, et al. Necl-5/poliovirus receptor interacts with VEGFR2 and regulates VEGF-induced angiogenesis. Circ Res 2012;110:716-726.
637. D'Angelo G, Martini JF, Iiri T, Fantl WJ, Martial J, Weiner RI. 16K human prolactin inhibits vascular endothelial growth factor-induced activation of Ras in capillary endothelial cells. Mol Endocrinol 1999;13:692-704.
638. Chu LY, Ramakrishnan DP, Silverstein RL. Thrombospondin-1 modulates VEGF signaling via CD36 by recruiting SHP-1 to VEGFR2 complex in microvascular endothelial cells. Blood 2013;122:1822-1832.
639. Sun Z, Li X, Massena S, et al. VEGFR2 induces c-Src signaling and vascular permeability in vivo via the adaptor protein TSAd. J Exp Med 2012;209:1363-1377.
640. Adachi K, Nikaido I, Ohta H, et al. Context-dependent wiring of Sox2 regulatory networks for self-renewal of embryonic and trophoblast stem cells. Mol Cell 2013;52:380-392.
641. Bhattacharya R, Senbanerjee S, Lin Z, et al. Inhibition of vascular permeability factor/vascular endothelial growth factor-mediated angiogenesis by the Kruppel-like factor KLF2. J Biol Chem 2005;280:28848-28851.
642. Meissner M, Stein M, Urbich C, et al. PPARalpha activators inhibit vascular endothelial growth factor receptor-2 expression by repressing Sp1-dependent DNA binding and transactivation. Circ Res 2004;94:324-332.

643. Bartoli M, Gu X, Tsai NT, et al. Vascular endothelial growth factor activates STAT proteins in aortic endothelial cells. J Biol Chem 2000;275:33189-33192.
644. Korpelainen EI, Karkkainen M, Gunji Y, Vikkula M, Alitalo K. Endothelial receptor tyrosine kinases activate the STAT signaling pathway: mutant Tie-2 causing venous malformations signals a distinct STAT activation response. Oncogene 1999;18:1-8.
645. Bhattacharya R, Sinha S, Yang SP, et al. The neurotransmitter dopamine modulates vascular permeability in the endothelium. J Mol Signal 2008;3:14.
646. Affar el B, Gay F, Shi Y, et al. Essential dosage-dependent functions of the transcription factor yin yang 1 in late embryonic development and cell cycle progression. Mol Cell Biol 2006;26:3565-3581.
647. Galiano MR, Jha S, Ho TS, et al. A distal axonal cytoskeleton forms an intra-axonal boundary that controls axon initial segment assembly. Cell 2012;149:1125-1139.
648. Teng J, Rai T, Tanaka Y, et al. The KIF3 motor transports N-cadherin and organizes the developing neuroepithelium. Nat Cell Biol 2005;7:474-482.
649. Lolkema MP, Mans DA, Snijckers CM, et al. The von Hippel-Lindau tumour suppressor interacts with microtubules through kinesin-2. FEBS Lett 2007;581:4571-4576.
650. Blanchard F, Duplomb L, Raher S, et al. Mannose 6-Phosphate/Insulin-like growth factor II receptor mediates internalization and degradation of leukemia inhibitory factor but not signal transduction. J Biol Chem 1999;274:24685-24693.
651. Takaki E, Fujimoto M, Sugahara K, et al. Maintenance of olfactory neurogenesis requires HSF1, a major heat shock transcription factor in mice. J Biol Chem 2006;281:4931-4937.
652. Boulanger MJ, Bankovich AJ, Kortemme T, Baker D, Garcia KC. Convergent mechanisms for recognition of divergent cytokines by the shared signaling receptor gp130. Mol Cell 2003;12:577-589.
653. Dror N, Alter-Koltunoff M, Azriel A, et al. Identification of IRF-8 and IRF-1 target genes in activated macrophages. Mol Immunol 2007;44:338-346.
654. Hu B, Castillo E, Harewood L, et al. Multifocal epithelial tumors and field cancerization from loss of mesenchymal CSL signaling. Cell 2012;149:1207-1220.
655. Coqueret O, Gascan H. Functional interaction of STAT3 transcription factor with the cell cycle inhibitor p21WAF1/CIP1/SDI1. J Biol Chem 2000;275:18794-18800.
656. Souza PP, Palmqvist P, Lundberg P, et al. Interleukin-4 and interleukin-13 inhibit the expression of leukemia inhibitory factor and interleukin-11 in fibroblasts. Mol Immunol 2012;49:601-610.
657. Baxter EW, Milner J. p53 Regulates LIF expression in human medulloblastoma cells. J Neurooncol 2010;97:373-382.
658. Choi SY, Goldberg IJ, Curtiss LK, Cooper AD. Interaction between ApoB and hepatic lipase mediates the uptake of ApoB-containing lipoproteins. J Biol Chem 1998;273:20456-20462.
659. Doolittle MH, Ben-Zeev O, Bassilian S, Whitelegge JP, Peterfy M, Wong H. Hepatic lipase maturation: a partial proteome of interacting factors. J Lipid Res 2009;50:1173-1184.
660. Rufibach LE, Duncan SA, Battle M, Deeb SS. Transcriptional regulation of the human hepatic lipase (LIPC) gene promoter. J Lipid Res 2006;47:1463-1477.
661. de Boer AG, Gaillard PJ. Drug targeting to the brain. Annu Rev Pharmacol Toxicol 2007;47:323-355.
662. Sirvent A, Verhoeven AJ, Jansen H, et al. Farnesoid X receptor represses hepatic lipase gene expression. J Lipid Res 2004;45:2110-2115.
663. Phuc Le P, Friedman JR, Schug J, et al. Glucocorticoid receptor-dependent gene regulatory networks. PLoS Genet 2005;1:e16.
664. Horton LE, Templeton DJ. The cyclin box and C-terminus of cyclins A and E specify CDK activation and substrate specificity. Oncogene 1997;14:491-498.
665. Chang KH, Multani PS, Sun KH, et al. Nuclear envelope dispersion triggered by deregulated Cdk5 precedes neuronal death. Mol Biol Cell 2011;22:1452-1462.

666. Grebien F, Vedadi M, Getlik M, et al. Pharmacological targeting of the Wdr5-MLL interaction in C/EBPalpha N-terminal leukemia. Nat Chem Biol 2015;11:571-578.
667. Mathew R, Seiler MP, Scanlon ST, et al. BTB-ZF factors recruit the E3 ligase cullin 3 to regulate lymphoid effector programs. Nature 2012;491:618-621.
668. Pan X, Papasani M, Hao Y, et al. YY1 controls Igkappa repertoire and B-cell development, and localizes with condensin on the Igkappa locus. EMBO J 2013;32:1168-1182.
669. Holaska JM, Wilson KL. An emerin "proteome": purification of distinct emerin-containing complexes from HeLa cells suggests molecular basis for diverse roles including gene regulation, mRNA splicing, signaling, mechanosensing, and nuclear architecture. Biochemistry 2007;46:8897-8908.
670. Chen VC, Kristensen AR, Foster LJ, Naus CC. Association of connexin43 with E3 ubiquitin ligase TRIM21 reveals a mechanism for gap junction phosphodegron control. J Proteome Res 2012;11:6134-6146.
671. Liu GH, Barkho BZ, Ruiz S, et al. Recapitulation of premature ageing with iPSCs from Hutchinson-Gilford progeria syndrome. Nature 2011;472:221-225.
672. Ye Q, Worman HJ. Protein-protein interactions between human nuclear lamins expressed in yeast. Exp Cell Res 1995;219:292-298.
673. Liu GH, Qu J, Suzuki K, et al. Progressive degeneration of human neural stem cells caused by pathogenic LRRK2. Nature 2012;491:603-607.
674. Costa Y, Ding J, Theunissen TW, et al. NANOG-dependent function of TET1 and TET2 in establishment of pluripotency. Nature 2013;495:370-374.
675. Ma L, Tsai MY, Wang S, et al. Requirement for Nudel and dynein for assembly of the lamin B spindle matrix. Nat Cell Biol 2009;11:247-256.
676. Tabellini G, Bortul R, Aluigi M, et al. Binding of elements of protein kinase C-alpha regulatory domain to lamin B1. Cell Signal 2002;14:819-827.
677. Sandrock K, Bielek H, Schradi K, Schmidt G, Klugbauer N. The nuclear import of the small GTPase Rac1 is mediated by the direct interaction with karyopherin alpha2. Traffic 2010;11:198-209.
678. Brown MJ, Hallam JA, Liu Y, Yamada KM, Shaw S. Cutting edge: integration of human T lymphocyte cytoskeleton by the cytolinker plectin. J Immunol 2001;167:641-645.
679. Sanchez-Morgan N, Kirsch KH, Trackman PC, Sonenshein GE. The lysyl oxidase propeptide interacts with the receptor-type protein tyrosine phosphatase kappa and inhibits beta-catenin transcriptional activity in lung cancer cells. Mol Cell Biol 2011;31:3286-3297.
680. Horiguchi M, Inoue T, Ohbayashi T, et al. Fibulin-4 conducts proper elastogenesis via interaction with cross-linking enzyme lysyl oxidase. Proc Natl Acad Sci U S A 2009;106:19029-19034.
681. Lelievre E, Hinek A, Lupu F, Buquet C, Soncin F, Mattot V. VE-statin/egfl7 regulates vascular elastogenesis by interacting with lysyl oxidases. EMBO J 2008;27:1658-1670.
682. Wang V, Davis DA, Haque M, Huang LE, Yarchoan R. Differential gene up-regulation by hypoxia-inducible factor-1alpha and hypoxia-inducible factor-2alpha in HEK293T cells. Cancer Res 2005;65:3299-3306.
683. Giampuzzi M, Oleggini R, Di Donato A. Demonstration of in vitro interaction between tumor suppressor lysyl oxidase and histones H1 and H2: definition of the regions involved. Biochim Biophys Acta 2003;1647:245-251.
684. Ostareck-Lederer A, Ostareck DH, Cans C, et al. c-Src-mediated phosphorylation of hnRNP K drives translational activation of specifically silenced mRNAs. Mol Cell Biol 2002;22:4535-4543.
685. Sato S, Trackman PC, Maki JM, Myllyharju J, Kirsch KH, Sonenshein GE. The Ras signaling inhibitor LOX-PP interacts with Hsp70 and c-Raf to reduce Erk activation and transformed phenotype of breast cancer cells. Mol Cell Biol 2011;31:2683-2695.
686. Sipione S, Rigamonti D, Valenza M, et al. Early transcriptional profiles in huntingtin-inducible striatal cells by microarray analyses. Hum Mol Genet 2002;11:1953-1965.

687. Martinez-Martinez E, Rodriguez C, Galan M, et al. The lysyl oxidase inhibitor (beta-aminopropionitrile) reduces leptin profibrotic effects and ameliorates cardiovascular remodeling in diet-induced obesity in rats. J Mol Cell Cardiol 2016;92:96-104.
688. Okkelman IA, Sukaeva AZ, Kirukhina EV, Korneenko TV, Pestov NB. Nuclear translocation of lysyl oxidase is promoted by interaction with transcription repressor p66beta. Cell Tissue Res 2014;358:481-489.
689. Jeay S, Pianetti S, Kagan HM, Sonenshein GE. Lysyl oxidase inhibits ras-mediated transformation by preventing activation of NF-kappa B. Mol Cell Biol 2003;23:2251-2263.
690. Sahlgren C, Gustafsson MV, Jin S, Poellinger L, Lendahl U. Notch signaling mediates hypoxia-induced tumor cell migration and invasion. Proc Natl Acad Sci U S A 2008;105:6392-6397.
691. Yu Z, Sato S, Trackman PC, Kirsch KH, Sonenshein GE. Blimp1 activation by AP-1 in human lung cancer cells promotes a migratory phenotype and is inhibited by the lysyl oxidase propeptide. PLoS One 2012;7:e33287.
692. Sato S, Zhao Y, Imai M, et al. Inhibition of CIN85-mediated invasion by a novel SH3 domain binding motif in the lysyl oxidase propeptide. PLoS One 2013;8:e77288.
693. Cox JL, Wilder PJ, Gilmore JM, Wuebben EL, Washburn MP, Rizzino A. The SOX2-interactome in brain cancer cells identifies the requirement of MSI2 and USP9X for the growth of brain tumor cells. PLoS One 2013;8:e62857.
694. He F, Melamed J, Tang MS, Huang C, Wu XR. Oncogenic HRAS Activates Epithelial-to-Mesenchymal Transition and Confers Stemness to p53-Deficient Urothelial Cells to Drive Muscle Invasion of Basal Subtype Carcinomas. Cancer Res 2015;75:2017-2028.
695. Dunlevy JR, Rada JA. Interaction of lumican with aggrecan in the aging human sclera. Invest Ophthalmol Vis Sci 2004;45:3849-3856.
696. Frye M, Gardner C, Li ER, Arnold I, Watt FM. Evidence that Myc activation depletes the epidermal stem cell compartment by modulating adhesive interactions with the local microenvironment. Development 2003;130:2793-2808.
697. Willy PJ, Murray IR, Qian J, et al. Regulation of PPARgamma coactivator 1alpha (PGC-1alpha) signaling by an estrogen-related receptor alpha (ERRalpha) ligand. Proc Natl Acad Sci U S A 2004;101:8912-8917.
698. Ou XM, Chen K, Shih JC. Dual functions of transcription factors, transforming growth factor-beta-inducible early gene (TIEG)2 and Sp3, are mediated by CACCC element and Sp1 sites of human monoamine oxidase (MAO) B gene. J Biol Chem 2004;279:21021-21028.
699. Wong WK, Ou XM, Chen K, Shih JC. Activation of human monoamine oxidase B gene expression by a protein kinase C MAPK signal transduction pathway involves c-Jun and Egr-1. J Biol Chem 2002;277:22222-22230.
700. Derkinderen P, Scales TM, Hanger DP, et al. Tyrosine 394 is phosphorylated in Alzheimer's paired helical filament tau and in fetal tau with c-Abl as the candidate tyrosine kinase. J Neurosci 2005;25:6584-6593.
701. Tremblay MA, Acker CM, Davies P. Tau phosphorylated at tyrosine 394 is found in Alzheimer's disease tangles and can be a product of the Abl-related kinase, Arg. J Alzheimers Dis 2010;19:721-733.
702. Park H, Kam TI, Kim Y, et al. Neuropathogenic role of adenylate kinase-1 in Abeta-mediated tau phosphorylation via AMPK and GSK3beta. Hum Mol Genet 2012;21:2725-2737.
703. Dickey CA, Koren J, Zhang YJ, et al. Akt and CHIP coregulate tau degradation through coordinated interactions. Proc Natl Acad Sci U S A 2008;105:3622-3627.
704. Guo JP, Arai T, Miklossy J, McGeer PL. Abeta and tau form soluble complexes that may promote self aggregation of both into the insoluble forms observed in Alzheimer's disease. Proc Natl Acad Sci U S A 2006;103:1953-1958.

705. Elliott E, Tsvetkov P, Ginzburg I. BAG-1 associates with Hsc70.Tau complex and regulates the proteasomal degradation of Tau protein. J Biol Chem 2007;282:37276-37284.
706. Padilla R, Maccioni RB, Avila J. Calmodulin binds to a tubulin binding site of the microtubule-associated protein tau. Mol Cell Biochem 1990;97:35-41.
707. Virdee K, Yoshida H, Peak-Chew S, Goedert M. Phosphorylation of human microtubule-associated protein tau by protein kinases of the AGC subfamily. FEBS Lett 2007;581:2657-2662.
708. Gamblin TC, Chen F, Zambrano A, et al. Caspase cleavage of tau: linking amyloid and neurofibrillary tangles in Alzheimer's disease. Proc Natl Acad Sci U S A 2003;100:10032-10037.
709. Vincent I, Jicha G, Rosado M, Dickson DW. Aberrant expression of mitotic cdc2/cyclin B1 kinase in degenerating neurons of Alzheimer's disease brain. J Neurosci 1997;17:3588-3598.
710. Jinwal UK, Trotter JH, Abisambra JF, et al. The Hsp90 kinase co-chaperone Cdc37 regulates tau stability and phosphorylation dynamics. J Biol Chem 2011;286:16976-16983.
711. Sasaki K, Shimura H, Itaya M, et al. Excitatory amino acid transporter 2 associates with phosphorylated tau and is localized in neurofibrillary tangles of tauopathic brains. FEBS Lett 2009;583:2194-2200.
712. Mendoza J, Sekiya M, Taniguchi T, Iijima KM, Wang R, Ando K. Global analysis of phosphorylation of tau by the checkpoint kinases Chk1 and Chk2 in vitro. J Proteome Res 2013;12:2654-2665.
713. Goldbaum O, Richter-Landsberg C. Proteolytic stress causes heat shock protein induction, tau ubiquitination, and the recruitment of ubiquitin to tau-positive aggregates in oligodendrocytes in culture. J Neurosci 2004;24:5748-5757.
714. Sadik G, Tanaka T, Kato K, et al. Phosphorylation of tau at Ser214 mediates its interaction with 14-3-3 protein: implications for the mechanism of tau aggregation. J Neurochem 2009;108:33-43.
715. Paquet C, Mouton-Liger F, Meurs EF, et al. The PKR activator PACT is induced by Abeta: involvement in Alzheimer's disease. Brain Pathol 2012;22:219-229.
716. Chambraud B, Sardin E, Giustiniani J, et al. A role for FKBP52 in Tau protein function. Proc Natl Acad Sci U S A 2010;107:2658-2663.
717. Reynolds CH, Garwood CJ, Wray S, et al. Phosphorylation regulates tau interactions with Src homology 3 domains of phosphatidylinositol 3-kinase, phospholipase Cgamma1, Grb2, and Src family kinases. J Biol Chem 2008;283:18177-18186.
718. Karagoz GE, Duarte AM, Akoury E, et al. Hsp90-Tau complex reveals molecular basis for specificity in chaperone action. Cell 2014;156:963-974.
719. Jinwal UK, Akoury E, Abisambra JF, et al. Imbalance of Hsp70 family variants fosters tau accumulation. FASEB J 2013;27:1450-1459.
720. Mills J, Digicaylioglu M, Legg AT, et al. Role of integrin-linked kinase in nerve growth factor-stimulated neurite outgrowth. J Neurosci 2003;23:1638-1648.
721. Schubert M, Gautam D, Surjo D, et al. Role for neuronal insulin resistance in neurodegenerative diseases. Proc Natl Acad Sci U S A 2004;101:3100-3105.
722. Awasthi S, Sharma A, Wong K, et al. A human T-cell lymphotropic virus type 1 enhancer of Myc transforming potential stabilizes Myc-TIP60 transcriptional interactions. Mol Cell Biol 2005;25:6178-6198.
723. Utton MA, Noble WJ, Hill JE, Anderton BH, Hanger DP. Molecular motors implicated in the axonal transport of tau and alpha-synuclein. J Cell Sci 2005;118:4645-4654.
724. Kawakami F, Yabata T, Ohta E, et al. LRRK2 phosphorylates tubulin-associated tau but not the free molecule: LRRK2-mediated regulation of the tau-tubulin association and neurite outgrowth. PLoS One 2012;7:e30834.
725. Salehi A, Delcroix JD, Mobley WC. Traffic at the intersection of neurotrophic factor signaling and neurodegeneration. Trends Neurosci 2003;26:73-80.

726. Martin B, Brenneman R, Becker KG, Gucek M, Cole RN, Maudsley S. iTRAQ analysis of complex proteome alterations in 3xTgAD Alzheimer's mice: understanding the interface between physiology and disease. PLoS One 2008;3:e2750.
727. Goedert M, Hasegawa M, Jakes R, Lawler S, Cuenda A, Cohen P. Phosphorylation of microtubule-associated protein tau by stress-activated protein kinases. FEBS Lett 1997;409:57-62.
728. Gu GJ, Lund H, Wu D, et al. Role of individual MARK isoforms in phosphorylation of tau at Ser(2)(6)(2) in Alzheimer's disease. Neuromolecular Med 2013;15:458-469.
729. Tang Z, Bereczki E, Zhang H, et al. Mammalian target of rapamycin (mTor) mediates tau protein dyshomeostasis: implication for Alzheimer disease. J Biol Chem 2013;288:15556-15570.
730. Xu LR, Liu XL, Chen J, Liang Y. Protein disulfide isomerase interacts with tau protein and inhibits its fibrillization. PLoS One 2013;8:e76657.
731. Lu PJ, Wulf G, Zhou XZ, Davies P, Lu KP. The prolyl isomerase Pin1 restores the function of Alzheimer-associated phosphorylated tau protein. Nature 1999;399:784-788.
732. Taniguchi T, Kawamata T, Mukai H, et al. Phosphorylation of tau is regulated by PKN. J Biol Chem 2001;276:10025-10031.
733. Flores-Delgado G, Liu CW, Sposto R, Berndt N. A limited screen for protein interactions reveals new roles for protein phosphatase 1 in cell cycle control and apoptosis. J Proteome Res 2007;6:1165-1175.
734. Eroglu B, Moskophidis D, Mivechi NF. Loss of Hsp110 leads to age-dependent tau hyperphosphorylation and early accumulation of insoluble amyloid beta. Mol Cell Biol 2010;30:4626-4643.
735. Lloret A, Badia MC, Giraldo E, et al. Amyloid-beta toxicity and tau hyperphosphorylation are linked via RCAN1 in Alzheimer's disease. J Alzheimers Dis 2011;27:701-709.
736. Liu F, Zaidi T, Iqbal K, Grundke-Iqbal I, Gong CX. Aberrant glycosylation modulates phosphorylation of tau by protein kinase A and dephosphorylation of tau by protein phosphatase 2A and 5. Neuroscience 2002;115:829-837.
737. Babu JR, Geetha T, Wooten MW. Sequestosome 1/p62 shuttles polyubiquitinated tau for proteasomal degradation. J Neurochem 2005;94:192-203.
738. Ohkubo N, Lee YD, Morishima A, et al. Apolipoprotein E and Reelin ligands modulate tau phosphorylation through an apolipoprotein E receptor/disabled-1/glycogen synthase kinase-3beta cascade. FASEB J 2003;17:295-297.
739. Kawakami F, Suzuki M, Shimada N, et al. Stimulatory effect of alpha-synuclein on the tau-phosphorylation by GSK-3beta. FEBS J 2011;278:4895-4904.
740. Bhaskar K, Yen SH, Lee G. Disease-related modifications in tau affect the interaction between Fyn and Tau. J Biol Chem 2005;280:35119-35125.
741. Hong Y, Chan CB, Kwon IS, et al. SRPK2 phosphorylates tau and mediates the cognitive defects in Alzheimer's disease. J Neurosci 2012;32:17262-17272.
742. Villace P, Marion RM, Ortin J. The composition of Staufen-containing RNA granules from human cells indicates their role in the regulated transport and translation of messenger RNAs. Nucleic Acids Res 2004;32:2411-2420.
743. Shimura H, Schwartz D, Gygi SP, Kosik KS. CHIP-Hsc70 complex ubiquitinates phosphorylated tau and enhances cell survival. J Biol Chem 2004;279:4869-4876.
744. Liu C, Song X, Nisbet R, Gotz J. Co-immunoprecipitation with Tau Isoform-specific Antibodies Reveals Distinct Protein Interactions and Highlights a Putative Role for 2N Tau in Disease. J Biol Chem 2016;291:8173-8188.
745. Flach K, Ramminger E, Hilbrich I, et al. Axotrophin/MARCH7 acts as an E3 ubiquitin ligase and ubiquitinates tau protein in vitro impairing microtubule binding. Biochim Biophys Acta 2014;1842:1527-1538.

746. Souter S, Lee G. Microtubule-associated protein tau in human prostate cancer cells: isoforms, phosphorylation, and interactions. J Cell Biochem 2009;108:555-564.
747. Sze CI, Su M, Pugazhenthi S, et al. Down-regulation of WW domain-containing oxidoreductase induces Tau phosphorylation in vitro. A potential role in Alzheimer's disease. J Biol Chem 2004;279:30498-30506.
748. Nellist M, Goedbloed MA, de Winter C, et al. Identification and characterization of the interaction between tuberin and 14-3-3zeta. J Biol Chem 2002;277:39417-39424.
749. Salton M, Elkon R, Borodina T, et al. Matrin 3 binds and stabilizes mRNA. PLoS One 2011;6:e23882.
750. Shen X, Valencia CA, Szostak JW, Dong B, Liu R. Scanning the human proteome for calmodulin-binding proteins. Proc Natl Acad Sci U S A 2005;102:5969-5974.
751. Hennig S, Kong G, Mannen T, et al. Prion-like domains in RNA binding proteins are essential for building subnuclear paraspeckles. J Cell Biol 2015;210:529-539.
752. Singh G, Kucukural A, Cenik C, et al. The cellular EJC interactome reveals higher-order mRNP structure and an EJC-SR protein nexus. Cell 2012;151:750-764.
753. Arjomand A, Baker MA, Li C, et al. The alpha-importome of mammalian germ cell maturation provides novel insights for importin biology. FASEB J 2014;28:3480-3493.
754. Wang BS, Liu YZ, Yang Y, et al. Autophagy negatively regulates cancer cell proliferation via selectively targeting VPRBP. Clin Sci (Lond) 2013;124:203-214.
755. Issaeva I, Zonis Y, Rozovskaia T, et al. Knockdown of ALR (MLL2) reveals ALR target genes and leads to alterations in cell adhesion and growth. Mol Cell Biol 2007;27:1889-1903.
756. Polydorides AD, Okano HJ, Yang YY, Stefani G, Darnell RB. A brain-enriched polypyrimidine tract-binding protein antagonizes the ability of Nova to regulate neuron-specific alternative splicing. Proc Natl Acad Sci U S A 2000;97:6350-6355.
757. Matafora V, D'Amato A, Mori S, Blasi F, Bachi A. Proteomics analysis of nucleolar SUMO-1 target proteins upon proteasome inhibition. Mol Cell Proteomics 2009;8:2243-2255.
758. Green MR, Monti S, Dalla-Favera R, et al. Signatures of murine B-cell development implicate Yy1 as a regulator of the germinal center-specific program. P Natl Acad Sci USA 2011;108:2873-2878.
759. Gingrich JR, Pelkey KA, Fam SR, et al. Unique domain anchoring of Src to synaptic NMDA receptors via the mitochondrial protein NADH dehydrogenase subunit 2. Proc Natl Acad Sci U S A 2004;101:6237-6242.
760. Ostrowski J, Wyrwicz L, Rychlewski L, Bomsztyk K. Heterogeneous nuclear ribonucleoprotein K protein associates with multiple mitochondrial transcripts within the organelle. J Biol Chem 2002;277:6303-6310.
761. Budihardjo I, Oliver H, Lutter M, Luo X, Wang X. Biochemical pathways of caspase activation during apoptosis. Annu Rev Cell Dev Biol 1999;15:269-290.
762. Davoodi J, Lin L, Kelly J, Liston P, MacKenzie AE. Neuronal apoptosis-inhibitory protein does not interact with Smac and requires ATP to bind caspase-9. J Biol Chem 2004;279:40622-40628.
763. Burstein E, Ganesh L, Dick RD, et al. A novel role for XIAP in copper homeostasis through regulation of MURR1. EMBO J 2004;23:244-254.
764. Sanna MG, da Silva Correia J, Ducrey O, et al. IAP suppression of apoptosis involves distinct mechanisms: the TAK1/JNK1 signaling cascade and caspase inhibition. Mol Cell Biol 2002;22:1754-1766.
765. Nguyen MD, Lariviere RC, Julien JP. Deregulation of Cdk5 in a mouse model of ALS: toxicity alleviated by perikaryal neurofilament inclusions. Neuron 2001;30:135-147.
766. Hallows JL, Chen K, DePinho RA, Vincent I. Decreased cyclin-dependent kinase 5 (cdk5) activity is accompanied by redistribution of cdk5 and cytoskeletal proteins and increased cytoskeletal protein phosphorylation in p35 null mice. J Neurosci 2003;23:10633-10644.
767. Yang Y, Dowling J, Yu QC, Kouklis P, Cleveland DW, Fuchs E. An essential cytoskeletal linker protein connecting actin microfilaments to intermediate filaments. Cell 1996;86:655-665.

768. Bourquin JP, Subramanian A, Langebrake C, et al. Identification of distinct molecular phenotypes in acute megakaryoblastic leukemia by gene expression profiling. Proc Natl Acad Sci U S A 2006;103:3339-3344.
769. Takeda K, Hatai T, Hamazaki TS, Nishitoh H, Saitoh M, Ichijo H. Apoptosis signal-regulating kinase 1 (ASK1) induces neuronal differentiation and survival of PC12 cells. J Biol Chem 2000;275:9805-9813.
770. Veeranna, Amin ND, Ahn NG, et al. Mitogen-activated protein kinases (Erk1,2) phosphorylate Lys-Ser-Pro (KSP) repeats in neurofilament proteins NF-H and NF-M. J Neurosci 1998;18:4008-4021.
771. Ching GY, Liem RK. RE1 silencing transcription factor is involved in regulating neuron-specific expression of alpha-internexin and neurofilament genes. J Neurochem 2009;109:1610-1623.
772. Nguyen MD, Lariviere RC, Julien JP. Reduction of axonal caliber does not alleviate motor neuron disease caused by mutant superoxide dismutase 1. Proc Natl Acad Sci U S A 2000;97:12306-12311.
773. Kesavapany S, Patel V, Zheng YL, et al. Inhibition of Pin1 reduces glutamate-induced perikaryal accumulation of phosphorylated neurofilament-H in neurons. Mol Biol Cell 2007;18:3645-3655.
774. Mukai H, Toshimori M, Shibata H, et al. PKN associates and phosphorylates the head-rod domain of neurofilament protein. J Biol Chem 1996;271:9816-9822.
775. Lucas EK, Dougherty SE, McMeekin LJ, et al. PGC-1alpha provides a transcriptional framework for synchronous neurotransmitter release from parvalbumin-positive interneurons. J Neurosci 2014;34:14375-14387.
776. Cluskey S, Ramsden DB. Mechanisms of neurodegeneration in amyotrophic lateral sclerosis. Mol Pathol 2001;54:386-392.
777. Khalili K, Del Valle L, Muralidharan V, et al. Puralpha is essential for postnatal brain development and developmentally coupled cellular proliferation as revealed by genetic inactivation in the mouse. Mol Cell Biol 2003;23:6857-6875.
778. Bocquet A, Berges R, Frank R, Robert P, Peterson AC, Eyer J. Neurofilaments bind tubulin and modulate its polymerization. J Neurosci 2009;29:11043-11054.
779. Murata H, Tajima N, Nagashima Y, et al. Von Hippel-Lindau tumor suppressor protein transforms human neuroblastoma cells into functional neuron-like cells. Cancer Res 2002;62:7004-7011.
780. Letwin K, Mizzen L, Motro B, Ben-David Y, Bernstein A, Pawson T. A mammalian dual specificity protein kinase, Nek1, is related to the NIMA cell cycle regulator and highly expressed in meiotic germ cells. EMBO J 1992;11:3521-3531.
781. Lehner B, Sanderson CM. A protein interaction framework for human mRNA degradation. Genome Res 2004;14:1315-1323.
782. Surpili MJ, Delben TM, Kobarg J. Identification of proteins that interact with the central coiled-coil region of the human protein kinase NEK1. Biochemistry 2003;42:15369-15376.
783. Yim H, Sung CK, You J, Tian Y, Benjamin T. Nek1 and TAZ interact to maintain normal levels of polycystin 2. J Am Soc Nephrol 2011;22:832-837.
784. Patil M, Pabla N, Huang S, Dong Z. Nek1 phosphorylates Von Hippel-Lindau tumor suppressor to promote its proteasomal degradation and ciliary destabilization. Cell Cycle 2013;12:166-171.
785. Tang M, Pelkey KA, Ng D, et al. Neto1 is an auxiliary subunit of native synaptic kainate receptors. J Neurosci 2011;31:10009-10018.
786. Sando R, 3rd, Gounko N, Pieraut S, Liao L, Yates J, 3rd, Maximov A. HDAC4 governs a transcriptional program essential for synaptic plasticity and memory. Cell 2012;151:821-834.
787. Renou JP, Bierie B, Miyoshi K, et al. Identification of genes differentially expressed in mouse mammary epithelium transformed by an activated beta-catenin. Oncogene 2003;22:4594-4610.
788. Kamenisch Y, Fousteri M, Knoch J, et al. Proteins of nucleotide and base excision repair pathways interact in mitochondria to protect from loss of subcutaneous fat, a hallmark of aging. J Exp Med 2010;207:379-390.

789. Noren Hooten N, Fitzpatrick M, Kompaniez K, et al. Coordination of DNA repair by NEIL1 and PARP-1: a possible link to aging. Aging (Albany NY) 2012;4:674-685.
790. Dantzer F, Luna L, Bjoras M, Seeberg E. Human OGG1 undergoes serine phosphorylation and associates with the nuclear matrix and mitotic chromatin in vivo. Nucleic Acids Res 2002;30:2349-2357.
791. Youn CK, Kim SH, Lee DY, et al. Cadmium down-regulates human OGG1 through suppression of Sp1 activity. J Biol Chem 2005;280:25185-25195.
792. Achanta G, Huang P. Role of p53 in sensing oxidative DNA damage in response to reactive oxygen species-generating agents. Cancer Res 2004;64:6233-6239.
793. D'Errico M, Parlanti E, Teson M, et al. New functions of XPC in the protection of human skin cells from oxidative damage. EMBO J 2006;25:4305-4315.
794. Journo C, Filipe J, About F, et al. NRP/Optineurin Cooperates with TAX1BP1 to potentiate the activation of NF-kappaB by human T-lymphotropic virus type 1 tax protein. PLoS Pathog 2009;5:e1000521.
795. Nagabhushana A, Bansal M, Swarup G. Optineurin is required for CYLD-dependent inhibition of TNFalpha-induced NF-kappaB activation. PLoS One 2011;6:e17477.
796. Li S, Wang L, Berman M, Kong YY, Dorf ME. Mapping a dynamic innate immunity protein interaction network regulating type I interferon production. Immunity 2011;35:426-440.
797. Wild P, Farhan H, McEwan DG, et al. Phosphorylation of the autophagy receptor optineurin restricts Salmonella growth. Science 2011;333:228-233.
798. Hattula K, Peranen J. FIP-2, a coiled-coil protein, links Huntingtin to Rab8 and modulates cellular morphogenesis. Curr Biol 2000;10:1603-1606.
799. Tumbarello DA, Manna PT, Allen M, et al. The Autophagy Receptor TAX1BP1 and the Molecular Motor Myosin VI Are Required for Clearance of Salmonella Typhimurium by Autophagy. PLoS Pathog 2015;11:e1005174.
800. Sowa ME, Bennett EJ, Gygi SP, Harper JW. Defining the human deubiquitinating enzyme interaction landscape. Cell 2009;138:389-403.
801. Kelly EE, Horgan CP, Adams C, et al. Class I Rab11-family interacting proteins are binding targets for the Rab14 GTPase. Biol Cell 2009;102:51-62.
802. Korac J, Schaeffer V, Kovacevic I, et al. Ubiquitin-independent function of optineurin in autophagic clearance of protein aggregates. J Cell Sci 2013;126:580-592.
803. Klingseisen L, Ehrenschwender M, Heigl U, et al. E3-14.7K is recruited to TNF-receptor 1 and blocks TNF cytolysis independent from interaction with optineurin. PLoS One 2012;7:e38348.
804. Mitsugi H, Niki T, Takahashi-Niki K, et al. Identification of the recognition sequence and target proteins for DJ-1 protease. FEBS Lett 2013;587:2493-2499.
805. Deeg S, Gralle M, Sroka K, Bahr M, Wouters FS, Kermer P. BAG1 restores formation of functional DJ-1 L166P dimers and DJ-1 chaperone activity. J Cell Biol 2010;188:505-513.
806. Ren H, Fu K, Wang D, Mu C, Wang G. Oxidized DJ-1 interacts with the mitochondrial protein BCL-XL. J Biol Chem 2011;286:35308-35317.
807. Fu K, Ren H, Wang Y, Fei E, Wang H, Wang G. DJ-1 inhibits TRAIL-induced apoptosis by blocking pro-caspase-8 recruitment to FADD. Oncogene 2012;31:1311-1322.
808. Opsahl JA, Hjornevik LV, Bull VH, et al. Increased interaction between DJ-1 and the Mi-2/ nucleosome remodelling and deacetylase complex during cellular stress. Proteomics 2010;10:1494-1504.
809. Malty RH, Aoki H, Kumar A, et al. A Map of Human Mitochondrial Protein Interactions Linked to Neurodegeneration Reveals New Mechanisms of Redox Homeostasis and NF-kappaB Signaling. Cell Syst 2017;5:564-577 e512.
810. Inberg A, Linial M. Protection of pancreatic beta-cells from various stress conditions is mediated by DJ-1. J Biol Chem 2010;285:25686-25698.

811. McNally RS, Davis BK, Clements CM, Accavitti-Loper MA, Mak TW, Ting JP. DJ-1 enhances cell survival through the binding of Cezanne, a negative regulator of NF-kappaB. J Biol Chem 2011;286:4098-4106.
812. Varaljai R, Islam AB, Beshiri ML, Rehman J, Lopez-Bigas N, Benevolenskaya EV. Increased mitochondrial function downstream from KDM5A histone demethylase rescues differentiation in pRB-deficient cells. Genes Dev 2015;29:1817-1834.
813. Waak J, Weber SS, Gorner K, et al. Oxidizable residues mediating protein stability and cytoprotective interaction of DJ-1 with apoptosis signal-regulating kinase 1. J Biol Chem 2009;284:14245-14257.
814. Moore DJ, Zhang L, Troncoso J, et al. Association of DJ-1 and parkin mediated by pathogenic DJ-1 mutations and oxidative stress. Hum Mol Genet 2005;14:71-84.
815. Takahashi K, Taira T, Niki T, Seino C, Iguchi-Ariga SM, Ariga H. DJ-1 positively regulates the androgen receptor by impairing the binding of PIASx alpha to the receptor. J Biol Chem 2001;276:37556-37563.
816. Kim SJ, Park YJ, Hwang IY, Youdim MB, Park KS, Oh YJ. Nuclear translocation of DJ-1 during oxidative stress-induced neuronal cell death. Free Radic Biol Med 2012;53:936-950.
817. Sablina AA, Chen W, Arroyo JD, et al. The tumor suppressor PP2A Abeta regulates the RalA GTPase. Cell 2007;129:969-982.
818. Kim YC, Kitaura H, Taira T, Iguchi-Ariga SM, Ariga H. Oxidation of DJ-1-dependent cell transformation through direct binding of DJ-1 to PTEN. Int J Oncol 2009;35:1331-1341.
819. Ma J, Wu R, Zhang Q, et al. DJ-1 interacts with RACK1 and protects neurons from oxidative-stress-induced apoptosis. Biochem J 2014;462:489-497.
820. Xu J, Zhong N, Wang H, et al. The Parkinson's disease-associated DJ-1 protein is a transcriptional co-activator that protects against neuronal apoptosis. Hum Mol Genet 2005;14:1231-1241.
821. Meulener MC, Graves CL, Sampathu DM, Armstrong-Gold CE, Bonini NM, Giasson BI. DJ-1 is present in a large molecular complex in human brain tissue and interacts with alpha-synuclein. J Neurochem 2005;93:1524-1532.
822. Xu XM, Lin H, Maple J, et al. The Arabidopsis DJ-1a protein confers stress protection through cytosolic SOD activation. J Cell Sci 2010;123:1644-1651.
823. Zhong N, Xu J. Synergistic activation of the human MnSOD promoter by DJ-1 and PGC-1alpha: regulation by SUMOylation and oxidation. Hum Mol Genet 2008;17:3357-3367.
824. Kim JH, Choi DJ, Jeong HK, et al. DJ-1 facilitates the interaction between STAT1 and its phosphatase, SHP-1, in brain microglia and astrocytes: A novel anti-inflammatory function of DJ-1. Neurobiol Dis 2013;60:1-10.
825. Junn E, Taniguchi H, Jeong BS, Zhao X, Ichijo H, Mouradian MM. Interaction of DJ-1 with Daxx inhibits apoptosis signal-regulating kinase 1 activity and cell death. Proc Natl Acad Sci U S A 2005;102:9691-9696.
826. Teachenor R, Beck K, Wright LY, Shen Z, Briggs SP, Murre C. Biochemical and phosphoproteomic analysis of the helix-loop-helix protein E47. Mol Cell Biol 2012;32:1671-1682.
827. Zucchelli S, Vilotti S, Calligaris R, et al. Aggresome-forming TTRAP mediates pro-apoptotic properties of Parkinson's disease-associated DJ-1 missense mutations. Cell Death Differ 2009;16:428-438.
828. Usami Y, Hatano T, Imai S, et al. DJ-1 associates with synaptic membranes. Neurobiol Dis 2011;43:651-662.
829. Parsanejad M, Zhang Y, Qu D, et al. Regulation of the VHL/HIF-1 pathway by DJ-1. J Neurosci 2014;34:8043-8050.
830. Wang X, Xiong LW, El Ayadi A, Boehning D, Putkey JA. The calmodulin regulator protein, PEP-19, sensitizes ATP-induced Ca2+ release. J Biol Chem 2013;288:2040-2048.

831. Woodman B, Butler R, Landles C, et al. The Hdh(Q150/Q150) knock-in mouse model of HD and the R6/2 exon 1 model develop comparable and widespread molecular phenotypes. Brain Res Bull 2007;72:83-97.
832. Caposio P, Gugliesi F, Zannetti C, et al. A novel role of the interferon-inducible protein IFI16 as inducer of proinflammatory molecules in endothelial cells. J Biol Chem 2007;282:33515-33529.
833. Boettner B, Govek EE, Cross J, Van Aelst L. The junctional multidomain protein AF-6 is a binding partner of the Rap1A GTPase and associates with the actin cytoskeletal regulator profilin. Proc Natl Acad Sci U S A 2000;97:9064-9069.
834. Lau E, Kluger H, Varsano T, et al. PKCepsilon promotes oncogenic functions of ATF2 in the nucleus while blocking its apoptotic function at mitochondria. Cell 2012;148:543-555.
835. Babusiak M, Man P, Petrak J, Vyoral D. Native proteomic analysis of protein complexes in murine intestinal brush border membranes. Proteomics 2007;7:121-129.
836. Witke W, Podtelejnikov AV, Di Nardo A, et al. In mouse brain profilin I and profilin II associate with regulators of the endocytic pathway and actin assembly. EMBO J 1998;17:967-976.
837. Lin YM, Ono K, Satoh S, et al. Identification of AF17 as a downstream gene of the beta-catenin/T-cell factor pathway and its involvement in colorectal carcinogenesis. Cancer Res 2001;61:6345-6349.
838. Gareus R, Di Nardo A, Rybin V, Witke W. Mouse profilin 2 regulates endocytosis and competes with SH3 ligand binding to dynamin 1. J Biol Chem 2006;281:2803-2811.
839. Durchdewald M, Guinea-Viniegra J, Haag D, et al. Podoplanin is a novel fos target gene in skin carcinogenesis. Cancer Res 2008;68:6877-6883.
840. Li M, Yasumura D, Ma AA, et al. Intravitreal administration of HA-1077, a ROCK inhibitor, improves retinal function in a mouse model of huntington disease. PLoS One 2013;8:e56026.
841. Anton IM, Lu W, Mayer BJ, Ramesh N, Geha RS. The Wiskott-Aldrich syndrome protein-interacting protein (WIP) binds to the adaptor protein Nck. J Biol Chem 1998;273:20992-20995.
842. Wang X, Kibschull M, Laue MM, Lichte B, Petrasch-Parwez E, Kilimann MW. Aczonin, a 550-kD putative scaffolding protein of presynaptic active zones, shares homology regions with Rim and Bassoon and binds profilin. J Cell Biol 1999;147:151-162.
843. Mahoney NM, Rozwarski DA, Fedorov E, Fedorov AA, Almo SC. Profilin binds proline-rich ligands in two distinct amide backbone orientations. Nat Struct Biol 1999;6:666-671.
844. Du Y, Zhou J, Fan J, Shen Z, Chen X. Streamline proteomic approach for characterizing protein-protein interaction network in a RAD52 protein complex. J Proteome Res 2009;8:2211-2217.
845. Giesemann T, Rathke-Hartlieb S, Rothkegel M, et al. A role for polyproline motifs in the spinal muscular atrophy protein SMN. Profilins bind to and colocalize with smn in nuclear gems. J Biol Chem 1999;274:37908-37914.
846. Choi YN, Lee SK, Seo TW, Lee JS, Yoo SJ. C-Terminus of Hsc70-interacting protein regulates profilin1 and breast cancer cell migration. Biochem Biophys Res Commun 2014;446:1060-1066.
847. Yarmola EG, Parikh S, Bubb MR. Formation and implications of a ternary complex of profilin, thymosin beta 4, and actin. J Biol Chem 2001;276:45555-45563.
848. Burnum KE, Hirota Y, Baker ES, et al. Uterine deletion of Trp53 compromises antioxidant responses in the mouse decidua. Endocrinology 2012;153:4568-4579.
849. Wolting CD, Griffiths EK, Sarao R, Prevost BC, Wybenga-Groot LE, McGlade CJ. Biochemical and computational analysis of LNX1 interacting proteins. PLoS One 2011;6:e26248.
850. Ernkvist M, Luna Persson N, Audebert S, et al. The Amot/Patj/Syx signaling complex spatially controls RhoA GTPase activity in migrating endothelial cells. Blood 2009;113:244-253.
851. Liu M, Horowitz A. A PDZ-binding motif as a critical determinant of Rho guanine exchange factor function and cell phenotype. Mol Biol Cell 2006;17:1880-1887.
852. Sorenson RC, Bisgaier CL, Aviram M, Hsu C, Billecke S, La Du BN. Human serum Paraoxonase/Arylesterase's retained hydrophobic N-terminal leader sequence associates with HDLs by

binding phospholipids : apolipoprotein A-I stabilizes activity. Arterioscler Thromb Vasc Biol 1999;19:2214-2225.
853. Parra S, Marsillach J, Aragones G, et al. Methodological constraints in interpreting serum paraoxonase-1 activity measurements: an example from a study in HIV-infected patients. Lipids Health Dis 2010;9:32.
854. Kelso GJ, Stuart WD, Richter RJ, Furlong CE, Jordan-Starck TC, Harmony JA. Apolipoprotein J is associated with paraoxonase in human plasma. Biochemistry 1994;33:832-839.
855. Leung CL, Sun D, Liem RK. The intermediate filament protein peripherin is the specific interaction partner of mouse BPAG1-n (dystonin) in neurons. J Cell Biol 1999;144:435-446.
856. Kourtis N, Moubarak RS, Aranda-Orgilles B, et al. FBXW7 modulates cellular stress response and metastatic potential through HSF1 post-translational modification. Nat Cell Biol 2015;17:322-332.
857. Cluskey M, Kim YK. Use and perceived effectiveness of strategies for enhancing food and nutrient intakes among elderly persons in long-term care. J Am Diet Assoc 2001;101:111-114.
858. Dykes IM, Tempest L, Lee SI, Turner EE. Brn3a and Islet1 act epistatically to regulate the gene expression program of sensory differentiation. J Neurosci 2011;31:9789-9799.
859. Waaijers S, Koorman T, Kerver J, Boxem M. Identification of human protein interaction domains using an ORFeome-based yeast two-hybrid fragment library. J Proteome Res 2013;12:3181-3192.
860. Guo QM, Malek RL, Kim S, et al. Identification of c-myc responsive genes using rat cDNA microarray. Cancer Res 2000;60:5922-5928.
861. Finotto S, Krieglstein K, Schober A, et al. Analysis of mice carrying targeted mutations of the glucocorticoid receptor gene argues against an essential role of glucocorticoid signalling for generating adrenal chromaffin cells. Development 1999;126:2935-2944.
862. Robertson J, Doroudchi MM, Nguyen MD, et al. A neurotoxic peripherin splice variant in a mouse model of ALS. J Cell Biol 2003;160:939-949.
863. Bhoumik A, Fichtman B, Derossi C, et al. Suppressor role of activating transcription factor 2 (ATF2) in skin cancer. Proc Natl Acad Sci U S A 2008;105:1674-1679.
864. Mitsuda T, Hayakawa Y, Itoh M, Ohta K, Nakagawa T. ATF4 regulates gamma-secretase activity during amino acid imbalance. Biochem Biophys Res Commun 2007;352:722-727.
865. Jeon AH, Bohm C, Chen F, et al. Interactome analyses of mature gamma-secretase complexes reveal distinct molecular environments of presenilin (PS) paralogs and preferential binding of signal peptide peptidase to PS2. J Biol Chem 2013;288:15352-15366.
866. Alberici A, Moratto D, Benussi L, et al. Presenilin 1 protein directly interacts with Bcl-2. J Biol Chem 1999;274:30764-30769.
867. Stemmer N, Strekalova E, Djogo N, et al. Generation of amyloid-beta is reduced by the interaction of calreticulin with amyloid precursor protein, presenilin and nicastrin. PLoS One 2013;8:e61299.
868. Nishiyama K, Trapp BD, Ikezu T, et al. Caveolin-3 upregulation activates beta-secretase-mediated cleavage of the amyloid precursor protein in Alzheimer's disease. J Neurosci 1999;19:6538-6548.
869. Schwarzman AL, Singh N, Tsiper M, et al. Endogenous presenilin 1 redistributes to the surface of lamellipodia upon adhesion of Jurkat cells to a collagen matrix. Proc Natl Acad Sci U S A 1999;96:7932-7937.
870. Serban G, Kouchi Z, Baki L, et al. Cadherins mediate both the association between PS1 and beta-catenin and the effects of PS1 on beta-catenin stability. J Biol Chem 2005;280:36007-36012.
871. Maesako M, Uemura K, Kubota M, et al. Insulin regulates Presenilin 1 localization via PI3K/Akt signaling. Neurosci Lett 2010;483:157-161.
872. Gupta S, Singh R, Datta P, et al. The C-terminal tail of presenilin regulates Omi/HtrA2 protease activity. J Biol Chem 2004;279:45844-45854.

873. Prager K, Wang-Eckhardt L, Fluhrer R, et al. A structural switch of presenilin 1 by glycogen synthase kinase 3beta-mediated phosphorylation regulates the interaction with beta-catenin and its nuclear signaling. J Biol Chem 2007;282:14083-14093.
874. Shrestha H, Ryu T, Seo YW, et al. Hakai, an E3-ligase for E-cadherin, stabilizes delta-catenin through Src kinase. Cell Signal 2017;31:135-145.
875. Biederer T, Cao X, Sudhof TC, Liu X. Regulation of APP-dependent transcription complexes by Mint/X11s: differential functions of Mint isoforms. J Neurosci 2002;22:7340-7351.
876. Georgakopoulos A, Litterst C, Ghersi E, et al. Metalloproteinase/Presenilin1 processing of ephrinB regulates EphB-induced Src phosphorylation and signaling. EMBO J 2006;25:1242-1252.
877. Ni CY, Murphy MP, Golde TE, Carpenter G. gamma -Secretase cleavage and nuclear localization of ErbB-4 receptor tyrosine kinase. Science 2001;294:2179-2181.
878. Teranishi Y, Hur JY, Gu GJ, et al. Erlin-2 is associated with active gamma-secretase in brain and affects amyloid beta-peptide production. Biochem Biophys Res Commun 2012;424:476-481.
879. Pastorcic M, Das HK. Regulation of transcription of the human presenilin-1 gene by ets transcription factors and the p53 protooncogene. J Biol Chem 2000;275:34938-34945.
880. Li J, Pauley AM, Myers RL, et al. SEL-10 interacts with presenilin 1, facilitates its ubiquitination, and alters A-beta peptide production. J Neurochem 2002;82:1540-1548.
881. Wu J, Petralia RS, Kurushima H, et al. Arc/Arg3.1 regulates an endosomal pathway essential for activity-dependent beta-amyloid generation. Cell 2011;147:615-628.
882. Nizzari M, Venezia V, Repetto E, et al. Amyloid precursor protein and Presenilin1 interact with the adaptor GRB2 and modulate ERK 1,2 signaling. J Biol Chem 2007;282:13833-13844.
883. Meyer EL, Strutz N, Gahring LC, Rogers SW. Glutamate receptor subunit 3 is modified by site-specific limited proteolysis including cleavage by gamma-secretase. J Biol Chem 2003;278:23786-23796.
884. Saura CA, Choi SY, Beglopoulos V, et al. Loss of presenilin function causes impairments of memory and synaptic plasticity followed by age-dependent neurodegeneration. Neuron 2004;42:23-36.
885. Nakamura K, Iwamoto R, Mekada E. Membrane-anchored heparin-binding EGF-like growth factor (HB-EGF) and diphtheria toxin receptor-associated protein (DRAP27)/CD9 form a complex with integrin alpha 3 beta 1 at cell-cell contact sites. J Cell Biol 1995;129:1691-1705.
886. Sai X, Kokame K, Shiraishi H, et al. The ubiquitin-like domain of Herp is involved in Herp degradation, but not necessary for its enhancement of amyloid beta-protein generation. FEBS Lett 2003;553:151-156.
887. Gray CW, Ward RV, Karran E, et al. Characterization of human HtrA2, a novel serine protease involved in the mammalian cellular stress response. Eur J Biochem 2000;267:5699-5710.
888. Elzinga BM, Twomey C, Powell JC, Harte F, McCarthy JV. Interleukin-1 receptor type 1 is a substrate for gamma-secretase-dependent regulated intramembrane proteolysis. J Biol Chem 2009;284:1394-1409.
889. Hemming ML, Elias JE, Gygi SP, Selkoe DJ. Proteomic profiling of gamma-secretase substrates and mapping of substrate requirements. PLoS Biol 2008;6:e257.
890. Raurell I, Codina M, Casagolda D, et al. Gamma-secretase-dependent and -independent effects of presenilin1 on beta-catenin.Tcf-4 transcriptional activity. PLoS One 2008;3:e4080.
891. Sehgal N, Gupta A, Valli RK, et al. Withania somnifera reverses Alzheimer's disease pathology by enhancing low-density lipoprotein receptor-related protein in liver. Proc Natl Acad Sci U S A 2012;109:3510-3515.
892. Takashima A, Murayama M, Murayama O, et al. Presenilin 1 associates with glycogen synthase kinase-3beta and its substrate tau. Proc Natl Acad Sci U S A 1998;95:9637-9641.
893. Kim SH, Ikeuchi T, Yu C, Sisodia SS. Regulated hyperaccumulation of presenilin-1 and the "gamma-secretase" complex. Evidence for differential intramembranous processing of transmembrane subatrates. J Biol Chem 2003;278:33992-34002.

894. Ray WJ, Yao M, Nowotny P, et al. Evidence for a physical interaction between presenilin and Notch. Proc Natl Acad Sci U S A 1999;96:3263-3268.
895. Baki L, Shioi J, Wen P, et al. PS1 activates PI3K thus inhibiting GSK-3 activity and tau overphosphorylation: effects of FAD mutations. EMBO J 2004;23:2586-2596.
896. Walter J, Grunberg J, Schindzielorz A, Haass C. Proteolytic fragments of the Alzheimer's disease associated presenilins-1 and -2 are phosphorylated in vivo by distinct cellular mechanisms. Biochemistry 1998;37:5961-5967.
897. Van Gassen G, De Jonghe C, Pype S, et al. Alzheimer's disease associated presenilin 1 interacts with HC5 and ZETA, subunits of the catalytic 20S proteasome. Neurobiol Dis 1999;6:376-391.
898. Smith SK, Anderson HA, Yu G, et al. Identification of syntaxin 1A as a novel binding protein for presenilin-1. Brain Res Mol Brain Res 2000;78:100-107.
899. Chen F, Hasegawa H, Schmitt-Ulms G, et al. TMP21 is a presenilin complex component that modulates gamma-secretase but not epsilon-secretase activity. Nature 2006;440:1208-1212.
900. Thomas AV, Herl L, Spoelgen R, et al. Interaction between presenilin 1 and ubiquilin 1 as detected by fluorescence lifetime imaging microscopy and a high-throughput fluorescent plate reader. J Biol Chem 2006;281:26400-26407.
901. Feng R, Wang H, Wang J, Shrom D, Zeng X, Tsien JZ. Forebrain degeneration and ventricle enlargement caused by double knockout of Alzheimer's presenilin-1 and presenilin-2. Proc Natl Acad Sci U S A 2004;101:8162-8167.
902. Fukuhara A, Irie K, Nakanishi H, et al. Involvement of nectin in the localization of junctional adhesion molecule at tight junctions. Oncogene 2002;21:7642-7655.
903. Kikyo M, Matozaki T, Kodama A, Kawabe H, Nakanishi H, Takai Y. Cell-cell adhesion-mediated tyrosine phosphorylation of nectin-2delta, an immunoglobulin-like cell adhesion molecule at adherens junctions. Oncogene 2000;19:4022-4028.
904. Mueller S, Cao X, Welker R, Wimmer E. Interaction of the poliovirus receptor CD155 with the dynein light chain Tctex-1 and its implication for poliovirus pathogenesis. J Biol Chem 2002;277:7897-7904.
905. Solecki DJ, Gromeier M, Mueller S, Bernhardt G, Wimmer E. Expression of the human poliovirus receptor/CD155 gene is activated by sonic hedgehog. J Biol Chem 2002;277:25697-25702.
906. Minami Y, Ikeda W, Kajita M, et al. Necl-5/poliovirus receptor interacts in cis with integrin alphaVbeta3 and regulates its clustering and focal complex formation. J Biol Chem 2007;282:18481-18496.
907. Mizutani K, Kawano S, Minami A, Waseda M, Ikeda W, Takai Y. Interaction of nectin-like molecule 2 with integrin alpha6beta4 and inhibition of disassembly of integrin alpha6beta4 from hemidesmosomes. J Biol Chem 2011;286:36667-36676.
908. Harrison OJ, Vendome J, Brasch J, et al. Nectin ectodomain structures reveal a canonical adhesive interface. Nat Struct Mol Biol 2012;19:906-915.
909. Duszka K, Bogner-Strauss JG, Hackl H, et al. Nr4a1 is required for fasting-induced down-regulation of Ppargamma2 in white adipose tissue. Mol Endocrinol 2013;27:135-149.
910. Bomberger JM, Parameswaran N, Hall CS, Aiyar N, Spielman WS. Novel function for receptor activity-modifying proteins (RAMPs) in post-endocytic receptor trafficking. J Biol Chem 2005;280:9297-9307.
911. Bock J, Mochmann LH, Schlee C, et al. ERG transcriptional networks in primary acute leukemia cells implicate a role for ERG in deregulated kinase signaling. PLoS One 2013;8:e52872.
912. Kubben N, Adriaens M, Meuleman W, Voncken JW, van Steensel B, Misteli T. Mapping of lamin A- and progerin-interacting genome regions. Chromosoma 2012;121:447-464.
913. Oster SK, Ho CS, Soucie EL, Penn LZ. The myc oncogene: MarvelouslY Complex. Adv Cancer Res 2002;84:81-154.

914. Dekker RJ, Boon RA, Rondaij MG, et al. KLF2 provokes a gene expression pattern that establishes functional quiescent differentiation of the endothelium. Blood 2006;107:4354-4363.
915. Iyer S, Holloway DE, Kumar K, Shapiro R, Acharya KR. Molecular recognition of human eosinophil-derived neurotoxin (RNase 2) by placental ribonuclease inhibitor. J Mol Biol 2005;347:637-655.
916. Huang Y, Niwa J, Sobue G, Breitwieser GE. Calcium-sensing receptor ubiquitination and degradation mediated by the E3 ubiquitin ligase dorfin. J Biol Chem 2006;281:11610-11617.
917. Paces-Fessy M, Boucher D, Petit E, Paute-Briand S, Blanchet-Tournier MF. The negative regulator of Gli, Suppressor of fused (Sufu), interacts with SAP18, Galectin3 and other nuclear proteins. Biochem J 2004;378:353-362.
918. Niwa J, Yamada S, Ishigaki S, et al. Disulfide bond mediates aggregation, toxicity, and ubiquitylation of familial amyotrophic lateral sclerosis-linked mutant SOD1. J Biol Chem 2007;282:28087-28095.
919. Gunther M, Laithier M, Brison O. A set of proteins interacting with transcription factor Sp1 identified in a two-hybrid screening. Mol Cell Biochem 2000;210:131-142.
920. Molday LL, Wu WW, Molday RS. Retinoschisin (RS1), the protein encoded by the X-linked retinoschisis gene, is anchored to the surface of retinal photoreceptor and bipolar cells through its interactions with a Na/K ATPase-SARM1 complex. J Biol Chem 2007;282:32792-32801.
921. Kim Y, Zhou P, Qian L, et al. MyD88-5 links mitochondria, microtubules, and JNK3 in neurons and regulates neuronal survival. J Exp Med 2007;204:2063-2074.
922. Ulrichts P, Peelman F, Beyaert R, Tavernier J. MAPPIT analysis of TLR adaptor complexes. FEBS Lett 2007;581:629-636.
923. Murata H, Sakaguchi M, Kataoka K, Huh NH. SARM1 and TRAF6 bind to and stabilize PINK1 on depolarized mitochondria. Mol Biol Cell 2013;24:2772-2784.
924. Sethurathinam S, Singh LP, Panneerselvam P, Byrne B, Ding JL. UXT plays dual opposing roles on SARM-induced apoptosis. FEBS Lett 2013;587:3296-3302.
925. Matsumoto M, Fujikawa A, Suzuki R, et al. SAP97 promotes the stability of Nax channels at the plasma membrane. FEBS Lett 2012;586:3805-3812.
926. de Coupade C, Solito E, Levine JD. Dexamethasone enhances interaction of endogenous annexin 1 with L-selectin and triggers shedding of L-selectin in the monocytic cell line U-937. Br J Pharmacol 2003;140:133-145.
927. Matala E, Alexander SR, Kishimoto TK, Walcheck B. The cytoplasmic domain of L-selectin participates in regulating L-selectin endoproteolysis. J Immunol 2001;167:1617-1623.
928. Kawashima H, Watanabe N, Hirose M, et al. Collagen XVIII, a basement membrane heparan sulfate proteoglycan, interacts with L-selectin and monocyte chemoattractant protein-1. J Biol Chem 2003;278:13069-13076.
929. Ivetic A, Deka J, Ridley A, Ager A. The cytoplasmic tail of L-selectin interacts with members of the Ezrin-Radixin-Moesin (ERM) family of proteins: cell activation-dependent binding of Moesin but not Ezrin. J Biol Chem 2002;277:2321-2329.
930. Lou Y, Lu X, Dang X. FOXO1 Up-Regulates Human L-selectin Expression Through Binding to a Consensus FOXO1 Motif. Gene Regul Syst Bio 2012;6:139-149.
931. Chae WJ, Henegariu O, Lee SK, Bothwell AL. The mutant leucine-zipper domain impairs both dimerization and suppressive function of Foxp3 in T cells. Proc Natl Acad Sci U S A 2006;103:9631-9636.
932. Brenner B, Gulbins E, Schlottmann K, et al. L-selectin activates the Ras pathway via the tyrosine kinase p56lck. Proc Natl Acad Sci U S A 1996;93:15376-15381.
933. Dang X, Raffler NA, Ley K. Transcriptional regulation of mouse L-selectin. Biochim Biophys Acta 2009;1789:146-152.
934. Vu TT, Gatto D, Turner V, et al. Impaired B cell development in the absence of Kruppel-like factor 3. J Immunol 2011;187:5032-5042.

935. Porter CM, Clipstone NA. Sustained NFAT signaling promotes a Th1-like pattern of gene expression in primary murine CD4+ T cells. J Immunol 2002;168:4936-4945.
936. Kilian K, Dernedde J, Mueller EC, Bahr I, Tauber R. The interaction of protein kinase C isozymes alpha, iota, and theta with the cytoplasmic domain of L-selectin is modulated by phosphorylation of the receptor. J Biol Chem 2004;279:34472-34480.
937. Ahlfors H, Limaye A, Elo LL, et al. SATB1 dictates expression of multiple genes including IL-5 involved in human T helper cell differentiation. Blood 2010;116:1443-1453.
938. Stadtmann A, Germena G, Block H, et al. The PSGL-1-L-selectin signaling complex regulates neutrophil adhesion under flow. J Exp Med 2013;210:2171-2180.
939. Nakayama M, Kikuno R, Ohara O. Protein-protein interactions between large proteins: two-hybrid screening using a functionally classified library composed of long cDNAs. Genome Res 2002;12:1773-1784.
940. Wang G, Cui Y, Zhang G, Garen A, Song X. Regulation of proto-oncogene transcription, cell proliferation, and tumorigenesis in mice by PSF protein and a VL30 noncoding RNA. Proc Natl Acad Sci U S A 2009;106:16794-16798.
941. Kenzelmann Broz D, Spano Mello S, Bieging KT, et al. Global genomic profiling reveals an extensive p53-regulated autophagy program contributing to key p53 responses. Genes Dev 2013;27:1016-1031.
942. Gocke CB, Yu H, Kang J. Systematic identification and analysis of mammalian small ubiquitin-like modifier substrates. J Biol Chem 2005;280:5004-5012.
943. Hatchi E, Skourti-Stathaki K, Ventz S, et al. BRCA1 recruitment to transcriptional pause sites is required for R-loop-driven DNA damage repair. Mol Cell 2015;57:636-647.
944. Shen X, Kim W, Fujiwara Y, et al. Jumonji modulates polycomb activity and self-renewal versus differentiation of stem cells. Cell 2009;139:1303-1314.
945. Paramanik V, Thakur MK. Estrogen receptor beta and its domains interact with casein kinase 2, phosphokinase C, and N-myristoylation sites of mitochondrial and nuclear proteins in mouse brain. J Biol Chem 2012;287:22305-22316.
946. Bennett CL, Chen Y, Vignali M, et al. Protein interaction analysis of senataxin and the ALS4 L389S mutant yields insights into senataxin post-translational modification and uncovers mutant-specific binding with a brain cytoplasmic RNA-encoded peptide. PLoS One 2013;8:e78837.
947. Chen PB, Hung JH, Hickman TL, et al. Hdac6 regulates Tip60-p400 function in stem cells. Elife 2013;2:e01557.
948. Zhao DY, Gish G, Braunschweig U, et al. SMN and symmetric arginine dimethylation of RNA polymerase II C-terminal domain control termination. Nature 2016;529:48-53.
949. Verrastro I, Tveen-Jensen K, Woscholski R, Spickett CM, Pitt AR. Reversible oxidation of phosphatase and tensin homolog (PTEN) alters its interactions with signaling and regulatory proteins. Free Radic Biol Med 2016;90:24-34.
950. Hecker CM, Rabiller M, Haglund K, Bayer P, Dikic I. Specification of SUMO1- and SUMO2-interacting motifs. J Biol Chem 2006;281:16117-16127.
951. Hayashi T, Su TP. Regulating ankyrin dynamics: Roles of sigma-1 receptors. Proc Natl Acad Sci U S A 2001;98:491-496.
952. Genda EN, Jackson JG, Sheldon AL, et al. Co-compartmentalization of the astroglial glutamate transporter, GLT-1, with glycolytic enzymes and mitochondria. J Neurosci 2011;31:18275-18288.
953. Lee SG, Kim K, Kegelman TP, et al. Oncogene AEG-1 promotes glioma-induced neurodegeneration by increasing glutamate excitotoxicity. Cancer Res 2011;71:6514-6523.
954. Cadoret A, Ovejero C, Terris B, et al. New targets of beta-catenin signaling in the liver are involved in the glutamine metabolism. Oncogene 2002;21:8293-8301.
955. Underhill SM, Wheeler DS, Amara SG. Differential regulation of two isoforms of the glial glutamate transporter EAAT2 by DLG1 and CaMKII. J Neurosci 2015;35:5260-5270.

956. Karki P, Webb A, Smith K, et al. Yin Yang 1 is a repressor of glutamate transporter EAAT2, and it mediates manganese-induced decrease of EAAT2 expression in astrocytes. Mol Cell Biol 2014;34:1280-1289.
957. Rinn JL, Wang JK, Allen N, et al. A dermal HOX transcriptional program regulates site-specific epidermal fate. Genes Dev 2008;22:303-307.
958. Sitcheran R, Gupta P, Fisher PB, Baldwin AS. Positive and negative regulation of EAAT2 by NF-kappaB: a role for N-myc in TNFalpha-controlled repression. EMBO J 2005;24:510-520.
959. Gibb SL, Boston-Howes W, Lavina ZS, et al. A caspase-3-cleaved fragment of the glial glutamate transporter EAAT2 is sumoylated and targeted to promyelocytic leukemia nuclear bodies in mutant SOD1-linked amyotrophic lateral sclerosis. J Biol Chem 2007;282:32480-32490.
960. Kang HS, Angers M, Beak JY, et al. Gene expression profiling reveals a regulatory role for ROR alpha and ROR gamma in phase I and phase II metabolism. Physiol Genomics 2007;31:281-294.
961. Yernool D, Boudker O, Jin Y, Gouaux E. Structure of a glutamate transporter homologue from Pyrococcus horikoshii. Nature 2004;431:811-818.
962. Foerster S, Kacprowski T, Dhople VM, et al. Characterization of the EGFR interactome reveals associated protein complex networks and intracellular receptor dynamics. Proteomics 2013;13:3131-3144.
963. Iwahashi H, Eguchi Y, Yasuhara N, Hanafusa T, Matsuzawa Y, Tsujimoto Y. Synergistic anti-apoptotic activity between Bcl-2 and SMN implicated in spinal muscular atrophy. Nature 1997;390:413-417.
964. Hebert MD, Shpargel KB, Ospina JK, Tucker KE, Matera AG. Coilin methylation regulates nuclear body formation. Dev Cell 2002;3:329-337.
965. Custer SK, Todd AG, Singh NN, Androphy EJ. Dilysine motifs in exon 2b of SMN protein mediate binding to the COPI vesicle protein alpha-COP and neurite outgrowth in a cell culture model of spinal muscular atrophy. Hum Mol Genet 2013;22:4043-4052.
966. Pellizzoni L, Charroux B, Rappsilber J, Mann M, Dreyfuss G. A functional interaction between the survival motor neuron complex and RNA polymerase II. J Cell Biol 2001;152:75-85.
967. Joshi P, Greco TM, Guise AJ, et al. The functional interactome landscape of the human histone deacetylase family. Mol Syst Biol 2013;9:672.
968. Claus P, Doring F, Gringel S, et al. Differential intranuclear localization of fibroblast growth factor-2 isoforms and specific interaction with the survival of motoneuron protein. J Biol Chem 2003;278:479-485.
969. Pellizzoni L, Baccon J, Charroux B, Dreyfuss G. The survival of motor neurons (SMN) protein interacts with the snoRNP proteins fibrillarin and GAR1. Curr Biol 2001;11:1079-1088.
970. Ash MR, Faelber K, Kosslick D, et al. Conserved beta-hairpin recognition by the GYF domains of Smy2 and GIGYF2 in mRNA surveillance and vesicular transport complexes. Structure 2010;18:944-954.
971. Zou J, Barahmand-pour F, Blackburn ML, Matsui Y, Chansky HA, Yang L. Survival motor neuron (SMN) protein interacts with transcription corepressor mSin3A. J Biol Chem 2004;279:14922-14928.
972. Yang Y, Lu Y, Espejo A, et al. TDRD3 is an effector molecule for arginine-methylated histone marks. Mol Cell 2010;40:1016-1023.
973. Chen HH, Chang JG, Lu RM, Peng TY, Tarn WY. The RNA binding protein hnRNP Q modulates the utilization of exon 7 in the survival motor neuron 2 (SMN2) gene. Mol Cell Biol 2008;28:6929-6938.
974. Pedrotti S, Bielli P, Paronetto MP, et al. The splicing regulator Sam68 binds to a novel exonic splicing silencer and functions in SMN2 alternative splicing in spinal muscular atrophy. EMBO J 2010;29:1235-1247.
975. Narayanan U, Achsel T, Luhrmann R, Matera AG. Coupled in vitro import of U snRNPs and SMN, the spinal muscular atrophy protein. Mol Cell 2004;16:223-234.

976. Park JW, Voss PG, Grabski S, Wang JL, Patterson RJ. Association of galectin-1 and galectin-3 with Gemin4 in complexes containing the SMN protein. Nucleic Acids Res 2001;29:3595-3602.
977. Friesen WJ, Dreyfuss G. Specific sequences of the Sm and Sm-like (Lsm) proteins mediate their interaction with the spinal muscular atrophy disease gene product (SMN). J Biol Chem 2000;275:26370-26375.
978. Kwon JE, Kim EK, Choi EJ. Stabilization of the survival motor neuron protein by ASK1. FEBS Lett 2011;585:1287-1292.
979. Kwon DY, Dimitriadi M, Terzic B, et al. The E3 ubiquitin ligase mind bomb 1 ubiquitinates and promotes the degradation of survival of motor neuron protein. Mol Biol Cell 2013;24:1863-1871.
980. O'Connell BC, Cheung AF, Simkevich CP, et al. A large scale genetic analysis of c-Myc-regulated gene expression patterns. J Biol Chem 2003;278:12563-12573.
981. Carnegie GK, Sleeman JE, Morrice N, et al. Protein phosphatase 4 interacts with the Survival of Motor Neurons complex and enhances the temporal localisation of snRNPs. J Cell Sci 2003;116:1905-1913.
982. Wang J, Yuan Y, Zhou Y, et al. Protein interaction data set highlighted with human Ras-MAPK/PI3K signaling pathways. J Proteome Res 2008;7:3879-3889.
983. Hegele A, Kamburov A, Grossmann A, et al. Dynamic protein-protein interaction wiring of the human spliceosome. Mol Cell 2012;45:567-580.
984. Piazzon N, Schlotter F, Lefebvre S, et al. Implication of the SMN complex in the biogenesis and steady state level of the signal recognition particle. Nucleic Acids Res 2013;41:1255-1272.
985. Bachand F, Boisvert FM, Cote J, Richard S, Autexier C. The product of the survival of motor neuron (SMN) gene is a human telomerase-associated protein. Mol Biol Cell 2002;13:3192-3202.
986. Fong KW, Li Y, Wang W, et al. Whole-genome screening identifies proteins localized to distinct nuclear bodies. J Cell Biol 2013;203:149-164.
987. Hsu SH, Lai MC, Er TK, et al. Ubiquitin carboxyl-terminal hydrolase L1 (UCHL1) regulates the level of SMN expression through ubiquitination in primary spinal muscular atrophy fibroblasts. Clin Chim Acta 2010;411:1920-1928.
988. Yang X, Coulombe-Huntington J, Kang S, et al. Widespread Expansion of Protein Interaction Capabilities by Alternative Splicing. Cell 2016;164:805-817.
989. Buhler D, Raker V, Luhrmann R, Fischer U. Essential role for the tudor domain of SMN in spliceosomal U snRNP assembly: implications for spinal muscular atrophy. Hum Mol Genet 1999;8:2351-2357.
990. Han KJ, Foster DG, Zhang NY, et al. Ubiquitin-specific protease 9x deubiquitinates and stabilizes the spinal muscular atrophy protein-survival motor neuron. J Biol Chem 2012;287:43741-43752.
991. Hofer P, Boeszoermenyi A, Jaeger D, et al. Fatty Acid-binding Proteins Interact with Comparative Gene Identification-58 Linking Lipolysis with Lipid Ligand Shuttling. J Biol Chem 2015;290:18438-18453.
992. Kittanakom S, Barrios-Rodiles M, Petschnigg J, et al. CHIP-MYTH: a novel interactive proteomics method for the assessment of agonist-dependent interactions of the human beta(2)-adrenergic receptor. Biochem Biophys Res Commun 2014;445:746-756.
993. Liu C, Qu L, Lian S, et al. Unconventional secretion of synuclein-gamma promotes tumor cell invasion. FEBS J 2014;281:5159-5171.
994. Pan ZZ, Bruening W, Giasson BI, Lee VM, Godwin AK. Gamma-synuclein promotes cancer cell survival and inhibits stress- and chemotherapy drug-induced apoptosis by modulating MAPK pathways. J Biol Chem 2002;277:35050-35060.
995. Quina LA, Wang S, Ng L, Turner EE. Brn3a and Nurr1 mediate a gene regulatory pathway for habenula development. J Neurosci 2009;29:14309-14322.
996. Campeau PM, Astapova O, Martins R, et al. Clinical and molecular characterization of a severe form of partial lipodystrophy expanding the phenotype of PPARgamma deficiency. J Lipid Res 2012;53:1968-1978.

997. Ying Z, Wang H, Fan H, et al. Gp78, an ER associated E3, promotes SOD1 and ataxin-3 degradation. Hum Mol Genet 2009;18:4268-4281.
998. Pasinelli P, Belford ME, Lennon N, et al. Amyotrophic lateral sclerosis-associated SOD1 mutant proteins bind and aggregate with Bcl-2 in spinal cord mitochondria. Neuron 2004;43:19-30.
999. Karve TM, Rosen EM. B-cell translocation gene 2 (BTG2) stimulates cellular antioxidant defenses through the antioxidant transcription factor NFE2L2 in human mammary epithelial cells. J Biol Chem 2012;287:31503-31514.
1000. Kim YH, Yoo HY, Chang MS, Jung G, Rho HM. C/EBP alpha is a major activator for the transcription of rat Cu/Zn superoxide dismutase gene in liver cell. FEBS Lett 1997;401:267-270.
1001. Urushitani M, Sik A, Sakurai T, Nukina N, Takahashi R, Julien JP. Chromogranin-mediated secretion of mutant superoxide dismutase proteins linked to amyotrophic lateral sclerosis. Nat Neurosci 2006;9:108-118.
1002. Vonk WI, Kakkar V, Bartuzi P, et al. The Copper Metabolism MURR1 domain protein 1 (COMMD1) modulates the aggregation of misfolded protein species in a client-specific manner. PLoS One 2014;9:e92408.
1003. Yerbury JJ, Gower D, Vanags L, Roberts K, Lee JA, Ecroyd H. The small heat shock proteins alphaB-crystallin and Hsp27 suppress SOD1 aggregation in vitro. Cell Stress Chaperones 2013;18:251-257.
1004. Homma K, Fujisawa T, Tsuburaya N, et al. SOD1 as a molecular switch for initiating the homeostatic ER stress response under zinc deficiency. Mol Cell 2013;52:75-86.
1005. Zhang F, Strom AL, Fukada K, Lee S, Hayward LJ, Zhu H. Interaction between familial amyotrophic lateral sclerosis (ALS)-linked SOD1 mutants and the dynein complex. J Biol Chem 2007;282:16691-16699.
1006. Gamerdinger M, Kaya AM, Wolfrum U, Clement AM, Behl C. BAG3 mediates chaperone-based aggresome-targeting and selective autophagy of misfolded proteins. EMBO Rep 2011;12:149-156.
1007. Minc E, de Coppet P, Masson P, et al. The human copper-zinc superoxide dismutase gene (SOD1) proximal promoter is regulated by Sp1, Egr-1, and WT1 via non-canonical binding sites. J Biol Chem 1999;274:503-509.
1008. Scortegagna M, Ding K, Oktay Y, et al. Multiple organ pathology, metabolic abnormalities and impaired homeostasis of reactive oxygen species in Epas1-/- mice. Nat Genet 2003;35:331-340.
1009. Miyashiro KY, Beckel-Mitchener A, Purk TP, et al. RNA cargoes associating with FMRP reveal deficits in cellular functioning in Fmr1 null mice. Neuron 2003;37:417-431.
1010. Li J, Du W, Maynard S, Andreassen PR, Pang Q. Oxidative stress-specific interaction between FANCD2 and FOXO3a. Blood 2010;115:1545-1548.
1011. Shinder GA, Lacourse MC, Minotti S, Durham HD. Mutant Cu/Zn-superoxide dismutase proteins have altered solubility and interact with heat shock/stress proteins in models of amyotrophic lateral sclerosis. J Biol Chem 2001;276:12791-12796.
1012. Wang J, Farr GW, Zeiss CJ, et al. Progressive aggregation despite chaperone associations of a mutant SOD1-YFP in transgenic mice that develop ALS. Proc Natl Acad Sci U S A 2009;106:1392-1397.
1013. Choi JS, Cho S, Park SG, Park BC, Lee DH. Co-chaperone CHIP associates with mutant Cu/Zn-superoxide dismutase proteins linked to familial amyotrophic lateral sclerosis and promotes their degradation by proteasomes. Biochem Biophys Res Commun 2004;321:574-583.
1014. Zetterstrom P, Graffmo KS, Andersen PM, Brannstrom T, Marklund SL. Proteins that bind to misfolded mutant superoxide dismutase-1 in spinal cords from transgenic amyotrophic lateral sclerosis (ALS) model mice. J Biol Chem 2011;286:20130-20136.
1015. Reijonen S, Kukkonen JP, Hyrskyluoto A, et al. Downregulation of NF-kappaB signaling by mutant huntingtin proteins induces oxidative stress and cell death. Cell Mol Life Sci 2010;67:1929-1941.
1016. Vargas MR, Pehar M, Cassina P, et al. Fibroblast growth factor-1 induces heme oxygenase-1 via nuclear factor erythroid 2-related factor 2 (Nrf2) in spinal cord astrocytes: consequences for motor neuron survival. J Biol Chem 2005;280:25571-25579.

1017. Benedusi V, Martorana F, Brambilla L, Maggi A, Rossi D. The peroxisome proliferator-activated receptor gamma (PPARgamma) controls natural protective mechanisms against lipid peroxidation in amyotrophic lateral sclerosis. J Biol Chem 2012;287:35899-35911.
1018. Volakakis N, Kadkhodaei B, Joodmardi E, et al. NR4A orphan nuclear receptors as mediators of CREB-dependent neuroprotection. Proc Natl Acad Sci U S A 2010;107:12317-12322.
1019. Harraz MM, Marden JJ, Zhou W, et al. SOD1 mutations disrupt redox-sensitive Rac regulation of NADPH oxidase in a familial ALS model. J Clin Invest 2008;118:659-670.
1020. Ishigaki S, Niwa J, Yamada S, et al. Dorfin-CHIP chimeric proteins potently ubiquitylate and degrade familial ALS-related mutant SOD1 proteins and reduce their cellular toxicity. Neurobiol Dis 2007;25:331-341.
1021. Guegan C, Przedborski S. Programmed cell death in amyotrophic lateral sclerosis. J Clin Invest 2003;111:153-161.
1022. Mishra A, Maheshwari M, Chhangani D, et al. E6-AP association promotes SOD1 aggresomes degradation and suppresses toxicity. Neurobiol Aging 2013;34:1310 e1311-1323.
1023. Rico de Souza A, Zago M, Pollock SJ, Sime PJ, Phipps RP, Baglole CJ. Genetic ablation of the aryl hydrocarbon receptor causes cigarette smoke-induced mitochondrial dysfunction and apoptosis. J Biol Chem 2011;286:43214-43228.
1024. Gutierrez-Uzquiza A, Arechederra M, Bragado P, Aguirre-Ghiso JA, Porras A. p38alpha mediates cell survival in response to oxidative stress via induction of antioxidant genes: effect on the p70S6K pathway. J Biol Chem 2012;287:2632-2642.
1025. Araujo J, Breuer P, Dieringer S, et al. FOXO4-dependent upregulation of superoxide dismutase-2 in response to oxidative stress is impaired in spinocerebellar ataxia type 3. Hum Mol Genet 2011;20:2928-2941.
1026. Tanaka H, Matsumura I, Ezoe S, et al. E2F1 and c-Myc potentiate apoptosis through inhibition of NF-kappaB activity that facilitates MnSOD-mediated ROS elimination. Mol Cell 2002;9:1017-1029.
1027. Maehara K, Oh-Hashi K, Isobe KI. Early growth-responsive-1-dependent manganese superoxide dismutase gene transcription mediated by platelet-derived growth factor. FASEB J 2001;15:2025-2026.
1028. Dioum EM, Chen R, Alexander MS, et al. Regulation of hypoxia-inducible factor 2alpha signaling by the stress-responsive deacetylase sirtuin 1. Science 2009;324:1289-1293.
1029. Adachi M, Osawa Y, Uchinami H, Kitamura T, Accili D, Brenner DA. The forkhead transcription factor FoxO1 regulates proliferation and transdifferentiation of hepatic stellate cells. Gastroenterology 2007;132:1434-1446.
1030. Jacobs KM, Pennington JD, Bisht KS, et al. SIRT3 interacts with the daf-16 homolog FOXO3a in the mitochondria, as well as increases FOXO3a dependent gene expression. Int J Biol Sci 2008;4:291-299.
1031. Gao YH, Li CX, Shen SM, et al. Hypoxia-inducible factor 1alpha mediates the down-regulation of superoxide dismutase 2 in von Hippel-Lindau deficient renal clear cell carcinoma. Biochem Biophys Res Commun 2013;435:46-51.
1032. Creaven M, Hans F, Mutskov V, et al. Control of the histone-acetyltransferase activity of Tip60 by the HIV-1 transactivator protein, Tat. Biochemistry 1999;38:8826-8830.
1033. Fernandez-Zapico ME, Mladek A, Ellenrieder V, Folch-Puy E, Miller L, Urrutia R. An mSin3A interaction domain links the transcriptional activity of KLF11 with its role in growth regulation. EMBO J 2003;22:4748-4758.
1034. Kuo S, Chesrown SE, Mellott JK, Rogers RJ, Hsu JL, Nick HS. In vivo architecture of the manganese superoxide dismutase promoter. J Biol Chem 1999;274:3345-3354.
1035. Du K, Yu Y, Zhang D, et al. NFkappaB1 (p50) suppresses SOD2 expression by inhibiting FoxO3a transactivation in a miR190/PHLPP1/Akt-dependent axis. Mol Biol Cell 2013;24:3577-3583.
1036. Andres-Mateos E, Perier C, Zhang L, et al. DJ-1 gene deletion reveals that DJ-1 is an atypical peroxiredoxin-like peroxidase. Proc Natl Acad Sci U S A 2007;104:14807-14812.

1037. Olmos Y, Valle I, Borniquel S, et al. Mutual dependence of Foxo3a and PGC-1alpha in the induction of oxidative stress genes. J Biol Chem 2009;284:14476-14484.
1038. Leeman JR, Weniger MA, Barth TF, Gilmore TD. Deletion analysis and alternative splicing define a transactivation inhibitory domain in human oncoprotein REL. Oncogene 2008;27:6770-6781.
1039. Jung JE, Kim GS, Narasimhan P, Song YS, Chan PH. Regulation of Mn-superoxide dismutase activity and neuroprotection by STAT3 in mice after cerebral ischemia. J Neurosci 2009;29:7003-7014.
1040. Rawat SJ, Creasy CL, Peterson JR, Chernoff J. The tumor suppressor Mst1 promotes changes in the cellular redox state by phosphorylation and inactivation of peroxiredoxin-1 protein. J Biol Chem 2013;288:8762-8771.
1041. Zhu CH, Huang Y, Oberley LW, Domann FE. A family of AP-2 proteins down-regulate manganese superoxide dismutase expression. J Biol Chem 2001;276:14407-14413.
1042. Bakthavatchalu V, Dey S, Xu Y, et al. Manganese superoxide dismutase is a mitochondrial fidelity protein that protects Polgamma against UV-induced inactivation. Oncogene 2012;31:2129-2139.
1043. Smits P, Lefebvre V. Sox5 and Sox6 are required for notochord extracellular matrix sheath formation, notochord cell survival and development of the nucleus pulposus of intervertebral discs. Development 2003;130:1135-1148.
1044. Yoon BS, Ovchinnikov DA, Yoshii I, Mishina Y, Behringer RR, Lyons KM. Bmpr1a and Bmpr1b have overlapping functions and are essential for chondrogenesis in vivo. Proc Natl Acad Sci U S A 2005;102:5062-5067.
1045. Anders L, Ke N, Hydbring P, et al. A systematic screen for CDK4/6 substrates links FOXM1 phosphorylation to senescence suppression in cancer cells. Cancer Cell 2011;20:620-634.
1046. Youssef KK, Lapouge G, Bouvree K, et al. Adult interfollicular tumour-initiating cells are reprogrammed into an embryonic hair follicle progenitor-like fate during basal cell carcinoma initiation. Nat Cell Biol 2012;14:1282-1294.
1047. Hojo H, Ohba S, Taniguchi K, et al. Hedgehog-Gli activators direct osteo-chondrogenic function of bone morphogenetic protein toward osteogenesis in the perichondrium. J Biol Chem 2013;288:9924-9932.
1048. Tanaka S, Suto A, Iwamoto T, et al. Sox5 and c-Maf cooperatively induce Th17 cell differentiation via RORgammat induction as downstream targets of Stat3. J Exp Med 2014;211:1857-1874.
1049. Kimura A, Inose H, Yano F, et al. Runx1 and Runx2 cooperate during sternal morphogenesis. Development 2010;137:1159-1167.
1050. Engelen E, Akinci U, Bryne JC, et al. Sox2 cooperates with Chd7 to regulate genes that are mutated in human syndromes. Nat Genet 2011;43:607-611.
1051. Daftary GS, Tetrault AM, Jorgensen EM, Sarno J, Taylor HS. A novel role for the AAA ATPase spastin as a HOXA10 transcriptional corepressor in Ishikawa endometrial cells. Mol Endocrinol 2011;25:1539-1549.
1052. Sung CK, Choi WS, Sanchez-Margalet V. Guanosine triphosphatase-activating protein-associated protein, but not src-associated protein p68 in mitosis, is a part of insulin signaling complexes. Endocrinology 1998;139:2392-2398.
1053. Doi H, Adachi H, Katsuno M, et al. p62/SQSTM1 differentially removes the toxic mutant androgen receptor via autophagy and inclusion formation in a spinal and bulbar muscular atrophy mouse model. J Neurosci 2013;33:7710-7727.
1054. Lee J, Kim HR, Quinley C, et al. Autophagy suppresses interleukin-1beta (IL-1beta) signaling by activation of p62 degradation via lysosomal and proteasomal pathways. J Biol Chem 2012;287:4033-4040.
1055. Fracchiolla D, Sawa-Makarska J, Zens B, et al. Mechanism of cargo-directed Atg8 conjugation during selective autophagy. Elife 2016;5.
1056. Chevrier N, Mertins P, Artyomov MN, et al. Systematic discovery of TLR signaling components delineates viral-sensing circuits. Cell 2011;147:853-867.

1057. Zhou L, Wang H, Chen D, Gao F, Ying Z, Wang G. p62/sequestosome 1 regulates aggresome formation of pathogenic ataxin-3 with expanded polyglutamine. Int J Mol Sci 2014;15:14997-15010.
1058. Gamerdinger M, Hajieva P, Kaya AM, Wolfrum U, Hartl FU, Behl C. Protein quality control during aging involves recruitment of the macroautophagy pathway by BAG3. EMBO J 2009;28:889-901.
1059. Zhou L, Wang H, Ren H, Hu Q, Ying Z, Wang G. Bcl-2 Decreases the Affinity of SQSTM1/p62 to Poly-Ubiquitin Chains and Suppresses the Aggregation of Misfolded Protein in Neurodegenerative Disease. Mol Neurobiol 2015;52:1180-1189.
1060. Tang F, Wang B, Li N, et al. RNF185, a novel mitochondrial ubiquitin E3 ligase, regulates autophagy through interaction with BNIP1. PLoS One 2011;6:e24367.
1061. Pridgeon JW, Geetha T, Wooten MW. A Method to Identify p62's UBA Domain Interacting Proteins. Biol Proced Online 2003;5:228-237.
1062. Huang S, Okamoto K, Yu C, Sinicrope FA. p62/sequestosome-1 up-regulation promotes ABT-263-induced caspase-8 aggregation/activation on the autophagosome. J Biol Chem 2013;288:33654-33666.
1063. Liang XL, Wei SQ, Lee SJ, et al. p62 Sequestosome 1/Light Chain 3b Complex Confers Cytoprotection on Lung Epithelial Cells after Hyperoxia. Am J Resp Cell Mol 2013;48:489-496.
1064. Linares JF, Amanchy R, Greis K, Diaz-Meco MT, Moscat J. Phosphorylation of p62 by cdk1 controls the timely transit of cells through mitosis and tumor cell proliferation. Mol Cell Biol 2011;31:105-117.
1065. Villella VR, Esposito S, Bruscia EM, et al. Disease-relevant proteostasis regulation of cystic fibrosis transmembrane conductance regulator. Cell Death Differ 2013;20:1101-1115.
1066. Tanji K, Zhang HX, Mori F, Kakita A, Takahashi H, Wakabayashi K. p62/sequestosome 1 binds to TDP-43 in brains with frontotemporal lobar degeneration with TDP-43 inclusions. J Neurosci Res 2012;90:2034-2042.
1067. Mori Y, Mori T, Wakabayashi M, et al. Involvement of selective autophagy mediated by p62/SQSTM1 in KLHL3-dependent WNK4 degradation. Biochem J 2015;472:33-41.
1068. Kim JY, Ozato K. The sequestosome 1/p62 attenuates cytokine gene expression in activated macrophages by inhibiting IFN regulatory factor 8 and TNF receptor-associated factor 6/NF-kappaB activity. J Immunol 2009;182:2131-2140.
1069. Rezvani K, Teng Y, Shim D, De Biasi M. Nicotine regulates multiple synaptic proteins by inhibiting proteasomal activity. J Neurosci 2007;27:10508-10519.
1070. Liu XD, Yao J, Tripathi DN, et al. Autophagy mediates HIF2alpha degradation and suppresses renal tumorigenesis. Oncogene 2015;34:2450-2460.
1071. Jin Z, Li Y, Pitti R, et al. Cullin3-based polyubiquitination and p62-dependent aggregation of caspase-8 mediate extrinsic apoptosis signaling. Cell 2009;137:721-735.
1072. Liang X, Wei SQ, Lee SJ, et al. p62 sequestosome 1/light chain 3b complex confers cytoprotection on lung epithelial cells after hyperoxia. Am J Respir Cell Mol Biol 2013;48:489-496.
1073. Martins-Marques T, Catarino S, Zuzarte M, et al. Ischaemia-induced autophagy leads to degradation of gap junction protein connexin43 in cardiomyocytes. Biochem J 2015;467:231-245.
1074. Tsai LC, Xie L, Dore K, et al. Zeta Inhibitory Peptide Disrupts Electrostatic Interactions That Maintain Atypical Protein Kinase C in Its Active Conformation on the Scaffold p62. J Biol Chem 2015;290:21845-21856.
1075. Jiang J, Parameshwaran K, Seibenhener ML, et al. AMPA receptor trafficking and synaptic plasticity require SQSTM1/p62. Hippocampus 2009;19:392-406.
1076. Liu XW, Cai TY, Zhu H, et al. Q6, a novel hypoxia-targeted drug, regulates hypoxia-inducible factor signaling via an autophagy-dependent mechanism in hepatocellular carcinoma. Autophagy 2014;10:111-122.
1077. Filimonenko M, Isakson P, Finley KD, et al. The selective macroautophagic degradation of aggregated proteins requires the PI3P-binding protein Alfy. Mol Cell 2010;38:265-279.

1078. Zotti T, Scudiero I, Settembre P, et al. TRAF6-mediated ubiquitination of NEMO requires p62/sequestosome-1. Mol Immunol 2014;58:27-31.
1079. Milan E, Perini T, Resnati M, et al. A plastic SQSTM1/p62-dependent autophagic reserve maintains proteostasis and determines proteasome inhibitor susceptibility in multiple myeloma cells. Autophagy 2015;11:1161-1178.
1080. Sanz L, Diaz-Meco MT, Nakano H, Moscat J. The atypical PKC-interacting protein p62 channels NF-kappaB activation by the IL-1-TRAF6 pathway. EMBO J 2000;19:1576-1586.
1081. Nakashima H, Nguyen T, Goins WF, Chiocca EA. Interferon-stimulated gene 15 (ISG15) and ISG15-linked proteins can associate with members of the selective autophagic process, histone deacetylase 6 (HDAC6) and SQSTM1/p62. J Biol Chem 2015;290:1485-1495.
1082. Guo L, Huang JX, Liu Y, et al. Transactivation of Atg4b by C/EBPbeta promotes autophagy to facilitate adipogenesis. Mol Cell Biol 2013;33:3180-3190.
1083. Zatloukal B, Kufferath I, Thueringer A, Landegren U, Zatloukal K, Haybaeck J. Sensitivity and specificity of in situ proximity ligation for protein interaction analysis in a model of steatohepatitis with Mallory-Denk bodies. PLoS One 2014;9:e96690.
1084. Joung I, Strominger JL, Shin J. Molecular cloning of a phosphotyrosine-independent ligand of the p56lck SH2 domain. Proc Natl Acad Sci U S A 1996;93:5991-5995.
1085. Komatsu M, Waguri S, Koike M, et al. Homeostatic levels of p62 control cytoplasmic inclusion body formation in autophagy-deficient mice. Cell 2007;131:1149-1163.
1086. Nakamura K, Kimple AJ, Siderovski DP, Johnson GL. PB1 domain interaction of p62/sequestosome 1 and MEKK3 regulates NF-kappaB activation. J Biol Chem 2010;285:2077-2089.
1087. Rui YN, Xu Z, Patel B, et al. Huntingtin functions as a scaffold for selective macroautophagy. Nat Cell Biol 2015;17:262-275.
1088. Lambert JP, Tucholska M, Go C, Knight JD, Gingras AC. Proximity biotinylation and affinity purification are complementary approaches for the interactome mapping of chromatin-associated protein complexes. J Proteomics 2015;118:81-94.
1089. Duran A, Amanchy R, Linares JF, et al. p62 is a key regulator of nutrient sensing in the mTORC1 pathway. Mol Cell 2011;44:134-146.
1090. Into T, Inomata M, Niida S, Murakami Y, Shibata K. Regulation of MyD88 aggregation and the MyD88-dependent signaling pathway by sequestosome 1 and histone deacetylase 6. J Biol Chem 2010;285:35759-35769.
1091. Jain A, Lamark T, Sjottem E, et al. p62/SQSTM1 is a target gene for transcription factor NRF2 and creates a positive feedback loop by inducing antioxidant response element-driven gene transcription. J Biol Chem 2010;285:22576-22591.
1092. Williams JA, Thomas AM, Li G, et al. Tissue specific induction of p62/Sqstm1 by farnesoid X receptor. PLoS One 2012;7:e43961.
1093. Marcus SL, Winrow CJ, Capone JP, Rachubinski RA. A p56(lck) ligand serves as a coactivator of an orphan nuclear hormone receptor. J Biol Chem 1996;271:27197-27200.
1094. Zheng C, Geetha T, Gearing M, Babu JR. Amyloid beta-abrogated TrkA ubiquitination in PC12 cells analogous to Alzheimer's disease. J Neurochem 2015;133:919-925.
1095. Liu Z, Chen P, Gao H, et al. Ubiquitylation of autophagy receptor Optineurin by HACE1 activates selective autophagy for tumor suppression. Cancer Cell 2014;26:106-120.
1096. Seibenhener ML, Babu JR, Geetha T, Wong HC, Krishna NR, Wooten MW. Sequestosome 1/p62 is a polyubiquitin chain binding protein involved in ubiquitin proteasome degradation. Mol Cell Biol 2004;24:8055-8068.
1097. Besche HC, Haas W, Gygi SP, Goldberg AL. Isolation of mammalian 26S proteasomes and p97/VCP complexes using the ubiquitin-like domain from HHR23B reveals novel proteasome-associated proteins. Biochemistry 2009;48:2538-2549.

1098. Tsuchiya M, Isogai S, Taniguchi H, et al. Selective autophagic receptor p62 regulates the abundance of transcriptional coregulator ARIP4 during nutrient starvation. Sci Rep 2015;5:14498.
1099. Brajenovic M, Joberty G, Kuster B, Bouwmeester T, Drewes G. Comprehensive proteomic analysis of human Par protein complexes reveals an interconnected protein network. J Biol Chem 2004;279:12804-12811.
1100. Yang L, Chai W, Wang Y, et al. Reactive oxygen species regulate the differentiation of acute promyelocytic leukemia cells through HMGB1-mediated autophagy. Am J Cancer Res 2015;5:714-725.
1101. Shimokawa N, Haglund K, Holter SM, et al. CIN85 regulates dopamine receptor endocytosis and governs behaviour in mice. EMBO J 2010;29:2421-2432.
1102. Gal J, Strom AL, Kilty R, Zhang F, Zhu H. p62 accumulates and enhances aggregate formation in model systems of familial amyotrophic lateral sclerosis. J Biol Chem 2007;282:11068-11077.
1103. Urbanczyk A, Enz R. Spartin recruits PKC-zeta via the PKC-zeta-interacting proteins ZIP1 and ZIP3 to lipid droplets. J Neurochem 2011;118:737-748.
1104. Watanabe Y, Tanaka M. p62/SQSTM1 in autophagic clearance of a non-ubiquitylated substrate. J Cell Sci 2011;124:2692-2701.
1105. Kang JH, Lee JS, Hong D, et al. Renal cell carcinoma escapes death by p53 depletion through transglutaminase 2-chaperoned autophagy. Cell Death Dis 2016;7:e2163.
1106. Greenfeld H, Takasaki K, Walsh MJ, et al. TRAF1 Coordinates Polyubiquitin Signaling to Enhance Epstein-Barr Virus LMP1-Mediated Growth and Survival Pathway Activation. PLoS Pathog 2015;11:e1004890.
1107. Hua F, Li K, Yu JJ, et al. TRB3 links insulin/IGF to tumour promotion by interacting with p62 and impeding autophagic/proteasomal degradations. Nat Commun 2015;6:7951.
1108. Qiang L, Zhao B, Ming M, et al. Regulation of cell proliferation and migration by p62 through stabilization of Twist1. Proc Natl Acad Sci U S A 2014;111:9241-9246.
1109. Raman M, Sergeev M, Garnaas M, et al. Systematic proteomics of the VCP-UBXD adaptor network identifies a role for UBXN10 in regulating ciliogenesis. Nat Cell Biol 2015;17:1356-1369.
1110. Ro SH, Semple IA, Park H, et al. Sestrin2 promotes Unc-51-like kinase 1 mediated phosphorylation of p62/sequestosome-1. FEBS J 2014;281:3816-3827.
1111. Rae FK, Martinez G, Gillinder KR, et al. Anlaysis of complementary expression profiles following WT1 induction versus repression reveals the cholesterol/fatty acid synthetic pathways as a possible major target of WT1. Oncogene 2004;23:3067-3079.
1112. Aizawa H, Hu SC, Bobb K, et al. Dendrite development regulated by CREST, a calcium-regulated transcriptional activator. Science 2004;303:197-202.
1113. Ravasi T, Suzuki H, Cannistraci CV, et al. An atlas of combinatorial transcriptional regulation in mouse and man. Cell 2010;140:744-752.
1114. Ho L, Ronan JL, Wu J, et al. An embryonic stem cell chromatin remodeling complex, esBAF, is essential for embryonic stem cell self-renewal and pluripotency. Proc Natl Acad Sci U S A 2009;106:5181-5186.
1115. Qiu Z, Ghosh A. A calcium-dependent switch in a CREST-BRG1 complex regulates activity-dependent gene expression. Neuron 2008;60:775-787.
1116. Staahl BT, Tang J, Wu W, et al. Kinetic analysis of npBAF to nBAF switching reveals exchange of SS18 with CREST and integration with neural developmental pathways. J Neurosci 2013;33:10348-10361.
1117. Mislow JM, Holaska JM, Kim MS, et al. Nesprin-1alpha self-associates and binds directly to emerin and lamin A in vitro. FEBS Lett 2002;525:135-140.
1118. Kim DI, Birendra KC, Zhu W, Motamedchaboki K, Doye V, Roux KJ. Probing nuclear pore complex architecture with proximity-dependent biotinylation. Proc Natl Acad Sci U S A 2014;111:E2453-2461.
1119. Giannone RJ, McDonald HW, Hurst GB, Shen RF, Wang Y, Liu Y. The protein network surrounding the human telomere repeat binding factors TRF1, TRF2, and POT1. PLoS One 2010;5:e12407.

1120. Dubois T, Howell S, Zemlickova E, Aitken A. Identification of casein kinase Ialpha interacting protein partners. FEBS Lett 2002;517:167-171.
1121. Li C, Ullrich B, Zhang JZ, Anderson RG, Brose N, Sudhof TC. Ca(2+)-dependent and -independent activities of neural and non-neural synaptotagmins. Nature 1995;375:594-599.
1122. Mizutani A, Fukuda M, Ibata K, Shiraishi Y, Mikoshiba K. SYNCRIP, a cytoplasmic counterpart of heterogeneous nuclear ribonucleoprotein R, interacts with ubiquitous synaptotagmin isoforms. J Biol Chem 2000;275:9823-9831.
1123. Haberman Y, Grimberg E, Fukuda M, Sagi-Eisenberg R. Synaptotagmin IX, a possible linker between the perinuclear endocytic recycling compartment and the microtubules. J Cell Sci 2003;116:4307-4318.
1124. Lee BH, Min X, Heise CJ, et al. WNK1 phosphorylates synaptotagmin 2 and modulates its membrane binding. Mol Cell 2004;15:741-751.
1125. Hoffmann A, Chiang CM, Oelgeschlager T, et al. A histone octamer-like structure within TFIID. Nature 1996;380:356-359.
1126. Kwon I, Kato M, Xiang S, et al. Phosphorylation-regulated binding of RNA polymerase II to fibrous polymers of low-complexity domains. Cell 2013;155:1049-1060.
1127. Wilker EW, van Vugt MA, Artim SA, et al. 14-3-3sigma controls mitotic translation to facilitate cytokinesis. Nature 2007;446:329-332.
1128. Hoey T, Zhang S, Schmidt N, et al. Distinct requirements for the naturally occurring splice forms Stat4alpha and Stat4beta in IL-12 responses. EMBO J 2003;22:4237-4248.
1129. Bertolotti A, Melot T, Acker J, Vigneron M, Delattre O, Tora L. EWS, but not EWS-FLI-1, is associated with both TFIID and RNA polymerase II: interactions between two members of the TET family, EWS and hTAFII68, and subunits of TFIID and RNA polymerase II complexes. Mol Cell Biol 1998;18:1489-1497.
1130. Swarup V, Audet JN, Phaneuf D, Kriz J, Julien JP. Abnormal regenerative responses and impaired axonal outgrowth after nerve crush in TDP-43 transgenic mouse models of amyotrophic lateral sclerosis. J Neurosci 2012;32:18186-18195.
1131. Liu X, Li D, Zhang W, Guo M, Zhan Q. Long non-coding RNA gadd7 interacts with TDP-43 and regulates Cdk6 mRNA decay. EMBO J 2012;31:4415-4427.
1132. Buratti E, Baralle FE. Characterization and functional implications of the RNA binding properties of nuclear factor TDP-43, a novel splicing regulator of CFTR exon 9. J Biol Chem 2001;276:36337-36343.
1133. Uchida T, Tamaki Y, Ayaki T, et al. CUL2-mediated clearance of misfolded TDP-43 is paradoxically affected by VHL in oligodendrocytes in ALS. Sci Rep 2016;6:19118.
1134. Kawahara Y, Mieda-Sato A. TDP-43 promotes microRNA biogenesis as a component of the Drosha and Dicer complexes. Proc Natl Acad Sci U S A 2012;109:3347-3352.
1135. Shirasaki DI, Greiner ER, Al-Ramahi I, et al. Network organization of the huntingtin proteomic interactome in mammalian brain. Neuron 2012;75:41-57.
1136. !!! INVALID CITATION !!! 412.
1137. Hans F, Fiesel FC, Strong JC, et al. UBE2E ubiquitin-conjugating enzymes and ubiquitin isopeptidase Y regulate TDP-43 protein ubiquitination. J Biol Chem 2014;289:19164-19179.
1138. Volkening K, Leystra-Lantz C, Yang W, Jaffee H, Strong MJ. Tar DNA binding protein of 43 kDa (TDP-43), 14-3-3 proteins and copper/zinc superoxide dismutase (SOD1) interact to modulate NFL mRNA stability. Implications for altered RNA processing in amyotrophic lateral sclerosis (ALS). Brain Res 2009;1305:168-182.
1139. Lu YC, Kim I, Lye E, et al. Differential role for c-Rel and C/EBPbeta/delta in TLR-mediated induction of proinflammatory cytokines. J Immunol 2009;182:7212-7221.

1140. Cassel JA, Reitz AB. Ubiquilin-2 (UBQLN2) binds with high affinity to the C-terminal region of TDP-43 and modulates TDP-43 levels in H4 cells: characterization of inhibition by nucleic acids and 4-aminoquinolines. Biochim Biophys Acta 2013;1834:964-971.
1141. Unterstab G, Ludwig S, Anton A, et al. Viral targeting of the interferon-{beta}-inducing Traf family member-associated NF-{kappa}B activator (TANK)-binding kinase-1. Proc Natl Acad Sci U S A 2005;102:13640-13645.
1142. Friedman CS, O'Donnell MA, Legarda-Addison D, et al. The tumour suppressor CYLD is a negative regulator of RIG-I-mediated antiviral response. EMBO Rep 2008;9:930-936.
1143. Soulat D, Burckstummer T, Westermayer S, et al. The DEAD-box helicase DDX3X is a critical component of the TANK-binding kinase 1-dependent innate immune response. EMBO J 2008;27:2135-2146.
1144. Chau TL, Gioia R, Gatot JS, et al. Are the IKKs and IKK-related kinases TBK1 and IKK-epsilon similarly activated? Trends Biochem Sci 2008;33:171-180.
1145. Simicek M, Lievens S, Laga M, et al. The deubiquitylase USP33 discriminates between RALB functions in autophagy and innate immune response. Nat Cell Biol 2013;15:1220-1230.
1146. Sharif-Askari E, Vassen L, Kosan C, et al. Zinc finger protein Gfi1 controls the endotoxin-mediated Toll-like receptor inflammatory response by antagonizing NF-kappaB p65. Mol Cell Biol 2010;30:3929-3942.
1147. Al-Hakim AK, Bashkurov M, Gingras AC, Durocher D, Pelletier L. Interaction proteomics identify NEURL4 and the HECT E3 ligase HERC2 as novel modulators of centrosome architecture. Mol Cell Proteomics 2012;11:M111 014233.
1148. Liu XY, Chen W, Wei B, Shan YF, Wang C. IFN-induced TPR protein IFIT3 potentiates antiviral signaling by bridging MAVS and TBK1. J Immunol 2011;187:2559-2568.
1149. Jin J, Xiao Y, Chang JH, et al. The kinase TBK1 controls IgA class switching by negatively regulating noncanonical NF-kappaB signaling. Nat Immunol 2012;13:1101-1109.
1150. Ikeda F, Hecker CM, Rozenknop A, et al. Involvement of the ubiquitin-like domain of TBK1/IKK-i kinases in regulation of IFN-inducible genes. EMBO J 2007;26:3451-3462.
1151. Hacker H, Redecke V, Blagoev B, et al. Specificity in Toll-like receptor signalling through distinct effector functions of TRAF3 and TRAF6. Nature 2006;439:204-207.
1152. Chou MM, Hanafusa H. A novel ligand for SH3 domains. The Nck adaptor protein binds to a serine/threonine kinase via an SH3 domain. J Biol Chem 1995;270:7359-7364.
1153. Jung SY, Malovannaya A, Wei J, O'Malley BW, Qin J. Proteomic analysis of steady-state nuclear hormone receptor coactivator complexes. Mol Endocrinol 2005;19:2451-2465.
1154. Persaud A, Alberts P, Amsen EM, et al. Comparison of substrate specificity of the ubiquitin ligases Nedd4 and Nedd4-2 using proteome arrays. Mol Syst Biol 2009;5:333.
1155. Freischmidt A, Wieland T, Richter B, et al. Haploinsufficiency of TBK1 causes familial ALS and fronto-temporal dementia. Nat Neurosci 2015;18:631-636.
1156. Brikos C, O'Neill LA. Signalling of toll-like receptors. Handb Exp Pharmacol 2008:21-50.
1157. Charoenthongtrakul S, Gao L, Parvatiyar K, Lee D, Harhaj EW. RING finger protein 11 targets TBK1/IKKi kinases to inhibit antiviral signaling. PLoS One 2013;8:e53717.
1158. Kawai T, Akira S. The role of pattern-recognition receptors in innate immunity: update on Toll-like receptors. Nat Immunol 2010;11:373-384.
1159. Johnsen IB, Nguyen TT, Bergstroem B, Fitzgerald KA, Anthonsen MW. The tyrosine kinase c-Src enhances RIG-I (retinoic acid-inducible gene I)-elicited antiviral signaling. J Biol Chem 2009;284:19122-19131.
1160. Parker D, Cohen TS, Alhede M, Harfenist BS, Martin FJ, Prince A. Induction of type I interferon signaling by Pseudomonas aeruginosa is diminished in cystic fibrosis epithelial cells. Am J Respir Cell Mol Biol 2012;46:6-13.

1161. Chen H, Sun H, You F, et al. Activation of STAT6 by STING is critical for antiviral innate immunity. Cell 2011;147:436-446.
1162. Renner F, Saul VV, Pagenstecher A, Wittwer T, Schmitz ML. Inducible SUMO modification of TANK alleviates its repression of TLR7 signalling. EMBO Rep 2011;12:129-135.
1163. Gao L, Coope H, Grant S, Ma A, Ley SC, Harhaj EW. ABIN1 protein cooperates with TAX1BP1 and A20 proteins to inhibit antiviral signaling. J Biol Chem 2011;286:36592-36602.
1164. Kuai J, Wooters J, Hall JP, et al. NAK is recruited to the TNFR1 complex in a TNFalpha-dependent manner and mediates the production of RANTES: identification of endogenous TNFR-interacting proteins by a proteomic approach. J Biol Chem 2004;279:53266-53271.
1165. Zha J, Han KJ, Xu LG, et al. The Ret finger protein inhibits signaling mediated by the noncanonical and canonical IkappaB kinase family members. J Immunol 2006;176:1072-1080.
1166. Nakhaei P, Sun Q, Solis M, et al. IkappaB kinase epsilon-dependent phosphorylation and degradation of X-linked inhibitor of apoptosis sensitizes cells to virus-induced apoptosis. J Virol 2012;86:726-737.
1167. Krapivinsky G, Krapivinsky L, Manasian Y, Clapham DE. The TRPM7 chanzyme is cleaved to release a chromatin-modifying kinase. Cell 2014;157:1061-1072.
1168. Ryazanova LV, Dorovkov MV, Ansari A, Ryazanov AG. Characterization of the protein kinase activity of TRPM7/ChaK1, a protein kinase fused to the transient receptor potential ion channel. J Biol Chem 2004;279:3708-3716.
1169. Clark K, Middelbeek J, Dorovkov MV, et al. The alpha-kinases TRPM6 and TRPM7, but not eEF-2 kinase, phosphorylate the assembly domain of myosin IIA, IIB and IIC. FEBS Lett 2008;582:2993-2997.
1170. Clark K, Langeslag M, van Leeuwen B, et al. TRPM7, a novel regulator of actomyosin contractility and cell adhesion. EMBO J 2006;25:290-301.
1171. Runnels LW, Yue L, Clapham DE. The TRPM7 channel is inactivated by PIP(2) hydrolysis. Nat Cell Biol 2002;4:329-336.
1172. Li Y, Franklin S, Zhang MJ, Vondriska TM. Highly efficient purification of protein complexes from mammalian cells using a novel streptavidin-binding peptide and hexahistidine tandem tag system: application to Bruton's tyrosine kinase. Protein Sci 2011;20:140-149.
1173. Ligon LA, Shelly SS, Tokito MK, Holzbaur EL. Microtubule binding proteins CLIP-170, EB1, and p150Glued form distinct plus-end complexes. FEBS Lett 2006;580:1327-1332.
1174. Tian Q, Feetham MC, Tao WA, et al. Proteomic analysis identifies that 14-3-3zeta interacts with beta-catenin and facilitates its activation by Akt. Proc Natl Acad Sci U S A 2004;101:15370-15375.
1175. Teixeira FR, Yokoo S, Gartner CA, et al. Identification of FBXO25-interacting proteins using an integrated proteomics approach. Proteomics 2010;10:2746-2757.
1176. Vecchione A, Cooper HJ, Trim KJ, Akbarzadeh S, Heath JK, Wheldon LM. Protein partners in the life history of activated fibroblast growth factor receptors. Proteomics 2007;7:4565-4578.
1177. Kean MJ, Ceccarelli DF, Goudreault M, et al. Structure-function analysis of core STRIPAK Proteins: a signaling complex implicated in Golgi polarization. J Biol Chem 2011;286:25065-25075.
1178. Titz B, Low T, Komisopoulou E, Chen SS, Rubbi L, Graeber TG. The proximal signaling network of the BCR-ABL1 oncogene shows a modular organization. Oncogene 2010;29:5895-5910.
1179. Husi H, Ward MA, Choudhary JS, Blackstock WP, Grant SG. Proteomic analysis of NMDA receptor-adhesion protein signaling complexes. Nat Neurosci 2000;3:661-669.
1180. Law BM, Spain VA, Leinster VH, et al. A direct interaction between leucine-rich repeat kinase 2 and specific beta-tubulin isoforms regulates tubulin acetylation. J Biol Chem 2014;289:895-908.
1181. Nitzsche A, Paszkowski-Rogacz M, Matarese F, et al. RAD21 cooperates with pluripotency transcription factors in the maintenance of embryonic stem cell identity. PLoS One 2011;6:e19470.
1182. Latouche C, Sainte-Marie Y, Steenman M, et al. Molecular signature of mineralocorticoid receptor signaling in cardiomyocytes: from cultured cells to mouse heart. Endocrinology 2010;151:4467-4476.

1183. Glatter T, Wepf A, Aebersold R, Gstaiger M. An integrated workflow for charting the human interaction proteome: insights into the PP2A system. Mol Syst Biol 2009;5:237.
1184. Qiu T, Grizzle WE, Oelschlager DK, Shen X, Cao X. Control of prostate cell growth: BMP antagonizes androgen mitogenic activity with incorporation of MAPK signals in Smad1. EMBO J 2007;26:346-357.
1185. Krebs DL, Uren RT, Metcalf D, et al. SOCS-6 binds to insulin receptor substrate 4, and mice lacking the SOCS-6 gene exhibit mild growth retardation. Mol Cell Biol 2002;22:4567-4578.
1186. Bhaskar K, Shareef MM, Sharma VM, et al. Co-purification and localization of Munc18-1 (p67) and Cdk5 with neuronal cytoskeletal proteins. Neurochem Int 2004;44:35-44.
1187. Takeuchi T, Inoue S, Yokosawa H. Identification and Herc5-mediated ISGylation of novel target proteins. Biochem Biophys Res Commun 2006;348:473-477.
1188. Kirkpatrick DS, Hathaway NA, Hanna J, et al. Quantitative analysis of in vitro ubiquitinated cyclin B1 reveals complex chain topology. Nat Cell Biol 2006;8:700-710.
1189. Xia Y, Yan LH, Huang B, Liu M, Liu X, Huang C. Pathogenic mutation of UBQLN2 impairs its interaction with UBXD8 and disrupts endoplasmic reticulum-associated protein degradation. J Neurochem 2014;129:99-106.
1190. Kim TY, Kim E, Yoon SK, Yoon JB. Herp enhances ER-associated protein degradation by recruiting ubiquilins. Biochem Biophys Res Commun 2008;369:741-746.
1191. Zeng L, Wang B, Merillat SA, et al. Differential recruitment of UBQLN2 to nuclear inclusions in the polyglutamine diseases HD and SCA3. Neurobiol Dis 2015;82:281-288.
1192. Besche HC, Sha Z, Kukushkin NV, et al. Autoubiquitination of the 26S proteasome on Rpn13 regulates breakdown of ubiquitin conjugates. EMBO J 2014;33:1159-1176.
1193. Wu AL, Wang J, Zheleznyak A, Brown EJ. Ubiquitin-related proteins regulate interaction of vimentin intermediate filaments with the plasma membrane. Mol Cell 1999;4:619-625.
1194. Murillas R, Simms KS, Hatakeyama S, Weissman AM, Kuehn MR. Identification of developmentally expressed proteins that functionally interact with Nedd4 ubiquitin ligase. J Biol Chem 2002;277:2897-2907.
1195. Chang L, Monteiro MJ. Defective Proteasome Delivery of Polyubiquitinated Proteins by Ubiquilin-2 Proteins Containing ALS Mutations. PLoS One 2015;10:e0130162.
1196. Aguileta MA, Korac J, Durcan TM, et al. The E3 ubiquitin ligase parkin is recruited to the 26 S proteasome via the proteasomal ubiquitin receptor Rpn13. J Biol Chem 2015;290:7492-7505.
1197. Kang Y, Zhang N, Koepp DM, Walters KJ. Ubiquitin receptor proteins hHR23a and hPLIC2 interact. J Mol Biol 2007;365:1093-1101.
1198. Kleijnen MF, Shih AH, Zhou P, et al. The hPLIC proteins may provide a link between the ubiquitination machinery and the proteasome. Mol Cell 2000;6:409-419.
1199. Ford DL, Monteiro MJ. Dimerization of ubiquilin is dependent upon the central region of the protein: evidence that the monomer, but not the dimer, is involved in binding presenilins. Biochem J 2006;399:397-404.
1200. Muller CS, Haupt A, Bildl W, et al. Quantitative proteomics of the Cav2 channel nano-environments in the mammalian brain. Proc Natl Acad Sci U S A 2010;107:14950-14957.
1201. Junge HJ, Rhee JS, Jahn O, et al. Calmodulin and Munc13 form a Ca2+ sensor/effector complex that controls short-term synaptic plasticity. Cell 2004;118:389-401.
1202. Betz A, Okamoto M, Benseler F, Brose N. Direct interaction of the rat unc-13 homologue Munc13-1 with the N terminus of syntaxin. J Biol Chem 1997;272:2520-2526.
1203. Baron Y, Pedrioli PG, Tyagi K, et al. VAPB/ALS8 interacts with FFAT-like proteins including the p97 cofactor FAF1 and the ASNA1 ATPase. BMC Biol 2014;12:39.
1204. Rybak-Wolf A, Jens M, Murakawa Y, Herzog M, Landthaler M, Rajewsky N. A variety of dicer substrates in human and C. elegans. Cell 2014;159:1153-1167.

1205. Tsuda H, Han SM, Yang Y, et al. The amyotrophic lateral sclerosis 8 protein VAPB is cleaved, secreted, and acts as a ligand for Eph receptors. Cell 2008;133:963-977.
1206. Christianson JC, Olzmann JA, Shaler TA, et al. Defining human ERAD networks through an integrative mapping strategy. Nat Cell Biol 2011;14:93-105.
1207. Si W, Huang W, Zheng Y, et al. Dysfunction of the Reciprocal Feedback Loop between GATA3- and ZEB2-Nucleated Repression Programs Contributes to Breast Cancer Metastasis. Cancer Cell 2015;27:822-836.
1208. !!! INVALID CITATION !!! 870.
1209. Taguwa S, Okamoto T, Abe T, et al. Human butyrate-induced transcript 1 interacts with hepatitis C virus NS5A and regulates viral replication. J Virol 2008;82:2631-2641.
1210. Liu X, Miller BR, Rebec GV, Clemmer DE. Protein expression in the striatum and cortex regions of the brain for a mouse model of Huntington's disease. J Proteome Res 2007;6:3134-3142.
1211. Gong Y, Lee JN, Brown MS, Goldstein JL, Ye J. Juxtamembranous aspartic acid in Insig-1 and Insig-2 is required for cholesterol homeostasis. Proc Natl Acad Sci U S A 2006;103:6154-6159.
1212. Li G, Alexander EA, Schwartz JH. Syntaxin isoform specificity in the regulation of renal H+-ATPase exocytosis. J Biol Chem 2003;278:19791-19797.
1213. Nishimura Y, Hayashi M, Inada H, Tanaka T. Molecular cloning and characterization of mammalian homologues of vesicle-associated membrane protein-associated (VAMP-associated) proteins. Biochem Biophys Res Commun 1999;254:21-26.
1214. Lai Y, Song M, Hakala K, Weintraub ST, Shiio Y. The interaction of the von Hippel-Lindau tumor suppressor and heterochromatin protein 1. Arch Biochem Biophys 2012;518:103-110.
1215. Schaafhausen A, Rost S, Oldenburg J, Muller CR. Identification of VKORC1 interaction partners by split-ubiquitin system and coimmunoprecipitation. Thromb Haemost 2011;105:285-294.
1216. Palicharla VR, Maddika S. HACE1 mediated K27 ubiquitin linkage leads to YB-1 protein secretion. Cell Signal 2015;27:2355-2362.
1217. Saita S, Shirane M, Natume T, Iemura S, Nakayama KI. Promotion of neurite extension by protrudin requires its interaction with vesicle-associated membrane protein-associated protein. J Biol Chem 2009;284:13766-13777.
1218. Yu CC, Yang JC, Chang YC, et al. VCP phosphorylation-dependent interaction partners prevent apoptosis in Helicobacter pylori-infected gastric epithelial cells. PLoS One 2013;8:e55724.
1219. Rao R, Balusu R, Fiskus W, et al. Combination of pan-histone deacetylase inhibitor and autophagy inhibitor exerts superior efficacy against triple-negative human breast cancer cells. Mol Cancer Ther 2012;11:973-983.
1220. Wang H, Yu X, Guo C, et al. Enhanced endoplasmic reticulum entry of tumor antigen is crucial for cross-presentation induced by dendritic cell-targeted vaccination. J Immunol 2013;191:6010-6021.
1221. Elkabetz Y, Shapira I, Rabinovich E, Bar-Nun S. Distinct steps in dislocation of luminal endoplasmic reticulum-associated degradation substrates: roles of endoplamic reticulum-bound p97/Cdc48p and proteasome. J Biol Chem 2004;279:3980-3989.
1222. Lee JN, Zhang X, Feramisco JD, Gong Y, Ye J. Unsaturated fatty acids inhibit proteasomal degradation of Insig-1 at a postubiquitination step. J Biol Chem 2008;283:33772-33783.
1223. Yoshida Y, Adachi E, Fukiya K, Iwai K, Tanaka K. Glycoprotein-specific ubiquitin ligases recognize N-glycans in unfolded substrates. EMBO Rep 2005;6:239-244.
1224. Lu JP, Wang Y, Sliter DA, Pearce MM, Wojcikiewicz RJ. RNF170 protein, an endoplasmic reticulum membrane ubiquitin ligase, mediates inositol 1,4,5-trisphosphate receptor ubiquitination and degradation. J Biol Chem 2011;286:24426-24433.
1225. Abisambra J, Jinwal UK, Miyata Y, et al. Allosteric heat shock protein 70 inhibitors rapidly rescue synaptic plasticity deficits by reducing aberrant tau. Biol Psychiatry 2013;74:367-374.

1226. Marutani T, Maeda T, Tanabe C, et al. ER-stress-inducible Herp, facilitates the degradation of immature nicastrin. Biochim Biophys Acta 2011;1810:790-798.
1227. Tu LC, Yan X, Hood L, Lin B. Proteomics analysis of the interactome of N-myc downstream regulated gene 1 and its interactions with the androgen response program in prostate cancer cells. Mol Cell Proteomics 2007;6:575-588.
1228. Min T, Bodas M, Mazur S, Vij N. Critical role of proteostasis-imbalance in pathogenesis of COPD and severe emphysema. J Mol Med (Berl) 2011;89:577-593.
1229. Zhang Z, Wang Y, Li C, et al. The Transitional Endoplasmic Reticulum ATPase p97 Regulates the Alternative Nuclear Factor NF-kappaB Signaling via Partial Degradation of the NF-kappaB Subunit p100. J Biol Chem 2015;290:19558-19568.
1230. Fernandez-Arenas E, Calleja E, Martinez-Martin N, et al. beta-Arrestin-1 mediates the TCR-triggered re-routing of distal receptors to the immunological synapse by a PKC-mediated mechanism. EMBO J 2014;33:559-577.
1231. Grelle G, Kostka S, Otto A, et al. Identification of VCP/p97, carboxyl terminus of Hsp70-interacting protein (CHIP), and amphiphysin II interaction partners using membrane-based human proteome arrays. Mol Cell Proteomics 2006;5:234-244.
1232. Cid C, Garcia-Bonilla L, Camafeita E, Burda J, Salinas M, Alcazar A. Proteomic characterization of protein phosphatase 1 complexes in ischemia-reperfusion and ischemic tolerance. Proteomics 2007;7:3207-3218.
1233. Yakovlev VA. Nitric oxide-dependent downregulation of BRCA1 expression promotes genetic instability. Cancer Res 2013;73:706-715.
1234. Al-Hakim AK, Goransson O, Deak M, et al. 14-3-3 cooperates with LKB1 to regulate the activity and localization of QSK and SIK. J Cell Sci 2005;118:5661-5673.
1235. Lim PJ, Danner R, Liang J, et al. Ubiquilin and p97/VCP bind erasin, forming a complex involved in ERAD. J Cell Biol 2009;187:201-217.
1236. Braunstein I, Zach L, Allan S, Kalies KU, Stanhill A. Proteasomal degradation of preemptive quality control (pQC) substrates is mediated by an AIRAPL-p97 complex. Mol Biol Cell 2015;26:3719-3727.
1237. Arrojo EDR, Egri P, Jo S, Gereben B, Bianco AC. The type II deiodinase is retrotranslocated to the cytoplasm and proteasomes via p97/Atx3 complex. Mol Endocrinol 2013;27:2105-2115.
1238. Chen SF, Wu CH, Lee YM, et al. Caveolin-1 interacts with Derlin-1 and promotes ubiquitination and degradation of cyclooxygenase-2 via collaboration with p97 complex. J Biol Chem 2013;288:33462-33469.
1239. Zhang SH, Liu J, Kobayashi R, Tonks NK. Identification of the cell cycle regulator VCP (p97/CDC48) as a substrate of the band 4.1-related protein-tyrosine phosphatase PTPH1. J Biol Chem 1999;274:17806-17812.
1240. Hsu SH, Motiwala T, Roy S, et al. Methylation of the PTPRO gene in human hepatocellular carcinoma and identification of VCP as its substrate. J Cell Biochem 2013;114:1810-1818.
1241. Laco MN, Cortes L, Travis SM, Paulson HL, Rego AC. Valosin-containing protein (VCP/p97) is an activator of wild-type ataxin-3. PLoS One 2012;7:e43563.
1242. Chen N, Onisko B, Napoli JL. The nuclear transcription factor RARalpha associates with neuronal RNA granules and suppresses translation. J Biol Chem 2008;283:20841-20847.
1243. Ishigaki S, Hishikawa N, Niwa J, et al. Physical and functional interaction between Dorfin and Valosin-containing protein that are colocalized in ubiquitylated inclusions in neurodegenerative disorders. J Biol Chem 2004;279:51376-51385.
1244. Marti F, King PD. The p95-100 kDa ligand of the T cell-specific adaptor (TSAd) protein Src-homology-2 (SH2) domain implicated in TSAd nuclear import is p97 Valosin-containing protein (VCP). Immunol Lett 2005;97:235-243.

1245. Yang FC, Lin YH, Chen WH, et al. Interaction between salt-inducible kinase 2 (SIK2) and p97/valosin-containing protein (VCP) regulates endoplasmic reticulum (ER)-associated protein degradation in mammalian cells. J Biol Chem 2013;288:33861-33872.
1246. Cayli S, Erdemir F, Ocakli S, et al. Interaction between Smad1 and p97/VCP in rat testis and epididymis during the postnatal development. Reprod Sci 2012;19:190-201.
1247. Mosbech A, Gibbs-Seymour I, Kagias K, et al. DVC1 (C1orf124) is a DNA damage-targeting p97 adaptor that promotes ubiquitin-dependent responses to replication blocks. Nat Struct Mol Biol 2012;19:1084-1092.
1248. Liu S, Fu QS, Zhao J, Hu HY. Structural and mechanistic insights into the arginine/lysine-rich peptide motifs that interact with P97/VCP. Biochim Biophys Acta 2013;1834:2672-2678.
1249. LaLonde DP, Bretscher A. The UBX protein SAKS1 negatively regulates endoplasmic reticulum-associated degradation and p97-dependent degradation. J Biol Chem 2011;286:4892-4901.
1250. Alexandru G, Graumann J, Smith GT, Kolawa NJ, Fang R, Deshaies RJ. UBXD7 binds multiple ubiquitin ligases and implicates p97 in HIF1alpha turnover. Cell 2008;134:804-816.
1251. Dougherty U, Mustafi R, Sadiq F, et al. The renin-angiotensin system mediates EGF receptor-vitamin d receptor cross-talk in colitis-associated colon cancer. Clin Cancer Res 2014;20:5848-5859.
1252. Ting HJ, Yasmin-Karim S, Yan SJ, et al. A positive feedback signaling loop between ATM and the vitamin D receptor is critical for cancer chemoprevention by vitamin D. Cancer Res 2012;72:958-968.
1253. Guzey M, Takayama S, Reed JC. BAG1L enhances trans-activation function of the vitamin D receptor. J Biol Chem 2000;275:40749-40756.
1254. Deng C, Ueda E, Chen KE, et al. Prolactin blocks nuclear translocation of VDR by regulating its interaction with BRCA1 in osteosarcoma cells. Mol Endocrinol 2009;23:226-236.
1255. Malloy PJ, Feldman D. Inactivation of the human vitamin D receptor by caspase-3. Endocrinology 2009;150:679-686.
1256. Chen J, Doroudi M, Cheung J, Grozier AL, Schwartz Z, Boyan BD. Plasma membrane Pdia3 and VDR interact to elicit rapid responses to 1alpha,25(OH)(2)D(3). Cell Signal 2013;25:2362-2373.
1257. Luderer HF, Nazarian RM, Zhu ED, Demay MB. Ligand-dependent actions of the vitamin D receptor are required for activation of TGF-beta signaling during the inflammatory response to cutaneous injury. Endocrinology 2013;154:16-24.
1258. Pervin S, Hewison M, Braga M, et al. Down-regulation of vitamin D receptor in mammospheres: implications for vitamin D resistance in breast cancer and potential for combination therapy. PLoS One 2013;8:e53287.
1259. Thorne JL, Maguire O, Doig CL, et al. Epigenetic control of a VDR-governed feed-forward loop that regulates p21(waf1/cip1) expression and function in non-malignant prostate cells. Nucleic Acids Res 2011;39:2045-2056.
1260. Song CS, Echchgadda I, Seo YK, et al. An essential role of the CAAT/enhancer binding protein-alpha in the vitamin D-induced expression of the human steroid/bile acid-sulfotransferase (SULT2A1). Mol Endocrinol 2006;20:795-808.
1261. Potter JJ, Liu X, Koteish A, Mezey E. 1,25-dihydroxyvitamin D3 and its nuclear receptor repress human alpha1 (I) collagen expression and type I collagen formation. Liver Int 2013;33:677-686.
1262. Jimenez-Lara AM, Aranda A. Lysine 246 of the vitamin D receptor is crucial for ligand-dependent interaction with coactivators and transcriptional activity. J Biol Chem 1999;274:13503-13510.
1263. Arriagada G, Paredes R, Olate J, et al. Phosphorylation at serine 208 of the 1alpha,25-dihydroxy Vitamin D3 receptor modulates the interaction with transcriptional coactivators. J Steroid Biochem Mol Biol 2007;103:425-429.
1264. Palmer HG, Gonzalez-Sancho JM, Espada J, et al. Vitamin D(3) promotes the differentiation of colon carcinoma cells by the induction of E-cadherin and the inhibition of beta-catenin signaling. J Cell Biol 2001;154:369-387.

1265. Pan L, Matloob AF, Du J, et al. Vitamin D stimulates apoptosis in gastric cancer cells in synergy with trichostatin A /sodium butyrate-induced and 5-aza-2'-deoxycytidine-induced PTEN upregulation. FEBS J 2010;277:989-999.
1266. Dwivedi PP, Omdahl JL, Kola I, Hume DA, May BK. Regulation of rat cytochrome P450C24 (CYP24) gene expression. Evidence for functional cooperation of Ras-activated Ets transcription factors with the vitamin D receptor in 1,25-dihydroxyvitamin D(3)-mediated induction. J Biol Chem 2000;275:47-55.
1267. Towers TL, Staeva TP, Freedman LP. A two-hit mechanism for vitamin D3-mediated transcriptional repression of the granulocyte-macrophage colony-stimulating factor gene: vitamin D receptor competes for DNA binding with NFAT1 and stabilizes c-Jun. Mol Cell Biol 1999;19:4191-4199.
1268. Schuur ER, Loktev AV, Sharma M, Sun Z, Roth RA, Weigel RJ. Ligand-dependent interaction of estrogen receptor-alpha with members of the forkhead transcription factor family. J Biol Chem 2001;276:33554-33560.
1269. An BS, Tavera-Mendoza LE, Dimitrov V, et al. Stimulation of Sirt1-regulated FoxO protein function by the ligand-bound vitamin D receptor. Mol Cell Biol 2010;30:4890-4900.
1270. Albers M, Kranz H, Kober I, et al. Automated yeast two-hybrid screening for nuclear receptor-interacting proteins. Mol Cell Proteomics 2005;4:205-213.
1271. Jiang F, Li P, Fornace AJ, Jr., Nicosia SV, Bai W. G2/M arrest by 1,25-dihydroxyvitamin D3 in ovarian cancer cells mediated through the induction of GADD45 via an exonic enhancer. J Biol Chem 2003;278:48030-48040.
1272. Khandanpour C, Phelan JD, Vassen L, et al. Growth factor independence 1 antagonizes a p53-induced DNA damage response pathway in lymphoblastic leukemia. Cancer Cell 2013;23:200-214.
1273. Jiang YJ, Teichert AE, Fong F, Oda Y, Bikle DD. 1alpha,25(OH)2-dihydroxyvitamin D3/VDR protects the skin from UVB-induced tumor formation by interacting with the beta-catenin pathway. J Steroid Biochem Mol Biol 2013;136:229-232.
1274. Regl G, Kasper M, Schnidar H, et al. The zinc-finger transcription factor GLI2 antagonizes contact inhibition and differentiation of human epidermal cells. Oncogene 2004;23:1263-1274.
1275. Ismail A, Nguyen CV, Ahene A, Fleet JC, Uskokovic MR, Peleg S. Effect of cellular environment on the selective activation of the vitamin D receptor by 1alpha,25-dihydroxyvitamin D3 and its analog 1alpha-fluoro-16-ene-20-epi-23-ene-26,27-bishomo-25-hydroxyvitamin D3 (Ro-26-9228). Mol Endocrinol 2004;18:874-887.
1276. Han S, Li T, Ellis E, Strom S, Chiang JY. A novel bile acid-activated vitamin D receptor signaling in human hepatocytes. Mol Endocrinol 2010;24:1151-1164.
1277. Bjorkhem I, Leoni V, Meaney S. Genetic connections between neurological disorders and cholesterol metabolism. J Lipid Res 2010;51:2489-2503.
1278. Szeto FL, Reardon CA, Yoon D, et al. Vitamin D receptor signaling inhibits atherosclerosis in mice. Mol Endocrinol 2012;26:1091-1101.
1279. Ewing AK, Attner M, Chakravarti D. Novel regulatory role for human Acf1 in transcriptional repression of vitamin D3 receptor-regulated genes. Mol Endocrinol 2007;21:1791-1806.
1280. Yu S, Zhao J, Cantorna MT. Invariant NKT cell defects in vitamin D receptor knockout mice prevents experimental lung inflammation. J Immunol 2011;187:4907-4912.
1281. Liu NQ, Kaplan AT, Lagishetty V, et al. Vitamin D and the regulation of placental inflammation. J Immunol 2011;186:5968-5974.
1282. De Bosscher K, Vanden Berghe W, Haegeman G. The interplay between the glucocorticoid receptor and nuclear factor-kappaB or activator protein-1: molecular mechanisms for gene repression. Endocr Rev 2003;24:488-522.
1283. Chan SW, Hong W. Retinoblastoma-binding protein 2 (Rbp2) potentiates nuclear hormone receptor-mediated transcription. J Biol Chem 2001;276:28402-28412.

1284. Kaneko I, Sabir MS, Dussik CM, et al. 1,25-Dihydroxyvitamin D regulates expression of the tryptophan hydroxylase 2 and leptin genes: implication for behavioral influences of vitamin D. FASEB J 2015;29:4023-4035.
1285. Heyne K, Heil TC, Bette B, Reichrath J, Roemer K. MDM2 binds and inhibits vitamin D receptor. Cell Cycle 2015;14:2003-2010.
1286. Rachez C, Lemon BD, Suldan Z, et al. Ligand-dependent transcription activation by nuclear receptors requires the DRIP complex. Nature 1999;398:824-828.
1287. Salehi-Tabar R, Nguyen-Yamamoto L, Tavera-Mendoza LE, et al. Vitamin D receptor as a master regulator of the c-MYC/MXD1 network. Proc Natl Acad Sci U S A 2012;109:18827-18832.
1288. Ellison TI, Dowd DR, MacDonald PN. Calmodulin-dependent kinase IV stimulates vitamin D receptor-mediated transcription. Mol Endocrinol 2005;19:2309-2319.
1289. Ting HJ, Bao BY, Hsu CL, Lee YF. Androgen-receptor coregulators mediate the suppressive effect of androgen signals on vitamin D receptor activity. Endocrine 2005;26:1-9.
1290. Sauve F, McBroom LD, Gallant J, Moraitis AN, Labrie F, Giguere V. CIA, a novel estrogen receptor coactivator with a bifunctional nuclear receptor interacting determinant. Mol Cell Biol 2001;21:343-353.
1291. Mahajan MA, Samuels HH. A new family of nuclear receptor coregulators that integrate nuclear receptor signaling through CREB-binding protein. Mol Cell Biol 2000;20:5048-5063.
1292. Puccetti E, Obradovic D, Beissert T, et al. AML-associated translocation products block vitamin D(3)-induced differentiation by sequestering the vitamin D(3) receptor. Cancer Res 2002;62:7050-7058.
1293. Wang XX, Jiang T, Shen Y, et al. Vitamin D receptor agonist doxercalciferol modulates dietary fat-induced renal disease and renal lipid metabolism. Am J Physiol Renal Physiol 2011;300:F801-810.
1294. Marshall PA, Hernandez Z, Kaneko I, et al. Discovery of novel vitamin D receptor interacting proteins that modulate 1,25-dihydroxyvitamin D3 signaling. J Steroid Biochem Mol Biol 2012;132:147-159.
1295. Sequeira VB, Rybchyn MS, Tongkao-On W, et al. The role of the vitamin D receptor and ERp57 in photoprotection by 1alpha,25-dihydroxyvitamin D3. Mol Endocrinol 2012;26:574-582.
1296. von Essen MR, Kongsbak M, Schjerling P, Olgaard K, Odum N, Geisler C. Vitamin D controls T cell antigen receptor signaling and activation of human T cells. Nat Immunol 2010;11:344-349.
1297. Alimirah F, Peng X, Yuan L, et al. Crosstalk between the peroxisome proliferator-activated receptor gamma (PPARgamma) and the vitamin D receptor (VDR) in human breast cancer cells: PPARgamma binds to VDR and inhibits 1alpha,25-dihydroxyvitamin D3 mediated transactivation. Exp Cell Res 2012;318:2490-2497.
1298. Wang Q, He Y, Shen Y, et al. Vitamin D inhibits COX-2 expression and inflammatory response by targeting thioesterase superfamily member 4. J Biol Chem 2014;289:11681-11694.
1299. Zhi HY, Hou SW, Li RS, et al. PTPH1 cooperates with vitamin D receptor to stimulate breast cancer growth through their mutual stabilization. Oncogene 2011;30:1706-1715.
1300. Attar PS, Wertz PW, McArthur M, Imakado S, Bickenbach JR, Roop DR. Inhibition of retinoid signaling in transgenic mice alters lipid processing and disrupts epidermal barrier function. Mol Endocrinol 1997;11:792-800.
1301. Yuan W, Pan W, Kong J, et al. 1,25-dihydroxyvitamin D3 suppresses renin gene transcription by blocking the activity of the cyclic AMP response element in the renin gene promoter. J Biol Chem 2007;282:29821-29830.
1302. Marcellini S, Bruna C, Henriquez JP, et al. Evolution of the interaction between Runx2 and VDR, two transcription factors involved in osteoblastogenesis. BMC Evol Biol 2010;10:78.
1303. Subramaniam N, Leong GM, Cock TA, et al. Cross-talk between 1,25-dihydroxyvitamin D3 and transforming growth factor-beta signaling requires binding of VDR and Smad3 proteins to their cognate DNA recognition elements. J Biol Chem 2001;276:15741-15746.

1304. Baudino TA, Kraichely DM, Jefcoat SC, Jr., Winchester SK, Partridge NC, MacDonald PN. Isolation and characterization of a novel coactivator protein, NCoA-62, involved in vitamin D-mediated transcription. J Biol Chem 1998;273:16434-16441.
1305. Vidal M, Ramana CV, Dusso AS. Stat1-vitamin D receptor interactions antagonize 1,25-dihydroxyvitamin D transcriptional activity and enhance stat1-mediated transcription. Mol Cell Biol 2002;22:2777-2787.
1306. O'Kelly J, Hisatake J, Hisatake Y, Bishop J, Norman A, Koeffler HP. Normal myelopoiesis but abnormal T lymphocyte responses in vitamin D receptor knockout mice. J Clin Invest 2002;109:1091-1099.
1307. Lavigne AC, Mengus G, Gangloff YG, Wurtz JM, Davidson I. Human TAF(II)55 interacts with the vitamin D(3) and thyroid hormone receptors and with derivatives of the retinoid X receptor that have altered transactivation properties. Mol Cell Biol 1999;19:5486-5494.
1308. Endo I, Inoue D, Mitsui T, et al. Deletion of vitamin D receptor gene in mice results in abnormal skeletal muscle development with deregulated expression of myoregulatory transcription factors. Endocrinology 2003;144:5138-5144.
1309. Jiang F, Bao J, Li P, Nicosia SV, Bai W. Induction of ovarian cancer cell apoptosis by 1,25-dihydroxyvitamin D3 through the down-regulation of telomerase. J Biol Chem 2004;279:53213-53221.
1310. Stambolsky P, Tabach Y, Fontemaggi G, et al. Modulation of the vitamin D3 response by cancer-associated mutant p53. Cancer Cell 2010;17:273-285.
1311. Ito M, Yuan CX, Malik S, et al. Identity between TRAP and SMCC complexes indicates novel pathways for the function of nuclear receptors and diverse mammalian activators. Mol Cell 1999;3:361-370.
1312. Barletta F, Freedman LP, Christakos S. Enhancement of VDR-mediated transcription by phosphorylation: correlation with increased interaction between the VDR and DRIP205, a subunit of the VDR-interacting protein coactivator complex. Mol Endocrinol 2002;16:301-314.
1313. Hill NT, Zhang J, Leonard MK, Lee M, Shamma HN, Kadakia M. 1alpha, 25-Dihydroxyvitamin D(3) and the vitamin D receptor regulates DeltaNp63alpha levels and keratinocyte proliferation. Cell Death Dis 2015;6:e1781.
1314. Maruyama R, Aoki F, Toyota M, et al. Comparative genome analysis identifies the vitamin D receptor gene as a direct target of p53-mediated transcriptional activation. Cancer Res 2006;66:4574-4583.
1315. Le Douarin B, Nielsen AL, Garnier JM, et al. A possible involvement of TIF1 alpha and TIF1 beta in the epigenetic control of transcription by nuclear receptors. EMBO J 1996;15:6701-6715.
1316. Shaffer PL, Gewirth DT. Structural basis of VDR-DNA interactions on direct repeat response elements. EMBO J 2002;21:2242-2252.
1317. Lee TH, Pelletier J. Functional characterization of WT1 binding sites within the human vitamin D receptor gene promoter. Physiol Genomics 2001;7:187-200.
1318. Bhattacharjee G, Asplin IR, Wu SM, Gawdi G, Pizzo SV. The conformation-dependent interaction of alpha 2-macroglobulin with vascular endothelial growth factor. A novel mechanism of alpha 2-macroglobulin/growth factor binding. J Biol Chem 2000;275:26806-26811.
1319. Ichihara S, Yamada Y, Ichihara G, et al. A role for the aryl hydrocarbon receptor in regulation of ischemia-induced angiogenesis. Arterioscler Thromb Vasc Biol 2007;27:1297-1304.
1320. Roybal CN, Hunsaker LA, Barbash O, Vander Jagt DL, Abcouwer SF. The oxidative stressor arsenite activates vascular endothelial growth factor mRNA transcription by an ATF4-dependent mechanism. J Biol Chem 2005;280:20331-20339.
1321. Cvetanovic M, Patel JM, Marti HH, Kini AR, Opal P. Vascular endothelial growth factor ameliorates the ataxic phenotype in a mouse model of spinocerebellar ataxia type 1. Nat Med 2011;17:1445-1447.
1322. Kollmann K, Heller G, Schneckenleithner C, et al. A kinase-independent function of CDK6 links the cell cycle to tumor angiogenesis. Cancer Cell 2013;24:167-181.

1323. Ruan Q, Han S, Jiang WG, et al. alphaB-crystallin, an effector of unfolded protein response, confers anti-VEGF resistance to breast cancer via maintenance of intracrine VEGF in endothelial cells. Mol Cancer Res 2011;9:1632-1643.
1324. Worden B, Yang XP, Lee TL, et al. Hepatocyte growth factor/scatter factor differentially regulates expression of proangiogenic factors through Egr-1 in head and neck squamous cell carcinoma. Cancer Res 2005;65:7071-7080.
1325. Luo W, Hu H, Chang R, et al. Pyruvate kinase M2 is a PHD3-stimulated coactivator for hypoxia-inducible factor 1. Cell 2011;145:732-744.
1326. Valter MM, Hugel A, Huang HJ, et al. Expression of the Ets-1 transcription factor in human astrocytomas is associated with Fms-like tyrosine kinase-1 (Flt-1)/vascular endothelial growth factor receptor-1 synthesis and neoangiogenesis. Cancer Res 1999;59:5608-5614.
1327. Bassus S, Herkert O, Kronemann N, et al. Thrombin causes vascular endothelial growth factor expression in vascular smooth muscle cells: role of reactive oxygen species. Arterioscler Thromb Vasc Biol 2001;21:1550-1555.
1328. Wang Z, Banerjee S, Kong D, Li Y, Sarkar FH. Down-regulation of Forkhead Box M1 transcription factor leads to the inhibition of invasion and angiogenesis of pancreatic cancer cells. Cancer Res 2007;67:8293-8300.
1329. Tang TT, Lasky LA. The forkhead transcription factor FOXO4 induces the down-regulation of hypoxia-inducible factor 1 alpha by a von Hippel-Lindau protein-independent mechanism. J Biol Chem 2003;278:30125-30135.
1330. Nakahira K, Morita A, Kim NS, Yanagihara I. Phosphorylation of FOXP3 by LCK downregulates MMP9 expression and represses cell invasion. PLoS One 2013;8:e77099.
1331. Zhu H, Carpenter RL, Han W, Lo HW. The GLI1 splice variant TGLI1 promotes glioblastoma angiogenesis and growth. Cancer Lett 2014;343:51-61.
1332. Sun X, Wei L, Chen Q, Terek RM. HDAC4 represses vascular endothelial growth factor expression in chondrosarcoma by modulating RUNX2 activity. J Biol Chem 2009;284:21881-21890.
1333. Shang Y, Doan CN, Arnold TD, et al. Transcriptional corepressors HIPK1 and HIPK2 control angiogenesis via TGF-beta-TAK1-dependent mechanism. PLoS Biol 2013;11:e1001527.
1334. Kishor A, Tandukar B, Ly YV, et al. Hsp70 is a novel posttranscriptional regulator of gene expression that binds and stabilizes selected mRNAs containing AU-rich elements. Mol Cell Biol 2013;33:71-84.
1335. Park JH, Kim SH, Choi MC, et al. Class II histone deacetylases play pivotal roles in heat shock protein 90-mediated proteasomal degradation of vascular endothelial growth factor receptors. Biochem Biophys Res Commun 2008;368:318-322.
1336. Ozawa K, Tsukamoto Y, Hori O, et al. Regulation of tumor angiogenesis by oxygen-regulated protein 150, an inducible endoplasmic reticulum chaperone. Cancer Res 2001;61:4206-4213.
1337. Gerald D, Berra E, Frapart YM, et al. JunD reduces tumor angiogenesis by protecting cells from oxidative stress. Cell 2004;118:781-794.
1338. Luque A, Carpizo DR, Iruela-Arispe ML. ADAMTS1/METH1 inhibits endothelial cell proliferation by direct binding and sequestration of VEGF165. J Biol Chem 2003;278:23656-23665.
1339. Lapchak PH, Melter M, Pal S, et al. CD40-induced transcriptional activation of vascular endothelial growth factor involves a 68-bp region of the promoter containing a CpG island. Am J Physiol Renal Physiol 2004;287:F512-520.
1340. Braun S, Hanselmann C, Gassmann MG, et al. Nrf2 transcription factor, a novel target of keratinocyte growth factor action which regulates gene expression and inflammation in the healing skin wound. Mol Cell Biol 2002;22:5492-5505.
1341. Walczak R, Joseph SB, Laffitte BA, Castrillo A, Pei L, Tontonoz P. Transcription of the vascular endothelial growth factor gene in macrophages is regulated by liver X receptors. J Biol Chem 2004;279:9905-9911.

1342. Ball SG, Shuttleworth CA, Kielty CM. Vascular endothelial growth factor can signal through platelet-derived growth factor receptors. J Cell Biol 2007;177:489-500.
1343. Sun K, Wernstedt Asterholm I, Kusminski CM, et al. Dichotomous effects of VEGF-A on adipose tissue dysfunction. Proc Natl Acad Sci U S A 2012;109:5874-5879.
1344. Ohnuki H, Inoue H, Takemori N, et al. BAZF, a novel component of cullin3-based E3 ligase complex, mediates VEGFR and Notch cross-signaling in angiogenesis. Blood 2012;119:2688-2698.
1345. Ter Elst A, Ma B, Scherpen FJ, et al. Repression of vascular endothelial growth factor expression by the runt-related transcription factor 1 in acute myeloid leukemia. Cancer Res 2011;71:2761-2771.
1346. Tripurani SK, Cook RW, Eldin KW, Pangas SA. BMP-specific SMADs function as novel repressors of PDGFA and modulate its expression in ovarian granulosa cells and tumors. Oncogene 2013;32:3877-3885.
1347. Sanchez-Elsner T, Botella LM, Velasco B, Corbi A, Attisano L, Bernabeu C. Synergistic cooperation between hypoxia and transforming growth factor-beta pathways on human vascular endothelial growth factor gene expression. J Biol Chem 2001;276:38527-38535.
1348. Shi Q, Le X, Abbruzzese JL, et al. Constitutive Sp1 activity is essential for differential constitutive expression of vascular endothelial growth factor in human pancreatic adenocarcinoma. Cancer Res 2001;61:4143-4154.
1349. Sun L, Halaihel N, Zhang W, Rogers T, Levi M. Role of sterol regulatory element-binding protein 1 in regulation of renal lipid metabolism and glomerulosclerosis in diabetes mellitus. J Biol Chem 2002;277:18919-18927.
1350. Steingrimsson E, Tessarollo L, Reid SW, Jenkins NA, Copeland NG. The bHLH-Zip transcription factor Tfeb is essential for placental vascularization. Development 1998;125:4607-4616.
1351. Hamdollah Zadeh MA, Amin EM, Hoareau-Aveilla C, et al. Alternative splicing of TIA-1 in human colon cancer regulates VEGF isoform expression, angiogenesis, tumour growth and bevacizumab resistance. Mol Oncol 2015;9:167-178.
1352. Senoo M, Matsumura Y, Habu S. TAp63gamma (p51A) and dNp63alpha (p73L), two major isoforms of the p63 gene, exert opposite effects on the vascular endothelial growth factor (VEGF) gene expression. Oncogene 2002;21:2455-2465.
1353. Dulloo I, Phang BH, Othman R, et al. Hypoxia-inducible TAp73 supports tumorigenesis by regulating the angiogenic transcriptome. Nat Cell Biol 2015;17:511-523.
1354. Torres-Padilla ME, Zernicka-Goetz M. Role of TIF1alpha as a modulator of embryonic transcription in the mouse zygote. J Cell Biol 2006;174:329-338.
1355. Li Z, Wang D, Na X, Schoen SR, Messing EM, Wu G. The VHL protein recruits a novel KRAB-A domain protein to repress HIF-1alpha transcriptional activity. EMBO J 2003;22:1857-1867.
1356. McCarty G, Awad O, Loeb DM. WT1 protein directly regulates expression of vascular endothelial growth factor and is a mediator of tumor response to hypoxia. J Biol Chem 2011;286:43634-43643.
1357. Hendrickx A, Beullens M, Ceulemans H, et al. Docking motif-guided mapping of the interactome of protein phosphatase-1. Chem Biol 2009;16:365-371.
1358. Perez-Victoria FJ, Schindler C, Magadan JG, et al. Ang2/fat-free is a conserved subunit of the Golgi-associated retrograde protein complex. Mol Biol Cell 2010;21:3386-3395.
1359. Shin JH, Ko HS, Kang H, et al. PARIS (ZNF746) repression of PGC-1alpha contributes to neurodegeneration in Parkinson's disease. Cell 2011;144:689-702.
1360. Steuble M, Gerrits B, Ludwig A, et al. Molecular characterization of a trafficking organelle: dissecting the axonal paths of calsyntenin-1 transport vesicles. Proteomics 2010;10:3775-3788.
1361. Lapierre LA, Avant KM, Caldwell CM, et al. Characterization of immunoisolated human gastric parietal cells tubulovesicles: identification of regulators of apical recycling. Am J Physiol Gastrointest Liver Physiol 2007;292:G1249-1262.
1362. Huang L, Kuo YM, Gitschier J. The pallid gene encodes a novel, syntaxin 13-interacting protein involved in platelet storage pool deficiency. Nat Genet 1999;23:329-332.

1363. McBride HM, Rybin V, Murphy C, Giner A, Teasdale R, Zerial M. Oligomeric complexes link Rab5 effectors with NSF and drive membrane fusion via interactions between EEA1 and syntaxin 13. Cell 1999;98:377-386.
1364. Bethani I, Lang T, Geumann U, Sieber JJ, Jahn R, Rizzoli SO. The specificity of SNARE pairing in biological membranes is mediated by both proof-reading and spatial segregation. EMBO J 2007;26:3981-3992.
1365. Izumi S, Saito A, Kanemoto S, et al. The endoplasmic reticulum stress transducer BBF2H7 suppresses apoptosis by activating the ATF5-MCL1 pathway in growth plate cartilage. J Biol Chem 2012;287:36190-36200.
1366. Saito A, Kanemoto S, Zhang Y, Asada R, Hino K, Imaizumi K. Chondrocyte proliferation regulated by secreted luminal domain of ER stress transducer BBF2H7/CREB3L2. Mol Cell 2014;53:127-139.
1367. Liu J, Yao F, Wu R, et al. Mediation of the DCC apoptotic signal by DIP13 alpha. J Biol Chem 2002;277:26281-26285.
1368. Mehlen P, Rabizadeh S, Snipas SJ, Assa-Munt N, Salvesen GS, Bredesen DE. The DCC gene product induces apoptosis by a mechanism requiring receptor proteolysis. Nature 1998;395:801-804.
1369. Forcet C, Ye X, Granger L, et al. The dependence receptor DCC (deleted in colorectal cancer) defines an alternative mechanism for caspase activation. Proc Natl Acad Sci U S A 2001;98:3416-3421.
1370. Ly A, Nikolaev A, Suresh G, Zheng Y, Tessier-Lavigne M, Stein E. DSCAM is a netrin receptor that collaborates with DCC in mediating turning responses to netrin-1. Cell 2008;133:1241-1254.
1371. Tcherkezian J, Brittis PA, Thomas F, Roux PP, Flanagan JG. Transmembrane receptor DCC associates with protein synthesis machinery and regulates translation. Cell 2010;141:632-644.
1372. Li W, Lee J, Vikis HG, et al. Activation of FAK and Src are receptor-proximal events required for netrin signaling. Nat Neurosci 2004;7:1213-1221.
1373. Bull SJ, Bin JM, Beaumont E, et al. Progressive disorganization of paranodal junctions and compact myelin due to loss of DCC expression by oligodendrocytes. J Neurosci 2014;34:9768-9778.
1374. Deming PB, Campbell SL, Stone JB, Rivard RL, Mercier AL, Howe AK. Anchoring of protein kinase A by ERM (ezrin-radixin-moesin) proteins is required for proper netrin signaling through DCC (deleted in colorectal cancer). J Biol Chem 2015;290:5783-5796.
1375. Qi Q, Li DY, Luo HR, Guan KL, Ye K. Netrin-1 exerts oncogenic activities through enhancing Yes-associated protein stability. Proc Natl Acad Sci U S A 2015;112:7255-7260.
1376. Arroyo R, Duran-Frigola M, Berenguer C, Soler-Lopez M, Aloy P. Charting the molecular links between driver and susceptibility genes in colorectal cancer. Biochem Biophys Res Commun 2014;445:734-738.
1377. Hu G, Zhang S, Vidal M, Baer JL, Xu T, Fearon ER. Mammalian homologs of seven in absentia regulate DCC via the ubiquitin-proteasome pathway. Genes Dev 1997;11:2701-2714.
1378. Qu C, Dwyer T, Shao Q, Yang T, Huang H, Liu G. Direct binding of TUBB3 with DCC couples netrin-1 signaling to intracellular microtubule dynamics in axon outgrowth and guidance. J Cell Sci 2013;126:3070-3081.
1379. Kamal A, Stokin GB, Yang Z, Xia CH, Goldstein LS. Axonal transport of amyloid precursor protein is mediated by direct binding to the kinesin light chain subunit of kinesin-I. Neuron 2000;28:449-459.
1380. Kim JY, Shen S, Dietz K, et al. HDAC1 nuclear export induced by pathological conditions is essential for the onset of axonal damage. Nat Neurosci 2010;13:180-189.
1381. Nakajima K, Yin X, Takei Y, Seog DH, Homma N, Hirokawa N. Molecular motor KIF5A is essential for GABA(A) receptor transport, and KIF5A deletion causes epilepsy. Neuron 2012;76:945-961.
1382. Mandal M, Wei J, Zhong P, et al. Impaired alpha-amino-3-hydroxy-5-methyl-4-isoxazolepropionic acid (AMPA) receptor trafficking and function by mutant huntingtin. J Biol Chem 2011;286:33719-33728.
1383. Boutchueng-Djidjou M, Collard-Simard G, Fortier S, et al. The last enzyme of the de novo purine synthesis pathway 5-aminoimidazole-4-carboxamide ribonucleotide formyltransferase/IMP

cyclohydrolase (ATIC) plays a central role in insulin signaling and the Golgi/endosomes protein network. Mol Cell Proteomics 2015;14:1079-1092.
1384. Kanai Y, Okada Y, Tanaka Y, Harada A, Terada S, Hirokawa N. KIF5C, a novel neuronal kinesin enriched in motor neurons. J Neurosci 2000;20:6374-6384.
1385. Rahman A, Kamal A, Roberts EA, Goldstein LS. Defective kinesin heavy chain behavior in mouse kinesin light chain mutants. J Cell Biol 1999;146:1277-1288.
1386. Setou M, Seog DH, Tanaka Y, et al. Glutamate-receptor-interacting protein GRIP1 directly steers kinesin to dendrites. Nature 2002;417:83-87.
1387. Brendel C, Rehbein M, Kreienkamp HJ, Buck F, Richter D, Kindler S. Characterization of Staufen 1 ribonucleoprotein complexes. Biochem J 2004;384:239-246.
1388. Amit I, Yakir L, Katz M, et al. Tal, a Tsg101-specific E3 ubiquitin ligase, regulates receptor endocytosis and retrovirus budding. Genes Dev 2004;18:1737-1752.
1389. Matsuzaki F, Shirane M, Matsumoto M, Nakayama KI. Protrudin serves as an adaptor molecule that connects KIF5 and its cargoes in vesicular transport during process formation. Mol Biol Cell 2011;22:4602-4620.
1390. Sirvent A, Benistant C, Roche S. Oncogenic signaling by tyrosine kinases of the SRC family in advanced colorectal cancer. Am J Cancer Res 2012;2:357-371.
1391. Qiu XB, Goldberg AL. The membrane-associated inhibitor of apoptosis protein, BRUCE/Apollon, antagonizes both the precursor and mature forms of Smac and caspase-9. J Biol Chem 2005;280:174-182.
1392. Kikuchi R, Ohata H, Ohoka N, Kawabata A, Naito M. APOLLON protein promotes early mitotic CYCLIN A degradation independent of the spindle assembly checkpoint. J Biol Chem 2014;289:3457-3467.
1393. Zhang M, Han G, Wang C, et al. A bead-based approach for large-scale identification of in vitro kinase substrates. Proteomics 2011;11:4632-4637.
1394. Hao Y, Sekine K, Kawabata A, et al. Apollon ubiquitinates SMAC and caspase-9, and has an essential cytoprotection function. Nat Cell Biol 2004;6:849-860.
1395. Pohl C, Jentsch S. Final stages of cytokinesis and midbody ring formation are controlled by BRUCE. Cell 2008;132:832-845.
1396. Tang W, Xue R, Weng S, et al. BIRC6 promotes hepatocellular carcinogenesis: interaction of BIRC6 with p53 facilitating p53 degradation. Int J Cancer 2015;136:E475-487.
1397. Aoki T, Ichimura S, Itoh A, et al. Identification of the neuroblastoma-amplified gene product as a component of the syntaxin 18 complex implicated in Golgi-to-endoplasmic reticulum retrograde transport. Mol Biol Cell 2009;20:2639-2649.
1398. Shorter J, Beard MB, Seemann J, Dirac-Svejstrup AB, Warren G. Sequential tethering of Golgins and catalysis of SNAREpin assembly by the vesicle-tethering protein p115. J Cell Biol 2002;157:45-62.
1399. Reynard LN, Cocquet J, Burgoyne PS. The multi-copy mouse gene Sycp3-like Y-linked (Sly) encodes an abundant spermatid protein that interacts with a histone acetyltransferase and an acrosomal protein. Biol Reprod 2009;81:250-257.
1400. Dachsel JC, Taylor JP, Mok SS, et al. Identification of potential protein interactors of Lrrk2. Parkinsonism Relat Disord 2007;13:382-385.
1401. Liu L, Rodriguez-Belmonte EM, Mazloum N, Xie B, Lee MY. Identification of a novel protein, PDIP38, that interacts with the p50 subunit of DNA polymerase delta and proliferating cell nuclear antigen. J Biol Chem 2003;278:10041-10047.
1402. Furge LL, Chen K, Cohen S. Annexin VII and annexin XI are tyrosine phosphorylated in peroxovanadate-treated dogs and in platelet-derived growth factor-treated rat vascular smooth muscle cells. J Biol Chem 1999;274:33504-33509.
1403. Tomas A, Futter C, Moss SE. Annexin 11 is required for midbody formation and completion of the terminal phase of cytokinesis. J Cell Biol 2004;165:813-822.

1404. Sahni N, Yi S, Taipale M, et al. Widespread macromolecular interaction perturbations in human genetic disorders. Cell 2015;161:647-660.
1405. Butler R, Bates GP. Histone deacetylase inhibitors as therapeutics for polyglutamine disorders. Nat Rev Neurosci 2006;7:784-796.
1406. Suter B, Fontaine JF, Yildirimman R, et al. Development and application of a DNA microarray-based yeast two-hybrid system. Nucleic Acids Res 2013;41:1496-1507.
1407. Bolger TA, Zhao X, Cohen TJ, Tsai CC, Yao TP. The neurodegenerative disease protein ataxin-1 antagonizes the neuronal survival function of myocyte enhancer factor-2. J Biol Chem 2007;282:29186-29192.
1408. Choi JY, Ryu JH, Kim HS, et al. Co-chaperone CHIP promotes aggregation of ataxin-1. Mol Cell Neurosci 2007;34:69-79.
1409. Serra HG, Duvick L, Zu T, et al. RORalpha-mediated Purkinje cell development determines disease severity in adult SCA1 mice. Cell 2006;127:697-708.
1410. Mizutani A, Wang L, Rajan H, et al. Boat, an AXH domain protein, suppresses the cytotoxicity of mutant ataxin-1. EMBO J 2005;24:3339-3351.
1411. Guo L, Giasson BI, Glavis-Bloom A, et al. A cellular system that degrades misfolded proteins and protects against neurodegeneration. Mol Cell 2014;55:15-30.
1412. Tong X, Gui H, Jin F, et al. Ataxin-1 and Brother of ataxin-1 are components of the Notch signalling pathway. EMBO Rep 2011;12:428-435.
1413. Kang S, Hong S. SUMO-1 interacts with mutant ataxin-1 and colocalizes to its aggregates in Purkinje cells of SCA1 transgenic mice. Arch Ital Biol 2010;148:351-363.
1414. Huber MD, Vesely PW, Datta K, Gerace L. Erlins restrict SREBP activation in the ER and regulate cellular cholesterol homeostasis. J Cell Biol 2013;203:427-436.
1415. Pearce MM, Wormer DB, Wilkens S, Wojcikiewicz RJ. An endoplasmic reticulum (ER) membrane complex composed of SPFH1 and SPFH2 mediates the ER-associated degradation of inositol 1,4,5-trisphosphate receptors. J Biol Chem 2009;284:10433-10445.
1416. Tsai NP, Ho PC, Wei LN. Regulation of stress granule dynamics by Grb7 and FAK signalling pathway. EMBO J 2008;27:715-726.
1417. Yu C, York B, Wang S, Feng Q, Xu J, O'Malley BW. An essential function of the SRC-3 coactivator in suppression of cytokine mRNA translation and inflammatory response. Mol Cell 2007;25:765-778.
1418. Delestienne N, Wauquier C, Soin R, Dierick JF, Gueydan C, Kruys V. The splicing factor ASF/SF2 is associated with TIA-1-related/TIA-1-containing ribonucleoproteic complexes and contributes to post-transcriptional repression of gene expression. FEBS J 2010;277:2496-2514.
1419. Pullmann R, Jr., Kim HH, Abdelmohsen K, et al. Analysis of turnover and translation regulatory RNA-binding protein expression through binding to cognate mRNAs. Mol Cell Biol 2007;27:6265-6278.

Supplementary Table 3

The list of all ALS proteins and the number of their interaction with other ALS related gene products. The table includes gene ID, Entrez Gene name, their total number of protein interactions, location of the protein and the protein type.

ID	Entrez Gene Name	Total binding partner	Location	Type(s)
A2M	alpha-2-macroglobulin	4	Extracellular Space	transporter
ABCA1	ATP binding cassette subfamily A member 1	4	Plasma Membrane	transporter
ABCE1	ATP binding cassette subfamily E member 1	4	Cytoplasm	transporter
ABHD5	abhydrolase domain containing 5	3	Cytoplasm	enzyme
ABL1	ABL proto-oncogene 1, non-receptor tyrosine kinase	6	Nucleus	kinase
ABL2	ABL proto-oncogene 2, non-receptor tyrosine kinase	3	Cytoplasm	kinase
ACACA	acetyl-CoA carboxylase alpha	5	Cytoplasm	enzyme
ACAN	aggrecan	3	Extracellular Space	other
ACTA1	actin, alpha 1, skeletal muscle	4	Cytoplasm	other
ACTB	actin beta	13	Cytoplasm	other
ACTG1	actin gamma 1	4	Cytoplasm	other
ACTN1	actinin alpha 1	5	Cytoplasm	transcription regulator
ACTN2	actinin alpha 2	5	Nucleus	transcription regulator
ADAR	adenosine deaminase, RNA specific	3	Nucleus	enzyme
ADRB2	adrenoceptor beta 2	9	Plasma Membrane	G-protein coupled receptor
AES	amino-terminal enhancer of split	3	Nucleus	transcription regulator
AFDN	afadin, adherens junction formation factor	3	Nucleus	other
AGO1	argonaute 1, RISC catalytic component	3	Cytoplasm	translation regulator
AGO2	argonaute 2, RISC catalytic component	5	Cytoplasm	translation regulator
AGT	angiotensinogen	3	Extracellular Space	growth factor
AHR	aryl hydrocarbon receptor	4	Nucleus	lig-dependent nuc. receptor
AHSA1	activator of HSP90 ATPase activity 1	3	Cytoplasm	other
AIMP2	aminoacyl tRNA synthetase complex interacting multifunctional protein 2	3	Plasma Membrane	other
AK1	adenylate kinase 1	3	Cytoplasm	kinase
AKAP8	A-kinase anchoring protein 8	3	Nucleus	other
AKT1	AKT serine/threonine kinase 1	7	Cytoplasm	kinase
ALB	albumin	9	Extracellular Space	transporter
ALDOA	aldolase, fructose-bisphosphate A	3	Cytoplasm	enzyme
ALS2	ALS2, alsin Rho guanine nucleotide exchange factor	4	Cytoplasm	other
ALYREF	Aly/REF export factor	3	Nucleus	transcription regulator
AMFR	autocrine motility factor receptor	3	Plasma Membrane	transmembrane receptor
ANK2	ankyrin 2	5	Plasma Membrane	other
ANKHD1	ankyrin repeat and KH domain containing 1	3	Other	other
ANKLE2	ankyrin repeat and LEM domain containing 2	3	Nucleus	transcription regulator
ANP32A	acidic nuclear phosphoprotein 32 family member A	3	Other	other
ANXA1	annexin A1	3	Plasma Membrane	enzyme
ANXA2	annexin A2	4	Plasma Membrane	other
ANXA5	annexin A5	3	Plasma Membrane	transporter
APC	APC, WNT signaling pathway regulator	4	Nucleus	enzyme
APEX1	apurinic/apyrimidinic endodeoxyribonuclease 1	4	Nucleus	enzyme
APLP1	amyloid beta precursor like protein 1	4	Extracellular Space	other
APOA1	apolipoprotein A1	7	Extracellular Space	transporter
APOBEC3B	apolipoprotein B mRNA editing enzyme catalytic subunit 3B	4	Cytoplasm	enzyme
APOE	apolipoprotein E	5	Extracellular Space	transporter

APP	amyloid beta precursor protein	19	Plasma Membrane	other
APPL1	adaptor protein, phosphotyrosine interacting with PH domain and leucine zi	3	Cytoplasm	other
AR	androgen receptor	7	Nucleus	lig-dependent nuc. receptor
ARF6	ADP ribosylation factor 6	7	Plasma Membrane	transporter
ARFGAP1	ADP ribosylation factor GTPase activating protein 1	4	Cytoplasm	transporter
ARHGEF28	Rho guanine nucleotide exchange factor 28	5	Cytoplasm	other
ARID5A	AT-rich interaction domain 5A	4	Nucleus	transcription regulator
ARRB1	arrestin beta 1	3	Cytoplasm	other
ARRB2	arrestin beta 2	5	Cytoplasm	other
ASB2	ankyrin repeat and SOCS box containing 2	3	Nucleus	transcription regulator
ATF2	activating transcription factor 2	3	Nucleus	transcription regulator
ATF4	activating transcription factor 4	6	Nucleus	transcription regulator
ATF5	activating transcription factor 5	3	Nucleus	transcription regulator
ATG101	autophagy related 101	3	Cytoplasm	other
ATG13	autophagy related 13	3	Cytoplasm	other
ATG16L1	autophagy related 16 like 1	3	Cytoplasm	enzyme
ATG5	autophagy related 5	3	Cytoplasm	other
ATM	ATM serine/threonine kinase	5	Nucleus	kinase
ATN1	atrophin 1	7	Nucleus	transcription regulator
ATP1A3	ATPase Na+/K+ transporting subunit alpha 3	3	Plasma Membrane	transporter
ATP2A2	ATPase sarcoplasmic/endoplasmic reticulum Ca2+ transporting 2	4	Cytoplasm	transporter
ATP4A	ATPase H+/K+ transporting alpha subunit	3	Plasma Membrane	transporter
ATP5A1	ATP synthase F1 subunit alpha	6	Cytoplasm	transporter
ATP5B	ATP synthase F1 subunit beta	3	Cytoplasm	transporter
ATP6AP2	ATPase H+ transporting accessory protein 2	4	Cytoplasm	transporter
ATP6V0D1	ATPase H+ transporting V0 subunit d1	3	Cytoplasm	transporter
ATP6V1A	ATPase H+ transporting V1 subunit A	5	Plasma Membrane	transporter
ATRX	ATRX, chromatin remodeler	4	Nucleus	transcription regulator
ATXN1	ataxin 1	5	Nucleus	transcription regulator
ATXN2	ataxin 2	4	Nucleus	other
ATXN2L	ataxin 2 like	6	Nucleus	other
ATXN3	ataxin 3	3	Nucleus	peptidase
ATXN7	ataxin 7	5	Nucleus	peptidase
AURKA	aurora kinase A	6	Nucleus	kinase
BACE1	beta-secretase 1	3	Cytoplasm	peptidase
BAG1	BCL2 associated athanogene 1	5	Cytoplasm	other
BAG2	BCL2 associated athanogene 2	10	Cytoplasm	other
BAG3	BCL2 associated athanogene 3	7	Cytoplasm	other
BAG6	BCL2 associated athanogene 6	8	Nucleus	enzyme
BARD1	BRCA1 associated RING domain 1	3	Nucleus	transcription regulator
BAX	BCL2 associated X, apoptosis regulator	3	Cytoplasm	transporter
BCAR3	BCAR3, NSP family adaptor protein	3	Cytoplasm	other
BCL11B	B cell CLL/lymphoma 11B	4	Nucleus	transcription regulator
BCL2	BCL2, apoptosis regulator	6	Cytoplasm	transporter
BCL2L1	BCL2 like 1	4	Cytoplasm	other

BCL6	B cell CLL/lymphoma 6	3	Nucleus	transcription regulator
BCLAF1	BCL2 associated transcription factor 1	3	Nucleus	transcription regulator
BMI1	BMI1 proto-oncogene, polycomb ring finger	10	Nucleus	transcription regulator
BMPR1A	bone morphogenetic protein receptor type 1A	5	Plasma Membrane	kinase
BNIP1	BCL2 interacting protein 1	3	Cytoplasm	transporter
BRCA1	BRCA1, DNA repair associated	9	Nucleus	transcription regulator
BTG2	BTG anti-proliferation factor 2	3	Nucleus	transcription regulator
BTK	Bruton tyrosine kinase	3	Cytoplasm	kinase
BTRC	beta-transducin repeat containing E3 ubiquitin protein ligase	4	Cytoplasm	enzyme
BYSL	bystin like	4	Nucleus	other
C1QBP	complement C1q binding protein	6	Cytoplasm	transcription regulator
C21orf2	chromosome 21 open reading frame 2	4	Cytoplasm	other
C3	complement C3	3	Extracellular Space	peptidase
C9orf72	chromosome 9 open reading frame 72	3	Cytoplasm	other
CACNA1A	calcium voltage-gated channel subunit alpha1 A	9	Plasma Membrane	ion channel
CALM1	calmodulin 1	9	Cytoplasm	other
CALR	calreticulin	8	Cytoplasm	transcription regulator
CAMK2A	calcium/calmodulin dependent protein kinase II alpha	5	Cytoplasm	kinase
CAND1	cullin associated and neddylation dissociated 1	12	Cytoplasm	transcription regulator
CANX	calnexin	5	Cytoplasm	other
CAPZA2	capping actin protein of muscle Z-line alpha subunit 2	4	Cytoplasm	other
CARD8	caspase recruitment domain family member 8	3	Nucleus	other
CASP3	caspase 3	10	Cytoplasm	peptidase
CASP6	caspase 6	4	Cytoplasm	peptidase
CASP7	caspase 7	7	Cytoplasm	peptidase
CASP8	caspase 8	5	Nucleus	peptidase
CASP9	caspase 9	5	Cytoplasm	peptidase
CAV1	caveolin 1	5	Plasma Membrane	transmembrane receptor
CAV3	caveolin 3	3	Plasma Membrane	enzyme
CBL	Cbl proto-oncogene	4	Nucleus	transcription regulator
CCAR2	cell cycle and apoptosis regulator 2	3	Cytoplasm	peptidase
CCDC8	coiled-coil domain containing 8	11	Plasma Membrane	other
CCL2	C-C motif chemokine ligand 2	5	Extracellular Space	cytokine
CCNA2	cyclin A2	3	Nucleus	other
CCNB1	cyclin B1	7	Cytoplasm	kinase
CCND1	cyclin D1	6	Nucleus	transcription regulator
CCS	copper chaperone for superoxide dismutase	4	Cytoplasm	enzyme
CCT2	chaperonin containing TCP1 subunit 2	4	Cytoplasm	kinase
CCT3	chaperonin containing TCP1 subunit 3	7	Cytoplasm	other
CCT4	chaperonin containing TCP1 subunit 4	7	Cytoplasm	other
CCT5	chaperonin containing TCP1 subunit 5	7	Cytoplasm	other
CCT6A	chaperonin containing TCP1 subunit 6A	6	Cytoplasm	other
CCT7	chaperonin containing TCP1 subunit 7	4	Cytoplasm	other
CCT8	chaperonin containing TCP1 subunit 8	5	Cytoplasm	enzyme
CD2AP	CD2 associated protein	4	Cytoplasm	other

CD4	CD4 molecule	3	Plasma Membrane	transmembrane receptor
CD44	CD44 molecule (Indian blood group)	5	Plasma Membrane	other
CD81	CD81 molecule	3	Plasma Membrane	other
CDC37	cell division cycle 37	9	Cytoplasm	kinase
CDC42	cell division cycle 42	4	Cytoplasm	enzyme
CDC5L	cell division cycle 5 like	14	Nucleus	transcription regulator
CDH1	cadherin 1	4	Plasma Membrane	other
CDH2	cadherin 2	3	Plasma Membrane	other
CDK1	cyclin dependent kinase 1	10	Nucleus	kinase
CDK2	cyclin dependent kinase 2	10	Nucleus	kinase
CDK5	cyclin dependent kinase 5	6	Nucleus	kinase
CDK5R1	cyclin dependent kinase 5 regulatory subunit 1	3	Nucleus	kinase
CDK6	cyclin dependent kinase 6	4	Nucleus	kinase
CDK9	cyclin dependent kinase 9	5	Nucleus	kinase
CDKN1A	cyclin dependent kinase inhibitor 1A	6	Nucleus	kinase
CDKN2A	cyclin dependent kinase inhibitor 2A	3	Nucleus	transcription regulator
CEBPA	CCAAT/enhancer binding protein alpha	7	Nucleus	transcription regulator
CENPE	centromere protein E	6	Nucleus	other
CEP104	centrosomal protein 104	3	Cytoplasm	other
CEP170	centrosomal protein 170	3	Nucleus	other
CEP19	centrosomal protein 19	3	Cytoplasm	other
CEP290	centrosomal protein 290	7	Cytoplasm	transcription regulator
CEP55	centrosomal protein 55	4	Cytoplasm	other
CEP63	centrosomal protein 63	3	Cytoplasm	other
CFL1	cofilin 1	7	Nucleus	other
CFTR	cystic fibrosis transmembrane conductance regulator	7	Plasma Membrane	ion channel
CHEK1	checkpoint kinase 1	3	Nucleus	kinase
CHEK2	checkpoint kinase 2	3	Nucleus	kinase
CHGB	chromogranin B	6	Extracellular Space	other
CHMP2B	charged multivesicular body protein 2B	6	Cytoplasm	other
CHMP4B	charged multivesicular body protein 4B	5	Cytoplasm	other
CHTOP	chromatin target of PRMT1	4	Nucleus	other
CIRBP	cold inducible RNA binding protein	3	Nucleus	translation regulator
CKAP5	cytoskeleton associated protein 5	4	Nucleus	transcription regulator
CLIP1	CAP-Gly domain containing linker protein 1	4	Cytoplasm	other
CLTA	clathrin light chain A	4	Plasma Membrane	other
CLTC	clathrin heavy chain	9	Plasma Membrane	other
CLU	clusterin	3	Cytoplasm	other
CNTF	ciliary neurotrophic factor	4	Extracellular Space	cytokine
COIL	coilin	3	Nucleus	enzyme
COL18A1	collagen type XVIII alpha 1 chain	3	Extracellular Space	other
COL1A1	collagen type I alpha 1 chain	3	Extracellular Space	other
COMMD1	copper metabolism domain containing 1	3	Nucleus	transporter
COPS5	COP9 signalosome subunit 5	11	Nucleus	transcription regulator
COPS6	COP9 signalosome subunit 6	5	Nucleus	other

CREB1	cAMP responsive element binding protein 1	11	Nucleus	transcription regulator
CREBBP	CREB binding protein	8	Nucleus	transcription regulator
CREM	cAMP responsive element modulator	3	Nucleus	transcription regulator
CRK	CRK proto-oncogene, adaptor protein	5	Cytoplasm	other
CRKL	CRK like proto-oncogene, adaptor protein	4	Cytoplasm	kinase
CRMP1	collapsin response mediator protein 1	3	Cytoplasm	enzyme
CRY1	cryptochrome circadian regulator 1	4	Nucleus	enzyme
CRY2	cryptochrome circadian regulator 2	5	Nucleus	enzyme
CRYAB	crystallin alpha B	4	Nucleus	other
CSF1	colony stimulating factor 1	3	Extracellular Space	cytokine
CSF2	colony stimulating factor 2	5	Extracellular Space	cytokine
CSN2	casein beta	3	Extracellular Space	kinase
CSNK1A1	casein kinase 1 alpha 1	4	Cytoplasm	kinase
CSNK2A1	casein kinase 2 alpha 1	6	Nucleus	kinase
CSNK2B	casein kinase 2 beta	4	Cytoplasm	kinase
CST3	cystatin C	3	Extracellular Space	other
CTNNB1	catenin beta 1	17	Nucleus	transcription regulator
CTNND1	catenin delta 1	4	Nucleus	other
CUL1	cullin 1	12	Nucleus	enzyme
CUL2	cullin 2	9	Nucleus	enzyme
CUL3	cullin 3	21	Nucleus	enzyme
CUL4A	cullin 4A	7	Nucleus	other
CUL4B	cullin 4B	8	Nucleus	other
CUL5	cullin 5	10	Nucleus	ion channel
CUL7	cullin 7	14	Cytoplasm	other
CYFIP2	cytoplasmic FMR1 interacting protein 2	3	Cytoplasm	other
CYLD	CYLD lysine 63 deubiquitinase	3	Nucleus	transcription regulator
DAO	D-amino acid oxidase	4	Cytoplasm	enzyme
DAP3	death associated protein 3	4	Cytoplasm	other
DAZAP2	DAZ associated protein 2	3	Nucleus	other
DCAF7	DDB1 and CUL4 associated factor 7	4	Cytoplasm	other
DCC	DCC netrin 1 receptor	3	Plasma Membrane	transmembrane receptor
DCLK1	doublecortin like kinase 1	3	Plasma Membrane	kinase
DCP2	decapping mRNA 2	3	Nucleus	enzyme
DCTN1	dynactin subunit 1	8	Cytoplasm	other
DCTN2	dynactin subunit 2	3	Cytoplasm	other
DCUN1D1	defective in cullin neddylation 1 domain containing 1	6	Nucleus	other
DDB1	damage specific DNA binding protein 1	3	Nucleus	other
DDX1	DEAD-box helicase 1	3	Nucleus	enzyme
DDX17	DEAD-box helicase 17	4	Nucleus	enzyme
DDX3X	DEAD-box helicase 3, X-linked	8	Cytoplasm	enzyme
DDX5	DEAD-box helicase 5	8	Nucleus	enzyme
DDX6	DEAD-box helicase 6	4	Nucleus	enzyme
DERL1	derlin 1	3	Cytoplasm	other
DGCR8	DGCR8, microprocessor complex subunit	3	Nucleus	enzyme

DHX15	DEAH-box helicase 15	3	Nucleus	enzyme
DHX9	DExH-box helicase 9	7	Nucleus	enzyme
DIABLO	diablo IAP-binding mitochondrial protein	4	Cytoplasm	other
DIAPH3	diaphanous related formin 3	3	Cytoplasm	enzyme
DICER1	dicer 1, ribonuclease III	4	Cytoplasm	enzyme
DISC1	disrupted in schizophrenia 1	8	Cytoplasm	other
DLG1	discs large MAGUK scaffold protein 1	3	Plasma Membrane	kinase
DLG4	discs large MAGUK scaffold protein 4	10	Plasma Membrane	kinase
DNAAF2	dynein axonemal assembly factor 2	4	Cytoplasm	other
DNAJC10	DnaJ heat shock protein family (Hsp40) member C10	3	Cytoplasm	enzyme
DNM1	dynamin 1	4	Cytoplasm	enzyme
DROSHA	drosha ribonuclease III	4	Nucleus	enzyme
DSP	desmoplakin	4	Plasma Membrane	other
DST	dystonin	7	Plasma Membrane	other
DTNBP1	dystrobrevin binding protein 1	4	Plasma Membrane	other
DYNC1H1	dynein cytoplasmic 1 heavy chain 1	6	Cytoplasm	peptidase
DYNC1I1	dynein cytoplasmic 1 intermediate chain 1	5	Cytoplasm	other
DYNC1I2	dynein cytoplasmic 1 intermediate chain 2	5	Cytoplasm	other
DYNC1LI1	dynein cytoplasmic 1 light intermediate chain 1	4	Cytoplasm	other
DYNLL1	dynein light chain LC8-type 1	9	Cytoplasm	other
DYNLL2	dynein light chain LC8-type 2	4	Cytoplasm	other
DYNLT1	dynein light chain Tctex-type 1	5	Cytoplasm	other
E2F1	E2F transcription factor 1	4	Nucleus	transcription regulator
EDC4	enhancer of mRNA decapping 4	3	Cytoplasm	other
EEA1	early endosome antigen 1	3	Cytoplasm	other
EED	embryonic ectoderm development	18	Nucleus	transcription regulator
EEF1A1	eukaryotic translation elongation factor 1 alpha 1	8	Cytoplasm	translation regulator
EEF1D	eukaryotic translation elongation factor 1 delta	4	Cytoplasm	translation regulator
EEF1G	eukaryotic translation elongation factor 1 gamma	3	Cytoplasm	translation regulator
EEF2	eukaryotic translation elongation factor 2	6	Cytoplasm	translation regulator
EFEMP2	EGF containing fibulin extracellular matrix protein 2	3	Extracellular Space	other
EFNB2	ephrin B2	3	Plasma Membrane	kinase
EGFL7	EGF like domain multiple 7	3	Extracellular Space	other
EGFR	epidermal growth factor receptor	18	Plasma Membrane	kinase
EGR1	early growth response 1	5	Nucleus	transcription regulator
EGR2	early growth response 2	3	Nucleus	transcription regulator
EIF1B	eukaryotic translation initiation factor 1B	4	Cytoplasm	translation regulator
EIF2AK2	eukaryotic translation initiation factor 2 alpha kinase 2	3	Cytoplasm	kinase
EIF2B1	eukaryotic translation initiation factor 2B subunit alpha	3	Cytoplasm	translation regulator
EIF2B2	eukaryotic translation initiation factor 2B subunit beta	3	Cytoplasm	other
EIF2S3	eukaryotic translation initiation factor 2 subunit gamma	3	Cytoplasm	translation regulator
EIF3E	eukaryotic translation initiation factor 3 subunit E	3	Cytoplasm	other
EIF3H	eukaryotic translation initiation factor 3 subunit H	3	Cytoplasm	other
EIF4A2	eukaryotic translation initiation factor 4A2	4	Cytoplasm	translation regulator
EIF5A2	eukaryotic translation initiation factor 5A2	3	Cytoplasm	translation regulator

ELAVL1	ELAV like RNA binding protein 1	18	Cytoplasm	other
ELF3	E74 like ETS transcription factor 3	3	Nucleus	transcription regulator
EMC2	ER membrane protein complex subunit 2	3	Nucleus	other
EMD	emerin	4	Nucleus	other
ENO1	enolase 1	5	Cytoplasm	enzyme
EP300	E1A binding protein p300	12	Nucleus	transcription regulator
EPAS1	endothelial PAS domain protein 1	8	Nucleus	transcription regulator
EPB41L3	erythrocyte membrane protein band 4.1 like 3	3	Plasma Membrane	other
EPHA4	EPH receptor A4	3	Plasma Membrane	kinase
ERBB2	erb-b2 receptor tyrosine kinase 2	3	Plasma Membrane	kinase
ERBB3	erb-b2 receptor tyrosine kinase 3	4	Plasma Membrane	kinase
ERBB4	erb-b2 receptor tyrosine kinase 4	3	Plasma Membrane	kinase
ERCC2	ERCC excision repair 2, TFIIH core complex helicase subunit	3	Nucleus	enzyme
ERG	ERG, ETS transcription factor	6	Nucleus	transcription regulator
ERLIN2	ER lipid raft associated 2	4	Plasma Membrane	kinase
ESR1	estrogen receptor 1	11	Nucleus	lig-dependent nuc. receptor
ESRRA	estrogen related receptor alpha	4	Nucleus	lig-dependent nuc. receptor
ESYT2	extended synaptotagmin 2	3	Plasma Membrane	other
ETS1	ETS proto-oncogene 1, transcription factor	3	Nucleus	transcription regulator
EWSR1	EWS RNA binding protein 1	14	Nucleus	other
EXOC4	exocyst complex component 4	3	Cytoplasm	transporter
EXOSC4	exosome component 4	3	Nucleus	enzyme
EXT2	exostosin glycosyltransferase 2	3	Cytoplasm	enzyme
EZH2	enhancer of zeste 2 polycomb repressive complex 2 subunit	10	Nucleus	transcription regulator
EZR	ezrin	4	Plasma Membrane	other
F2	coagulation factor II, thrombin	3	Extracellular Space	peptidase
FADD	Fas associated via death domain	3	Cytoplasm	other
FAF2	Fas associated factor family member 2	7	Cytoplasm	other
FAM120A	family with sequence similarity 120A	3	Cytoplasm	other
FAM98A	family with sequence similarity 98 member A	3	Other	other
FAS	Fas cell surface death receptor	3	Plasma Membrane	transmembrane receptor
FBF1	Fas binding factor 1	3	Nucleus	other
FBXO25	F-box protein 25	3	Nucleus	enzyme
FBXO32	F-box protein 32	5	Cytoplasm	enzyme
FBXO6	F-box protein 6	13	Cytoplasm	enzyme
FBXW11	F-box and WD repeat domain containing 11	7	Cytoplasm	enzyme
FBXW7	F-box and WD repeat domain containing 7	3	Nucleus	transcription regulator
FEZ1	fasciculation and elongation protein zeta 1	3	Cytoplasm	other
FGFR1	fibroblast growth factor receptor 1	4	Plasma Membrane	kinase
FHL2	four and a half LIM domains 2	3	Nucleus	transcription regulator
FHL3	four and a half LIM domains 3	3	Plasma Membrane	other
FKBP4	FK506 binding protein 4	3	Nucleus	enzyme
FLOT1	flotillin 1	4	Plasma Membrane	other
FLOT2	flotillin 2	4	Plasma Membrane	other
FMR1	fragile X mental retardation 1	4	Cytoplasm	translation regulator

FN1	fibronectin 1	11	Extracellular Space	enzyme
FOS	Fos proto-oncogene, AP-1 transcription factor subunit	7	Nucleus	transcription regulator
FOXM1	forkhead box M1	6	Nucleus	transcription regulator
FOXO1	forkhead box O1	4	Nucleus	transcription regulator
FOXO3	forkhead box O3	3	Nucleus	transcription regulator
FOXO4	forkhead box O4	4	Nucleus	transcription regulator
FOXP3	forkhead box P3	9	Nucleus	transcription regulator
FUBP3	far upstream element binding protein 3	4	Nucleus	transcription regulator
FUS	FUS RNA binding protein	18	Nucleus	transcription regulator
FYN	FYN proto-oncogene, Src family tyrosine kinase	7	Plasma Membrane	kinase
G3BP1	G3BP stress granule assembly factor 1	3	Nucleus	enzyme
G3BP2	G3BP stress granule assembly factor 2	3	Cytoplasm	enzyme
GABARAP	GABA type A receptor-associated protein	4	Cytoplasm	transporter
GABARAPL1	GABA type A receptor associated protein like 1	4	Cytoplasm	other
GABARAPL2	GABA type A receptor associated protein like 2	6	Cytoplasm	other
GADD45A	growth arrest and DNA damage inducible alpha	3	Nucleus	other
GAN	gigaxonin	5	Cytoplasm	other
GAPDH	glyceraldehyde-3-phosphate dehydrogenase	13	Cytoplasm	enzyme
GAR1	GAR1 ribonucleoprotein	3	Nucleus	other
GARS	glycyl-tRNA synthetase	3	Cytoplasm	enzyme
GATA1	GATA binding protein 1	4	Nucleus	transcription regulator
GATA3	GATA binding protein 3	3	Nucleus	transcription regulator
GET4	golgi to ER traffic protein 4	4	Cytoplasm	other
GFI1	growth factor independent 1 transcriptional repressor	3	Nucleus	transcription regulator
GFI1B	growth factor independent 1B transcriptional repressor	4	Nucleus	transcription regulator
GIGYF2	GRB10 interacting GYF protein 2	3	Cytoplasm	other
GJA1	gap junction protein alpha 1	7	Plasma Membrane	transporter
GLI1	GLI family zinc finger 1	6	Nucleus	transcription regulator
GLI2	GLI family zinc finger 2	3	Nucleus	transcription regulator
GLRX3	glutaredoxin 3	3	Cytoplasm	enzyme
GLUD1	glutamate dehydrogenase 1	3	Cytoplasm	enzyme
GNB1	G protein subunit beta 1	3	Plasma Membrane	enzyme
GOLGA2	golgin A2	5	Cytoplasm	other
GOLT1B	golgi transport 1B	3	Cytoplasm	other
GRB10	growth factor receptor bound protein 10	3	Cytoplasm	other
GRB14	growth factor receptor bound protein 14	3	Plasma Membrane	other
GRB2	growth factor receptor bound protein 2	19	Cytoplasm	kinase
GRB7	growth factor receptor bound protein 7	3	Plasma Membrane	other
GRIA1	glutamate ionotropic receptor AMPA type subunit 1	3	Plasma Membrane	ion channel
GRIA2	glutamate ionotropic receptor AMPA type subunit 2	3	Plasma Membrane	ion channel
GRIA3	glutamate ionotropic receptor AMPA type subunit 3	3	Plasma Membrane	ion channel
GRIN1	glutamate ionotropic receptor NMDA type subunit 1	6	Plasma Membrane	ion channel
GRIP1	glutamate receptor interacting protein 1	4	Plasma Membrane	transcription regulator
GSK3B	glycogen synthase kinase 3 beta	11	Nucleus	kinase
GSTP1	glutathione S-transferase pi 1	3	Cytoplasm	enzyme

GTF2E2	general transcription factor IIE subunit 2	3	Nucleus	transcription regulator
GTF2F1	general transcription factor IIF subunit 1	3	Nucleus	transcription regulator
GTF2I	general transcription factor IIi	3	Nucleus	transcription regulator
H2AFX	H2A histone family member X	5	Nucleus	transcription regulator
HACD3	3-hydroxyacyl-CoA dehydratase 3	3	Cytoplasm	enzyme
HADHA	hydroxyacyl-CoA dehydrogenase trifunctional multienzyme complex subunit	3	Cytoplasm	enzyme
HAP1	huntingtin associated protein 1	3	Cytoplasm	other
HAUS1	HAUS augmin like complex subunit 1	3	Nucleus	other
HAX1	HCLS1 associated protein X-1	3	Cytoplasm	other
HBB	hemoglobin subunit beta	3	Cytoplasm	transporter
HBEGF	heparin binding EGF like growth factor	4	Extracellular Space	growth factor
HDAC1	histone deacetylase 1	14	Nucleus	transcription regulator
HDAC2	histone deacetylase 2	6	Nucleus	transcription regulator
HDAC3	histone deacetylase 3	4	Nucleus	transcription regulator
HDAC4	histone deacetylase 4	6	Nucleus	transcription regulator
HDAC5	histone deacetylase 5	5	Nucleus	transcription regulator
HDAC6	histone deacetylase 6	13	Nucleus	transcription regulator
HDAC7	histone deacetylase 7	3	Nucleus	transcription regulator
HECW2	HECT, C2 and WW domain containing E3 ubiquitin protein ligase 2	5	Extracellular Space	enzyme
HERC2	HECT and RLD domain containing E3 ubiquitin protein ligase 2	4	Cytoplasm	enzyme
HERPUD1	homocysteine inducible ER protein with ubiquitin like domain 1	3	Cytoplasm	other
HEYL	hes related family bHLH transcription factor with YRPW motif-like	3	Nucleus	transcription regulator
HIF1A	hypoxia inducible factor 1 alpha subunit	9	Nucleus	transcription regulator
HIPK2	homeodomain interacting protein kinase 2	3	Nucleus	kinase
HIPK3	homeodomain interacting protein kinase 3	3	Nucleus	kinase
HIST1H1A	histone cluster 1 H1 family member a	3	Nucleus	other
HIST1H3A	histone cluster 1 H3 family member a	3	Nucleus	other
HIST3H3	histone cluster 3 H3	5	Nucleus	other
HK1	hexokinase 1	3	Cytoplasm	kinase
HLA-B	major histocompatibility complex, class I, B	3	Plasma Membrane	transmembrane receptor
HMGA1	high mobility group AT-hook 1	3	Nucleus	transcription regulator
HMGB1	high mobility group box 1	8	Nucleus	transcription regulator
HMGB2	high mobility group box 2	3	Nucleus	transcription regulator
HMMR	hyaluronan mediated motility receptor	3	Plasma Membrane	transmembrane receptor
HNF1A	HNF1 homeobox A	5	Nucleus	transcription regulator
HNF4A	hepatocyte nuclear factor 4 alpha	18	Nucleus	transcription regulator
HNRNPA0	heterogeneous nuclear ribonucleoprotein A0	3	Nucleus	other
HNRNPA1	heterogeneous nuclear ribonucleoprotein A1	15	Nucleus	enzyme
HNRNPA2B1	heterogeneous nuclear ribonucleoprotein A2/B1	12	Nucleus	other
HNRNPA3	heterogeneous nuclear ribonucleoprotein A3	8	Nucleus	other
HNRNPC	heterogeneous nuclear ribonucleoprotein C (C1/C2)	4	Nucleus	other
HNRNPD	heterogeneous nuclear ribonucleoprotein D	8	Nucleus	transcription regulator
HNRNPDL	heterogeneous nuclear ribonucleoprotein D like	4	Nucleus	other
HNRNPF	heterogeneous nuclear ribonucleoprotein F	5	Nucleus	other
HNRNPH1	heterogeneous nuclear ribonucleoprotein H1	7	Nucleus	other

HNRNPH2	heterogeneous nuclear ribonucleoprotein H2	5	Nucleus	other
HNRNPH3	heterogeneous nuclear ribonucleoprotein H3	6	Nucleus	other
HNRNPK	heterogeneous nuclear ribonucleoprotein K	9	Nucleus	transcription regulator
HNRNPL	heterogeneous nuclear ribonucleoprotein L	8	Nucleus	other
HNRNPM	heterogeneous nuclear ribonucleoprotein M	6	Nucleus	other
HNRNPR	heterogeneous nuclear ribonucleoprotein R	6	Nucleus	other
HNRNPU	heterogeneous nuclear ribonucleoprotein U	6	Nucleus	transporter
HNRNPUL1	heterogeneous nuclear ribonucleoprotein U like 1	5	Nucleus	other
HNRNPUL2	heterogeneous nuclear ribonucleoprotein U like 2	3	Nucleus	other
HOXA10	homeobox A10	3	Nucleus	transcription regulator
HOXA13	homeobox A13	3	Nucleus	transcription regulator
HSBP1	heat shock factor binding protein 1	4	Nucleus	other
HSF1	heat shock transcription factor 1	4	Nucleus	transcription regulator
HSP90AA1	heat shock protein 90 alpha family class A member 1	11	Cytoplasm	enzyme
HSP90AB1	heat shock protein 90 alpha family class B member 1	9	Cytoplasm	enzyme
HSP90B1	heat shock protein 90 beta family member 1	5	Cytoplasm	other
HSPA1A	heat shock protein family A (Hsp70) member 1A	10	Cytoplasm	enzyme
HSPA1B	heat shock protein family A (Hsp70) member 1A	3	Cytoplasm	enzyme
HSPA2	heat shock protein family A (Hsp70) member 2	4	Cytoplasm	other
HSPA4	heat shock protein family A (Hsp70) member 4	8	Cytoplasm	other
HSPA5	heat shock protein family A (Hsp70) member 5	14	Cytoplasm	enzyme
HSPA8	heat shock protein family A (Hsp70) member 8	12	Cytoplasm	enzyme
HSPA9	heat shock protein family A (Hsp70) member 9	7	Cytoplasm	other
HSPB1	heat shock protein family B (small) member 1	11	Cytoplasm	other
HSPB2	heat shock protein family B (small) member 2	4	Cytoplasm	other
HSPD1	heat shock protein family D (Hsp60) member 1	4	Cytoplasm	enzyme
HSPG2	heparan sulfate proteoglycan 2	3	Extracellular Space	enzyme
HSPH1	heat shock protein family H (Hsp110) member 1	3	Cytoplasm	other
HTRA2	HtrA serine peptidase 2	4	Cytoplasm	peptidase
HTT	huntingtin	23	Cytoplasm	transcription regulator
HUWE1	HECT, UBA and WWE domain containing 1, E3 ubiquitin protein ligase	3	Nucleus	transcription regulator
HYOU1	hypoxia up-regulated 1	4	Cytoplasm	other
ICAM1	intercellular adhesion molecule 1	6	Plasma Membrane	transmembrane receptor
ID2	inhibitor of DNA binding 2	3	Nucleus	transcription regulator
ID3	inhibitor of DNA binding 3, HLH protein	3	Nucleus	transcription regulator
IFI16	interferon gamma inducible protein 16	5	Nucleus	transcription regulator
IFIT2	interferon induced protein with tetratricopeptide repeats 2	3	Cytoplasm	other
IFIT3	interferon induced protein with tetratricopeptide repeats 3	4	Cytoplasm	other
IFNG	interferon gamma	3	Extracellular Space	cytokine
IGF2BP2	insulin like growth factor 2 mRNA binding protein 2	3	Cytoplasm	translation regulator
IGSF8	immunoglobulin superfamily member 8	4	Plasma Membrane	other
IKBKE	inhibitor of nuclear factor kappa B kinase subunit epsilon	4	Cytoplasm	kinase
IKBKG	inhibitor of nuclear factor kappa B kinase subunit gamma	4	Nucleus	kinase
IL10	interleukin 10	3	Extracellular Space	cytokine
IL4	interleukin 4	3	Extracellular Space	cytokine

IL6	interleukin 6	4	Extracellular Space	cytokine
IL6ST	interleukin 6 signal transducer	3	Plasma Membrane	transmembrane receptor
ILF2	interleukin enhancer binding factor 2	5	Nucleus	transcription regulator
ILF3	interleukin enhancer binding factor 3	10	Nucleus	transcription regulator
ILK	integrin linked kinase	6	Plasma Membrane	kinase
IMMT	inner membrane mitochondrial protein	4	Cytoplasm	other
INA	internexin neuronal intermediate filament protein alpha	3	Cytoplasm	other
INSIG1	insulin induced gene 1	3	Cytoplasm	other
INSR	insulin receptor	6	Plasma Membrane	kinase
INVS	inversin	3	Nucleus	transcription regulator
IQCB1	IQ motif containing B1	7	Extracellular Space	other
IQGAP1	IQ motif containing GTPase activating protein 1	4	Cytoplasm	other
IRAK1	interleukin 1 receptor associated kinase 1	3	Plasma Membrane	kinase
IRAK2	interleukin 1 receptor associated kinase 2	3	Plasma Membrane	kinase
IRF8	interferon regulatory factor 8	3	Nucleus	transcription regulator
ISG15	ISG15 ubiquitin-like modifier	5	Extracellular Space	other
ISL1	ISL LIM homeobox 1	3	Nucleus	transcription regulator
ISLR	immunoglobulin superfamily containing leucine rich repeat	3	Extracellular Space	other
ITGA4	integrin subunit alpha 4	11	Plasma Membrane	transmembrane receptor
ITGAV	integrin subunit alpha V	3	Plasma Membrane	transmembrane receptor
ITGB1	integrin subunit beta 1	4	Plasma Membrane	transmembrane receptor
ITGB3	integrin subunit beta 3	5	Plasma Membrane	transmembrane receptor
ITGB4	integrin subunit beta 4	3	Plasma Membrane	transmembrane receptor
ITPR1	inositol 1,4,5-trisphosphate receptor type 1	6	Cytoplasm	ion channel
ITPR2	inositol 1,4,5-trisphosphate receptor type 2	3	Cytoplasm	ion channel
ITSN2	intersectin 2	3	Cytoplasm	other
JRK	Jrk helix-turn-helix protein	3	Nucleus	other
JUN	Jun proto-oncogene, AP-1 transcription factor subunit	12	Nucleus	transcription regulator
JUNB	JunB proto-oncogene, AP-1 transcription factor subunit	4	Nucleus	transcription regulator
JUND	JunD proto-oncogene, AP-1 transcription factor subunit	4	Nucleus	transcription regulator
JUP	junction plakoglobin	4	Plasma Membrane	other
KAT5	lysine acetyltransferase 5	7	Nucleus	transcription regulator
KCNMA1	potassium calcium-activated channel subfamily M alpha 1	7	Plasma Membrane	ion channel
KCTD17	potassium channel tetramerization domain containing 17	3	Cytoplasm	other
KCTD5	potassium channel tetramerization domain containing 5	4	Cytoplasm	other
KDM5A	lysine demethylase 5A	3	Nucleus	transcription regulator
KDR	kinase insert domain receptor	4	Plasma Membrane	kinase
KHDRBS1	KH RNA binding domain containing, signal transduction associated 1	5	Nucleus	transcription regulator
KHDRBS2	KH RNA binding domain containing, signal transduction associated 2	4	Nucleus	other
KIAA0368	KIAA0368	3	Cytoplasm	other
KIF1BP	KIF1 binding protein	3	Cytoplasm	enzyme
KIF22	kinesin family member 22	3	Nucleus	other
KIF23	kinesin family member 23	3	Cytoplasm	other
KIF3A	kinesin family member 3A	5	Cytoplasm	enzyme
KIF4A	kinesin family member 4A	3	Nucleus	other

KIF5A	kinesin family member 5A	6	Cytoplasm	transporter
KIF5B	kinesin family member 5B	5	Cytoplasm	other
KIFAP3	kinesin associated protein 3	4	Cytoplasm	other
KIFC3	kinesin family member C3	3	Cytoplasm	enzyme
KLC1	kinesin light chain 1	3	Cytoplasm	other
KLC2	kinesin light chain 2	4	Cytoplasm	other
KLF11	Kruppel like factor 11	3	Nucleus	transcription regulator
KLF2	Kruppel like factor 2	4	Nucleus	transcription regulator
KLF3	Kruppel like factor 3	7	Nucleus	transcription regulator
KLF4	Kruppel like factor 4	4	Nucleus	transcription regulator
KMT2B	lysine methyltransferase 2B	4	Nucleus	transcription regulator
KPNA2	karyopherin subunit alpha 2	7	Nucleus	transporter
KPNB1	karyopherin subunit beta 1	3	Nucleus	transporter
KRT8	keratin 8	3	Cytoplasm	other
KRTAP10-3	keratin associated protein 10-3	4	Cytoplasm	other
KRTAP10-7	keratin associated protein 10-7	4	Cytoplasm	other
KRTAP10-9	keratin associated protein 10-9	3	Cytoplasm	other
KTN1	kinectin 1	4	Plasma Membrane	transmembrane receptor
LCK	LCK proto-oncogene, Src family tyrosine kinase	4	Cytoplasm	kinase
LDHA	lactate dehydrogenase A	3	Cytoplasm	enzyme
LEP	leptin	3	Extracellular Space	growth factor
LHX2	LIM homeobox 2	3	Nucleus	transcription regulator
LIF	LIF, interleukin 6 family cytokine	3	Extracellular Space	cytokine
LIG4	DNA ligase 4	3	Nucleus	enzyme
LMNA	lamin A/C	9	Nucleus	other
LMNB1	lamin B1	3	Nucleus	other
LNX1	ligand of numb-protein X 1	5	Cytoplasm	enzyme
LRIF1	ligand dependent nuclear receptor interacting factor 1	3	Nucleus	other
LRIG1	leucine rich repeats and immunoglobulin like domains 1	3	Extracellular Space	other
LRP1	LDL receptor related protein 1	3	Plasma Membrane	transmembrane receptor
LRPPRC	leucine rich pentatricopeptide repeat containing	3	Cytoplasm	other
LRRK1	leucine rich repeat kinase 1	5	Cytoplasm	kinase
LRRK2	leucine rich repeat kinase 2	9	Cytoplasm	kinase
MAG	myelin associated glycoprotein	3	Plasma Membrane	other
MAP1A	microtubule associated protein 1A	4	Cytoplasm	other
MAP1B	microtubule associated protein 1B	4	Cytoplasm	other
MAP1LC3A	microtubule associated protein 1 light chain 3 alpha	4	Cytoplasm	other
MAP1LC3B	microtubule associated protein 1 light chain 3 beta	5	Cytoplasm	other
MAP1LC3C	microtubule associated protein 1 light chain 3 gamma	3	Cytoplasm	other
MAP2K1	mitogen-activated protein kinase kinase 1	3	Cytoplasm	kinase
MAP3K14	mitogen-activated protein kinase kinase kinase 14	4	Cytoplasm	kinase
MAP3K3	mitogen-activated protein kinase kinase kinase 3	5	Cytoplasm	kinase
MAP3K5	mitogen-activated protein kinase kinase kinase 5	4	Cytoplasm	kinase
MAP3K7	mitogen-activated protein kinase kinase kinase 7	3	Cytoplasm	kinase
MAPK1	mitogen-activated protein kinase 1	9	Cytoplasm	kinase

MAPK13	mitogen-activated protein kinase 13	7	Cytoplasm	kinase
MAPK3	mitogen-activated protein kinase 3	16	Cytoplasm	kinase
MAPK8	mitogen-activated protein kinase 8	5	Cytoplasm	kinase
MAPRE1	microtubule associated protein RP/EB family member 1	3	Cytoplasm	other
MAPT	microtubule associated protein tau	9	Plasma Membrane	other
MARK2	microtubule affinity regulating kinase 2	4	Cytoplasm	kinase
MATR3	matrin 3	9	Nucleus	other
MAX	MYC associated factor X	5	Nucleus	transcription regulator
MBD3	methyl-CpG binding domain protein 3	3	Nucleus	enzyme
MBP	myelin basic protein	7	Extracellular Space	other
MCC	mutated in colorectal cancers	3	Cytoplasm	other
MCM2	minichromosome maintenance complex component 2	16	Nucleus	enzyme
MCM4	minichromosome maintenance complex component 4	3	Nucleus	enzyme
MCM5	minichromosome maintenance complex component 5	4	Nucleus	enzyme
MDC1	mediator of DNA damage checkpoint 1	3	Nucleus	other
MDM2	MDM2 proto-oncogene	7	Nucleus	transcription regulator
MDN1	midasin AAA ATPase 1	3	Nucleus	other
MECP2	methyl-CpG binding protein 2	3	Nucleus	transcription regulator
MED16	mediator complex subunit 16	3	Nucleus	transcription regulator
MED23	mediator complex subunit 23	3	Nucleus	transcription regulator
MED24	mediator complex subunit 24	4	Nucleus	transcription regulator
MED4	mediator complex subunit 4	4	Nucleus	transcription regulator
MED6	mediator complex subunit 6	3	Nucleus	transcription regulator
MEPCE	methylphosphate capping enzyme	3	Other	enzyme
MIB1	mindbomb E3 ubiquitin protein ligase 1	8	Cytoplasm	enzyme
MLST8	MTOR associated protein, LST8 homolog	3	Cytoplasm	other
MOV10	Mov10 RISC complex RNA helicase	12	Nucleus	enzyme
MPP1	membrane palmitoylated protein 1	3	Plasma Membrane	kinase
MRPL48	mitochondrial ribosomal protein L48	3	Cytoplasm	other
MSN	moesin	4	Plasma Membrane	other
MTA1	metastasis associated 1	4	Nucleus	transcription regulator
MTA2	metastasis associated 1 family member 2	3	Nucleus	transcription regulator
MTNR1B	melatonin receptor 1B	4	Plasma Membrane	G-protein coupled receptor
MTOR	mechanistic target of rapamycin kinase	3	Nucleus	kinase
MTUS2	microtubule associated scaffold protein 2	4	Nucleus	other
MYBBP1A	MYB binding protein 1a	3	Nucleus	transcription regulator
MYC	MYC proto-oncogene, bHLH transcription factor	18	Nucleus	transcription regulator
MYCN	MYCN proto-oncogene, bHLH transcription factor	3	Nucleus	transcription regulator
MYD88	myeloid differentiation primary response 88	3	Plasma Membrane	other
MYH10	myosin heavy chain 10	5	Cytoplasm	enzyme
MYH14	myosin heavy chain 14	3	Extracellular Space	enzyme
MYH9	myosin heavy chain 9	7	Cytoplasm	enzyme
MYO1C	myosin IC	3	Cytoplasm	enzyme
MYO6	myosin VI	3	Cytoplasm	other
NACA	nascent polypeptide-associated complex alpha subunit	3	Cytoplasm	transcription regulator

NANOG	Nanog homeobox	6	Nucleus	transcription regulator
NAP1L1	nucleosome assembly protein 1 like 1	3	Nucleus	other
NAP1L4	nucleosome assembly protein 1 like 4	3	Cytoplasm	other
NCBP1	nuclear cap binding protein subunit 1	3	Nucleus	other
NCK1	NCK adaptor protein 1	5	Cytoplasm	kinase
NCK2	NCK adaptor protein 2	3	Cytoplasm	kinase
NCKAP1	NCK associated protein 1	3	Plasma Membrane	other
NCL	nucleolin	5	Nucleus	other
NCOA1	nuclear receptor coactivator 1	3	Nucleus	transcription regulator
NCOA2	nuclear receptor coactivator 2	4	Nucleus	transcription regulator
NCOA3	nuclear receptor coactivator 3	4	Nucleus	transcription regulator
NCOA4	nuclear receptor coactivator 4	4	Nucleus	transcription regulator
NCOA5	nuclear receptor coactivator 5	4	Nucleus	other
NCOA6	nuclear receptor coactivator 6	5	Nucleus	transcription regulator
NCOR1	nuclear receptor corepressor 1	6	Nucleus	transcription regulator
NCOR2	nuclear receptor corepressor 2	4	Nucleus	transcription regulator
NCSTN	nicastrin	3	Plasma Membrane	peptidase
NDE1	nudE neurodevelopment protein 1	4	Nucleus	other
NDEL1	nudE neurodevelopment protein 1 like 1	7	Nucleus	peptidase
NDRG1	N-myc downstream regulated 1	3	Nucleus	kinase
NECTIN3	nectin cell adhesion molecule 3	3	Plasma Membrane	other
NEDD1	neural precursor cell expressed, developmentally down-regulated 1	3	Cytoplasm	other
NEDD4	neural precursor cell expressed, developmentally down-regulated 4, E3 ubi	5	Cytoplasm	enzyme
NEDD8	neural precursor cell expressed, developmentally down-regulated 8	6	Nucleus	enzyme
NEFH	neurofilament heavy	5	Cytoplasm	other
NEFL	neurofilament light	5	Cytoplasm	other
NEFM	neurofilament medium	6	Plasma Membrane	other
NEK1	NIMA related kinase 1	5	Nucleus	kinase
NEK4	NIMA related kinase 4	6	Nucleus	kinase
NEK5	NIMA related kinase 5	3	Other	kinase
NFATC1	nuclear factor of activated T cells 1	4	Nucleus	transcription regulator
NFATC2	nuclear factor of activated T cells 2	3	Nucleus	transcription regulator
NFE2L2	nuclear factor, erythroid 2 like 2	8	Nucleus	transcription regulator
NFKB1	nuclear factor kappa B subunit 1	6	Nucleus	transcription regulator
NFKB2	nuclear factor kappa B subunit 2	4	Nucleus	transcription regulator
NFKBIA	NFKB inhibitor alpha	14	Cytoplasm	transcription regulator
NFS1	NFS1, cysteine desulfurase	3	Cytoplasm	enzyme
NGFR	nerve growth factor receptor	5	Plasma Membrane	transmembrane receptor
NIN	ninein	3	Cytoplasm	other
NINL	ninein like	4	Cytoplasm	other
NKRF	NFKB repressing factor	3	Nucleus	transcription regulator
NKX2-1	NK2 homeobox 1	5	Nucleus	transcription regulator
NONO	non-POU domain containing octamer binding	10	Nucleus	other
NOP56	NOP56 ribonucleoprotein	3	Nucleus	other
NOS2	nitric oxide synthase 2	5	Cytoplasm	enzyme

NOS3	nitric oxide synthase 3	5	Cytoplasm	enzyme
NOTCH1	notch 1	6	Plasma Membrane	transcription regulator
NOVA1	NOVA alternative splicing regulator 1	4	Nucleus	other
NPM1	nucleophosmin 1	12	Nucleus	transcription regulator
NR0B2	nuclear receptor subfamily 0 group B member 2	3	Nucleus	lig-dependent nuc. receptor
NR1H2	nuclear receptor subfamily 1 group H member 2	4	Nucleus	lig-dependent nuc. receptor
NR1H3	nuclear receptor subfamily 1 group H member 3	3	Nucleus	lig-dependent nuc. receptor
NR1H4	nuclear receptor subfamily 1 group H member 4	4	Nucleus	lig-dependent nuc. receptor
NR2F1	nuclear receptor subfamily 2 group F member 1	3	Nucleus	lig-dependent nuc. receptor
NR2F2	nuclear receptor subfamily 2 group F member 2	4	Nucleus	lig-dependent nuc. receptor
NR3C1	nuclear receptor subfamily 3 group C member 1	5	Nucleus	lig-dependent nuc. receptor
NR3C2	nuclear receptor subfamily 3 group C member 2	3	Nucleus	lig-dependent nuc. receptor
NR4A1	nuclear receptor subfamily 4 group A member 1	8	Nucleus	lig-dependent nuc. receptor
NRP1	neuropilin 1	3	Plasma Membrane	transmembrane receptor
NSF	N-ethylmaleimide sensitive factor, vesicle fusing ATPase	7	Cytoplasm	transporter
NSFL1C	NSFL1 cofactor	3	Cytoplasm	other
NTRK1	neurotrophic receptor tyrosine kinase 1	26	Plasma Membrane	kinase
NUDC	nuclear distribution C, dynein complex regulator	3	Cytoplasm	other
NUDT21	nudix hydrolase 21	7	Nucleus	other
NUMA1	nuclear mitotic apparatus protein 1	3	Nucleus	other
NUP107	nucleoporin 107	4	Nucleus	other
NUP98	nucleoporin 98	3	Nucleus	transporter
NXF1	nuclear RNA export factor 1	15	Nucleus	other
OBSL1	obscurin like 1	12	Cytoplasm	other
OFD1	OFD1, centriole and centriolar satellite protein	4	Plasma Membrane	other
OGG1	8-oxoguanine DNA glycosylase	4	Nucleus	enzyme
ONECUT1	one cut homeobox 1	5	Nucleus	transcription regulator
OPTN	optineurin	7	Cytoplasm	other
OSBPL1A	oxysterol binding protein like 1A	3	Cytoplasm	transporter
P4HB	prolyl 4-hydroxylase subunit beta	4	Cytoplasm	enzyme
PA2G4	proliferation-associated 2G4	4	Nucleus	transcription regulator
PABPC1	poly(A) binding protein cytoplasmic 1	4	Cytoplasm	translation regulator
PAFAH1B1	platelet activating factor acetylhydrolase 1b regulatory subunit 1	4	Cytoplasm	enzyme
PAN2	PAN2 poly(A) specific ribonuclease subunit	5	Cytoplasm	peptidase
PARK7	Parkinsonism associated deglycase	8	Nucleus	enzyme
PARP1	poly(ADP-ribose) polymerase 1	4	Nucleus	enzyme
PCBP1	poly(rC) binding protein 1	4	Nucleus	translation regulator
PCBP2	poly(rC) binding protein 2	4	Nucleus	other
PCLO	piccolo presynaptic cytomatrix protein	3	Cytoplasm	transporter
PCNA	proliferating cell nuclear antigen	4	Nucleus	enzyme
PDCD4	programmed cell death 4	5	Nucleus	other
PDCD6	programmed cell death 6	5	Cytoplasm	other
PDCD6IP	programmed cell death 6 interacting protein	3	Cytoplasm	other
PDGFRA	platelet derived growth factor receptor alpha	3	Plasma Membrane	kinase
PDIA3	protein disulfide isomerase family A member 3	4	Cytoplasm	peptidase

PDPK1	3-phosphoinositide dependent protein kinase 1	3	Cytoplasm	kinase
PDX1	pancreatic and duodenal homeobox 1	4	Nucleus	transcription regulator
PEX14	peroxisomal biogenesis factor 14	3	Cytoplasm	transcription regulator
PFKL	phosphofructokinase, liver type	3	Cytoplasm	kinase
PFKP	phosphofructokinase, platelet	4	Cytoplasm	kinase
PFN1	profilin 1	7	Cytoplasm	other
PGK1	phosphoglycerate kinase 1	4	Cytoplasm	kinase
PHB	prohibitin	6	Nucleus	transcription regulator
PHB2	prohibitin 2	3	Cytoplasm	transcription regulator
PHGDH	phosphoglycerate dehydrogenase	4	Cytoplasm	enzyme
PIAS1	protein inhibitor of activated STAT 1	4	Nucleus	transcription regulator
PIAS2	protein inhibitor of activated STAT 2	5	Nucleus	transcription regulator
PIAS3	protein inhibitor of activated STAT 3	3	Nucleus	transcription regulator
PIAS4	protein inhibitor of activated STAT 4	5	Nucleus	transcription regulator
PIK3R1	phosphoinositide-3-kinase regulatory subunit 1	3	Cytoplasm	kinase
PIK3R2	phosphoinositide-3-kinase regulatory subunit 2	6	Cytoplasm	kinase
PIN1	peptidylprolyl cis/trans isomerase, NIMA-interacting 1	4	Nucleus	enzyme
PIP	prolactin induced protein	5	Extracellular Space	peptidase
PKM	pyruvate kinase M1/2	7	Cytoplasm	kinase
PKN1	protein kinase N1	3	Cytoplasm	kinase
PKN2	protein kinase N2	5	Cytoplasm	kinase
PKP1	plakophilin 1	3	Plasma Membrane	other
PLCB1	phospholipase C beta 1	4	Cytoplasm	enzyme
PLCG1	phospholipase C gamma 1	4	Cytoplasm	enzyme
PLCG2	phospholipase C gamma 2	3	Cytoplasm	enzyme
PLEC	plectin	5	Cytoplasm	other
PLK1	polo like kinase 1	5	Nucleus	kinase
PLSCR1	phospholipid scramblase 1	3	Plasma Membrane	enzyme
PML	promyelocytic leukemia	7	Nucleus	transcription regulator
PNKD	paroxysmal nonkinesigenic dyskinesia	3	Nucleus	other
POLR2A	RNA polymerase II subunit A	6	Nucleus	enzyme
POT1	protection of telomeres 1	4	Nucleus	other
POU2F1	POU class 2 homeobox 1	5	Nucleus	transcription regulator
POU4F1	POU class 4 homeobox 1	7	Nucleus	transcription regulator
POU5F1	POU class 5 homeobox 1	10	Nucleus	transcription regulator
PPARA	peroxisome proliferator activated receptor alpha	3	Nucleus	lig-dependent nuc. receptor
PPARG	peroxisome proliferator activated receptor gamma	6	Nucleus	lig-dependent nuc. receptor
PPARGC1A	PPARG coactivator 1 alpha	8	Nucleus	transcription regulator
PPHLN1	periphilin 1	3	Nucleus	other
PPIA	peptidylprolyl isomerase A	4	Cytoplasm	enzyme
PPP1CA	protein phosphatase 1 catalytic subunit alpha	8	Cytoplasm	phosphatase
PPP1CB	protein phosphatase 1 catalytic subunit beta	4	Cytoplasm	phosphatase
PPP1CC	protein phosphatase 1 catalytic subunit gamma	6	Nucleus	phosphatase
PPP1R12A	protein phosphatase 1 regulatory subunit 12A	3	Cytoplasm	phosphatase
PPP2CA	protein phosphatase 2 catalytic subunit alpha	3	Cytoplasm	phosphatase

PPP2R1A	protein phosphatase 2 scaffold subunit Aalpha	8	Cytoplasm	phosphatase
PPP2R1B	protein phosphatase 2 scaffold subunit Abeta	3	Plasma Membrane	phosphatase
PPP2R2A	protein phosphatase 2 regulatory subunit Balpha	3	Cytoplasm	phosphatase
PPP2R5D	protein phosphatase 2 regulatory subunit B'delta	3	Nucleus	phosphatase
PPP3CA	protein phosphatase 3 catalytic subunit alpha	3	Cytoplasm	phosphatase
PPP5C	protein phosphatase 5 catalytic subunit	3	Nucleus	phosphatase
PPP6R2	protein phosphatase 6 regulatory subunit 2	3	Cytoplasm	other
PRDX1	peroxiredoxin 1	4	Cytoplasm	enzyme
PRKAA1	protein kinase AMP-activated catalytic subunit alpha 1	3	Cytoplasm	kinase
PRKACA	protein kinase cAMP-activated catalytic subunit alpha	3	Cytoplasm	kinase
PRKCA	protein kinase C alpha	3	Cytoplasm	kinase
PRKCB	protein kinase C beta	5	Cytoplasm	kinase
PRKCD	protein kinase C delta	3	Cytoplasm	kinase
PRKCG	protein kinase C gamma	5	Cytoplasm	kinase
PRKCZ	protein kinase C zeta	3	Cytoplasm	kinase
PRKDC	protein kinase, DNA-activated, catalytic polypeptide	5	Nucleus	kinase
PARK2	parkin RBR E3 ubiquitin protein ligase	14	Cytoplasm	enzyme
PRMT1	protein arginine methyltransferase 1	4	Nucleus	enzyme
PRNP	prion protein	5	Plasma Membrane	other
PRPF19	pre-mRNA processing factor 19	4	Nucleus	enzyme
PRPF4	pre-mRNA processing factor 4	3	Nucleus	other
PRPH	peripherin	3	Plasma Membrane	other
PRRC2A	proline rich coiled-coil 2A	4	Cytoplasm	other
PSEN1	presenilin 1	5	Plasma Membrane	peptidase
PSMA3	proteasome subunit alpha 3	4	Cytoplasm	peptidase
PSMA5	proteasome subunit alpha 5	4	Cytoplasm	peptidase
PSMA6	proteasome subunit alpha 6	3	Cytoplasm	peptidase
PSMC2	proteasome 26S subunit, ATPase 2	3	Nucleus	peptidase
PSMC3	proteasome 26S subunit, ATPase 3	5	Nucleus	enzyme
PSMC5	proteasome 26S subunit, ATPase 5	3	Nucleus	transcription regulator
PSMD4	proteasome 26S subunit, non-ATPase 4	3	Cytoplasm	other
PTBP1	polypyrimidine tract binding protein 1	6	Nucleus	enzyme
PTCH1	patched 1	3	Plasma Membrane	transmembrane receptor
PTEN	phosphatase and tensin homolog	6	Cytoplasm	phosphatase
PTGDS	prostaglandin D2 synthase	3	Cytoplasm	enzyme
PTGS2	prostaglandin-endoperoxide synthase 2	3	Cytoplasm	enzyme
PTK2	protein tyrosine kinase 2	3	Cytoplasm	kinase
PTK2B	protein tyrosine kinase 2 beta	3	Cytoplasm	kinase
PTPN11	protein tyrosine phosphatase, non-receptor type 11	3	Cytoplasm	phosphatase
PTPN23	protein tyrosine phosphatase, non-receptor type 23	5	Cytoplasm	phosphatase
PTPN3	protein tyrosine phosphatase, non-receptor type 3	3	Cytoplasm	phosphatase
PTPRK	protein tyrosine phosphatase, receptor type K	3	Plasma Membrane	phosphatase
PTPRO	protein tyrosine phosphatase, receptor type O	3	Plasma Membrane	phosphatase
PURA	purine rich element binding protein A	6	Nucleus	transcription regulator
PVR	poliovirus receptor	3	Plasma Membrane	other

RAB14	RAB14, member RAS oncogene family	3	Cytoplasm	enzyme
RAB5C	RAB5C, member RAS oncogene family	5	Cytoplasm	enzyme
RAB7A	RAB7A, member RAS oncogene family	5	Cytoplasm	enzyme
RAC1	Rac family small GTPase 1	6	Plasma Membrane	enzyme
RACGAP1	Rac GTPase activating protein 1	3	Cytoplasm	transporter
RACK1	receptor for activated C kinase 1	7	Cytoplasm	enzyme
RAD23A	RAD23 homolog A, nucleotide excision repair protein	4	Nucleus	other
RAD23B	RAD23 homolog B, nucleotide excision repair protein	3	Nucleus	other
RAD50	RAD50 double strand break repair protein	3	Nucleus	enzyme
RAD51	RAD51 recombinase	3	Nucleus	enzyme
RAD54L2	RAD54 like 2	3	Nucleus	transcription regulator
RAF1	Raf-1 proto-oncogene, serine/threonine kinase	8	Cytoplasm	kinase
RALY	RALY heterogeneous nuclear ribonucleoprotein	5	Nucleus	transcription regulator
RAN	RAN, member RAS oncogene family	3	Nucleus	enzyme
RAPGEF2	Rap guanine nucleotide exchange factor 2	4	Cytoplasm	other
RAPGEF4	Rap guanine nucleotide exchange factor 4	3	Cytoplasm	other
RARA	retinoic acid receptor alpha	3	Nucleus	lig-dependent nuc. receptor
RASA1	RAS p21 protein activator 1	4	Cytoplasm	transporter
RB1	RB transcriptional corepressor 1	5	Nucleus	transcription regulator
RB1CC1	RB1 inducible coiled-coil 1	4	Nucleus	other
RBBP4	RB binding protein 4, chromatin remodeling factor	6	Nucleus	enzyme
RBBP5	RB binding protein 5, histone lysine methyltransferase complex subunit	3	Nucleus	enzyme
RBBP7	RB binding protein 7, chromatin remodeling factor	4	Nucleus	transcription regulator
RBFOX1	RNA binding fox-1 homolog 1	3	Cytoplasm	other
RBFOX2	RNA binding fox-1 homolog 2	7	Nucleus	transcription regulator
RBM12B	RNA binding motif protein 12B	3	Other	other
RBM14	RNA binding motif protein 14	4	Nucleus	transcription regulator
RBM26	RNA binding motif protein 26	3	Other	other
RBM3	RNA binding motif protein 3	4	Cytoplasm	other
RBM6	RNA binding motif protein 6	3	Nucleus	other
RBMX	RNA binding motif protein, X-linked	6	Nucleus	other
RBPJ	recombination signal binding protein for immunoglobulin kappa J region	4	Nucleus	transcription regulator
RBPMS	RNA binding protein, mRNA processing factor	3	Cytoplasm	transcription regulator
RCN1	reticulocalbin 1	3	Cytoplasm	other
REL	REL proto-oncogene, NF-kB subunit	4	Nucleus	transcription regulator
RELA	RELA proto-oncogene, NF-kB subunit	10	Nucleus	transcription regulator
RELN	reelin	3	Extracellular Space	peptidase
REN	renin	3	Extracellular Space	peptidase
REST	RE1 silencing transcription factor	3	Nucleus	transcription regulator
RIF1	replication timing regulatory factor 1	3	Nucleus	other
RMDN3	regulator of microtubule dynamics 3	3	Cytoplasm	other
RNF11	ring finger protein 11	3	Cytoplasm	enzyme
RNF14	ring finger protein 14	3	Cytoplasm	transcription regulator
RNF19A	ring finger protein 19A, RBR E3 ubiquitin protein ligase	3	Nucleus	enzyme
RNF2	ring finger protein 2	13	Nucleus	transcription regulator

RNF4	ring finger protein 4	3	Nucleus	transcription regulator
RNF41	ring finger protein 41	4	Cytoplasm	enzyme
RNH1	ribonuclease/angiogenin inhibitor 1	4	Cytoplasm	other
RORA	RAR related orphan receptor A	3	Nucleus	lig-dependent nuc. receptor
RORC	RAR related orphan receptor C	5	Nucleus	lig-dependent nuc. receptor
RPA1	replication protein A1	9	Nucleus	other
RPA2	replication protein A2	10	Nucleus	other
RPA3	replication protein A3	8	Nucleus	other
RPGRIP1L	RPGRIP1 like	7	Cytoplasm	other
RPL10	ribosomal protein L10	5	Cytoplasm	translation regulator
RPL11	ribosomal protein L11	6	Cytoplasm	other
RPL12	ribosomal protein L12	5	Nucleus	other
RPL13	ribosomal protein L13	5	Nucleus	other
RPL21	ribosomal protein L21	3	Cytoplasm	other
RPL22	ribosomal protein L22	3	Nucleus	other
RPL23	ribosomal protein L23	5	Cytoplasm	other
RPL23A	ribosomal protein L23a	3	Cytoplasm	other
RPL26	ribosomal protein L26	3	Cytoplasm	other
RPL4	ribosomal protein L4	5	Cytoplasm	enzyme
RPL6	ribosomal protein L6	3	Nucleus	other
RPL7	ribosomal protein L7	3	Nucleus	transcription regulator
RPL9	ribosomal protein L9	3	Nucleus	other
RPLP0	ribosomal protein lateral stalk subunit P0	5	Cytoplasm	other
RPLP1	ribosomal protein lateral stalk subunit P1	3	Cytoplasm	other
RPS14	ribosomal protein S14	3	Cytoplasm	translation regulator
RPS2	ribosomal protein S2	3	Cytoplasm	other
RPS25	ribosomal protein S25	3	Cytoplasm	other
RPS27A	ribosomal protein S27a	3	Cytoplasm	other
RPS3	ribosomal protein S3	3	Cytoplasm	enzyme
RPS3A	ribosomal protein S3A	4	Nucleus	other
RPS4X	ribosomal protein S4, X-linked	3	Cytoplasm	other
RPS5	ribosomal protein S5	4	Cytoplasm	other
RPS6	ribosomal protein S6	4	Cytoplasm	other
RPS6KA1	ribosomal protein S6 kinase A1	3	Cytoplasm	kinase
RPS6KB2	ribosomal protein S6 kinase B2	4	Cytoplasm	kinase
RPS8	ribosomal protein S8	3	Cytoplasm	other
RPS9	ribosomal protein S9	3	Cytoplasm	translation regulator
RTCB	RNA 2',3'-cyclic phosphate and 5'-OH ligase	4	Cytoplasm	enzyme
RUNX1	runt related transcription factor 1	3	Nucleus	transcription regulator
RUNX2	runt related transcription factor 2	3	Nucleus	transcription regulator
RUVBL1	RuvB like AAA ATPase 1	3	Nucleus	transcription regulator
RUVBL2	RuvB like AAA ATPase 2	7	Nucleus	transcription regulator
RXRA	retinoid X receptor alpha	5	Nucleus	lig-dependent nuc. receptor
RXRB	retinoid X receptor beta	4	Nucleus	lig-dependent nuc. receptor
S100A8	S100 calcium binding protein A8	3	Cytoplasm	other

SAFB	scaffold attachment factor B	4	Nucleus	other
SAFB2	scaffold attachment factor B2	6	Cytoplasm	other
SCARB1	scavenger receptor class B member 1	3	Plasma Membrane	transporter
SCFD1	sec1 family domain containing 1	3	Cytoplasm	transporter
SCLT1	sodium channel and clathrin linker 1	3	Plasma Membrane	transporter
SDF4	stromal cell derived factor 4	3	Cytoplasm	other
SEC16A	SEC16 homolog A, endoplasmic reticulum export factor	4	Cytoplasm	phosphatase
SEC22B	SEC22 homolog B, vesicle trafficking protein (gene/pseudogene)	3	Cytoplasm	other
SHFM1	SEM1, 26S proteasome complex subunit	4	Nucleus	peptidase
SERBP1	SERPINE1 mRNA binding protein 1	3	Cytoplasm	other
SET	SET nuclear proto-oncogene	4	Nucleus	phosphatase
SETDB1	SET domain bifurcated 1	5	Nucleus	enzyme
SETX	senataxin	3	Nucleus	enzyme
SF1	splicing factor 1	5	Nucleus	transcription regulator
SF3A1	splicing factor 3a subunit 1	3	Nucleus	other
SF3A2	splicing factor 3a subunit 2	3	Nucleus	other
SFN	stratifin	3	Cytoplasm	other
SFPQ	splicing factor proline and glutamine rich	8	Nucleus	other
SGK1	serum/glucocorticoid regulated kinase 1	3	Cytoplasm	kinase
SH2D2A	SH2 domain containing 2A	4	Cytoplasm	other
SH3KBP1	SH3 domain containing kinase binding protein 1	3	Cytoplasm	other
SHC1	SHC adaptor protein 1	5	Cytoplasm	other
SHMT2	serine hydroxymethyltransferase 2	3	Cytoplasm	enzyme
SIAH1	siah E3 ubiquitin protein ligase 1	3	Nucleus	enzyme
SIK2	salt inducible kinase 2	3	Cytoplasm	kinase
SIN3A	SIN3 transcription regulator family member A	5	Nucleus	transcription regulator
SIN3B	SIN3 transcription regulator family member B	3	Nucleus	transcription regulator
SIRT1	sirtuin 1	11	Nucleus	transcription regulator
SIRT7	sirtuin 7	6	Nucleus	enzyme
SKAP1	src kinase associated phosphoprotein 1	6	Cytoplasm	kinase
SKI	SKI proto-oncogene	4	Nucleus	transcription regulator
SKP1	S-phase kinase associated protein 1	3	Nucleus	transcription regulator
SLC1A2	solute carrier family 1 member 2	3	Plasma Membrane	transporter
SLC25A3	solute carrier family 25 member 3	3	Cytoplasm	transporter
SLC25A4	solute carrier family 25 member 4	3	Cytoplasm	transporter
SLC25A5	solute carrier family 25 member 5	3	Cytoplasm	transporter
SMAD1	SMAD family member 1	7	Nucleus	transcription regulator
SMAD2	SMAD family member 2	6	Nucleus	transcription regulator
SMAD3	SMAD family member 3	5	Nucleus	transcription regulator
SMAD4	SMAD family member 4	4	Nucleus	transcription regulator
SMAD6	SMAD family member 6	3	Nucleus	transcription regulator
SMAD7	SMAD family member 7	3	Nucleus	transcription regulator
SMARCA2	SWI/SNF related, matrix associated, actin dependent regulator of chromatir	3	Nucleus	transcription regulator
SMARCA4	SWI/SNF related, matrix associated, actin dependent regulator of chromatir	3	Nucleus	transcription regulator
SMARCC1	SWI/SNF related, matrix associated, actin dependent regulator of chromatir	3	Nucleus	transcription regulator

SMARCE1	SWI/SNF related, matrix associated, actin dependent regulator of chromatir	3	Nucleus	transcription regulator
SMCR8	Smith-Magenis syndrome chromosome region, candidate 8	3	Nucleus	other
SMN1	survival of motor neuron 1, telomeric	8	Nucleus	other
SMURF1	SMAD specific E3 ubiquitin protein ligase 1	6	Cytoplasm	enzyme
SNAP25	synaptosome associated protein 25	5	Plasma Membrane	transporter
SNAP29	synaptosome associated protein 29	3	Cytoplasm	transporter
SNCA	synuclein alpha	9	Cytoplasm	enzyme
SNRNP70	small nuclear ribonucleoprotein U1 subunit 70	3	Nucleus	other
SNRPB	small nuclear ribonucleoprotein polypeptides B and B1	4	Nucleus	other
SNRPD1	small nuclear ribonucleoprotein D1 polypeptide	3	Nucleus	other
SNRPD2	small nuclear ribonucleoprotein D2 polypeptide	4	Nucleus	other
SNRPD3	small nuclear ribonucleoprotein D3 polypeptide	3	Nucleus	other
SNRPF	small nuclear ribonucleoprotein polypeptide F	3	Nucleus	other
SNW1	SNW domain containing 1	10	Nucleus	transcription regulator
SNX6	sorting nexin 6	4	Cytoplasm	transporter
SOCS6	suppressor of cytokine signaling 6	3	Cytoplasm	other
SOD1	superoxide dismutase 1	10	Cytoplasm	enzyme
SOD2	superoxide dismutase 2	3	Cytoplasm	enzyme
SORT1	sortilin 1	6	Plasma Membrane	G-protein coupled receptor
SOX2	SRY-box 2	7	Nucleus	transcription regulator
SOX5	SRY-box 5	3	Nucleus	transcription regulator
SOX9	SRY-box 9	5	Nucleus	transcription regulator
SP1	Sp1 transcription factor	8	Nucleus	transcription regulator
SP3	Sp3 transcription factor	3	Nucleus	transcription regulator
SPAST	spastin	5	Nucleus	enzyme
SPATA7	spermatogenesis associated 7	3	Cytoplasm	other
SPIB	Spi-B transcription factor	3	Nucleus	transcription regulator
SPICE1	spindle and centriole associated protein 1	3	Cytoplasm	other
SPRTN	SprT-like N-terminal domain	3	Nucleus	other
SPTAN1	spectrin alpha, non-erythrocytic 1	5	Plasma Membrane	other
SPTBN1	spectrin beta, non-erythrocytic 1	4	Plasma Membrane	other
SQSTM1	sequestosome 1	13	Cytoplasm	transcription regulator
SRC	SRC proto-oncogene, non-receptor tyrosine kinase	10	Cytoplasm	kinase
SRCIN1	SRC kinase signaling inhibitor 1	3	Cytoplasm	other
SRPK1	SRSF protein kinase 1	10	Nucleus	kinase
SRPK2	SRSF protein kinase 2	5	Nucleus	kinase
SRRM2	serine/arginine repetitive matrix 2	6	Nucleus	other
SRRT	serrate, RNA effector molecule	3	Nucleus	other
SRSF1	serine and arginine rich splicing factor 1	7	Nucleus	other
SRSF2	serine and arginine rich splicing factor 2	5	Nucleus	transcription regulator
ST13	ST13, Hsp70 interacting protein	4	Cytoplasm	other
STAT1	signal transducer and activator of transcription 1	9	Nucleus	transcription regulator
STAT3	signal transducer and activator of transcription 3	6	Nucleus	transcription regulator
STAT4	signal transducer and activator of transcription 4	3	Nucleus	transcription regulator
STAT6	signal transducer and activator of transcription 6	6	Nucleus	transcription regulator

STAU1	staufen double-stranded RNA binding protein 1	9	Cytoplasm	transporter
STK4	serine/threonine kinase 4	4	Cytoplasm	kinase
STRAP	serine/threonine kinase receptor associated protein	3	Plasma Membrane	other
STUB1	STIP1 homology and U-box containing protein 1	7	Cytoplasm	enzyme
STX12	syntaxin 12	3	Cytoplasm	other
STX18	syntaxin 18	3	Cytoplasm	transporter
STX1A	syntaxin 1A	4	Cytoplasm	transporter
STX1B	syntaxin 1B	4	Plasma Membrane	other
STX4	syntaxin 4	3	Plasma Membrane	transporter
STX5	syntaxin 5	5	Cytoplasm	transporter
STX6	syntaxin 6	3	Cytoplasm	transporter
STXBP1	syntaxin binding protein 1	6	Cytoplasm	transporter
STXBP5L	syntaxin binding protein 5 like	5	Cytoplasm	other
SUGP2	SURP and G-patch domain containing 2	3	Nucleus	other
SUMO1	small ubiquitin-like modifier 1	7	Nucleus	enzyme
SUMO2	small ubiquitin-like modifier 2	3	Nucleus	enzyme
SUMO3	small ubiquitin-like modifier 3	9	Nucleus	other
SUZ12	SUZ12 polycomb repressive complex 2 subunit	7	Nucleus	enzyme
SYK	spleen associated tyrosine kinase	5	Cytoplasm	kinase
SYNCRIP	synaptotagmin binding cytoplasmic RNA interacting protein	9	Nucleus	other
SYVN1	synoviolin 1	5	Cytoplasm	transporter
TAF15	TATA-box binding protein associated factor 15	6	Nucleus	other
TAF4	TATA-box binding protein associated factor 4	4	Nucleus	transcription regulator
TAF7	TATA-box binding protein associated factor 7	3	Nucleus	transcription regulator
TARDBP	TAR DNA binding protein	10	Nucleus	transcription regulator
TAX1BP1	Tax1 binding protein 1	3	Cytoplasm	other
TAZ	tafazzin	3	Nucleus	enzyme
TBK1	TANK binding kinase 1	5	Cytoplasm	kinase
T	T-box transcription factor T	3	Nucleus	transcription regulator
TCF3	transcription factor 3	6	Nucleus	transcription regulator
TCF7	transcription factor 7	3	Nucleus	transcription regulator
TCF7L2	transcription factor 7 like 2	5	Nucleus	transcription regulator
TCP1	t-complex 1	6	Cytoplasm	other
TCTN3	tectonic family member 3	3	Cytoplasm	other
TDG	thymine DNA glycosylase	4	Nucleus	enzyme
TDP2	tyrosyl-DNA phosphodiesterase 2	3	Cytoplasm	transcription regulator
TEKT4	tektin 4	3	Nucleus	transporter
TERF1	telomeric repeat binding factor 1	4	Nucleus	other
TERF2	telomeric repeat binding factor 2	3	Nucleus	other
TERF2IP	TERF2 interacting protein	4	Nucleus	other
TERT	telomerase reverse transcriptase	3	Nucleus	enzyme
TFAP2A	transcription factor AP-2 alpha	5	Nucleus	transcription regulator
TFAP2C	transcription factor AP-2 gamma	4	Nucleus	transcription regulator
TFEB	transcription factor EB	6	Nucleus	transcription regulator
TFG	TRK-fused gene	3	Cytoplasm	other

TGFB1	transforming growth factor beta 1	3	Extracellular Space	growth factor
TGM2	transglutaminase 2	3	Cytoplasm	enzyme
THRAP3	thyroid hormone receptor associated protein 3	3	Nucleus	transcription regulator
TIA1	TIA1 cytotoxic granule associated RNA binding protein	3	Nucleus	other
TJP1	tight junction protein 1	4	Plasma Membrane	other
TK1	thymidine kinase 1	3	Cytoplasm	kinase
TLE1	transducin like enhancer of split 1	3	Nucleus	transcription regulator
TMED2	transmembrane p24 trafficking protein 2	4	Cytoplasm	transporter
TMEM17	transmembrane protein 17	4	Extracellular Space	other
TMEM216	transmembrane protein 216	4	Extracellular Space	other
TNF	tumor necrosis factor	7	Extracellular Space	cytokine
TNFRSF10B	TNF receptor superfamily member 10b	3	Plasma Membrane	transmembrane receptor
TNFRSF1A	TNF receptor superfamily member 1A	3	Plasma Membrane	transmembrane receptor
TNIK	TRAF2 and NCK interacting kinase	5	Plasma Membrane	kinase
TNIP1	TNFAIP3 interacting protein 1	4	Nucleus	other
TNK1	tyrosine kinase non receptor 1	3	Cytoplasm	kinase
TNKS2	tankyrase 2	3	Nucleus	enzyme
TOB1	transducer of ERBB2, 1	3	Nucleus	transcription regulator
TOP1	DNA topoisomerase I	6	Nucleus	enzyme
TP53	tumor protein p53	26	Nucleus	transcription regulator
TP53BP1	tumor protein p53 binding protein 1	4	Nucleus	transcription regulator
TP63	tumor protein p63	4	Nucleus	transcription regulator
TP73	tumor protein p73	3	Nucleus	transcription regulator
TRAF1	TNF receptor associated factor 1	4	Cytoplasm	other
TRAF2	TNF receptor associated factor 2	6	Cytoplasm	enzyme
TRAF6	TNF receptor associated factor 6	11	Cytoplasm	enzyme
TRIB3	tribbles pseudokinase 3	3	Nucleus	kinase
TRIM24	tripartite motif containing 24	3	Nucleus	transcription regulator
TRIM27	tripartite motif containing 27	3	Nucleus	transcription regulator
TRIM28	tripartite motif containing 28	7	Nucleus	transcription regulator
TRIP6	thyroid hormone receptor interactor 6	3	Extracellular Space	cytokine
TSC22D1	TSC22 domain family member 1	3	Nucleus	transcription regulator
TSG101	tumor susceptibility 101	7	Cytoplasm	transcription regulator
TSPAN3	tetraspanin 3	3	Plasma Membrane	other
TTF2	transcription termination factor 2	4	Cytoplasm	transcription regulator
TUBA1A	tubulin alpha 1a	13	Cytoplasm	other
TUBA1B	tubulin alpha 1b	6	Cytoplasm	other
TUBA1C	tubulin alpha 1c	8	Cytoplasm	other
TUBA4A	tubulin alpha 4a	4	Cytoplasm	other
TUBB	tubulin beta class I	10	Cytoplasm	other
TUBB2A	tubulin beta 2A class IIa	7	Cytoplasm	other
TUBB2B	tubulin beta 2B class IIb	4	Cytoplasm	other
TUBB3	tubulin beta 3 class III	6	Cytoplasm	other
TUBB4A	tubulin beta 4A class IVa	4	Cytoplasm	other
TUBB4B	tubulin beta 4B class IVb	4	Cytoplasm	other

TUBG1	tubulin gamma 1	4	Cytoplasm	other
TWIST1	twist family bHLH transcription factor 1	3	Nucleus	transcription regulator
TXN	thioredoxin	3	Cytoplasm	enzyme
U2AF2	U2 small nuclear RNA auxiliary factor 2	3	Nucleus	other
UBA5	ubiquitin like modifier activating enzyme 5	4	Cytoplasm	enzyme
UBB	ubiquitin B	4	Cytoplasm	enzyme
UBE2I	ubiquitin conjugating enzyme E2 I	8	Nucleus	enzyme
UBE3A	ubiquitin protein ligase E3A	3	Nucleus	enzyme
UBL4A	ubiquitin like 4A	7	Cytoplasm	enzyme
UBQLN1	ubiquilin 1	7	Cytoplasm	other
UBQLN2	ubiquilin 2	4	Nucleus	other
UBR2	ubiquitin protein ligase E3 component n-recognin 2	3	Nucleus	enzyme
UBR5	ubiquitin protein ligase E3 component n-recognin 5	4	Nucleus	enzyme
UBXN1	UBX domain protein 1	3	Cytoplasm	other
UFL1	UFM1 specific ligase 1	3	Cytoplasm	enzyme
ULK1	unc-51 like autophagy activating kinase 1	3	Cytoplasm	kinase
UNC119	unc-119 lipid binding chaperone	3	Cytoplasm	other
UNK	unkempt family zinc finger	6	Nucleus	transporter
UPF1	UPF1, RNA helicase and ATPase	3	Nucleus	enzyme
USP10	ubiquitin specific peptidase 10	3	Cytoplasm	peptidase
USP12	ubiquitin specific peptidase 12	3	Cytoplasm	peptidase
USP7	ubiquitin specific peptidase 7	5	Nucleus	peptidase
USP8	ubiquitin specific peptidase 8	4	Cytoplasm	peptidase
USP9X	ubiquitin specific peptidase 9, X-linked	3	Plasma Membrane	peptidase
UXT	ubiquitously expressed prefoldin like chaperone	4	Cytoplasm	transcription regulator
VAMP2	vesicle associated membrane protein 2	4	Plasma Membrane	other
VAPA	VAMP associated protein A	5	Plasma Membrane	other
VAPB	VAMP associated protein B and C	5	Plasma Membrane	other
VAV2	vav guanine nucleotide exchange factor 2	5	Cytoplasm	transcription regulator
VCAM1	vascular cell adhesion molecule 1	9	Plasma Membrane	transmembrane receptor
VCL	vinculin	8	Plasma Membrane	enzyme
VCP	valosin containing protein	16	Cytoplasm	enzyme
VEGFA	vascular endothelial growth factor A	5	Extracellular Space	growth factor
VHL	von Hippel-Lindau tumor suppressor	20	Nucleus	transcription regulator
VIM	vimentin	6	Cytoplasm	other
VPS26B	VPS26, retromer complex component B	4	Cytoplasm	transporter
VPS28	VPS28, ESCRT-I subunit	3	Cytoplasm	transporter
VTN	vitronectin	5	Extracellular Space	other
WDR7	WD repeat domain 7	3	Cytoplasm	other
WDR77	WD repeat domain 77	4	Nucleus	transcription regulator
WDYHV1	WDYHV motif containing 1	3	Cytoplasm	enzyme
WNK1	WNK lysine deficient protein kinase 1	4	Cytoplasm	kinase
WT1	Wilms tumor 1	4	Nucleus	transcription regulator
WWOX	WW domain containing oxidoreductase	9	Cytoplasm	enzyme
XIAP	X-linked inhibitor of apoptosis	4	Cytoplasm	enzyme

XPC	XPC complex subunit, DNA damage recognition and repair factor	3	Nucleus	other
XPO1	exportin 1	12	Nucleus	transporter
XRCC5	X-ray repair cross complementing 5	8	Nucleus	enzyme
XRCC6	X-ray repair cross complementing 6	5	Nucleus	enzyme
XRN2	5'-3' exoribonuclease 2	8	Nucleus	enzyme
YAP1	Yes associated protein 1	5	Nucleus	transcription regulator
YBX1	Y-box binding protein 1	7	Nucleus	transcription regulator
YBX3	Y-box binding protein 3	3	Nucleus	transcription regulator
YES1	YES proto-oncogene 1, Src family tyrosine kinase	3	Cytoplasm	kinase
YLPM1	YLP motif containing 1	5	Nucleus	transcription regulator
YTHDF2	YTH N6-methyladenosine RNA binding protein 2	3	Cytoplasm	other
YWHAB	tyrosine 3-monooxygenase/tryptophan 5-monooxygenase activation protein	6	Cytoplasm	transcription regulator
YWHAE	tyrosine 3-monooxygenase/tryptophan 5-monooxygenase activation protein	11	Cytoplasm	other
YWHAG	tyrosine 3-monooxygenase/tryptophan 5-monooxygenase activation protein	9	Cytoplasm	other
YWHAH	tyrosine 3-monooxygenase/tryptophan 5-monooxygenase activation protein	7	Cytoplasm	transcription regulator
YWHAQ	tyrosine 3-monooxygenase/tryptophan 5-monooxygenase activation protein	14	Cytoplasm	other
YWHAZ	tyrosine 3-monooxygenase/tryptophan 5-monooxygenase activation protein	16	Cytoplasm	enzyme
YY1	YY1 transcription factor	7	Nucleus	transcription regulator
ZBTB16	zinc finger and BTB domain containing 16	5	Nucleus	transcription regulator
ZBTB7A	zinc finger and BTB domain containing 7A	3	Nucleus	transcription regulator
ZFR	zinc finger RNA binding protein	3	Nucleus	other
ZFYVE27	zinc finger FYVE-type containing 27	3	Plasma Membrane	other
ZMYM2	zinc finger MYM-type containing 2	3	Nucleus	kinase
ZNF202	zinc finger protein 202	3	Nucleus	transcription regulator
ZNF326	zinc finger protein 326	4	Nucleus	transcription regulator
ZNF638	zinc finger protein 638	3	Nucleus	other

Supplementary Table 4

Comparison of p values with Fisher Exact Test and Benjamini-Hochberg method of multiple testing correction. The table includes the canonical pathways identified to be significant by IPA, and the p values obtained using two different tests.

	Fisher Exact test values		B-H Multiple test values	
	p value	ratio	p value	ratio
CYTOSKELETAL DYNAMICS				
ILK Signaling	3.69E-27	62/177	1.79E-25	62/177
HIPPO Signaling	4.54E-14	30/84	1.35E-12	30/84
Integrin Signaling	2.65E-11	49/200	1.01E-12	49/200
FAK signaling	6.35E-11	28/95	9.38E-10	28/95
Paxillin Signaling	1.66E-10	29/105	2.33E-09	29/105
Actin cytoskeleton Signaling	1.75E-08	41/205	1.01E-10	41/205
Ephrin receptor Signaling	2.68E-08	34/167	9.55E-08	34/167
GROWTH FACTORS				
Neureglin Signaling	1.27E-26	44/82	7.62E-26	44/82
PTEN signaling	2.87E-23	46/115	1.30E-20	46/115
VEGF signaling	8.72E-21	40/98	1.50E-18	40/98
NGF signaling	7.31E-19	41/114	1.50E-17	41/114
HGF signaling	8.46E-19	40/109	9.45E-17	40/109
IGF-1 signaling	3.93E-17	37/103	7.49E-17	37/103
EGF signaling	2.86E-15	28/67	1.02E-13	28/67
Insulin receptor signaling	1.03E-14	39/132	1.06E-13	39/132
PDGF signaling	1.18E-13	31/87	6.21E-13	31/87
GNRH signaling	1.07E-13	41/154	1.09E-12	41/154
TGF-b signaling	1.61E-13	29/82	6.80E-13	29/82
PEDF signaling	4.66E-13	29/85	1.87E-12	29/85
Neurotropin/ TRK signaling	9.80E-13	27/76	1.88E-11	27/76
GH signaling	2.11E-12	28/79	8.61E-12	28/79
FGF signaling	1.64E-10	26/86	4.20E-10	26/86
GDNF signaling	1.01E-09	23/74	9.70E-09	23/74
CNTF signaling	6.81E-07	18/58	4.02E-07	18/58
RESPONSE FOR HYPOXIC INSULT				
Production of nitric oxide and ROS	1.88E-22	55/169	1.85E-20	55/169
NRF2-mediated oxidative stress	4.15E-04	45/177	2.21E-12	45/177
iNOS signaling	6.78E-12	20/45	8.43E-11	20/45
Hypoxic signaling	1.22E-11	25/72	1.83E-10	25/72
HIF1a signaling	3.15E-09	27/104	7.91E-09	27/104

DNA DAMAGE AND REPAIR				
Telomerase signaling	2.35E-21	42/105	5.25E-19	42/105
ATM signaling	4.05E-18	36/92	3.43E-16	36/92
UVA-induced MAPK signaling	4.07E-10	28/96	1.21E-09	28/96
Telomerase extension by Telomerase	7.34E-08	9/14	2.81E-07	9/14
GADD45 signaling	1.85E-07	10/19	7.18E-07	10/19
DNA damage induce 14-3-3 signaling	2.17E-05	8/18	4.51E-05	8/18
DNA DS break repaired by homologues recombination	3.40E-05	7/14	5.64E-05	7/14
DNA DS break repaired by non homologues end joining	2.17E-05	7/14	5.64E-05	7/14
LIPID HOMEOSTASIS				
PPAR signaling	2.75E-21	38/86	8.44E-21	38/86
PPARa/RxR activation	7.08E-19	49/160	5.18E-18	49/160
Adipogenesis pathway	6.40E-17	41/127	3.27E-17	41/127
TX/RxR activation	1.49E-09	29/64	3.32E-09	29/64
Docosahexaenoic acid (DHA) signaling	1.65E-07	16/49	8.15E-07	16/49
PROTEIN HOMEOSTASIS				
EIF2 signaling	9.09E-28	68/207	4.12E-25	68/207
p70S6K protein response	6.35E-21	45/123	1.44E-18	45/123
Sumolytion pathway	8.28E-21	39/93	1.91E-19	39/93
Unfolded protein response	1.20E-12	23/51	2.71E-13	23/51
Protein ubiquitination pathway	2.30E-12	51/242	9.91E-12	51/242
ER stress pathway	3.45E-07	10/20	1.28E-06	10/20

Supplementary Table 5

Canonical pathways and proteins involved in lipid homeostasis. The table includes, gene ID, Entrez gene name, their total number of protein interactions, location of the protein and the protein type.

CANONICAL PATHWAYS RELATED TO LIPID HOMEOSTASIS

PPAR SIGNALING

Symbol	Entrez Gene Name	Expr Other	Expected	Location	Type(s)
CREBBP	CREB binding protein	8	Up	Nucleus	transcription regulator
EP300	E1A binding protein p300	12	Up	Nucleus	transcription regulator
FOS	Fos proto-oncogene, AP-1 transcription factor subunit	7	Down	Nucleus	transcription regulator
GRB2	growth factor receptor bound protein 2	19	Down	Cytoplasm	kinase
HSP90AA1	heat shock protein 90 alpha family class A member 1	11	Down	Cytoplasm	enzyme
HSP90AB1	heat shock protein 90 alpha family class B member 1	9	Down	Cytoplasm	enzyme
HSP90B1	heat shock protein 90 beta family member 1	5	Down	Cytoplasm	other
IKBKE	inhibitor of nuclear factor kappa B kinase subunit epsilon	4	Down	Cytoplasm	kinase
IKBKG	inhibitor of nuclear factor kappa B kinase subunit gamma	4	Down	Nucleus	kinase
INSR	insulin receptor	6	Down	Plasma Membrane	kinase
JUN	Jun proto-oncogene, AP-1 transcription factor subunit	12	Down	Nucleus	transcription regulator
MAP2K1	mitogen-activated protein kinase kinase 1	3	Down	Cytoplasm	kinase
MAP3K7	mitogen-activated protein kinase kinase kinase 7	3	Down	Cytoplasm	kinase
MAPK1	mitogen-activated protein kinase 1	9	Down	Cytoplasm	kinase
MAPK3	mitogen-activated protein kinase 3	16	Down	Cytoplasm	kinase
NCOA1	nuclear receptor coactivator 1	3	Up	Nucleus	transcription regulator
NCOR1	nuclear receptor corepressor 1	6	Down	Nucleus	transcription regulator
NCOR2	nuclear receptor corepressor 2	4	Down	Nucleus	transcription regulator
NFKB1	nuclear factor kappa B subunit 1	6	Down	Nucleus	transcription regulator
NFKB2	nuclear factor kappa B subunit 2	4	Down	Nucleus	transcription regulator
NFKBIA	NFKB inhibitor alpha	14	Down	Cytoplasm	transcription regulator
NGFR	nerve growth factor receptor	5	Down	Plasma Membrane	transmembrane receptor
NR1H3	nuclear receptor subfamily 1 group H member 3	3	Down	Nucleus	lig-dependent nuc. receptor
NR2F1	nuclear receptor subfamily 2 group F member 1	3		Nucleus	lig-dependent nuc. receptor
PDGFRA	platelet derived growth factor receptor alpha	3	Down	Plasma Membrane	kinase
PPARA	peroxisome proliferator activated receptor alpha	3	Up	Nucleus	lig-dependent nuc. receptor
PPARG	peroxisome proliferator activated receptor gamma	6	Up	Nucleus	lig-dependent nuc. receptor
PPARGC1A	PPARG coactivator 1 alpha	8	Up	Nucleus	transcription regulator
PTGS2	prostaglandin-endoperoxide synthase 2	3	Down	Cytoplasm	enzyme
RAF1	Raf-1 proto-oncogene, serine/threonine kinase	8	Down	Cytoplasm	kinase
RELA	RELA proto-oncogene, NF-kB subunit	10	Down	Nucleus	transcription regulator
RXRA	retinoid X receptor alpha	5	Up	Nucleus	lig-dependent nuc. receptor
SHC1	SHC adaptor protein 1	5	Down	Cytoplasm	other
SNW1	SNW domain containing 1	10	Up	Nucleus	transcription regulator
TNF	tumor necrosis factor	7	Down	Extracellular Space	cytokine
TNFRSF1A	TNF receptor superfamily member 1A	3	Down	Plasma Membrane	transmembrane receptor
TRAF2	TNF receptor associated factor 2	6	Down	Cytoplasm	enzyme
TRAF6	TNF receptor associated factor 6	11	Down	Cytoplasm	enzyme

PPARA/RXRA ACTIVATION					
**Symbol**	**Entrez Gene Name**	**Expr Other**	**Expected**	**Location**	**Type(s)**
ABCA1	ATP binding cassette subfamily A member 1	4	Up	Plasma Membrane	transporter
APOA1	apolipoprotein A1	7	Up	Extracellular Space	transporter
CAND1	cullin associated and neddylation dissociated 1	12		Cytoplasm	transcription regulator
CKAP5	cytoskeleton associated protein 5	4		Nucleus	transcription regulator
CREBBP	CREB binding protein	8	Down	Nucleus	transcription regulator
EP300	E1A binding protein p300	12	Down	Nucleus	transcription regulator
GRB2	growth factor receptor bound protein 2	19	Down	Cytoplasm	kinase
HSP90AA1	heat shock protein 90 alpha family class A member 1	11	Down	Cytoplasm	enzyme
HSP90AB1	heat shock protein 90 alpha family class B member 1	9	Down	Cytoplasm	enzyme
HSP90B1	heat shock protein 90 beta family member 1	5	Down	Cytoplasm	other
IKBKE	inhibitor of nuclear factor kappa B kinase subunit epsilon	4	Down	Cytoplasm	kinase
IKBKG	inhibitor of nuclear factor kappa B kinase subunit gamma	4	Down	Nucleus	kinase
IL6	interleukin 6	4	Down	Extracellular Space	cytokine
INSR	insulin receptor	6	Down	Plasma Membrane	kinase
JUN	Jun proto-oncogene, AP-1 transcription factor subunit	12	Down	Nucleus	transcription regulator
MAP2K1	mitogen-activated protein kinase kinase 1	3	Down	Cytoplasm	kinase
MAP3K7	mitogen-activated protein kinase kinase kinase 7	3	Down	Cytoplasm	kinase
MAPK1	mitogen-activated protein kinase 1	9	Down	Cytoplasm	kinase
MAPK3	mitogen-activated protein kinase 3	16	Down	Cytoplasm	kinase
MAPK8	mitogen-activated protein kinase 8	5	Down	Cytoplasm	kinase
MED23	mediator complex subunit 23	3		Nucleus	transcription regulator
MED24	mediator complex subunit 24	4		Nucleus	transcription regulator
NCOA3	nuclear receptor coactivator 3	4	Down	Nucleus	transcription regulator
NCOA6	nuclear receptor coactivator 6	5		Nucleus	transcription regulator
NCOR1	nuclear receptor corepressor 1	6	Down	Nucleus	transcription regulator
NCOR2	nuclear receptor corepressor 2	4	Down	Nucleus	transcription regulator
NFKB1	nuclear factor kappa B subunit 1	6	Down	Nucleus	transcription regulator
NFKB2	nuclear factor kappa B subunit 2	4	Down	Nucleus	transcription regulator
NFKBIA	NFKB inhibitor alpha	14		Cytoplasm	transcription regulator
NR2F1	nuclear receptor subfamily 2 group F member 1	3	Down	Nucleus	lig-dependent nuc. receptor
PDIA3	protein disulfide isomerase family A member 3	4	Down	Cytoplasm	peptidase
PLCB1	phospholipase C beta 1	4	Down	Cytoplasm	enzyme
PLCG1	phospholipase C gamma 1	4	Down	Cytoplasm	enzyme
PLCG2	phospholipase C gamma 2	3	Down	Cytoplasm	enzyme
PPARA	peroxisome proliferator activated receptor alpha	3	Up	Nucleus	lig-dependent nuc. receptor
PPARGC1A	PPARG coactivator 1 alpha	8	Up	Nucleus	transcription regulator

PRKAA1	protein kinase AMP-activated catalytic subunit alpha 1	3	Up	Cytoplasm	kinase
PRKACA	protein kinase cAMP-activated catalytic subunit alpha	3	Up	Cytoplasm	kinase
PRKCA	protein kinase C alpha	3	Up	Cytoplasm	kinase
PRKCB	protein kinase C beta	5	Up	Cytoplasm	kinase
RAF1	Raf-1 proto-oncogene, serine/threonine kinase	8	Down	Cytoplasm	kinase
RELA	RELA proto-oncogene, NF-kB subunit	10	Down	Nucleus	transcription regulator
RXRA	retinoid X receptor alpha	5	Up	Nucleus	lig-dependent nuc. receptor
SHC1	SHC adaptor protein 1	5	Down	Cytoplasm	other
SMAD2	SMAD family member 2	6	Down	Nucleus	transcription regulator
SMAD3	SMAD family member 3	5	Down	Nucleus	transcription regulator
SMAD4	SMAD family member 4	4	Down	Nucleus	transcription regulator
TGFB1	transforming growth factor beta 1	3	Down	Extracellular Space	growth factor
TRAF6	TNF receptor associated factor 6	11	Down	Cytoplasm	enzyme

ADIPOGENESIS PATHWAY

Symbol	Entrez Gene Name	Expr Other	Expected	Location	Type(s)
AKT1	AKT serine/threonine kinase 1	7		Cytoplasm	kinase
ATG5	autophagy related 5	3		Cytoplasm	other
BMPR1A	bone morphogenetic protein receptor type 1A	5		Plasma Membrane	kinase
CDK5	cyclin dependent kinase 5	6		Nucleus	kinase
CEBPA	CCAAT/enhancer binding protein alpha	7		Nucleus	transcription regulator
CTNNB1	catenin beta 1	17		Nucleus	transcription regulator
EGR2	early growth response 2	3		Nucleus	transcription regulator
ERCC2	ERCC excision repair 2, TFIIH core complex helicase subunit	3		Nucleus	enzyme
EZH2	enhancer of zeste 2 polycomb repressive complex 2 subunit	10		Nucleus	transcription regulator
FBXW7	F-box and WD repeat domain containing 7	3		Nucleus	transcription regulator
FGFR1	fibroblast growth factor receptor 1	4		Plasma Membrane	kinase
FOXO1	forkhead box O1	4		Nucleus	transcription regulator
HDAC1	histone deacetylase 1	14		Nucleus	transcription regulator
HDAC2	histone deacetylase 2	6		Nucleus	transcription regulator
HDAC3	histone deacetylase 3	4		Nucleus	transcription regulator
HDAC4	histone deacetylase 4	6		Nucleus	transcription regulator
HDAC5	histone deacetylase 5	5		Nucleus	transcription regulator
HDAC6	histone deacetylase 6	13		Nucleus	transcription regulator
HDAC7	histone deacetylase 7	3		Nucleus	transcription regulator
HIF1A	hypoxia inducible factor 1 alpha subunit	9		Nucleus	transcription regulator
KLF3	Kruppel like factor 3	7		Nucleus	transcription regulator
KMT2B	lysine methyltransferase 2B	4		Nucleus	transcription regulator
LEP	leptin	3		Extracellular Space	growth factor

NR2F2	nuclear receptor subfamily 2 group F member 2	4		Nucleus	lig-dependent nuc. receptor
PPARG	peroxisome proliferator activated receptor gamma	6		Nucleus	lig-dependent nuc. receptor
RB1	RB transcriptional corepressor 1	5		Nucleus	transcription regulator
RBBP4	RB binding protein 4, chromatin remodeling factor	6		Nucleus	enzyme
RBBP7	RB binding protein 7, chromatin remodeling factor	4		Nucleus	transcription regulator
RPS6KA1	ribosomal protein S6 kinase A1	3		Cytoplasm	kinase
SETDB1	SET domain bifurcated 1	5		Nucleus	enzyme
SIN3A	SIN3 transcription regulator family member A	5		Nucleus	transcription regulator
SIN3B	SIN3 transcription regulator family member B	3		Nucleus	transcription regulator
SIRT1	sirtuin 1	11		Nucleus	transcription regulator
SMAD1	SMAD family member 1	7		Nucleus	transcription regulator
SMAD3	SMAD family member 3	5		Nucleus	transcription regulator
SOX9	SRY-box 9	5		Nucleus	transcription regulator
TCF7	transcription factor 7	3		Nucleus	transcription regulator
TGFB1	transforming growth factor beta 1	3		Extracellular Space	growth factor
TNF	tumor necrosis factor	7		Extracellular Space	cytokine
TNFRSF1A	TNF receptor superfamily member 1A	3		Plasma Membrane	transmembrane receptor
TP53	tumor protein p53	26		Nucleus	transcription regulator

TR/RXR ACTIVATION

Symbol	Entrez Gene Name	Expr Other	Expected	Location	Type(s)
ACACA	acetyl-CoA carboxylase alpha	5		Cytoplasm	enzyme
AKT1	AKT serine/threonine kinase 1	7		Cytoplasm	kinase
ATM	ATM serine/threonine kinase	5		Nucleus	kinase
ENO1	enolase 1	5		Cytoplasm	enzyme
EP300	E1A binding protein p300	12		Nucleus	transcription regulator
FGFR1	fibroblast growth factor receptor 1	4		Plasma Membrane	kinase
GRB2	growth factor receptor bound protein 2	19		Cytoplasm	kinase
HDAC3	histone deacetylase 3	4		Nucleus	transcription regulator
HIF1A	hypoxia inducible factor 1 alpha subunit	9		Nucleus	transcription regulator
MDM2	MDM2 proto-oncogene	7		Nucleus	transcription regulator
MTOR	mechanistic target of rapamycin kinase	3		Nucleus	kinase
NCOA1	nuclear receptor coactivator 1	3		Nucleus	transcription regulator
NCOA2	nuclear receptor coactivator 2	4		Nucleus	transcription regulator
NCOA3	nuclear receptor coactivator 3	4		Nucleus	transcription regulator
NCOA4	nuclear receptor coactivator 4	4		Nucleus	transcription regulator
NCOA6	nuclear receptor coactivator 6	5		Nucleus	transcription regulator
NCOR1	nuclear receptor corepressor 1	6		Nucleus	transcription regulator

NCOR2	nuclear receptor corepressor 2	4		Nucleus	transcription regulator
PFKP	phosphofructokinase, platelet	4		Cytoplasm	kinase
PIK3R1	phosphoinositide-3-kinase regulatory subunit 1	3		Cytoplasm	kinase
PIK3R2	phosphoinositide-3-kinase regulatory subunit 2	6		Cytoplasm	kinase
PPARGC1A	PPARG coactivator 1 alpha	8		Nucleus	transcription regulator
PTPN11	protein tyrosine phosphatase, non-receptor type 11	3		Cytoplasm	phosphatase
RXRA	retinoid X receptor alpha	5		Nucleus	lig-dependent nuc. receptor
RXRB	retinoid X receptor beta	4		Nucleus	lig-dependent nuc. receptor
SCARB1	scavenger receptor class B member 1	3		Plasma Membrane	transporter

DHA SIGNALING

Symbol	Entrez Gene Name	Expr Other	Expected	Location	Type(s)
AKT1	AKT serine/threonine kinase 1	7		Cytoplasm	kinase
APP	amyloid beta precursor protein	19		Plasma Membrane	other
ATM	ATM serine/threonine kinase	5		Nucleus	kinase
BAX	BCL2 associated X, apoptosis regulator	3		Cytoplasm	transporter
BCL2	BCL2, apoptosis regulator	6		Cytoplasm	transporter
BCL2L1	BCL2 like 1	4		Cytoplasm	other
CASP3	caspase 3	10		Cytoplasm	peptidase
CASP9	caspase 9	5		Cytoplasm	peptidase
FGFR1	fibroblast growth factor receptor 1	4		Plasma Membrane	kinase
FOXO1	forkhead box O1	4		Nucleus	transcription regulator
GRB2	growth factor receptor bound protein 2	19		Cytoplasm	kinase
GSK3B	glycogen synthase kinase 3 beta	11		Nucleus	kinase
PDPK1	3-phosphoinositide dependent protein kinase 1	3		Cytoplasm	kinase
PIK3R1	phosphoinositide-3-kinase regulatory subunit 1	3		Cytoplasm	kinase
PIK3R2	phosphoinositide-3-kinase regulatory subunit 2	6		Cytoplasm	kinase
PTPN11	protein tyrosine phosphatase, non-receptor type 11	3		Cytoplasm	phosphatase

Supplementary Table 6

Canonical pathways and proteins involved in protein homeostasis. The table includes, gene ID, Entrez Gene name, their total number of protein interactions, location of the protein and the protein type.

CANONICAL PATHWAYS RELATED TO PROTEIN HOMEOSTASIS					
**EIF2 SIGNALING**					
**Symbol**	**Entrez Gene Name**	**Expr Other**	**Expected**	**Location**	**Type(s)**
ACTA1	actin, alpha 1, skeletal muscle	4		Cytoplasm	other
ACTB	actin beta	13		Cytoplasm	other
AGO1	argonaute 1, RISC catalytic component	3		Cytoplasm	translation regulator
AGO2	argonaute 2, RISC catalytic component	5		Cytoplasm	translation regulator
AKT1	AKT serine/threonine kinase 1	7	Up	Cytoplasm	kinase
ATF4	activating transcription factor 4	6	Down	Nucleus	transcription regulator
ATF5	activating transcription factor 5	3	Down	Nucleus	transcription regulator
ATM	ATM serine/threonine kinase	5	Up	Nucleus	kinase
BCL2	BCL2, apoptosis regulator	6	Up	Cytoplasm	transporter
CCND1	cyclin D1	6	Up	Nucleus	transcription regulator
EIF2AK2	eukaryotic translation initiation factor 2 alpha kinase 2	3	Up	Cytoplasm	kinase
EIF2B1	eukaryotic translation initiation factor 2B subunit alpha	3	Up	Cytoplasm	translation regulator
EIF2B2	eukaryotic translation initiation factor 2B subunit beta	3	Up	Cytoplasm	other
EIF2S3	eukaryotic translation initiation factor 2 subunit gamma	3		Cytoplasm	translation regulator
EIF3E	eukaryotic translation initiation factor 3 subunit E	3		Cytoplasm	other
EIF3H	eukaryotic translation initiation factor 3 subunit H	3		Cytoplasm	other
EIF4A2	eukaryotic translation initiation factor 4A2	4		Cytoplasm	translation regulator
FGFR1	fibroblast growth factor receptor 1	4	Up	Plasma Membrane	kinase
GRB2	growth factor receptor bound protein 2	19	Up	Cytoplasm	kinase
GSK3B	glycogen synthase kinase 3 beta	11	Down	Nucleus	kinase
HNRNPA1	heterogeneous nuclear ribonucleoprotein A1	15		Nucleus	enzyme
HSPA5	heat shock protein family A (Hsp70) member 5	14	Down	Cytoplasm	enzyme
INSR	insulin receptor	6	Up	Plasma Membrane	kinase
MAP2K1	mitogen-activated protein kinase kinase 1	3	Up	Cytoplasm	kinase
MAPK1	mitogen-activated protein kinase 1	9	Up	Cytoplasm	kinase
MAPK3	mitogen-activated protein kinase 3	16	Up	Cytoplasm	kinase
MYC	MYC proto-oncogene, bHLH transcription factor	18	Down	Nucleus	transcription regulator
MYCN	MYCN proto-oncogene, bHLH transcription factor	3	Up	Nucleus	transcription regulator
PABPC1	poly(A) binding protein cytoplasmic 1	4		Cytoplasm	translation regulator
PDPK1	3-phosphoinositide dependent protein kinase 1	3	Up	Cytoplasm	kinase
PIK3R1	phosphoinositide-3-kinase regulatory subunit 1	3	Up	Cytoplasm	kinase
PIK3R2	phosphoinositide-3-kinase regulatory subunit 2	6	Up	Cytoplasm	kinase
PPP1CA	protein phosphatase 1 catalytic subunit alpha	8	Up	Cytoplasm	phosphatase
PPP1CB	protein phosphatase 1 catalytic subunit beta	4	Up	Cytoplasm	phosphatase
PPP1CC	protein phosphatase 1 catalytic subunit gamma	6	Up	Nucleus	phosphatase
PTBP1	polypyrimidine tract binding protein 1	6		Nucleus	enzyme
PTPN11	protein tyrosine phosphatase, non-receptor type 11	3	Up	Cytoplasm	phosphatase
RAF1	Raf-1 proto-oncogene, serine/threonine kinase	8	Up	Cytoplasm	kinase

RPL4	ribosomal protein L4	5	Up	Cytoplasm	enzyme
RPL6	ribosomal protein L6	3	Up	Nucleus	other
RPL7	ribosomal protein L7	3	Up	Nucleus	transcription regulator
RPL9	ribosomal protein L9	3	Up	Nucleus	other
RPL10	ribosomal protein L10	5	Up	Cytoplasm	translation regulator
RPL11	ribosomal protein L11	6	Up	Cytoplasm	other
RPL12	ribosomal protein L12	5	Up	Nucleus	other
RPL13	ribosomal protein L13	5	Up	Nucleus	other
RPL21	ribosomal protein L21	3	Up	Cytoplasm	other
RPL22	ribosomal protein L22	3	Up	Nucleus	other
RPL23	ribosomal protein L23	5	Up	Cytoplasm	other
RPL26	ribosomal protein L26	3	Up	Cytoplasm	other
RPL23A	ribosomal protein L23a	3	Up	Cytoplasm	other
RPLP0	ribosomal protein lateral stalk subunit P0	5	Up	Cytoplasm	other
RPLP1	ribosomal protein lateral stalk subunit P1	3	Up	Cytoplasm	other
RPS2	ribosomal protein S2	3		Cytoplasm	other
RPS3	ribosomal protein S3	3		Cytoplasm	enzyme
RPS5	ribosomal protein S5	4		Cytoplasm	other
RPS6	ribosomal protein S6	4	Up	Cytoplasm	other
RPS8	ribosomal protein S8	3		Cytoplasm	other
RPS9	ribosomal protein S9	3		Cytoplasm	translation regulator
RPS14	ribosomal protein S14	3		Cytoplasm	translation regulator
RPS25	ribosomal protein S25	3		Cytoplasm	other
RPS27A	ribosomal protein S27a	3		Cytoplasm	other
RPS3A	ribosomal protein S3A	4		Nucleus	other
RPS4X	ribosomal protein S4, X-linked	3		Cytoplasm	other
SHC1	SHC adaptor protein 1	5	Up	Cytoplasm	other
TRIB3	tribbles pseudokinase 3	3	Down	Nucleus	kinase
VEGFA	vascular endothelial growth factor A	5		Extracellular Space	growth factor
XIAP	X-linked inhibitor of apoptosis	4	Up	Cytoplasm	enzyme

SUMOYLATION PATHWAY

Symbol	Entrez Gene Name	Expr Other	Expected	Location	Type(s)
AR	androgen receptor	7	Down	Nucleus	lig-dependent nuc. receptor
CDH1	cadherin 1	4	Up	Plasma Membrane	other
CEBPA	CCAAT/enhancer binding protein alpha	7	Down	Nucleus	transcription regulator
CREBBP	CREB binding protein	8	Down	Nucleus	transcription regulator
EP300	E1A binding protein p300	12	Down	Nucleus	transcription regulator
ETS1	ETS proto-oncogene 1, transcription factor	3		Nucleus	transcription regulator
FAS	Fas cell surface death receptor	3	Up	Plasma Membrane	transmembrane receptor

FOS	Fos proto-oncogene, AP-1 transcription factor subunit	7	Up	Nucleus	transcription regulator
HDAC1	histone deacetylase 1	14	Down	Nucleus	transcription regulator
HDAC2	histone deacetylase 2	6		Nucleus	transcription regulator
JUN	Jun proto-oncogene, AP-1 transcription factor subunit	12	Up	Nucleus	transcription regulator
MAP3K5	mitogen-activated protein kinase kinase kinase 5	4	Up	Cytoplasm	kinase
MAPK8	mitogen-activated protein kinase 8	5	Up	Cytoplasm	kinase
MDM2	MDM2 proto-oncogene	7	Down	Nucleus	transcription regulator
NFKB1	nuclear factor kappa B subunit 1	6	Down	Nucleus	transcription regulator
NFKB2	nuclear factor kappa B subunit 2	4	Down	Nucleus	transcription regulator
NFKBIA	NFKB inhibitor alpha	14		Cytoplasm	transcription regulator
NR3C1	nuclear receptor subfamily 3 group C member 1	5	Down	Nucleus	lig-dependent nuc. receptor
PCNA	proliferating cell nuclear antigen	4	Down	Nucleus	enzyme
PIAS1	protein inhibitor of activated STAT 1	4	Down	Nucleus	transcription regulator
PIAS4	protein inhibitor of activated STAT 4	5	Down	Nucleus	transcription regulator
PML	promyelocytic leukemia	7	Up	Nucleus	transcription regulator
RAN	RAN, member RAS oncogene family	3	Up	Nucleus	enzyme
RNF4	ring finger protein 4	3	Up	Nucleus	transcription regulator
RPA1	replication protein A1	9	Down	Nucleus	other
SERBP1	SERPINE1 mRNA binding protein 1	3	Down	Cytoplasm	other
SIRT1	sirtuin 1	11	Down	Nucleus	transcription regulator
SMAD4	SMAD family member 4	4	Up	Nucleus	transcription regulator
SNCA	synuclein alpha	9	Down	Cytoplasm	enzyme
SP1	Sp1 transcription factor	8	Up	Nucleus	transcription regulator
SP3	Sp3 transcription factor	3	Up	Nucleus	transcription regulator
STUB1	STIP1 homology and U-box containing protein 1	7	Down	Cytoplasm	enzyme
SUMO1	small ubiquitin-like modifier 1	7	Up	Nucleus	enzyme
SUMO2	small ubiquitin-like modifier 2	3	Up	Nucleus	enzyme
SUMO3	small ubiquitin-like modifier 3	9	Up	Nucleus	other
TDG	thymine DNA glycosylase	4	Down	Nucleus	enzyme
TP53	tumor protein p53	26	Up	Nucleus	transcription regulator
UBE2I	ubiquitin conjugating enzyme E2 I	8	Up	Nucleus	enzyme
XIAP	X-linked inhibitor of apoptosis	4		Cytoplasm	enzyme

P70S6K SIGNALING

Symbol	Entrez Gene Name	Expr Other	Expected	Location	Type(s)
AGT	angiotensinogen	3	Up	Extracellular Space	growth factor
AKT1	AKT serine/threonine kinase 1	7	Up	Cytoplasm	kinase
ATM	ATM serine/threonine kinase	5	Up	Nucleus	kinase
BTK	Bruton tyrosine kinase	3	Up	Cytoplasm	kinase
EEF2	eukaryotic translation elongation factor 2	6	Up	Cytoplasm	translation regulator

EGFR	epidermal growth factor receptor	18	Up	Plasma Membrane	kinase
F2	coagulation factor II, thrombin	3	Up	Extracellular Space	peptidase
FGFR1	fibroblast growth factor receptor 1	4	Up	Plasma Membrane	kinase
GRB2	growth factor receptor bound protein 2	19	Up	Cytoplasm	kinase
IL4	interleukin 4	3	Up	Extracellular Space	cytokine
MAP2K1	mitogen-activated protein kinase kinase 1	3	Up	Cytoplasm	kinase
MAPK1	mitogen-activated protein kinase 1	9	Up	Cytoplasm	kinase
MAPK3	mitogen-activated protein kinase 3	16	Up	Cytoplasm	kinase
MAPT	microtubule associated protein tau	9	Up	Plasma Membrane	other
MTOR	mechanistic target of rapamycin kinase	3	Up	Nucleus	kinase
PDIA3	protein disulfide isomerase family A member 3	4	Up	Cytoplasm	peptidase
PDPK1	3-phosphoinositide dependent protein kinase 1	3	Up	Cytoplasm	kinase
PIK3R1	phosphoinositide-3-kinase regulatory subunit 1	3	Up	Cytoplasm	kinase
PIK3R2	phosphoinositide-3-kinase regulatory subunit 2	6	Up	Cytoplasm	kinase
PLCB1	phospholipase C beta 1	4	Up	Cytoplasm	enzyme
PLCG1	phospholipase C gamma 1	4	Up	Cytoplasm	enzyme
PLCG2	phospholipase C gamma 2	3	Up	Cytoplasm	enzyme
PPP2CA	protein phosphatase 2 catalytic subunit alpha	3	Down	Cytoplasm	phosphatase
PPP2R1A	protein phosphatase 2 scaffold subunit Aalpha	8	Down	Cytoplasm	phosphatase
PPP2R1B	protein phosphatase 2 scaffold subunit Abeta	3	Down	Plasma Membrane	phosphatase
PPP2R2A	protein phosphatase 2 regulatory subunit Balpha	3	Down	Cytoplasm	phosphatase
PPP2R5D	protein phosphatase 2 regulatory subunit B'delta	3	Down	Nucleus	phosphatase
PRKCA	protein kinase C alpha	3	Up	Cytoplasm	kinase
PRKCB	protein kinase C beta	5	Up	Cytoplasm	kinase
PRKCD	protein kinase C delta	3	Up	Cytoplasm	kinase
PRKCG	protein kinase C gamma	5	Up	Cytoplasm	kinase
PRKCZ	protein kinase C zeta	3	Up	Cytoplasm	kinase
PTPN11	protein tyrosine phosphatase, non-receptor type 11	3	Up	Cytoplasm	phosphatase
RAF1	Raf-1 proto-oncogene, serine/threonine kinase	8	Up	Cytoplasm	kinase
RPS6	ribosomal protein S6	4	Up	Cytoplasm	other
SFN	stratifin	3		Cytoplasm	other
SHC1	SHC adaptor protein 1	5	Up	Cytoplasm	other
SRC	SRC proto-oncogene, non-receptor tyrosine kinase	10	Up	Cytoplasm	kinase
SYK	spleen associated tyrosine kinase	5	Up	Cytoplasm	kinase
YWHAB	tyrosine 3-monooxygenase/tryptophan 5-monooxygenase activatio	6		Cytoplasm	transcription regulator
YWHAE	tyrosine 3-monooxygenase/tryptophan 5-monooxygenase activatio	11		Cytoplasm	other
YWHAG	tyrosine 3-monooxygenase/tryptophan 5-monooxygenase activatio	9		Cytoplasm	other
YWHAH	tyrosine 3-monooxygenase/tryptophan 5-monooxygenase activatio	7		Cytoplasm	transcription regulator
YWHAQ	tyrosine 3-monooxygenase/tryptophan 5-monooxygenase activatio	14		Cytoplasm	other
YWHAZ	tyrosine 3-monooxygenase/tryptophan 5-monooxygenase activatio	16		Cytoplasm	enzyme

UNFOLDED PROTEIN RESPONSE

Symbol	Entrez Gene Name	Expr Other	Expected	Location	Type(s)
AMFR	autocrine motility factor receptor	3		Plasma Membrane	transmembrane receptor
ATF4	activating transcription factor 4	6		Nucleus	transcription regulator
BCL2	BCL2, apoptosis regulator	6		Cytoplasm	transporter
CALR	calreticulin	8		Cytoplasm	transcription regulator
CANX	calnexin	5		Cytoplasm	other
CEBPA	CCAAT/enhancer binding protein alpha	7		Nucleus	transcription regulator
HSP90B1	heat shock protein 90 beta family member 1	5		Cytoplasm	other
HSPA2	heat shock protein family A (Hsp70) member 2	4		Cytoplasm	other
HSPA4	heat shock protein family A (Hsp70) member 4	8		Cytoplasm	other
HSPA5	heat shock protein family A (Hsp70) member 5	14		Cytoplasm	enzyme
HSPA8	heat shock protein family A (Hsp70) member 8	12		Cytoplasm	enzyme
HSPA9	heat shock protein family A (Hsp70) member 9	7		Cytoplasm	other
HSPA1A/HSPA1B	heat shock protein family A (Hsp70) member 1A	10		Cytoplasm	enzyme
HSPH1	heat shock protein family H (Hsp110) member 1	3		Cytoplasm	other
INSIG1	insulin induced gene 1	3		Cytoplasm	other
MAP3K5	mitogen-activated protein kinase kinase kinase 5	4		Cytoplasm	kinase
MAPK8	mitogen-activated protein kinase 8	5		Cytoplasm	kinase
NFE2L2	nuclear factor, erythroid 2 like 2	8		Nucleus	transcription regulator
P4HB	prolyl 4-hydroxylase subunit beta	4		Cytoplasm	enzyme
PPARG	peroxisome proliferator activated receptor gamma	6		Nucleus	lig-dependent nuc. receptor
SYVN1	synoviolin 1	5		Cytoplasm	transporter
TRAF2	TNF receptor associated factor 2	6		Cytoplasm	enzyme
VCP	valosin containing protein	16		Cytoplasm	enzyme
VCP	valosin containing protein	16		Cytoplasm	enzyme

PROTEIN UBIQUITINATION PATHWAY

Symbol	Entrez Gene Name	Expr Other	Expected	Location	Type(s)
AMFR	autocrine motility factor receptor	3		Plasma Membrane	transmembrane receptor
BAG1	BCL2 associated athanogene 1	5		Cytoplasm	other
BRCA1	BRCA1, DNA repair associated	9		Nucleus	transcription regulator
BTRC	beta-transducin repeat containing E3 ubiquitin protein ligase	4		Cytoplasm	enzyme
CBL	Cbl proto-oncogene	4		Nucleus	transcription regulator
CRYAB	crystallin alpha B	4		Nucleus	other
CUL1	cullin 1	12		Nucleus	enzyme
CUL2	cullin 2	9		Nucleus	enzyme
DNAJC10	DnaJ heat shock protein family (Hsp40) member C10	3		Cytoplasm	enzyme
FBXW7	F-box and WD repeat domain containing 7	3		Nucleus	transcription regulator
HLA-B	major histocompatibility complex, class I, B	3		Plasma Membrane	transmembrane receptor
HSP90AA1	heat shock protein 90 alpha family class A member 1	11		Cytoplasm	enzyme

HSP90AB1	heat shock protein 90 alpha family class B member 1	9	Cytoplasm	enzyme
HSP90B1	heat shock protein 90 beta family member 1	5	Cytoplasm	other
HSPA2	heat shock protein family A (Hsp70) member 2	4	Cytoplasm	other
HSPA4	heat shock protein family A (Hsp70) member 4	8	Cytoplasm	other
HSPA5	heat shock protein family A (Hsp70) member 5	14	Cytoplasm	enzyme
HSPA8	heat shock protein family A (Hsp70) member 8	12	Cytoplasm	enzyme
HSPA9	heat shock protein family A (Hsp70) member 9	7	Cytoplasm	other
HSPA1A/HSPA1B	heat shock protein family A (Hsp70) member 1A	10	Cytoplasm	enzyme
HSPB1	heat shock protein family B (small) member 1	11	Cytoplasm	other
HSPB2	heat shock protein family B (small) member 2	4	Cytoplasm	other
HSPD1	heat shock protein family D (Hsp60) member 1	4	Cytoplasm	enzyme
HSPH1	heat shock protein family H (Hsp110) member 1	3	Cytoplasm	other
IFNG	interferon gamma	3	Extracellular Space	cytokine
MDM2	MDM2 proto-oncogene	7	Nucleus	transcription regulator
NEDD4	neural precursor cell expressed, developmentally down-regulated [illegible]	5	Cytoplasm	enzyme
PRKN	parkin RBR E3 ubiquitin protein ligase	14	Cytoplasm	enzyme
PSMA3	proteasome subunit alpha 3	4	Cytoplasm	peptidase
PSMA5	proteasome subunit alpha 5	4	Cytoplasm	peptidase
PSMA6	proteasome subunit alpha 6	3	Cytoplasm	peptidase
PSMC2	proteasome 26S subunit, ATPase 2	3	Nucleus	peptidase
PSMC3	proteasome 26S subunit, ATPase 3	5	Nucleus	enzyme
PSMC5	proteasome 26S subunit, ATPase 5	3	Nucleus	transcription regulator
PSMD4	proteasome 26S subunit, non-ATPase 4	3	Cytoplasm	other
RPS27A	ribosomal protein S27a	3	Cytoplasm	other
SKP1	S-phase kinase associated protein 1	3	Nucleus	transcription regulator
SMURF1	SMAD specific E3 ubiquitin protein ligase 1	6	Cytoplasm	enzyme
STUB1	STIP1 homology and U-box containing protein 1	7	Cytoplasm	enzyme
TRAF6	TNF receptor associated factor 6	11	Cytoplasm	enzyme
UBB	ubiquitin B	4	Cytoplasm	enzyme
UBE2I	ubiquitin conjugating enzyme E2 I	8	Nucleus	enzyme
UBE3A	ubiquitin protein ligase E3A	3	Nucleus	enzyme
UBR2	ubiquitin protein ligase E3 component n-recognin 2	3	Nucleus	enzyme
USP7	ubiquitin specific peptidase 7	5	Nucleus	peptidase
USP8	ubiquitin specific peptidase 8	4	Cytoplasm	peptidase
USP10	ubiquitin specific peptidase 10	3	Cytoplasm	peptidase
USP12	ubiquitin specific peptidase 12	3	Cytoplasm	peptidase
USP9X	ubiquitin specific peptidase 9, X-linked	3	Plasma Membrane	peptidase
VHL	von Hippel-Lindau tumor suppressor	20	Nucleus	transcription regulator
XIAP	X-linked inhibitor of apoptosis	4	Cytoplasm	enzyme

ER STRESS PATHWAY					
**Symbol**	**Entrez Gene Name**	**Expr Other**	**Expected**	**Location**	**Type(s)**
ATF4	activating transcription factor 4	6		Nucleus	transcription regulator
CALR	calreticulin	8		Cytoplasm	transcription regulator
CASP3	caspase 3	10		Cytoplasm	peptidase
CASP7	caspase 7	7		Cytoplasm	peptidase
CASP9	caspase 9	5		Cytoplasm	peptidase
HSP90B1	heat shock protein 90 beta family member 1	5		Cytoplasm	other
HSPA5	heat shock protein family A (Hsp70) member 5	14		Cytoplasm	enzyme
MAP3K5	mitogen-activated protein kinase kinase kinase 5	4		Cytoplasm	kinase
MAPK8	mitogen-activated protein kinase 8	5		Cytoplasm	kinase
TRAF2	TNF receptor associated factor 2	6		Cytoplasm	enzyme

Supplementary Table 7

Canonical pathways and proteins involved in hypoxic response. The table includes, gene, ID, Entrez Gene name, their total number of protein interactions, location of the protein and the protein type.

CANONICAL PATHWAYS RELATED TO HYPOXIA					
**PRODUCTION OF NITRIC OXIDE AND REACTIVE OXYGEN SPECIES**					
**Symbol**	**Entrez Gene Name**	**Expr Other**	**Expected**	**Location**	**Type(s)**
AKT1	AKT serine/threonine kinase 1	7	Up	Cytoplasm	kinase
APOA1	apolipoprotein A1	7	Up	Extracellular Space	transporter
APOE	apolipoprotein E	5	Up	Extracellular Space	transporter
ATM	ATM serine/threonine kinase	5	Up	Nucleus	kinase
CLU	clusterin	3	Up	Cytoplasm	other
CREBBP	CREB binding protein	8	Up	Nucleus	transcription regulator
FGFR1	fibroblast growth factor receptor 1	4	Up	Plasma Membrane	kinase
FOS	Fos proto-oncogene, AP-1 transcription factor subunit	7	Up	Nucleus	transcription regulator
GRB2	growth factor receptor bound protein 2	19	Up	Cytoplasm	kinase
HOXA10	homeobox A10	3	Down	Nucleus	transcription regulator
IFNG	interferon gamma	3	Up	Extracellular Space	cytokine
IKBKE	inhibitor of nuclear factor kappa B kinase subunit epsilon	4	Up	Cytoplasm	kinase
IKBKG	inhibitor of nuclear factor kappa B kinase subunit gamma	4	Up	Nucleus	kinase
IL4	interleukin 4	3		Extracellular Space	cytokine
IRF8	interferon regulatory factor 8	3	Up	Nucleus	transcription regulator
JUN	Jun proto-oncogene, AP-1 transcription factor subunit	12	Up	Nucleus	transcription regulator
MAP2K1	mitogen-activated protein kinase kinase 1	3	Up	Cytoplasm	kinase
MAP3K3	mitogen-activated protein kinase kinase kinase 3	5	Up	Cytoplasm	kinase
MAP3K5	mitogen-activated protein kinase kinase kinase 5	4	Up	Cytoplasm	kinase
MAP3K7	mitogen-activated protein kinase kinase kinase 7	3	Up	Cytoplasm	kinase
MAPK1	mitogen-activated protein kinase 1	9	Up	Cytoplasm	kinase
MAPK3	mitogen-activated protein kinase 3	16	Up	Cytoplasm	kinase
MAPK8	mitogen-activated protein kinase 8	5	Up	Cytoplasm	kinase
MAPK13	mitogen-activated protein kinase 13	7	Up	Cytoplasm	kinase
NFKB1	nuclear factor kappa B subunit 1	6	Up	Nucleus	transcription regulator
NFKB2	nuclear factor kappa B subunit 2	4	Up	Nucleus	transcription regulator
NFKBIA	NFKB inhibitor alpha	14	Up	Cytoplasm	transcription regulator
NGFR	nerve growth factor receptor	5	Up	Plasma Membrane	transmembrane receptor
NOS2	nitric oxide synthase 2	5	Up	Cytoplasm	enzyme
PIK3R1	phosphoinositide-3-kinase regulatory subunit 1	3	Up	Cytoplasm	kinase
PIK3R2	phosphoinositide-3-kinase regulatory subunit 2	6	Up	Cytoplasm	kinase
PLCG1	phospholipase C gamma 1	4	Up	Cytoplasm	enzyme
PLCG2	phospholipase C gamma 2	3	Up	Cytoplasm	enzyme
PPARA	peroxisome proliferator activated receptor alpha	3	Up	Nucleus	lig-dependent nuc. receptor
PPP1CA	protein phosphatase 1 catalytic subunit alpha	8	Down	Cytoplasm	phosphatase
PPP1CB	protein phosphatase 1 catalytic subunit beta	4	Down	Cytoplasm	phosphatase
PPP1CC	protein phosphatase 1 catalytic subunit gamma	6	Down	Nucleus	phosphatase
PPP1R12A	protein phosphatase 1 regulatory subunit 12A	3	Down	Cytoplasm	phosphatase

PPP2CA	protein phosphatase 2 catalytic subunit alpha	3	Down	Cytoplasm	phosphatase
PPP2R1A	protein phosphatase 2 scaffold subunit Aalpha	8	Down	Cytoplasm	phosphatase
PPP2R1B	protein phosphatase 2 scaffold subunit Abeta	3	Down	Plasma Membrane	phosphatase
PPP2R2A	protein phosphatase 2 regulatory subunit Balpha	3	Down	Cytoplasm	phosphatase
PPP2R5D	protein phosphatase 2 regulatory subunit B'delta	3	Down	Nucleus	phosphatase
PRKCA	protein kinase C alpha	3	Up	Cytoplasm	kinase
PRKCB	protein kinase C beta	5	Up	Cytoplasm	kinase
PRKCD	protein kinase C delta	3	Up	Cytoplasm	kinase
PRKCG	protein kinase C gamma	5	Up	Cytoplasm	kinase
PRKCZ	protein kinase C zeta	3	Up	Cytoplasm	kinase
PTPN11	protein tyrosine phosphatase, non-receptor type 11	3	Up	Cytoplasm	phosphatase
RAC1	Rac family small GTPase 1	6	Up	Plasma Membrane	enzyme
RELA	RELA proto-oncogene, NF-kB subunit	10	Up	Nucleus	transcription regulator
S100A8	S100 calcium binding protein A8	3	Up	Cytoplasm	other
STAT1	signal transducer and activator of transcription 1	9	Up	Nucleus	transcription regulator
TNF	tumor necrosis factor	7	Up	Extracellular Space	cytokine
TNFRSF1A	TNF receptor superfamily member 1A	3	Up	Plasma Membrane	transmembrane receptor

NRF2-MEDIATED OXIDATIVE STRESS

Symbol	Entrez Gene Name	Expr Other	Expected	Location	Type(s)
ACTA1	actin, alpha 1, skeletal muscle	4		Cytoplasm	other
ACTB	actin beta	13		Cytoplasm	other
ACTG1	actin gamma 1	4		Cytoplasm	other
AKT1	AKT serine/threonine kinase 1	7		Cytoplasm	kinase
ATF4	activating transcription factor 4	6	Up	Nucleus	transcription regulator
ATM	ATM serine/threonine kinase	5		Nucleus	kinase
CCT7	chaperonin containing TCP1 subunit 7	4	Up	Cytoplasm	other
CREBBP	CREB binding protein	8	Up	Nucleus	transcription regulator
CUL3	cullin 3	21		Nucleus	enzyme
DNAJC10	DnaJ heat shock protein family (Hsp40) member C10	3		Cytoplasm	enzyme
EP300	E1A binding protein p300	12	Up	Nucleus	transcription regulator
FGFR1	fibroblast growth factor receptor 1	4		Plasma Membrane	kinase
FOS	Fos proto-oncogene, AP-1 transcription factor subunit	7	Down	Nucleus	transcription regulator
GRB2	growth factor receptor bound protein 2	19		Cytoplasm	kinase
GSK3B	glycogen synthase kinase 3 beta	11		Nucleus	kinase
GSTP1	glutathione S-transferase pi 1	3		Cytoplasm	enzyme
HACD3	3-hydroxyacyl-CoA dehydratase 3	3	Up	Cytoplasm	enzyme
HERPUD1	homocysteine inducible ER protein with ubiquitin like domain 1	3	Up	Cytoplasm	other
JUN	Jun proto-oncogene, AP-1 transcription factor subunit	12	Up	Nucleus	transcription regulator
JUNB	JunB proto-oncogene, AP-1 transcription factor subunit	4	Up	Nucleus	transcription regulator

JUND	JunD proto-oncogene, AP-1 transcription factor subunit	4	Up	Nucleus	transcription regulator
MAP2K1	mitogen-activated protein kinase kinase 1	3	Up	Cytoplasm	kinase
MAP3K5	mitogen-activated protein kinase kinase kinase 5	4	Up	Cytoplasm	kinase
MAP3K7	mitogen-activated protein kinase kinase kinase 7	3	Up	Cytoplasm	kinase
MAPK1	mitogen-activated protein kinase 1	9	Up	Cytoplasm	kinase
MAPK3	mitogen-activated protein kinase 3	16	Up	Cytoplasm	kinase
MAPK8	mitogen-activated protein kinase 8	5	Up	Cytoplasm	kinase
NFE2L2	nuclear factor, erythroid 2 like 2	8	Up	Nucleus	transcription regulator
PIK3R1	phosphoinositide-3-kinase regulatory subunit 1	3		Cytoplasm	kinase
PIK3R2	phosphoinositide-3-kinase regulatory subunit 2	6		Cytoplasm	kinase
PRDX1	peroxiredoxin 1	4	Up	Cytoplasm	enzyme
PRKCA	protein kinase C alpha	3	Up	Cytoplasm	kinase
PRKCB	protein kinase C beta	5	Up	Cytoplasm	kinase
PRKCD	protein kinase C delta	3	Up	Cytoplasm	kinase
PRKCG	protein kinase C gamma	5	Up	Cytoplasm	kinase
PRKCZ	protein kinase C zeta	3	Up	Cytoplasm	kinase
PTPN11	protein tyrosine phosphatase, non-receptor type 11	3		Cytoplasm	phosphatase
RAF1	Raf-1 proto-oncogene, serine/threonine kinase	8	Up	Cytoplasm	kinase
SCARB1	scavenger receptor class B member 1	3	Up	Plasma Membrane	transporter
SOD1	superoxide dismutase 1	10		Cytoplasm	enzyme
SOD2	superoxide dismutase 2	3		Cytoplasm	enzyme
SQSTM1	sequestosome 1	13	Up	Cytoplasm	transcription regulator
TXN	thioredoxin	3	Up	Cytoplasm	enzyme
UBB	ubiquitin B	4	Up	Cytoplasm	enzyme
VCP	valosin containing protein	16	Up	Cytoplasm	enzyme

iNOS SIGNALING

Symbol	Entrez Gene Name	Expr Other	Expected	Location	Type(s)
CALM1 (includes othe	calmodulin 1	9		Cytoplasm	other
CREBBP	CREB binding protein	8	Up	Nucleus	transcription regulator
FOS	Fos proto-oncogene, AP-1 transcription factor subunit	7	Up	Nucleus	transcription regulator
HMGA1	high mobility group AT-hook 1	3		Nucleus	transcription regulator
IFNG	interferon gamma	3	Up	Extracellular Space	cytokine
IKBKE	inhibitor of nuclear factor kappa B kinase subunit epsilon	4	Up	Cytoplasm	kinase
IKBKG	inhibitor of nuclear factor kappa B kinase subunit gamma	4	Up	Nucleus	kinase
IRAK1	interleukin 1 receptor associated kinase 1	3	Up	Plasma Membrane	kinase
IRAK2	interleukin 1 receptor associated kinase 2	3	Up	Plasma Membrane	kinase
JUN	Jun proto-oncogene, AP-1 transcription factor subunit	12	Up	Nucleus	transcription regulator
MAPK1	mitogen-activated protein kinase 1	9	Up	Cytoplasm	kinase
MAPK13	mitogen-activated protein kinase 13	7	Up	Cytoplasm	kinase
MYD88	myeloid differentiation primary response 88	3	Up	Plasma Membrane	other

NFKB1	nuclear factor kappa B subunit 1	6	Up	Nucleus	transcription regulator
NFKB2	nuclear factor kappa B subunit 2	4	Up	Nucleus	transcription regulator
NFKBIA	NFKB inhibitor alpha	14	Up	Cytoplasm	transcription regulator
NOS2	nitric oxide synthase 2	5	Up	Cytoplasm	enzyme
RELA	RELA proto-oncogene, NF-kB subunit	10	Up	Nucleus	transcription regulator
STAT1	signal transducer and activator of transcription 1	9	Up	Nucleus	transcription regulator
TRAF6	TNF receptor associated factor 6	11	Up	Cytoplasm	enzyme

HYPOXIA SIGNALING

Symbol	Entrez Gene Name	Expr Other	Expected	Location	Type(s)
AKT1	AKT serine/threonine kinase 1	7	Up	Cytoplasm	kinase
ATF2	activating transcription factor 2	3	Up	Nucleus	transcription regulator
ATF4	activating transcription factor 4	6	Up	Nucleus	transcription regulator
ATM	ATM serine/threonine kinase	5	Down	Nucleus	kinase
COPS5	COP9 signalosome subunit 5	11	Up	Nucleus	transcription regulator
CREB1	cAMP responsive element binding protein 1	11	Up	Nucleus	transcription regulator
CREBBP	CREB binding protein	8	Up	Nucleus	transcription regulator
EP300	E1A binding protein p300	12	Up	Nucleus	transcription regulator
HIF1A	hypoxia inducible factor 1 alpha subunit	9	Up	Nucleus	transcription regulator
HSP90AA1	heat shock protein 90 alpha family class A member 1	11		Cytoplasm	enzyme
HSP90AB1	heat shock protein 90 alpha family class B member 1	9		Cytoplasm	enzyme
HSP90B1	heat shock protein 90 beta family member 1	5		Cytoplasm	other
IKBKG	inhibitor of nuclear factor kappa B kinase subunit gamma	4	Up	Nucleus	kinase
JUN	Jun proto-oncogene, AP-1 transcription factor subunit	12	Up	Nucleus	transcription regulator
LDHA	lactate dehydrogenase A	3	Up	Cytoplasm	enzyme
MDM2	MDM2 proto-oncogene	7	Up	Nucleus	transcription regulator
NFKBIA	NFKB inhibitor alpha	14	Up	Cytoplasm	transcription regulator
NOS3	nitric oxide synthase 3	5	Up	Cytoplasm	enzyme
P4HB	prolyl 4-hydroxylase subunit beta	4		Cytoplasm	enzyme
PTEN	phosphatase and tensin homolog	6	Down	Cytoplasm	phosphatase
SUMO1	small ubiquitin-like modifier 1	7		Nucleus	enzyme
TP53	tumor protein p53	26	Down	Nucleus	transcription regulator
UBE2I	ubiquitin conjugating enzyme E2 I	8		Nucleus	enzyme
VEGFA	vascular endothelial growth factor A	5	Up	Extracellular Space	growth factor
VHL	von Hippel-Lindau tumor suppressor	20	Down	Nucleus	transcription regulator

HIF1a SIGNALING

Symbol	Entrez Gene Name	Expr Other	Expected	Location	Type(s)
AKT1	AKT serine/threonine kinase 1	7		Cytoplasm	kinase
APEX1	apurinic/apyrimidinic endodeoxyribonuclease 1	4		Nucleus	enzyme
ATM	ATM serine/threonine kinase	5		Nucleus	kinase

COPS5	COP9 signalosome subunit 5	11	Nucleus	transcription regulator
CREBBP	CREB binding protein	8	Nucleus	transcription regulator
CUL2	cullin 2	9	Nucleus	enzyme
EP300	E1A binding protein p300	12	Nucleus	transcription regulator
FGFR1	fibroblast growth factor receptor 1	4	Plasma Membrane	kinase
GRB2	growth factor receptor bound protein 2	19	Cytoplasm	kinase
HIF1A	hypoxia inducible factor 1 alpha subunit	9	Nucleus	transcription regulator
HSP90AA1	heat shock protein 90 alpha family class A member 1	11	Cytoplasm	enzyme
JUN	Jun proto-oncogene, AP-1 transcription factor subunit	12	Nucleus	transcription regulator
LDHA	lactate dehydrogenase A	3	Cytoplasm	enzyme
MAPK1	mitogen-activated protein kinase 1	9	Cytoplasm	kinase
MAPK3	mitogen-activated protein kinase 3	16	Cytoplasm	kinase
MAPK8	mitogen-activated protein kinase 8	5	Cytoplasm	kinase
MAPK13	mitogen-activated protein kinase 13	7	Cytoplasm	kinase
MDM2	MDM2 proto-oncogene	7	Nucleus	transcription regulator
NCOA1	nuclear receptor coactivator 1	3	Nucleus	transcription regulator
NOS2	nitric oxide synthase 2	5	Cytoplasm	enzyme
NOS3	nitric oxide synthase 3	5	Cytoplasm	enzyme
PIK3R1	phosphoinositide-3-kinase regulatory subunit 1	3	Cytoplasm	kinase
PIK3R2	phosphoinositide-3-kinase regulatory subunit 2	6	Cytoplasm	kinase
PTPN11	protein tyrosine phosphatase, non-receptor type 11	3	Cytoplasm	phosphatase
TP53	tumor protein p53	26	Nucleus	transcription regulator
VEGFA	vascular endothelial growth factor A	5	Extracellular Space	growth factor
VHL	von Hippel-Lindau tumor suppressor	20	Nucleus	transcription regulator

Supplementary Table 8

Canonical pathways and proteins involved in DNA damage and repair. The table includes, gene ID, Entrez Gene name, their total number of protein interactions, location of the protein and the protein type.

CANONICAL PATHWAYS RELATED TO DNA DAMAGE AND REPAIR					
**TELOMERASE SIGNALING**					
**Symbol**	**Entrez Gene Name**	**Expr Other**	**Expected**	**Location**	**Type(s)**
ABL1	ABL proto-oncogene 1, non-receptor tyrosine kinase	6	Down	Nucleus	kinase
AKT1	AKT serine/threonine kinase 1	7	Up	Cytoplasm	kinase
ATM	ATM serine/threonine kinase	5	Up	Nucleus	kinase
CDKN1A	cyclin dependent kinase inhibitor 1A	6		Nucleus	kinase
E2F1	E2F transcription factor 1	4		Nucleus	transcription regulator
EGFR	epidermal growth factor receptor	18	Up	Plasma Membrane	kinase
ELF3	E74 like ETS transcription factor 3	3	Up	Nucleus	transcription regulator
ETS1	ETS proto-oncogene 1, transcription factor	3	Up	Nucleus	transcription regulator
FGFR1	fibroblast growth factor receptor 1	4	Up	Plasma Membrane	kinase
GRB2	growth factor receptor bound protein 2	19	Up	Cytoplasm	kinase
HDAC1	histone deacetylase 1	14		Nucleus	transcription regulator
HDAC2	histone deacetylase 2	6		Nucleus	transcription regulator
HDAC3	histone deacetylase 3	4		Nucleus	transcription regulator
HDAC4	histone deacetylase 4	6		Nucleus	transcription regulator
HDAC5	histone deacetylase 5	5		Nucleus	transcription regulator
HDAC6	histone deacetylase 6	13		Nucleus	transcription regulator
HDAC7	histone deacetylase 7	3		Nucleus	transcription regulator
HSP90AA1	heat shock protein 90 alpha family class A member 1	11	Up	Cytoplasm	enzyme
HSP90AB1	heat shock protein 90 alpha family class B member 1	9	Up	Cytoplasm	enzyme
HSP90B1	heat shock protein 90 beta family member 1	5	Up	Cytoplasm	other
MAP2K1	mitogen-activated protein kinase kinase 1	3	Up	Cytoplasm	kinase
MAPK1	mitogen-activated protein kinase 1	9	Up	Cytoplasm	kinase
MAPK3	mitogen-activated protein kinase 3	16	Up	Cytoplasm	kinase
MYC	MYC proto-oncogene, bHLH transcription factor	18	Up	Nucleus	transcription regulator
PDPK1	3-phosphoinositide dependent protein kinase 1	3	Up	Cytoplasm	kinase
PIK3R1	phosphoinositide-3-kinase regulatory subunit 1	3	Up	Cytoplasm	kinase
PIK3R2	phosphoinositide-3-kinase regulatory subunit 2	6	Up	Cytoplasm	kinase
PPP2CA	protein phosphatase 2 catalytic subunit alpha	3	Down	Cytoplasm	phosphatase
PPP2R1A	protein phosphatase 2 scaffold subunit Aalpha	8	Down	Cytoplasm	phosphatase
PPP2R1B	protein phosphatase 2 scaffold subunit Abeta	3	Down	Plasma Membrane	phosphatase
PPP2R2A	protein phosphatase 2 regulatory subunit Balpha	3	Down	Cytoplasm	phosphatase
PPP2R5D	protein phosphatase 2 regulatory subunit B'delta	3	Down	Nucleus	phosphatase
PTPN11	protein tyrosine phosphatase, non-receptor type 11	3	Up	Cytoplasm	phosphatase
RAF1	Raf-1 proto-oncogene, serine/threonine kinase	8	Up	Cytoplasm	kinase
RB1	RB transcriptional corepressor 1	5		Nucleus	transcription regulator
SHC1	SHC adaptor protein 1	5	Up	Cytoplasm	other
SP1	Sp1 transcription factor	8		Nucleus	transcription regulator
TERF1	telomeric repeat binding factor 1	4		Nucleus	other

TERF2	telomeric repeat binding factor 2	3		Nucleus	other
TERF2IP	TERF2 interacting protein	4		Nucleus	other
TERT	telomerase reverse transcriptase	3	Up	Nucleus	enzyme
TP53	tumor protein p53	26	Down	Nucleus	transcription regulator

ATM SIGNALING

Symbol	Entrez Gene Name	Expr Other	Expected	Location	Type(s)
ABL1	ABL proto-oncogene 1, non-receptor tyrosine kinase	6	Up	Nucleus	kinase
ATF2	activating transcription factor 2	3	Up	Nucleus	transcription regulator
ATF4	activating transcription factor 4	6	Down	Nucleus	transcription regulator
ATM	ATM serine/threonine kinase	5	Up	Nucleus	kinase
BRCA1	BRCA1, DNA repair associated	9	Up	Nucleus	transcription regulator
CCNB1	cyclin B1	7	Down	Cytoplasm	kinase
CDK1	cyclin dependent kinase 1	10	Down	Nucleus	kinase
CDK2	cyclin dependent kinase 2	10	Down	Nucleus	kinase
CDKN1A	cyclin dependent kinase inhibitor 1A	6	Up	Nucleus	kinase
CHEK1	checkpoint kinase 1	3	Up	Nucleus	kinase
CHEK2	checkpoint kinase 2	3	Up	Nucleus	kinase
CREB1	cAMP responsive element binding protein 1	11	Down	Nucleus	transcription regulator
CREBBP	CREB binding protein	8	Down	Nucleus	transcription regulator
EP300	E1A binding protein p300	12	Down	Nucleus	transcription regulator
GADD45A	growth arrest and DNA damage inducible alpha	3		Nucleus	other
H2AFX	H2A histone family member X	5	Up	Nucleus	transcription regulator
HERC2	HECT and RLD domain containing E3 ubiquitin protein ligase 2	4	Up	Cytoplasm	enzyme
JUN	Jun proto-oncogene, AP-1 transcription factor subunit	12	Up	Nucleus	transcription regulator
MAPK8	mitogen-activated protein kinase 8	5	Up	Cytoplasm	kinase
MAPK13	mitogen-activated protein kinase 13	7	Up	Cytoplasm	kinase
MDC1	mediator of DNA damage checkpoint 1	3	Up	Nucleus	other
MDM2	MDM2 proto-oncogene	7	Down	Nucleus	transcription regulator
NFKBIA	NFKB inhibitor alpha	14		Cytoplasm	transcription regulator
PPP1CC	protein phosphatase 1 catalytic subunit gamma	6	Down	Nucleus	phosphatase
PPP2CA	protein phosphatase 2 catalytic subunit alpha	3	Down	Cytoplasm	phosphatase
PPP2R1A	protein phosphatase 2 scaffold subunit Aalpha	8	Down	Cytoplasm	phosphatase
PPP2R1B	protein phosphatase 2 scaffold subunit Abeta	3	Down	Plasma Membrane	phosphatase
PPP2R2A	protein phosphatase 2 regulatory subunit Balpha	3	Down	Cytoplasm	phosphatase
PPP2R5D	protein phosphatase 2 regulatory subunit B'delta	3	Down	Nucleus	phosphatase
RAD50	RAD50 double strand break repair protein	3		Nucleus	enzyme
RAD51	RAD51 recombinase	3	Up	Nucleus	enzyme
TP53	tumor protein p53	26	Up	Nucleus	transcription regulator
TP73	tumor protein p73	3	Up	Nucleus	transcription regulator
TP53BP1	tumor protein p53 binding protein 1	4	Up	Nucleus	transcription regulator

TRIM28	tripartite motif containing 28	7	Up	Nucleus	transcription regulator
USP7	ubiquitin specific peptidase 7	5	Up	Nucleus	peptidase

UVA-INDUCED MAPK SIGNALING

Symbol	Entrez Gene Name	Expr Other	Expected	Location	Type(s)
ATM	ATM serine/threonine kinase	5		Nucleus	kinase
BCL2L1	BCL2 like 1	4		Cytoplasm	other
CASP3	caspase 3	10		Cytoplasm	peptidase
CASP9	caspase 9	5		Cytoplasm	peptidase
EGFR	epidermal growth factor receptor	18	Up	Plasma Membrane	kinase
FGFR1	fibroblast growth factor receptor 1	4	Up	Plasma Membrane	kinase
FOS	Fos proto-oncogene, AP-1 transcription factor subunit	7		Nucleus	transcription regulator
GRB2	growth factor receptor bound protein 2	19	Up	Cytoplasm	kinase
JUN	Jun proto-oncogene, AP-1 transcription factor subunit	12		Nucleus	transcription regulator
MAPK1	mitogen-activated protein kinase 1	9	Up	Cytoplasm	kinase
MAPK3	mitogen-activated protein kinase 3	16	Up	Cytoplasm	kinase
MAPK8	mitogen-activated protein kinase 8	5		Cytoplasm	kinase
MAPK13	mitogen-activated protein kinase 13	7		Cytoplasm	kinase
MTOR	mechanistic target of rapamycin kinase	3		Nucleus	kinase
PARP1	poly(ADP-ribose) polymerase 1	4		Nucleus	enzyme
PDIA3	protein disulfide isomerase family A member 3	4	Up	Cytoplasm	peptidase
PIK3R1	phosphoinositide-3-kinase regulatory subunit 1	3	Up	Cytoplasm	kinase
PIK3R2	phosphoinositide-3-kinase regulatory subunit 2	6	Up	Cytoplasm	kinase
PLCB1	phospholipase C beta 1	4	Up	Cytoplasm	enzyme
PLCG1	phospholipase C gamma 1	4	Up	Cytoplasm	enzyme
PLCG2	phospholipase C gamma 2	3	Up	Cytoplasm	enzyme
PRKCA	protein kinase C alpha	3	Up	Cytoplasm	kinase
PTPN11	protein tyrosine phosphatase, non-receptor type 11	3	Up	Cytoplasm	phosphatase
RPS6KA1	ribosomal protein S6 kinase A1	3	Up	Cytoplasm	kinase
RPS6KB2	ribosomal protein S6 kinase B2	4	Up	Cytoplasm	kinase
STAT1	signal transducer and activator of transcription 1	9	Up	Nucleus	transcription regulator
TNKS2	tankyrase 2	3		Nucleus	enzyme
TP53	tumor protein p53	26		Nucleus	transcription regulator

TELOMERE EXTENSION BY TELOMERASE

Symbol	Entrez Gene Name	Expr Other	Expected	Location	Type(s)
HNRNPA1	heterogeneous nuclear ribonucleoprotein A1	15		Nucleus	enzyme
HNRNPA2B1	heterogeneous nuclear ribonucleoprotein A2/B1	12		Nucleus	other
RAD50	RAD50 double strand break repair protein	3		Nucleus	enzyme
TERF1	telomeric repeat binding factor 1	4		Nucleus	other
TERF2	telomeric repeat binding factor 2	3		Nucleus	other

TERF2IP	TERF2 interacting protein	4		Nucleus	other
TNKS2	tankyrase 2	3		Nucleus	enzyme
XRCC5	X-ray repair cross complementing 5	8		Nucleus	enzyme
XRCC6	X-ray repair cross complementing 6	5		Nucleus	enzyme

GADD45 SIGNALING

Symbol	Entrez Gene Name	Expr Other	Expected	Location	Type(s)
ATM	ATM serine/threonine kinase	5		Nucleus	kinase
BRCA1	BRCA1, DNA repair associated	9		Nucleus	transcription regulator
CCNB1	cyclin B1	7		Cytoplasm	kinase
CCND1	cyclin D1	6		Nucleus	transcription regulator
CDK1	cyclin dependent kinase 1	10		Nucleus	kinase
CDK2	cyclin dependent kinase 2	10		Nucleus	kinase
CDKN1A	cyclin dependent kinase inhibitor 1A	6		Nucleus	kinase
GADD45A	growth arrest and DNA damage inducible alpha	3		Nucleus	other
PCNA	proliferating cell nuclear antigen	4		Nucleus	enzyme
TP53	tumor protein p53	26		Nucleus	transcription regulator

DNA DAMAGE INDUCED 14-3-3 SIGNALING

Symbol	Entrez Gene Name	Expr Other	Expected	Location	Type(s)
AKT1	AKT serine/threonine kinase 1	7		Cytoplasm	kinase
ATM	ATM serine/threonine kinase	5		Nucleus	kinase
BRCA1	BRCA1, DNA repair associated	9		Nucleus	transcription regulator
CCNB1	cyclin B1	7		Cytoplasm	kinase
CDK1	cyclin dependent kinase 1	10		Nucleus	kinase
CDK2	cyclin dependent kinase 2	10		Nucleus	kinase
SFN	stratifin	3		Cytoplasm	other
TP53	tumor protein p53	26		Nucleus	transcription regulator

DNA DOUBLE STRAND BREAK REPAIR BY HOMOLOGOUS RECOMBINATION

Symbol	Entrez Gene Name	Expr Other	Expected	Location	Type(s)
ABL1	ABL proto-oncogene 1, non-receptor tyrosine kinase	6		Nucleus	kinase
ATM	ATM serine/threonine kinase	5		Nucleus	kinase
ATRX	ATRX, chromatin remodeler	4		Nucleus	transcription regulator
BRCA1	BRCA1, DNA repair associated	9		Nucleus	transcription regulator
RAD50	RAD50 double strand break repair protein	3		Nucleus	enzyme
RAD51	RAD51 recombinase	3		Nucleus	enzyme
RPA1	replication protein A1	9		Nucleus	other

DNA DOUBLE STRAND BREAK REPAIR BY NON-HOMOLOGOUS END JOINING

Symbol	Entrez Gene Name	Expr Other	Expected	Location	Type(s)

ATM	ATM serine/threonine kinase	5	Nucleus	kinase
LIG4	DNA ligase 4	3	Nucleus	enzyme
PARP1	poly(ADP-ribose) polymerase 1	4	Nucleus	enzyme
PRKDC	protein kinase, DNA-activated, catalytic polypeptide	5	Nucleus	kinase
RAD50	RAD50 double strand break repair protein	3	Nucleus	enzyme
XRCC5	X-ray repair cross complementing 5	8	Nucleus	enzyme
XRCC6	X-ray repair cross complementing 6	5	Nucleus	enzyme

Supplementary Table 9

Canonical pathways and proteins involved in cytoskeleton dynamics. The table includes, gene ID, Entrez Gene name, their total number of protein interactions, location of the protein and the protein type.

**CANONICAL PATHWAYS RELATED TO MAINTAINING THE CYTOSKELETAL DYNAMICS**					
**ILK SIGNALING**					
**Symbol**	**Entrez Gene Name**	**Expr Other**	**Expected**	**Location**	**Type(s)**
ACTA1	actin, alpha 1, skeletal muscle	4	Up	Cytoplasm	other
ACTB	actin beta	13	Up	Cytoplasm	other
ACTG1	actin gamma 1	4	Up	Cytoplasm	other
ACTN1	actinin alpha 1	5	Up	Cytoplasm	transcription regulator
ACTN2	actinin alpha 2	5	Up	Nucleus	transcription regulator
AKT1	AKT serine/threonine kinase 1	7	Up	Cytoplasm	kinase
ATF2	activating transcription factor 2	3	Up	Nucleus	transcription regulator
ATF4	activating transcription factor 4	6	Up	Nucleus	transcription regulator
ATM	ATM serine/threonine kinase	5	Up	Nucleus	kinase
CASP3	caspase 3	10	Down	Cytoplasm	peptidase
CCND1	cyclin D1	6	Up	Nucleus	transcription regulator
CDC42	cell division cycle 42	4	Up	Cytoplasm	enzyme
CDH1	cadherin 1	4	Up	Plasma Membrane	other
CFL1	cofilin 1	7	Up	Nucleus	other
CREB1	cAMP responsive element binding protein 1	11	Up	Nucleus	transcription regulator
CREBBP	CREB binding protein	8	Up	Nucleus	transcription regulator
CTNNB1	catenin beta 1	17	Up	Nucleus	transcription regulator
DSP	desmoplakin	4		Plasma Membrane	other
EP300	E1A binding protein p300	12	Up	Nucleus	transcription regulator
FGFR1	fibroblast growth factor receptor 1	4	Up	Plasma Membrane	kinase
FN1	fibronectin 1	11	Up	Extracellular Space	enzyme
FOS	Fos proto-oncogene, AP-1 transcription factor subunit	7	Up	Nucleus	transcription regulator
GRB2	growth factor receptor bound protein 2	19	Up	Cytoplasm	kinase
GSK3B	glycogen synthase kinase 3 beta	11		Nucleus	kinase
HIF1A	hypoxia inducible factor 1 alpha subunit	9	Up	Nucleus	transcription regulator
ILK	integrin linked kinase	6	Up	Plasma Membrane	kinase
ITGB1	integrin subunit beta 1	4	Up	Plasma Membrane	transmembrane receptor
ITGB3	integrin subunit beta 3	5	Up	Plasma Membrane	transmembrane receptor
ITGB4	integrin subunit beta 4	3	Up	Plasma Membrane	transmembrane receptor
JUN	Jun proto-oncogene, AP-1 transcription factor subunit	12	Up	Nucleus	transcription regulator
MAPK1	mitogen-activated protein kinase 1	9	Up	Cytoplasm	kinase
MAPK3	mitogen-activated protein kinase 3	16	Up	Cytoplasm	kinase
MAPK8	mitogen-activated protein kinase 8	5	Up	Cytoplasm	kinase
MTOR	mechanistic target of rapamycin kinase	3	Up	Nucleus	kinase
MYC	MYC proto-oncogene, bHLH transcription factor	18	Up	Nucleus	transcription regulator
MYH9	myosin heavy chain 9	7	Up	Cytoplasm	enzyme
MYH10	myosin heavy chain 10	5	Up	Cytoplasm	enzyme
MYH14	myosin heavy chain 14	3	Up	Extracellular Space	enzyme

NACA	nascent polypeptide-associated complex alpha subunit	3	Up	Cytoplasm	transcription regulator
NCK2	NCK adaptor protein 2	3	Up	Cytoplasm	kinase
NFKB1	nuclear factor kappa B subunit 1	6	Up	Nucleus	transcription regulator
NFKB2	nuclear factor kappa B subunit 2	4	Up	Nucleus	transcription regulator
NOS2	nitric oxide synthase 2	5		Cytoplasm	enzyme
PDPK1	3-phosphoinositide dependent protein kinase 1	3	Up	Cytoplasm	kinase
PIK3R1	phosphoinositide-3-kinase regulatory subunit 1	3	Up	Cytoplasm	kinase
PIK3R2	phosphoinositide-3-kinase regulatory subunit 2	6	Up	Cytoplasm	kinase
PPP1R12A	protein phosphatase 1 regulatory subunit 12A	3		Cytoplasm	phosphatase
PPP2CA	protein phosphatase 2 catalytic subunit alpha	3	Down	Cytoplasm	phosphatase
PPP2R1A	protein phosphatase 2 scaffold subunit Aalpha	8	Down	Cytoplasm	phosphatase
PPP2R1B	protein phosphatase 2 scaffold subunit Abeta	3	Down	Plasma Membrane	phosphatase
PPP2R2A	protein phosphatase 2 regulatory subunit Balpha	3	Down	Cytoplasm	phosphatase
PPP2R5D	protein phosphatase 2 regulatory subunit B'delta	3	Down	Nucleus	phosphatase
PTEN	phosphatase and tensin homolog	6	Down	Cytoplasm	phosphatase
PTGS2	prostaglandin-endoperoxide synthase 2	3		Cytoplasm	enzyme
PTK2	protein tyrosine kinase 2	3	Up	Cytoplasm	kinase
PTPN11	protein tyrosine phosphatase, non-receptor type 11	3	Up	Cytoplasm	phosphatase
RELA	RELA proto-oncogene, NF-kB subunit	10	Up	Nucleus	transcription regulator
TNF	tumor necrosis factor	7	Up	Extracellular Space	cytokine
TNFRSF1A	TNF receptor superfamily member 1A	3	Up	Plasma Membrane	transmembrane receptor
VCL	vinculin	8	Down	Plasma Membrane	enzyme
VEGFA	vascular endothelial growth factor A	5	Up	Extracellular Space	growth factor
VIM	vimentin	6		Cytoplasm	other

INTEGRIN SIGNALING

Symbol	Entrez Gene Name	Expr Other	Expected	Location	Type(s)
ABL1	ABL proto-oncogene 1, non-receptor tyrosine kinase	6	Up	Nucleus	kinase
ACTA1	actin, alpha 1, skeletal muscle	4	Up	Cytoplasm	other
ACTB	actin beta	13	Up	Cytoplasm	other
ACTG1	actin gamma 1	4	Up	Cytoplasm	other
ACTN1	actinin alpha 1	5	Up	Cytoplasm	transcription regulator
ACTN2	actinin alpha 2	5	Up	Nucleus	transcription regulator
AKT1	AKT serine/threonine kinase 1	7	Up	Cytoplasm	kinase
ARF6	ADP ribosylation factor 6	7	Down	Plasma Membrane	transporter
ATM	ATM serine/threonine kinase	5	Up	Nucleus	kinase
BCAR3	BCAR3, NSP family adaptor protein	3	Up	Cytoplasm	other
CAV1	caveolin 1	5	Up	Plasma Membrane	transmembrane receptor
CDC42	cell division cycle 42	4	Up	Cytoplasm	enzyme

CRK	CRK proto-oncogene, adaptor protein	5	Up	Cytoplasm	other
CRKL	CRK like proto-oncogene, adaptor protein	4	Up	Cytoplasm	kinase
FGFR1	fibroblast growth factor receptor 1	4	Up	Plasma Membrane	kinase
FYN	FYN proto-oncogene, Src family tyrosine kinase	7	Up	Plasma Membrane	kinase
GRB2	growth factor receptor bound protein 2	19	Up	Cytoplasm	kinase
GRB7	growth factor receptor bound protein 7	3	Up	Plasma Membrane	other
GSK3B	glycogen synthase kinase 3 beta	11	Down	Nucleus	kinase
ILK	integrin linked kinase	6	Up	Plasma Membrane	kinase
ITGA4	integrin subunit alpha 4	11	Up	Plasma Membrane	transmembrane receptor
ITGAV	integrin subunit alpha V	3	Up	Plasma Membrane	transmembrane receptor
ITGB1	integrin subunit beta 1	4	Up	Plasma Membrane	transmembrane receptor
ITGB3	integrin subunit beta 3	5	Up	Plasma Membrane	transmembrane receptor
ITGB4	integrin subunit beta 4	3	Up	Plasma Membrane	transmembrane receptor
MAP2K1	mitogen-activated protein kinase kinase 1	3	Up	Cytoplasm	kinase
MAPK1	mitogen-activated protein kinase 1	9	Up	Cytoplasm	kinase
MAPK3	mitogen-activated protein kinase 3	16	Up	Cytoplasm	kinase
MAPK8	mitogen-activated protein kinase 8	5	Up	Cytoplasm	kinase
NCK1	NCK adaptor protein 1	5	Up	Cytoplasm	kinase
NCK2	NCK adaptor protein 2	3	Up	Cytoplasm	kinase
PFN1	profilin 1	7	Up	Cytoplasm	other
PIK3R1	phosphoinositide-3-kinase regulatory subunit 1	3	Up	Cytoplasm	kinase
PIK3R2	phosphoinositide-3-kinase regulatory subunit 2	6	Up	Cytoplasm	kinase
PLCG1	phospholipase C gamma 1	4	Up	Cytoplasm	enzyme
PLCG2	phospholipase C gamma 2	3	Up	Cytoplasm	enzyme
PPP1CB	protein phosphatase 1 catalytic subunit beta	4		Cytoplasm	phosphatase
PPP1R12A	protein phosphatase 1 regulatory subunit 12A	3		Cytoplasm	phosphatase
PTEN	phosphatase and tensin homolog	6	Down	Cytoplasm	phosphatase
PTK2	protein tyrosine kinase 2	3	Up	Cytoplasm	kinase
PTPN11	protein tyrosine phosphatase, non-receptor type 11	3	Up	Cytoplasm	phosphatase
RAC1	Rac family small GTPase 1	6	Up	Plasma Membrane	enzyme
RAF1	Raf-1 proto-oncogene, serine/threonine kinase	8	Up	Cytoplasm	kinase
SHC1	SHC adaptor protein 1	5	Up	Cytoplasm	other
SRC	SRC proto-oncogene, non-receptor tyrosine kinase	10	Up	Cytoplasm	kinase
TSPAN3	tetraspanin 3	3		Plasma Membrane	other
VCL	vinculin	8	Up	Plasma Membrane	enzyme

HIPPO SIGNALING

Symbol	Entrez Gene Name	Expr Other	Expected	Location	Type(s)
BTRC	beta-transducin repeat containing E3 ubiquitin protein ligase	4		Cytoplasm	enzyme

CD44	CD44 molecule (Indian blood group)	5	Up	Plasma Membrane	other
CUL1	cullin 1	12		Nucleus	enzyme
DLG1	discs large MAGUK scaffold protein 1	3		Plasma Membrane	kinase
DLG4	discs large MAGUK scaffold protein 4	10		Plasma Membrane	kinase
PPP1CA	protein phosphatase 1 catalytic subunit alpha	8		Cytoplasm	phosphatase
PPP1CB	protein phosphatase 1 catalytic subunit beta	4		Cytoplasm	phosphatase
PPP1CC	protein phosphatase 1 catalytic subunit gamma	6		Nucleus	phosphatase
PPP1R12A	protein phosphatase 1 regulatory subunit 12A	3		Cytoplasm	phosphatase
PPP2CA	protein phosphatase 2 catalytic subunit alpha	3	Down	Cytoplasm	phosphatase
PPP2R1A	protein phosphatase 2 scaffold subunit Aalpha	8	Down	Cytoplasm	phosphatase
PPP2R1B	protein phosphatase 2 scaffold subunit Abeta	3	Down	Plasma Membrane	phosphatase
PPP2R2A	protein phosphatase 2 regulatory subunit Balpha	3	Down	Cytoplasm	phosphatase
PPP2R5D	protein phosphatase 2 regulatory subunit B'delta	3	Down	Nucleus	phosphatase
PRKCZ	protein kinase C zeta	3	Down	Cytoplasm	kinase
SFN	stratifin	3	Down	Cytoplasm	other
SKP1	S-phase kinase associated protein 1	3		Nucleus	transcription regulator
SMAD1	SMAD family member 1	7		Nucleus	transcription regulator
SMAD2	SMAD family member 2	6		Nucleus	transcription regulator
SMAD3	SMAD family member 3	5		Nucleus	transcription regulator
SMAD4	SMAD family member 4	4		Nucleus	transcription regulator
STK4	serine/threonine kinase 4	4	Up	Cytoplasm	kinase
TAZ	tafazzin	3	Down	Nucleus	enzyme
YAP1	Yes associated protein 1	5	Down	Nucleus	transcription regulator
YWHAB	tyrosine 3-monooxygenase/tryptophan 5-monooxygenase activat	6	Down	Cytoplasm	transcription regulator
YWHAE	tyrosine 3-monooxygenase/tryptophan 5-monooxygenase activat	11	Down	Cytoplasm	other
YWHAG	tyrosine 3-monooxygenase/tryptophan 5-monooxygenase activat	9	Down	Cytoplasm	other
YWHAH	tyrosine 3-monooxygenase/tryptophan 5-monooxygenase activat	7	Down	Cytoplasm	transcription regulator
YWHAQ	tyrosine 3-monooxygenase/tryptophan 5-monooxygenase activat	14	Down	Cytoplasm	other
YWHAZ	tyrosine 3-monooxygenase/tryptophan 5-monooxygenase activat	16	Down	Cytoplasm	enzyme

ACTIN CYTOSKELETON SIGNALING

Symbol	Entrez Gene Name	Expr Other	Expected	Location	Type(s)
ACTA1	actin, alpha 1, skeletal muscle	4	Up	Cytoplasm	other
ACTB	actin beta	13	Up	Cytoplasm	other
ACTG1	actin gamma 1	4	Up	Cytoplasm	other
ACTN1	actinin alpha 1	5	Up	Cytoplasm	transcription regulator
ACTN2	actinin alpha 2	5	Up	Nucleus	transcription regulator
APC	APC, WNT signaling pathway regulator	4	Up	Nucleus	enzyme
ATM	ATM serine/threonine kinase	5	Up	Nucleus	kinase
CDC42	cell division cycle 42	4	Up	Cytoplasm	enzyme
CFL1	cofilin 1	7	Down	Nucleus	other

CRK	CRK proto-oncogene, adaptor protein	5	Up	Cytoplasm	other
CRKL	CRK like proto-oncogene, adaptor protein	4	Up	Cytoplasm	kinase
CYFIP2	cytoplasmic FMR1 interacting protein 2	3		Cytoplasm	other
DIAPH3	diaphanous related formin 3	3	Up	Cytoplasm	enzyme
EZR	ezrin	4	Up	Plasma Membrane	other
F2	coagulation factor II, thrombin	3	Up	Extracellular Space	peptidase
FGFR1	fibroblast growth factor receptor 1	4	Up	Plasma Membrane	kinase
FN1	fibronectin 1	11	Up	Extracellular Space	enzyme
GRB2	growth factor receptor bound protein 2	19	Up	Cytoplasm	kinase
IQGAP1	IQ motif containing GTPase activating protein 1	4	Down	Cytoplasm	other
ITGA4	integrin subunit alpha 4	11	Up	Plasma Membrane	transmembrane receptor
ITGB1	integrin subunit beta 1	4	Up	Plasma Membrane	transmembrane receptor
MAP2K1	mitogen-activated protein kinase kinase 1	3	Up	Cytoplasm	kinase
MAPK1	mitogen-activated protein kinase 1	9	Up	Cytoplasm	kinase
MAPK3	mitogen-activated protein kinase 3	16	Up	Cytoplasm	kinase
MSN	moesin	4	Up	Plasma Membrane	other
MYH9	myosin heavy chain 9	7	Up	Cytoplasm	enzyme
MYH10	myosin heavy chain 10	5	Up	Cytoplasm	enzyme
MYH14	myosin heavy chain 14	3	Up	Extracellular Space	enzyme
NCKAP1	NCK associated protein 1	3		Plasma Membrane	other
PFN1	profilin 1	7	Up	Cytoplasm	other
PIK3R1	phosphoinositide-3-kinase regulatory subunit 1	3	Up	Cytoplasm	kinase
PIK3R2	phosphoinositide-3-kinase regulatory subunit 2	6	Up	Cytoplasm	kinase
PPP1CB	protein phosphatase 1 catalytic subunit beta	4	Down	Cytoplasm	phosphatase
PPP1R12A	protein phosphatase 1 regulatory subunit 12A	3	Down	Cytoplasm	phosphatase
PTK2	protein tyrosine kinase 2	3	Up	Cytoplasm	kinase
PTPN11	protein tyrosine phosphatase, non-receptor type 11	3	Up	Cytoplasm	phosphatase
RAC1	Rac family small GTPase 1	6	Up	Plasma Membrane	enzyme
RAF1	Raf-1 proto-oncogene, serine/threonine kinase	8	Up	Cytoplasm	kinase
SHC1	SHC adaptor protein 1	5	Up	Cytoplasm	other
VAV2	vav guanine nucleotide exchange factor 2	5	Up	Cytoplasm	transcription regulator
VCL	vinculin	8	Up	Plasma Membrane	enzyme

FAK SIGNALING

Symbol	Entrez Gene Name	Expr Other	Expected	Location	Type(s)
ACTA1	actin, alpha 1, skeletal muscle	4		Cytoplasm	other

ACTB	actin beta	13		Cytoplasm	other
ACTG1	actin gamma 1	4		Cytoplasm	other
AKT1	AKT serine/threonine kinase 1	7		Cytoplasm	kinase
ATM	ATM serine/threonine kinase	5		Nucleus	kinase
CRK	CRK proto-oncogene, adaptor protein	5		Cytoplasm	other
EGFR	epidermal growth factor receptor	18		Plasma Membrane	kinase
FGFR1	fibroblast growth factor receptor 1	4		Plasma Membrane	kinase
FYN	FYN proto-oncogene, Src family tyrosine kinase	7		Plasma Membrane	kinase
GRB2	growth factor receptor bound protein 2	19		Cytoplasm	kinase
HMMR	hyaluronan mediated motility receptor	3		Plasma Membrane	transmembrane receptor
ITGA4	integrin subunit alpha 4	11		Plasma Membrane	transmembrane receptor
ITGB1	integrin subunit beta 1	4		Plasma Membrane	transmembrane receptor
MAP2K1	mitogen-activated protein kinase kinase 1	3		Cytoplasm	kinase
MAPK1	mitogen-activated protein kinase 1	9		Cytoplasm	kinase
MAPK3	mitogen-activated protein kinase 3	16		Cytoplasm	kinase
PDPK1	3-phosphoinositide dependent protein kinase 1	3		Cytoplasm	kinase
PIK3R1	phosphoinositide-3-kinase regulatory subunit 1	3		Cytoplasm	kinase
PIK3R2	phosphoinositide-3-kinase regulatory subunit 2	6		Cytoplasm	kinase
PLCG1	phospholipase C gamma 1	4		Cytoplasm	enzyme
PLCG2	phospholipase C gamma 2	3		Cytoplasm	enzyme
PTEN	phosphatase and tensin homolog	6		Cytoplasm	phosphatase
PTK2	protein tyrosine kinase 2	3		Cytoplasm	kinase
PTPN11	protein tyrosine phosphatase, non-receptor type 11	3		Cytoplasm	phosphatase
RAC1	Rac family small GTPase 1	6		Plasma Membrane	enzyme
RAF1	Raf-1 proto-oncogene, serine/threonine kinase	8		Cytoplasm	kinase
SRC	SRC proto-oncogene, non-receptor tyrosine kinase	10		Cytoplasm	kinase
VCL	vinculin	8		Plasma Membrane	enzyme

PAXILLIN SIGNALING

Symbol	Entrez Gene Name	Expr Other	Expected	Location	Type(s)
ACTA1	actin, alpha 1, skeletal muscle	4	Up	Cytoplasm	other
ACTB	actin beta	13	Up	Cytoplasm	other
ACTG1	actin gamma 1	4	Up	Cytoplasm	other
ACTN1	actinin alpha 1	5	Up	Cytoplasm	transcription regulator
ACTN2	actinin alpha 2	5	Up	Nucleus	transcription regulator
ARF6	ADP ribosylation factor 6	7	Up	Plasma Membrane	transporter
ATM	ATM serine/threonine kinase	5	Up	Nucleus	kinase
CDC42	cell division cycle 42	4	Up	Cytoplasm	enzyme
CRK	CRK proto-oncogene, adaptor protein	5	Up	Cytoplasm	other
FGFR1	fibroblast growth factor receptor 1	4	Up	Plasma Membrane	kinase
GRB2	growth factor receptor bound protein 2	19	Up	Cytoplasm	kinase

ITGA4	integrin subunit alpha 4	11	Up	Plasma Membrane	transmembrane receptor
ITGAV	integrin subunit alpha V	3		Plasma Membrane	transmembrane receptor
ITGB1	integrin subunit beta 1	4	Up	Plasma Membrane	transmembrane receptor
ITGB3	integrin subunit beta 3	5		Plasma Membrane	transmembrane receptor
ITGB4	integrin subunit beta 4	3		Plasma Membrane	transmembrane receptor
MAPK1	mitogen-activated protein kinase 1	9	Up	Cytoplasm	kinase
MAPK8	mitogen-activated protein kinase 8	5	Up	Cytoplasm	kinase
MAPK13	mitogen-activated protein kinase 13	7	Up	Cytoplasm	kinase
NCK1	NCK adaptor protein 1	5	Up	Cytoplasm	kinase
NCK2	NCK adaptor protein 2	3	Up	Cytoplasm	kinase
PIK3R1	phosphoinositide-3-kinase regulatory subunit 1	3	Up	Cytoplasm	kinase
PIK3R2	phosphoinositide-3-kinase regulatory subunit 2	6	Up	Cytoplasm	kinase
PTK2	protein tyrosine kinase 2	3	Up	Cytoplasm	kinase
PTK2B	protein tyrosine kinase 2 beta	3	Up	Cytoplasm	kinase
PTPN11	protein tyrosine phosphatase, non-receptor type 11	3	Up	Cytoplasm	phosphatase
RAC1	Rac family small GTPase 1	6	Up	Plasma Membrane	enzyme
SRC	SRC proto-oncogene, non-receptor tyrosine kinase	10	Up	Cytoplasm	kinase
VCL	vinculin	8	Up	Plasma Membrane	enzyme

EPHRIN RECEPTOR SIGNALING

Symbol	Entrez Gene Name	Expr Other	Expected	Location	Type(s)
ABL1	ABL proto-oncogene 1, non-receptor tyrosine kinase	6	Up	Nucleus	kinase
AKT1	AKT serine/threonine kinase 1	7	Up	Cytoplasm	kinase
ATF2	activating transcription factor 2	3	Up	Nucleus	transcription regulator
ATF4	activating transcription factor 4	6	Up	Nucleus	transcription regulator
CDC42	cell division cycle 42	4	Up	Cytoplasm	enzyme
CFL1	cofilin 1	7	Up	Nucleus	other
CREB1	cAMP responsive element binding protein 1	11	Up	Nucleus	transcription regulator
CREBBP	CREB binding protein	8	Up	Nucleus	transcription regulator
CRK	CRK proto-oncogene, adaptor protein	5	Up	Cytoplasm	other
CRKL	CRK like proto-oncogene, adaptor protein	4	Up	Cytoplasm	kinase
EFNB2	ephrin B2	3		Plasma Membrane	kinase
EP300	E1A binding protein p300	12	Up	Nucleus	transcription regulator
EPHA4	EPH receptor A4	3		Plasma Membrane	kinase
FYN	FYN proto-oncogene, Src family tyrosine kinase	7	Up	Plasma Membrane	kinase
GNB1	G protein subunit beta 1	3	Up	Plasma Membrane	enzyme
GRB2	growth factor receptor bound protein 2	19	Up	Cytoplasm	kinase
GRIN1	glutamate ionotropic receptor NMDA type subunit 1	6	Up	Plasma Membrane	ion channel
ITGA4	integrin subunit alpha 4	11	Up	Plasma Membrane	transmembrane receptor
ITGB1	integrin subunit beta 1	4	Up	Plasma Membrane	transmembrane receptor
MAP2K1	mitogen-activated protein kinase kinase 1	3	Up	Cytoplasm	kinase

MAPK1	mitogen-activated protein kinase 1	9	Up	Cytoplasm	kinase
MAPK3	mitogen-activated protein kinase 3	16	Up	Cytoplasm	kinase
NCK1	NCK adaptor protein 1	5	Up	Cytoplasm	kinase
NCK2	NCK adaptor protein 2	3	Up	Cytoplasm	kinase
PTK2	protein tyrosine kinase 2	3	Up	Cytoplasm	kinase
PTPN11	protein tyrosine phosphatase, non-receptor type 11	3	Up	Cytoplasm	phosphatase
RAC1	Rac family small GTPase 1	6	Up	Plasma Membrane	enzyme
RACK1	receptor for activated C kinase 1	7	Up	Cytoplasm	enzyme
RAF1	Raf-1 proto-oncogene, serine/threonine kinase	8	Up	Cytoplasm	kinase
RASA1	RAS p21 protein activator 1	4		Cytoplasm	transporter
SHC1	SHC adaptor protein 1	5	Up	Cytoplasm	other
SRC	SRC proto-oncogene, non-receptor tyrosine kinase	10	Up	Cytoplasm	kinase
STAT3	signal transducer and activator of transcription 3	6	Up	Nucleus	transcription regulator
VEGFA	vascular endothelial growth factor A	5		Extracellular Space	growth factor

Supplementary Table 10

Canonical pathways and proteins involved with growth factors. The table includes, gene ID, Entrez Gene name, their total number of protein interactions, location of the protein and the protein type.

CANONICAL PATHWAYS RELATED TO GROWTH FACTOR SIGNALING					
**NEUREGULIN SIGNALING**					
**Symbol**	**Entrez Gene Name**	**Expr Other**	**Expected**	**Location**	**Type(s)**
AKT1	AKT serine/threonine kinase 1	7	Up	Cytoplasm	kinase
CDK5	cyclin dependent kinase 5	6	Up	Nucleus	kinase
CDK5R1	cyclin dependent kinase 5 regulatory subunit 1	3	Up	Nucleus	kinase
CRK	CRK proto-oncogene, adaptor protein	5		Cytoplasm	other
CRKL	CRK like proto-oncogene, adaptor protein	4		Cytoplasm	kinase
DLG4	discs large MAGUK scaffold protein 4	10	Up	Plasma Membrane	kinase
EGFR	epidermal growth factor receptor	18	Up	Plasma Membrane	kinase
ERBB2	erb-b2 receptor tyrosine kinase 2	3	Up	Plasma Membrane	kinase
ERBB3	erb-b2 receptor tyrosine kinase 3	4	Up	Plasma Membrane	kinase
ERBB4	erb-b2 receptor tyrosine kinase 4	3	Up	Plasma Membrane	kinase
GRB2	growth factor receptor bound protein 2	19	Up	Cytoplasm	kinase
GRB7	growth factor receptor bound protein 7	3	Up	Plasma Membrane	other
HBEGF	heparin binding EGF like growth factor	4	Up	Extracellular Space	growth factor
HSP90AA1	heat shock protein 90 alpha family class A member 1	11		Cytoplasm	enzyme
HSP90AB1	heat shock protein 90 alpha family class B member 1	9		Cytoplasm	enzyme
HSP90B1	heat shock protein 90 beta family member 1	5		Cytoplasm	other
ITGA4	integrin subunit alpha 4	11		Plasma Membrane	transmembrane receptor
ITGB1	integrin subunit beta 1	4		Plasma Membrane	transmembrane receptor
MAP2K1	mitogen-activated protein kinase kinase 1	3	Up	Cytoplasm	kinase
MAPK1	mitogen-activated protein kinase 1	9	Up	Cytoplasm	kinase
MAPK3	mitogen-activated protein kinase 3	16	Up	Cytoplasm	kinase
MTOR	mechanistic target of rapamycin kinase	3	Up	Nucleus	kinase
MYC	MYC proto-oncogene, bHLH transcription factor	18	Up	Nucleus	transcription regulator
PDPK1	3-phosphoinositide dependent protein kinase 1	3	Up	Cytoplasm	kinase
PIK3R1	phosphoinositide-3-kinase regulatory subunit 1	3	Up	Cytoplasm	kinase
PIK3R2	phosphoinositide-3-kinase regulatory subunit 2	6	Up	Cytoplasm	kinase
PLCG1	phospholipase C gamma 1	4	Up	Cytoplasm	enzyme
PLCG2	phospholipase C gamma 2	3	Up	Cytoplasm	enzyme
PRKCA	protein kinase C alpha	3	Up	Cytoplasm	kinase
PRKCB	protein kinase C beta	5	Up	Cytoplasm	kinase
PRKCD	protein kinase C delta	3	Up	Cytoplasm	kinase
PRKCG	protein kinase C gamma	5	Up	Cytoplasm	kinase
PRKCZ	protein kinase C zeta	3	Up	Cytoplasm	kinase
PSEN1	presenilin 1	5	Up	Plasma Membrane	peptidase
PTEN	phosphatase and tensin homolog	6	Down	Cytoplasm	phosphatase
PTPN11	protein tyrosine phosphatase, non-receptor type 11	3	Up	Cytoplasm	phosphatase
RAF1	Raf-1 proto-oncogene, serine/threonine kinase	8	Up	Cytoplasm	kinase
RNF41	ring finger protein 41	4	Down	Cytoplasm	enzyme

RPS6	ribosomal protein S6	4	Up	Cytoplasm	other
RPS6KB2	ribosomal protein S6 kinase B2	4	Up	Cytoplasm	kinase
SHC1	SHC adaptor protein 1	5	Up	Cytoplasm	other
SRC	SRC proto-oncogene, non-receptor tyrosine kinase	10	Up	Cytoplasm	kinase

PTEN SIGNALING

Symbol	Entrez Gene Name	Expr Other	Expected	Location	Type(s)
AKT1	AKT serine/threonine kinase 1	7	Down	Cytoplasm	kinase
BCL2	BCL2, apoptosis regulator	6	Down	Cytoplasm	transporter
BCL2L1	BCL2 like 1	4	Down	Cytoplasm	other
BMPR1A	bone morphogenetic protein receptor type 1A	5	Down	Plasma Membrane	kinase
CASP3	caspase 3	10	Up	Cytoplasm	peptidase
CASP9	caspase 9	5	Up	Cytoplasm	peptidase
CBL	Cbl proto-oncogene	4		Nucleus	transcription regulator
CCND1	cyclin D1	6	Down	Nucleus	transcription regulator
CDC42	cell division cycle 42	4	Down	Cytoplasm	enzyme
CDKN1A	cyclin dependent kinase inhibitor 1A	6	Up	Nucleus	kinase
CSNK2A1	casein kinase 2 alpha 1	6	Down	Nucleus	kinase
CSNK2B	casein kinase 2 beta	4	Down	Cytoplasm	kinase
EGFR	epidermal growth factor receptor	18	Down	Plasma Membrane	kinase
FGFR1	fibroblast growth factor receptor 1	4	Down	Plasma Membrane	kinase
FOXO1	forkhead box O1	4	Down	Nucleus	transcription regulator
FOXO3	forkhead box O3	3	Down	Nucleus	transcription regulator
FOXO4	forkhead box O4	4	Down	Nucleus	transcription regulator
GRB2	growth factor receptor bound protein 2	19	Down	Cytoplasm	kinase
GSK3B	glycogen synthase kinase 3 beta	11	Up	Nucleus	kinase
IKBKE	inhibitor of nuclear factor kappa B kinase subunit epsilon	4	Down	Cytoplasm	kinase
IKBKG	inhibitor of nuclear factor kappa B kinase subunit gamma	4	Down	Nucleus	kinase
ILK	integrin linked kinase	6	Down	Plasma Membrane	kinase
INSR	insulin receptor	6	Down	Plasma Membrane	kinase
ITGA4	integrin subunit alpha 4	11	Down	Plasma Membrane	transmembrane receptor
ITGB1	integrin subunit beta 1	4	Down	Plasma Membrane	transmembrane receptor
KDR	kinase insert domain receptor	4	Down	Plasma Membrane	kinase
MAP2K1	mitogen-activated protein kinase kinase 1	3	Down	Cytoplasm	kinase
MAPK1	mitogen-activated protein kinase 1	9	Down	Cytoplasm	kinase
MAPK3	mitogen-activated protein kinase 3	16	Down	Cytoplasm	kinase
NFKB1	nuclear factor kappa B subunit 1	6	Down	Nucleus	transcription regulator
NFKB2	nuclear factor kappa B subunit 2	4	Down	Nucleus	transcription regulator
NGFR	nerve growth factor receptor	5	Down	Plasma Membrane	transmembrane receptor

NTRK1	neurotrophic receptor tyrosine kinase 1	26	Down	Plasma Membrane	kinase
PDGFRA	platelet derived growth factor receptor alpha	3	Down	Plasma Membrane	kinase
PDPK1	3-phosphoinositide dependent protein kinase 1	3	Down	Cytoplasm	kinase
PIK3R1	phosphoinositide-3-kinase regulatory subunit 1	3		Cytoplasm	kinase
PIK3R2	phosphoinositide-3-kinase regulatory subunit 2	6		Cytoplasm	kinase
PRKCZ	protein kinase C zeta	3	Down	Cytoplasm	kinase
PTEN	phosphatase and tensin homolog	6	Up	Cytoplasm	phosphatase
PTK2	protein tyrosine kinase 2	3	Down	Cytoplasm	kinase
RAC1	Rac family small GTPase 1	6	Down	Plasma Membrane	enzyme
RAF1	Raf-1 proto-oncogene, serine/threonine kinase	8	Down	Cytoplasm	kinase
RELA	RELA proto-oncogene, NF-kB subunit	10	Down	Nucleus	transcription regulator
RPS6KB2	ribosomal protein S6 kinase B2	4	Down	Cytoplasm	kinase
SHC1	SHC adaptor protein 1	5	Down	Cytoplasm	other
YWHAH	tyrosine 3-monooxygenase/tryptophan 5-monooxygenase activa	7	Down	Cytoplasm	transcription regulator

VEGF SIGNALING

Symbol	Entrez Gene Name	Expr Other	Expected	Location	Type(s)
ACTA1	actin, alpha 1, skeletal muscle	4	Up	Cytoplasm	other
ACTB	actin beta	13	Up	Cytoplasm	other
ACTG1	actin gamma 1	4	Up	Cytoplasm	other
ACTN1	actinin alpha 1	5	Up	Cytoplasm	transcription regulator
ACTN2	actinin alpha 2	5	Up	Nucleus	transcription regulator
AKT1	AKT serine/threonine kinase 1	7	Up	Cytoplasm	kinase
ATM	ATM serine/threonine kinase	5	Up	Nucleus	kinase
BCL2	BCL2, apoptosis regulator	6	Up	Cytoplasm	transporter
BCL2L1	BCL2 like 1	4	Up	Cytoplasm	other
EIF2B1	eukaryotic translation initiation factor 2B subunit alpha	3		Cytoplasm	translation regulator
EIF2B2	eukaryotic translation initiation factor 2B subunit beta	3		Cytoplasm	other
EIF2S3	eukaryotic translation initiation factor 2 subunit gamma	3		Cytoplasm	translation regulator
ELAVL1	ELAV like RNA binding protein 1	18		Cytoplasm	other
FGFR1	fibroblast growth factor receptor 1	4	Up	Plasma Membrane	kinase
FOXO1	forkhead box O1	4		Nucleus	transcription regulator
FOXO3	forkhead box O3	3		Nucleus	transcription regulator
GRB2	growth factor receptor bound protein 2	19	Up	Cytoplasm	kinase
HIF1A	hypoxia inducible factor 1 alpha subunit	9	Up	Nucleus	transcription regulator
KDR	kinase insert domain receptor	4	Up	Plasma Membrane	kinase
MAP2K1	mitogen-activated protein kinase kinase 1	3	Up	Cytoplasm	kinase
MAPK1	mitogen-activated protein kinase 1	9	Up	Cytoplasm	kinase
MAPK3	mitogen-activated protein kinase 3	16	Up	Cytoplasm	kinase
NOS3	nitric oxide synthase 3	5	Up	Cytoplasm	enzyme
PIK3R1	phosphoinositide-3-kinase regulatory subunit 1	3	Up	Cytoplasm	kinase

PIK3R2	phosphoinositide-3-kinase regulatory subunit 2	6	Up	Cytoplasm	kinase
PLCG1	phospholipase C gamma 1	4	Up	Cytoplasm	enzyme
PLCG2	phospholipase C gamma 2	3	Up	Cytoplasm	enzyme
PRKCA	protein kinase C alpha	3	Down	Cytoplasm	kinase
PRKCB	protein kinase C beta	5	Down	Cytoplasm	kinase
PTK2	protein tyrosine kinase 2	3	Up	Cytoplasm	kinase
PTK2B	protein tyrosine kinase 2 beta	3	Up	Cytoplasm	kinase
PTPN11	protein tyrosine phosphatase, non-receptor type 11	3	Down	Cytoplasm	phosphatase
RAF1	Raf-1 proto-oncogene, serine/threonine kinase	8	Up	Cytoplasm	kinase
SFN	stratifin	3	Up	Cytoplasm	other
SH2D2A	SH2 domain containing 2A	4	Up	Cytoplasm	other
SHC1	SHC adaptor protein 1	5	Up	Cytoplasm	other
SRC	SRC proto-oncogene, non-receptor tyrosine kinase	10	Up	Cytoplasm	kinase
VCL	vinculin	8	Down	Plasma Membrane	enzyme
VEGFA	vascular endothelial growth factor A	5	Up	Extracellular Space	growth factor
YWHAE	tyrosine 3-monooxygenase/tryptophan 5-monooxygenase activa	11		Cytoplasm	other

NGF SIGNALING

Symbol	Entrez Gene Name	Expr Other	Expected	Location	Type(s)
AKT1	AKT serine/threonine kinase 1	7	Up	Cytoplasm	kinase
ATF2	activating transcription factor 2	3	Up	Nucleus	transcription regulator
ATF4	activating transcription factor 4	6	Up	Nucleus	transcription regulator
ATM	ATM serine/threonine kinase	5	Up	Nucleus	kinase
BAX	BCL2 associated X, apoptosis regulator	3	Up	Cytoplasm	transporter
CDC42	cell division cycle 42	4	Up	Cytoplasm	enzyme
CREB1	cAMP responsive element binding protein 1	11	Up	Nucleus	transcription regulator
CREBBP	CREB binding protein	8	Up	Nucleus	transcription regulator
CRK	CRK proto-oncogene, adaptor protein	5	Up	Cytoplasm	other
EP300	E1A binding protein p300	12	Up	Nucleus	transcription regulator
FGFR1	fibroblast growth factor receptor 1	4	Up	Plasma Membrane	kinase
GRB2	growth factor receptor bound protein 2	19	Up	Cytoplasm	kinase
IKBKE	inhibitor of nuclear factor kappa B kinase subunit epsilon	4	Up	Cytoplasm	kinase
IKBKG	inhibitor of nuclear factor kappa B kinase subunit gamma	4	Up	Nucleus	kinase
MAP2K1	mitogen-activated protein kinase kinase 1	3	Up	Cytoplasm	kinase
MAP3K3	mitogen-activated protein kinase kinase kinase 3	5	Up	Cytoplasm	kinase
MAP3K5	mitogen-activated protein kinase kinase kinase 5	4	Up	Cytoplasm	kinase
MAP3K7	mitogen-activated protein kinase kinase kinase 7	3	Up	Cytoplasm	kinase
MAPK1	mitogen-activated protein kinase 1	9	Up	Cytoplasm	kinase
MAPK3	mitogen-activated protein kinase 3	16	Up	Cytoplasm	kinase
MAPK8	mitogen-activated protein kinase 8	5	Up	Cytoplasm	kinase
NFKB1	nuclear factor kappa B subunit 1	6	Up	Nucleus	transcription regulator

NFKB2	nuclear factor kappa B subunit 2	4	Up	Nucleus	transcription regulator
NGFR	nerve growth factor receptor	5	Up	Plasma Membrane	transmembrane receptor
NTRK1	neurotrophic receptor tyrosine kinase 1	26	Up	Plasma Membrane	kinase
PDPK1	3-phosphoinositide dependent protein kinase 1	3	Up	Cytoplasm	kinase
PIK3R1	phosphoinositide-3-kinase regulatory subunit 1	3	Up	Cytoplasm	kinase
PIK3R2	phosphoinositide-3-kinase regulatory subunit 2	6	Up	Cytoplasm	kinase
PLCG1	phospholipase C gamma 1	4	Up	Cytoplasm	enzyme
PLCG2	phospholipase C gamma 2	3	Up	Cytoplasm	enzyme
PRKCD	protein kinase C delta	3	Up	Cytoplasm	kinase
PRKCZ	protein kinase C zeta	3	Up	Cytoplasm	kinase
PTPN11	protein tyrosine phosphatase, non-receptor type 11	3	Up	Cytoplasm	phosphatase
RAC1	Rac family small GTPase 1	6	Up	Plasma Membrane	enzyme
RAF1	Raf-1 proto-oncogene, serine/threonine kinase	8	Up	Cytoplasm	kinase
RELA	RELA proto-oncogene, NF-kB subunit	10	Up	Nucleus	transcription regulator
RPS6KA1	ribosomal protein S6 kinase A1	3	Up	Cytoplasm	kinase
RPS6KB2	ribosomal protein S6 kinase B2	4	Up	Cytoplasm	kinase
SHC1	SHC adaptor protein 1	5	Up	Cytoplasm	other
TP53	tumor protein p53	26	Up	Nucleus	transcription regulator
TRAF6	TNF receptor associated factor 6	11	Up	Cytoplasm	enzyme

IGF-1 SIGNALING

Symbol	Entrez Gene Name	Expr Other	Expected	Location	Type(s)
AKT1	AKT serine/threonine kinase 1	7	Up	Cytoplasm	kinase
ATM	ATM serine/threonine kinase	5	Up	Nucleus	kinase
CASP9	caspase 9	5	Down	Cytoplasm	peptidase
CSNK2A1	casein kinase 2 alpha 1	6	Up	Nucleus	kinase
CSNK2B	casein kinase 2 beta	4	Up	Cytoplasm	kinase
FGFR1	fibroblast growth factor receptor 1	4	Up	Plasma Membrane	kinase
FOS	Fos proto-oncogene, AP-1 transcription factor subunit	7	Up	Nucleus	transcription regulator
FOXO1	forkhead box O1	4		Nucleus	transcription regulator
FOXO3	forkhead box O3	3		Nucleus	transcription regulator
GRB2	growth factor receptor bound protein 2	19	Up	Cytoplasm	kinase
GRB10	growth factor receptor bound protein 10	3	Up	Cytoplasm	other
JUN	Jun proto-oncogene, AP-1 transcription factor subunit	12	Up	Nucleus	transcription regulator
MAP2K1	mitogen-activated protein kinase kinase 1	3	Up	Cytoplasm	kinase
MAPK1	mitogen-activated protein kinase 1	9	Up	Cytoplasm	kinase
MAPK3	mitogen-activated protein kinase 3	16	Up	Cytoplasm	kinase
MAPK8	mitogen-activated protein kinase 8	5	Up	Cytoplasm	kinase
NEDD4	neural precursor cell expressed, developmentally down-regulate	5		Cytoplasm	enzyme
PDPK1	3-phosphoinositide dependent protein kinase 1	3	Up	Cytoplasm	kinase

PIK3R1	phosphoinositide-3-kinase regulatory subunit 1	3	Up	Cytoplasm	kinase
PIK3R2	phosphoinositide-3-kinase regulatory subunit 2	6	Up	Cytoplasm	kinase
PRKACA	protein kinase cAMP-activated catalytic subunit alpha	3	Up	Cytoplasm	kinase
PRKCZ	protein kinase C zeta	3	Up	Cytoplasm	kinase
PTK2	protein tyrosine kinase 2	3	Up	Cytoplasm	kinase
PTPN11	protein tyrosine phosphatase, non-receptor type 11	3	Up	Cytoplasm	phosphatase
RAF1	Raf-1 proto-oncogene, serine/threonine kinase	8	Up	Cytoplasm	kinase
RASA1	RAS p21 protein activator 1	4	Down	Cytoplasm	transporter
RPS6KB2	ribosomal protein S6 kinase B2	4	Up	Cytoplasm	kinase
SFN	stratifin	3		Cytoplasm	other
SHC1	SHC adaptor protein 1	5	Up	Cytoplasm	other
SOCS6	suppressor of cytokine signaling 6	3	Down	Cytoplasm	other
STAT3	signal transducer and activator of transcription 3	6	Up	Nucleus	transcription regulator
YWHAB	tyrosine 3-monooxygenase/tryptophan 5-monooxygenase activa	6		Cytoplasm	transcription regulator
YWHAE	tyrosine 3-monooxygenase/tryptophan 5-monooxygenase activa	11		Cytoplasm	other
YWHAG	tyrosine 3-monooxygenase/tryptophan 5-monooxygenase activa	9		Cytoplasm	other
YWHAH	tyrosine 3-monooxygenase/tryptophan 5-monooxygenase activa	7		Cytoplasm	transcription regulator
YWHAQ	tyrosine 3-monooxygenase/tryptophan 5-monooxygenase activa	14		Cytoplasm	other
YWHAZ	tyrosine 3-monooxygenase/tryptophan 5-monooxygenase activa	16		Cytoplasm	enzyme

HGF SIGNALING

Symbol	Entrez Gene Name	Expr Other	Expected	Location	Type(s)
AKT1	AKT serine/threonine kinase 1	7	Up	Cytoplasm	kinase
ATF2	activating transcription factor 2	3	Up	Nucleus	transcription regulator
ATM	ATM serine/threonine kinase	5	Up	Nucleus	kinase
CCND1	cyclin D1	6	Up	Nucleus	transcription regulator
CDC42	cell division cycle 42	4	Up	Cytoplasm	enzyme
CDK2	cyclin dependent kinase 2	10	Up	Nucleus	kinase
CDKN1A	cyclin dependent kinase inhibitor 1A	6	Down	Nucleus	kinase
CDKN2A	cyclin dependent kinase inhibitor 2A	3	Down	Nucleus	transcription regulator
CRKL	CRK like proto-oncogene, adaptor protein	4	Up	Cytoplasm	kinase
ELF3	E74 like ETS transcription factor 3	3		Nucleus	transcription regulator
ETS1	ETS proto-oncogene 1, transcription factor	3		Nucleus	transcription regulator
FGFR1	fibroblast growth factor receptor 1	4	Up	Plasma Membrane	kinase
FOS	Fos proto-oncogene, AP-1 transcription factor subunit	7	Up	Nucleus	transcription regulator
GRB2	growth factor receptor bound protein 2	19	Up	Cytoplasm	kinase
IL6	interleukin 6	4	Up	Extracellular Space	cytokine
ITGA4	integrin subunit alpha 4	11	Up	Plasma Membrane	transmembrane receptor
ITGB1	integrin subunit beta 1	4	Up	Plasma Membrane	transmembrane receptor

JUN	Jun proto-oncogene, AP-1 transcription factor subunit	12	Up	Nucleus	transcription regulator
MAP2K1	mitogen-activated protein kinase kinase 1	3	Up	Cytoplasm	kinase
MAP3K3	mitogen-activated protein kinase kinase kinase 3	5	Up	Cytoplasm	kinase
MAP3K5	mitogen-activated protein kinase kinase kinase 5	4	Up	Cytoplasm	kinase
MAP3K7	mitogen-activated protein kinase kinase kinase 7	3	Up	Cytoplasm	kinase
MAPK1	mitogen-activated protein kinase 1	9	Up	Cytoplasm	kinase
MAPK3	mitogen-activated protein kinase 3	16	Up	Cytoplasm	kinase
MAPK8	mitogen-activated protein kinase 8	5	Up	Cytoplasm	kinase
PIK3R1	phosphoinositide-3-kinase regulatory subunit 1	3	Up	Cytoplasm	kinase
PIK3R2	phosphoinositide-3-kinase regulatory subunit 2	6	Up	Cytoplasm	kinase
PLCG1	phospholipase C gamma 1	4	Up	Cytoplasm	enzyme
PLCG2	phospholipase C gamma 2	3	Up	Cytoplasm	enzyme
PRKCA	protein kinase C alpha	3	Up	Cytoplasm	kinase
PRKCB	protein kinase C beta	5	Up	Cytoplasm	kinase
PRKCD	protein kinase C delta	3	Up	Cytoplasm	kinase
PRKCG	protein kinase C gamma	5	Up	Cytoplasm	kinase
PRKCZ	protein kinase C zeta	3	Up	Cytoplasm	kinase
PTGS2	prostaglandin-endoperoxide synthase 2	3		Cytoplasm	enzyme
PTK2	protein tyrosine kinase 2	3	Up	Cytoplasm	kinase
PTPN11	protein tyrosine phosphatase, non-receptor type 11	3	Up	Cytoplasm	phosphatase
RAC1	Rac family small GTPase 1	6	Up	Plasma Membrane	enzyme
RAF1	Raf-1 proto-oncogene, serine/threonine kinase	8	Up	Cytoplasm	kinase
STAT3	signal transducer and activator of transcription 3	6	Up	Nucleus	transcription regulator

EGF SIGNALING

Symbol	Entrez Gene Name	Expr Other	Expected	Location	Type(s)
AKT1	AKT serine/threonine kinase 1	7	Up	Cytoplasm	kinase
ATM	ATM serine/threonine kinase	5	Up	Nucleus	kinase
CSNK2A1	casein kinase 2 alpha 1	6	Up	Nucleus	kinase
CSNK2B	casein kinase 2 beta	4	Up	Cytoplasm	kinase
EGFR	epidermal growth factor receptor	18	Up	Plasma Membrane	kinase
FGFR1	fibroblast growth factor receptor 1	4	Up	Plasma Membrane	kinase
FOS	Fos proto-oncogene, AP-1 transcription factor subunit	7	Up	Nucleus	transcription regulator
GRB2	growth factor receptor bound protein 2	19	Up	Cytoplasm	kinase
ITPR1	inositol 1,4,5-trisphosphate receptor type 1	6	Up	Cytoplasm	ion channel
ITPR2	inositol 1,4,5-trisphosphate receptor type 2	3	Up	Cytoplasm	ion channel
JUN	Jun proto-oncogene, AP-1 transcription factor subunit	12	Up	Nucleus	transcription regulator
MAP2K1	mitogen-activated protein kinase kinase 1	3	Up	Cytoplasm	kinase
MAPK1	mitogen-activated protein kinase 1	9	Up	Cytoplasm	kinase
MAPK3	mitogen-activated protein kinase 3	16	Up	Cytoplasm	kinase
MAPK8	mitogen-activated protein kinase 8	5	Up	Cytoplasm	kinase

MAPK13	mitogen-activated protein kinase 13	7	Up	Cytoplasm	kinase
MTOR	mechanistic target of rapamycin kinase	3	Up	Nucleus	kinase
PIK3R1	phosphoinositide-3-kinase regulatory subunit 1	3	Up	Cytoplasm	kinase
PIK3R2	phosphoinositide-3-kinase regulatory subunit 2	6	Up	Cytoplasm	kinase
PLCG1	phospholipase C gamma 1	4	Up	Cytoplasm	enzyme
PRKCA	protein kinase C alpha	3	Up	Cytoplasm	kinase
PTPN11	protein tyrosine phosphatase, non-receptor type 11	3	Up	Cytoplasm	phosphatase
RAF1	Raf-1 proto-oncogene, serine/threonine kinase	8	Up	Cytoplasm	kinase
RASA1	RAS p21 protein activator 1	4	Down	Cytoplasm	transporter
SHC1	SHC adaptor protein 1	5	Up	Cytoplasm	other
SRC	SRC proto-oncogene, non-receptor tyrosine kinase	10	Up	Cytoplasm	kinase
STAT1	signal transducer and activator of transcription 1	9	Up	Nucleus	transcription regulator
STAT3	signal transducer and activator of transcription 3	6	Up	Nucleus	transcription regulator

INSULIN RECEPTOR SIGNALING

Symbol	Entrez Gene Name	Expr Other	Expected	Location	Type(s)
AKT1	AKT serine/threonine kinase 1	7	Up	Cytoplasm	kinase
ATM	ATM serine/threonine kinase	5	Up	Nucleus	kinase
CBL	Cbl proto-oncogene	4	Up	Nucleus	transcription regulator
CRK	CRK proto-oncogene, adaptor protein	5	Up	Cytoplasm	other
CRKL	CRK like proto-oncogene, adaptor protein	4	Up	Cytoplasm	kinase
EIF2B1	eukaryotic translation initiation factor 2B subunit alpha	3	Up	Cytoplasm	translation regulator
EIF2B2	eukaryotic translation initiation factor 2B subunit beta	3	Up	Cytoplasm	other
FGFR1	fibroblast growth factor receptor 1	4	Up	Plasma Membrane	kinase
FOXO1	forkhead box O1	4	Down	Nucleus	transcription regulator
FOXO3	forkhead box O3	3	Down	Nucleus	transcription regulator
FOXO4	forkhead box O4	4	Down	Nucleus	transcription regulator
FYN	FYN proto-oncogene, Src family tyrosine kinase	7	Up	Plasma Membrane	kinase
GRB2	growth factor receptor bound protein 2	19	Up	Cytoplasm	kinase
GRB10	growth factor receptor bound protein 10	3	Up	Cytoplasm	other
GSK3B	glycogen synthase kinase 3 beta	11	Down	Nucleus	kinase
INSR	insulin receptor	6	Up	Plasma Membrane	kinase
MAP2K1	mitogen-activated protein kinase kinase 1	3	Up	Cytoplasm	kinase
MAPK1	mitogen-activated protein kinase 1	9	Up	Cytoplasm	kinase
MAPK3	mitogen-activated protein kinase 3	16	Up	Cytoplasm	kinase
MAPK8	mitogen-activated protein kinase 8	5	Down	Cytoplasm	kinase
MTOR	mechanistic target of rapamycin kinase	3	Up	Nucleus	kinase
NCK1	NCK adaptor protein 1	5	Up	Cytoplasm	kinase
PDPK1	3-phosphoinositide dependent protein kinase 1	3	Up	Cytoplasm	kinase
PIK3R1	phosphoinositide-3-kinase regulatory subunit 1	3	Up	Cytoplasm	kinase
PIK3R2	phosphoinositide-3-kinase regulatory subunit 2	6	Up	Cytoplasm	kinase

PPP1CA	protein phosphatase 1 catalytic subunit alpha	8	Up	Cytoplasm	phosphatase
PPP1CB	protein phosphatase 1 catalytic subunit beta	4	Up	Cytoplasm	phosphatase
PPP1CC	protein phosphatase 1 catalytic subunit gamma	6	Up	Nucleus	phosphatase
PPP1R12A	protein phosphatase 1 regulatory subunit 12A	3	Up	Cytoplasm	phosphatase
PRKACA	protein kinase cAMP-activated catalytic subunit alpha	3	Down	Cytoplasm	kinase
PRKCZ	protein kinase C zeta	3	Up	Cytoplasm	kinase
PTEN	phosphatase and tensin homolog	6	Down	Cytoplasm	phosphatase
PTPN11	protein tyrosine phosphatase, non-receptor type 11	3	Up	Cytoplasm	phosphatase
RAF1	Raf-1 proto-oncogene, serine/threonine kinase	8	Up	Cytoplasm	kinase
RPS6KB2	ribosomal protein S6 kinase B2	4	Up	Cytoplasm	kinase
SGK1	serum/glucocorticoid regulated kinase 1	3	Up	Cytoplasm	kinase
SHC1	SHC adaptor protein 1	5	Up	Cytoplasm	other
STX4	syntaxin 4	3		Plasma Membrane	transporter
VAMP2	vesicle associated membrane protein 2	4	Up	Plasma Membrane	other

PDGF SIGNALING

Symbol	Entrez Gene Name	Expr Other	Expected	Location	Type(s)
ABL1	ABL proto-oncogene 1, non-receptor tyrosine kinase	6	Up	Nucleus	kinase
ATM	ATM serine/threonine kinase	5	Up	Nucleus	kinase
CAV1	caveolin 1	5	Down	Plasma Membrane	transmembrane receptor
CRK	CRK proto-oncogene, adaptor protein	5	Up	Cytoplasm	other
CRKL	CRK like proto-oncogene, adaptor protein	4	Up	Cytoplasm	kinase
CSNK2A1	casein kinase 2 alpha 1	6	Up	Nucleus	kinase
CSNK2B	casein kinase 2 beta	4	Up	Cytoplasm	kinase
EIF2AK2	eukaryotic translation initiation factor 2 alpha kinase 2	3	Up	Cytoplasm	kinase
FGFR1	fibroblast growth factor receptor 1	4	Up	Plasma Membrane	kinase
FOS	Fos proto-oncogene, AP-1 transcription factor subunit	7	Up	Nucleus	transcription regulator
GRB2	growth factor receptor bound protein 2	19	Up	Cytoplasm	kinase
JUN	Jun proto-oncogene, AP-1 transcription factor subunit	12	Up	Nucleus	transcription regulator
MAP2K1	mitogen-activated protein kinase kinase 1	3	Up	Cytoplasm	kinase
MAPK1	mitogen-activated protein kinase 1	9	Up	Cytoplasm	kinase
MAPK3	mitogen-activated protein kinase 3	16	Up	Cytoplasm	kinase
MAPK8	mitogen-activated protein kinase 8	5	Up	Cytoplasm	kinase
MYC	MYC proto-oncogene, bHLH transcription factor	18	Up	Nucleus	transcription regulator
PDGFRA	platelet derived growth factor receptor alpha	3	Up	Plasma Membrane	kinase
PIK3R1	phosphoinositide-3-kinase regulatory subunit 1	3	Up	Cytoplasm	kinase
PIK3R2	phosphoinositide-3-kinase regulatory subunit 2	6	Up	Cytoplasm	kinase
PLCG1	phospholipase C gamma 1	4	Up	Cytoplasm	enzyme
PLCG2	phospholipase C gamma 2	3	Up	Cytoplasm	enzyme
PRKCA	protein kinase C alpha	3	Up	Cytoplasm	kinase

PRKCB	protein kinase C beta	5	Up	Cytoplasm	kinase
PTPN11	protein tyrosine phosphatase, non-receptor type 11	3	Up	Cytoplasm	phosphatase
RAF1	Raf-1 proto-oncogene, serine/threonine kinase	8	Up	Cytoplasm	kinase
RASA1	RAS p21 protein activator 1	4	Down	Cytoplasm	transporter
SHC1	SHC adaptor protein 1	5	Up	Cytoplasm	other
SRC	SRC proto-oncogene, non-receptor tyrosine kinase	10	Up	Cytoplasm	kinase
STAT1	signal transducer and activator of transcription 1	9	Up	Nucleus	transcription regulator
STAT3	signal transducer and activator of transcription 3	6	Up	Nucleus	transcription regulator

TGFb SIGNALING

Symbol	Entrez Gene Name	Expr Other	Expected	Location	Type(s)
BCL2	BCL2, apoptosis regulator	6		Cytoplasm	transporter
BMPR1A	bone morphogenetic protein receptor type 1A	5	Up	Plasma Membrane	kinase
CDC42	cell division cycle 42	4	Up	Cytoplasm	enzyme
CREBBP	CREB binding protein	8	Up	Nucleus	transcription regulator
EP300	E1A binding protein p300	12	Up	Nucleus	transcription regulator
FOS	Fos proto-oncogene, AP-1 transcription factor subunit	7	Up	Nucleus	transcription regulator
GRB2	growth factor receptor bound protein 2	19	Up	Cytoplasm	kinase
HDAC1	histone deacetylase 1	14	Up	Nucleus	transcription regulator
HNF4A	hepatocyte nuclear factor 4 alpha	18	Up	Nucleus	transcription regulator
JUN	Jun proto-oncogene, AP-1 transcription factor subunit	12	Up	Nucleus	transcription regulator
MAP2K1	mitogen-activated protein kinase kinase 1	3	Up	Cytoplasm	kinase
MAP3K7	mitogen-activated protein kinase kinase kinase 7	3	Up	Cytoplasm	kinase
MAPK1	mitogen-activated protein kinase 1	9	Up	Cytoplasm	kinase
MAPK3	mitogen-activated protein kinase 3	16	Up	Cytoplasm	kinase
MAPK8	mitogen-activated protein kinase 8	5	Up	Cytoplasm	kinase
MAPK13	mitogen-activated protein kinase 13	7	Up	Cytoplasm	kinase
PIAS4	protein inhibitor of activated STAT 4	5		Nucleus	transcription regulator
RAF1	Raf-1 proto-oncogene, serine/threonine kinase	8	Up	Cytoplasm	kinase
RUNX2	runt related transcription factor 2	3	Up	Nucleus	transcription regulator
SKI	SKI proto-oncogene	4	Down	Nucleus	transcription regulator
SMAD1	SMAD family member 1	7	Up	Nucleus	transcription regulator
SMAD2	SMAD family member 2	6	Up	Nucleus	transcription regulator
SMAD3	SMAD family member 3	5	Up	Nucleus	transcription regulator
SMAD4	SMAD family member 4	4	Up	Nucleus	transcription regulator
SMAD6	SMAD family member 6	3	Down	Nucleus	transcription regulator
SMAD7	SMAD family member 7	3	Down	Nucleus	transcription regulator
SMURF1	SMAD specific E3 ubiquitin protein ligase 1	6	Down	Cytoplasm	enzyme
TGFB1	transforming growth factor beta 1	3	Up	Extracellular Space	growth factor
TRAF6	TNF receptor associated factor 6	11	Up	Cytoplasm	enzyme

GNRH SIGNALING					
**Symbol**	**Entrez Gene Name**	**Expr Other**	**Expected**	**Location**	**Type(s)**
ATF2	activating transcription factor 2	3	Up	Nucleus	transcription regulator
ATF4	activating transcription factor 4	6	Up	Nucleus	transcription regulator
CACNA1A	calcium voltage-gated channel subunit alpha1 A	9		Plasma Membrane	ion channel
CALM1 (include	calmodulin 1	9		Cytoplasm	other
CAMK2A	calcium/calmodulin dependent protein kinase II alpha	5	Up	Cytoplasm	kinase
CDC42	cell division cycle 42	4	Up	Cytoplasm	enzyme
CREB1	cAMP responsive element binding protein 1	11	Up	Nucleus	transcription regulator
CREBBP	CREB binding protein	8	Up	Nucleus	transcription regulator
EGFR	epidermal growth factor receptor	18	Up	Plasma Membrane	kinase
EGR1	early growth response 1	5	Up	Nucleus	transcription regulator
EP300	E1A binding protein p300	12	Up	Nucleus	transcription regulator
FOS	Fos proto-oncogene, AP-1 transcription factor subunit	7	Up	Nucleus	transcription regulator
GNB1	G protein subunit beta 1	3	Up	Plasma Membrane	enzyme
GRB2	growth factor receptor bound protein 2	19	Up	Cytoplasm	kinase
HBEGF	heparin binding EGF like growth factor	4	Up	Extracellular Space	growth factor
ITPR1	inositol 1,4,5-trisphosphate receptor type 1	6	Up	Cytoplasm	ion channel
ITPR2	inositol 1,4,5-trisphosphate receptor type 2	3	Up	Cytoplasm	ion channel
JUN	Jun proto-oncogene, AP-1 transcription factor subunit	12	Up	Nucleus	transcription regulator
MAP2K1	mitogen-activated protein kinase kinase 1	3	Up	Cytoplasm	kinase
MAP3K3	mitogen-activated protein kinase kinase kinase 3	5	Up	Cytoplasm	kinase
MAP3K5	mitogen-activated protein kinase kinase kinase 5	4	Up	Cytoplasm	kinase
MAP3K7	mitogen-activated protein kinase kinase kinase 7	3	Up	Cytoplasm	kinase
MAPK1	mitogen-activated protein kinase 1	9	Up	Cytoplasm	kinase
MAPK3	mitogen-activated protein kinase 3	16	Up	Cytoplasm	kinase
MAPK8	mitogen-activated protein kinase 8	5	Up	Cytoplasm	kinase
MAPK13	mitogen-activated protein kinase 13	7	Up	Cytoplasm	kinase
NFKB1	nuclear factor kappa B subunit 1	6	Down	Nucleus	transcription regulator
NFKB2	nuclear factor kappa B subunit 2	4	Down	Nucleus	transcription regulator
PLCB1	phospholipase C beta 1	4	Up	Cytoplasm	enzyme
PRKACA	protein kinase cAMP-activated catalytic subunit alpha	3	Up	Cytoplasm	kinase
PRKCA	protein kinase C alpha	3	Up	Cytoplasm	kinase
PRKCB	protein kinase C beta	5	Up	Cytoplasm	kinase
PRKCD	protein kinase C delta	3	Up	Cytoplasm	kinase
PRKCG	protein kinase C gamma	5	Up	Cytoplasm	kinase
PRKCZ	protein kinase C zeta	3	Up	Cytoplasm	kinase
PTK2	protein tyrosine kinase 2	3	Up	Cytoplasm	kinase
PTK2B	protein tyrosine kinase 2 beta	3	Up	Cytoplasm	kinase
RAC1	Rac family small GTPase 1	6	Up	Plasma Membrane	enzyme

RAF1	Raf-1 proto-oncogene, serine/threonine kinase	8	Up	Cytoplasm	kinase
RELA	RELA proto-oncogene, NF-kB subunit	10	Down	Nucleus	transcription regulator
SRC	SRC proto-oncogene, non-receptor tyrosine kinase	10	Up	Cytoplasm	kinase

PEDF SIGNALING

Symbol	Entrez Gene Name	Expr Other	Expected	Location	Type(s)
AKT1	AKT serine/threonine kinase 1	7	Up	Cytoplasm	kinase
ATM	ATM serine/threonine kinase	5	Up	Nucleus	kinase
BCL2	BCL2, apoptosis regulator	6	Down	Cytoplasm	transporter
BCL2L1	BCL2 like 1	4	Down	Cytoplasm	other
CASP7	caspase 7	7	Up	Cytoplasm	peptidase
CASP8	caspase 8	5	Up	Nucleus	peptidase
FAS	Fas cell surface death receptor	3	Up	Plasma Membrane	transmembrane receptor
FGFR1	fibroblast growth factor receptor 1	4	Up	Plasma Membrane	kinase
GRB2	growth factor receptor bound protein 2	19	Up	Cytoplasm	kinase
HNF1A	HNF1 homeobox A	5	Up	Nucleus	transcription regulator
IKBKE	inhibitor of nuclear factor kappa B kinase subunit epsilon	4	Up	Cytoplasm	kinase
IKBKG	inhibitor of nuclear factor kappa B kinase subunit gamma	4	Up	Nucleus	kinase
MAPK1	mitogen-activated protein kinase 1	9	Up	Cytoplasm	kinase
MAPK3	mitogen-activated protein kinase 3	16	Up	Cytoplasm	kinase
MAPK13	mitogen-activated protein kinase 13	7	Up	Cytoplasm	kinase
NFKB1	nuclear factor kappa B subunit 1	6	Up	Nucleus	transcription regulator
NFKB2	nuclear factor kappa B subunit 2	4	Up	Nucleus	transcription regulator
NFKBIA	NFKB inhibitor alpha	14		Cytoplasm	transcription regulator
PIK3R1	phosphoinositide-3-kinase regulatory subunit 1	3	Up	Cytoplasm	kinase
PIK3R2	phosphoinositide-3-kinase regulatory subunit 2	6	Up	Cytoplasm	kinase
PPARG	peroxisome proliferator activated receptor gamma	6	Up	Nucleus	lig-dependent nuc. receptor
PTPN11	protein tyrosine phosphatase, non-receptor type 11	3	Up	Cytoplasm	phosphatase
RAC1	Rac family small GTPase 1	6	Up	Plasma Membrane	enzyme
RAF1	Raf-1 proto-oncogene, serine/threonine kinase	8	Up	Cytoplasm	kinase
RELA	RELA proto-oncogene, NF-kB subunit	10	Up	Nucleus	transcription regulator
SOD2	superoxide dismutase 2	3	Down	Cytoplasm	enzyme
TCF7	transcription factor 7	3	Up	Nucleus	transcription regulator
TCF7L2	transcription factor 7 like 2	5	Up	Nucleus	transcription regulator
TP53	tumor protein p53	26	Up	Nucleus	transcription regulator

GROWTH HORMONE SIGNALING

Symbol	Entrez Gene Name	Expr Other	Expected	Location	Type(s)
A2M	alpha-2-macroglobulin	4	Up	Extracellular Space	transporter

ATM	ATM serine/threonine kinase	5	Up	Nucleus	kinase
CEBPA	CCAAT/enhancer binding protein alpha	7	Up	Nucleus	transcription regulator
FGFR1	fibroblast growth factor receptor 1	4	Up	Plasma Membrane	kinase
FOS	Fos proto-oncogene, AP-1 transcription factor subunit	7	Up	Nucleus	transcription regulator
GRB2	growth factor receptor bound protein 2	19	Up	Cytoplasm	kinase
MAPK1	mitogen-activated protein kinase 1	9	Up	Cytoplasm	kinase
MAPK3	mitogen-activated protein kinase 3	16	Up	Cytoplasm	kinase
ONECUT1	one cut homeobox 1	5		Nucleus	transcription regulator
PDPK1	3-phosphoinositide dependent protein kinase 1	3	Up	Cytoplasm	kinase
PIK3R1	phosphoinositide-3-kinase regulatory subunit 1	3	Up	Cytoplasm	kinase
PIK3R2	phosphoinositide-3-kinase regulatory subunit 2	6	Up	Cytoplasm	kinase
PLCG1	phospholipase C gamma 1	4	Up	Cytoplasm	enzyme
PLCG2	phospholipase C gamma 2	3	Up	Cytoplasm	enzyme
PRKCA	protein kinase C alpha	3	Up	Cytoplasm	kinase
PRKCB	protein kinase C beta	5	Up	Cytoplasm	kinase
PRKCD	protein kinase C delta	3	Up	Cytoplasm	kinase
PRKCG	protein kinase C gamma	5	Up	Cytoplasm	kinase
PRKCZ	protein kinase C zeta	3	Up	Cytoplasm	kinase
PTPN11	protein tyrosine phosphatase, non-receptor type 11	3	Up	Cytoplasm	phosphatase
RPS6KA1	ribosomal protein S6 kinase A1	3	Up	Cytoplasm	kinase
RPS6KB2	ribosomal protein S6 kinase B2	4	Up	Cytoplasm	kinase
SOCS6	suppressor of cytokine signaling 6	3	Down	Cytoplasm	other
STAT1	signal transducer and activator of transcription 1	9	Up	Nucleus	transcription regulator
STAT3	signal transducer and activator of transcription 3	6	Up	Nucleus	transcription regulator

NEUROTROPHIN/TRK SIGNALING

Symbol	Entrez Gene Name	Expr Other	Expected	Location	Type(s)
AKT1	AKT serine/threonine kinase 1	7	Up	Cytoplasm	kinase
ATF2	activating transcription factor 2	3	Up	Nucleus	transcription regulator
ATF4	activating transcription factor 4	6	Up	Nucleus	transcription regulator
ATM	ATM serine/threonine kinase	5	Up	Nucleus	kinase
CDC42	cell division cycle 42	4	Up	Cytoplasm	enzyme
CREB1	cAMP responsive element binding protein 1	11	Up	Nucleus	transcription regulator
CREBBP	CREB binding protein	8	Up	Nucleus	transcription regulator
EP300	E1A binding protein p300	12	Up	Nucleus	transcription regulator
FGFR1	fibroblast growth factor receptor 1	4	Up	Plasma Membrane	kinase
FOS	Fos proto-oncogene, AP-1 transcription factor subunit	7	Up	Nucleus	transcription regulator
GRB2	growth factor receptor bound protein 2	19	Up	Cytoplasm	kinase

JUN	Jun proto-oncogene, AP-1 transcription factor subunit	12	Up	Nucleus	transcription regulator
MAP2K1	mitogen-activated protein kinase kinase 1	3	Up	Cytoplasm	kinase
MAP3K5	mitogen-activated protein kinase kinase kinase 5	4	Up	Cytoplasm	kinase
MAPK1	mitogen-activated protein kinase 1	9	Up	Cytoplasm	kinase
MAPK3	mitogen-activated protein kinase 3	16	Up	Cytoplasm	kinase
MAPK8	mitogen-activated protein kinase 8	5	Up	Cytoplasm	kinase
NGFR	nerve growth factor receptor	5	Up	Plasma Membrane	transmembrane receptor
NTRK1	neurotrophic receptor tyrosine kinase 1	26		Plasma Membrane	kinase
PDPK1	3-phosphoinositide dependent protein kinase 1	3	Up	Cytoplasm	kinase
PIK3R1	phosphoinositide-3-kinase regulatory subunit 1	3	Up	Cytoplasm	kinase
PIK3R2	phosphoinositide-3-kinase regulatory subunit 2	6	Up	Cytoplasm	kinase
PLCG1	phospholipase C gamma 1	4	Up	Cytoplasm	enzyme
PTPN11	protein tyrosine phosphatase, non-receptor type 11	3	Up	Cytoplasm	phosphatase
RAF1	Raf-1 proto-oncogene, serine/threonine kinase	8	Up	Cytoplasm	kinase
RPS6KA1	ribosomal protein S6 kinase A1	3	Up	Cytoplasm	kinase
SHC1	SHC adaptor protein 1	5	Up	Cytoplasm	other

FGF SIGNALING

Symbol	Entrez Gene Name	Expr Other	Expected	Location	Type(s)
AKT1	AKT serine/threonine kinase 1	7	Up	Cytoplasm	kinase
ATF2	activating transcription factor 2	3	Up	Nucleus	transcription regulator
ATF4	activating transcription factor 4	6	Up	Nucleus	transcription regulator
ATM	ATM serine/threonine kinase	5	Up	Nucleus	kinase
CREB1	cAMP responsive element binding protein 1	11	Up	Nucleus	transcription regulator
CREBBP	CREB binding protein	8	Up	Nucleus	transcription regulator
CRK	CRK proto-oncogene, adaptor protein	5	Up	Cytoplasm	other
CRKL	CRK like proto-oncogene, adaptor protein	4	Up	Cytoplasm	kinase
EP300	E1A binding protein p300	12	Up	Nucleus	transcription regulator
FGFR1	fibroblast growth factor receptor 1	4	Up	Plasma Membrane	kinase
GRB2	growth factor receptor bound protein 2	19	Up	Cytoplasm	kinase
ITPR1	inositol 1,4,5-trisphosphate receptor type 1	6	Up	Cytoplasm	ion channel
MAP2K1	mitogen-activated protein kinase kinase 1	3	Up	Cytoplasm	kinase
MAP3K5	mitogen-activated protein kinase kinase kinase 5	4	Up	Cytoplasm	kinase
MAPK1	mitogen-activated protein kinase 1	9	Up	Cytoplasm	kinase
MAPK3	mitogen-activated protein kinase 3	16	Up	Cytoplasm	kinase
MAPK8	mitogen-activated protein kinase 8	5	Up	Cytoplasm	kinase
MAPK13	mitogen-activated protein kinase 13	7	Up	Cytoplasm	kinase
PIK3R1	phosphoinositide-3-kinase regulatory subunit 1	3	Up	Cytoplasm	kinase
PIK3R2	phosphoinositide-3-kinase regulatory subunit 2	6	Up	Cytoplasm	kinase
PLCG1	phospholipase C gamma 1	4	Up	Cytoplasm	enzyme
PRKCA	protein kinase C alpha	3	Up	Cytoplasm	kinase

PTPN11	protein tyrosine phosphatase, non-receptor type 11	3	Up	Cytoplasm	phosphatase
RAC1	Rac family small GTPase 1	6	Up	Plasma Membrane	enzyme
RAF1	Raf-1 proto-oncogene, serine/threonine kinase	8	Up	Cytoplasm	kinase
STAT3	signal transducer and activator of transcription 3	6	Up	Nucleus	transcription regulator

GDNF SIGNALING

Symbol	Entrez Gene Name	Expr Other	Expected	Location	Type(s)
ATM	ATM serine/threonine kinase	5	Up	Nucleus	kinase
CDC42	cell division cycle 42	4	Up	Cytoplasm	enzyme
CREB1	cAMP responsive element binding protein 1	11	Up	Nucleus	transcription regulator
FGFR1	fibroblast growth factor receptor 1	4	Up	Plasma Membrane	kinase
FOS	Fos proto-oncogene, AP-1 transcription factor subunit	7	Up	Nucleus	transcription regulator
GRB2	growth factor receptor bound protein 2	19	Up	Cytoplasm	kinase
ITPR1	inositol 1,4,5-trisphosphate receptor type 1	6		Cytoplasm	ion channel
ITPR2	inositol 1,4,5-trisphosphate receptor type 2	3		Cytoplasm	ion channel
JUN	Jun proto-oncogene, AP-1 transcription factor subunit	12	Up	Nucleus	transcription regulator
MAP2K1	mitogen-activated protein kinase kinase 1	3	Up	Cytoplasm	kinase
MAPK1	mitogen-activated protein kinase 1	9	Up	Cytoplasm	kinase
MAPK3	mitogen-activated protein kinase 3	16	Up	Cytoplasm	kinase
MAPK8	mitogen-activated protein kinase 8	5	Up	Cytoplasm	kinase
NCK1	NCK adaptor protein 1	5	Up	Cytoplasm	kinase
PIK3R1	phosphoinositide-3-kinase regulatory subunit 1	3	Up	Cytoplasm	kinase
PIK3R2	phosphoinositide-3-kinase regulatory subunit 2	6	Up	Cytoplasm	kinase
PLCG1	phospholipase C gamma 1	4	Up	Cytoplasm	enzyme
PLCG2	phospholipase C gamma 2	3	Up	Cytoplasm	enzyme
PTPN11	protein tyrosine phosphatase, non-receptor type 11	3	Up	Cytoplasm	phosphatase
RAC1	Rac family small GTPase 1	6	Up	Plasma Membrane	enzyme
RAF1	Raf-1 proto-oncogene, serine/threonine kinase	8	Up	Cytoplasm	kinase
RASA1	RAS p21 protein activator 1	4		Cytoplasm	transporter
SHC1	SHC adaptor protein 1	5	Up	Cytoplasm	other

CNTF SIGNALING

Symbol	Entrez Gene Name	Expr Other	Expected	Location	Type(s)
AKT1	AKT serine/threonine kinase 1	7	Up	Cytoplasm	kinase
ATM	ATM serine/threonine kinase	5	Up	Nucleus	kinase
CNTF	ciliary neurotrophic factor	4	Up	Extracellular Space	cytokine
FGFR1	fibroblast growth factor receptor 1	4	Up	Plasma Membrane	kinase
GRB2	growth factor receptor bound protein 2	19	Up	Cytoplasm	kinase
IL6ST	interleukin 6 signal transducer	3	Up	Plasma Membrane	transmembrane receptor
MAP2K1	mitogen-activated protein kinase kinase 1	3	Up	Cytoplasm	kinase

MAPK1	mitogen-activated protein kinase 1	9	Up	Cytoplasm	kinase
MAPK3	mitogen-activated protein kinase 3	16	Up	Cytoplasm	kinase
MTOR	mechanistic target of rapamycin kinase	3	Up	Nucleus	kinase
PIK3R1	phosphoinositide-3-kinase regulatory subunit 1	3	Up	Cytoplasm	kinase
PIK3R2	phosphoinositide-3-kinase regulatory subunit 2	6	Up	Cytoplasm	kinase
PTPN11	protein tyrosine phosphatase, non-receptor type 11	3	Up	Cytoplasm	phosphatase
RAF1	Raf-1 proto-oncogene, serine/threonine kinase	8	Up	Cytoplasm	kinase
RPS6KA1	ribosomal protein S6 kinase A1	3	Up	Cytoplasm	kinase
RPS6KB2	ribosomal protein S6 kinase B2	4	Up	Cytoplasm	kinase
STAT1	signal transducer and activator of transcription 1	9	Up	Nucleus	transcription regulator
STAT3	signal transducer and activator of transcription 3	6	Up	Nucleus	transcription regulator
